# NHC Catalysis for Umpolung Pyridinium Alkylation via Deoxy‐Breslow Intermediates

**DOI:** 10.1002/anie.202117524

**Published:** 2022-02-18

**Authors:** Terence Wu, Matthew R. Tatton, Michael F. Greaney

**Affiliations:** ^1^ School of Chemistry The University of Manchester Oxford Road Manchester M13 9PL UK; ^2^ Early Chemical Development Pharmaceutical Sciences R&D, AstraZeneca Macclesfield SK10 2NA UK

**Keywords:** Deoxy-Breslow Intermediate, N-Heterocyclic Carbenes, Organocatalysis, Pyridinium Substrates, Umpolung

## Abstract

Umpolung N‐heterocyclic carbene (NHC) catalysis of non‐aldehyde substrates offers new pathways for C−C bond formation, but has proven challenging to develop in terms of viable substrate classes. Here, we demonstrate that pyridinium ions can undergo NHC addition and subsequent intramolecular C−C bond formation through a deoxy‐Breslow intermediate. The alkylation demonstrates, for the first time, that deoxy‐Breslow intermediates are viable for catalytic umpolung of areniums.

## Graphical abstract

NHC deoxy‐Breslow catalysis offers new umpolung possibilities for electron‐poor arene rings. NHC organocatalysis is largely restricted to aldehydes, with other electrophiles proving difficult to harness. It is shown that a pyridinium system can react successfully with an NHC, enabling intramolecular C−C bond formation with a Michael acceptor through a deoxy‐Breslow intermediate.

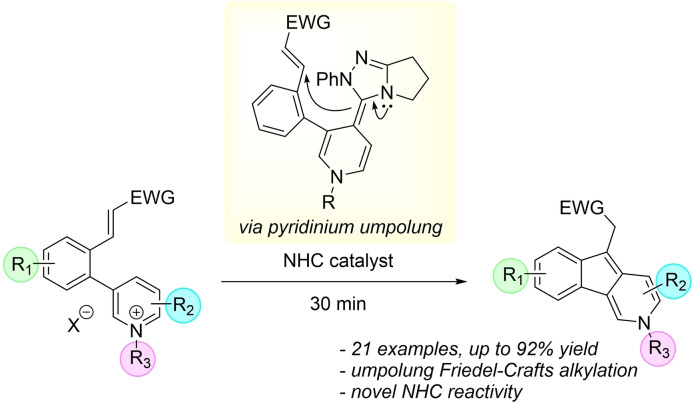

N‐Heterocyclic carbene (NHC) catalysis for aldehyde umpolung reactivity is a well‐developed area of organocatalysis, creating unique acyl anion reactivity via the Breslow intermediate **1** (Scheme 1).[^1^, ^2^] Subsequent reaction with electrophiles has been richly exemplified, and represents a fundamental carbon–carbon bond forming technology in synthesis. Catalytic NHC addition and Breslow‐type intermediate formation with other (non‐aldehyde) electrophilic acceptors, by contrast, remains far less developed.^[3]^ While the potential synthetic application of such umpolung methods is vast, capturing sufficient reactivity of the analogous Breslow intermediates has proven challenging in the catalysis regime.

**Scheme 1:**
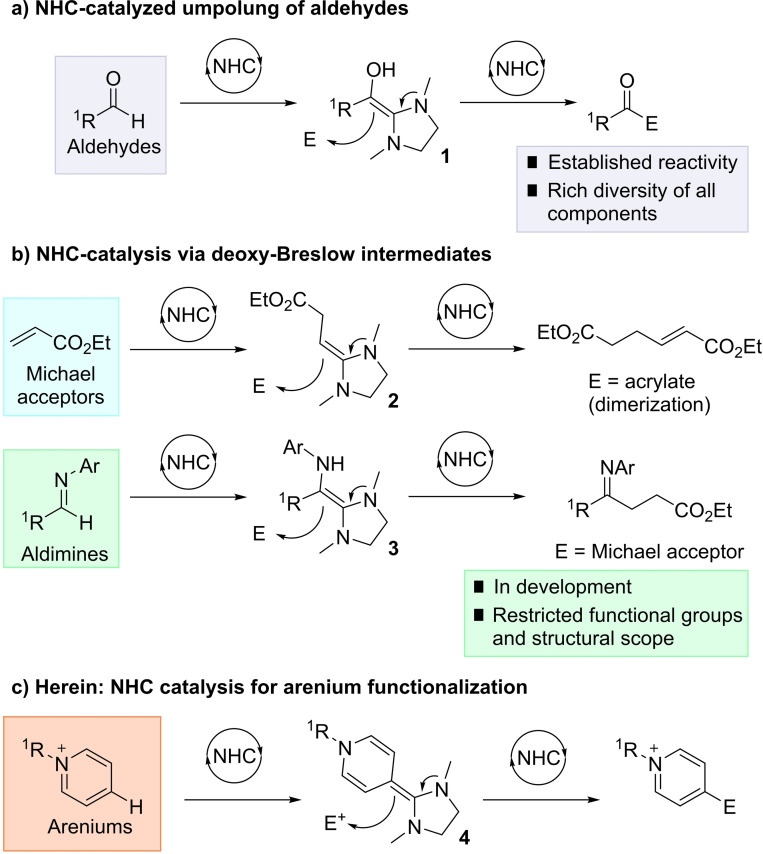
Breslow and deoxy‐Breslow intermediates for umpolung catalysis.

Seminal work from the groups of Fu, Glorius, Matsuoka, and others established that Michael acceptors such as acrylates (Scheme 1b) could successfully function as electrophiles for NHC catalysis via deoxy‐Breslow intermediates **2**.^[4]^ More recently, the groups of Biju, Suresh, and Lupton have disclosed studies on aldimines as NHC electrophiles, forming the aza‐Breslow intermediate **3** which can add to Michael acceptors.^[5]^ Despite these advances, the scope of the reaction is currently small in terms of viable substrate classes for catalytic deoxy‐Breslow formation.

In light of these pioneering contributions, we were interested in the concept of NHC addition to an arenium system as a new class of acceptor electrophile. Such a reaction would, in principle, enable umpolung reactivity where an electron‐poor arene functions as a nucleophile in a C−C bond forming reaction at a C−H position (Scheme 1c). This umpolung Friedel–Crafts reactivity, realized through organocatalysis would present new pathways for arene functionalization.

Such a system clearly raises a number of questions concerning the desired reactivity for NHC catalysis, e.g. how feasible is initial arene addition of the NHC to an arenium, and what is the reactivity of the heavily conjugated deoxy‐Breslow intermediate **4** with respect to electrophiles?^[6]^ Site selectivity of NHC addition to the arenium must also be considered, with choice of the nitrogen substituent likely a key factor. To study these questions we designed model substrate **5**, containing an alkylated pyridinium ion as the umpolung arene, tethered to a Michael acceptor electrophile. The desired catalysis cycle is set out in Scheme 2, forming the extended deoxy‐Breslow intermediate **8** on proton loss from adduct **7**, which would then enable C−C bond formation to give the cyclic pyridinium **11**, and ultimately the indenopyridine product **6**.

**Scheme 2:**
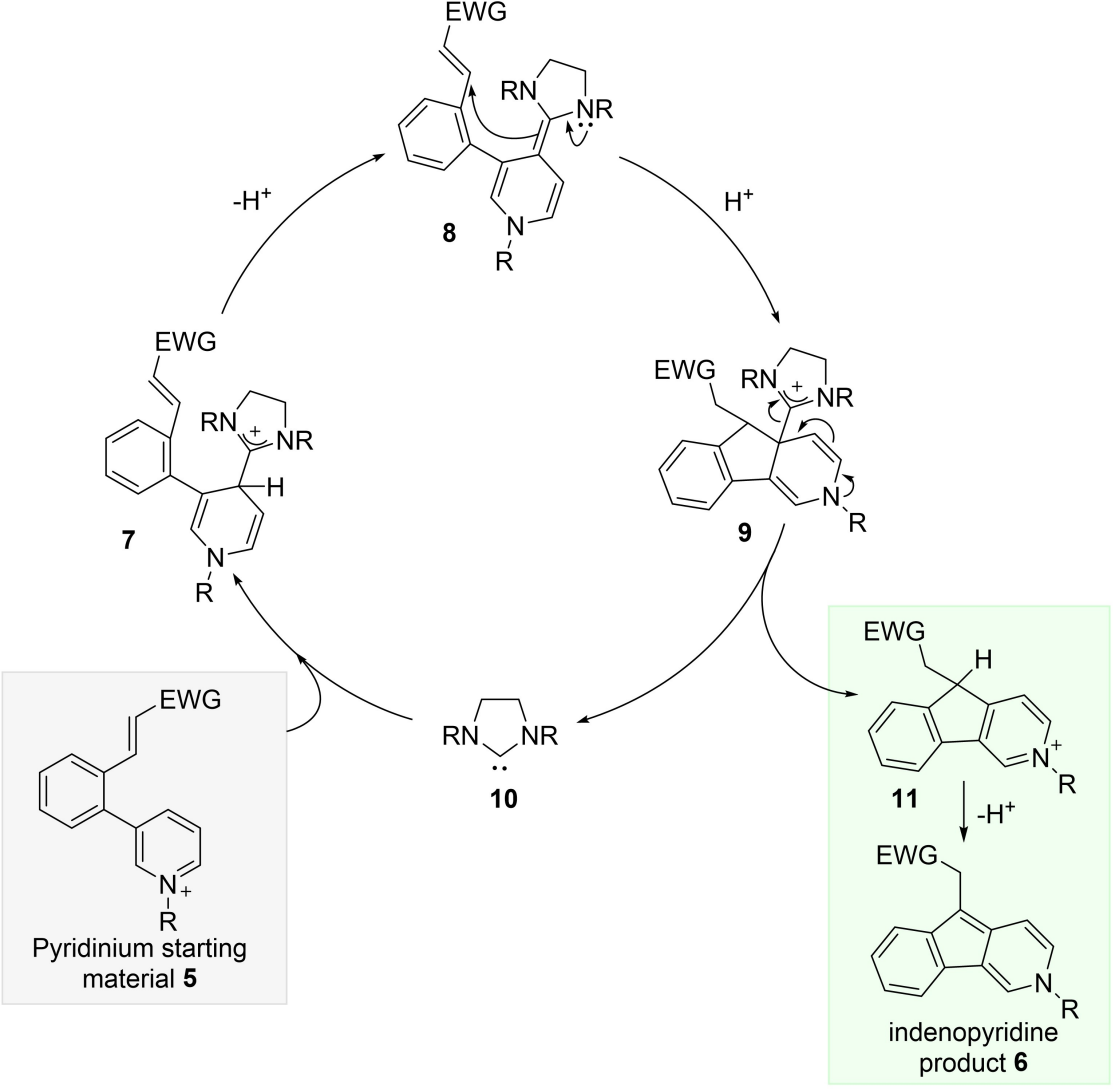
Proposed catalysis cycle for NHC addition to pyridinium **5**.

Our substrate design was influenced, first, by a report of Hansmann and co‐workers who prepared a series of organic redox reagents through stoichiometric addition of NHCs to pyridinium ions, demonstrating the feasibility of the proposed first step.^[7]^ Second, tethered intramolecular systems analogous to **5** have been effective in defining umpolung Breslow reactivity for several Michael acceptor and aldimine systems.[^4^, ^5^] Third, we were aware of a report from Rovis and co‐workers that implicated NHC addition/protonation on a pyridinium ion as an off‐cycle side‐reaction in their study of a conventional NHC‐catalyzed aldehyde addition to a pyridinium acceptor.^[8]^ Finally, the pyridine/pyridinium/dihydropyridine family of heteroarenes is very prominent in pharmaceuticals, complex natural products, and other functional molecules, making sustainable methods for C−C bond functionalization highly desirable.^[9]^ Collectively, these antecedents suggested pyridinium **5** would be a good system for probing the idea of NHC addition and arene umpolung. The starting material **5** **a** (Table 1) was prepared in good yield through a three‐step process from 3‐bromopyridine via Suzuki–Miyaura cross‐coupling, Horner–Wadsworth–Emmons reaction, and pyridine benzylation (see Supporting Information).

**Table 1:**
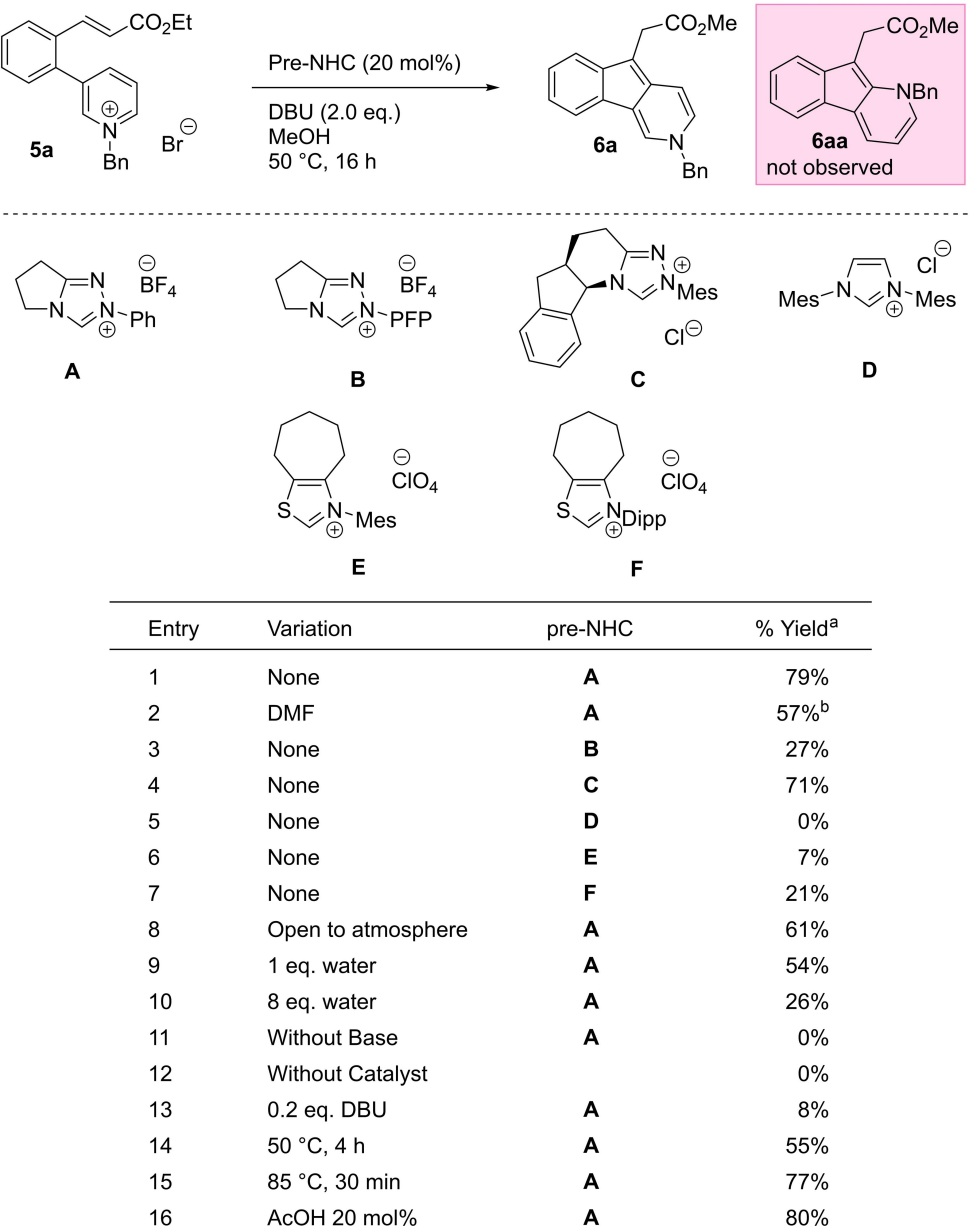
Standard conditions: **5** **a** (0.1 mmol), pre‐NHC **A** (0.02 mmol), DBU (0.2 mmol), MeOH (1.0 mL), 50 °C, 16 h.

[a] Yields of **5** **a** were determined by ^1^H NMR analysis of the crude reaction mixtures using trimethoxybenzene as an internal standard. [b] Ethyl ester product.

We began our investigations on **5** **a** using triazolium pre‐catalyst **A** and 2 equivalents of DBU in methanol, and were delighted to observe good conversion to the desired indeno[1,2‐c]pyridine **6** **a** (Table 1, entry 1) as the methyl ester resulting from transesterification with the solvent.^[10]^ The product **6** **a** was isolated as a single regio‐isomer, with no sign of **6** **aa** which could arise from C−C bond at the pyridinium 2‐position. Switching solvents to DMF allowed us to retain the ethyl ester but at a lower yield (entry 2). Triazolium precatalyst **C** was also effective in the reaction, but the nucleophilic imidazolium **D** and thiazoliums **E** and **F** were poor (entries 3–7). Further variation of base or solvent confirmed DBU and methanol as optimal (see Supporting Information). The reaction was somewhat affected by the presence of both oxygen (entry 8) and water (entries 9 and 10). Control experiments without catalyst or base (entries 11 and 12) showed complete cessation of reactivity, and sub‐stoichiometric amounts of base also led to very low yields (entry 13); unsurprisingly given that one equivalent of base is required for the off‐cycle deprotonation of pyridinium ion **11** to form product **6**. Increasing the temperature of the reaction to 85 °C allowed us to reduce the reaction times to half an hour without significantly affecting the yield (entries 14 and 15) and diluting the reaction gave a moderate improvement to yield, probably by reducing unwanted intermolecular side reactions (see Supporting Information). An additive of AcOH (entry 16) shown previously to promote the regeneration of the NHC catalyst did not significantly alter the reactivity.^[8]^

With optimal conditions established, we sought to investigate the scope of this transformation (Scheme 3). Variation of the N‐alkyl group could be tolerated (**6** **a**–**6** **f**), with both electron‐rich and electron‐poor benzyl groups being good substrates, whereas simple methyl and ethyl groups were less effective. The aryl linking architecture could be varied to install functional handles into the product indenopyridines, with methyl fluoro, methoxy, and fused arene rings successful in the reaction (**6** **g**–**6** **m**, **6** **j** structure confirmed through X‐ray analysis).^[11]^

**Scheme 3:**
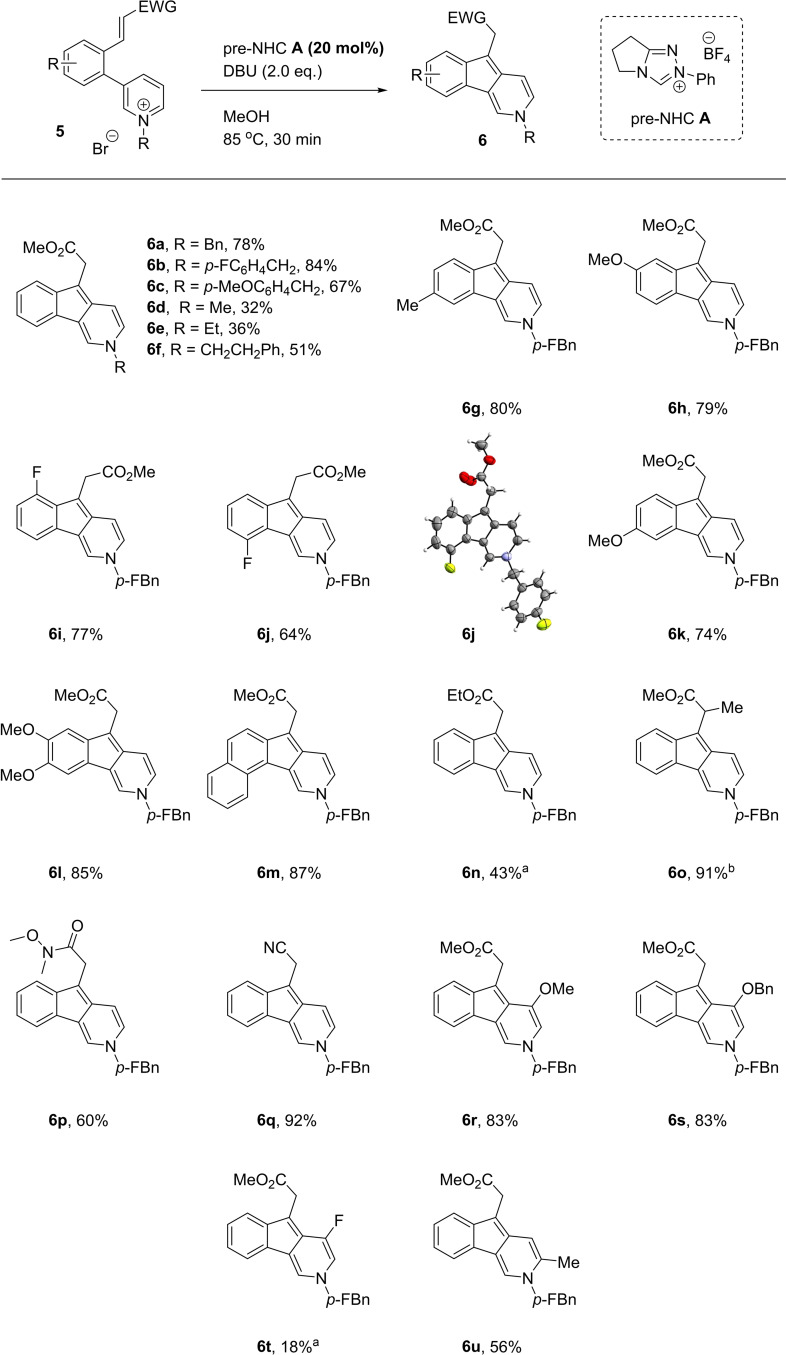
Standard conditions: Pyridinium **5** (0.10 mmol), pre‐NHC **A** (0.02 mmol), DBU (0.20 mmol), MeOH (4.0 mL), 85 °C, 30 min. [a] Reaction conducted with DMF instead of MeOH. [b] Isolated as a mixture of Me and Et esters (2 : 1). *p‐*FBn = *p*‐fluorobenzyl

Turning to the Michael acceptor, running the reaction in DMF suppressed the transesterification reaction, allowing different esters to be employed (**6** **n**). A methyl group was tolerated at the α‐position of the Michael acceptor, affording **6** **o** in an excellent 91 % yield (an important observation for the mechanism of the transformation, see below). The versatile Weinreb amide and nitrile Michael acceptors were also effective (**6** **p** and **6** **q**). Substituting the enoate group for vinyl sulfone, vinyl nitro, enone or a simple aldehyde gave little to no conversion in each case. We also examined the role of enoate geometry in the reaction, and found the *Z*‐isomer of **5** **b** to be an inferior substrate to the *E*, giving a moderate 47 % conversion to product **6** **b**.

The pyridinium ring was found to be significantly more sensitive to change. Some substitution at the 5‐position could be tolerated, with the electron‐rich methoxy and benzyloxy groups proving excellent substrates to give indenopyridines **6** **r** and **6** **s**, but simple methyl and phenyl substitution was detrimental and yielded only trace amounts of product. Halide substituents such as Cl or Br produced a complex mixture with no trace of product. When the substituent was F, under the normal reaction conditions, methoxy substituted product **6** **r** was produced, presumably through S_*N*_Ar reaction with the solvent followed by cyclization. By exchanging the solvent to DMF, a low yield of the intended indenopyridine **6** **t** was obtained. Examining substituents at other sites, it was found that methyl substituents at either the 2 or 4 position of the pyridinium ring provided no product. In the case of 4‐substitution, one might expect the C−C bond to be formed between the C‐2 carbon of the pyridinium ring instead of C‐4 to give an indeno[2,1‐b]pyridine product analogous to **6** **aa** (Table 1) but this was not observed. In both cases, the restricted rotation a methyl group in the 2 or 4 position confers on the biaryl system may contribute to their poor reactivity. Substitution at the 6‐position of the pyridinium ion was the only other tolerated site providing a moderate yield (**6** **u**).

Difficulties encountered with the quaternization of *ortho* di‐substituted pyridinium ions hindered the further exploration of substitution on the pyridinium ring. However, it appears clear that both steric and electronic effects significantly contribute to the reactivity of the pyridinium ring. The reaction was amenable to gram‐scale, with **5** **r** affording 1.06 g of **6** **r** (70 %) on a 4.0 mmol scale. Further alkylation of the indenopyridine product proved very effective, proceeding in excellent yield to give the quaternary carbon stereocenter products **12** **a**–**12** **c**. Selective reduction using DIBAL‐H was also possible, affording the primary alcohol **13** (Scheme 4).

**Scheme 4:**
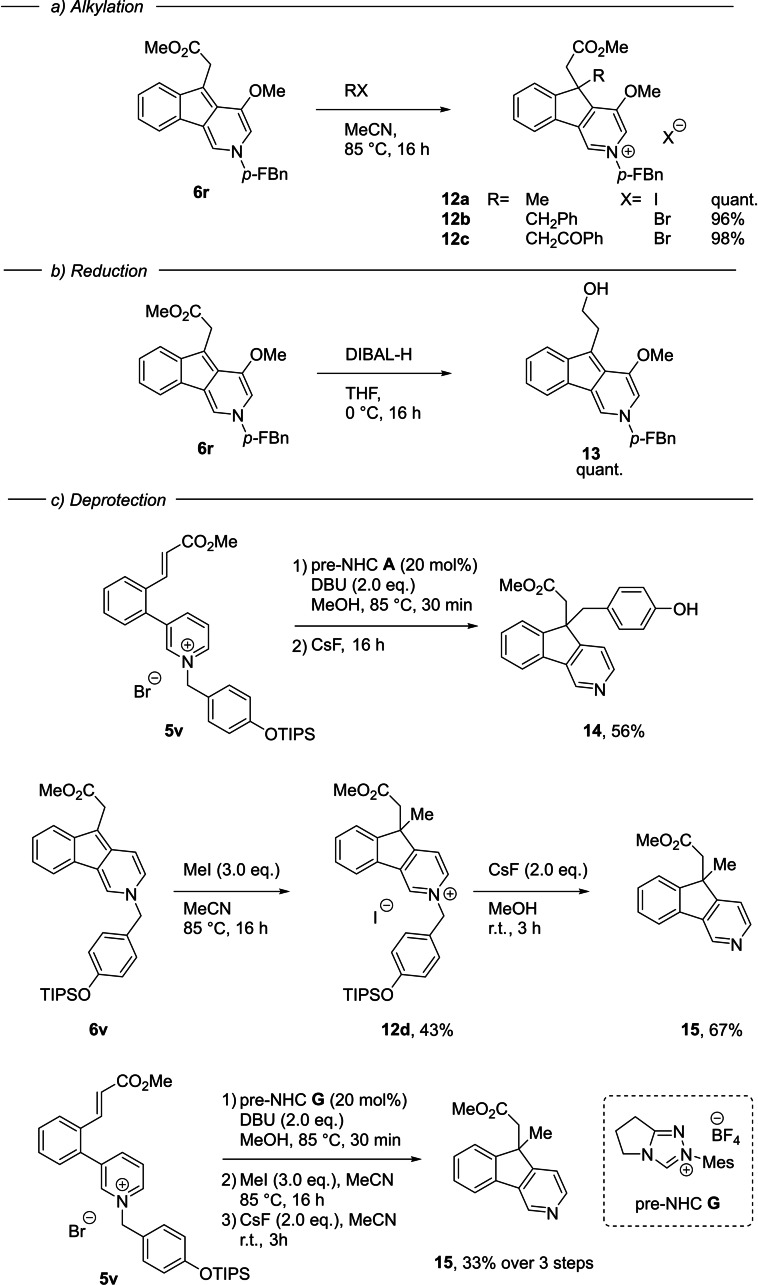
Transformation of indenopyridine products. *p‐*FBn = *p*‐fluorobenzyl.

Finally, we addressed possible deprotection strategies to access the parent pyridine heteroarenes. We synthesized pyridinium **5** **v** containing the *p*‐OTIPS benzyl group, recently developed by Donohoe and co‐workers as a cleavable activating group (cAG) in pyridine functionalization chemistry.^[12]^ NHC‐catalyzed alkylation followed by in situ fluoride treatment successfully revealed the pyridine, but with the surprising accompaniment of C‐benzylation to form **14**. The quinone methide formed on desilylative debenzylation is reacting with the extended enamine equivalent to form the cyclopentyl quaternary center. We could simplify the deprotection by alkylating **6** **v** to give the pyridinium **12** **d** containing the cAG, which could then be removed by fluoride treatment to yield pyridine **15**. This process could be done sequentially from **5** **v** without purification between each step, establishing an important pathway to pyridines from the indenopyridine series of primary alkylation products.

It is pertinent to consider, given the two electrophilic residues in the substrate **5**, whether the NHC is indeed activating the pyridinium ion as we envisaged (Scheme 2), or whether the alternative pathway of Michael addition to the enoate, and subsequent intramolecular addition to the pyridinium ring is the primary reaction mode (Scheme 5, path A). The pyridinium path B is likely operating for the following reasons: β‐Substituted Michael acceptors are rare in deoxy‐Breslow catalytic cycles,^[13]^ and tri‐substituted ones such as **5** **o** (forming **6** **o** in 91 % yield) are without precedent. To further investigate this possibility, however, we prepared the β‐substituted substrate **5** **w**. When **5** **w** was subject to the reaction conditions we observed a 27 % conversion to the product pyridinium **18** using 20 mol% NHC. A stoichiometric loading of NHC then gave **18** in 43 % isolated yield. Since the formation of a deoxy‐Breslow intermediate from the enoate is not possible, due to the β*‐*methyl group blockade, this rules out the Michael addition path A.

**Scheme 5:**
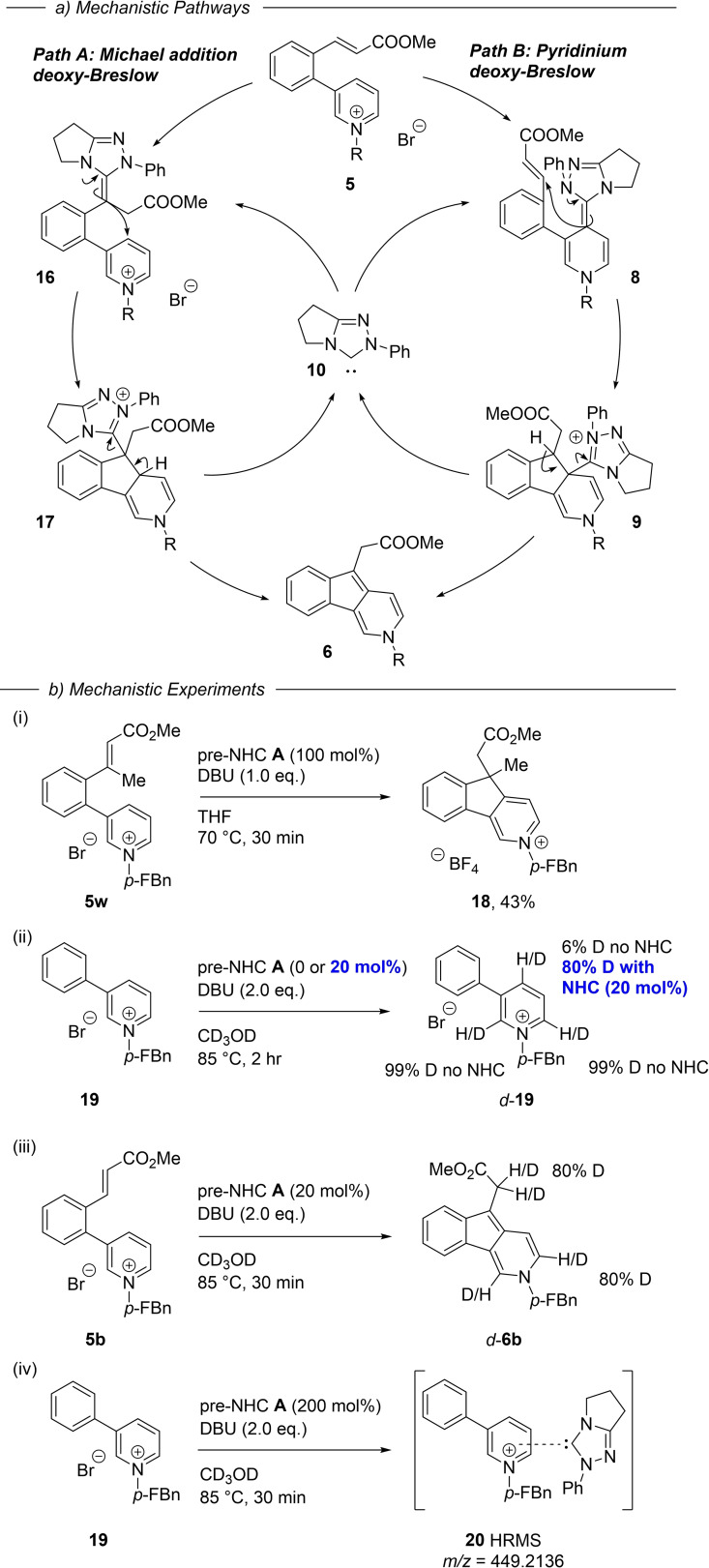
Mechanistic insights. *p‐*FBn = *p*‐fluorobenzyl

To gain further insights we examined deuterium incorporation into the simplified pyridinium **19** when run in CD_3_OD. Simple base‐mediated deuterium exchange at the pyridinium 2‐ and 6‐positions, but not the 4‐position, is substantial under these reaction conditions (Scheme 5(ii) and Supporting Information).^[14]^ In the presence of NHC catalyst (20 mol%), however, we see 80 % deuterium incorporation at the 4‐position after 2 hrs, indicating slower, reversible NHC addition / protonation via the arene deoxy‐Breslow intermediate.^[8]^

Running the umpolung reaction of **5** **b** in CD_3_OD shows the expected deuterium incorporation at pyridinium C2 and C6 along with the ester enolate position. Finally, we treated the simple 3‐phenylpyridinium salt **19** with stoichiometric pre‐NHC **A** and base to try and observe NHC‐pyridinium adduct formation (Scheme 5(iv)). A dark purple colour was observed on mixing, and analysis of the mixture identified the high‐resolution mass ion for an NHC‐substrate adduct along with small amounts of a new complex formed by ^19^F NMR (see Supporting Information). Attempts at isolating this species, however, were not successful.

In conclusion, we have shown for the first time that C−C bond formation is possible with areniums under NHC deoxy‐Breslow catalysis. The resulting umpolung reactivity enabled pyridinium alkylation at the C4 position with a variety of Michael acceptors, generating a novel series of indenopyridine structures which could be transformed to the analogous pyridinium ions via simple alkylation. Debenzylation to the parent pyridine structures was demonstrated to be feasible using a *para*‐siloxybenzyl moiety as a cleavable activating group. Further investigations into arenium NHC catalysis are underway in our laboratory.

## Conflict of interest

The authors declare no conflict of interest.

## Supporting information

As a service to our authors and readers, this journal provides supporting information supplied by the authors. Such materials are peer reviewed and may be re‐organized for online delivery, but are not copy‐edited or typeset. Technical support issues arising from supporting information (other than missing files) should be addressed to the authors.

Supporting Information

## Data Availability

The data that support the findings of this study are available from the corresponding author upon reasonable request.
