# Supplementary material for: NHC Catalysis for Umpolung Pyridinium Alkylation via Deoxy‐Breslow Intermediates
Source: Angew Chem Int Ed Engl. 2022 Feb 18;61(15):e202117524. doi: 10.1002/anie.202117524 (PMC9306516; doi:10.1002/anie.202117524)

Angewandte Chemie
Eine Zeitschrift der Gesellschaft Deutscher Chemiker

Supporting Information

NHC Catalysis for Umpolung Pyridinium Alkylation via Deoxy-Breslow Intermediates

*T. Wu, M. R. Tatton, M. F. Greaney**

Contents

General Considerations....2
Optimization....4
General procedure for optimization....4
Optimization of solvent....5
Optimization of catalyst loading....5
Optimization of base....6
Optimization of catalyst....6
Optimization of temperature....7
Control reactions....7
Base screen for enone Michael acceptor....8
Mechanistic Studies....9
Reaction of tri-substituted Michael acceptor 5v....9
Deuteration Studies....10
Investigation into effect of alkene isomer....11
Investigation into effect of catalyst loading and time....12
Investigation into pyridinium-NHC adduct by HRMS....13
Investigation into pyridinium-NHC adduct by ^{19}F NMR....14
Large scale synthesis of indenopyidine 6r....16
General Procedures....17
Representative scheme for the synthesis of pyridinium starting materials....17
General procedure A for the preparation of 2-(pyridinyl)benzaldehydes via Suzuki coupling 17
General procedure B for the Horner Wadsworth Emmons reaction of 2-(pyridinyl)benzaldehydes....18
General procedure C1 for the alkylation of pyridines....18
General procedure C2 for the alkylation of pyridines....18
General procedure D for the NHC catalysed formation of indenopyridines....19
Synthesis and Characterization of compounds....20
Preparation of 2-(pyridinyl)benzaldehydes....20
Preparation of Michael acceptors....26
Preparation of pyridiniums....35
Preparation of indenopyridines....51
Preparation of other compounds....64
References....73
^{1}H NMR, ^{13}C NMR and ^{19}F NMR Spectra of synthesised compounds....74

General Considerations

^{1}H NMR, ^{13}C NMR and ^{19}F NMR were recorded at 500/400 MHz, 126/101 MHz, and 471/376 MHz on a Bruker 500 or 400 spectrometers. All spectra are referenced to $CDCl_3$ residual chloroform peak (^{1}H NMR δ = 7.26 ppm; ^{13}C NMR δ = 77.16 ppm), to acetone-d_6 residual acetone peak (^{1}H NMR δ = 2.05 ppm; ^{13}C NMR δ = 29.84 ppm), methanol-d_4 residual methanol peak (^{1}H NMR δ = 3.31 ppm; ^{13}C NMR δ = 49.00 ppm) or DMSO-d_6 residual DMSO peak (^{1}H NMR δ = 2.50 ppm; ^{13}C NMR δ = 39.52 ppm). All chemical shifts are quoted in parts per million (ppm), measured from the centre of the signal except in the case of multiplets, which are quoted as a range. Coupling constants are quoted to the nearest 0.1 Hz. Splitting patterns are abbreviated as follows: singlet (s), doublet (d), triplet (t), quartet (q), multiplet (m), and combinations thereof. Low resolution mass spectrometry was performed on an Agilent 6100 mass spectrometer (ESI ionisation) and Hewlett Packard 5971 MSD (GC/MS with EI). High resolution mass spectrometry was performed on a Waters QTOF with ESI/APCI ionisation and a Thermo Finnigan MAT95XP (EI). Infra-red spectroscopy was recorded on a Bruker Alpha FTIR spectrometer (thin-film). Melting points were determined using a Kofler hot stage apparatus or Stuart Scientific SMP10 apparatus and are uncorrected. Thin layer chromatography (TLC) was performed using pre–coated Merck 60F254 silica plates. Visualization was performed using either UV light or treatment with acidified potassium permanganate solution. Flash chromatography was performed using Biotage Sfär cartridges on a Biotage Isolera automated machine. Microwave reactions were performed on a Biotage Initiator machine. Commercially available reagents and solvents were purchased and used without further purification. All reactions were done under an atmosphere of nitrogen using dry solvents unless stated otherwise.

NHC pre-catalyst **A**[1], **D**[2], **E**[3], **F**[3] and **G**[4] were synthesised according to literature procedures whilst **B** and **C** were commercially available.

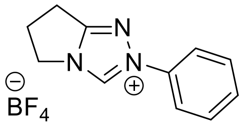

A

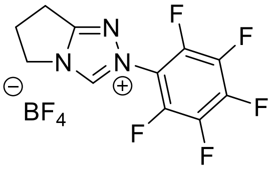

B

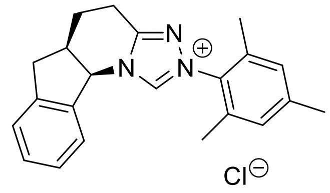

C

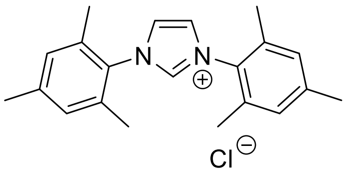

D

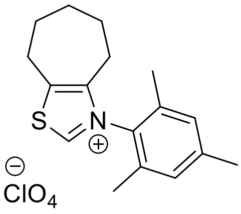

E

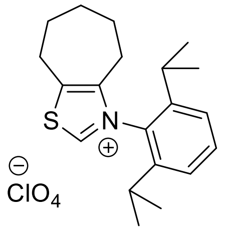

F

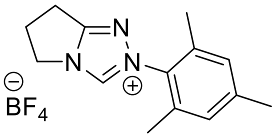

G

Optimization

General procedure for optimization

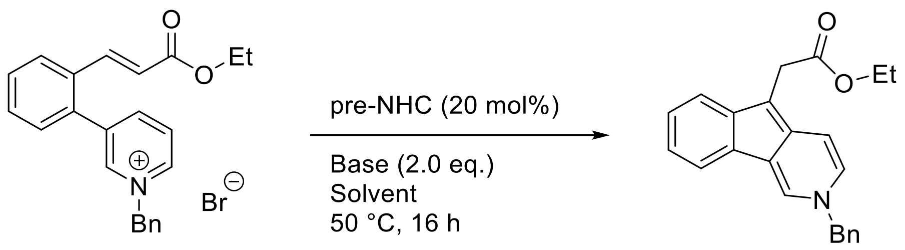

A flask containing appropriate pre-NHC was evacuated and backfilled 3 times with nitrogen. To this flask was added degassed solvent and appropriate base and the solution was stirred for 15 min.

A sealed microwave vial containing **5a** (42 mg, 0.10 mmol, 1.0 eq.) was evacuated and backfilled 3 times with nitrogen. To this vial was added the solution of catalyst and base. The reaction mixture was stirred at an appropriate temperature for an appropriate time before being quenched by addition of 1 M HCl (4 mL) and diethyl ether (20 mL) and extracted 3 times with 1 M HCl. The combined aqueous extracts were then basified with 2 M NaOH (20 mL) and extracted 3 times with diethyl ether. The combined organic extracts were washed, brine, dried over Na_2SO_4 and concentrated under reduced pressure. Yields of indenopyridine were determined by ^{1}H NMR analysis of the crude reaction mixtures using trimethoxybenzene as an internal standard.

Optimization of solvent

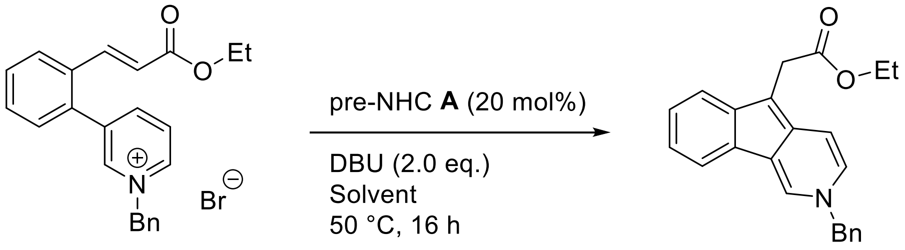

Entry	Change	% Yield
1	DMF	57%
2	THF	41%
3	MeOH	79%
4	DCM	37%
5	1,4-Dioxane	45%
6	Toluene	6%

Table S1: Reactions were carried out at 50 °C for 16 h with **5a** (42 mg, 0.10 mmol), pre-NHC **A** (10.9 mg, 0.04 mmol), DBU (30 µL, 0.20 mmol) and solvent (1 mL).

Optimization of catalyst loading

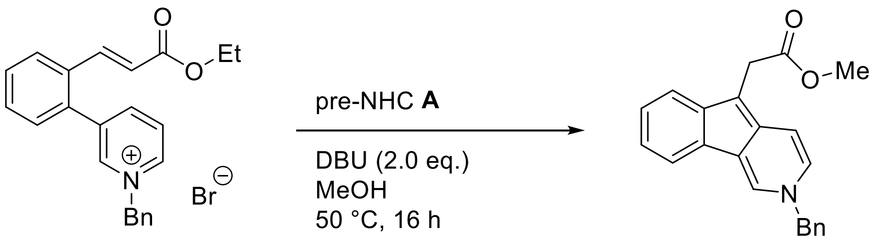

Entry	Catalyst Loading	% Yield
1	0 mol%	0%
2	5 mol%	41%
3	10 mol%	68%
4	20 mol%	83%
5	40 mol%	89%
6	100 mol%	92%

Table S2: Reactions were carried out at 50 °C for 16 h with **5a** (42 mg, 0.10 mmol) pre-NHC **A**, DBU (30 µL, 0.20 mmol) and MeOH (1 mL).

Optimization of base

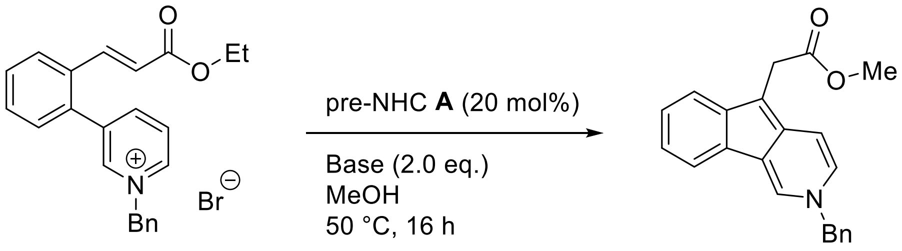

Entry	Base	% Yield
1	DIPEA	0%
2	TMG	61%
3	DABCO	0%
4	K_2CO_3	30%
5	NaOAc	31%
6	KO^{t}Bu	10%

Table S3: Reactions were carried out at 50 °C for 16 h with **5a** (42mg, 0.10 mmol) pre-NHC **A** (5.5 mg, 0.02 mmol), base (0.20 mmol) and MeOH (1 mL).

Optimization of catalyst

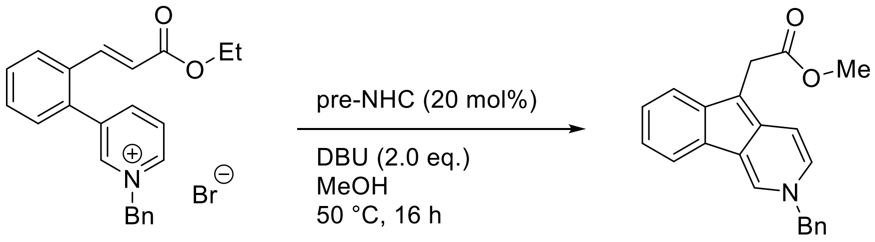

Entry	Catalyst Loading	% Yield
1	pre-NHC **A**	79%
2	pre-NHC **B**	27%
3	pre-NHC **C**	71%
4	pre-NHC **D**	0%
5	pre-NHC **E**	7%
6	pre-NHC **F**	21%

Table S4: Reactions were carried out at 50 °C for 16 h with **5a** (42 mg, 0.10 mmol) pre-NHC (0.02 mmol), DBU (30 µL, 0.20 mmol) and MeOH (1 mL).

Optimization of temperature

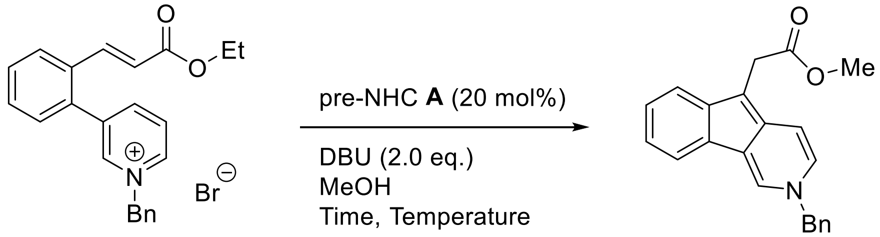

Entry	Time	Temperature	% Yield
1	16 h	0 °C	17%
2	16 h	50 °C	79%
3	16 h	85 °C	78%
4	4 h	50 °C	55%
5	4 h	85 °C	78%
6	0.5 h	85 °C	77%

Table S5: Reactions were carried out with **5a** (42mg, 0.10 mmol) pre-NHC **A** (5.5 mg, 0.02 mmol), DBU (30 µL, 0.20 mmol) and MeOH (4 mL).

Control reactions

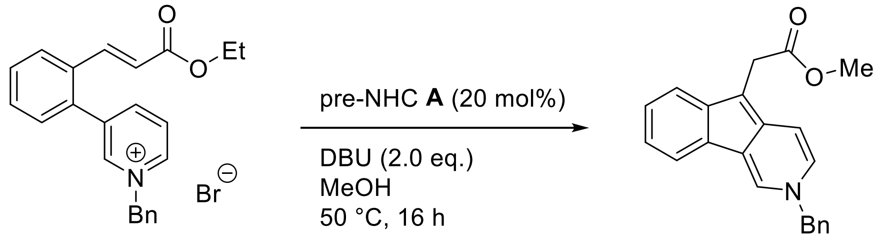

Entry	Variation	% Yield
1	Open to atmosphere	61%
2	1 eq. water	54%
3	8 eq. water	26%
4	No Base	0%
5	No Catalyst	0%
6	0.2 eq. DBU	8%

Table S6: Reactions were carried out at 50 °C for 16 h with **5a** (42 mg, 0.10 mmol) pre-NHC **A** (0.02 mmol), DBU (30 µL, 0.20 mmol) and MeOH (1 mL).

Base screen for enone Michael acceptor

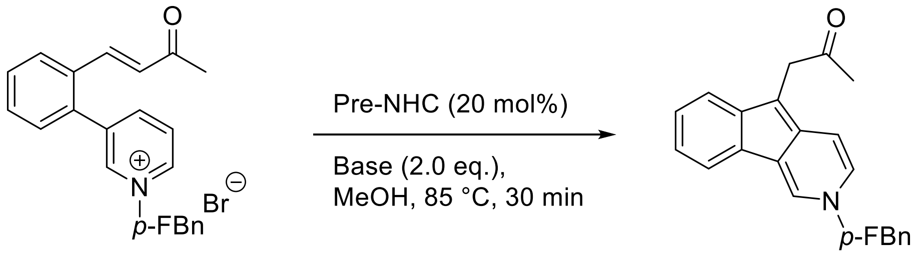

Entry	Base	pre-NHC	Solvent	Additive	% Yield
1	NaOAc	A	MeOH	-	3%
2	K_2CO_3	A	MeOH	-	35%
3	TMG	A	MeOH	-	12%
4	DBU	A	MeOH	-	19%
5	DBU	G	MeOH	-	30%
6	DBU	A	MeOH	AcOH (0.5 eq.)	9%
7	DBU	A	MeOH	AcOH (2.0 eq.)	0%
8	K_2CO_3	A	THF	-	6%
9	DBU	A	THF	-	9%

Mechanistic Studies

Reaction of tri-substituted Michael acceptor 5v

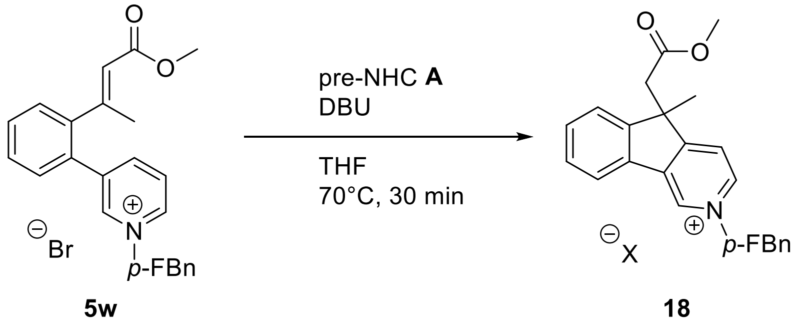

Entry	DBU	pre-NHC **A**	% Yield **18**
1	1.0 eq.	1.0 eq.	47%
2	1.0 eq.	0.2 eq.	27%
3[a]	1.0 eq.	0 eq.	0%
4[a]	0 eq.	1.0 eq.	0%

Table S7: a) reaction performed on 0.05 mmol scale instead.

A flask containing pre-NHC **A** was evacuated and backfilled 3 times with nitrogen. To this flask was added degassed THF (1 mL) and DBU and the solution was stirred for 15 min.

A sealed microwave vial containing **5w** (36 mg, 0.10 mmol, 1.0 eq.) was evacuated and backfilled 3 times with nitrogen. To this vial was added degassed THF (3 mL) followed by the solution of NHC and DBU in THF. The reaction mixture was stirred at 70 °C for 30 min before being cooled to room temperature and diluted with diethyl ether. The mixture was then filtered through a sinter glass filter and washed with diethyl ether. The residue was redissolved in methanol and concentrated. Yields of **18** were determined by ^{1}H NMR analysis of the crude reaction mixtures using trimethoxybenzene as an internal standard.

Deuteration Studies

Study 1

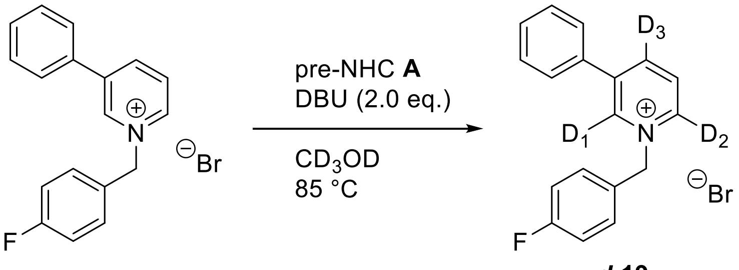

Entry	Catalyst Loading	Time	% D_1	% D_2	% D_3
1	0 mol%	30 min	72%	72%	0%
2	20 mol%	30 min	72%	71%	17%
3	50 mol%	30 min	98%	97%	93%
4	100 mol%	30 min	98%	98%	97%
5	0 mol%	2 h	99%	99%	6%
6	20 mol%	2 h	97%	97%	80%

Table S7: Deuteration studies.

A flask containing pre-NHC **A** was evacuated and backfilled 3 times with nitrogen. To this flask was added degassed deuterated methanol (2 mL) and DBU (15 µL, 0.10 mmol) and the solution was stirred for 15 min.
A sealed microwave vial containing **19** (17 mg, 0.05 mmol) was evacuated and backfilled 3 times with nitrogen. To this vial was added the solution of NHC and DBU in methanol. The reaction mixture was stirred at 85 °C before being quenched by addition of ND_4Cl (9 mg, 0.15 mmol) in D_2O (1 mL). The solvent was removed under reduced pressure and deuterium incorporation was determined by ^1H NMR analysis of the crude reaction mixtures.

Study 2

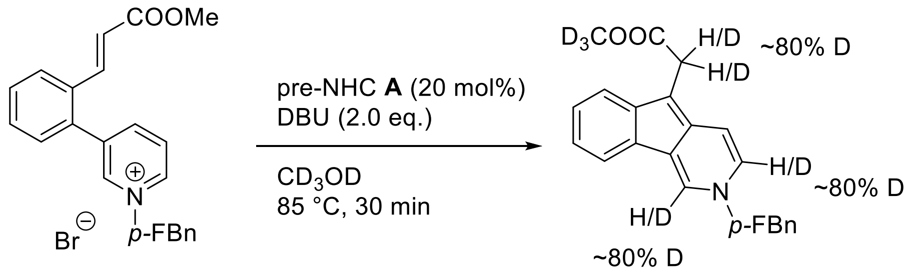

To a flask was added pre-NHC **A** (5.5 mg, 0.02 mmol, 0.2 eq.) and evacuated and backfilled 3 times with nitrogen. To this flask was added degassed deuterated methanol (4 mL) and DBU (30 µL, 0.20 mmol, 2.0 eq.) and the solution was stirred for 15 min.

To a microwave vial was added **5b** (43 mg, 0.10 mmol, 1.0 eq.) and sealed. The vial was evacuated and backfilled 3 times with nitrogen. To this vial was added the solution of NHC and DBU in methanol. The reaction mixture was stirred at 85 °C for 30 min before being quenched by addition of 1 M HCl (4 mL). The mixture was then basified with 2 M NaOH (10 mL) and extracted 3 times with diethyl ether. The combined organic extracts were washed with brine, dried over Na_2SO_4 and concentrated under reduced pressure. Deuterium incorporation was determined by ^{1}H NMR analysis of the crude reaction mixtures using trimethoxybenzene as an internal standard.

Investigation into effect of alkene isomer

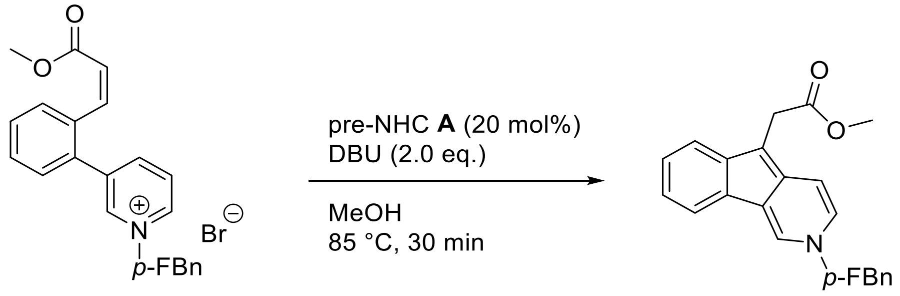

A flask containing pre-NHC **A** (5.5 mg, 0.02 mmol) was evacuated and backfilled 3 times with nitrogen. To this flask was added degassed deuterated methanol (4 mL) and DBU (30 µL, 0.20 mmol) and the solution was stirred for 15 min.
A sealed microwave vial containing **5b'** (42 mg, 0.10 mmol) was evacuated and backfilled 3 times with nitrogen. To this vial was added the solution of NHC and DBU in methanol. The reaction mixture was stirred at 85 °C for 30 min before being quenched by addition of 1 M HCl (4 mL). The mixture was then basified with 2 M NaOH (10 mL) and extracted 3 times with diethyl ether. The combined organic extracts were washed with brine, dried over Na_2SO_4 and concentrated under reduced pressure. Trimethoxybenzene was added as an internal standard and **6b** was obtained in a 47% yield by ^{1}H NMR analysis of the crude reaction.

Repeating the reaction using **5b** (the E isomer) gave **6b** in quantitative yield by ^{1}H NMR analysis of the crude reaction.

Investigation into effect of catalyst loading and time

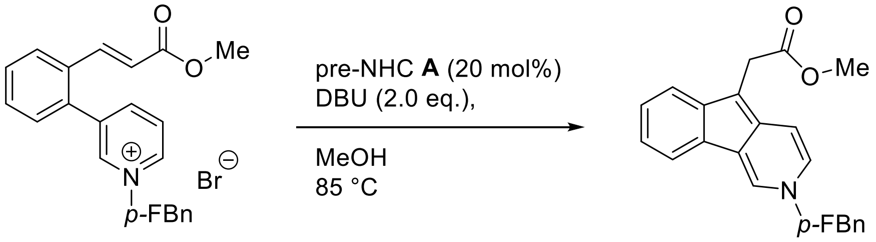

5b **6b**

Entry	Catalyst Loading	Time	% Yield
1	20 mol%	30 min	quant.
2	10 mol%	30 min	87%
3	5 mol%	30 min	66%
4	2 mol%	30 min	37%
5	10 mol%	2 h	86%
6	5 mol%	2 h	72%
7	2 mol%	2 h	43%

Table S2: Reactions were carried out at 85 °C with **5b** (43 mg, 0.10 mmol) pre-NHC **A**, DBU (30 µL, 0.20 mmol) and MeOH (4 mL). *p*-FBn = 4-Fluorobenzyl.

A flask containing pre-NHC **A** was evacuated and backfilled 3 times with nitrogen. To this flask was added degassed methanol (4 mL) and DBU (30 µL, 0.20 mmol) and the solution was stirred for 15 min.

A sealed microwave vial containing **5b** (43 mg, 0.10 mmol, 1.0 eq.) was evacuated and backfilled 3 times with nitrogen. To this vial was added the solution of NHC and DBU in methanol. The reaction mixture was stirred at 85 °C for an appropriate time before being quenched by addition of 1 M HCl (4 mL) and diethyl ether (20 mL) and extracted 3 times with 1 M HCl. The combined aqueous extracts were then basified with 2 M NaOH (20 mL) and extracted 3 times with diethyl ether. The combined organic extracts were washed, brine, dried over Na_2SO_4 and concentrated under reduced pressure. Yields of **6b** were determined by ^{1}H NMR analysis of the crude reaction mixtures using trimethoxybenzene as an internal standard.

Investigation into pyridinium-NHC adduct by HRMS

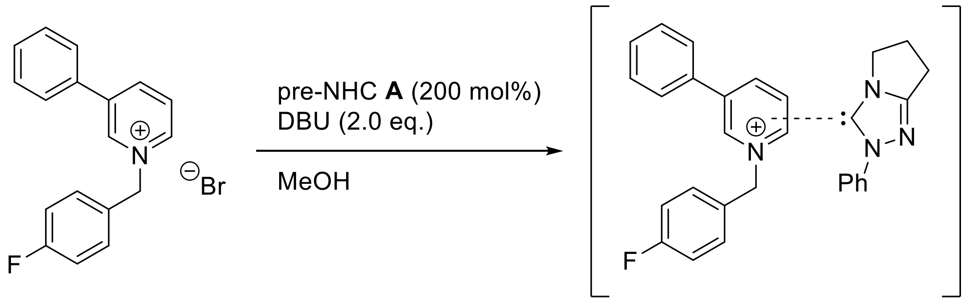

19 **20**

A flask containing pre-NHC **A** (5.5 mg, 0.02 mmol) was evacuated and backfilled 3 times with nitrogen. To this flask was added degassed methanol (2 mL) and DBU (3 µL, 0.02 mmol) and the solution was stirred for 15 min.

A sealed mass spectrometry vial containing **19** (3.4 mg, 0.01 mmol) was evacuated and backfilled 3 times with nitrogen. To this vial was added the solution of NHC and DBU in methanol. The reaction mixture was briefly stirred at 85 °C before being diluted in series to a concentration of around 0.01 mg/mL. The resulting mixture was analysed by mass spectrometry.
Masses corresponding to pyridinium **19**, NHC **A**, and DBU were observed. A mass corresponding to a pyridinium **19** and NHC **A** adduct (**20**) was also observed.

1,8-Diazabicyclo(5.4.0)undec-7-ene

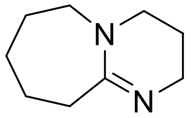

HRMS: Calculated for: $C_9H_{17}N_2$ $[M+H]^+$ 153.1386, found 153.1385.

Pyridinium **19**

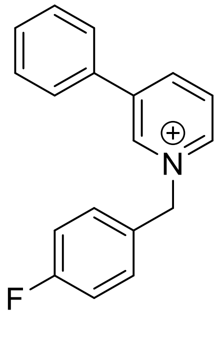

HRMS: Calculated for: $C_{18}H_{15}NF$ $[M]^+$ 264.1183, found 264.1179.

NHC **A**

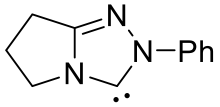

HRMS: Calculated for: $C_{11}H_{12}N_3$ $[M+H]^+$ 186.1026, found 186.1023.

Pyridinium 19 and NHC **A** adduct **(20)**

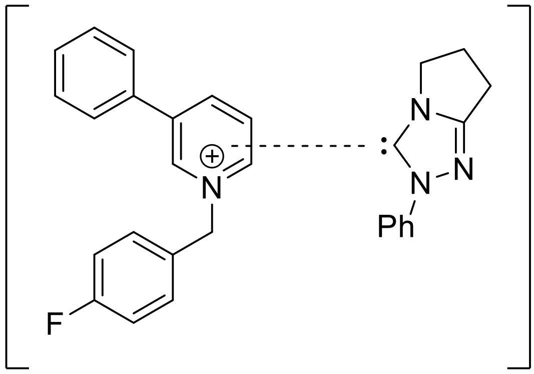

HRMS: Calculated for: $C_{29}H_{26}N_4F$ $[M]^+$ 449.2136, found 449.2133.

Investigation into pyridinium-NHC adduct by ^{19}F NMR

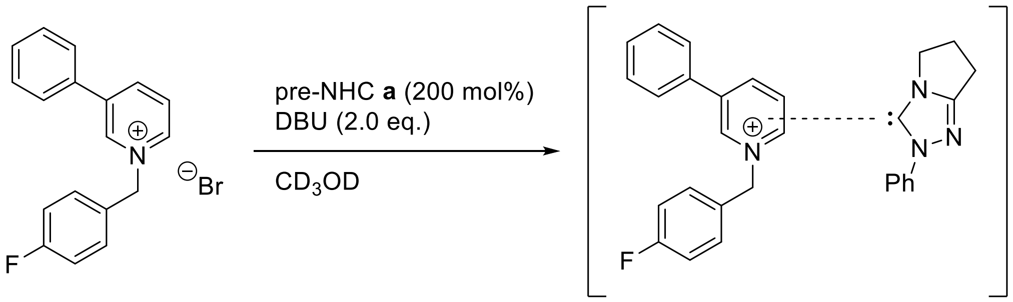

A flask containing pre-NHC **A** (5.5 mg, 0.02 mmol, 0.2 eq.) was evacuated and backfilled 3 times with nitrogen. To this flask was added degassed deuterated methanol (4 mL) and DBU (30 µL, 0.20 mmol, 2.0 eq.) and the solution was stirred for 15 min.
A sealed microwave vial containing **19** (0.10 mmol, 1.0 eq.) was evacuated and backfilled 3 times with nitrogen. To this vial was added the solution of NHC and DBU in methanol. The reaction mixture was briefly stirred at 85 °C before being transferred into a young's NMR tube and analysed by NMR spectroscopy (spectra **a**).

This reaction was repeated in the absence of pyridinium **19** (spectra **b**) and the absence pre-NHC **A** (spectra **c**).
A new peak at δ -116.82 not present in spectra **b** or spectra **c** was observed in the ^{19}F NMR of spectra **a**.

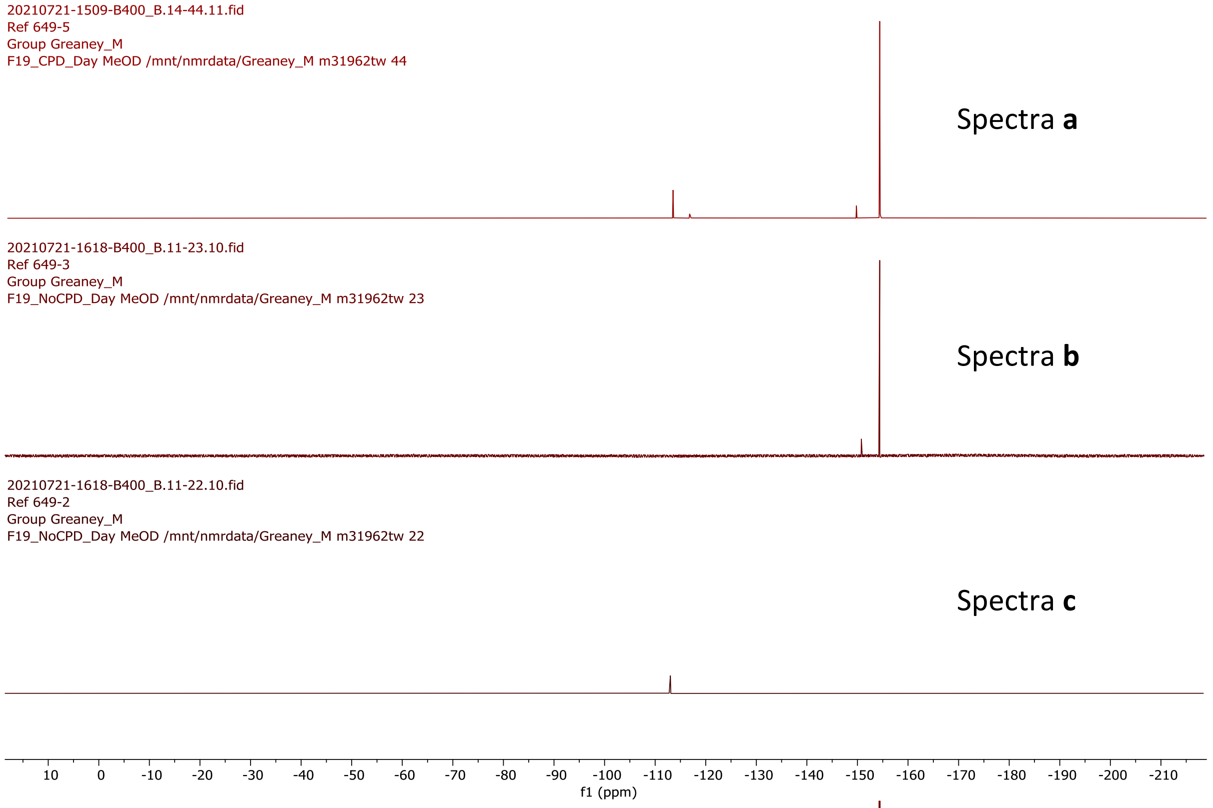
20210721-1509-B400_B.14-44.11.fid
Ref 649-5
Group Greaney_M
F19_CPD_Day MeOD /mnt/nmrdata/Greaney_M m31962tw 44
Spectra **a**
20210721-1618-B400_B.11-23.10.fid
Ref 649-3
Group Greaney_M
F19_NoCPD_Day MeOD /mnt/nmrdata/Greaney_M m31962tw 23
Spectra **b**
20210721-1618-B400_B.11-22.10.fid
Ref 649-2
Group Greaney_M
F19_NoCPD_Day MeOD /mnt/nmrdata/Greaney_M m31962tw 22
Spectra **c**
10 0 -10 -20 -30 -40 -50 -60 -70 -80 -90 -100 -110 -120 -130 -140 -150 -160 -170 -180 -190 -200 -210
f1 (ppm)

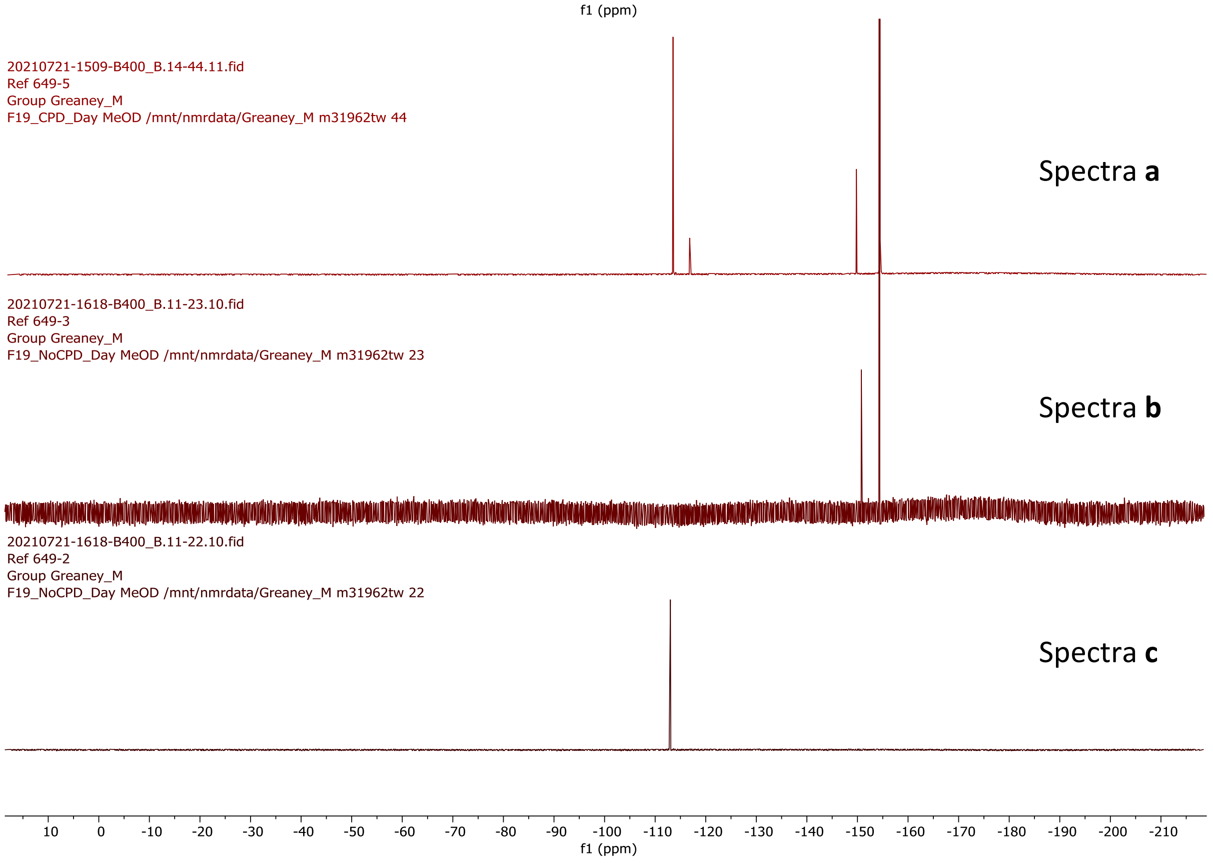
20210721-1509-B400_B.14-44.11.fid
Ref 649-5
Group Greaney_M
F19_CPD_Day MeOD /mnt/nmrdata/Greaney_M m31962tw 44
Spectra **a**
20210721-1618-B400_B.11-23.10.fid
Ref 649-3
Group Greaney_M
F19_NoCPD_Day MeOD /mnt/nmrdata/Greaney_M m31962tw 23
Spectra **b**
20210721-1618-B400_B.11-22.10.fid
Ref 649-2
Group Greaney_M
F19_NoCPD_Day MeOD /mnt/nmrdata/Greaney_M m31962tw 22
Spectra **c**
10 0 -10 -20 -30 -40 -50 -60 -70 -80 -90 -100 -110 -120 -130 -140 -150 -160 -170 -180 -190 -200 -210
f1 (ppm)

Large scale synthesis of indenopyidine 6r

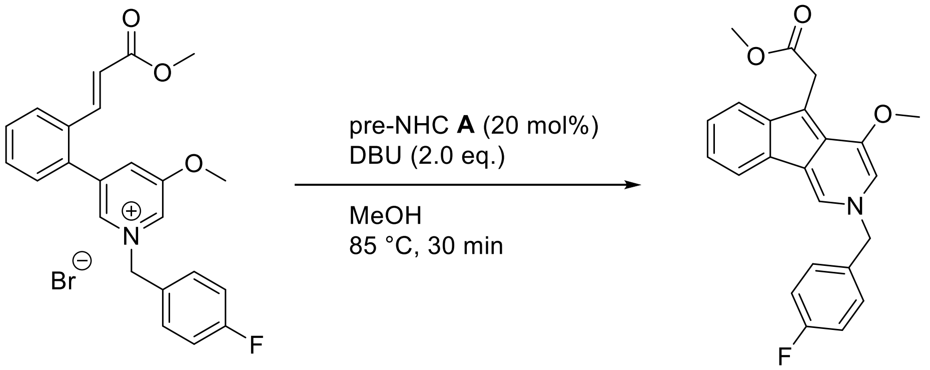

5s **6s**

A flask containing pre-NHC **A** (218 mg, 0.80 mmol) was evacuated and backfilled 3 times with nitrogen. To this flask was added degassed methanol (10 mL) and DBU (1.19 mL, 8.00 mmol) and the solution was stirred for 15 min.

A flask containing **5r** (1.83 g, 4.00 mmol) was evacuated and backfilled 3 times with nitrogen. To this flask was added degassed methanol (150 mL) followed by the solution of NHC and DBU in methanol. The reaction mixture was refluxed at 85 °C for 30 min before being quenched by addition of 1 M HCl (100 mL) and the solvent volume was reduced to about half on a rotary evaporator. To the mixture was added diethyl ether (100 mL) and extracted 3 times with 1 M HCl. The combined aqueous extracts were then basified with 2 M NaOH (20 mL) and extracted 3 times with diethyl ether. The combined organic extracts were washed with brine, dried over Na_2SO_4 and concentrated under reduced pressure. The resulting crude mixture was purified by recrystallization (hexanes:ethyl acetate) to yield **6r** as a yellow solid (1.07 g, 71%).

General Procedures

Representative scheme for the synthesis of pyridinium starting materials

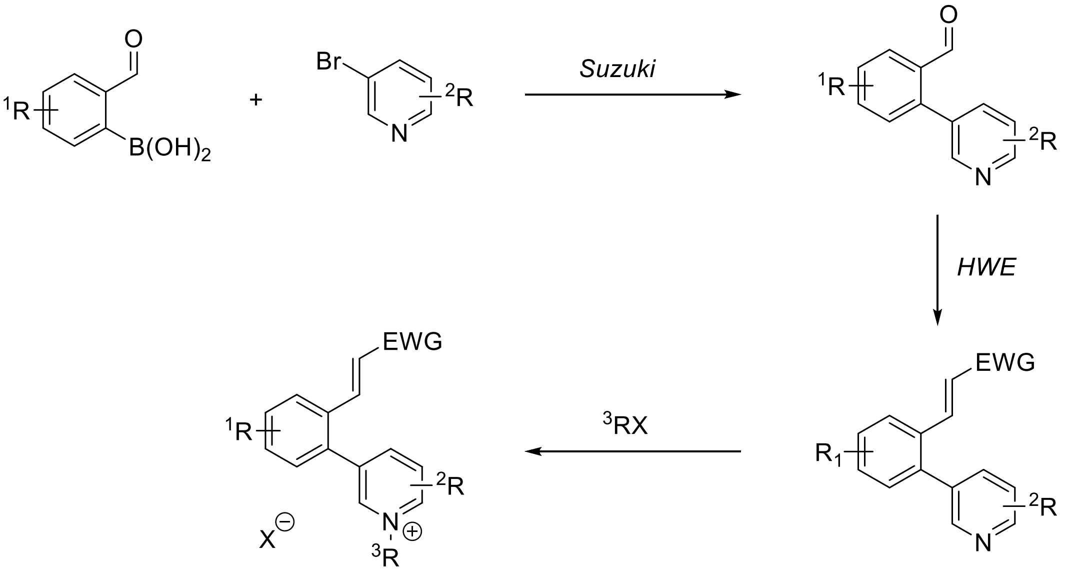

General procedure A for the preparation of 2-(pyridinyl)benzaldehydes via Suzuki coupling

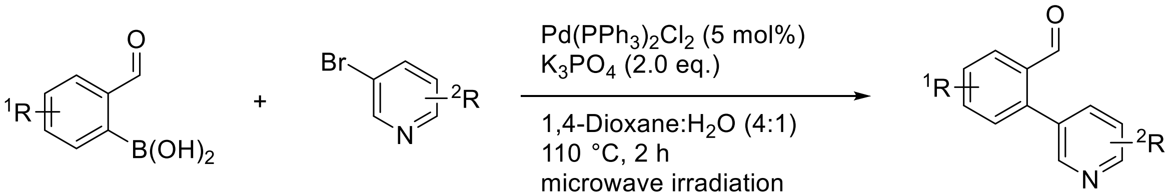

To a microwave vial was added appropriate aryl bromide (2.00 mmol, 1.0 eq.), appropriate aryl boronic acid (2.40 mmol, 1.2 eq.), $Pd(PPh_3)_2Cl_2$ (70 mg, 0.10 mmol, 0.05 eq.), K_3PO_4 (848 mg, 4.00 mmol, 2.0 eq.) and 1,4-dioxane:water (4:1, 4 mL). The vial was sealed and degassed with nitrogen for 1 h before being irradiated in microwave apparatus. After being cooled to room temperature, the reaction mixture was filtered through celite and the filtrate was extracted 3 times with ethyl acetate. The combined organic extracts were washed with brine, dried over Na_2SO_4 and concentrated under reduced pressure. The resulting crude mixture was purified by column chromatography on an automatic column machine (hexanes:ethyl acetate) to yield the product.

General procedure B for the Horner Wadsworth Emmons reaction of 2-(pyridinyl)benzaldehydes

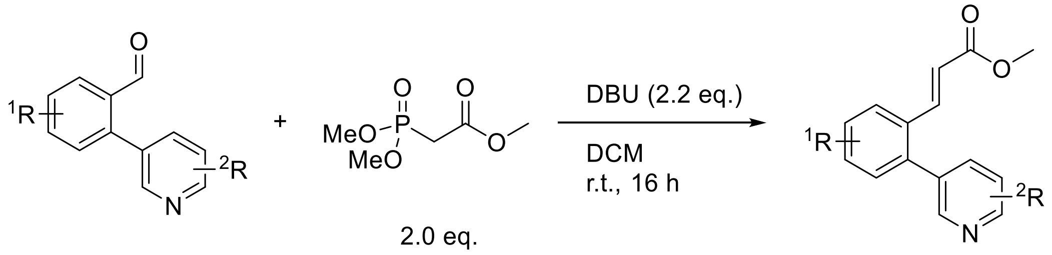

To a flask charged with methyl 2-(dimethoxyphosphoryl)acetate (162 µL, 1.00 mmol, 2.0 eq.) and DCM (1 mL) was added DBU (164 µL, 1.10 mmol, 2.2 eq.) and allowed to stir for 15 min. This solution was added to a solution of appropriate aldehyde (0.50 mmol, 1.0 eq.) in DCM (0.5 mL) and allowed to stir overnight at room temperature. The solvent was removed under reduced pressure and the resulting crude mixture was purified by column chromatography on an automatic column machine (hexanes:ethyl acetate) to yield the product.

General procedure C1 for the alkylation of pyridines

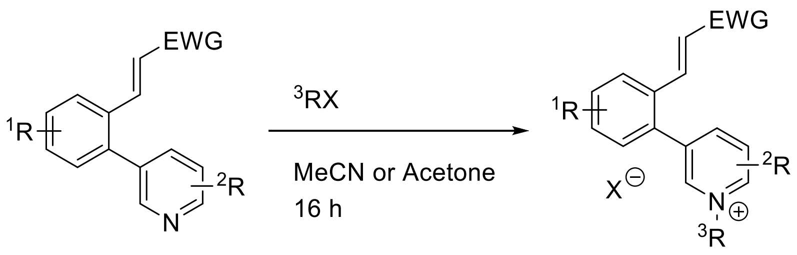

To a flask charged with appropriate pyridine (0.50 mmol, 1.0 eq.) and solvent was added appropriate alkylating agent (0.75 mmol, 1.5 eq.) and allowed to stir overnight at the appropriate temperature. The reaction mixture was diluted with diethyl ether, filtered and washed with more diethyl ether to yield the product.

General procedure C2 for the alkylation of pyridines

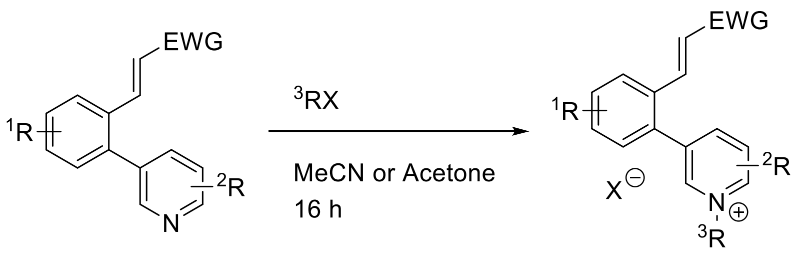

To a flask charged with appropriate pyridine (0.50 mmol, 1.0 eq.) and solvent was added appropriate alkylating agent (0.75 mmol, 1.5 eq.) and allowed to stir overnight at the appropriate temperature. The solvent was removed under reduced pressure and the resulting crude mixture was purified by column chromatography on an automated purification machine (hexanes:DCM:MeOH) to yield the product.

General procedure D for the NHC catalysed formation of indenopyridines

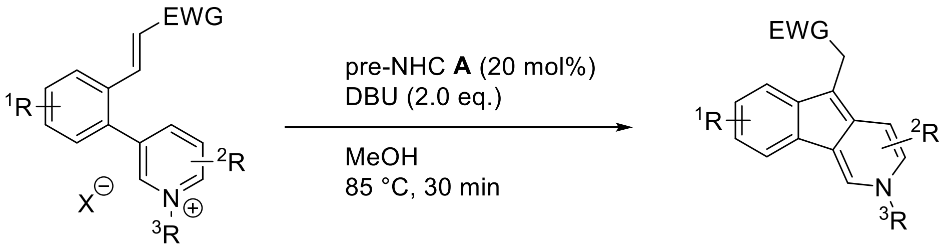

A flask containing pre-NHC **A** (5.5 mg, 0.02 mmol, 0.2 eq.) was evacuated and backfilled 3 times with nitrogen. To this flask was added degassed methanol (4 mL) and DBU (30 µL, 0.20 mmol, 2.0 eq.) and the solution was stirred for 15 min.

A sealed microwave vial containing appropriate pyridinium (0.10 mmol, 1.0 eq.) was evacuated and backfilled 3 times with nitrogen. To this vial was added the solution of NHC and DBU in methanol. The reaction mixture was stirred at 85 °C for 30 min before being quenched by addition of 1 M HCl (4 mL). The mixture was then basified with 2 M NaOH (10 mL) and extracted 3 times with diethyl ether. The combined organic extracts were washed with brine, dried over Na_2SO_4 and concentrated under reduced pressure. The resulting crude mixture was purified by column chromatography on an automatic column machine (hexanes:ethyl acetate:NEt_3) to yield the product.

Synthesis and Characterization of compounds

Preparation of 2-(pyridinyl)benzaldehydes

2-(Pyridin-3-yl)benzaldehyde (21a)

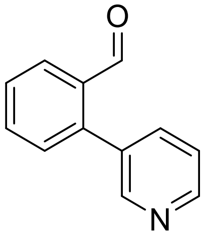

Prepared according to general procedure A using 3-bromopyridine (1.65 g, 10.0 mmol) and 2-formylphenylboronic acid (1.65 mg, 11.0 mmol) at 110 °C for 5 h to yield the titled compound as a white solid (1.53 g, 84%).
^{1}H NMR (400 MHz, Chloroform-*d*) δ 9.95 (s, 1H), 8.71 – 8.60 (m, 2H), 8.03 (dd, *J* = 7.8, 1.4 Hz, 1H), 7.74 – 7.62 (m, 2H), 7.58 – 7.49 (m, 1H), 7.39 (ddd, *J* = 7.8, 4.7, 1.0 Hz, 2H).
^{13}C NMR (101 MHz, Chloroform-*d*) δ 191.31, 150.13, 149.36, 141.81, 137.26, 133.95, 133.85, 133.74, 131.07, 128.74, 128.56, 123.21.
HRMS: Calculated for: $C_{12}H_{10}ON$ $[M+H]^+$ 184.0757, found 184.0754.
IR: 1680 1597 1194 826 756 712.
m.p. 60-61 °C.
Data is accordance to literature.[5]

4-Methyl-2-(pyridin-3-yl)benzaldehyde (21g)

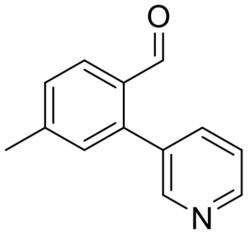

Prepared according to general procedure A using 2-bromo-4-methylbenzaldehyde (398 mg, 2.00 mmol) and pyridin-3-ylboronic acid (295 mg, 2.40 mmol) at 120 °C for 2 h to yield the titled compound as a yellow solid (150 mg, 38%).
^{1}H NMR (400 MHz, Chloroform-*d*) δ 9.91 (s, 1H), 8.68 (dd, *J* = 4.8, 1.6 Hz, 1H), 8.65 (d, *J* = 2.3 Hz, 1H), 7.96 (d, *J* = 7.9 Hz, 1H), 7.71 (dt, *J* = 7.9, 2.0 Hz, 1H), 7.41 (dd, *J* = 7.8, 4.9 Hz, 1H), 7.37 (dd, *J* = 7.8, 1.6 Hz, 1H), 7.21 (d, *J* = 1.7 Hz, 1H), 2.48 (s, 3H).
^{13}C NMR (101 MHz, Chloroform-*d*) δ 191.09, 150.02, 149.17, 145.15, 141.89, 137.45, 134.12, 131.76, 131.69, 129.70, 128.92, 123.24, 21.94.
HRMS: Calculated for: $C_{13}H_{12}ON$ $[M+H]^+$ 198.0913, found 198.0914.
IR: 1668 1602 1261 1180 1022 804 714.
m.p. 79-80 °C.

5-Methoxy-2-(pyridin-3-yl)benzaldehyde (21h)

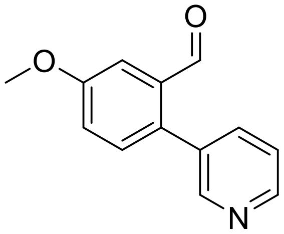

Prepared according to general procedure A using 2-bromo-5-methoxybenzaldehyde (430 mg, 2.00 mmol) and pyridin-3-ylboronic acid (295 mg, 2.40 mmol) at 120 °C for 2 h to yield the titled compound as a yellow solid (161 mg, 38%).

^{1}H NMR (400 MHz, Chloroform-*d*) δ 9.92 (s, 1H), 8.66 (dd, *J* = 4.9, 1.6 Hz, 1H), 8.63 (d, *J* = 2.2 Hz, 1H), 7.68 (dt, *J* = 7.8, 2.0 Hz, 1H), 7.54 (d, *J* = 2.8 Hz, 1H), 7.40 (ddd, *J* = 7.8, 4.9, 0.9 Hz, 1H), 7.34 (d, *J* = 8.5 Hz, 1H), 7.23 (dd, *J* = 8.5, 2.8 Hz, 1H), 3.91 (s, 3H).

^{13}C NMR (101 MHz, Chloroform-*d*) δ 191.27, 159.96, 150.31, 149.01, 137.55, 134.84, 134.80, 133.60, 132.41, 123.29, 121.63, 111.08, 55.80.

HRMS: Calculated for: $C_{13}H_{12}O_2N$ $[M]^+$ 214.0863, found 214.0861.

IR: 1675 1607 1471 1281 1234 1201 930 834 800 712.

m.p. 87-89 °C.

2-Fluoro-6-(pyridin-3-yl)benzaldehyde (21i)

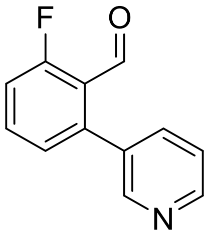

Prepared according to general procedure A using 3-bromopyridine (378 mg, 2.40 mmol) and (3-fluoro-2-formylphenyl)boronic acid (336 mg, 2.00 mmol) at 120 °C for 2 h to yield the titled compound as a pink solid (319 mg, 79%).

^{1}H NMR (400 MHz, Chloroform-*d*) δ 10.16 (s, 1H), 8.67 (dd, *J* = 4.9, 1.7 Hz, 1H), 8.59 (d, *J* = 2.3 Hz, 1H), 7.70 – 7.59 (m, 2H), 7.39 (ddd, *J* = 7.8, 4.9, 0.8 Hz, 1H), 7.35 – 7.21 (m, 1H), 7.18 (d, *J* = 7.6 Hz, 1H).

^{13}C NMR (101 MHz, Chloroform-*d*) δ 188.14 (d, *J* = 4.9 Hz), 163.75 (d, *J* = 261.7 Hz), 149.56, 149.39, 142.22 (d, *J* = 1.8 Hz), 136.95, 135.06 (d, *J* = 10.5 Hz), 134.00 (d, *J* = 2.5 Hz), 127.28 (d, *J* = 3.7 Hz), 123.07, 122.65 (d, *J* = 7.3 Hz), 116.73 (d, *J* = 21.7 Hz).

^{19}F NMR (376 MHz, Chloroform-*d*) δ -117.08.

HRMS: Calculated for: $C_{12}H_9ONF$ $[M+H]^+$ 202.0663, found 202.0663.

IR: 1687 1604 1407 1238 1186 1023 908 801 787 710.

m.p. 56-58 °C.

3-Fluoro-2-(pyridin-3-yl)benzaldehyde (21j)

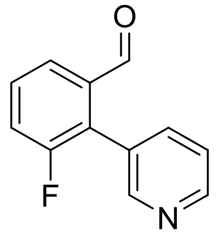

Prepared according to general procedure A using 2-bromo-3-fluorobenzaldehyde (406 mg, 2.00 mmol) and pyridin-3-ylboronic acid (295 mg, 2.40 mmol) at 120 °C for 2 h to yield the titled compound as a yellow oil (406 mg, 36%).

^{1}H NMR (400 MHz, Chloroform-*d*) δ 9.85 (d, *J* = 0.8 Hz, 1H), 8.78 – 8.70 (m, 1H), 8.64 (s, 1H), 7.87 (dd, *J* = 7.8, 1.2 Hz, 1H), 7.74 (ddt, *J* = 7.8, 2.4, 1.3 Hz, 1H), 7.61 – 7.51 (m, 1H), 7.51 –7.40 (m, 2H).

^{13}C NMR (101 MHz, Chloroform-*d*) δ 190.14 (d, *J* = 3.9 Hz), 159.97 (d, *J* = 249.0 Hz), 150.77, 149.75, 138.40, 135.86 (d, *J* = 2.0 Hz), 130.35 (d, *J* = 8.1 Hz), 128.84 (d, *J* = 16.5 Hz), 127.06, 124.50 (d, *J* = 3.4 Hz), 123.44, 121.35 (d, *J* = 22.7 Hz).

^{19}F NMR (376 MHz, Chloroform-*d*) δ -115.43.

HRMS: Calculated for: $C_{12}H_{9}ONF$ $[M+H]^{+}$ 202.0663, found 202.0661.

IR: 1688 1455 1409 1242 1001 785 752 735 713.

4-Methoxy-2-(pyridin-3-yl)benzaldehyde (21k)

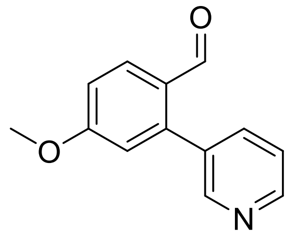

Prepared according to general procedure A using 2-bromo-4-methoxybenzaldehyde (430 mg, 2.00 mmol) and pyridin-3-ylboronic acid (295 mg, 2.40 mmol) at 120 °C for 2 h to yield the titled compound as a yellow solid (176 mg, 41%).

^{1}H NMR (400 MHz, Chloroform-*d*) δ 9.80 (s, 1H), 8.68 (dd, *J* = 4.9, 1.6 Hz, 1H), 8.65 (d, *J* = 2.3 Hz, 1H), 8.03 (d, *J* = 8.8 Hz, 1H), 7.71 (dt, *J* = 7.8, 2.0 Hz, 1H), 7.40 (ddd, *J* = 7.9, 4.9, 0.9 Hz, 1H), 7.08 – 7.01 (m, 1H), 6.84 (d, *J* = 2.5 Hz, 1H), 3.91 (s, 3H).

^{13}C NMR (101 MHz, Chloroform-*d*) δ 189.96, 163.83, 149.92, 149.39, 144.23, 137.31, 133.94, 131.21, 127.48, 123.21, 115.96, 114.55, 55.84.

HRMS: Calculated for: $C_{13}H_{12}O_{2}N$ $[M+H]^{+}$ 214.0863, found 214.0861.

IR: 1684 1672 1592 1573 1561 1300 1259 1224 1176 1127 1011 877 828 812 719.

m.p. 54-56 °C.

4,5-Dimethoxy-2-(pyridin-3-yl)benzaldehyde (21l)

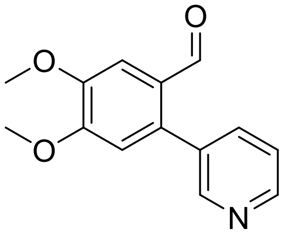

Prepared according to general procedure A using 2-bromo-4,5-dimethoxybenzaldehyde (490 mg, 2.00 mmol) and pyridin-3-ylboronic acid (295 mg, 2.40 mmol) at 120 °C for 2 h and used without further purification.

HRMS: Calculated for: $C_{14}H_{14}O_3N$ $[M+H]^+$ 244.0968, found 244.0966.

1-(Pyridin-3-yl)-2-naphthaldehyde (21m)

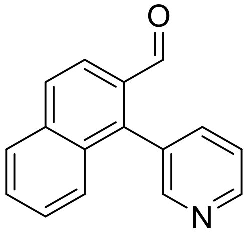

Prepared according to general procedure A using 1-bromo-2-naphthaldehyde (470 mg, 2.00 mmol) and pyridin-3-ylboronic acid (295 mg, 2.40 mmol) at 120 °C for 2 h to yield the titled compound as a brown oil (176 mg, 38%).

^{1}H NMR (400 MHz, Chloroform-*d*) δ 9.89 (d, *J* = 0.9 Hz, 1H), 8.81 (dd, *J* = 5.0, 1.7 Hz, 1H), 8.68 (d, *J* = 2.2 Hz, 1H), 8.10 (d, *J* = 8.6 Hz, 1H), 8.04 – 7.93 (m, 2H), 7.78 (dt, *J* = 7.7, 2.0 Hz, 1H), 7.66 (ddd, *J* = 8.2, 6.6, 1.4 Hz, 1H), 7.60 – 7.46 (m, 3H).

^{13}C NMR (101 MHz, Chloroform-*d*) δ 191.68, 150.96, 149.71, 142.11, 138.66, 136.22, 132.43, 131.85, 131.55, 129.43, 129.25, 128.61, 127.55, 127.16, 123.34, 122.58.

HRMS: Calculated for: $C_{16}H_{12}ON$ $[M+H]^+$ 234.0913, found 234.0909.

IR: 1685 1668 1242 819 790 767 747 722 713.

2-(5-Methoxypyridin-3-yl)benzaldehyde (21r)

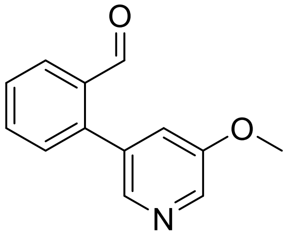

Prepared according to general procedure A using 3-bromo-5-methoxypyridine (376 mg, 2.00 mmol) and 2-formylphenylboronic acid (357 mg, 2.40 mmol) at 120 °C for 1 h to yield the titled compound as a white solid (347 mg, 81%).

^{1}H NMR (400 MHz, Chloroform-*d*) δ 9.98 (d, *J* = 0.8 Hz, 1H), 8.38 (d, *J* = 2.8 Hz, 1H), 8.24 (d, *J* = 1.8 Hz, 1H), 8.04 (dd, *J* = 7.9, 1.5 Hz, 1H), 7.67 (td, *J* = 7.5, 1.5 Hz, 1H), 7.55 (tt, *J* = 7.4, 1.1 Hz, 1H), 7.42 (dd, *J* = 7.6, 1.2 Hz, 1H), 7.20 (dd, *J* = 2.8, 1.8 Hz, 1H), 3.90 (s, 3H).

^{13}C NMR (101 MHz, Chloroform-*d*) δ 191.43, 155.40, 142.56, 141.74, 137.34, 134.28, 134.03, 133.97, 131.04, 128.82, 128.38, 121.79, 55.83.

HRMS: Calculated for: $C_{13}H_{12}O_2N$ $[M+H]^+$ 214.0863, found 214.0854.

IR: 1685 1585 1415 1405 1215 1196 1179 1014 875 826 762 712.

m.p. 102-104 °C.

2-(5-(Benzyloxy)pyridin-3-yl)benzaldehyde (21s)

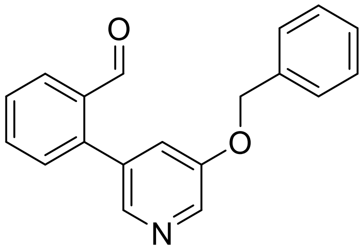

Prepared according to general procedure A using **23** (528 mg, 2.00 mmol) and 2-formylphenylboronic acid (357 mg, 2.40 mmol) at 120 °C for 1 h to yield the titled compound as a yellow solid (487 mg, 84%).

^{1}H NMR (400 MHz, Chloroform-*d*) δ 9.96 (d, *J* = 1.3 Hz, 1H), 8.48 (dd, *J* = 2.9, 1.3 Hz, 1H), 8.26 (t, *J* = 1.6 Hz, 1H), 8.05 (dt, *J* = 7.8, 1.5 Hz, 1H), 7.75 – 7.61 (m, 1H), 7.56 (t, *J* = 7.6 Hz, 1H), 7.50 – 7.32 (m, 6H), 7.29 (dt, *J* = 2.9, 1.5 Hz, 1H), 5.17 (d, *J* = 1.4 Hz, 2H).

^{13}C NMR (101 MHz, Chloroform-*d*) δ 191.25, 154.45, 142.69, 141.53, 137.95, 135.76, 134.20, 133.88 (2C), 130.95, 128.81 (2C), 128.74, 128.49, 128.29, 127.62 (2C), 122.77, 70.53.

HRMS: Calculated for: $C_{19}H_{16}O_2N$ [M+H]$^+$ 290.1176, found 290.1181.

IR: 1687 1582 1384 1312 1197 1166 1008 990 889 974 826 760 744 728 715 691 648.

m.p. 62-63°C.

2-(5-Fluoropyridin-3-yl)benzaldehyde (21t)

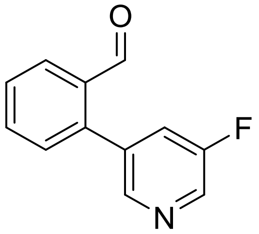

Prepared according to general procedure A using 3-bromo-5-fluoropyridine (352 mg, 2.00 mmol) and (2-formylphenyl)boronic acid (357 mg, 2.40 mmol) at 110 °C for 2 h to yield the titled compound as a white solid (334 mg, 85%).

^{1}H NMR (400 MHz, Chloroform-*d*) δ 9.97 (d, *J* = 1.3 Hz, 1H), 8.54 (t, *J* = 1.9 Hz, 1H), 8.44 (q, *J* = 1.5 Hz, 1H), 8.04 (dt, *J* = 7.8, 1.4 Hz, 1H), 7.69 (td, *J* = 7.5, 1.4 Hz, 1H), 7.63 – 7.54 (m, 1H), 7.45 (ddt, *J* = 8.8, 2.7, 1.4 Hz, 1H), 7.40 (dd, *J* = 7.6, 1.3 Hz, 1H).

^{13}C NMR (101 MHz, Chloroform-*d*) δ 190.91, 159.09 (d, *J* = 258.7 Hz), 146.12 (d, *J* = 4.0 Hz), 139.98, 137.82 (d, *J* = 23.2 Hz), 135.39 (d, *J* = 3.6 Hz), 134.10, 133.90, 131.13, 129.29, 129.18, 123.99 (d, *J* = 18.3 Hz).

^{19}F NMR (376 MHz, Chloroform-*d*) δ -126.53.

HRMS: Calculated for: $C_{12}H_9ONF$ [M+H]$^+$ 202.0663, found 202.0667.

IR: 1679 1594 1410 1185 896 823 758 708.

m.p. 106-108 °C.

2-(6-Methylpyridin-3-yl)benzaldehyde (21u)

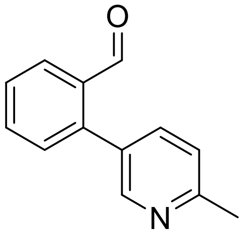

Prepared according to general procedure A using 5-bromo-2-methylpyridine (344 mg, 2.00 mmol) and 2-formylphenylboronic acid (357 mg, 2.40 mmol) at 120 °C for 1 h to yield the titled compound as a white solid (335 mg, 85%).

^{1}H NMR (400 MHz, Chloroform-*d*) δ 9.90 (d, *J* = 1.4 Hz, 1H), 8.47 – 8.42 (m, 1H), 7.95 (dt, *J* = 7.8, 1.6 Hz, 1H), 7.58 (tt, *J* = 7.6, 1.5 Hz, 1H), 7.52 (ddd, *J* = 7.8, 2.5, 1.3 Hz, 1H), 7.45 (t, *J* = 7.6 Hz, 1H), 7.37 – 7.30 (m, 1H), 7.20 (d, *J* = 7.9 Hz, 1H), 2.56 (d, *J* = 1.5 Hz, 3H).

^{13}C NMR (101 MHz, Chloroform-*d*) δ 191.32, 158.24, 149.33, 141.95, 137.44, 133.78, 133.74, 130.93, 130.53, 128.32, 128.24, 122.71, 24.17.

HRMS: Calculated for: $C_{13}H_{12}ON$ $[M+H]^+$ 198.0913, found 198.0916.

IR: 1694 1595 1473 1262 1201 1033 829 760 746 708.

m.p. 49-51 °C.

1-(2-(Pyridin-3-yl)phenyl)ethan-1-one (21w)

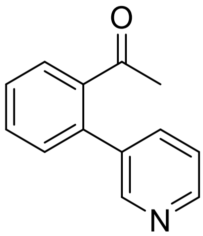

To a vial was added 1-(2-bromophenyl)ethan-1-one (995 mg, 5.00 mmol), pyridin-3-ylboronic acid (738 mg, 6.00 mmol), $Pd(PPh_3)_2Cl_2$ (175 mg, 0.30 mmol), K_3PO_4 (2.12 g, 10.00 mmol) and 1,4-dioxane:water (4:1, 10 mL). The vial was sealed and degassed with nitrogen for 1 h before being heated to 110 °C for 16 h. After being cooled to room temperature, the reaction mixture was filtered through celite and the filtrate was extracted 3 times with ethyl acetate. The combined organic extracts were washed with brine, dried over Na_2SO_4 and concentrated under reduced pressure. The resulting crude mixture was purified by column chromatography on an automatic column machine (hexanes:ethyl acetate) to yield the product as a colorless oil (299 mg, 30%).

^{1}H NMR (400 MHz, Chloroform-*d*) δ 8.64 (s, 1H), 8.59 (s, 1H), 7.65 (tt, *J* = 7.2, 1.6 Hz, 2H), 7.56 (td, *J* = 7.5, 1.5 Hz, 1H), 7.48 (td, *J* = 7.5, 1.4 Hz, 1H), 7.36 (dt, *J* = 8.0, 2.4 Hz, 2H), 2.20 (s, 3H).

^{13}C NMR (101 MHz, Chloroform-*d*) δ 202.96, 149.24, 148.89, 140.30, 137.13, 136.86, 136.33, 131.33, 130.94, 128.60, 128.40, 123.38, 30.41.

HRMS: Calculated for: $C_{13}H_{12}ON$ $[M+H]^+$ 198.0913, found 198.0907.

IR: 1687 1409 1356 1281 763 716.

Data is in accordance to literature.[6]

Preparation of Michael acceptors

Methyl (*E*)-3-(2-(pyridin-3-yl)phenyl)acrylate (22a)

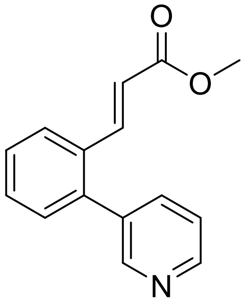

Prepared according to general procedure B using **21a** (375 mg, 2.05 mmol) to yield the titled compound as a light yellow solid (461 mg, 94%) and a mixture of isomers (*E:Z* = 17:1).
^{1}H NMR (500 MHz, Chloroform-*d*) δ 8.60 (dd, *J* = 4.9, 1.7 Hz, 1H), 8.55 (dd, *J* = 2.3, 0.9 Hz, 1H), 7.67 (dd, *J* = 7.7, 1.6 Hz, 1H), 7.62 – 7.57 (m, 2H), 7.45 – 7.36 (m, 2H), 7.36 – 7.28 (m, 2H), 6.38 (d, *J* = 15.9 Hz, 1H), 3.69 (s, 3H).
^{13}C NMR (126 MHz, Chloroform-*d*) δ 166.91, 150.08, 148.82, 142.74, 138.93, 137.03, 135.57, 132.89, 130.57, 130.12, 128.54, 127.13, 123.11, 119.85, 51.69.
HRMS: Calculated for: $C_{15}H_{13}O_2NNa$ [M+Na]$^+$ 262.0838, found 262.0829.
IR: 1709 1633 1319 1192 1172 768 754 717.
m.p. 35-36 °C.

Methyl (*Z*)-3-(2-(pyridin-3-yl)phenyl)acrylate (22a')

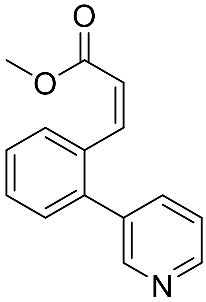

To a flask was added bis-(2,2,2-trifluoroethyl)(methoxycarbonylmethyl)phosphonate (159 mg, 0.50 mmol), 18-crown-6 (660 mg, 2.50 mmol), THF (4 mL) and was cooled to -78 °C. To this solution was added 0.4 M KHMDS solution in toluene (1.25 mL, 0.5 mmol) and the solution was allowed to stir for 15 min before a solution of **21a** in THF was added. The solution was allowed to stir at -78 °C for a further 2 h 30 min. Saturated ammonia chloride solution was added and the mixture was extracted 3 times with ethyl acetate. The combined organic extracts were washed with brine, dried over Na_2SO_4 and concentrated under reduced pressure. The resulting crude mixture was purified by column chromatography on an automatic column machine (hexanes:ethyl acetate) to yield the product as a colorless oil (77 mg, 64%) and a mixture of isomers (*E:Z* = 1:33).
^{1}H NMR (400 MHz, Chloroform-*d*) δ 8.67 – 8.52 (m, 2H), 7.70 (dt, *J* = 7.9, 2.1 Hz, 1H), 7.58 – 7.50 (m, 1H), 7.48 – 7.30 (m, 4H), 6.84 (d, *J* = 12.1 Hz, 1H), 5.97 (d, *J* = 12.2 Hz, 1H), 3.67 (s, 3H).
^{13}C NMR (101 MHz, Chloroform-*d*) δ 166.61, 150.29, 148.67, 143.54, 137.46, 137.37, 136.49, 134.41, 130.23, 129.68, 129.29, 127.88, 123.23, 121.41, 51.62.
HRMS: Calculated for: $C_{15}H_{14}O_2N$ [M+H]$^+$ 240.1019, found 240.1009.
IR: 1724 1467 1436 1408 1200 1163 1026 758 716.

Methyl (*E*)-3-(4-methyl-2-(pyridin-3-yl)phenyl)acrylate (22g)

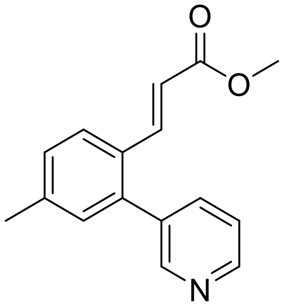

Prepared according to general procedure B using **21g** (98 mg, 0.50 mmol) to yield the titled compound as a beige solid (116 mg, 92%) and a mixture of isomers (*E:Z* = 20:1).

^{1}H NMR (400 MHz, Chloroform-*d*) δ 8.63 (dd, *J* = 4.9, 1.7 Hz, 1H), 8.59 – 8.55 (m, 1H), 7.65 – 7.54 (m, 3H), 7.36 (ddd, *J* = 7.8, 4.8, 0.9 Hz, 1H), 7.24 (dd, *J* = 8.1, 1.8 Hz, 1H), 7.17 – 7.12 (m, 1H), 6.36 (d, *J* = 15.9 Hz, 1H), 3.72 (s, 3H), 2.41 (s, 3H).

^{13}C NMR (101 MHz, Chloroform-*d*) δ 167.21, 150.16, 148.82, 142.74, 140.61, 139.07, 137.13, 135.81, 131.34, 130.20, 129.47, 127.13, 123.16, 118.89, 51.72, 21.43.

HRMS: Calculated for: $C_{16}H_{16}O_2N$ $[M+H]^+$ 254.1176, found 254.1163.

IR: 1716 1634 1607 1319 1276 1169.

m.p. 81-83 °C.

Methyl (*E*)-3-(5-methoxy-2-(pyridin-3-yl)phenyl)acrylate (22h)

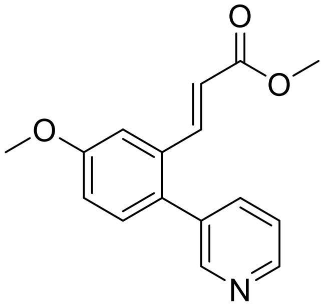

Prepared according to general procedure B using **21h** (107 mg, 0.50 mmol) to yield the titled compound as a beige solid (134 mg, 99%) and a mixture of isomers (*E:Z* = 17:1).

^{1}H NMR (400 MHz, Chloroform-*d*) δ 8.64 – 8.56 (m, 1H), 8.55 (d, *J* = 2.3 Hz, 1H), 7.63 – 7.54 (m, 2H), 7.38 – 7.30 (m, 1H), 7.26 (dd, *J* = 8.5, 1.5 Hz, 1H), 7.19 (d, *J* = 2.5 Hz, 1H), 7.05 – 6.97 (m, 1H), 6.39 (dd, *J* = 15.9, 1.2 Hz, 1H), 3.86 (q, *J* = 1.4 Hz, 3H), 3.73 (q, *J* = 1.2 Hz, 3H).

^{13}C NMR (101 MHz, Chloroform-*d*) δ 166.96, 159.68, 150.31, 148.54, 142.94, 137.20, 135.43, 134.07, 131.85, 131.77, 123.15, 120.09, 116.38, 111.83, 55.55, 51.81.

HRMS: Calculated for: $C_{16}H_{16}O_3N$ $[M+H]^+$ 270.1125, found 270.1121.

IR: 1716 1636 1604 1469 1319 1287 1224 1194 1172.

m.p. 62-64 °C.

Methyl (*E*)-3-(2-fluoro-6-(pyridin-3-yl)phenyl)acrylate (22i)

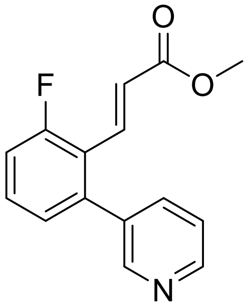

Prepared according to general procedure B using **21i** (101 mg, 0.50 mmol) to yield the titled compound as a beige solid (124 mg, 96%).

^{1}H NMR (400 MHz, Chloroform-*d*) δ 8.65 (dd, *J* = 4.9, 1.7 Hz, 1H), 8.57 (d, *J* = 2.3 Hz, 1H), 7.62 (dt, *J* = 7.9, 2.0 Hz, 1H), 7.44 – 7.34 (m, 3H), 7.22 – 7.09 (m, 2H), 6.54 (dd, *J* = 16.3, 1.5 Hz, 1H), 3.72 (d, *J* = 1.6 Hz, 3H).

^{13}C NMR (101 MHz, Chloroform-*d*) δ 167.23, 161.94 (d, *J* = 254.7 Hz), 150.05, 149.29, 141.33 (d, *J* = 3.1 Hz), 137.16, 136.46, 135.08 (d, *J* = 2.8 Hz), 130.71 (d, *J* = 10.0 Hz), 126.47 (d, *J* = 3.3 Hz), 124.49 (d, *J* = 13.7 Hz), 123.26, 121.31 (d, *J* = 11.7 Hz), 116.15 (d, *J* = 23.3 Hz), 51.85.

^{19}F NMR (376 MHz, Chloroform-*d*) δ -110.22.

HRMS: Calculated for: $C_{15}H_{13}O_2NF$ $[M+H]^+$ 258.0925, found 258.0923.

IR: 1718 1318 1271 1214 1200 1174 755 719.

m.p. 81-82 °C.

Methyl (*E*)-3-(3-fluoro-2-(pyridin-3-yl)phenyl)acrylate (22j)

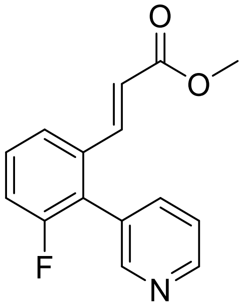

Prepared according to general procedure B using **21j** (101 mg, 0.50 mmol) to yield the titled compound as a colorless solid (124 mg, 97%) and a mixture of isomers (*E:Z* = 13:1).

^{1}H NMR (400 MHz, Chloroform-*d*) δ 8.69 (dd, *J* = 5.0, 1.6 Hz, 1H), 8.56 (d, *J* = 2.1 Hz, 1H), 7.70 – 7.63 (m, 1H), 7.56 – 7.49 (m, 1H), 7.49 – 7.32 (m, 3H), 7.22 (ddd, *J* = 9.3, 8.2, 1.2 Hz, 1H), 6.39 (d, *J* = 15.9 Hz, 1H), 3.73 (s, 3H).

^{13}C NMR (101 MHz, Chloroform-*d*) δ 166.72, 160.18 (d, *J* = 246.8 Hz), 150.65, 149.11, 141.41 (d, *J* = 3.5 Hz), 138.58, 135.78 (d, *J* = 2.8 Hz), 130.08 (d, *J* = 8.8 Hz), 129.15, 126.42, 123.54, 122.83 (d, *J* = 3.4 Hz), 121.37, 117.14 (d, *J* = 22.9 Hz), 51.98.

^{19}F NMR (376 MHz, Chloroform-*d*) δ -114.29.

HRMS: Calculated for: $C_{15}H_{13}O_2NF$ $[M+H]^+$ 258.0925, found 258.0920.

IR: 1719 1319 1276 1235 1194 1177 1165.

m.p. 60-62 °C.

Methyl (*E*)-3-(4-methoxy-2-(pyridin-3-yl)phenyl)acrylate (22k)

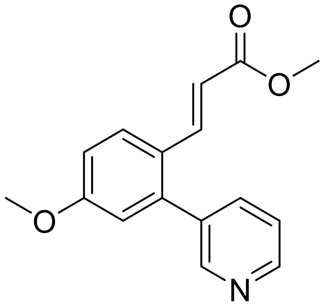

Prepared according to general procedure B using **21k** (107mg, 0.50 mmol) to yield the titled compound as a yellow solid (127 mg, 94%) and a mixture of isomers (*E:Z* = 20:1).
^{1}H NMR (400 MHz, Chloroform-*d*) δ 8.63 (dd, *J* = 4.9, 1.7 Hz, 1H), 8.58 (d, *J* = 2.3 Hz, 1H), 7.66 (d, *J* = 8.7 Hz, 1H), 7.61 (dt, *J* = 7.9, 2.0 Hz, 1H), 7.54 (d, *J* = 15.8 Hz, 1H), 7.36 (dd, *J* = 8.0, 4.8 Hz, 1H), 6.96 (dd, *J* = 8.7, 2.7 Hz, 1H), 6.82 (d, *J* = 2.6 Hz, 1H), 6.28 (dd, *J* = 16.0, 1.4 Hz, 1H), 3.84 (d, *J* = 1.6 Hz, 3H), 3.70 (d, *J* = 1.5 Hz, 3H).
^{13}C NMR (101 MHz, Chloroform-*d*) δ 167.34, 160.96, 150.04, 149.04, 142.31, 140.87, 137.05, 135.66, 128.72, 125.55, 123.17, 117.43, 115.54, 114.67, 55.57, 51.64.
HRMS: Calculated for: $C_{16}H_{16}O_3N$ [M+H]$^+$ 270.1125, found 270.1115.
IR: 1713 1602 1307 1274 1226 1191 1168.
m.p. 88-89 °C.

Methyl (*E*)-3-(4,5-dimethoxy-2-(pyridin-3-yl)phenyl)acrylate (22l)

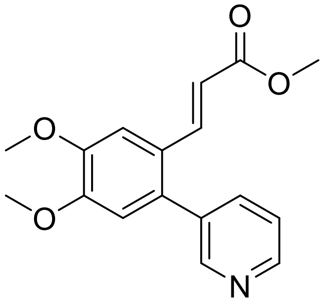

Prepared according to general procedure B using **21l** (122 mg, 0.50 mmol) and used without further purification.
HRMS: Calculated for: $C_{17}H_{18}O_4N$ [M+H]$^+$ 300.1230, found 300.1221.

Methyl (*E*)-3-(1-(pyridin-3-yl)naphthalen-2-yl)acrylate (22m)

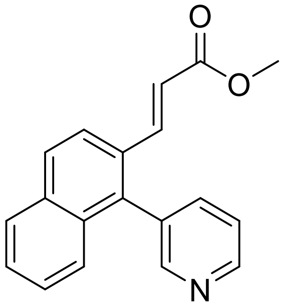

Prepared according to general procedure B using **21m** (117 mg, 0.50 mmol) to yield the titled compound as a beige solid (127 mg, 88%) and a mixture of isomers (*E:Z* = 13:1).
^{1}H NMR (400 MHz, Chloroform-*d*) δ 8.78 (dd, *J* = 4.9, 1.7 Hz, 1H), 8.59 – 8.56 (m, 1H), 7.96 – 7.88 (m, 2H), 7.82 (d, *J* = 8.8 Hz, 1H), 7.69 (dt, *J* = 7.8, 1.9 Hz, 1H), 7.58 – 7.37 (m, 5H), 6.49 (d, *J* = 15.9 Hz, 1H), 3.73 (s, 3H).

^{13}C NMR (101 MHz, Chloroform-*d*) δ 167.04, 151.08, 149.26, 142.59, 138.38, 137.24, 134.06, 133.48, 132.76, 130.70, 129.06, 128.19, 127.24, 127.12, 126.81, 123.38, 122.98, 119.71, 51.74.
HRMS: Calculated for: $C_{19}H_{15}O_2NNa$ [M+Na]$^+$ 312.0995, found 312.0984.
IR: 1717 1631 1434 1313 1299 1272 1260 1175 1158 818 716.
m.p. 90-92 °C.

Methyl (*E*)-3-(2-(6-methylpyridin-3-yl)phenyl)acrylate (22n)

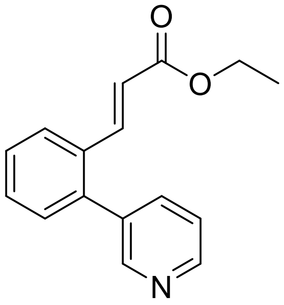

To a flask charged with **21a** (0.75 mmol, 1.0 eq.) in THF (1 mL) was added (carbethoxymethylene)triphenylphosphorane (348 mg, 1.00 mmol, 1.33 eq.) in THF (3 mL) and allowed to stir overnight at 50 °C. After being cooled to room temperature, the reaction was quenched by addition of water and the mixture was extracted with ethyl acetate 3 times. The combined organic extracts were washed with brine, dried over Na_2SO_4 and concentrated under reduced pressure. The resulting crude mixture was purified by column chromatography on an automated purification machine (hexanes:ethyl acetate) to yield the product as a yellow oil (190 mg, quant.) and a mixture of isomers (*E:Z* = 25:1).
^{1}H NMR (400 MHz, Chloroform-*d*) δ 8.64 (dd, *J* = 4.8, 1.7 Hz, 1H), 8.60 (d, *J* = 2.4 Hz, 1H), 7.74 – 7.68 (m, 1H), 7.68 – 7.55 (m, 2H), 7.45 (td, *J* = 7.3, 1.7 Hz, 2H), 7.41 – 7.32 (m, 2H), 6.41 (d, *J* = 15.9 Hz, 1H), 4.20 (q, *J* = 7.1 Hz, 2H), 1.28 (t, *J* = 7.1 Hz, 3H).
^{13}C NMR (101 MHz, Chloroform-*d*) δ 166.64, 150.20, 148.91, 142.63, 139.04, 137.24, 135.76, 133.15, 130.68, 130.18, 128.66, 127.25, 123.20, 120.45, 60.65, 14.38.
HRMS: Calculated for: $C_{16}H_{15}O_2NNa$ [M+Na]$^+$ 276.0995, found276.0987.
IR: 1708 1633 1314 1266 1175 768 756 717.
m.p. 60-62 °C.

Ethyl (*E*)-2-methyl-3-(2-(pyridin-3-yl)phenyl)acrylate (22o)

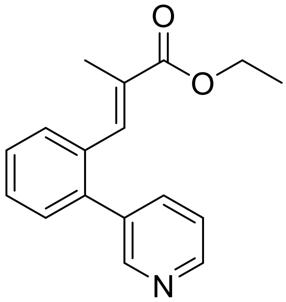

To a flask charged with ethyl 2-(diethoxyphosphoryl)propanoate (214 µL, 1.00 mmol, 1.33 eq.) and THF (3 mL) was added NaH (44 mg, 1.10 mmol, 1.47 eq.) at 0 °C and allowed to stir for 15 min. This solution was added to a solution of **21a** (0.75 mmol, 1.0 eq.) in THF (1 mL) and allowed to stir overnight at 50 °C. After being cooled to room temperature, the reaction was quenched by addition of water and the mixture was extracted with ethyl acetate 3 times. The combined organic extracts were washed with brine, dried over Na_2SO_4 and concentrated under reduced pressure. The resulting crude mixture was purified by column chromatography on an automated purification machine

(hexanes:ethyl acetate) to yield the product as a yellow solid (165 mg, 82%) and a mixture of isomers (*E:Z* = 10:1).
^{1}H NMR (400 MHz, Chloroform-*d*) δ 8.61 – 8.56 (m, 2H), 7.63 (dq, *J* = 7.8, 1.7 Hz, 1H), 7.49 (q, *J* = 1.6 Hz, 1H), 7.46 – 7.37 (m, 4H), 7.35 – 7.29 (m, 1H), 4.18 (q, *J* = 7.1 Hz, 2H), 1.97 (d, *J* = 1.5 Hz, 3H), 1.25 (td, *J* = 7.1, 0.8 Hz, 3H).
^{13}C NMR (101 MHz, Chloroform-*d*) δ 168.18, 150.09, 148.60, 138.18, 138.16, 136.96, 136.33, 134.58, 130.07, 130.04, 129.97, 128.74, 127.99, 123.07, 60.93, 14.34, 14.16.
HRMS: Calculated for: $C_{17}H_{17}O_2NNa$ $[M+Na]^+$ 290.1152, found 290.1149.
IR: 1705 1276 1247 1200 1120 755.
m.p. 60-62 °C.

(*E*)-*N*-Methoxy-*N*-methyl-3-(2-(pyridin-3-yl)phenyl)acrylamide (22p)

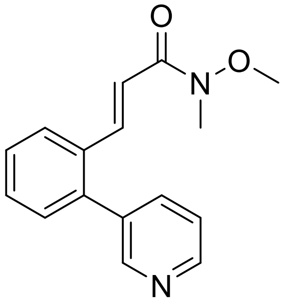

To a flask charged with diethyl (*N*-methoxy-*N*-methylcarbamoylmethyl)phosphonate (206 µL, 1.00 mmol, 1.33 eq.) and THF (3 mL) was added NaH (44 mg, 1.10 mmol, 1.47 eq.) at 0 °C and allowed to stir for 15 min. This solution was added to a solution of **21a** (0.75 mmol, 1.0 eq.) in THF (1 mL) and allowed to stir overnight at 50 °C. After being cooled to room temperature, the reaction was quenched by addition of water and the mixture was extracted with ethyl acetate 3 times. The combined organic extracts were washed with brine, dried over Na_2SO_4 and concentrated under reduced pressure. The resulting crude mixture was purified by column chromatography on an automated purification machine (DCM:MeOH) to yield the product as a colorless oil (185 mg, 92%).
^{1}H NMR (400 MHz, Chloroform-*d*) δ 8.65 – 8.58 (m, 2H), 7.79 – 7.72 (m, 1H), 7.71 – 7.61 (m, 2H), 7.48 – 7.41 (m, 2H), 7.40 – 7.30 (m, 2H), 6.95 (d, *J* = 15.7 Hz, 1H), 3.72 (s, 3H), 3.26 (s, 3H).
^{13}C NMR (101 MHz, Chloroform-*d*) δ 166.59, 150.09, 148.74, 141.55, 138.96, 137.28, 136.02, 133.94, 130.71, 129.78, 128.54, 127.52, 123.23, 118.30, 62.02, 32.54.
HRMS: Calculated for: $C_{16}H_{16}O_2N_2Na$ $[M+Na]^+$ 291.1104, found 291.1101.
IR: 1652 1615 1408 1378 997 761 717.

(*E*)-3-(2-(Pyridin-3-yl)phenyl)acrylonitrile (22q)

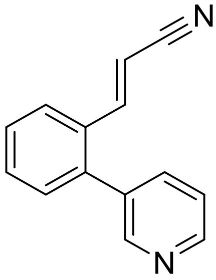

To a flask charged with diethyl cyanomethylphosphonate (124 µl, 0.77 mmol, 1.0 eq.) and THF (1 mL) was added NaH (31 mg, 0.77 mmol, 1.0 eq.) at 0 °C and allowed to stir for 15 min. This solution was added to a solution of **21a** (141 mg, 0.77 mmol, 1.0 eq.) in THF (1 mL) and allowed to stir for 1 h at 0 °C before being allowed to stir overnight at room temperature. The solvent was removed under reduced pressure and the resulting crude mixture was purified by column chromatography on an automatic column machine (hexanes:ethyl acetate) to yield the product as a colorless oil (63 mg, 39%).

^{1}H NMR (500 MHz, Chloroform-*d*) δ 8.69 (dd, *J* = 5.0, 1.7 Hz, 1H), 8.58 (d, *J* = 2.4 Hz, 1H), 7.64 (td, *J* = 6.8, 6.0, 1.7 Hz, 2H), 7.52 (dd, *J* = 7.5, 1.4 Hz, 1H), 7.50 – 7.42 (m, 2H), 7.37 (dd, *J* = 7.5, 1.5 Hz, 1H), 7.33 (d, *J* = 16.6 Hz, 1H), 5.88 (d, *J* = 16.5 Hz, 1H).

^{13}C NMR (126 MHz, Chloroform-*d*) δ 149.82, 149.03, 148.64, 138.73, 137.38, 135.27, 132.12, 131.22, 130.93, 128.98, 126.41, 123.51, 117.96, 98.29.

HRMS: Calculated for: $C_{14}H_{10}N_2Na$ [M+Na]$^+$ 229.0736, found 229.0733.

IR: 2217 1616 1467 1408 1001 969 813 758 716.

Data is in accordance to literature.[7]

Methyl (*E*)-3-(2-(5-methoxypyridin-3-yl)phenyl)acrylate (22r)

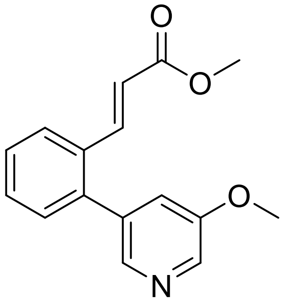

Prepared according to general procedure B using **21r** (107 mg, 0.50 mmol) to yield the titled compound as a white solid (135 mg, quant.) and a mixture of isomers (*E:Z* = 17:1).

^{1}H NMR (400 MHz, Chloroform-*d*) δ 8.35 (d, *J* = 2.8 Hz, 1H), 8.19 (d, *J* = 1.8 Hz, 1H), 7.71 (dd, *J* = 7.3, 1.9 Hz, 1H), 7.65 (d, *J* = 15.9 Hz, 1H), 7.46 (ddd, *J* = 8.2, 5.7, 1.8 Hz, 2H), 7.36 (dd, *J* = 7.2, 1.8 Hz, 1H), 7.14 (dd, *J* = 2.8, 1.8 Hz, 1H), 6.41 (d, *J* = 15.9 Hz, 1H), 3.89 (s, 3H), 3.75 (s, 3H).

^{13}C NMR (101 MHz, Chloroform-*d*) δ 167.06, 155.41, 142.93, 142.48, 138.82, 136.81, 136.28, 133.13, 130.67, 130.21, 128.74, 127.24, 121.82, 119.98, 55.83, 51.87.

HRMS: Calculated for: $C_{16}H_{16}O_3N$ [M+H]$^+$ 270.1125, found 270.1125.

IR: 1716 1316 1214 1197 1173 766.

m.p. 98-100 °C.

Methyl (*E*)-3-(2-(5-(benzyloxy)pyridin-3-yl)phenyl)acrylate (22s)

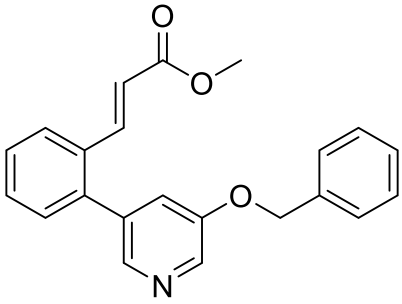

Prepared according to general procedure B using **21s** (145 mg, 0.50 mmol) to yield the titled compound as a colorless oil (172 mg, quant.) and a mixture of isomers (*E:Z* = 20:1).

^{1}H NMR (400 MHz, Chloroform-*d*) δ 8.42 (d, *J* = 2.8 Hz, 1H), 8.21 (d, *J* = 1.8 Hz, 1H), 7.71 (dd, *J* = 7.2, 1.9 Hz, 1H), 7.65 (d, *J* = 15.9 Hz, 1H), 7.48 – 7.32 (m, 8H), 7.22 (dd, *J* = 2.9, 1.7 Hz, 1H), 6.41 (d, *J* = 15.9 Hz, 1H), 5.14 (s, 2H), 3.75 (s, 3H).

^{13}C NMR (101 MHz, Chloroform-*d*) δ 167.03, 154.70, 142.90, 142.76, 138.74, 137.43, 136.34, 136.08, 133.13, 130.67, 130.20, 128.88 (2C), 128.74, 128.51, 127.74 (2C), 127.26, 122.89, 120.02, 70.74, 51.86.

HRMS: Calculated for: $C_{22}H_{20}O_3N$ $[M+H]^+$ 346.1438, found 346.1437.

IR: 1712 1419 1310 1194 1171 757.

Methyl (*E*)-3-(2-(5-fluoropyridin-3-yl)phenyl)acrylate (22t)

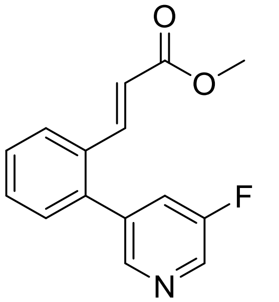

Prepared according to general procedure B using **21t** (101 mg, 0.50 mmol) to yield the titled compound as a white solid (128 mg, quant.) and a mixture of isomers (*E:Z* = 20:1).

^{1}H NMR (400 MHz, Chloroform-*d*) δ 8.52 (d, *J* = 2.7 Hz, 1H), 8.41 (d, *J* = 1.7 Hz, 1H), 7.76 – 7.69 (m, 1H), 7.59 (d, *J* = 15.9 Hz, 1H), 7.52 – 7.44 (m, 2H), 7.38 (ddd, *J* = 9.1, 2.8, 1.8 Hz, 1H), 7.36 – 7.32 (m, 1H), 6.42 (d, *J* = 15.9 Hz, 1H), 3.76 (s, 3H).

^{13}C NMR (101 MHz, Chloroform-*d*) δ 166.91, 159.20 (d, *J* = 258.3 Hz), 146.17 (d, *J* = 3.9 Hz), 142.33, 137.43, 137.35 (d, *J* = 23.1 Hz), 137.29, 133.18, 130.70, 130.35, 129.21, 127.45, 123.98 (d, *J* = 18.2 Hz), 120.61, 51.94.

^{19}F NMR (376 MHz, Chloroform-*d*) δ -126.60.

HRMS: Calculated for: $C_{15}H_{13}O_2NF$ $[M+H]^+$ 258.0925, found 258.0926.

IR: 1714 1634 1412 1317 1298 1269 1171 767.

m.p. 63-65 °C.

Methyl (*E*)-3-(2-(6-methylpyridin-3-yl)phenyl)acrylate (22u)

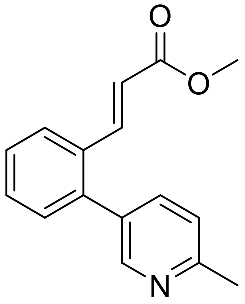

Prepared according to general procedure B using **21u** (99 mg, 0.50 mmol) to yield the titled compound as a colorless oil (115 mg, 91%) and a mixture of isomers (*E:Z* = 17:1).
^{1}H NMR (400 MHz, Chloroform-*d*) δ 8.47 (dd, *J* = 2.4, 0.8 Hz, 1H), 7.70 (dd, *J* = 7.7, 1.6 Hz, 1H), 7.65 (d, *J* = 15.9 Hz, 1H), 7.53 (dd, *J* = 7.9, 2.4 Hz, 1H), 7.49 – 7.39 (m, 2H), 7.34 (dd, *J* = 7.5, 1.6 Hz, 1H), 7.24 (d, *J* = 8.0 Hz, 1H), 6.41 (d, *J* = 15.9 Hz, 1H), 3.75 (s, 3H), 2.64 (s, 3H).
^{13}C NMR (101 MHz, Chloroform-*d*) δ 167.16, 157.76, 149.37, 143.16, 139.24, 137.63, 133.12, 132.78, 130.69, 130.13, 128.46, 127.24, 122.92, 119.79, 51.85, 24.30.
HRMS: Calculated for: $C_{16}H_{16}O_2N$ $[M+H]^+$ 254.1176, found 254.1171.
IR: 1713 1633 1317 1268 1194 1169 765 757.

Methyl 3-(2-(pyridin-3-yl)phenyl)but-2-enoate (22w)

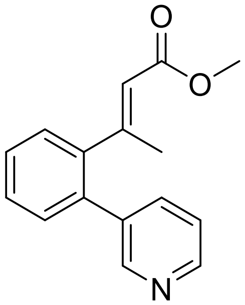

To a flask charged with methyl 2-(dimethoxyphosphoryl)acetate (450 µl, 2.78 mmol) and THF (10 mL) was added NaH (122 mg, 3.06 mmol) at 0 °C and allowed to stir for 15 min. To this solution was added a solution of **21w** (274 mg, 1.39 mmol) in THF (5 mL) and allowed to stir overnight at room temperature. Water was added to the reaction mixture and it was extracted 3 times with ethyl acetate. The combined organic extracts were washed with brine, dried over Na_2SO_4 and concentrated under reduced pressure. The crude product was used in the next step without further purification.
HRMS: Calculated for: $C_{16}H_{15}O_2NNa$ $[M+Na]^+$ 276.0995, found 276.0982.

Preparation of pyridiniums

(*E*)-1-Benzyl-3-(2-(3-ethoxy-3-oxoprop-1-en-1-yl)phenyl)pyridin-1-ium bromide (5a)

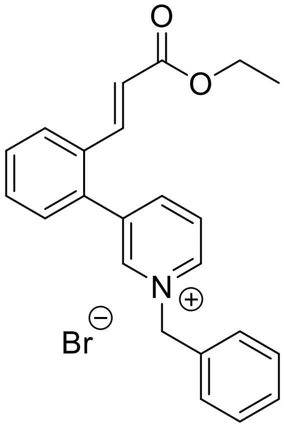

Prepared according to general procedure C2 using **22n** (1.52 g, 6.00 mmol) and benzyl bromide (1.43 mL, 12.00 mmol) in MeCN (30 mL) at 80 °C to yield the titled compound as a white solid (2.35 g, 92%) and a mixture of isomers (*E:Z* = 17:1).

^{1}H NMR (400 MHz, Methanol-d_4) δ 9.23 (d, *J* = 1.7 Hz, 1H), 9.17 (dt, *J* = 6.2, 1.4 Hz, 1H), 8.60 (dt, *J* = 8.1, 1.5 Hz, 1H), 8.23 (dd, *J* = 8.1, 6.1 Hz, 1H), 7.93 – 7.85 (m, 1H), 7.66 – 7.54 (m, 5H), 7.54 – 7.42 (m, 4H), 6.57 (d, *J* = 15.8 Hz, 1H), 5.98 (s, 2H), 4.21 (q, *J* = 7.1 Hz, 2H), 1.28 (t, *J* = 7.1 Hz, 3H).

^{13}C NMR (101 MHz, Methanol-d_4) δ 167.83, 147.73, 145.92, 144.69, 142.16, 142.05, 135.64, 134.47, 134.38, 132.00, 131.85, 131.71, 131.03, 130.76 (2C), 130.37 (2C), 129.44, 128.96, 123.14, 65.79, 61.88, 14.58.

HRMS: Calculated for: $C_{23}H_{22}O_2N$ $[M]^+$ 344.1645 found, 344.1637.

IR: 1703 1630 1313 1268 1177 1029 765 605 687.

m.p. 72-73 °C.

(*E*)-1-Benzyl-3-(2-(3-methoxy-3-oxoprop-1-en-1-yl)phenyl)pyridin-1-ium bromide (5a')

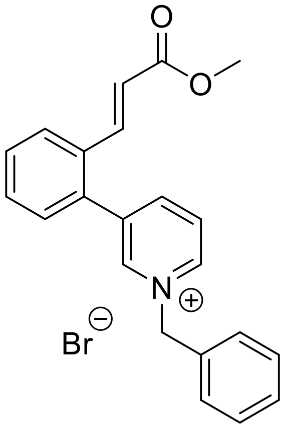

Prepared according to general procedure C2 using **22a** (350 mg, 1.46 mmol) and benzyl bromide (208 µL, 1.75 mmol) in MeCN (4.5 mL) at 80 °C to yield the titled compound as an off white solid (578 mg, 97%) and a mixture of isomers (*E:Z* = 17:1).

^{1}H NMR (500 MHz, Methanol-d_4) δ 9.20 (t, *J* = 1.6 Hz, 1H), 9.14 (dt, *J* = 6.3, 1.4 Hz, 1H), 8.61 (dt, *J* = 8.1, 1.5 Hz, 1H), 8.23 (dd, *J* = 8.1, 6.1 Hz, 1H), 7.93 – 7.88 (m, 1H), 7.65 – 7.53 (m, 5H), 7.53 – 7.44 (m, 4H), 6.57 (d, *J* = 15.7 Hz, 1H), 5.95 (s, 2H), 3.75 (s, 3H).

^{13}**C NMR** (126 MHz, Methanol-d_4) δ 168.31, 147.76, 145.97, 144.72, 142.35, 142.18, 135.70, 134.45, 134.42, 131.98, 131.90, 131.74, 131.08, 130.81 (2C), 130.32 (2C), 129.50, 128.99, 122.80, 65.89, 52.41.
HRMS: Calculated for: $C_{22}H_{20}O_2N$ $[M]^+$ 330.1489, found 330.1480.
IR: 1707 1629 1434 1320 1274 1198 1173 767 711 688.
m.p. 79-81 °C.

(*E*)-1-(4-Fluorobenzyl)-3-(2-(3-methoxy-3-oxoprop-1-en-1-yl)phenyl)pyridin-1-ium bromide (5b)

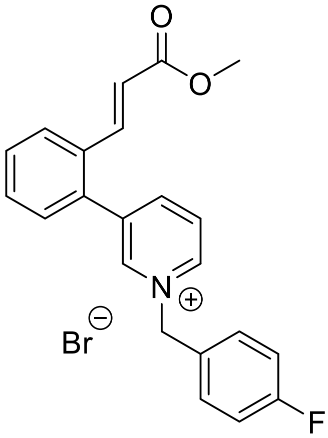

Prepared according to general procedure C1 using **22a** (239 mg, 1.00 mmol) and 4-fluorobenzyl bromide (187 µL, 1.50 mmol) in MeCN (3 mL) at 40 °C to yield the titled compound as a white solid (360 mg, 84%).
^{1}**H NMR** (400 MHz, Methanol-d_4) δ 9.21 (d, *J* = 1.7 Hz, 1H), 9.19 (dt, *J* = 6.1, 1.2 Hz, 1H), 8.62 (dt, *J* = 8.1, 1.5 Hz, 1H), 8.25 (dd, *J* = 8.1, 6.1 Hz, 1H), 7.93 – 7.86 (m, 1H), 7.75 – 7.66 (m, 2H), 7.66 – 7.55 (m, 3H), 7.48 (d, *J* = 15.8 Hz, 1H), 7.27 – 7.17 (m, 2H), 6.57 (d, *J* = 15.8 Hz, 1H), 5.97 (s, 2H), 3.75 (s, 3H).
^{13}**C NMR** (101 MHz, Methanol-d_4) δ 168.25, 164.93 (d, *J* = 248.4 Hz), 147.77, 145.90, 144.69, 142.34, 142.12, 135.67, 134.38, 132.88 (d, *J* = 8.8 Hz, 2C), 132.03, 131.90, 131.72, 130.59 (d, *J* = 3.4 Hz), 129.56, 128.98, 122.77, 117.58 (d, *J* = 22.1 Hz, 2C), 64.95, 52.39.
^{19}**F NMR** (376 MHz, Methanol-d_4) δ -112.86.
HRMS: Calculated for: $C_{22}H_{19}O_2NF$ $[M]^+$ 348.1394, found 348.1391.
IR: 1712 1508 1322 1172 1159 823 769 752 682.
m.p. 197-198 °C.

(*Z*)-1-(4-Fluorobenzyl)-3-(2-(3-methoxy-3-oxoprop-1-en-1-yl)phenyl)pyridin-1-ium bromide (5b')

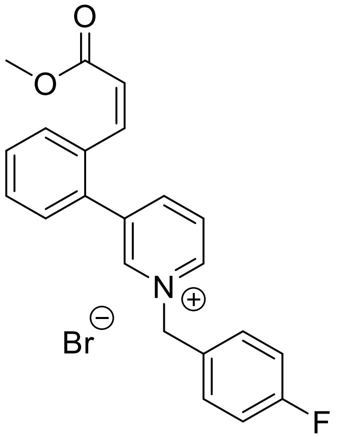

Prepared according to general procedure C2 using **22a'** (69 mg, 0.29 mmol) and 4-fluorobenzyl bromide (54 µL, 0.44 mmol) in MeCN (1 mL) at 80 °C to yield the titled compound as a white solid (85 mg, 68%) and a mixture of isomers (*E:Z* = 1:17).

^{1}H NMR (400 MHz, Methanol-d_4) δ 9.09 (dt, *J* = 6.1, 1.4 Hz, 1H), 9.01 (t, *J* = 1.9 Hz, 1H), 8.65 – 8.56 (m, 1H), 8.16 (dd, *J* = 8.1, 6.1 Hz, 1H), 7.65 – 7.59 (m, 2H), 7.58 – 7.49 (m, 3H), 7.42 – 7.38 (m, 1H), 7.27 – 7.19 (m, 2H), 7.03 (dd, *J* = 12.0, 0.9 Hz, 1H), 5.90 (s, 2H), 5.87 (d, *J* = 12.0 Hz, 1H), 3.49 (s, 3H).

^{13}C NMR (101 MHz, Methanol-d_4) δ 167.18, 165.02 (d, *J* = 248.6 Hz), 147.47, 145.59, 144.17, 143.20, 142.94, 136.39, 134.24, 132.98 (d, *J* = 8.8 Hz, 2C), 130.88, 130.78, 130.73, 130.54 (d, *J* = 3.3 Hz), 130.34, 129.18, 124.34, 117.58 (d, *J* = 22.1 Hz, 2C), 64.83, 51.91.

^{19}F NMR (376 MHz, Methanol-d_4) δ -112.82.

HRMS: Calculated for: $C_{22}H_{19}O_2NF[M]^+$ 348.1394, found 348.1380.

IR: 1715 1509 1224 1201 1159 823 758 685.

m.p. 58-60 °C.

(*E*)-3-(2-(3-Methoxy-3-oxoprop-1-en-1-yl)phenyl)-1-(4-methoxybenzyl)pyridin-1-ium chloride (5c)

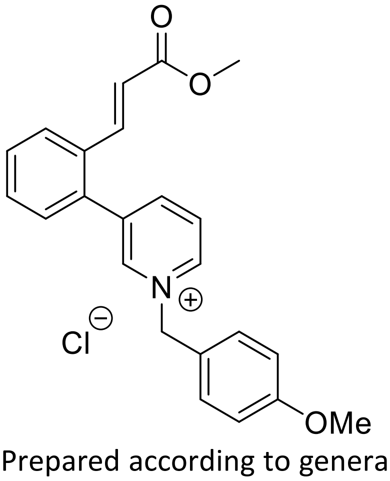

Prepared according to general procedure C2 using **22a** (115 mg, 0.48 mmol) and 4-methoxybenzyl chloride (97 µL, 0.72mmol) in MeCN (3 mL) at 80 °C to yield the titled compound as a yellow solid (190 mg, quant.) and a mixture of isomers (*E:Z* = 17:1).

^{1}H NMR (400 MHz, Methanol-d_4) δ 9.13 (dt, *J* = 6.2, 1.5 Hz, 2H), 8.59 (dt, *J* = 8.2, 1.5 Hz, 1H), 8.22 (dd, *J* = 7.8, 6.4 Hz, 1H), 7.93 – 7.84 (m, 1H), 7.66 – 7.59 (m, 2H), 7.59 – 7.51 (m, 3H), 7.46 (d, *J* = 15.9 Hz, 1H), 7.06 – 6.98 (m, 2H), 6.55 (dd, *J* = 15.8, 1.0 Hz, 1H), 5.87 (s, 2H), 3.82 (s, 3H), 3.75 (s, 3H).

^{13}C NMR (101 MHz, Methanol-d_4) δ 168.24, 162.41, 147.52, 145.62, 144.48, 142.34, 142.03, 135.74, 134.37, 132.14 (2C), 131.97, 131.91, 131.71, 129.42, 129.00, 126.14, 122.75, 116.07 (2C), 65.57, 55.90, 52.39.

HRMS: Calculated for: $C_{23}H_{22}O_3N$ $[M]^+$ 360.1594, found 360.1590.
IR: 1707 1631 1609 1512 1435 1320 1249 1175 1023 817 765.
m.p. 59-60°C.

(*E*)-3-(2-(3-Methoxy-3-oxoprop-1-en-1-yl)phenyl)-1-methylpyridin-1-ium iodide (5d)

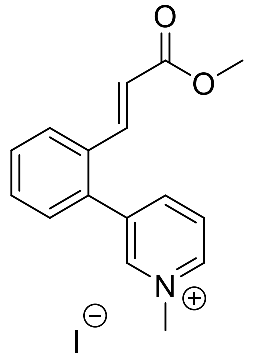

Prepared according to general procedure C1 using **22a** (120 mg, 0.50 mmol) and methyl iodide (62 µL, 1.00 mmol) in MeCN (2 mL) at 40 °C to yield the titled compound as a yellow solid (187 mg, 98%) and a mixture of isomers (*E:Z* = 17:1).
^{1}H NMR (500 MHz, Methanol-d_4) δ 9.06 (d, *J* = 2.0 Hz, 1H), 9.00 (d, *J* = 5.9 Hz, 1H), 8.54 (dt, *J* = 8.1, 1.5 Hz, 1H), 8.19 (dd, *J* = 8.1, 6.1 Hz, 1H), 7.98 – 7.91 (m, 1H), 7.65 – 7.60 (m, 2H), 7.60 – 7.52 (m, 2H), 6.63 (d, *J* = 15.8 Hz, 1H), 4.51 (s, 3H), 3.75 (s, 3H).
^{13}C NMR (126 MHz, Methanol-d_4) δ 168.54, 147.21, 146.86, 145.64, 142.39, 141.65, 135.88, 134.35, 131.88 (2C), 131.68, 128.83, 128.66, 122.54, 52.41, 49.18.
HRMS: Calculated for: $C_{16}H_{16}O_2N$ $[M]^+$ 254.1176, found 254.1165.
IR: 1711 1623 1320 1190 1175 1163 995 767 680.
m.p. 215-216 °C.

(*E*)-1-Ethyl-3-(2-(3-methoxy-3-oxoprop-1-en-1-yl)phenyl)pyridin-1-ium bromide (5e)

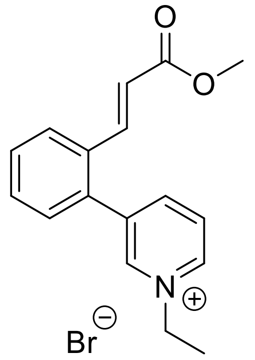

Prepared according to general procedure C2 using **22a** (239 mg, 1.00 mmol) and bromoethane (163 mg, 1.50 mmol) in MeCN (4 mL) at 100 °C to yield the titled compound as a yellow solid (315 mg, 91%) and a mixture of isomers (*E:Z* = 20:1).
^{1}H NMR (400 MHz, Methanol-d_4) δ 9.15 (d, *J* = 1.7 Hz, 1H), 9.12 (dd, *J* = 6.1, 1.3 Hz, 1H), 8.58 (dt, *J* = 8.2, 1.5 Hz, 1H), 8.23 (dd, *J* = 8.1, 6.0 Hz, 1H), 7.95 – 7.91 (m, 1H), 7.67 – 7.60 (m, 3H), 7.56 (d, *J* = 15.8 Hz, 1H), 6.62 (d, *J* = 15.8 Hz, 1H), 4.79 (q, *J* = 7.3 Hz, 2H), 3.75 (s, 3H), 1.73 (t, *J* = 7.3 Hz, 3H).
^{13}C NMR (101 MHz, Methanol-d_4) δ 168.44, 147.35, 145.78, 144.53, 142.47, 141.95, 135.85, 134.38, 131.92, 131.91, 131.69, 129.18, 128.86, 122.64, 58.82, 52.38, 16.99.
HRMS: Calculated for: $C_{17}H_{18}O_2N$ $[M]^+$ 268.1332, found 268.1319.
IR: 1703 1630 1434 1319 1271 1196 1162 767 687.
m.p. 58-60 °C.

(*E*)-3-(2-(3-Methoxy-3-oxoprop-1-en-1-yl)phenyl)-1-phenethylpyridin-1-ium bromide (5f)

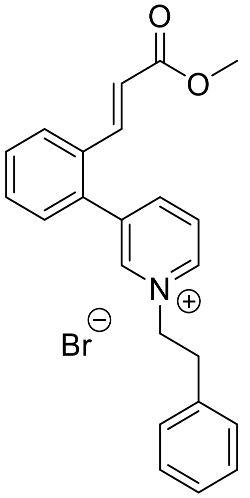

Prepared according to general procedure C2 using **22a** (239 mg, 1.00 mmol) and (2-bromoethyl)benzene (204 µL, 1.50 mmol) in MeCN (4 mL) at 100 °C to yield the titled compound as a beige solid (358 mg, 84%) and a mixture of isomers (*E:Z* = 20:1).

^{1}H NMR (400 MHz, Methanol-d_4) δ 8.93 – 8.82 (m, 2H), 8.55 – 8.47 (m, 1H), 8.16 – 8.05 (m, 1H), 7.94 – 7.86 (m, 1H), 7.66 – 7.54 (m, 2H), 7.49 (d, *J* = 15.8 Hz, 1H), 7.36 – 7.23 (m, 4H), 7.22 – 7.18 (m, 2H), 6.61 (d, *J* = 15.8 Hz, 1H), 5.00 (t, *J* = 7.0 Hz, 2H), 3.75 (s, 3H), 3.40 (t, *J* = 7.0 Hz, 2H).

^{13}C NMR (101 MHz, Methanol-d_4) δ 168.37, 147.60, 145.80, 144.77, 142.30, 141.73, 136.91, 135.64, 134.29, 131.78, 131.76, 131.69, 130.19 (2C), 130.10 (2C), 128.82, 128.80, 128.65, 122.65, 64.27, 52.39, 38.40.

HRMS: Calculated for: $C_{23}H_{22}O_2N$ $[M]^+$ 344.1645, found 344.1636.

IR: 1706 1631 1434 1320 1271 1197 1172 767 754 690.

m.p. 92-94 °C.

(*E*)-1-(4-Fluorobenzyl)-3-(2-(3-methoxy-3-oxoprop-1-en-1-yl)-5-methylphenyl)pyridin-1-ium bromide (5g)

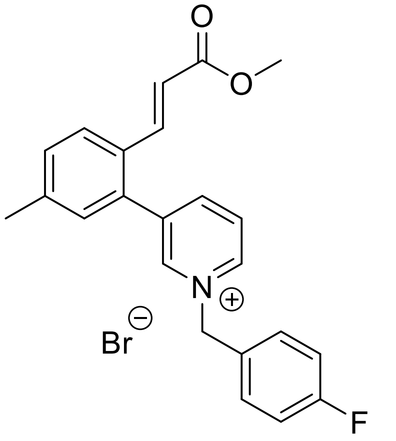

Prepared according to general procedure C2 using **22g** (109 mg, 0.43 mmol) and 4-fluorobenzyl bromide (87 µL, 0.70 mmol) in MeCN (3 mL) at 80 °C to yield the titled compound as a white solid (172 mg, 91%) and a mixture of isomers (*E:Z* = 20:1).

^{1}H NMR (400 MHz, Methanol-d_4) δ 9.25 – 9.11 (m, 2H), 8.60 (dt, *J* = 8.1, 1.5 Hz, 1H), 8.23 (dd, *J* = 8.0, 6.1 Hz, 1H), 7.79 (d, *J* = 8.0 Hz, 1H), 7.74 – 7.65 (m, 2H), 7.48 – 7.39 (m, 3H), 7.27 – 7.17 (m, 2H), 6.52 (dd, *J* = 15.8, 0.8 Hz, 1H), 5.95 (d, *J* = 2.0 Hz, 2H), 3.74 (s, 3H), 2.46 (s, 3H).

^{13}C NMR (101 MHz, Methanol-d_4) δ 168.48, 164.94 (d, *J* = 248.3 Hz), 147.75, 145.90, 144.61, 142.84, 142.26 (2C), 135.72, 132.86 (d, *J* = 8.8 Hz, 2C), 132.51, 132.45, 131.50, 130.61 (d, *J* = 3.3 Hz), 129.52, 128.89, 121.61, 117.59 (d, *J* = 22.1 Hz, 2C), 64.96, 52.33, 21.31.

^{19}F NMR (376 MHz, Methanol-d_4) δ -112.90.

HRMS: Calculated for: $C_{23}H_{21}O_2NF$ $[M]^+$ 362.1551, found 362.1551.

IR: 1705 1604 1509 1319 1224 1191 1168 818 685.

m.p. 173-174 °C.

(*E*)-1-(4-Fluorobenzyl)-3-(4-methoxy-2-(3-methoxy-3-oxoprop-1-en-1-yl)phenyl)pyridin-1-ium bromide (5h)

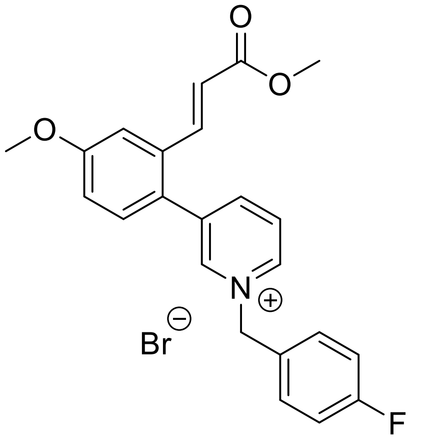

Prepared according to general procedure C2 using **22h** (126 mg, 0.47 mmol) and 4-fluorobenzyl bromide (87 µL, 0.70 mmol) in MeCN (3 mL) at 80 °C to yield the titled compound as a white solid (207 mg, 96%) and a mixture of isomers (*E:Z* = 17:1).

^{1}H NMR (400 MHz, Methanol-d_4) δ 9.15 – 9.06 (m, 2H), 8.57 (dt, *J* = 8.0, 1.5 Hz, 1H), 8.20 (dd, *J* = 8.1, 6.1 Hz, 1H), 7.67 (dddd, *J* = 9.0, 5.4, 3.4, 1.8 Hz, 2H), 7.51 (ddd, *J* = 8.7, 2.6, 1.4 Hz, 1H), 7.46 (d, *J* = 15.6 Hz, 1H), 7.38 (t, *J* = 2.4 Hz, 1H), 7.28 – 7.16 (m, 3H), 6.59 (dd, *J* = 15.8, 1.5 Hz, 1H), 5.91 (t, *J* = 2.3 Hz, 2H), 3.91 (d, *J* = 0.9 Hz, 3H), 3.76 (s, 3H).

^{13}C NMR (101 MHz, Methanol-*d*$_4$) δ 168.32, 164.97 (d, *J* = 248.4 Hz), 162.75, 147.68, 145.85, 144.12, 142.48, 142.18, 135.76, 133.48, 132.81 (d, *J* = 8.7 Hz, 2C), 130.61, 129.42, 128.06, 123.06, 117.88, 117.61 (d, *J* = 22.3 Hz, 2C), 113.76, 64.96, 56.26, 52.40.
^{19}F NMR (376 MHz, Methanol-*d*$_4$) δ -112.94.
HRMS: Calculated for: $C_{23}H_{21}O_3NF$ $[M]^+$ 378.1500, found 378.1500.
IR: 1705 1631 1510 1487 1289 1223 1194 1170 817.
m.p. 152-154 °C.

(*E*)-3-(3-Fluoro-2-(3-methoxy-3-oxoprop-1-en-1-yl)phenyl)-1-(4-fluorobenzyl)pyridin-1-ium bromide (5i)

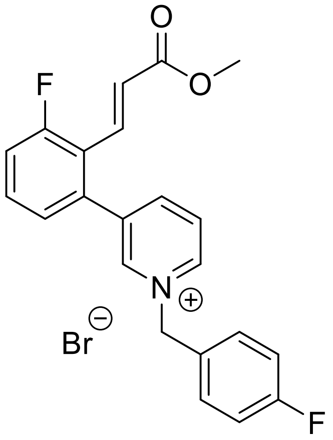

Prepared according to general procedure C1 using **22i** (117 mg, 0.46 mmol) and 4-fluorobenzyl bromide (87 µL, 0.70 mmol) in MeCN (3 mL) at 80 °C to yield the titled compound as a white solid (192 mg, 93%).
^{1}H NMR (400 MHz, Methanol-*d*$_4$) δ 9.24 (t, *J* = 1.6 Hz, 1H), 9.17 (dt, *J* = 6.2, 1.4 Hz, 1H), 8.65 (dt, *J* = 8.1, 1.5 Hz, 1H), 8.24 (dd, *J* = 8.1, 6.1 Hz, 1H), 7.70 – 7.58 (m, 3H), 7.48 – 7.39 (m, 2H), 7.32 (d, *J* = 16.3 Hz, 1H), 7.22 (t, *J* = 8.7 Hz, 2H), 6.46 (dd, *J* = 16.2, 1.4 Hz, 1H), 5.92 (s, 2H), 3.74 (s, 3H).
^{13}C NMR (101 MHz, Methanol-*d*$_4$) δ 168.12, 164.98 (d, *J* = 248.5 Hz), 162.85 (d, *J* = 253.2 Hz), 147.95, 146.17, 145.07, 141.34, 137.73, 136.25, 132.95 (d, *J* = 10.1 Hz), 132.79 (d, *J* = 8.8 Hz, 2C), 130.54, 129.67, 128.20 (d, *J* = 3.4 Hz), 127.08 (d, *J* = 12.0 Hz), 122.64 (d, *J* = 13.2 Hz), 119.00 (d, *J* = 23.4 Hz), 117.62 (d, *J* = 22.3 Hz, 2C), 65.08, 52.48.
^{19}F NMR (376 MHz, Methanol-*d*$_4$) δ -112.17, -112.89.
HRMS: Calculated for: $C_{22}H_{18}O_2NF_2$ $[M]^+$ 366.1300, found 366.1304.
IR: 1696 1316 1217 1202 1163 1144 825 806 771 757.
m.p. 194-195 °C.

(*E*)-3-(2-Fluoro-6-(3-methoxy-3-oxoprop-1-en-1-yl)phenyl)-1-(4-fluorobenzyl)pyridin-1-ium bromide (5j)

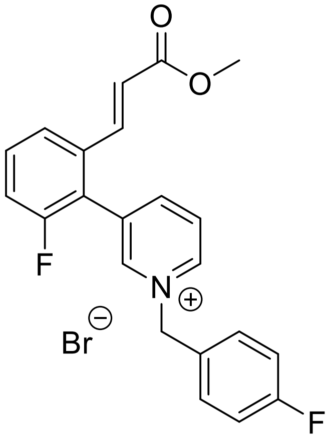

Prepared according to general procedure C2 using **22j** (117 mg, 0.46 mmol) and 4-fluorobenzyl bromide (87 µL, 0.70 mmol) in MeCN (3 mL) at 80 °C to yield the titled compound as a beige solid (123 mg, 61%) and a mixture of isomers (*E:Z* = 10:1).

^{1}H NMR (400 MHz, Methanol-d_4) δ 9.25 (d, *J* = 2.8 Hz, 2H), 8.70 – 8.63 (m, 1H), 8.33 – 8.25 (m, 1H), 7.77 (d, *J* = 7.9 Hz, 1H), 7.71 – 7.61 (m, 3H), 7.42 (t, *J* = 8.9 Hz, 1H), 7.32 (d, *J* = 15.8 Hz, 1H), 7.22 (t, *J* = 8.7 Hz, 2H), 6.59 (d, *J* = 15.8 Hz, 1H), 5.94 (t, *J* = 2.9 Hz, 2H), 3.75 (s, 3H).

^{13}C NMR (101 MHz, Methanol-d_4) δ 167.99, 164.98 (d, *J* = 248.6 Hz), 161.06 (d, *J* = 247.8 Hz), 149.15, 146.85, 145.61, 141.00 (d, *J* = 3.6 Hz), 136.95, 135.66, 133.51 (d, *J* = 9.2 Hz), 132.70 (d, *J* = 8.7 Hz, 2C), 130.60, 129.67, 124.81 (d, *J* = 3.4 Hz), 124.09, 123.08 (d, *J* = 15.3 Hz), 118.35 (d, *J* = 22.4 Hz), 117.63 (d, *J* = 22.2 Hz, 2C), 65.14, 52.46.

^{19}F NMR (376 MHz, Methanol-d_4) δ -112.88, -116.82.

HRMS: Calculated for: $C_{22}H_{18}O_2NF_2$ [M]$^+$ 366.1300, found 366.1300.

IR: 1708 1509 1224 1195 1162 801.

m.p. 76-78 °C.

(*E*)-3-(2-Fluoro-6-(3-methoxy-3-oxoprop-1-en-1-yl)phenyl)-1-(4-fluorobenzyl)pyridin-1-ium bromide (5k)

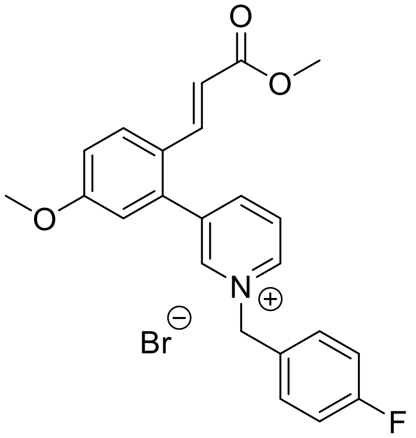

Prepared according to general procedure C1 using **22k** (119 mg, 0.44 mmol) and 4-fluorobenzyl bromide (87 µL, 0.70 mmol) in MeCN (3 mL) at 80 °C to yield the titled compound as a pale yellow solid (163 mg, 82%) and a mixture of isomers (*E:Z* = 20:1).

^{1}H NMR (400 MHz, Methanol-d_4) δ 9.21 (dd, *J* = 4.0, 2.0 Hz, 1H), 9.19 – 9.11 (m, 1H), 8.61 (dt, *J* = 8.1, 1.5 Hz, 1H), 8.23 (dd, *J* = 8.0, 6.1 Hz, 1H), 7.87 (dd, *J* = 8.8, 2.4 Hz, 1H), 7.73 – 7.64 (m, 2H), 7.39 (d, *J*

= 16.2 Hz, 1H), 7.28 – 7.10 (m, 4H), 6.46 (dd, *J* = 15.7, 2.1 Hz, 1H), 5.94 (t, *J* = 4.3 Hz, 2H), 3.91 (s, 3H), 3.73 (d, *J* = 1.3 Hz, 3H).
^{13}C NMR (101 MHz, Methanol-d_4) δ 168.78, 164.98 (d, *J* = 248.5 Hz), 162.93, 147.81, 146.01, 144.74, 141.97 (2C), 137.48, 132.88 (d, *J* = 8.0 Hz, 2C), 130.57 (2C), 129.52, 126.52, 119.87, 117.66, 117.61 (d, *J* = 22.1 Hz, 2C), 116.92, 65.00, 56.45, 52.27.
^{19}F NMR (376 MHz, Methanol-d_4) δ -112.90.
HRMS: Calculated for: $C_{23}H_{21}O_3NF$ $[M]^+$ 378.1500, found 378.1498.
IR: 1710 1597 1498 1240 1222 1167 1022 831 681.
m.p. 171-173 °C.

(*E*)-3-(4,5-Dimethoxy-2-(3-methoxy-3-oxoprop-1-en-1-yl)phenyl)-1-(4-fluorobenzyl)pyridin-1-ium bromide (5l)

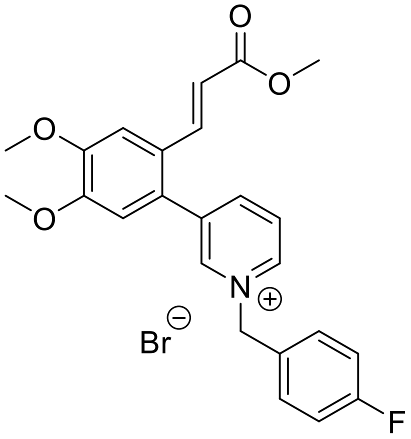

Prepared according to general procedure C1 using **22l** (129 mg, 0.43 mmol) and 4-fluorobenzyl bromide (87 µL, 0.70 mmol) in MeCN (3 mL) at 80 °C to yield the titled compound as a yellow solid (178 mg, 84%) and a mixture of isomers (*E:Z* = 33:1).
^{1}H NMR (400 MHz, DMSO-d_6) δ 9.49 (t, *J* = 1.6 Hz, 1H), 9.26 (dt, *J* = 6.2, 1.3 Hz, 1H), 8.59 (dt, *J* = 8.1, 1.4 Hz, 1H), 8.27 (dd, *J* = 8.1, 6.1 Hz, 1H), 7.83 – 7.73 (m, 2H), 7.53 (s, 1H), 7.35 – 7.20 (m, 4H), 6.73 (d, *J* = 15.7 Hz, 1H), 5.98 (s, 2H), 3.92 (s, 3H), 3.89 (s, 3H), 3.68 (s, 3H).
^{13}C NMR (101 MHz, DMSO-d_6) δ 166.57, 162.63 (d, *J* = 246.4 Hz), 150.69, 150.00, 146.33, 144.94, 143.03, 140.73, 139.24, 131.74 (d, *J* = 8.7 Hz, 2C), 130.39 (d, *J* = 3.1 Hz), 128.05, 127.90, 125.02, 119.07, 116.12 (d, *J* = 21.8 Hz, 2C), 113.82, 110.04, 62.28, 56.13, 56.07, 51.47.
^{19}F NMR (376 MHz, DMSO-d_6) δ -112.00.
HRMS: Calculated for: $C_{24}H_{23}O_4NF$ $[M]^+$ 408.1606, found 408.1604.
IR: 1704 1504 1281 1250 1220 1208 1191 1145 1020 683.
m.p. 222-223 °C.

(*E*)-1-(4-Fluorobenzyl)-3-(2-(3-methoxy-3-oxoprop-1-en-1-yl)naphthalen-1-yl)pyridin-1-ium bromide (5m)

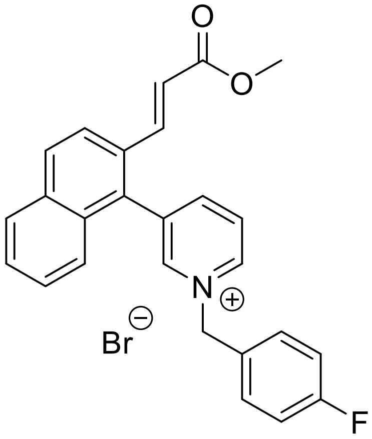

Prepared according to general procedure C1 using **22m** (119 mg, 0.41 mmol) and 4-fluorobenzyl bromide (87 µL, 0.70 mmol) in MeCN (3 mL) at 80 °C to yield the titled compound as a beige solid (170 mg, 85%) and a mixture of isomers (*E:Z* = 10:1).

^{1}H NMR (400 MHz, Methanol-d_4) δ 9.32 (dt, *J* = 6.2, 1.4 Hz, 1H), 9.21 (d, *J* = 1.7 Hz, 1H), 8.64 (dt, *J* = 8.0, 1.5 Hz, 1H), 8.36 (dd, *J* = 8.0, 6.1 Hz, 1H), 8.13 (d, *J* = 8.8 Hz, 1H), 7.70 – 7.58 (m, 4H), 7.54 (ddd, *J* = 8.3, 6.9, 1.4 Hz, 2H), 7.37 (d, *J* = 8.5 Hz, 1H), 7.32 (d, *J* = 15.8 Hz, 1H), 7.28 – 7.18 (m, 2H), 6.68 (d, *J* = 15.8 Hz, 1H), 5.97 (d, *J* = 4.9 Hz, 2H), 3.75 (s, 3H).

^{13}C NMR (101 MHz, Methanol-d_4) δ 168.45, 164.95 (d, *J* = 248.2 Hz), 149.45, 147.02, 145.71, 142.26, 140.31, 135.58, 133.43, 133.07, 132.62, 132.61 (d, *J* = 8.8 Hz, 2C), 131.97, 130.81, 129.87, 129.71, 129.24, 128.96, 126.78, 124.30, 122.52, 117.65 (d, *J* = 22.3 Hz, 2C), 65.15, 52.43.

^{19}F NMR (376 MHz, Methanol-d_4) δ -112.96.

HRMS: Calculated for: $C_{26}H_{21}O_2NF$ $[M]^+$ 398.1551, found 398.1552.

IR: 1709 1632 1512 1489 1456 1320 1301 1233 1178 1162 1147 815 783 756 682.

m.p. 228-229 °C.

(*E*)-3-(2-(3-Ethoxy-3-oxoprop-1-en-1-yl)phenyl)-1-(4-fluorobenzyl)pyridin-1-ium bromide (5n)

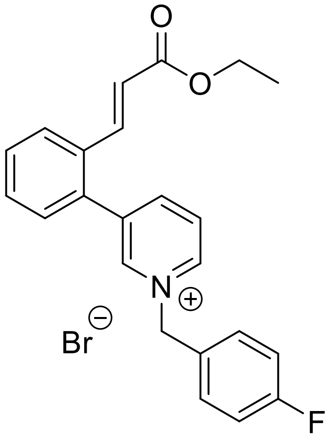

Prepared according to general procedure C1 using **22n** (190 mg, 0.75 mmol) and 4-fluorobenzyl bromide (124 µL, 1.00 mmol) in acetone (3 mL) at room temperature to yield the titled compound as a white solid (271 mg, 82%) and a mixture of isomers (*E:Z* = 50:1).

^{1}H NMR (400 MHz, Methanol-d_4) δ 9.24 (t, *J* = 1.5 Hz, 1H), 9.20 (dd, *J* = 6.0, 1.4 Hz, 1H), 8.62 (dt, *J* = 8.1, 1.5 Hz, 1H), 8.25 (dd, *J* = 8.1, 6.1 Hz, 1H), 7.89 (dd, *J* = 5.7, 3.0 Hz, 1H), 7.77 – 7.67 (m, 2H), 7.64 – 7.58 (m, 3H), 7.48 (d, *J* = 15.8 Hz, 1H), 7.27 – 7.16 (m, 2H), 6.56 (d, *J* = 15.8 Hz, 1H), 5.99 (s, 2H), 4.21 (q, *J* = 7.1 Hz, 2H), 1.28 (t, *J* = 7.1 Hz, 3H).

^{13}C NMR (101 MHz, Methanol-d_4) δ 167.81, 164.89 (d, J = 248.4 Hz), 147.77, 145.89, 144.68, 142.16, 142.08, 135.63, 134.38, 132.90 (d, J = 8.7 Hz, 2C), 132.04, 131.86, 131.71, 130.60 (d, J = 3.3 Hz), 129.52, 128.96, 123.15, 117.56 (d, J = 22.1 Hz, 2C), 64.90, 61.86, 14.57.
^{19}F NMR (376 MHz, Methanol-d_4) δ -112.83.
HRMS: Calculated for: $C_{23}H_{21}O_2NF$ $[M]^+$ 362.1551, found 362.1550.
IR: 1719 1508 1313 1222 1172 1157 820 769 754 683.
m.p. 167-168 °C.

(*E*)-3-(2-(3-Ethoxy-2-methyl-3-oxoprop-1-en-1-yl)phenyl)-1-(4-fluorobenzyl)pyridin-1-ium bromide (5o)

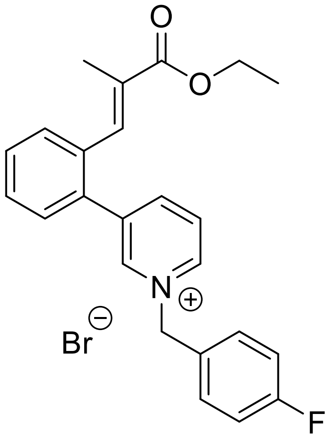

Prepared according to general procedure C2 using **22o** (165 mg, 0.62 mmol) and 4-fluorobenzyl bromide (124 µL, 1.00 mmol) in solvent (3 mL) at room temperature to yield the titled compound as a white solid (271 mg, 96%) and a mixture of isomers (*E:Z* = 9:1).
^{1}H NMR (400 MHz, Methanol-d_4) δ 9.16 – 9.09 (m, 2H), 8.62 (dt, J = 8.1, 1.5 Hz, 1H), 8.20 (dd, J = 8.1, 6.0 Hz, 1H), 7.70 – 7.57 (m, 5H), 7.51 (dq, J = 7.4, 1.1 Hz, 1H), 7.43 (d, J = 1.7 Hz, 1H), 7.28 – 7.15 (m, 2H), 5.93 (s, 2H), 4.18 (q, J = 7.1 Hz, 2H), 1.85 (d, J = 1.5 Hz, 3H), 1.25 (t, J = 7.1 Hz, 3H).
^{13}C NMR (101 MHz, Methanol-d_4) δ 168.85, 164.91 (d, J = 248.5 Hz), 147.42, 145.57, 144.44, 142.79, 137.64, 135.85, 134.92, 133.00, 132.78 (d, J = 8.6 Hz, 2C), 131.48, 131.33, 131.22, 130.59 (d, J = 3.2 Hz), 130.46, 129.40, 117.59 (d, J = 22.1 Hz, 2C), 64.85, 62.20, 14.48, 14.31.
^{19}F NMR (376 MHz, Methanol-d_4) δ -112.78.
HRMS: Calculated for: $C_{24}H_{23}O_2NF$ $[M]^+$ 376.1707, found 376.1706.
IR: 1702 1509 1246 1225 1120 1106 765.
m.p. 167-168 °C.

(*E*)-1-(4-Fluorobenzyl)-3-(2-(3-(methoxy(methyl)amino)-3-oxoprop-1-en-1-yl)phenyl)pyridin-1-ium bromide (5p)

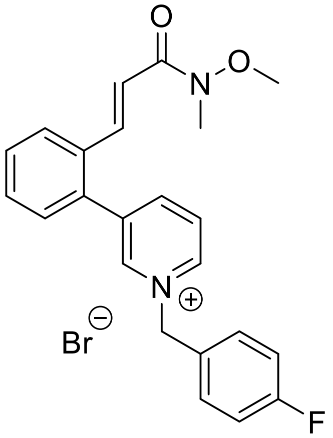

Prepared according to general procedure C2 using **22p** (185 mg, 0.69 mmol) and 4-fluorobenzyl bromide (124 µL, 1.00 mmol) in MeCN (2 mL) at 80 °C to yield the titled compound as an orange solid (172 mg, 54%).

^{1}H NMR (400 MHz, Methanol-*d*$_4$) δ 9.21 – 9.14 (m, 2H), 8.62 (dt, *J* = 8.0, 1.5 Hz, 1H), 8.24 (dd, *J* = 8.1, 6.1 Hz, 1H), 7.94 (dd, *J* = 7.1, 2.0 Hz, 1H), 7.71 – 7.65 (m, 2H), 7.65 – 7.57 (m, 3H), 7.43 (d, *J* = 15.6 Hz, 1H), 7.26 – 7.16 (m, 2H), 7.13 (d, *J* = 15.6 Hz, 1H), 5.96 (s, 2H), 3.79 (s, 3H), 3.29 (s, 3H).

^{13}C NMR (101 MHz, Methanol-*d*$_4$) δ 167.50, 164.90 (d, *J* = 248.1 Hz), 147.77, 145.97, 144.56, 142.27, 140.79, 135.65, 134.96, 132.89 (d, *J* = 8.6 Hz, 2C), 131.96, 131.71, 131.61, 130.56 (d, *J* = 3.4 Hz), 129.57, 128.95, 120.86, 117.59 (d, *J* = 22.1 Hz, 2C), 64.92, 62.74, 32.67.

^{19}F NMR (376 MHz, Methanol-*d*$_4$) δ -112.86.

HRMS: Calculated for: $C_{23}H_{22}O_2N_2F$ $[M]^+$ 377.1660, found 377.1665.

IR: 1649 1602 1511 1422 1383 1223 1033 824 767 691.

m.p. 176-177 °C.

(*E*)-3-(2-(2-Cyanovinyl)phenyl)-1-(4-fluorobenzyl)pyridin-1-ium bromide (5q)

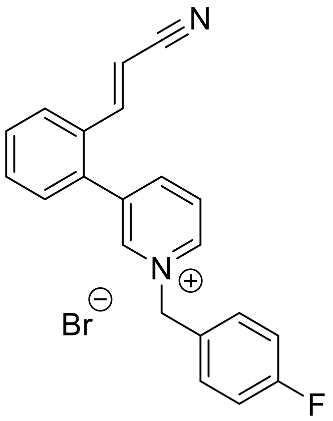

Prepared according to general procedure C1 using **22q** (63 mg, 0.30 mmol) 4-fluorobenzyl bromide (56 µL, 0.45 mmol) in MeCN (1 mL) at 50 °C to yield the titled compound as a white solid (109 mg, 92%).

^{1}H NMR (500 MHz, Methanol-*d*$_4$) δ 9.20 (d, *J* = 2.1 Hz, 1H), 9.17 (dd, *J* = 6.2, 1.7 Hz, 1H), 8.60 (dt, *J* = 8.0, 1.5 Hz, 1H), 8.24 (dd, *J* = 8.0, 6.1 Hz, 1H), 7.92 – 7.87 (m, 1H), 7.71 (ddd, *J* = 8.9, 5.2, 1.9 Hz, 2H), 7.68 – 7.62 (m, 2H), 7.60 – 7.57 (m, 1H), 7.39 (d, *J* = 16.4 Hz, 1H), 7.28 – 7.20 (m, 2H), 6.32 (d, *J* = 16.5 Hz, 1H), 5.96 (d, *J* = 2.1 Hz, 2H).

^{13}**C NMR** (126 MHz, Methanol-d_4) δ 164.99 (d, *J* = 248.8 Hz), 148.55, 147.82, 145.86, 144.77, 141.81, 135.45, 134.04, 132.84 (d, *J* = 8.6 Hz, 2C), 132.47, 132.11, 131.78, 130.56 (d, *J* = 3.2 Hz), 129.64, 128.39, 118.83, 117.69 (d, *J* = 22.1 Hz, 2C), 101.56, 65.00.
^{19}**F NMR** (471 MHz, Methanol-d_4) δ -112.79.
HRMS: Calculated for: $C_{21}H_{16}N_2F$ [M]$^+$ 315.1292, found 315.1292.
IR: 2222 1512 1223 1163 816 757 680.
m.p. 169-170 °C.

(*E*)-1-(4-Fluorobenzyl)-3-methoxy-5-(2-(3-methoxy-3-oxoprop-1-en-1-yl)phenyl)pyridin-1-ium (5r)

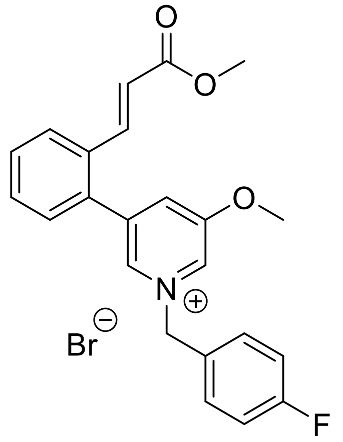

Prepared according to general procedure C2 using **22r** (120 mg, 0.47 mmol) and 4-fluorobenzyl bromide (92 µL, 0.75 mmol) in MeCN (2 mL) at 50 °C to yield the titled compound as a yellow solid (179 mg, 83%) and a mixture of isomers (*E:Z* = 13:1).
^{1}**H NMR** (400 MHz, Methanol-d_4) δ 9.00 (dd, *J* = 2.6, 1.3 Hz, 1H), 8.73 (d, *J* = 1.3 Hz, 1H), 8.19 (dd, *J* = 2.5, 1.4 Hz, 1H), 7.88 (dd, *J* = 5.6, 3.6 Hz, 1H), 7.69 (dd, *J* = 8.4, 5.2 Hz, 2H), 7.65 – 7.53 (m, 3H), 7.48 (d, *J* = 15.8 Hz, 1H), 7.26 – 7.16 (m, 2H), 6.55 (d, *J* = 15.8 Hz, 1H), 5.90 (s, 2H), 4.12 (s, 3H), 3.76 (s, 3H).
^{13}**C NMR** (101 MHz, Methanol-d_4) δ 168.34, 164.91 (d, *J* = 248.4 Hz), 160.36, 142.77, 142.47, 138.10, 135.83, 134.34, 132.85, 132.68 (d, *J* = 8.7 Hz, 2C), 132.14, 131.94, 131.83, 131.67, 130.71 (d, *J* = 3.5 Hz), 128.86, 122.52, 117.52 (d, *J* = 22.3 Hz, 2C), 65.14, 58.54, 52.39.
^{19}**F NMR** (376 MHz, Methanol-d_4) δ -112.97.
HRMS: Calculated for: $C_{23}H_{21}O_3NF$ [M]$^+$ 378.1500, found 378.1501.
IR: 1707 1592 1510 1318 1225 1195 1160 767.
m.p. 95-97 °C.

(*E*)-3-(Benzyloxy)-1-(4-fluorobenzyl)-5-(2-(3-methoxy-3-oxoprop-1-en-1-yl)phenyl)pyridin-1-ium bromide (5s)

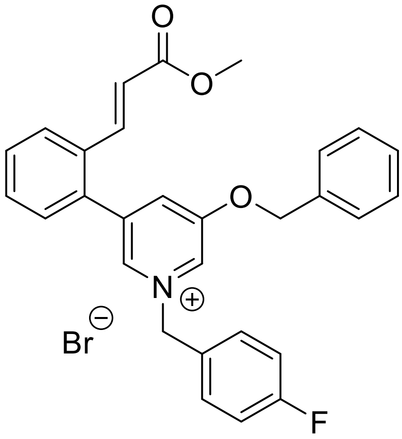

Prepared according to general procedure C2 using **22s** (162 mg, 0.47 mmol) and 4-fluorobenzyl bromide (124 µL, 1.00 mmol) in acetone (2 mL) at 30 °C to yield the titled compound as a white solid (218 mg, 87%) and a mixture of isomers (*E:Z* = 14:1).

^{1}H NMR (400 MHz, Methanol-d_4) δ 9.03 (dd, *J* = 2.4, 1.3 Hz, 1H), 8.76 (d, *J* = 1.5 Hz, 1H), 8.23 (dt, *J* = 2.5, 1.2 Hz, 1H), 7.91 – 7.82 (m, 1H), 7.66 (ddd, *J* = 8.8, 5.2, 1.4 Hz, 2H), 7.60 (dd, *J* = 5.8, 3.3 Hz, 2H), 7.57 – 7.53 (m, 1H), 7.53 – 7.43 (m, 3H), 7.43 – 7.34 (m, 3H), 7.19 (t, *J* = 8.7 Hz, 2H), 6.54 (d, *J* = 15.8 Hz, 1H), 5.89 (s, 2H), 5.43 (s, 2H), 3.75 (s, 3H).

^{13}C NMR (101 MHz, Methanol-d_4) δ 168.31, 164.88 (d, *J* = 248.2 Hz), 159.23, 142.73, 142.42, 138.34, 135.89, 135.75, 134.31, 133.21, 133.17, 132.69 (d, *J* = 8.7 Hz, 2C), 131.94, 131.83, 131.68, 130.64 (d, *J* = 3.3 Hz), 129.89, 129.86 (2C), 129.31 (2C), 128.89, 122.56, 117.53 (d, *J* = 22.1 Hz, 2C), 73.60, 65.13, 52.41.

^{19}F NMR (376 MHz, Methanol-d_4) δ -112.88.

HRMS: Calculated for: $C_{29}H_{25}O_3NF$ $[M]^+$ 454.1813, found 454.1812.

IR: 1709 1593 1317 1223 1196 1161 765 689.

m.p. 98-100 °C.

(*E*)-3-Fluoro-1-(4-fluorobenzyl)-5-(2-(3-methoxy-3-oxoprop-1-en-1-yl)phenyl)pyridin-1-ium bromide (5t)

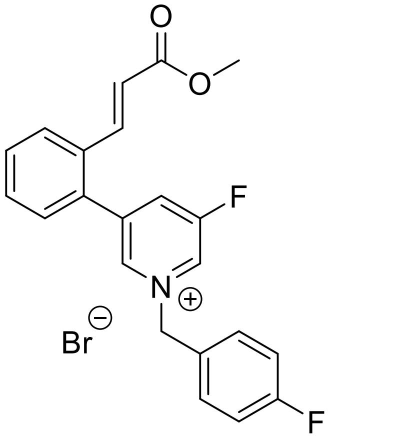

Prepared according to general procedure C1 using **22t** (121 mg, 0.47 mmol) and 4-fluorobenzyl bromide (124 µL, 1.00 mmol) in solvent (2 mL) at 30 °C to yield the titled compound as a white solid (71 mg, 34%) and a mixture of isomers (*E:Z* = 20:1).

^{1}H NMR (500 MHz, Methanol-d_4) δ 9.41 (q, J = 2.3, 1.8 Hz, 1H), 9.11 (d, J = 1.4 Hz, 1H), 8.65 (dt, J = 7.9, 1.9 Hz, 1H), 7.94 – 7.89 (m, 1H), 7.71 – 7.62 (m, 4H), 7.60 – 7.56 (m, 1H), 7.50 (d, J = 15.9 Hz, 1H), 7.28 – 7.19 (m, 2H), 6.60 (d, J = 15.8 Hz, 1H), 5.93 (s, 2H), 3.77 (s, 3H).
^{13}C NMR (126 MHz, Methanol-d_4) δ 168.34, 165.12 (d, J = 248.6 Hz), 162.01 (d, J = 256.9 Hz), 143.91 (d, J = 7.7 Hz), 143.24, 142.13, 135.16 (d, J = 19.0 Hz), 134.99, 134.82 (d, J = 1.7 Hz), 134.69, 134.52, 133.01 (d, J = 8.7 Hz, 2C), 132.08 (d, J = 7.7 Hz), 131.93, 130.02 (d, J = 3.3 Hz), 129.08, 123.11, 117.67 (d, J = 22.2 Hz, 2C), 65.66, 52.43.
^{19}F NMR (471 MHz, Methanol-d_4) δ -112.60, -116.21.
HRMS: Calculated for: $C_{22}H_{18}O_2NF_2$ [M]$^+$ 366.1300, found 366.1305.
IR: 1718 1702 1602 1512 1215 1174 1162.
m.p. 150-151 °C.

(*E*)-1-(4-Fluorobenzyl)-5-(2-(3-methoxy-3-oxoprop-1-en-1-yl)phenyl)-2-methylpyridin-1-ium bromide (5u)

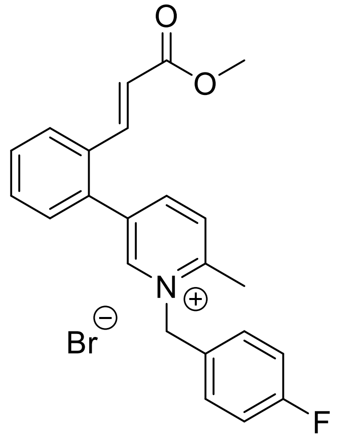

Prepared according to general procedure C1 using **22u** (118 mg, 0.47 mmol) and 4-fluorobenzyl bromide (124 µL, 1.00 mmol) in acetone (2 mL) at 30 °C to yield the titled compound as a white solid (73 mg, 36%) and a mixture of isomers (*E:Z* = 14:1).
^{1}H NMR (400 MHz, Methanol-d_4) δ 8.99 (d, J = 1.9 Hz, 1H), 8.53 (dd, J = 8.2, 2.0 Hz, 1H), 8.16 (d, J = 8.2 Hz, 1H), 7.95 – 7.86 (m, 1H), 7.64 – 7.54 (m, 4H), 7.47 (dd, J = 8.6, 5.2 Hz, 2H), 7.23 (t, J = 8.7 Hz, 2H), 6.60 (d, J = 15.8 Hz, 1H), 5.93 (s, 2H), 3.78 (s, 3H), 2.92 (s, 3H).
^{13}C NMR (101 MHz, Methanol-d_4) δ 168.42, 164.58 (d, J = 247.9 Hz), 156.60, 147.53, 146.70, 142.51, 139.67, 135.73, 134.37, 131.93 (2C), 131.80 (2C), 131.65 (d, J = 13.7 Hz, 2C), 129.45 (d, J = 3.4 Hz), 128.91, 122.59, 117.64 (d, J = 22.1 Hz, 2C), 61.90, 52.39, 20.57.
^{19}F NMR (376 MHz, Methanol-d_4) δ -113.69.
HRMS: Calculated for: $C_{23}H_{21}O_2NF$ [M]$^+$ 362.1551, found 362.1550.
IR: 1712 1631 1510 1321 1227 1197 1177 1161 833 768.
m.p. 200-202 °C.

(*E*)-3-(2-(3-Methoxy-3-oxoprop-1-en-1-yl)phenyl)-1-(4-((triisopropylsilyl)oxy)benzyl)pyridin-1-ium bromide (5v)

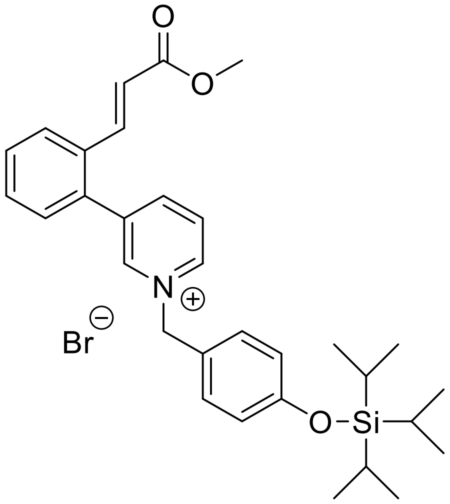

Prepared according to general procedure C2 using **22a** (239 mg, 1.00 mmol) and **25** (515 µL, 1.50 mmol) in acetone (3 mL) at room temperature to yield the titled compound as a white solid (557 mg, 96%) and a mixture of isomers (*E:Z* = 20:1).

^{1}H NMR (400 MHz, Methanol-d_4) δ 9.16 (dt, *J* = 6.2, 1.3 Hz, 1H), 9.13 (d, *J* = 1.7 Hz, 1H), 8.58 (dt, *J* = 8.1, 1.5 Hz, 1H), 8.23 (dd, *J* = 8.0, 6.1 Hz, 1H), 7.94 – 7.86 (m, 1H), 7.66 – 7.46 (m, 6H), 7.03 – 6.95 (m, 2H), 6.57 (d, *J* = 15.8 Hz, 1H), 5.91 (s, 2H), 3.76 (s, 3H), 1.37 – 1.22 (m, 3H), 1.11 (d, *J* = 7.4 Hz, 18H).

^{13}C NMR (101 MHz, Methanol-d_4) δ 168.23, 158.84, 147.54, 145.55, 144.55, 142.30, 141.89, 135.74, 134.27, 132.27 (2C), 132.01, 131.90, 131.67, 129.44, 128.89, 126.85, 122.68, 122.01 (2C), 65.39, 52.40, 18.34 (6C), 13.80 (3C).

HRMS: Calculated for: $C_{31}H_{40}O_3NSi$ $[M]^+$ 502.2772, found 502.2749.

IR: 2943 2865 1716 1509 1266 1195 1172 908 882 687.

m.p. 72-74°C.

1-(4-Fluorobenzyl)-3-(2-(4-methoxy-4-oxobut-2-en-2-yl)phenyl)pyridin-1-ium bromide (5w)

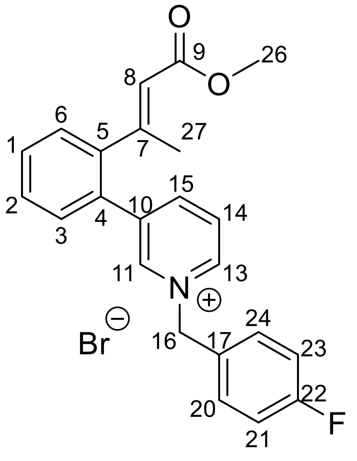

Prepared according to general procedure C2 using **22w** (459 mg, 1.81 mmol) and 4-fluorobenzyl bromide (520 µL, 4.17 mmol) in MeCN (5 mL) at 80 °C to yield the titled compound as a yellow solid (482 mg, 60%) and a mixture of isomers (*E:Z* = 7:3).

^{1}H NMR (400 MHz, Methanol-d_4) δ 9.15 (dt, *J* = 6.1, 1.4 Hz, *E*-13, 1H and *Z*-13, 1H), 9.09 (t, *J* = 1.7 Hz, *E*-11, 1H), 8.88 (s, *Z*-11, 1H), 8.65 (dt, *J* = 8.2, 1.5 Hz, *E*-15, 1H), 8.60 (dt, *J* = 8.1, 1.5 Hz, *Z*-15, 1H), 8.20 (dd, *J* = 8.0, 6.2 Hz, *E*-14, 1H and *Z*-14, 1H), 7.67 – 7.55 (m, *E*-1, *E*-3, *E*-20, *E*-24, 4H and *Z*-1, *Z*-3,

Z-20, *Z*-24, 4H), 7.55 – 7.49 (m, *E*-2, 1H and *Z*-2, 1H), 7.48 – 7.42 (m, *E*-6, 1H), 7.30 – 7.17 (m, *E*-21, *E*-23, 2H and *Z*-6, *Z*-21, *Z*-23, 3H), 5.92 (d, *J* = 4.8 Hz, *E*-16, 2H and *Z*-16, 2H), 5.70 (q, *J* = 1.4 Hz, *E*-8, 1H), 5.67 (d, *J* = 1.5 Hz, *Z*-8, 1H), 3.65 (s, *E*-26, 3H), 3.40 (s, *Z*-26, 3H), 2.15 (d, *J* = 1.4 Hz, *E*-27, 3H), 1.91 (d, *J* = 1.5 Hz, *Z*-27, 3H).
^{13}C NMR (101 MHz, Methanol-*d*$_4$) δ 167.31 (*E*-9),167.02 (*Z*-9), 164.97 (d, *J* = 248.7 Hz, *Z*-22), 164.91 (d, *J* = 248.5 Hz, *E*-22), 156.65 (*E*-5), 155.21 (*Z*-5), 147.20 (*E*-15), 147.18 (*Z*-15), 145.16 (*E*-11), 144.83 (*Z*-11), 144.51 (*E*-13 and *Z*-13), 144.22 (*E*-7), 143.39 (*E*-10), 143.04 (*Z*-10), 141.54 (*Z*-7), 133.11 – 132.75 (m, *E*-4 and *Z*-4, *Z*-20, *Z*-24, 3C), 132.53 (d, *J* = 8.8 Hz, *E*-20, *E*-24, 2C), 131.96 (*E*-2), 131.47 (*E*-3), 131.26 (*Z*-2), 130.85 (*Z*-3), 130.78 (d, *J* = 3.2 Hz, *E*-17), 130.67 (d, *J* = 3.3 Hz, *Z*-17), 130.40 (*E*-1), 130.13 (*E*-6), 129.72 (*Z*-14), 129.65 (*E*-14), 129.15 (*Z*-1), 128.86 (*Z*-6), 123.74 (*E*-8), 121.79 (*Z*-8), 117.70 (d, *J* = 22.3 Hz, *E*-21, *E*-23, 2C), 117.61 (d, *J* = 22.1 Hz, *Z*-21, *Z*-23, 2C), 64.89 (*E*-16), 64.78 (*Z*-16), 51.69 (*E*-26 and *Z*-26), 27.28 (*Z*-27), 21.23 (*E*-27).
^{19}F NMR (376 MHz, Methanol-*d*$_4$) δ -112.71 (*Z*), -112.74 (*E*).
HRMS: Calculated for: $C_{23}H_{21}O_2NF$ [M]$^+$ 362.1551, found 362.1540.
IR: 1710 1630 1604 1509 1434 1225 1192 1163 1033 823 768 685.
m.p. 59-61 °C.

Preparation of indenopyridines

Methyl 2-(2-benzyl-2*H*-indeno[1,2-c]pyridin-5-yl)acetate (6a)

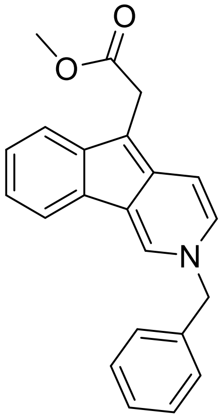

Prepared according to general procedure D using **5a’** (41 mg, 0.10 mmol) to yield the titled compound as an orange oil (26 mg, 78%).
^{1}H NMR (400 MHz, Acetone-*d*$_6$) δ 8.65 (d, *J* = 1.6 Hz, 1H), 8.10 (dt, *J* = 7.7, 1.0 Hz, 1H), 7.66 (dt, *J* = 8.0, 1.0 Hz, 1H), 7.35 (dtt, *J* = 11.9, 7.3, 1.6 Hz, 7H), 7.18 (d, *J* = 7.3 Hz, 1H), 7.11 (ddd, *J* = 7.9, 7.0, 1.1 Hz, 1H), 5.32 (s, 2H), 3.92 (s, 2H), 3.56 (s, 3H).
^{13}C NMR (101 MHz, Acetone-*d*$_6$) δ 172.89, 143.17, 138.69, 130.50, 130.44, 129.72 (2C), 128.87, 128.26 (2C), 128.01, 127.65, 125.82, 121.18, 120.52, 118.96, 117.92, 109.04, 102.45, 60.53, 51.64, 31.62.
HRMS: Calculated for: $C_{22}H_{20}O_2N$ [M+H]$^+$ 330.1489, found 330.1483.
IR: 1730 1640 1599 1238 1165 1130.

Methyl 2-(2-(4-fluorobenzyl)-2*H*-indeno[1,2-c]pyridin-5-yl)acetate (6b)

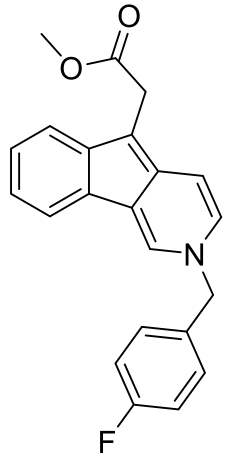

Prepared according to general procedure D using **5b** (43 mg, 0.10 mmol) to yield the titled compound as an orange oil (29 mg, 84%).

^{1}H NMR (400 MHz, Acetone-d_6) δ 8.64 (d, *J* = 1.5 Hz, 1H), 8.09 (dt, *J* = 7.8, 1.0 Hz, 1H), 7.66 (dt, *J* = 7.9, 1.0 Hz, 1H), 7.37 (dddd, *J* = 16.9, 10.0, 6.5, 1.8 Hz, 4H), 7.21 – 7.07 (m, 4H), 5.31 (s, 2H), 3.92 (s, 2H), 3.57 (s, 3H).

^{13}C NMR (101 MHz, Acetone-d_6) δ 172.89, 163.34 (d, *J* = 244.7 Hz), 143.18, 134.83 (d, *J* = 3.3 Hz), 130.45 (d, *J* = 8.3 Hz, 2C), 130.45, 130.31, 127.85, 127.66, 125.87, 121.24, 120.53, 119.02, 117.95, 116.43 (d, *J* = 21.9 Hz, 2C), 109.12, 102.59, 59.73, 51.65, 31.60.

^{19}F NMR (376 MHz, Acetone-d_6) δ -115.78.

HRMS: Calculated for: $C_{22}H_{18}O_2NFNa$ $[M+Na]^+$ 370.1214, found 370.1207.

IR: 1730 1639 1599 1510 1224 1158 1129.

Methyl 2 -(2-(4-methoxybenzyl)-2*H*-indeno[1,2-c]pyridin-5-yl)acetate (6c)

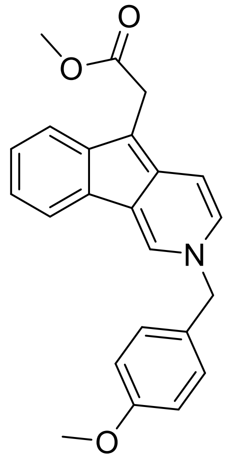

Prepared according to general procedure D using **5c** (40 mg, 0.10 mmol) to yield the titled compound as a yellow oil (24 mg, 67%).

^{1}H NMR (400 MHz, Acetone-d_6) δ 8.65 (d, *J* = 1.5 Hz, 1H), 8.10 (dt, *J* = 7.8, 1.0 Hz, 1H), 7.64 (dt, *J* = 8.0, 1.0 Hz, 1H), 7.40 – 7.27 (m, 4H), 7.17 (d, *J* = 7.3 Hz, 1H), 7.10 (ddd, *J* = 7.9, 7.1, 1.1 Hz, 1H), 6.95 – 6.88 (m, 2H), 5.26 (s, 2H), 3.91 (s, 2H), 3.76 (s, 3H), 3.56 (s, 3H).

^{13}C NMR (101 MHz, Acetone-d_6) δ 172.90, 160.59, 143.11, 130.60, 130.49, 130.30, 129.88 (2C), 127.84, 127.59, 125.75, 121.10, 120.48, 118.86, 117.88, 115.05 (2C), 109.01, 102.23, 60.14, 55.55, 51.62, 31.62.

HRMS: Calculated for: $C_{23}H_{22}O_3N$ $[M+H]^+$ 360.1594, found 360.1591.

IR: 1730 1639 1598 1513 1250 1164 1129.

Methyl 2-(2-methyl-2*H*-indeno[1,2-c]pyridin-5-yl)acetate (6d)

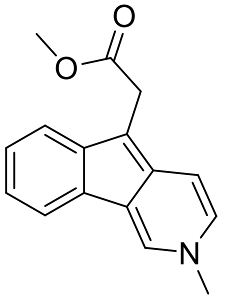

Prepared according to general procedure D using **5d** (38 mg, 0.10 mmol) to yield the titled compound as a yellow oil (8 mg, 32%).

^{1}H NMR (400 MHz, Acetone-d_6) δ 8.49 (d, *J* = 1.6 Hz, 1H), 8.09 (dt, *J* = 7.8, 1.0 Hz, 1H), 7.63 (dt, *J* = 8.0, 1.0 Hz, 1H), 7.35 (ddd, *J* = 8.0, 7.0, 1.2 Hz, 1H), 7.27 (dd, *J* = 7.2, 1.6 Hz, 1H), 7.16 (d, *J* = 7.3 Hz, 1H), 7.09 (ddd, *J* = 7.8, 7.0, 1.0 Hz, 1H), 3.96 (s, 3H), 3.91 (s, 2H), 3.56 (s, 3H).

^{13}C NMR (101 MHz, Acetone-d_6) δ 172.94, 142.98, 130.94, 130.48, 128.49, 127.34, 125.63, 120.87, 120.38, 118.68, 117.80, 108.83, 101.70, 51.60, 43.93, 31.64.

HRMS: Calculated for: $C_{16}H_{16}O_2N$ [M+H]$^+$ 254.1176, found 254.1171.

IR: 1729 1642 1598 1389 1174 1140.

Methyl 2-(2-ethyl-2*H*-indeno[1,2-c]pyridin-5-yl)acetate (6e)

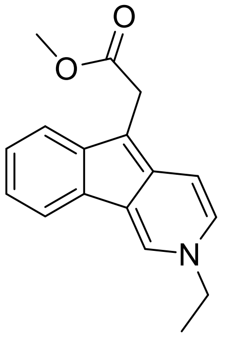

Prepared according to general procedure D using **5e** (35 mg, 0.10 mmol) to yield the titled compound as a yellow oil (10 mg, 36%).

^{1}H NMR (400 MHz, Acetone-d_6) δ 8.57 (d, *J* = 1.8 Hz, 1H), 8.10 (dt, *J* = 7.8, 1.0 Hz, 1H), 7.64 (dt, *J* = 8.0, 1.0 Hz, 1H), 7.35 (ddd, *J* = 8.3, 7.1, 1.3 Hz, 2H), 7.18 (d, *J* = 7.2 Hz, 1H), 7.09 (ddd, *J* = 7.9, 7.0, 1.0 Hz, 1H), 4.21 (q, *J* = 7.2 Hz, 2H), 3.92 (s, 2H), 3.57 (s, 3H), 1.50 (t, *J* = 7.2 Hz, 3H).

^{13}C NMR (101 MHz, Acetone-d_6) δ 172.96, 143.01, 130.77, 129.86, 127.40, 127.36, 125.61, 121.03, 120.42, 118.63, 117.78, 109.02, 101.66, 52.51, 51.61, 31.65, 17.11.

HRMS: Calculated for: $C_{17}H_{17}O_2NNa$ [M+Na]$^+$ 290.1152, found 290.1146.

IR: 1731 1640 1598 1350 1192 1168 1138.

Methyl 2-(2-phenethyl-2*H*-indeno[1,2-c]pyridin-5-yl)acetate (6f)

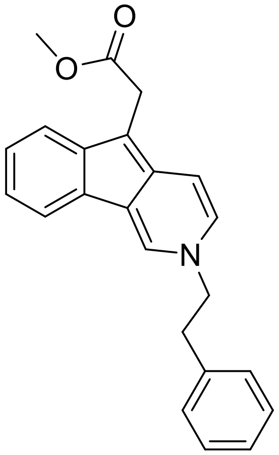

Prepared according to general procedure D using **5f** (42 mg, 0.10 mmol) to yield the titled compound as an orange oil (17 mg, 51%).

^{1}H NMR (400 MHz, Acetone-d_6) δ 8.49 (d, *J* = 1.5 Hz, 1H), 8.04 (dt, *J* = 7.8, 1.0 Hz, 1H), 7.64 (dt, *J* = 8.1, 1.0 Hz, 1H), 7.35 (ddd, *J* = 8.0, 7.0, 1.1 Hz, 1H), 7.31 – 7.21 (m, 6H), 7.12 (d, *J* = 7.3 Hz, 1H), 7.08 (ddd, *J* = 7.9, 7.0, 1.0 Hz, 1H), 4.43 – 4.36 (m, 2H), 3.91 (s, 2H), 3.57 (s, 3H), 3.22 (dd, *J* = 8.1, 6.7 Hz, 2H).

^{13}C NMR (101 MHz, Acetone-d_6) δ 172.94, 143.06, 138.90, 130.70, 130.17, 129.84 (2C), 129.39 (2C), 127.80, 127.53, 127.48, 125.65, 120.89, 120.38, 118.70, 117.83, 108.70, 101.88, 58.88, 51.61, 38.43, 31.65.

HRMS: Calculated for: $C_{23}H_{22}O_2N$ $[M+H]^+$ 344.1645, found 344.1640.

IR: 1729 1640 1598 1166 1131.

Methyl 2-(2-(4-fluorobenzyl)-8-methyl-2*H*-indeno[1,2-c]pyridin-5-yl)acetate (6g)

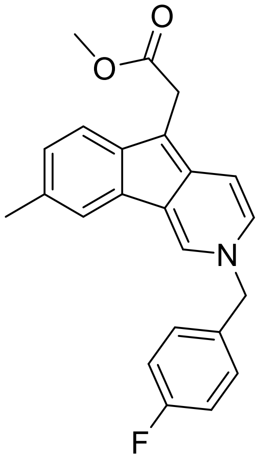

Prepared according to general procedure D using **5g** (44 mg, 0.10 mmol) to yield the titled compound as an orange oil (44 mg, 80%).

^{1}H NMR (400 MHz, Acetone-d_6) δ 8.57 (d, *J* = 1.6 Hz, 1H), 7.88 (dt, *J* = 1.7, 0.8 Hz, 1H), 7.54 (d, *J* = 8.0 Hz, 1H), 7.45 – 7.36 (m, 2H), 7.32 – 7.25 (m, 1H), 7.25 – 7.18 (m, 1H), 7.18 – 7.08 (m, 3H), 5.29 (s, 2H), 3.88 (s, 2H), 3.56 (s, 3H), 2.47 (s, 3H).

^{13}C NMR (101 MHz, Acetone-d_6) δ 172.89, 163.34 (d, *J* = 244.6 Hz), 141.23, 134.91 (d, *J* = 3.1 Hz), 130.47 (d, *J* = 8.3 Hz, 2C), 130.08, 129.81, 128.04, 127.96, 127.50, 127.45, 121.05, 120.59, 117.80, 116.42 (d, *J* = 21.6 Hz, 2C), 109.04, 102.46, 59.67, 51.62, 31.69, 21.74.

^{19}F NMR (376 MHz, Acetone-d_6) δ -115.85.

HRMS: Calculated for: $C_{23}H_{20}O_2NF$ $[M]^+$ 361.1473, found 361.1473.

IR: 1730 1639 1606 1510 1225 1156 1127.

Methyl 2-(2-(4-fluorobenzyl)-7-methoxy-2*H*-indeno[1,2-c]pyridin-5-yl)acetate (6h)

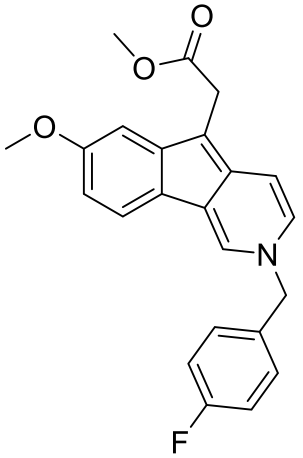

Prepared according to general procedure D using **5h** (46 mg, 0.10 mmol) to yield the titled compound as a yellow solid (30 mg, 79%).

^{1}H NMR (400 MHz, Acetone-d_6) δ 8.49 (d, *J* = 1.5 Hz, 1H), 7.96 (dd, *J* = 8.5, 0.5 Hz, 1H), 7.44 – 7.36 (m, 2H), 7.35 – 7.31 (m, 1H), 7.18 – 7.08 (m, 4H), 6.74 (dd, *J* = 8.5, 2.3 Hz, 1H), 5.31 (s, 2H), 3.88 (s, 2H), 3.87 (s, 3H), 3.57 (s, 3H).

^{13}C NMR (101 MHz, Acetone-d_6) δ 172.87, 163.33 (d, *J* = 244.6 Hz), 159.70, 144.84, 134.91 (d, *J* = 3.3 Hz), 131.60, 130.43 (d, *J* = 8.3 Hz, 2C), 128.71, 127.56, 121.44, 121.42 (2C), 116.42 (d, *J* = 21.7 Hz, 2C), 108.49, 108.32, 102.50, 100.42, 59.76, 55.48, 51.65, 31.65.

^{19}F NMR (376 MHz, Acetone-d_6) δ -115.88.

HRMS: Calculated for: $C_{23}H_{20}O_3NF$ $[M]^+$ 377.1422, found 377.1422.

IR: 1730 1601 1510 1226 1158 1132.

m.p. 136-138 °C.

Methyl 2-(6-fluoro-2-(4-fluorobenzyl)-2*H*-indeno[1,2-c]pyridin-5-yl)acetate (6i)

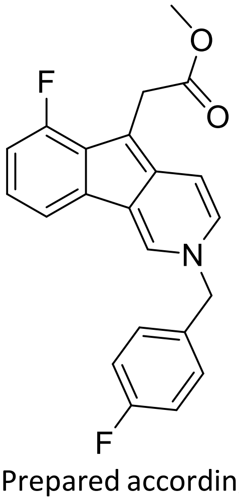

Prepared according to general procedure D using **5i** (45 mg, 0.10 mmol) to yield the titled compound as an orange solid (28 mg, 77%).

^{1}H NMR (400 MHz, Acetone-d_6) δ 8.77 (t, *J* = 1.6 Hz, 1H), 7.96 – 7.87 (m, 1H), 7.43 (ddd, *J* = 8.9, 5.0, 1.6 Hz, 3H), 7.22 (d, *J* = 7.3 Hz, 1H), 7.19 – 7.09 (m, 2H), 7.08 – 6.96 (m, 2H), 5.38 (d, *J* = 2.5 Hz, 2H), 4.06 (s, 2H), 3.59 (s, 3H).

^{13}C NMR (101 MHz, Acetone-d_6) δ 173.17, 163.41 (d, *J* = 245.0 Hz), 157.34 (d, *J* = 242.5 Hz), 134.62 (d, *J* = 3.3 Hz), 131.78, 130.99 (d, *J* = 9.6 Hz), 130.96, 130.57 (d, *J* = 8.5 Hz, 2C), 130.56 (d, *J* = 15.2

Hz), 128.23, 120.96 (d, J = 2.5 Hz), 119.10 (d, J = 6.6 Hz), 116.78 (d, J = 3.1 Hz), 116.50 (d, J = 21.9 Hz, 2C), 110.71 (d, J = 19.8 Hz), 109.74, 100.36 (d, J = 2.9 Hz), 59.92, 51.64, 32.32 (d, J = 3.3 Hz).
^{19}F NMR (376 MHz, Acetone-d_6) δ -115.63, -128.34.
HRMS: Calculated for: $C_{22}H_{17}O_2NF_2$ $[M]^+$ 365.1222, found 365.1226.
IR: 1732 1641 1510 1389 1223 1154 1142.
m.p. 128-130 °C.

Methyl 2-(9-fluoro-2-(4-fluorobenzyl)-2*H*-indeno[1,2-c]pyridin-5-yl)acetate (6j)

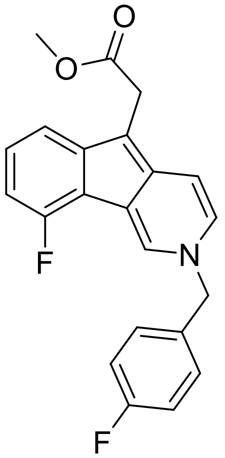

Prepared according to general procedure D using **5j** (45 mg, 0.10 mmol) to yield the titled compound as a yellow solid (23 mg, 64%).
^{1}H NMR (400 MHz, Acetone-d_6) δ 8.64 (d, J = 1.6 Hz, 1H), 7.46 (ddd, J = 8.9, 4.6, 2.0 Hz, 4H), 7.35 (td, J = 7.8, 5.4 Hz, 1H), 7.26 (d, J = 7.3 Hz, 1H), 7.16 (t, J = 8.8 Hz, 2H), 6.82 (ddd, J = 10.9, 7.8, 0.8 Hz, 1H), 5.46 (s, 2H), 3.93 (s, 2H), 3.57 (s, 3H).
^{13}C NMR (101 MHz, Acetone-d_6) δ 171.75, 162.42 (d, J = 244.7 Hz), 158.64 (d, J = 246.0 Hz), 144.88 (d, J = 7.5 Hz), 133.63 (d, J = 3.3 Hz), 132.11 (d, J = 4.7 Hz), 130.29, 129.63 (d, J = 8.3 Hz, 2C), 127.25, 125.57 (d, J = 7.9 Hz), 117.09 (d, J = 1.8 Hz), 115.51 (d, J = 21.9 Hz, 2C), 113.23 (d, J = 2.8 Hz), 112.93 (d, J = 16.5 Hz), 108.65, 103.15 (d, J = 19.2 Hz), 102.15 (d, J = 2.2 Hz), 58.88, 50.73, 30.59.
^{19}F NMR (376 MHz, Acetone-d_6) δ -115.62, -119.89.
HRMS: Calculated for: $C_{22}H_{17}O_2NF_2$ $[M]^+$ 365.1222, found 365.1226.
IR: 1731 1642 1613 1510 1222 1157 1142.
m.p. 101-102 °C.

Methyl 2-(2-(4-fluorobenzyl)-8-methoxy-2*H*-indeno[1,2-c]pyridin-5-yl)acetate (6k)

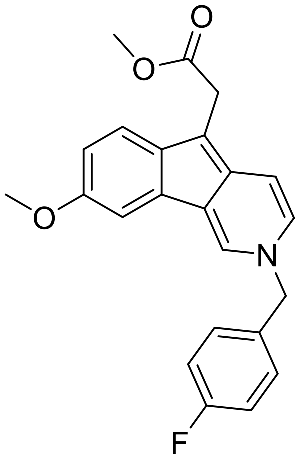

Prepared according to general procedure D using **5k** (46 mg, 0.10 mmol) to yield the titled compound as an orange solid (28 mg, 74%).

^{1}H NMR (400 MHz, Acetone-d_6) δ 8.60 (d, *J* = 1.5 Hz, 1H), 7.72 – 7.67 (m, 1H), 7.55 (dd, *J* = 8.6, 0.6 Hz, 1H), 7.44 – 7.34 (m, 2H), 7.24 (dd, *J* = 7.3, 1.6 Hz, 1H), 7.17 – 7.11 (m, 2H), 7.10 (d, *J* = 7.3 Hz, 1H), 7.06 (dd, *J* = 8.6, 2.4 Hz, 1H), 5.29 (s, 2H), 3.88 (s, 2H), 3.84 (s, 3H), 3.56 (s, 3H).

^{13}C NMR (101 MHz, Acetone-d_6) δ 172.88, 163.33 (d, *J* = 244.6 Hz), 154.87, 137.75, 134.96 (d, *J* = 3.0 Hz), 130.59, 130.39 (d, *J* = 8.3 Hz, 2C), 129.05, 128.42, 127.02, 120.99, 118.70, 116.42 (d, *J* = 21.9 Hz, 2C), 115.51, 109.30, 103.87, 102.22, 59.62, 55.90, 51.63, 31.73.

^{19}F NMR (376 MHz, Acetone-d_6) δ -115.90.

HRMS: Calculated for: $C_{23}H_{20}O_3NF$ $[M]^+$ 377.1422, found 377.1422.

IR: 1730 1638 1510 1224 1206 1128.

m.p. 35-36 °C.

Methyl 2-(2-(4-fluorobenzyl)-7,8-dimethoxy-2*H*-indeno[1,2-c]pyridin-5-yl)acetate (6l)

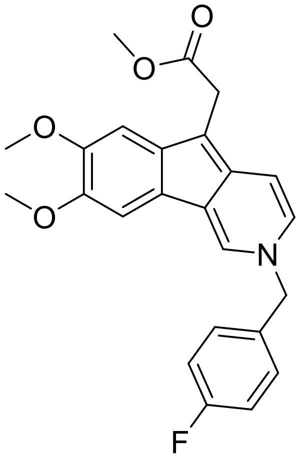

Prepared according to general procedure D using **5l** (49 mg, 0.10 mmol) to yield the titled compound as an orange oil (35 mg, 85%).

^{1}H NMR (400 MHz, Acetone-d_6) δ 8.49 (d, *J* = 1.6 Hz, 1H), 7.70 (s, 1H), 7.41 – 7.32 (m, 2H), 7.24 (dd, *J* = 7.3, 1.6 Hz, 1H), 7.22 (s, 1H), 7.16 – 7.07 (m, 3H), 5.28 (s, 2H), 3.90 (s, 3H), 3.89 (s, 2H), 3.84 (s, 3H), 3.58 (s, 3H).

^{13}C NMR (101 MHz, Acetone-d_6) δ 172.92, 163.31 (d, *J* = 244.7 Hz), 150.80, 145.55, 138.22, 135.03 (d, *J* = 3.0 Hz), 130.34 (d, *J* = 8.3 Hz, 2C), 129.38, 129.04, 126.32, 121.22, 120.20, 116.40 (d, *J* = 21.9 Hz, 2C), 108.59, 104.95, 102.14, 100.86, 59.71, 56.87, 56.08, 51.63, 31.81.

^{19}F NMR (376 MHz, Acetone-d_6) δ -115.93.

HRMS: Calculated for: $C_{24}H_{22}O_4NF$ $[M]^+$ 407.1527, found 407.1528.
IR: 1728 1606 1477 1202 1172 1132.

Methyl 2-(10-(4-fluorobenzyl)-10*H*-benzo[6,7]indeno[1,2-c]pyridin-7-yl)acetate (6m)

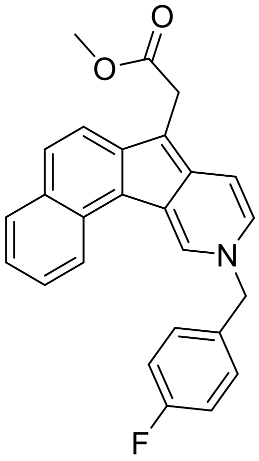

Prepared according to general procedure D using **5m** (48 mg, 0.10 mmol) to yield the titled compound as a red oil (34 mg, 87%).
^{1}H NMR (400 MHz, Acetone-d_6) δ 9.39 (d, *J* = 1.3 Hz, 1H), 8.66 (dt, *J* = 8.4, 0.9 Hz, 1H), 7.95 (dt, *J* = 8.1, 0.9 Hz, 1H), 7.90 (d, *J* = 8.7 Hz, 1H), 7.80 (d, *J* = 8.6 Hz, 1H), 7.54 (ddd, *J* = 8.3, 6.9, 1.4 Hz, 1H), 7.50 – 7.41 (m, 4H), 7.33 (ddd, *J* = 8.0, 6.9, 1.1 Hz, 1H), 7.19 – 7.09 (m, 2H), 5.56 (s, 2H), 4.06 (s, 2H), 3.58 (s, 3H).
^{13}C NMR (101 MHz, Acetone-d_6) δ 172.99, 163.38 (d, *J* = 244.7 Hz), 141.77, 134.82 (d, *J* = 3.1 Hz), 133.29, 131.56, 130.51 (d, *J* = 8.3 Hz, 2C), 130.41, 130.28, 130.09, 127.01, 126.99, 125.50, 123.59, 122.52, 120.76, 120.03, 118.77, 116.46 (d, *J* = 21.6 Hz, 2C), 110.68, 103.59, 60.41, 51.71, 31.48.
^{19}F NMR (376 MHz, Acetone-d_6) δ -115.67.
HRMS: Calculated for: $C_{26}H_{20}O_2NF$ $[M]^+$ 397.1473, found 397.1474.
IR: 1731 1510 1225 1160 1131.

Ethyl 2-(2-(4-fluorobenzyl)-2*H*-indeno[1,2-c]pyridin-5-yl)acetate (6n)

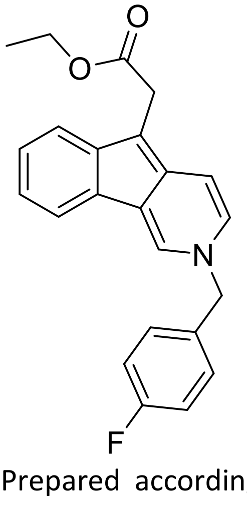

Prepared according to general procedure D2 using **5n** (44 mg, 0.10 mmol) to yield the titled compound as an orange oil (16 mg, 43%).
^{1}H NMR (400 MHz, Acetone-d_6) δ 8.67 (d, *J* = 1.5 Hz, 1H), 8.09 (dt, *J* = 7.8, 1.0 Hz, 1H), 7.66 (dt, *J* = 8.1, 1.0 Hz, 1H), 7.46 – 7.34 (m, 4H), 7.22 – 7.06 (m, 4H), 5.37 (s, 2H), 4.04 (q, *J* = 7.1 Hz, 2H), 3.89 (s, 2H), 1.16 (t, *J* = 7.1 Hz, 3H).

^{13}C NMR (101 MHz, Acetone-d_6) δ 172.36, 163.36 (d, J = 244.6 Hz), 143.24, 134.90 (d, J = 3.3 Hz), 130.48 (d, J = 8.4 Hz, 2C), 130.42, 130.30, 127.82, 127.69, 125.82, 121.29, 120.50, 118.99, 118.02, 116.45 (d, J = 21.7 Hz, 2C), 109.19, 102.80, 60.62, 59.76, 31.92, 14.62.
^{19}F NMR (376 MHz, Acetone-d_6) δ -115.85.
HRMS: Calculated for: $C_{23}H_{20}O_2NF$ $[M]^+$ 361.1473, found 361.1487.
IR: 1726 1640 1599 1528 1224 1158 1129.

Methyl 2-(2-(4-fluorobenzyl)-2*H*-indeno[1,2-c]pyridin-5-yl)propanoate (6o)

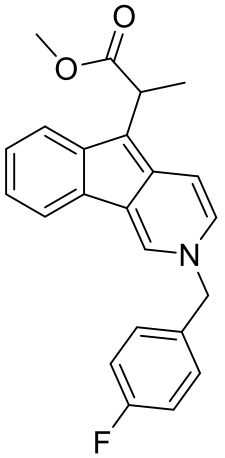

Prepared according to general procedure D using **5o** (46 mg, 0.10 mmol) to yield the titled compound as an orange oil (33 mg, 91%) and as a mixture of methyl and ethyl esters (2:1).
^{1}H NMR (400 MHz, Acetone-d_6) δ 8.65 (dd, J = 2.7, 1.6 Hz, 1H), 8.10 (dq, J = 7.8, 0.9 Hz, 1H), 7.71 (ddt, J = 8.0, 7.0, 1.0 Hz, 1H), 7.46 – 7.29 (m, 4H), 7.23 (dd, J = 7.4, 5.4 Hz, 1H), 7.19 – 7.06 (m, 3H), 5.32 (s, 2H), 4.31 (dq, J = 8.9, 7.2 Hz, 1H), 3.53 (s, 3H), 1.57 (dd, J = 7.2, 1.9 Hz, 3H).
^{13}C NMR (101 MHz, Acetone-d_6) δ 176.03, 163.36 (d, J = 244.9 Hz), 142.15, 134.80 (d, J = 3.4 Hz), 130.50 (d, J = 8.4 Hz, 2C), 130.30, 128.97, 127.80, 127.68, 125.81, 121.32, 120.57, 118.95, 118.19, 116.45 (d, J = 21.9 Hz, 2C), 109.34, 109.11, 59.72, 51.62, 37.59, 17.35.
^{19}F NMR (376 MHz, Acetone-d_6) δ -115.75.
HRMS: Calculated for: $C_{23}H_{20}O_2NFNa$ $[M+Na]^+$ 384.1370, found 384.1365.
IR: 1724 1639 1597 1510 1225 1169 1135.

2-(2-(4-Fluorobenzyl)-2*H*-indeno[1,2-c]pyridin-5-yl)-*N*-methoxy-*N*-methylacetamide (6p)

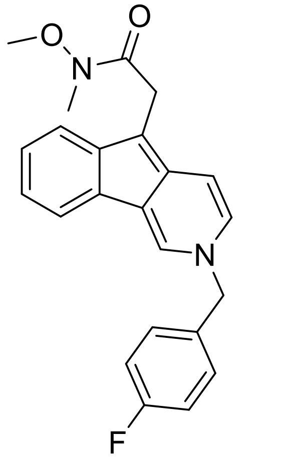

Prepared according to general procedure D using **5p** (46 mg, 0.10 mmol) to yield the titled compound as an orange oil (23 mg, 60%).

^{1}H NMR (400 MHz, Acetone-*d*$_6$) δ 8.61 (d, *J* = 1.5 Hz, 1H), 8.08 (dt, *J* = 7.8, 1.0 Hz, 1H), 7.74 (dt, *J* = 7.9, 0.9 Hz, 1H), 7.44 – 7.28 (m, 4H), 7.22 (d, *J* = 7.3 Hz, 1H), 7.18 – 7.05 (m, 3H), 5.30 (s, 2H), 4.02 (s, 2H), 3.54 (s, 3H), 3.08 (s, 3H).

^{13}C NMR (101 MHz, Acetone-*d*$_6$) δ 173.48, 163.32 (d, *J* = 244.6 Hz), 143.52, 134.93 (d, *J* = 3.1 Hz), 130.43 (d, *J* = 8.4 Hz, 2C), 130.22, 130.04, 127.67, 127.54, 125.71, 121.31, 120.37, 118.87, 118.44, 116.41 (d, *J* = 21.7 Hz, 2C), 109.59, 103.54, 61.40, 59.65, 32.43, 30.72.

^{19}F NMR (376 MHz, Acetone-*d*$_6$) δ -115.87.

HRMS: Calculated for: $C_{23}H_{21}O_2N_2F$ $[M]^+$ 376.1582, found 376.1600.

IR: 1639 1598 1509 1385 1224 1132.

2-(2-(4-Fluorobenzyl)-2*H*-indeno[1,2-c]pyridin-5-yl)acetonitrile (6q)

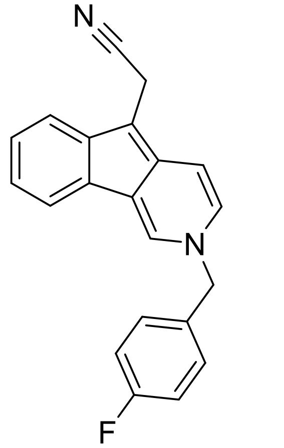

Prepared according to general procedure D using **5q** (40 mg, 0.10 mmol) to yield the titled compound as a yellow oil (29 mg, 92%).

^{1}H NMR (400 MHz, Acetone-*d*$_6$) δ 8.64 (d, *J* = 1.6 Hz, 1H), 8.00 (dt, *J* = 7.8, 1.0 Hz, 1H), 7.56 (dt, *J* = 7.9, 0.9 Hz, 1H), 7.36 (dd, *J* = 7.3, 1.5 Hz, 1H), 7.33 – 7.25 (m, 3H), 7.18 (d, *J* = 7.3 Hz, 1H), 7.07 – 6.94 (m, 3H), 5.28 (s, 2H), 3.99 (s, 2H).

^{13}C NMR (101 MHz, Acetone-*d*$_6$) δ 162.52 (d, *J* = 244.9 Hz), 141.11, 133.81 (d, *J* = 3.4 Hz), 130.06, 129.66, 129.62 (d, *J* = 8.2 Hz, 2C), 127.63, 126.70, 125.28, 120.30, 119.88, 118.63, 118.58, 116.46, 115.62 (d, *J* = 21.7 Hz, 2C), 107.73, 96.57, 59.07, 12.72.

^{19}F NMR (376 MHz, Acetone-*d*$_6$) δ -115.70.

HRMS: Calculated for: $C_{21}H_{15}N_2FNa$ $[M+Na]^+$ 337.1111, found 337.1108.

IR: 1639 1600 1510 1225 1133.

Methyl 2-(2-(4-fluorobenzyl)-4-methoxy-2*H*-indeno[1,2-c]pyridin-5-yl)acetate (6r)

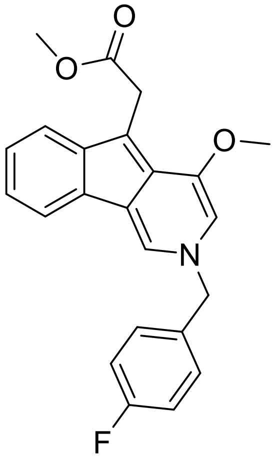

Prepared according to general procedure D using **5r** (46 mg, 0.10 mmol) to yield the titled compound as a yellow solid (31 mg, 83%).

^{1}H NMR (400 MHz, Acetone-d_6) δ 8.56 (d, *J* = 1.3 Hz, 1H), 8.06 (dt, *J* = 7.8, 1.0 Hz, 1H), 7.59 (dt, *J* = 8.0, 0.9 Hz, 1H), 7.46 – 7.36 (m, 2H), 7.31 (ddd, *J* = 8.0, 7.0, 1.1 Hz, 1H), 7.18 – 7.06 (m, 3H), 7.04 (d, *J* = 1.3 Hz, 1H), 5.31 (s, 2H), 4.22 (s, 2H), 3.87 (s, 3H), 3.58 (s, 3H).

^{13}C NMR (101 MHz, Acetone-d_6) δ 173.64, 163.33 (d, *J* = 244.6 Hz), 148.95, 142.43, 134.82 (d, *J* = 3.1 Hz), 130.39 (d, *J* = 8.3 Hz, 2C), 128.18 (2C), 125.09, 124.14, 120.56, 120.17, 119.18, 118.21, 116.38 (d, *J* = 21.6 Hz, 2C), 108.81, 103.07, 59.99, 56.02, 51.52, 32.38.

^{19}F NMR (376 MHz, Acetone-d_6) δ -115.91.

HRMS: Calculated for: $C_{23}H_{21}O_3NF$ [M+H]$^+$ 378.1500, found 378.1485.

IR: 1707 1260 1217 1129 1008 741.

m.p. 151-152 °C.

Methyl 2-(4-(benzyloxy)-2-(4-fluorobenzyl)-2*H*-indeno[1,2-c]pyridin-5-yl)acetate (6s)

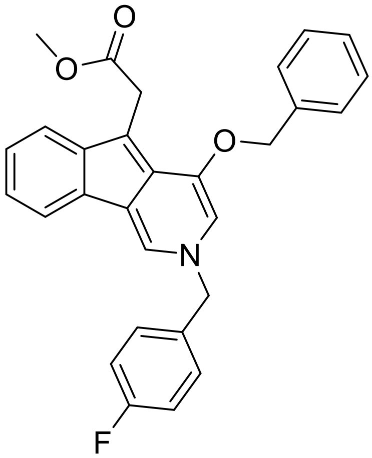

Prepared according to general procedure D using **5s** (52 mg, 0.10 mmol) to yield the titled compound as a yellow oil (38 mg, 83%).

^{1}H NMR (400 MHz, Acetone-d_6) δ 8.53 (t, *J* = 1.6 Hz, 1H), 8.07 (dt, *J* = 7.7, 1.0 Hz, 1H), 7.67 – 7.58 (m, 1H), 7.56 – 7.51 (m, 2H), 7.42 – 7.37 (m, 2H), 7.37 – 7.30 (m, 4H), 7.13 – 7.06 (m, 4H), 5.23 (t, *J* = 2.9 Hz, 2H), 5.16 (s, 2H), 4.27 (s, 2H), 3.55 (s, 3H).

^{13}C NMR (101 MHz, Acetone-d_6) δ 173.63, 163.29 (d, *J* = 244.5 Hz), 147.54, 142.52, 137.99, 134.68 (d, *J* = 3.2 Hz), 130.38 (d, *J* = 8.3 Hz, 2C), 129.30 (2C), 128.66, 128.36 (2C), 128.13 (2C), 125.18, 124.31,

120.82, 120.21, 119.23, 118.22, 116.36 (d, J = 21.9 Hz, 2C), 110.23, 103.04, 70.89, 59.99, 51.50, 32.37.
^{19}F NMR (376 MHz, Acetone-d_6) δ -115.85.
HRMS: Calculated for: $C_{29}H_{25}O_3NF$ $[M+H]^+$ 454.1813, found 454.1808.
IR: 1731 1630 1596 1510 1266 1224 1168 1133.

Methyl 2-(4-fluoro-2-(4-fluorobenzyl)-2*H*-indeno[1,2-c]pyridin-5-yl)acetate (6t)

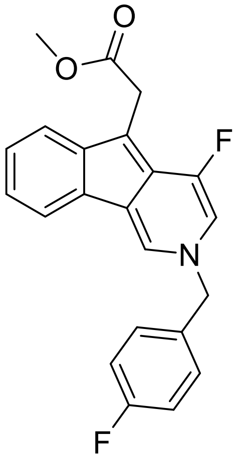

To a flask was added pre-NHC **A** (5.5 mg, 0.02 mmol, 0.2 eq.) and evacuated and backfilled 3 times with nitrogen. To this flask was added degassed DMF (4 mL) and DBU (30 µL, 0.20 mmol, 2.0 eq.) and the solution was stirred for 15 min.

To a microwave vial was added **5t** (0.10 mmol, 1.0 eq.) and sealed. The vial was evacuated and backfilled 3 times with nitrogen. To this vial was added the solution of NHC and DBU in DMF. The reaction mixture was stirred at 85 °C for 30 min before being quenched by addition of 1 M HCl (4 mL) and diethyl ether (20 mL) and extracted 3 times with 1 M HCl. The combined aqueous extracts were then basified with 2 M NaOH (20 mL) and extracted 3 times with diethyl ether. The combined organic extracts were washed 3 times with 5% LiCl solution, brine, dried over Na_2SO_4 and concentrated under reduced pressure. The resulting crude mixture was purified by column chromatography on an automatic column machine (hexanes:ethyl acetate:NEt_3) to yield the product as a yellow oil (6 mg, 18%).

^{1}H NMR (500 MHz, Acetone-d_6) δ 8.75 (t, J = 1.7 Hz, 1H), 8.12 (d, J = 7.8 Hz, 1H), 7.68 (d, J = 8.0 Hz, 1H), 7.55 – 7.45 (m, 3H), 7.43 – 7.36 (m, 1H), 7.16 (td, J = 7.8, 6.8, 1.9 Hz, 3H), 5.43 (s, 2H), 4.08 (s, 2H), 3.59 (s, 3H).
^{13}C NMR (126 MHz, Acetone-d_6) δ 172.89, 163.50 (d, J = 245.0 Hz), 152.37 (d, J = 242.2 Hz), 142.79, 134.36 (d, J = 3.1 Hz), 130.74 (d, J = 8.4 Hz, 2C), 129.18, 128.34, 126.12, 123.26 (d, J = 15.5 Hz), 121.52 (d, J = 16.0 Hz), 120.63, 119.99, 118.57, 116.55 (d, J = 21.7 Hz, 2C), 113.79 (d, J = 39.0 Hz), 101.92 (d, J = 3.9 Hz), 59.78, 51.73, 32.23 (d, J = 2.7 Hz).
^{19}F NMR (471 MHz, Acetone-d_6) δ -115.49, -144.76.
HRMS: Calculated for: $C_{22}H_{17}O_2NF_2Na$ $[M+Na]^+$ 388.1120, found 388.1114.
IR: 1732 1641 1599 1510 1256 1225 1193 1165 1129.

Methyl 2-(2-(4-fluorobenzyl)-3-methyl-2*H*-indeno[1,2-c]pyridin-5-yl)acetate (6u)

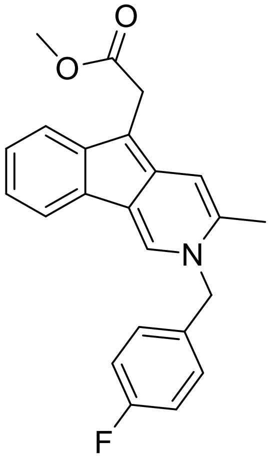

Prepared according to general procedure D using **5u** (44 mg, 0.10 mmol) to yield the titled compound as an orange oil (20 mg, 56%).

^{1}H NMR (400 MHz, Acetone-d_6) δ 8.63 – 8.57 (m, 1H), 8.05 (dt, *J* = 7.7, 1.0 Hz, 1H), 7.61 (dt, *J* = 8.0, 1.0 Hz, 1H), 7.35 (ddd, *J* = 8.1, 7.0, 1.1 Hz, 1H), 7.19 – 7.10 (m, 4H), 7.07 (ddd, *J* = 7.9, 7.1, 1.1 Hz, 1H), 7.02 (d, *J* = 1.0 Hz, 1H), 5.43 – 5.40 (m, 2H), 3.89 (s, 2H), 3.58 (s, 3H), 2.42 (s, 3H).

^{13}C NMR (101 MHz, Acetone-d_6) δ 172.96, 163.03 (d, *J* = 244.2 Hz), 143.68, 135.11, 135.08, 132.05, 131.97, 129.01 (d, *J* = 8.2 Hz, 2C), 127.80, 125.90, 121.02, 120.46, 118.79, 117.74, 116.50 (d, *J* = 21.8 Hz, 2C), 109.06, 101.56, 56.52, 51.65, 31.60, 19.97.

^{19}F NMR (376 MHz, Acetone-d_6) δ -116.51.

HRMS: Calculated for: $C_{23}H_{21}O_2NF$ $[M+H]^+$ 362.1551, found 362.1543.

IR: 1729 1645 1598 1509 1331 1224 1157 1132.

m.p. 131-132 °C.

Methyl 2-(2-(4-((triisopropylsilyl)oxy)benzyl)-2*H*-indeno[1,2-c]pyridin-5-yl)acetate (6v)

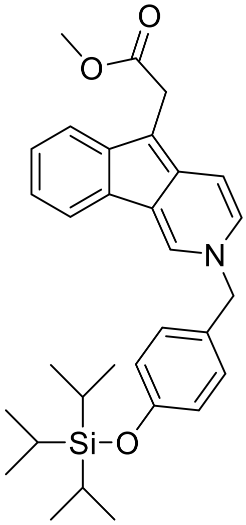

Prepared according to general procedure D using **5v** (58 mg, 0.10 mmol) to yield the titled compound as an orange oil (19 mg, 38%).

^{1}H NMR (500 MHz, Acetone-d_6) δ 8.66 (d, *J* = 1.5 Hz, 1H), 8.09 (dt, *J* = 7.8, 1.0 Hz, 1H), 7.63 (dt, *J* = 7.9, 1.0 Hz, 1H), 7.40 – 7.32 (m, 2H), 7.32 – 7.25 (m, 2H), 7.18 (d, *J* = 7.2 Hz, 1H), 7.09 (ddd, *J* = 7.9, 7.0, 1.0 Hz, 1H), 6.96 – 6.89 (m, 2H), 5.30 (s, 2H), 3.91 (s, 2H), 3.56 (s, 3H), 1.34 – 1.19 (m, 3H), 1.09 (d, *J* = 7.4 Hz, 18H).

^{13}C NMR (126 MHz, Acetone-d_6) δ 172.89, 156.85, 143.13, 131.24, 130.58, 130.35, 129.89, 127.89 (2C), 127.62, 125.76, 121.13 (2C), 120.96, 120.46, 118.88, 117.89, 109.04, 102.28, 60.14, 51.62, 31.62, 18.24 (6C), 13.35 (3C).
HRMS: Calculated for: $C_{31}H_{40}O_3NSi$ $[M+H]^+$ 502.2772, found 502.2760.
IR: 2945 2867 1733 1640 1600 1510 1265 1238 1164 1129 992 884 684.

Preparation of other compounds

2-(4-Fluorobenzyl)-4-methoxy-5-(2-methoxy-2-oxoethyl)-5-methyl-5*H*-indeno[1,2-c]pyridin-2-ium iodide (12a)

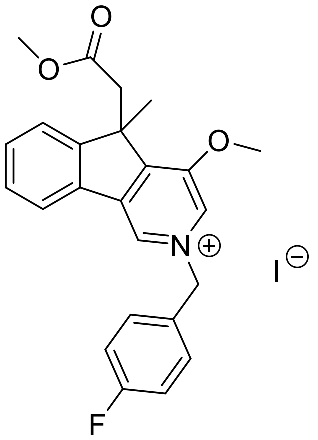

To a flask charged with **6r** (189 mg, 0.50 mmol) and MeCN (10 mL) was added methyl iodide (156 µL, 1.25 mmol) and allowed to stir for 16 h at 85 °C. The solvent was removed under reduced pressure and the resulting crude mixture was purified by column chromatography on an automated purification machine (hexanes:DCM:MeOH) to yield the product as a brown solid (259 mg, quant.).
^{1}H NMR (400 MHz, Acetone-d_6) δ 10.11 (s, 1H), 9.60 (s, 1H), 8.26 (d, *J* = 7.5 Hz, 1H), 8.13 (dd, *J* = 8.7, 5.4 Hz, 2H), 7.73 (d, *J* = 7.6 Hz, 1H), 7.56 (t, *J* = 7.5 Hz, 1H), 7.49 (t, *J* = 7.5 Hz, 1H), 7.20 (t, *J* = 8.8 Hz, 2H), 6.36 (d, *J* = 3.1 Hz, 2H), 4.33 (s, 3H), 3.54 (d, *J* = 16.1 Hz, 1H), 3.37 (d, *J* = 16.1 Hz, 1H), 3.22 (s, 3H), 1.61 (s, 3H).
^{13}C NMR (101 MHz, Acetone-d_6) δ 170.44, 164.13 (d, *J* = 247.1 Hz), 155.58, 154.93, 152.15, 142.16, 135.27, 132.85 (d, *J* = 8.7 Hz, 2C), 132.27, 131.72 (d, *J* = 3.1 Hz), 131.58, 129.24, 128.90, 123.91, 123.86, 116.71 (d, *J* = 21.8 Hz, 2C), 63.01, 59.75, 51.71, 51.57, 40.78, 23.47.
^{19}F NMR (376 MHz, Acetone-d_6) δ -113.32.
HRMS: Calculated for: $C_{24}H_{23}O_3NF$ $[M]^+$ 392.1656, found 392.1649.
IR: 1733 1587 1509 1458 1436 1341 1290 1225 1200 1156 1044.
m.p. 102-104 °C.

5-Benzyl-2-(4-fluorobenzyl)-4-methoxy-5-(2-methoxy-2-oxoethyl)-5*H*-indeno[1,2-c]pyridin-2-ium bromide (12b)

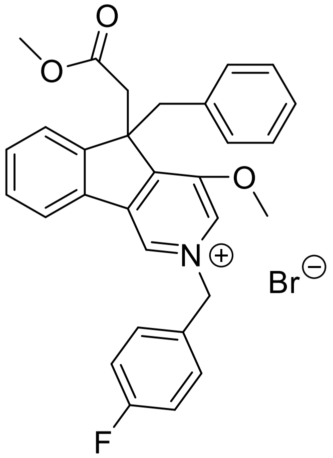

To a flask charged with **6r** (113 mg, 0.30 mmol) and MeCN (5 mL) was added benzyl bromide (54 µL, 0.45 mmol) and allowed to stir for 16 h at 85 °C. The solvent was removed under reduced pressure and the resulting crude mixture was purified by column chromatography on an automated purification machine (hexanes:DCM:MeOH) to yield the product as a white solid (158 mg, 96%).

^{1}H NMR (400 MHz, Methanol-d_4) δ 9.01 (s, 1H), 8.83 (s, 1H), 7.80 (dd, *J* = 13.0, 7.7 Hz, 2H), 7.64 (t, *J* = 7.6 Hz, 1H), 7.53 – 7.41 (m, 3H), 7.20 (t, *J* = 8.7 Hz, 2H), 6.87 (t, *J* = 7.4 Hz, 1H), 6.74 (t, *J* = 7.7 Hz, 2H), 6.35 (d, *J* = 6.9 Hz, 2H), 5.82 (d, *J* = 2.8 Hz, 2H), 4.30 (s, 3H), 3.79 (d, *J* = 15.9 Hz, 1H), 3.60 (d, *J* = 13.0 Hz, 1H), 3.53 (d, *J* = 15.9 Hz, 1H), 3.48 (d, *J* = 13.0 Hz, 1H), 3.25 (s, 3H).

^{13}C NMR (101 MHz, Methanol-d_4) δ 171.47, 164.76 (d, *J* = 248.0 Hz), 156.46, 154.20, 149.96, 143.81, 136.73, 135.86, 131.99, 131.70, 131.66 (d, *J* = 3.4 Hz), 131.54 (d, *J* = 8.7 Hz, 2C), 130.03 (2C), 129.98, 128.43 (2C), 128.13, 127.79, 125.22, 123.11, 117.33 (d, *J* = 22.1 Hz, 2C), 65.00, 58.74, 57.88, 51.96, 43.55, 40.90.

^{19}F NMR (376 MHz, Methanol-d_4) δ -113.64.

HRMS: Calculated for: $C_{30}H_{27}O_3NF$ $[M]^+$ 468.1969, found 468.1965.

IR: 1733 1590 1510 1437 1344 1290 1224 1158 1140 1047.

m.p. 116-117 °C.

2-(4-Fluorobenzyl)-4-methoxy-5-(2-methoxy-2-oxoethyl)-5-(2-oxo-2-phenylethyl)-5*H*-indeno[1,2-c]pyridin-2-ium bromide (12c)

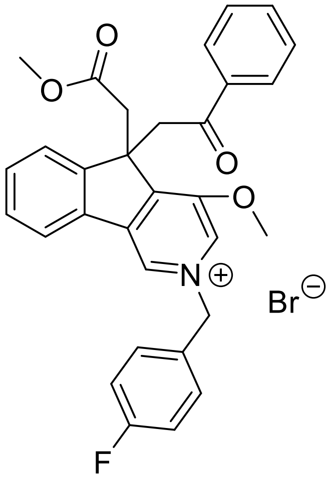

To a flask charged with **6r** (113 mg, 0.30 mmol) and MeCN (5 mL) was added phenacyl bromide (90 mg, 0.45 mmol) and allowed to stir for 16 h at 85 °C. The solvent was removed under reduced pressure and the resulting crude mixture was purified by column chromatography on an automated purification machine (hexanes:DCM:MeOH) to yield the product as a white solid (169 mg, 98%).

^{1}H NMR (400 MHz, Methanol-d_4) δ 9.33 (s, 1H), 8.80 (s, 1H), 8.06 – 7.96 (m, 1H), 7.70 (dd, *J* = 14.0, 8.7 Hz, 5H), 7.51 (t, *J* = 7.5 Hz, 1H), 7.49 – 7.44 (m, 2H), 7.40 – 7.32 (m, 2H), 7.24 (t, *J* = 8.7 Hz, 2H), 5.91 (s, 2H), 4.37 (d, *J* = 17.0 Hz, 1H), 4.14 (d, *J* = 17.0 Hz, 4H), 4.13 (s, 4H), 3.48 (d, *J* = 15.1 Hz, 1H), 3.42 (d, *J* = 15.1 Hz, 1H), 3.23 (s, 3H).
^{13}C NMR (101 MHz, Methanol-d_4) δ 197.76, 170.91, 164.87 (d, *J* = 248.0 Hz), 155.92, 154.98, 149.83, 144.28, 137.68, 136.80, 134.66, 132.09 (d, *J* = 8.6 Hz, 2C), 131.92, 131.71, 131.50 (d, *J* = 3.4 Hz), 129.91, 129.68 (2C), 129.01 (2C), 128.19, 124.60, 123.31, 117.42 (d, *J* = 22.1 Hz, 2C), 65.09, 58.63, 53.70, 51.96, 45.14, 41.54.
^{19}F NMR (376 MHz, Methanol-d_4) δ -113.43.
HRMS: Calculated for: $C_{31}H_{27}O_4NF$ $[M]^+$ 496.1919, found 496.1908.
IR: 1732 1671 1593 1510 1467 1437 1343 1287 1226 1209 1176 1162 1155 1141 1108 1049 1000 843 751 692.
m.p. 175-176 °C.

5-(2-Methoxy-2-oxoethyl)-5-methyl-2-(4-((triisopropylsilyl)oxy)benzyl)-5*H*-indeno[1,2-c]pyridin-2-ium iodide (12d)

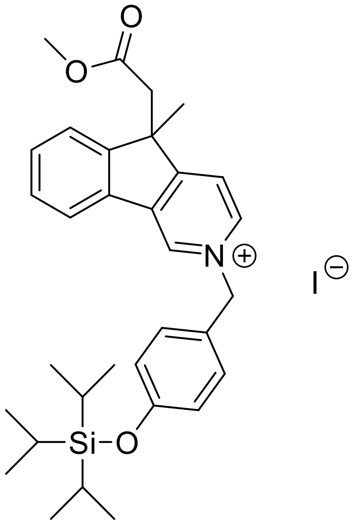

To a flask charged with **6v** (15 mg, 0.030 mmol) and MeCN (1 mL) was added methyl iodide (5.6 µL, 0.09 mmol) and allowed to stir for 16 h at 85 °C. The solvent was removed under reduced pressure and the resulting crude mixture was purified by column chromatography on an automated purification machine (hexanes:DCM:MeOH) to yield the product as a yellow solid (8.2 mg, 43%).
^{1}H NMR (400 MHz, Acetone-d_6) δ 10.81 – 10.59 (m, 1H), 9.45 (dd, *J* = 6.3, 1.4 Hz, 1H), 8.53 (s, 1H), 8.36 (dt, *J* = 7.5, 1.0 Hz, 1H), 7.93 – 7.85 (m, 2H), 7.75 (dt, *J* = 7.6, 0.9 Hz, 1H), 7.57 (td, *J* = 7.5, 1.3 Hz, 1H), 7.50 (td, *J* = 7.5, 1.2 Hz, 1H), 7.01 – 6.93 (m, 2H), 6.21 (s, 2H), 3.56 (d, *J* = 16.4 Hz, 1H), 3.35 (d, *J* = 16.5 Hz, 1H), 3.28 (s, 3H), 1.64 (s, 3H), 1.37 – 1.22 (m, 3H), 1.09 (d, *J* = 7.4 Hz, 18H).
^{13}C NMR (101 MHz, Acetone-d_6) δ 170.48, 170.22, 158.05, 152.07, 143.64, 141.82, 138.28, 135.06, 132.16 (2C), 131.78, 129.35, 128.10, 124.23, 124.09, 123.50, 121.25 (2C), 63.77, 51.65, 51.28, 42.44, 25.63, 18.22 (6C), 13.33 (3C).
HRMS: Calculated for: $C_{32}H_{42}O_3NSi$ $[M]^+$ 516.2928, found 516.2909.
IR: 2944 2866 1736 1607 1512 1459 1270 1232 1205 1175 910 883 684.
m.p. 90-92°C.

2-(2-(4-Fluorobenzyl)-4-methoxy-2*H*-indeno[1,2-c]pyridin-5-yl)ethan-1-ol (13)

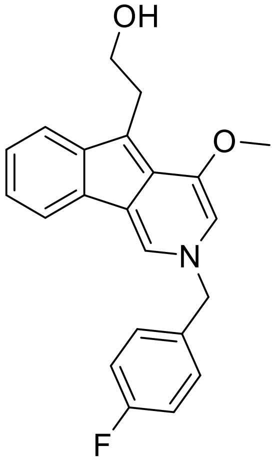

To a flask was added **6r** (113 mg, 0.30 mmol), THF (10 mL) and cooled to 0 °C. To this mixture was added 1M DIBAL-H solution in THF (750 µL, 0.75 mmol) and the solution was allowed to stir for 16 h at room temperature. A further 1M DIBAL-H solution in THF (300 µL, 0.30 mmol) was added and the reaction mixture stirred for an additional 3 h until completion by TLC. Methanol (10 mL) was added and the reaction mixture allowed to stir for 15 min. Saturated $NaHCO_3$ solution (10 mL) was added and the mixture was extracted 5 times with ethyl acetate. The combined organic extracts were washed with water, brine, dried over Na_2SO_4 and concentrated under reduced pressure to yield the title compound as a yellow solid (103 mg, 98%).
^{1}H NMR (400 MHz, Acetone-d_6) δ 8.52 (s, 1H), 8.05 (d, *J* = 7.8 Hz, 1H), 7.67 (d, *J* = 8.0 Hz, 1H), 7.41 (dd, *J* = 8.3, 5.4 Hz, 2H), 7.31 (t, *J* = 7.6 Hz, 1H), 7.13 (t, *J* = 8.8 Hz, 2H), 7.08 (t, *J* = 7.4 Hz, 2H), 6.99 (s, 1H), 5.33 (s, 2H), 3.89 (s, 3H), 3.77 (t, *J* = 7.6 Hz, 2H), 3.45 – 3.39 (m, 2H), 3.38 (s, 0H).
^{13}C NMR (101 MHz, Acetone-d_6) δ 163.28 (d, *J* = 244.6 Hz), 149.11, 142.79, 134.92 (d, *J* = 3.3 Hz), 130.32 (d, *J* = 8.3 Hz), 128.31, 127.79, 124.92, 123.40, 120.66, 120.13, 118.99, 118.08, 116.34 (d, *J* = 21.7 Hz), 108.15, 107.63, 64.24, 59.85, 55.91, 30.99.
^{19}F NMR (376 MHz, Acetone-d_6) δ -115.97.
HRMS: Calculated for: $C_{22}H_{21}O_2NF$ $[M+H]^+$ 350.1551, found 350.1536.
IR: 1627 1593 1508 1397 1329 1262 1222 1143 1129 1041 1012 759.
m.p. 70-72 °C.

Methyl 2-(5-(4-hydroxybenzyl)-5*H*-indeno[1,2-c]pyridin-5-yl)acetate (14)

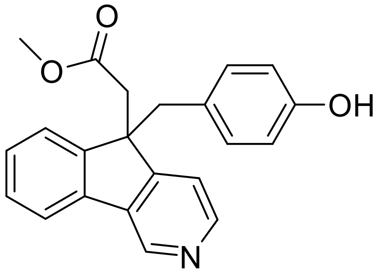

A flask containing pre-NHC **A** (5.5 mg, 0.02 mmol) was evacuated and backfilled 3 times with nitrogen. To this flask was added degassed methanol (4 mL) and DBU (30 µL, 0.20 mmol) and the solution was stirred for 15 min.
A sealed microwave vial containing **5v** (58 mg, 0.10 mmol) was evacuated and backfilled 3 times with nitrogen. To this vial was added the solution of NHC and DBU in methanol. The reaction mixture was stirred at 85 °C for 30 min before being cooled to room temperature. To the mixture was added cesium fluoride (30 mg, 0.20 mmol) and allowed to stir for 16 h at room temperature. The mixture was poured into saturated ammonium chloride solution and extracted 3 times with ethyl acetate.

The combined organic extracts were washed with brine, dried over Na_2SO_4 and concentrated under reduced pressure. The resulting crude mixture was purified by column chromatography on an automatic column machine (hexanes:ethyl acetate) to yield the product as a white solid (19 mg, 56%).

^{1}H NMR (400 MHz, Chloroform-*d*) δ 8.77 (d, *J* = 1.0 Hz, 1H), 8.47 (d, *J* = 5.2 Hz, 1H), 7.62 (dd, *J* = 6.7, 1.3 Hz, 1H), 7.50 – 7.44 (m, 1H), 7.44 – 7.31 (m, 3H), 6.44 – 6.34 (m, 4H), 3.39 (s, 4H), 3.27 (d, *J* = 13.4 Hz, 1H), 3.15 (d, *J* = 13.5 Hz, 1H), 3.14 (d, *J* = 15.2 Hz, 1H), 3.09 (d, *J* = 15.3 Hz, 1H).

^{13}C NMR (101 MHz, Chloroform-*d*) δ 170.47, 158.67, 155.70, 148.04, 145.93, 140.49, 137.64, 137.52, 131.29 (2C), 128.52, 128.20, 126.30, 123.94, 120.92, 120.20, 114.59 (2C), 54.29, 51.63, 44.41, 41.65.

HRMS: Calculated for: $C_{22}H_{20}O_3N$ $[M+H]^+$ 346.1438, found 346.1427.

IR: 1735 1610 1514 1458 1246 1220 1200 1167 832 750 729.

m.p. 59-61 °C.

Methyl 2-(5-methyl-5*H*-indeno[1,2-c]pyridin-5-yl)acetate (15)

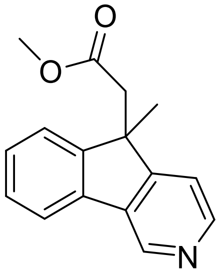

A flask containing pre-NHC **G** (12.6 mg, 0.04 mmol) was evacuated and backfilled 3 times with nitrogen. To this flask was added degassed methanol (8 mL) and DBU (60 µL, 0.20 mmol) and the solution was stirred for 15 min.

A sealed microwave vial containing **5v** (116 mg, 0.20 mmol) was evacuated and backfilled 3 times with nitrogen. To this vial was added the solution of NHC and DBU in methanol. The reaction mixture was stirred at 85 °C for 30 min before being quenched by addition of 1 M HCl (8 mL). The mixture was then basified with 2 M NaOH (20 mL) and extracted 3 times with diethyl ether. The combined organic extracts were washed with brine, dried over Na_2SO_4 and concentrated under reduced pressure.

The resulting crude mixture was transferred to a sealed microwave vial. To this vial was added MeCN (2 mL) and methyl iodide (38 µL, 0.60 mmol) and the reaction mixture was stirred at 85 °C for 16 h before being cooled to room temperature. To the mixture was added cesium fluoride (61 mg, 0.40 mmol) and methanol (2 mL) and the reaction mixture was stirred for a further 3 h at room temperature. The mixture was poured into saturated ammonium chloride solution and extracted 3 times with ethyl acetate. The combined organic extracts were washed with brine, dried over Na_2SO_4 and concentrated under reduced pressure. The resulting crude mixture was purified by column chromatography on an automatic column machine (hexanes:ethyl acetate) to yield the product as a yellow oil (17 mg, 33%).

^{1}H NMR (500 MHz, Chloroform-*d*) δ 9.01 (s, 1H), 8.56 (d, *J* = 5.0 Hz, 1H), 7.83 – 7.78 (m, 1H), 7.49 – 7.43 (m, 2H), 7.39 (pd, *J* = 7.3, 1.5 Hz, 2H), 3.44 (s, 3H), 2.93 (d, *J* = 15.0 Hz, 1H), 2.86 (d, *J* = 15.0 Hz, 1H), 1.58 (s, 3H).

^{13}C NMR (126 MHz, Chloroform-*d*) δ 170.63, 159.49, 150.35, 148.05, 142.03, 137.11, 136.02, 128.52, 128.09, 123.23, 120.81, 118.77, 51.52, 48.96, 43.36, 25.26.

HRMS: Calculated for: $C_{16}H_{16}O_2N$ $[M+H]^+$ 254.1176, found 254.1164.

IR: 1735 1446 1437 1413 1252 1217 1196 1173 1156 749.

Methyl 2-(5-methyl-5*H*-indeno[1,2-c]pyridin-5-yl)acetate (15)

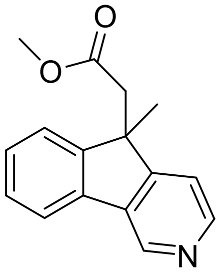

To a microwave vial containing **12d** (8.2 mg, 0.013 mmol) in methanol (0.5 mL) was added cesium fluoride (2.0 mg, 0.026 mmol) and allowed to stir for 3 h at room temperature. The mixture was poured into saturated ammonium chloride solution and extracted 3 times with ethyl acetate. The combined organic extracts were washed with brine, dried over Na_2SO_4 and concentrated under reduced pressure. The resulting crude mixture was purified by column chromatography on an automatic column machine (hexanes:ethyl acetate) to yield the product as a yellow oil (2.2 mg, 67%).

2-(4-Fluorobenzyl)-5-(2-methoxy-2-oxoethyl)-5-methyl-5*H*-indeno[1,2-c]pyridin-2-ium tetrafluoroborate (18)

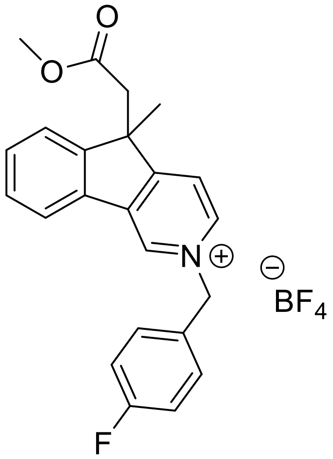

A flask containing pre-NHC **A** (27 mg, 0.10 mmol) was evacuated and backfilled 3 times with nitrogen. To this flask was added degassed THF (1 mL) and DBU (15 µL, 0.10 mmol, 1.0 eq.) and the solution was stirred for 15 min.

A microwave vial containing **5w** (36 mg, 0.10 mmol, 1.0 eq.) was evacuated and backfilled 3 times with nitrogen. To this vial was added degassed THF (3 mL) followed by the solution of NHC and DBU in THF. The reaction mixture was stirred at 70 °C for 30 min before being cooled to room temperature and diluted with diethyl ether. The mixture was then filtered through a sinter glass filter and washed with diethyl ether. The residue was redissolved in methanol and concentrated under reduced pressure. The resulting crude mixture was purified by column chromatography on an automatic column machine (DCM:MeOH) to yield the product as a white solid (19 mg, 43%)
^{1}H NMR (400 MHz, Acetone-d_6) δ 9.76 (dd, *J* = 1.3, 0.7 Hz, 1H), 9.17 (dd, *J* = 6.3, 1.4 Hz, 1H), 8.51 (dd, *J* = 6.3, 0.8 Hz, 1H), 8.17 – 8.10 (m, 1H), 7.85 – 7.74 (m, 3H), 7.57 (dtd, *J* = 23.5, 7.4, 1.2 Hz, 2H), 7.31 – 7.21 (m, 2H), 6.10 (s, 2H), 3.54 (d, *J* = 16.6 Hz, 1H), 3.38 (d, *J* = 16.6 Hz, 1H), 3.30 (s, 3H), 1.65 (s, 3H).
^{13}C NMR (101 MHz, Acetone-d_6) δ 170.85, 170.49, 164.27 (d, *J* = 247.1 Hz), 152.17, 143.88, 142.18, 137.40, 134.76, 132.55 (d, *J* = 8.8 Hz, 2C), 132.05, 131.20 (d, *J* = 3.1 Hz, 2C), 129.50, 124.35, 123.71, 123.46, 117.12 (d, *J* = 22.0 Hz), 64.61, 51.68, 51.28, 42.36, 25.62.

^{19}**F NMR** (471 MHz, Acetone-d_6) δ -113.11, -151.63, -151.69.
HRMS: Calculated for: $C_{23}H_{21}O_2NF$ $[M]^+$ 362.1551, found 362.1539.
IR: 1736 1513 1499 1459 1230 1066.
m.p. 63-65 °C.

1-(4-Fluorobenzyl)-3-phenylpyridin-1-ium bromide (19)

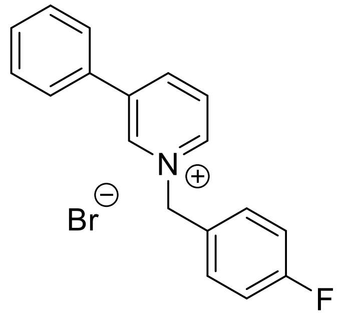

Prepared according to general procedure C2 using **24** (253 mg, 1.62 mmol) and 4-fluorobenzyl bromide (300 µL, 2.45 mmol) in MeCN (4 mL) at 80 °C to yield the titled compound as a white solid (387 mg, 69%).
^{1}**H NMR** (400 MHz, Methanol-d_4) δ 9.54 (t, *J* = 1.8 Hz, 1H), 9.03 (dt, *J* = 6.1, 1.3 Hz, 1H), 8.87 (ddd, *J* = 8.2, 2.0, 1.2 Hz, 1H), 8.18 (dd, *J* = 8.2, 6.1 Hz, 1H), 7.91 – 7.83 (m, 2H), 7.76 – 7.66 (m, 2H), 7.64 – 7.51 (m, 3H), 7.26 – 7.16 (m, 2H), 6.00 (s, 2H).
^{13}**C NMR** (101 MHz, Methanol-d_4) δ 164.91 (d, *J* = 248.5 Hz), 144.77, 143.91, 143.76, 142.99, 134.51, 132.78 (d, *J* = 8.7 Hz, 2C), 131.56, 130.84, 130.80 (2C), 129.76, 128.72 (2C), 117.48 (d, *J* = 22.1 Hz, 2C), 64.88.
^{19}**F NMR** (376 MHz, Methanol-d_4) δ -113.00.
HRMS: Calculated for: $C_{18}H_{15}NF$ $[M]^+$ 264.1183, found 264.1172.
IR: 1601 1508 1487 1218 1153 835 817 754 688.
m.p. 135-136 °C.

3-(Benzyloxy)-5-bromopyridine (23)

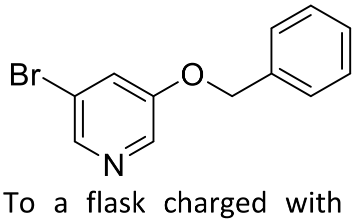

To a flask charged with NaH (420 mg, 10.5 mmol, 1.05 eq.) in DMF (10 mL) was added 5-bromopyridin-3-ol (1.47 g, 10.0 mmol, 1.10 eq.) slowly at 0 °C and allowed to stir for 15 min. Benzyl bromide (1.32 mL, 11.0 mmol, 1.10 eq.) was added and the mixture was allowed to stir for 2 h. The reaction mixture was poured into ice water and extracted 3 times with ethyl acetate. The combined organic extracts were washed 5 times with 5% LiCl solution, brine, dried over Na_2SO_4 and concentrated under reduced pressure. The resulting crude mixture was purified by column chromatography on an automated purification machine (hexanes:ethyl acetate) to yield the product as a red solid (840 mg, 32%).
^{1}**H NMR** (400 MHz, Chloroform-*d*) δ 8.30 (dd, *J* = 3.8, 2.2 Hz, 2H), 7.44 – 7.32 (m, 6H), 5.06 (s, 2H).
^{13}**C NMR** (101 MHz, Chloroform-*d*) δ 155.27, 143.27, 136.81, 135.56, 128.88 (2C), 128.61, 127.65 (2C), 124.42, 120.47, 70.71.
HRMS: Calculated for: $C_{12}H_{11}ONBr$ $[M+H]^+$ 264.0019, found 264.0018.
IR: 1550 1428 1247 992 855 751 691.
m.p. 33-35 °C.

Data is in accordance to literature.[8]

3-Phenylpyridine (24)

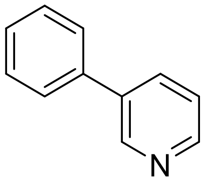

To a vial was added 3-bromopyridine (316 mg, 2.00 mmol), phenylboronic acid (293 mg, 2.40 mmol), $Pd(PPh_3)_2Cl_2$ (70 mg, 0.10 mmol, 0.05 eq.), K_3PO_4 (848 mg, 4.00 mmol) and 1,4-dioxane:water (4:1, 4 mL). The vial was sealed and degassed with nitrogen for 1 h before being heated to 110 °C for 16 h. After being cooled to room temperature, the reaction mixture was filtered through celite and the filtrate was extracted 3 times with ethyl acetate. The combined organic extracts were washed with brine, dried over Na_2SO_4 and concentrated under reduced pressure. The resulting crude mixture was purified by column chromatography on an automatic column machine (hexanes:ethyl acetate) to yield the product as a colorless oil (253 mg, 81%).
^{1}H NMR (400 MHz, Chloroform-*d*) δ 8.86 (dd, *J* = 2.5, 0.9 Hz, 1H), 8.60 (dd, *J* = 4.8, 1.7 Hz, 1H), 7.88 (ddd, *J* = 7.9, 2.4, 1.6 Hz, 1H), 7.62 – 7.55 (m, 2H), 7.53 – 7.45 (m, 2H), 7.45 – 7.33 (m, 2H).
^{13}C NMR (101 MHz, Chloroform-*d*) δ 148.39, 148.28, 137.90, 136.90, 134.70, 129.25 (2C), 128.31, 127.31(2C), 123.77.
HRMS: Calculated for: $C_{11}H_{10}N$ $[M+H]^+$ 156.0808, found 156.0804.
IR: 3030 1581 1472 1450 1407 1024 1006 812 752 709 696 638.
Data is accordance to literature. [9]

(4-(Bromomethyl)phenoxy)triisopropylsilane (25)

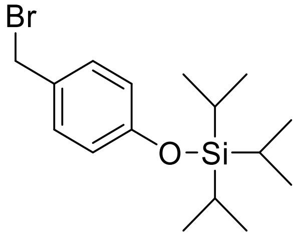

Prepared following a literature procedure.[10] To a flask charged with 4-hyrdoxybenaldehyde (2.44 g, 20 mmol) and imidazole (2.00 g, 30 mmol) in DCM (50 mL) was added triisopropylsilyl chloride (5.20 mL, 24 mmol) and stirred for 16 h at 40 °C. The reaction mixture was then cooled to room temperature and filtered through a pad of silica and then concentrated under reduced pressure. The resulting crude mixture was dissolved in ethanol (40 mL) and sodium borohydride (1.60 g, 40 mmol) was added in portions at room temperature. The resulting mixture was stirred at room temperature until completion by TLC. To the reaction mixture was added water and brine and allowed to stir for 15 min. The mixture was extracted 3 times with ethyl acetate and the combined organic extracts were washed with brine, dried over Mg_2SO_4 and concentrated under reduced pressure. The resulting crude mixture was dissolved in dry diethyl ether (100 mL) and PBr_3 (2.30 mL, 24 mmol) was added slowly at 0 °C. The reaction mixture was allowed to stir for 30 min at 0 °C before being added to a cold saturated solution of $NaHCO_3$ and extracted 3 times with diethyl ether. The combined organic extracts were washed with brine, dried over Mg_2SO_4 and concentrated under reduced pressure. The crude mixture was filtered through a pad of silica (hexane:diethyl ether, 95:5) to give the titled compound as a colorless oil (3.32 g, 48% over 3 steps).
^{1}H NMR (400 MHz, Chloroform-*d*) δ 7.26 (d, *J* = 8.5 Hz, 2H), 6.85 (d, *J* = 8.5 Hz, 2H), 4.49 (s, 2H), 1.35 – 1.20 (m, 3H), 1.11 (d, *J* = 7.4 Hz, 18H).

^{13}C NMR (101 MHz, Chloroform-*d*) δ 156.43, 130.51 (2C), 130.34, 120.24 (2C), 34.18, 18.03 (6C), 12.79 (3C).
HRMS: Calculated for: $C_{16}H_{28}OBrSi$ $[M+H]^+$ 343.1076, found 343.1087.
IR: 2944 2866 1607 1508 1463 1267 1232 1202 1169 908 881 837 672.
Data is accordance to literature. [10]

2-Phenyl-6,7-dihydro-5*H*-pyrrolo[2,1-c][1,2,4]triazol-2-ium tetrafluoroborate (pre-NHC A)

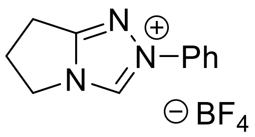

Prepared following a literature procedure.[11] To a flask charged with pyrrolidin-2-one (1.40 mL, 18.1 mmol) in DCM (120 mL) was added trimethyloxonium tetrafluoroborate (2.96 g, 20.0 mmol) and stirred overnight at room temperature. Then to this mixture was added phenylhydrazine (1.80 mL, 18.1 mmol) and allowed to stir for 5 days at room temperature. The solvent was removed under reduced pressure and the residue was redissolved in MeOH (10 mL). To this mixture was added triethylorthoformate (40 mL) and refluxed overnight at 100 °C. The reaction mixture was allowed to cool, filtered and the crude residue was purified by recrystallization from MeOH to yield the title compound as a tan solid (2.50 g, 51%).
^{1}H NMR (400 MHz, Methanol-*d*$_4$) δ 7.92 – 7.81 (m, 2H), 7.69 – 7.56 (m, 3H), 4.50 (t, *J* = 7.4 Hz, 2H), 3.24 (t, *J* = 7.7 Hz, 2H), 2.86 (p, *J* = 7.6 Hz, 2H).
^{13}C NMR (101 MHz, Methanol-*d*$_4$) δ 164.81, 137.35, 131.74, 131.25, 122.09, 48.38, 27.80, 22.42.
^{19}F NMR (376 MHz, Methanol-*d*$_4$) δ -154.52, -154.58.
HRMS: Calculated for: $C_{11}H_{12}N_3$ $[M]^+$ 186.1026, found 186.1019.
IR: 1585 1508 1386 1078 1047 1035 776.
m.p. 159-160 °C.
Data is accordance to literature. [11]

References

[1] M. S. Kerr, J. Read de Alaniz, T. Rovis, *J. Org. Chem.* **2005**, *70*, 5725–5728.

[2] J. Arduengo, R. Krafczyk, R. Schmutzler, H. A. Craig, J. R. Goerlich, W. J. Marshall, M. Unverzagt, *Tetrahedron* **1999**, *55*, 14523–14534.

[3] I. Piel, M. D. Pawelczyk, K. Hirano, R. Fröhlich, F. Glorius, *European Journal of Organic Chemistry* **2011**, *2011*, 5475–5484.

[4] Q.-Y. Meng, N. Döben, A. Studer, *Angewandte Chemie International Edition* **2020**, *59*, 19956–19960.

[5] J. K. Laha, K. P. Jethava, S. Patel, K. V. Patel, *J. Org. Chem.* **2017**, *82*, 76–85.

[6] J. K. Laha, K. V. Patel, G. Dubey, K. P. Jethava, *Org. Biomol. Chem.* **2017**, *15*, 2199–2210.

[7] J. B. Metternich, D. G. Artiukhin, M. C. Holland, M. von Bremen-Kühne, J. Neugebauer, R. Gilmour, *J. Org. Chem.* **2017**, *82*, 9955–9977.

[8] F. Fontaine, A. Héquet, A.-S. Voisin-Chiret, A. Bouillon, A. Lesnard, T. Cresteil, C. Jolivalt, S. Rault, *European Journal of Medicinal Chemistry* **2015**, *95*, 185–198.

[9] W.-C. Chen, Y.-C. Hsu, W.-C. Shih, C.-Y. Lee, W.-H. Chuang, Y.-F. Tsai, P. P.-Y. Chen, T.-G. Ong, *Chem. Commun.* **2012**, *48*, 6702–6704.

[10] A. Grozavu, H. B. Hepburn, E. P. Bailey, P. J. Lindsay-Scott, T. J. Donohoe, *Chem. Sci.* **2020**, *11*, 8595–8599.

[11] M. S. Kerr, J. Read de Alaniz, T. Rovis, *J. Org. Chem.* **2005**, *70*, 5725–5728.

^{1}H NMR, ^{13}C NMR and ^{19}F NMR Spectra of synthesised compounds

21a

20210429-1301-B400_B.11-41.10.fid
Ref 464-1
Group Greaney_M
H1_Night CDCl3 /mnt/nmrdata/Greaney_M m31962tw 41

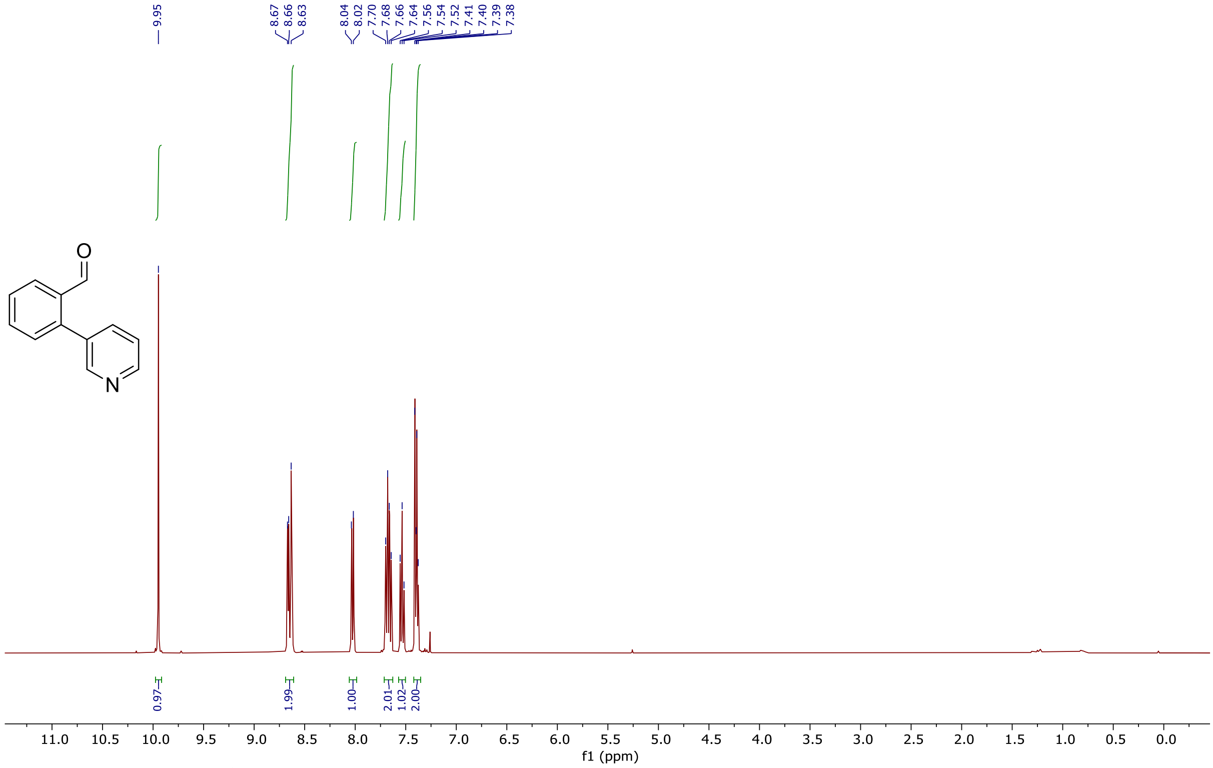

20210429-1301-B400_B.11-41.11.fid
Ref 464-1
Group Greaney_M
C13_CPD_Night256 CDCl3 /mnt/nmrdata/Greaney_M m31962tw 41

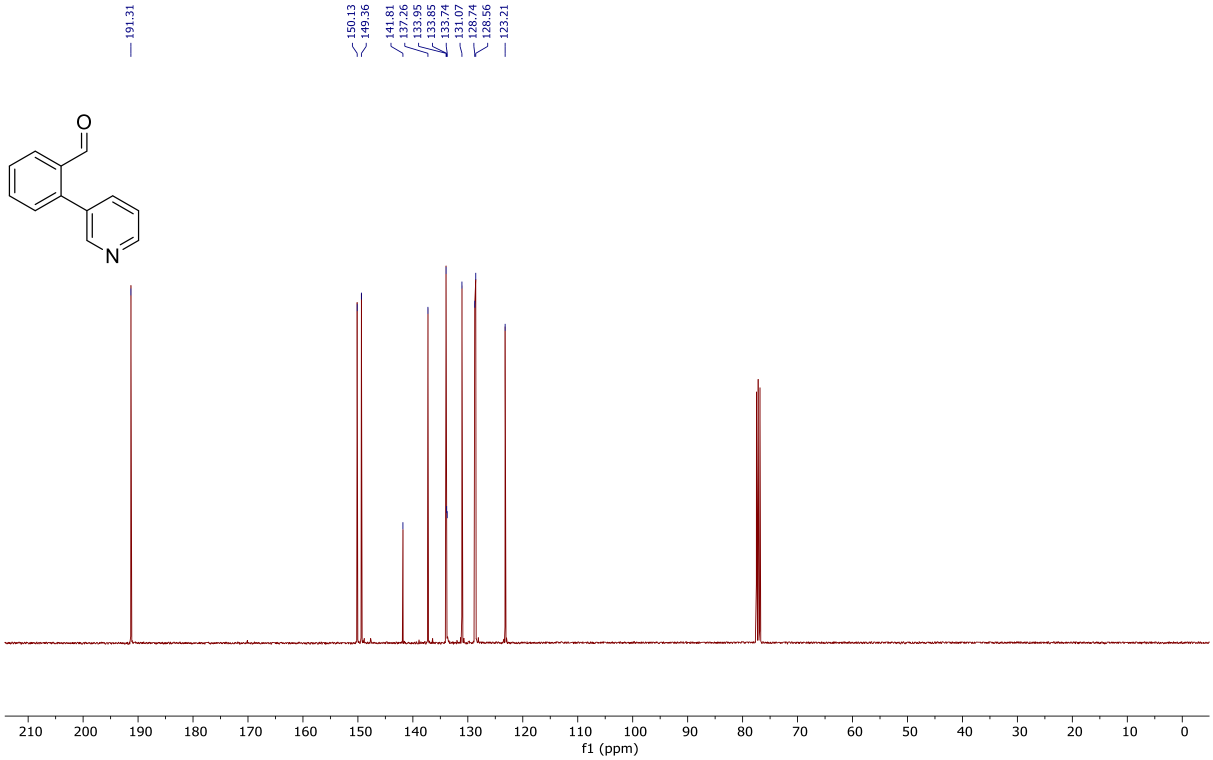

21g

20201204-1643-B400_B.11-48.10.fid
Ref 478-10
Group Greaney_M
H1_Night CDCl3 /mnt/nmrdata/Greaney_M m31962tw 48

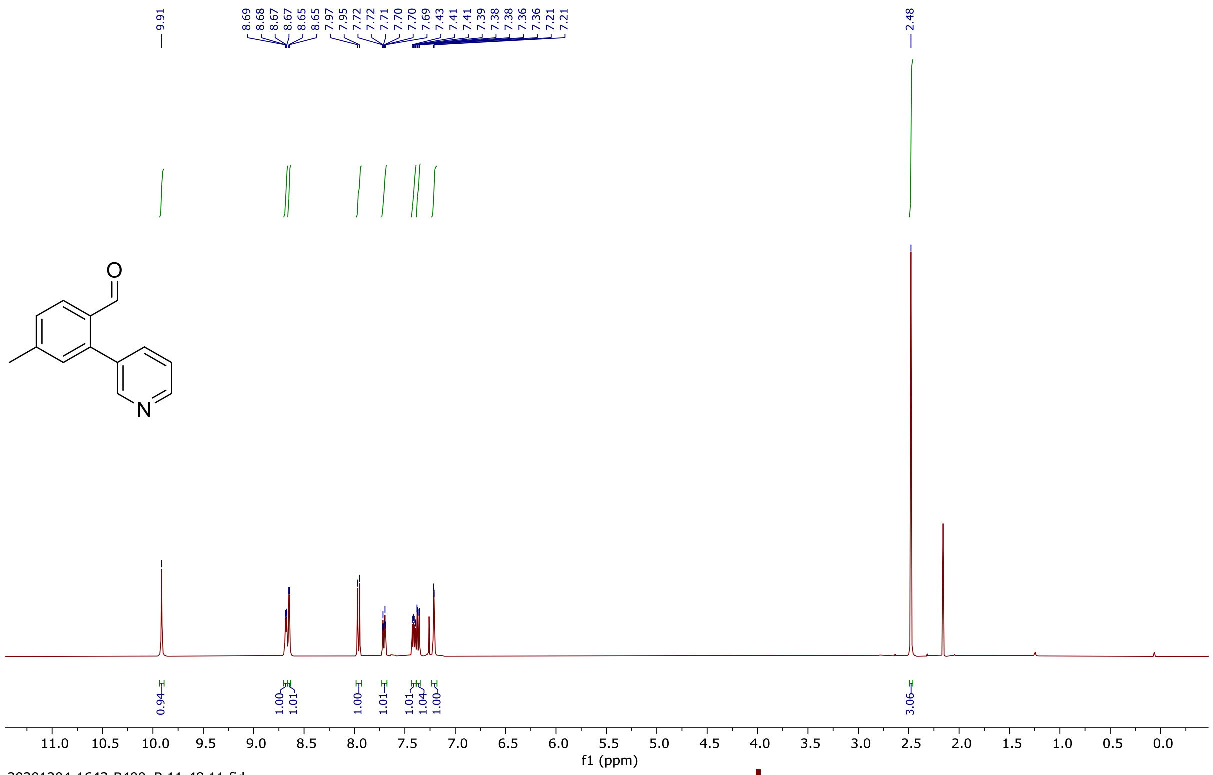

20201204-1643-B400_B.11-48.11.fid
Ref 478-10
Group Greaney_M
C13_CPD_Night256 CDCl3 /mnt/nmrdata/Greaney_M m31962tw 48

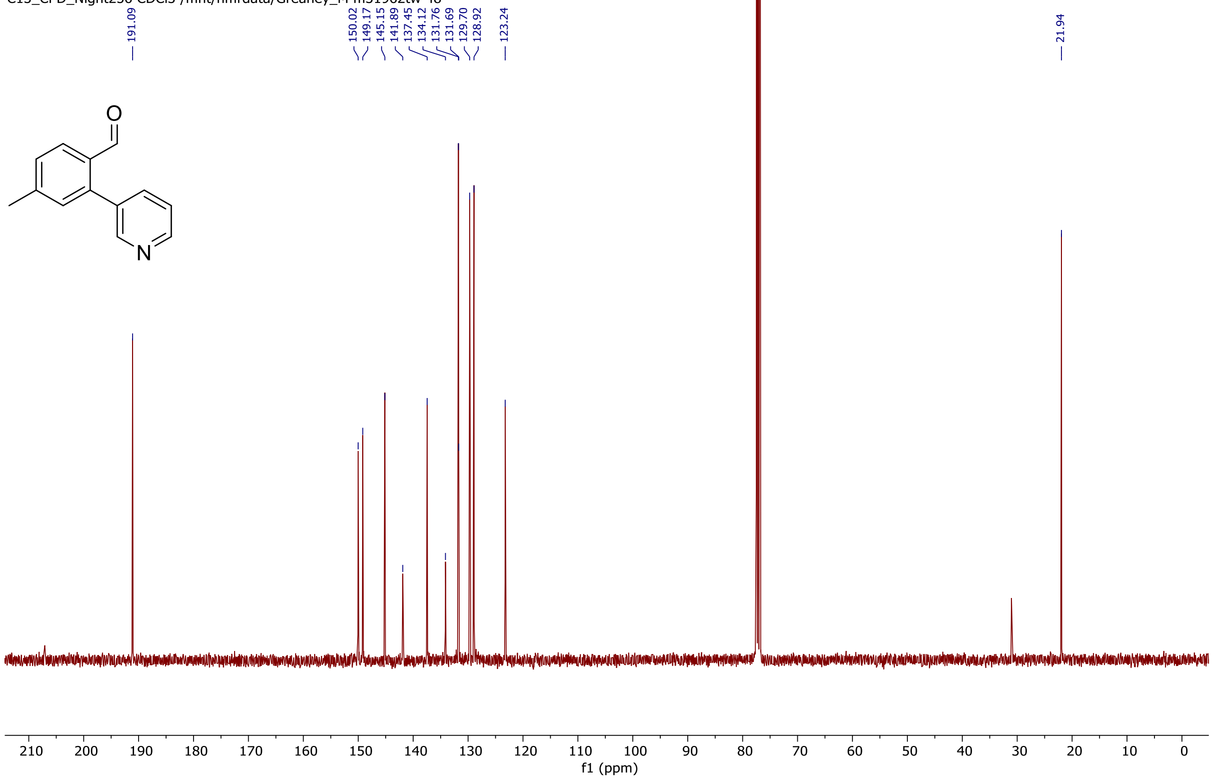

21h

20201204-1643-B400_B.11-47.10.fid
Ref 478-8
Group Greaney_M
H1_Night CDCl3 /mnt/nmrdata/Greaney_M m31962tw 47

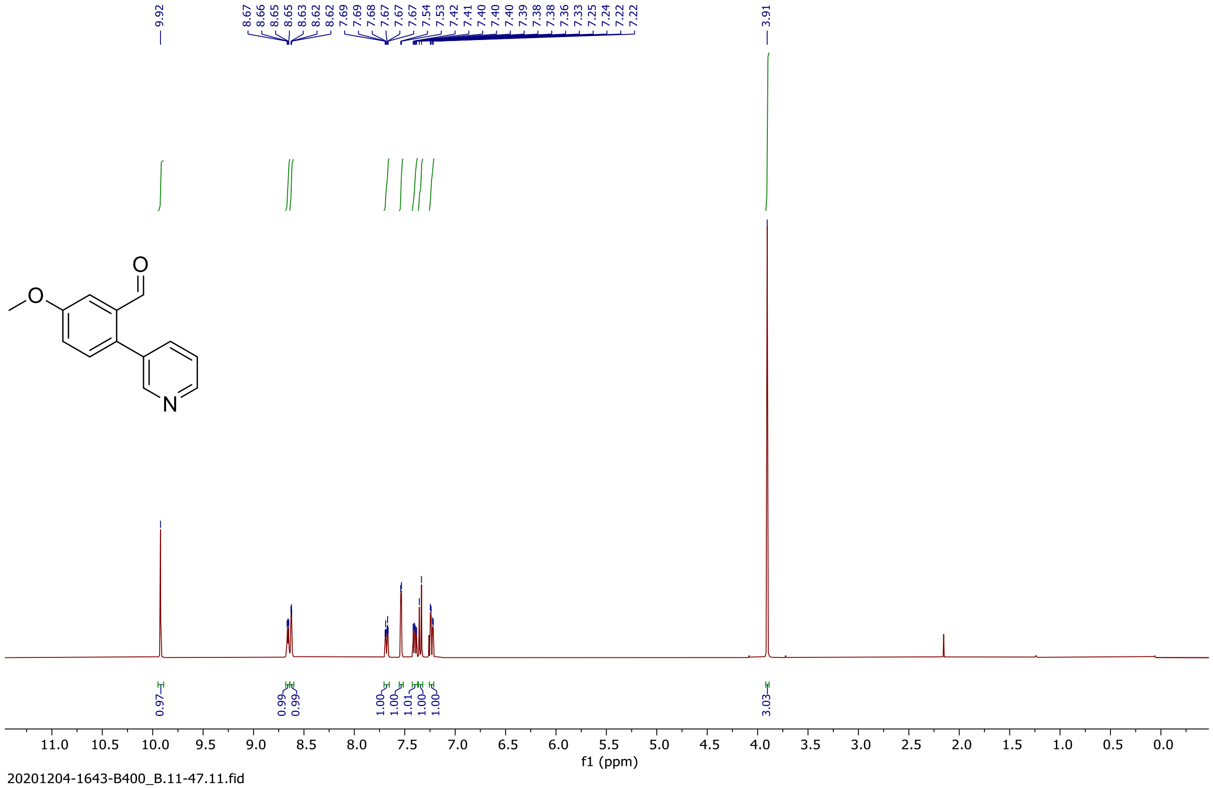

20201204-1643-B400_B.11-47.11.fid
Ref 478-8
Group Greaney_M
C13_CPD_Night256 CDCl3 /mnt/nmrdata/Greaney_M m31962tw 47

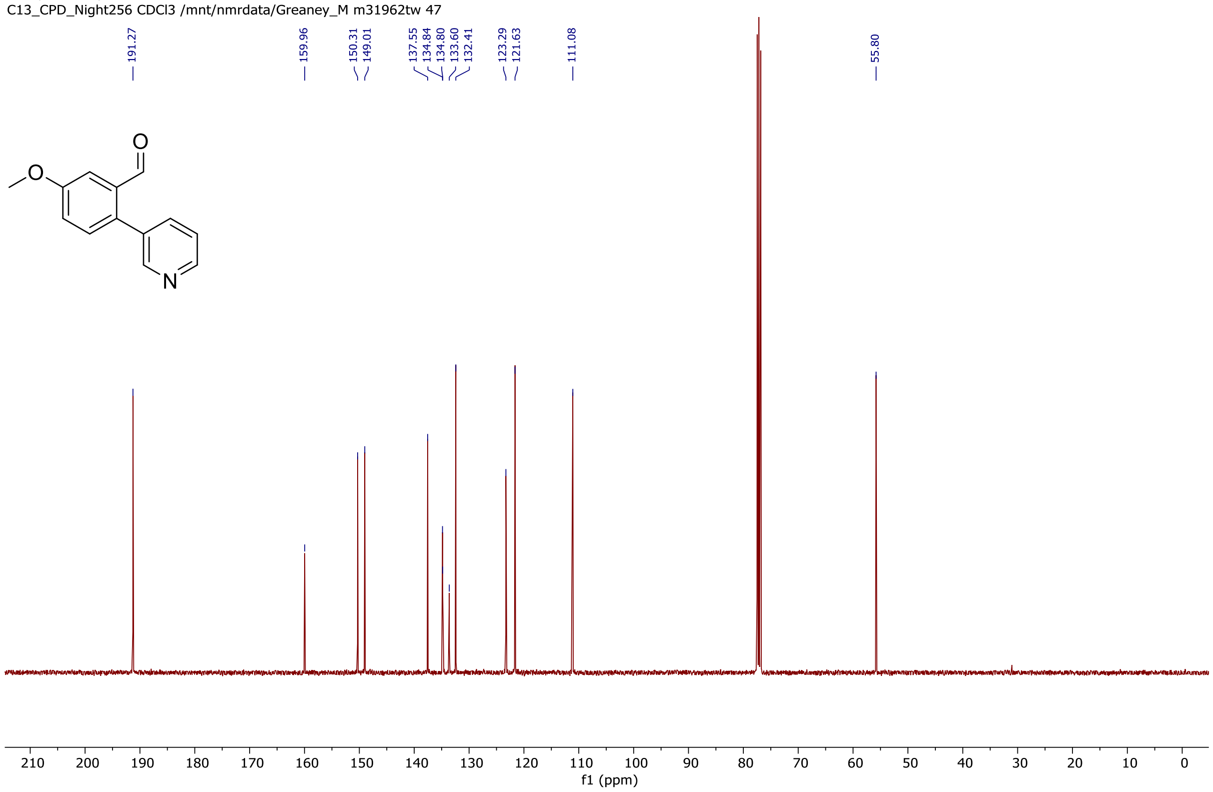

21i

20201204-1642-B400_B.11-45.10.fid
Ref 477-1
Group Greaney_M
H1_Night CDCl3 /mnt/nmrdata/Greaney_M m31962tw 45

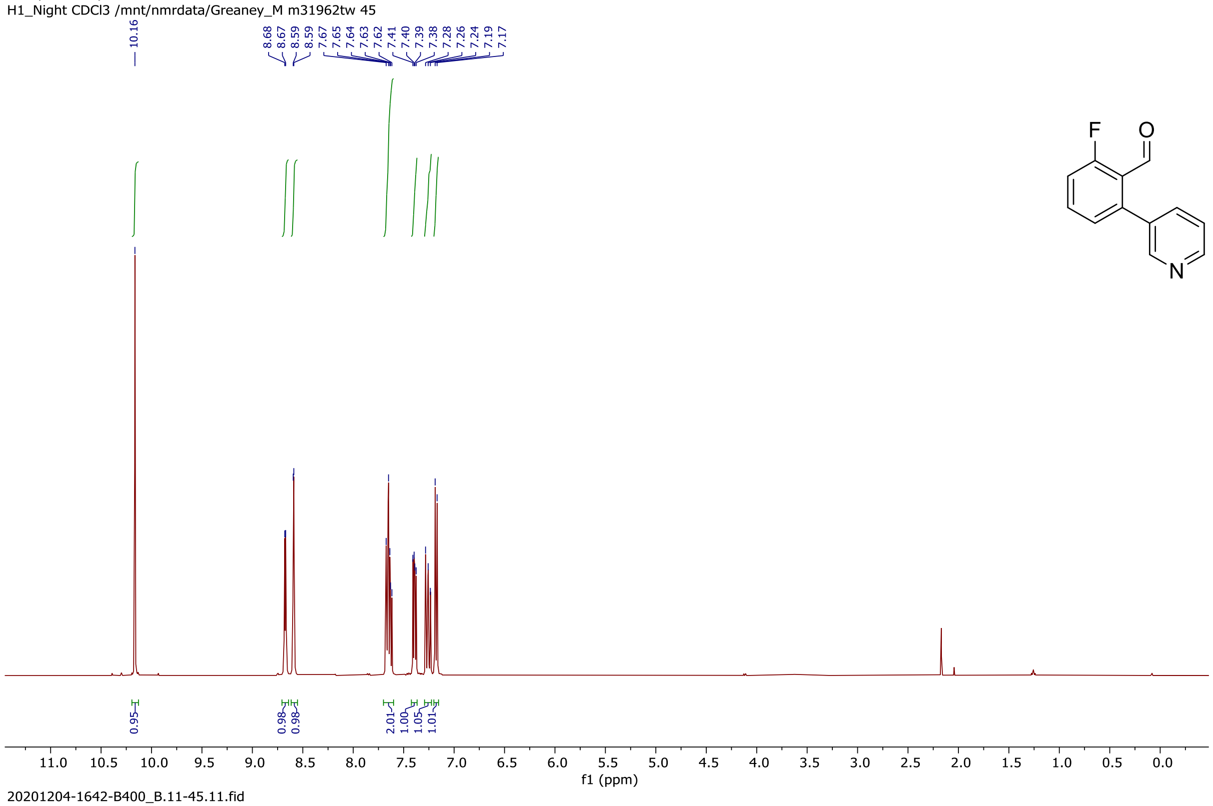

20201204-1642-B400_B.11-45.11.fid
Ref 477-1
Group Greaney_M
C13_CPD_Night256 CDCl3 /mnt/nmrdata/Greaney_M m31962tw 45

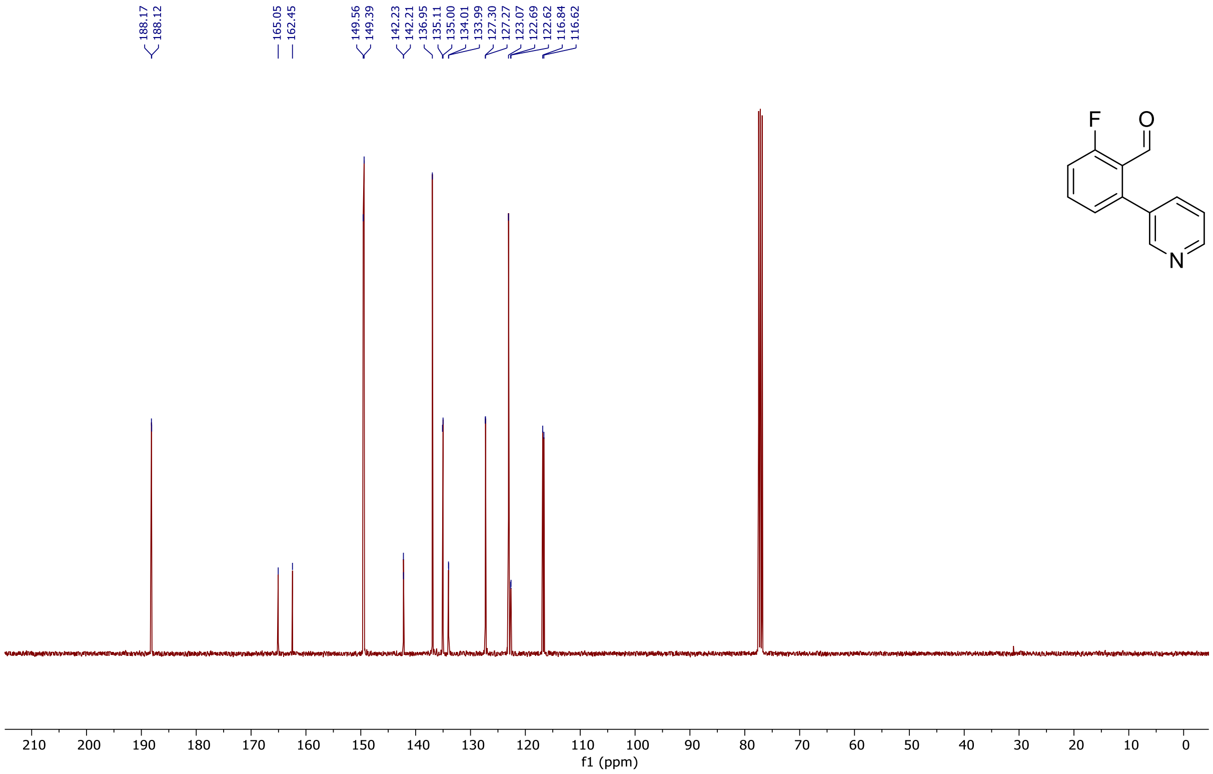

20201204-1642-B400_B.11-45.15.fid
Ref 477-1
Group Greaney_M
F19_NoCPD_Day CDCl3 /mnt/nmrdata/Greaney_M m31962tw 45

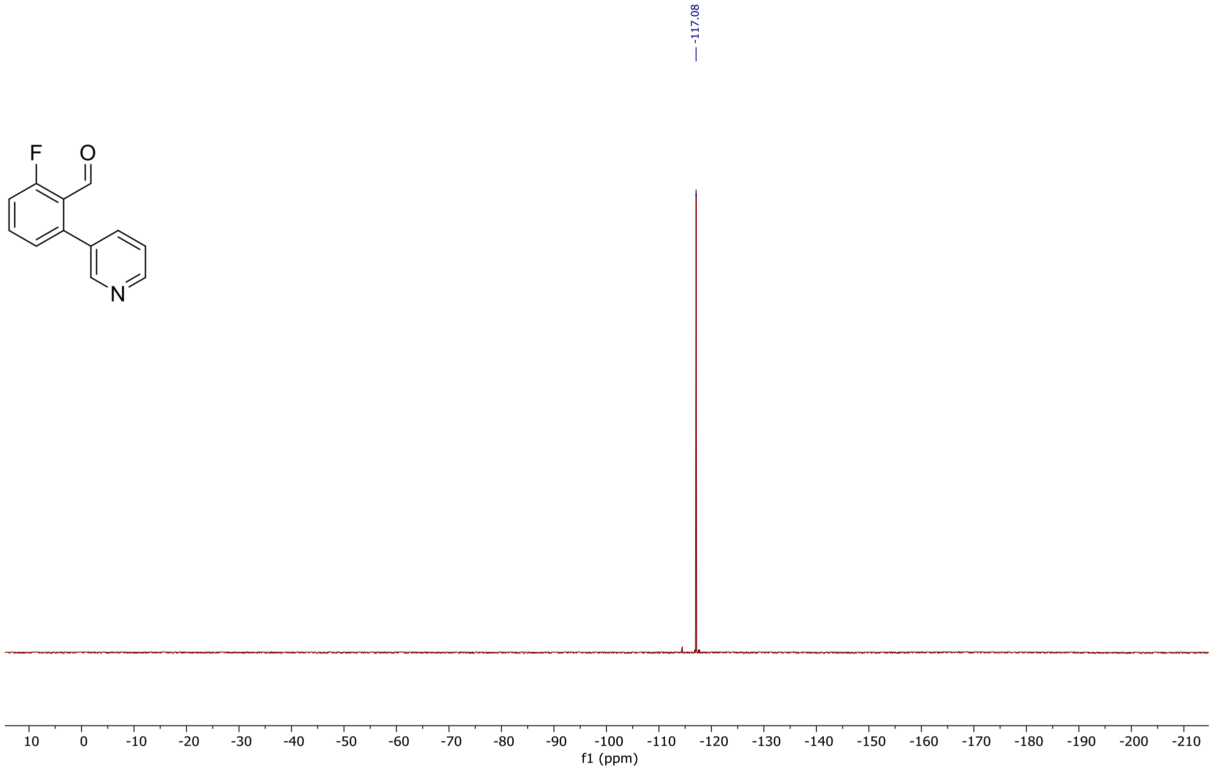

20201204-1644-B400_B.11-50.10.fid
Ref 478-12
Group Greaney_M
H1_Night CDCl3 /mnt/nmrdata/Greaney_M m31962tw 50

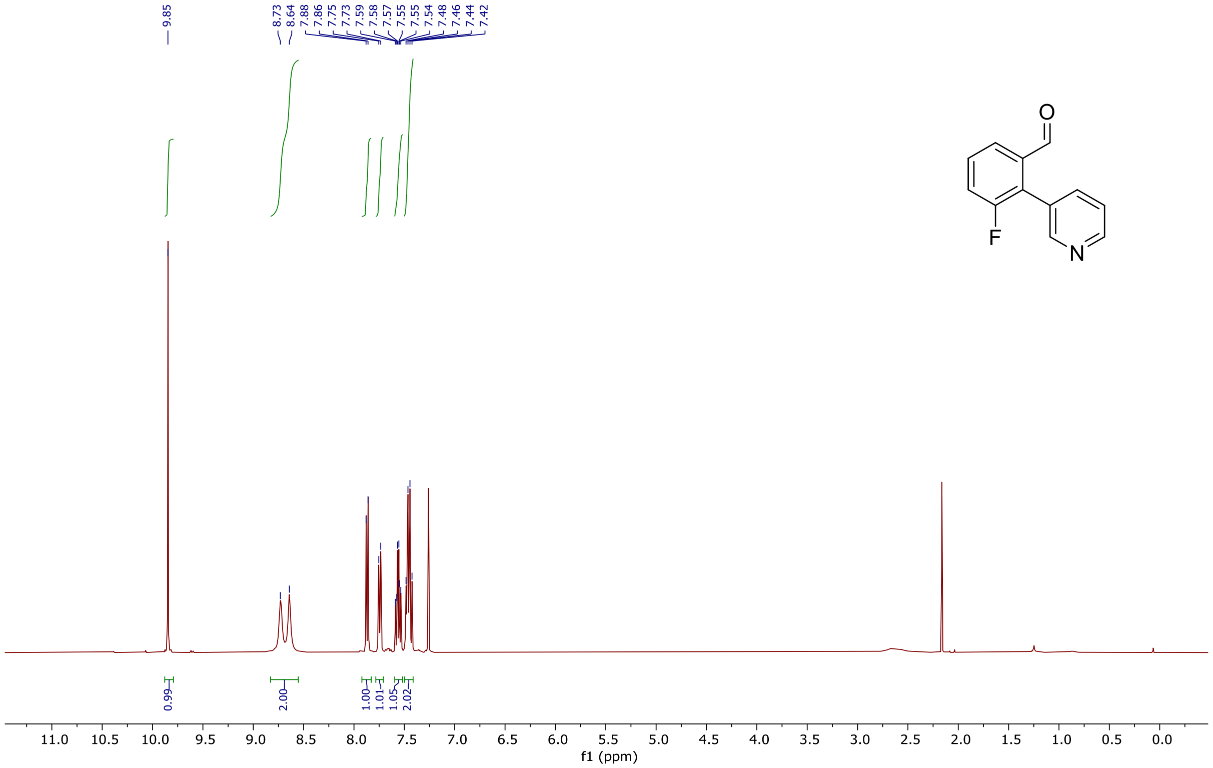

20201204-1644-B400_B.11-50.11.fid
Ref 478-12
Group Greaney_M
C13_CPD_Night256 CDCl3 /mnt/nmrdata/Greaney_M m31962tw 50

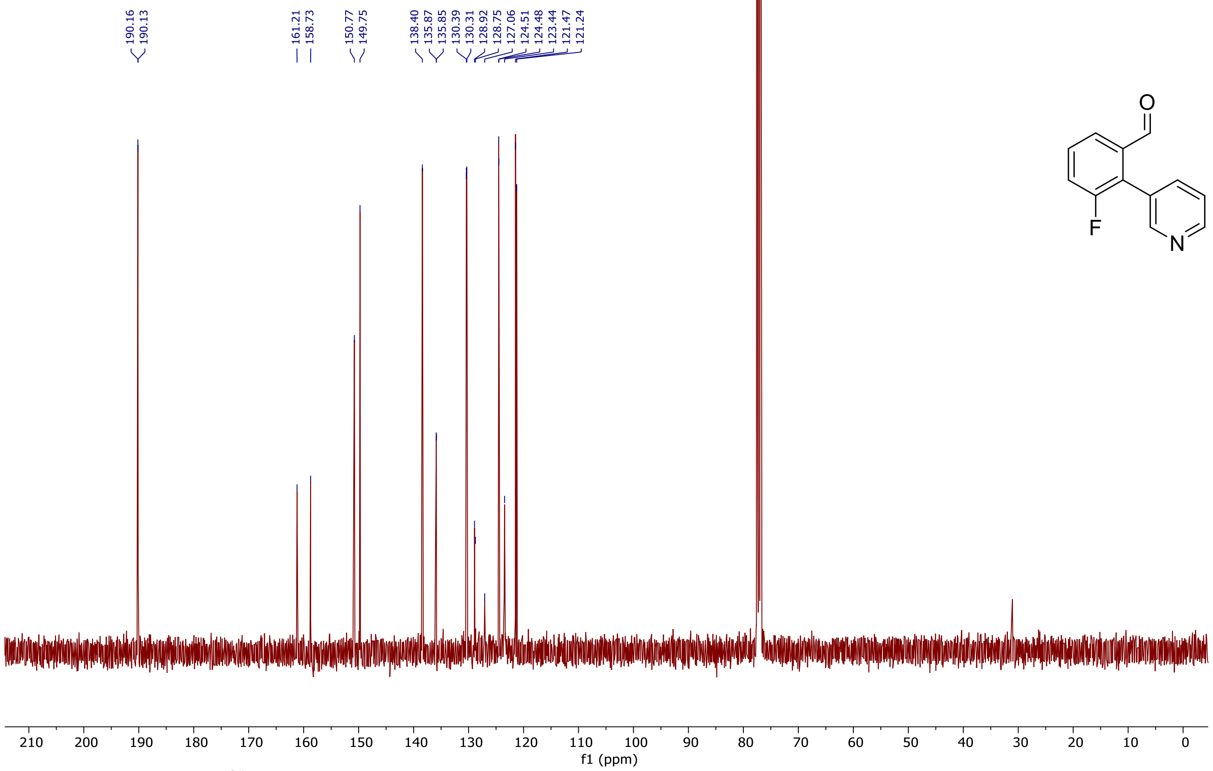

20201204-1644-B400_B.11-50.15.fid
Ref 478-12
Group Greaney_M
F19_NoCPD_Day CDCl3 /mnt/nmrdata/Greaney_M m31962tw 50

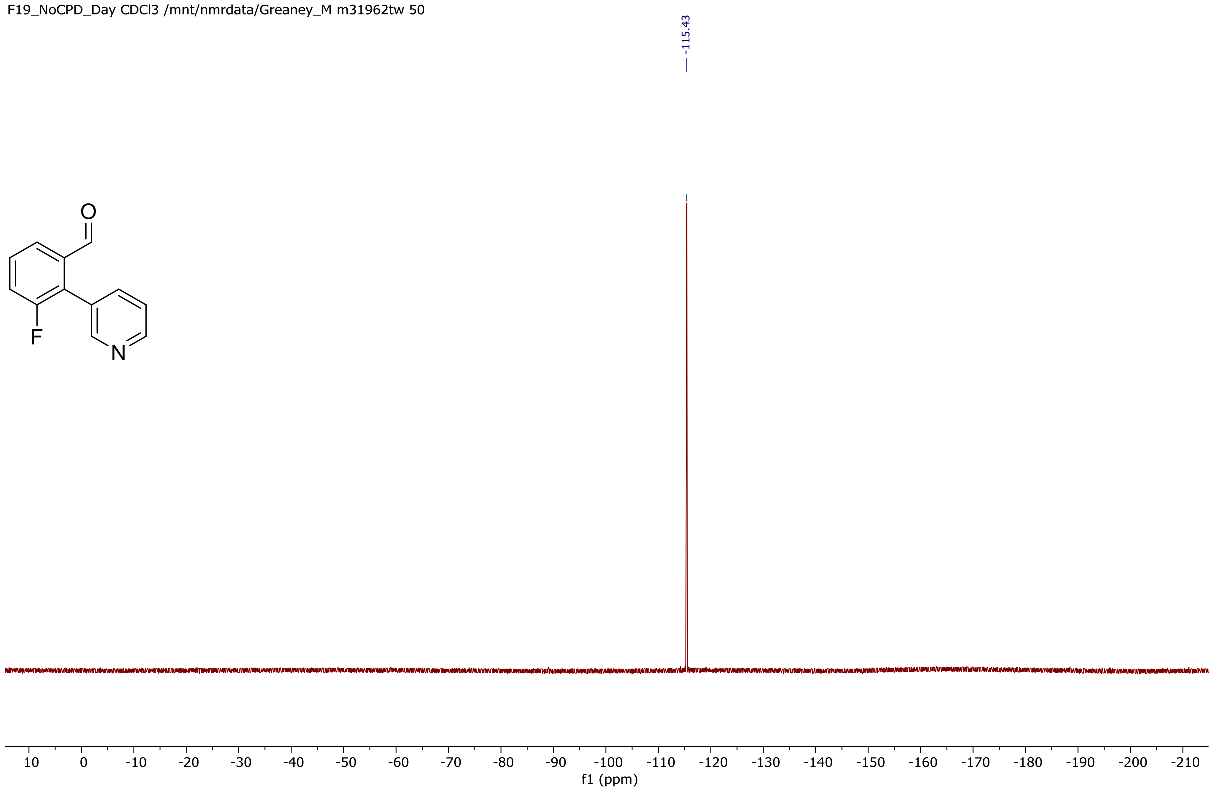

21k

20201204-1642-B400_B.11-46.10.fid
Ref 478-7
Group Greaney_M
H1_Night CDCl3 /mnt/nmrdata/Greaney_M m31962tw 46

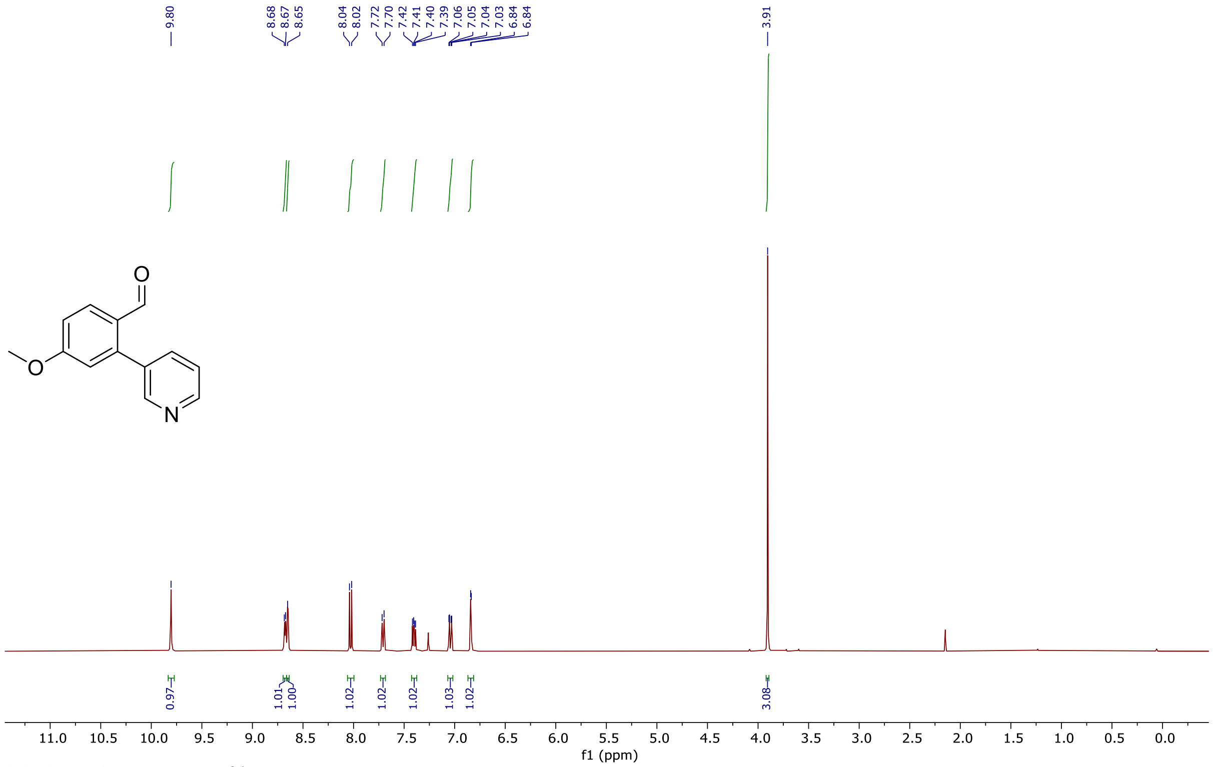

20201204-1642-B400_B.11-46.11.fid
Ref 478-7
Group Greaney_M
C13_CPD_Night256 CDCl3 /mnt/nmrdata/Greaney_M m31962tw 46

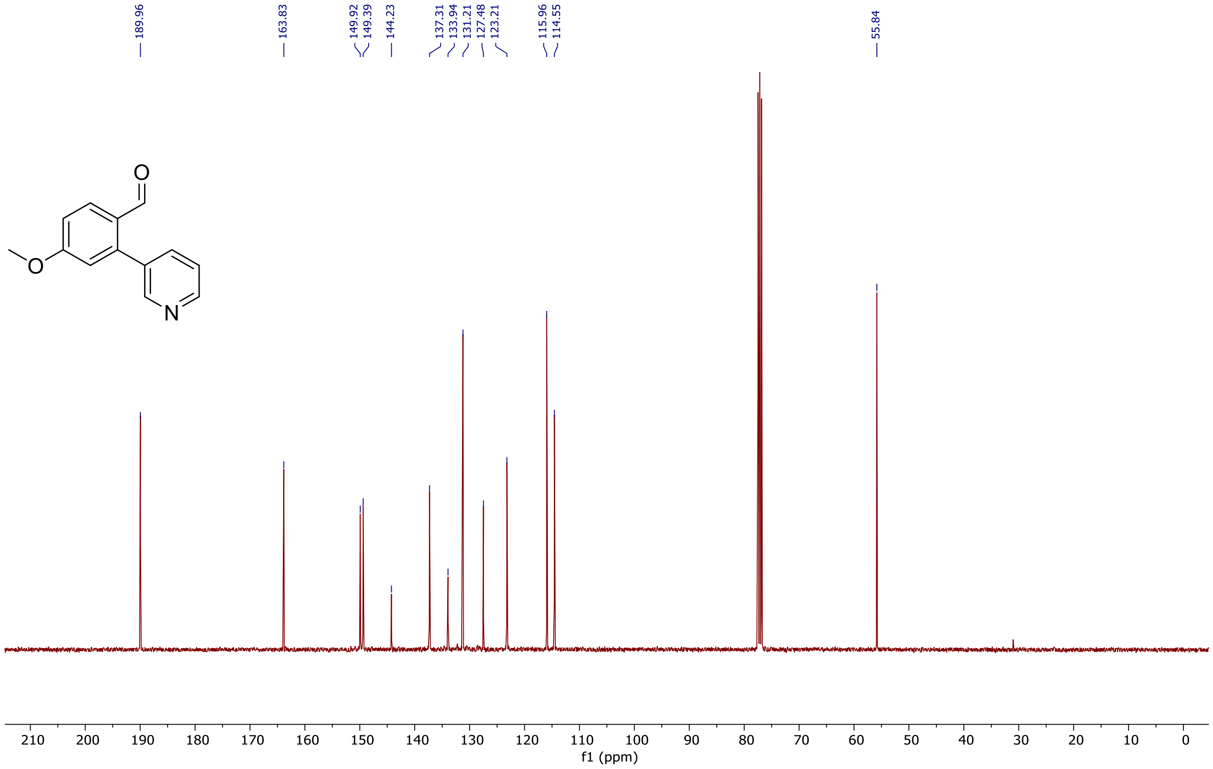

21m

20201204-1644-B400_B.11-49.10.fid
Ref 478-11
Group Greaney_M
H1_Night CDCl3 /mnt/nmrdata/Greaney_M m31962tw 49

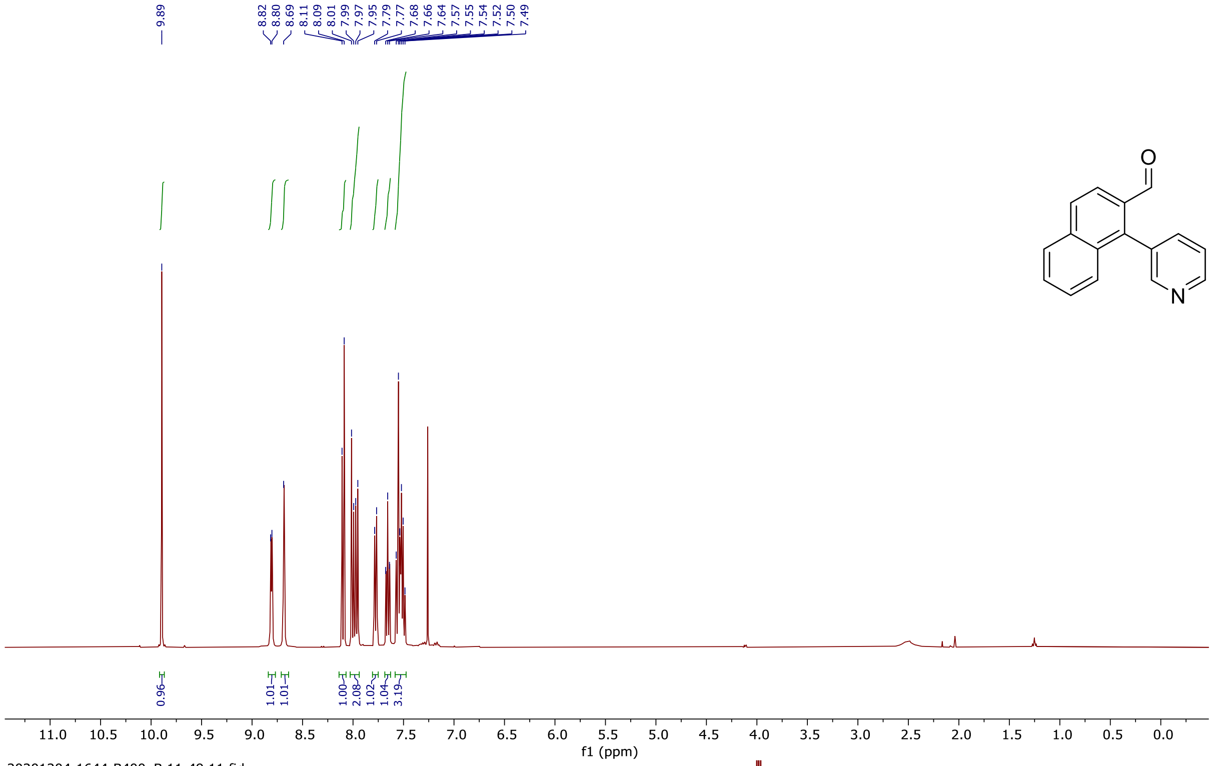

20201204-1644-B400_B.11-49.11.fid
Ref 478-11
Group Greaney_M
C13_CPD_Night256 CDCl3 /mnt/nmrdata/Greaney_M m31962tw 49

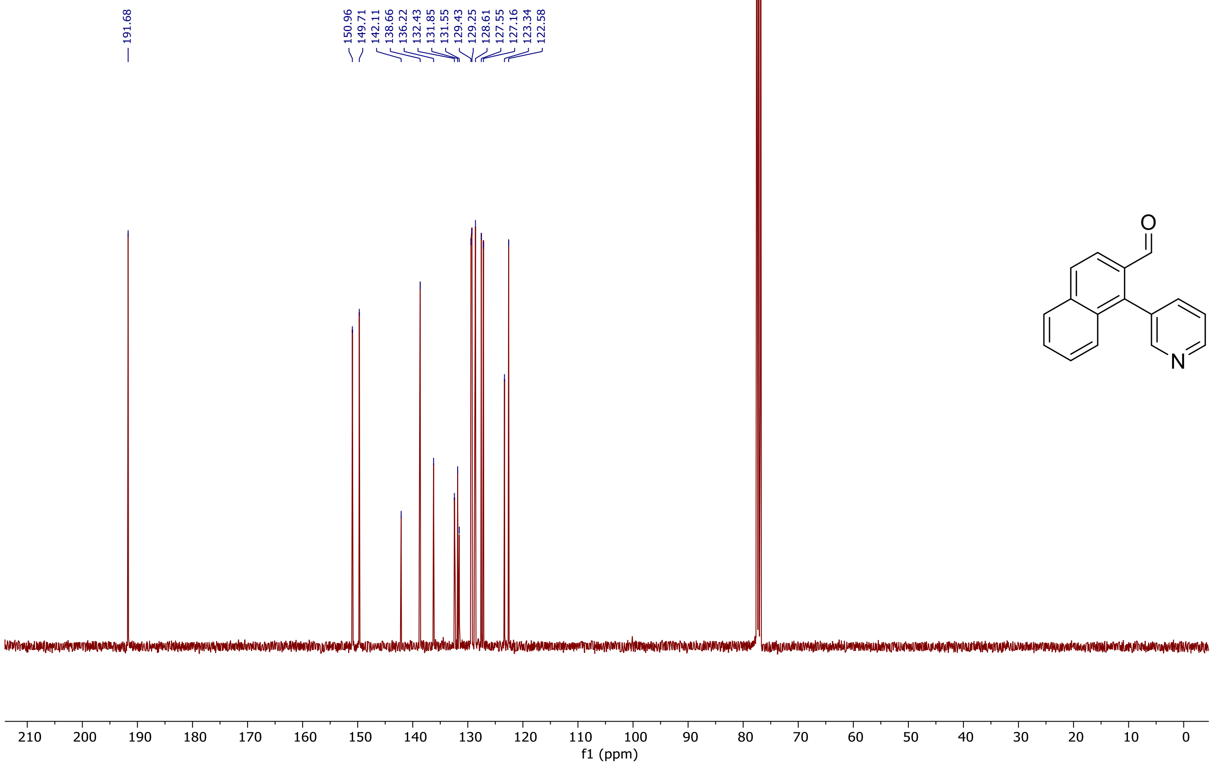

21r

20200930-1715-B400_B.11-3.10.fid
Ref 419-3
Group Greaney_M
H1_Day CDCl3 /mnt/nmrdata/Greaney_M m31962tw 3

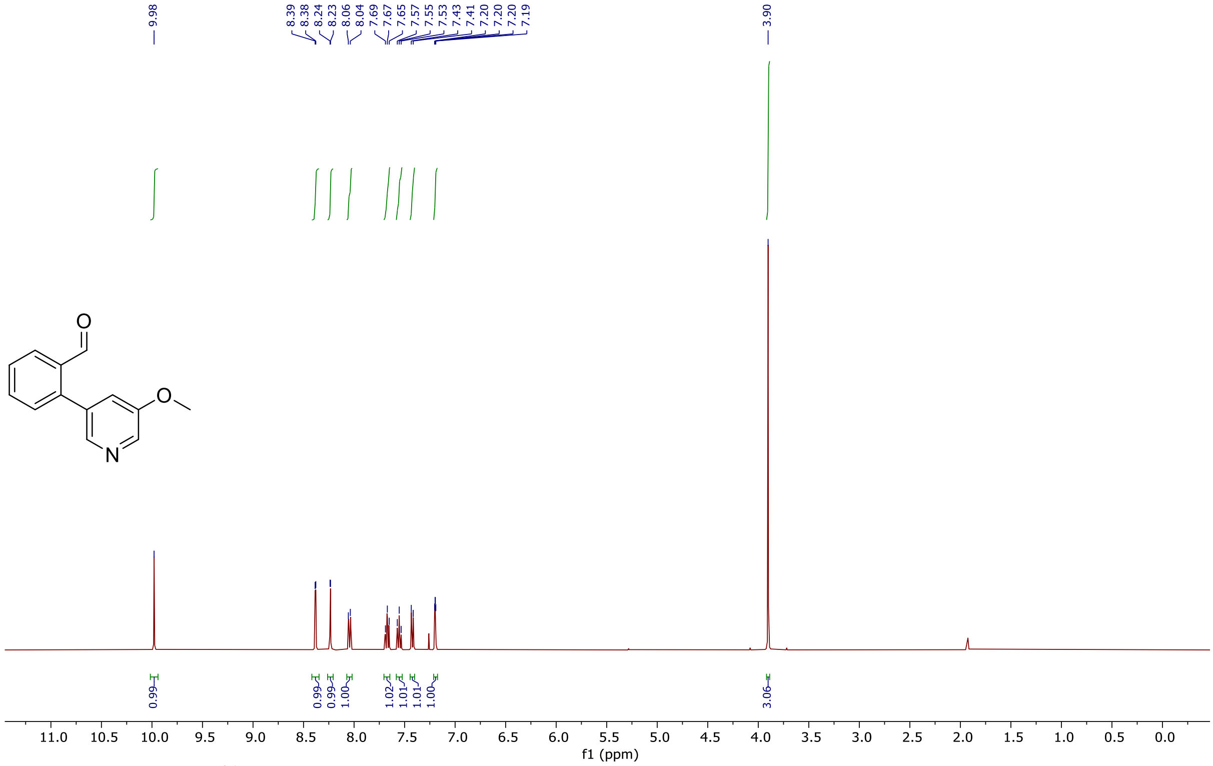

20200930-1715-B400_B.11-3.11.fid
Ref 419-3
Group Greaney_M
C13_CPD_Night256 CDCl3 /mnt/nmrdata/Greaney_M m31962tw 3

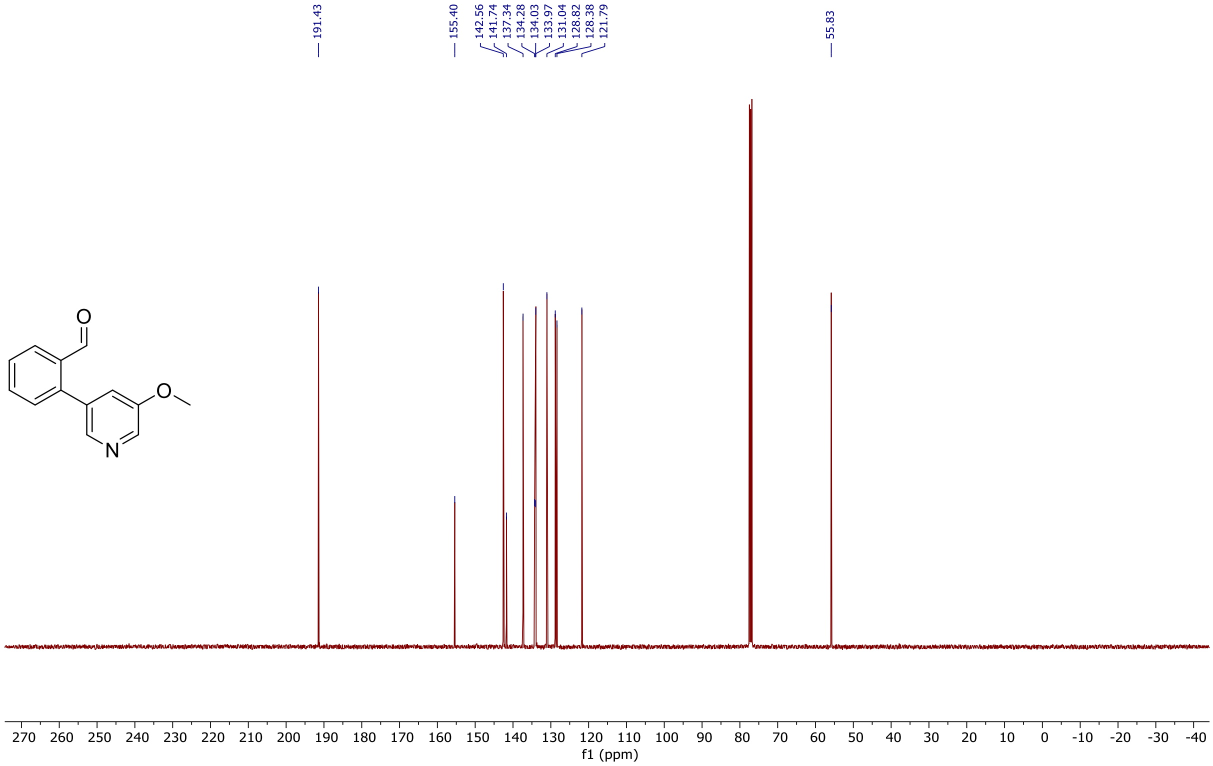

21s

20210414-1651-B400_B.12-43.10.fid
Ref 525-2
Group Greaney_M
H1_Night CDCl3 /mnt/nmrdata/Greaney_M m31962tw 43

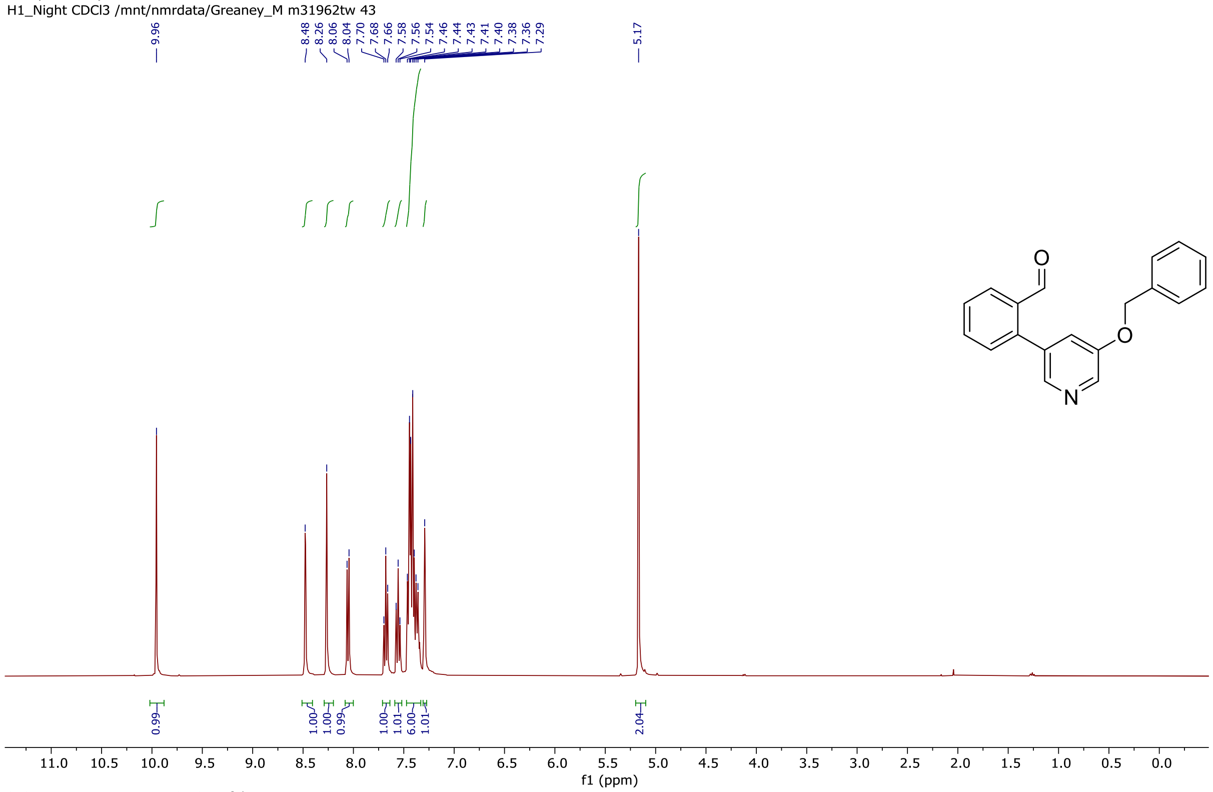

20210414-1651-B400_B.12-43.11.fid
Ref 525-2
Group Greaney_M
C13_CPD_Night256 CDCl3 /mnt/nmrdata/Greaney_M m31962tw 43

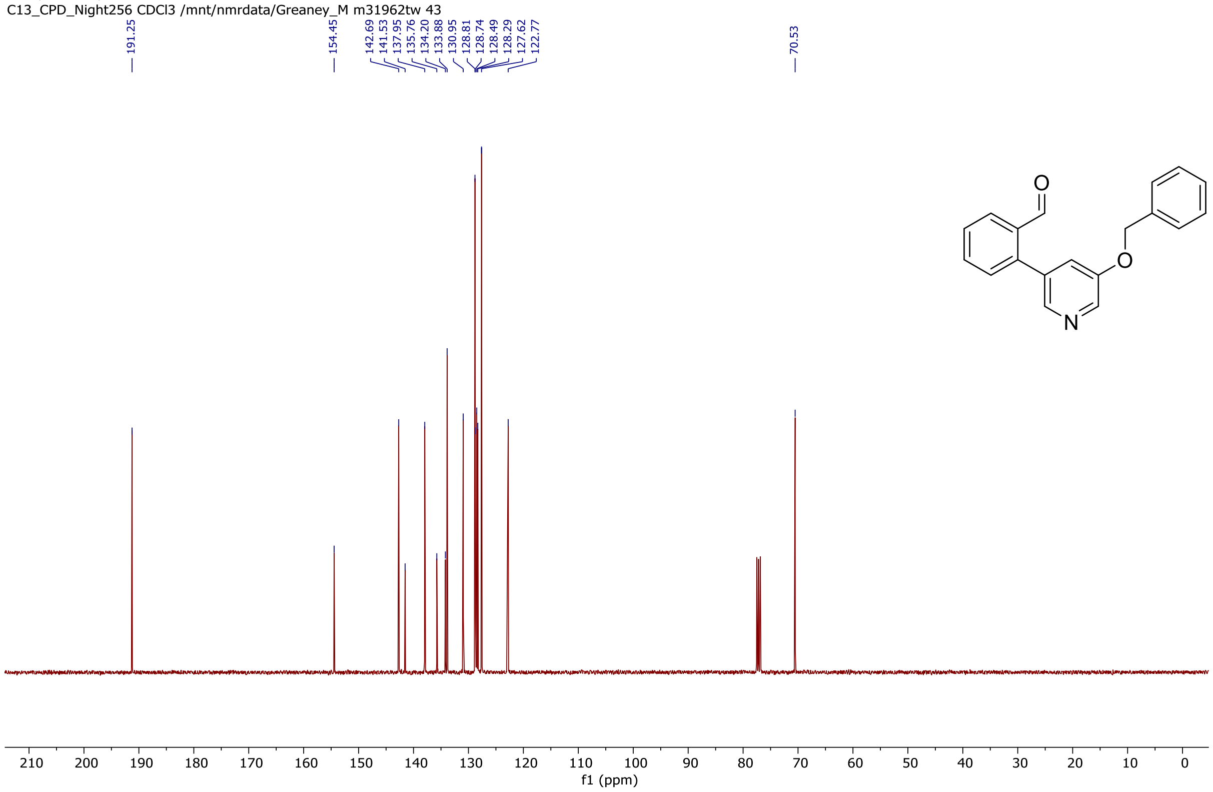

21t

20210415-1827-B400_B.12-28.10.fid
Ref 528-3
Group Greaney_M
H1_Night CDCl3 /mnt/nmrdata/Greaney_M m31962tw 28

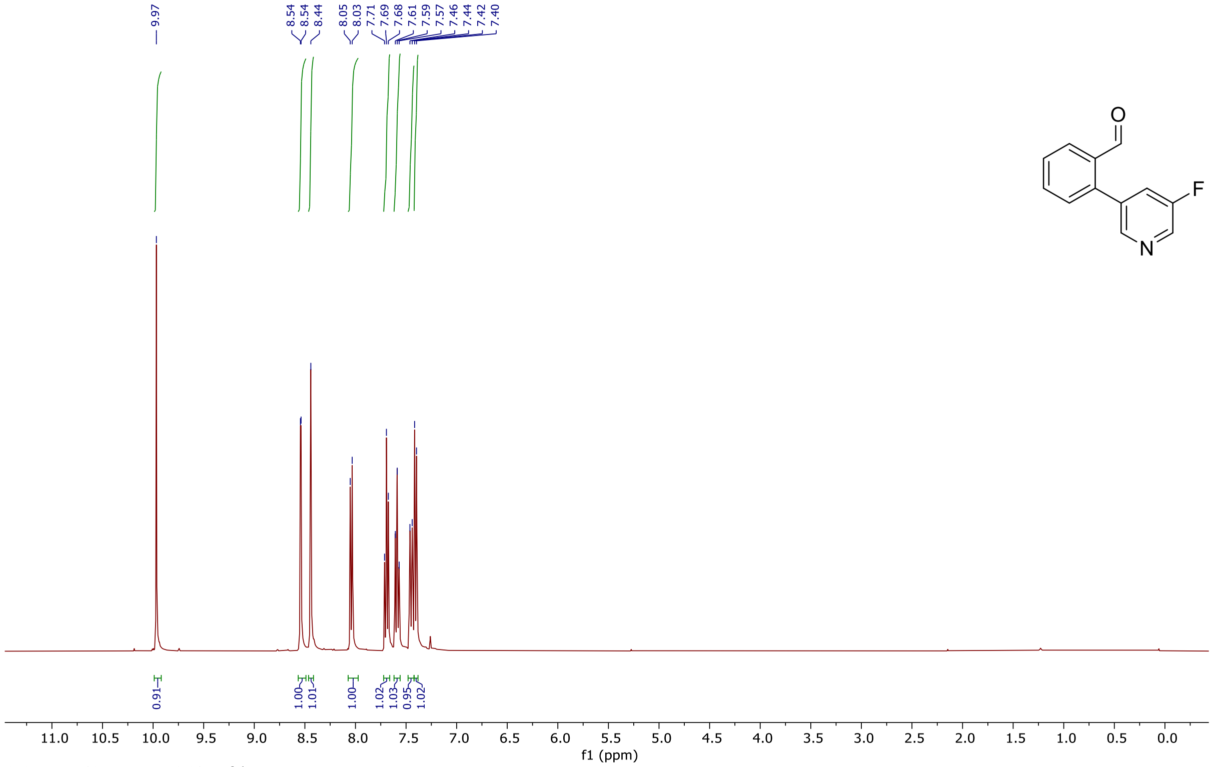

20210415-1827-B400_B.12-28.11.fid
Ref 528-3
Group Greaney_M
C13_CPD_Night256 CDCl3 /mnt/nmrdata/Greaney_M m31962tw 28

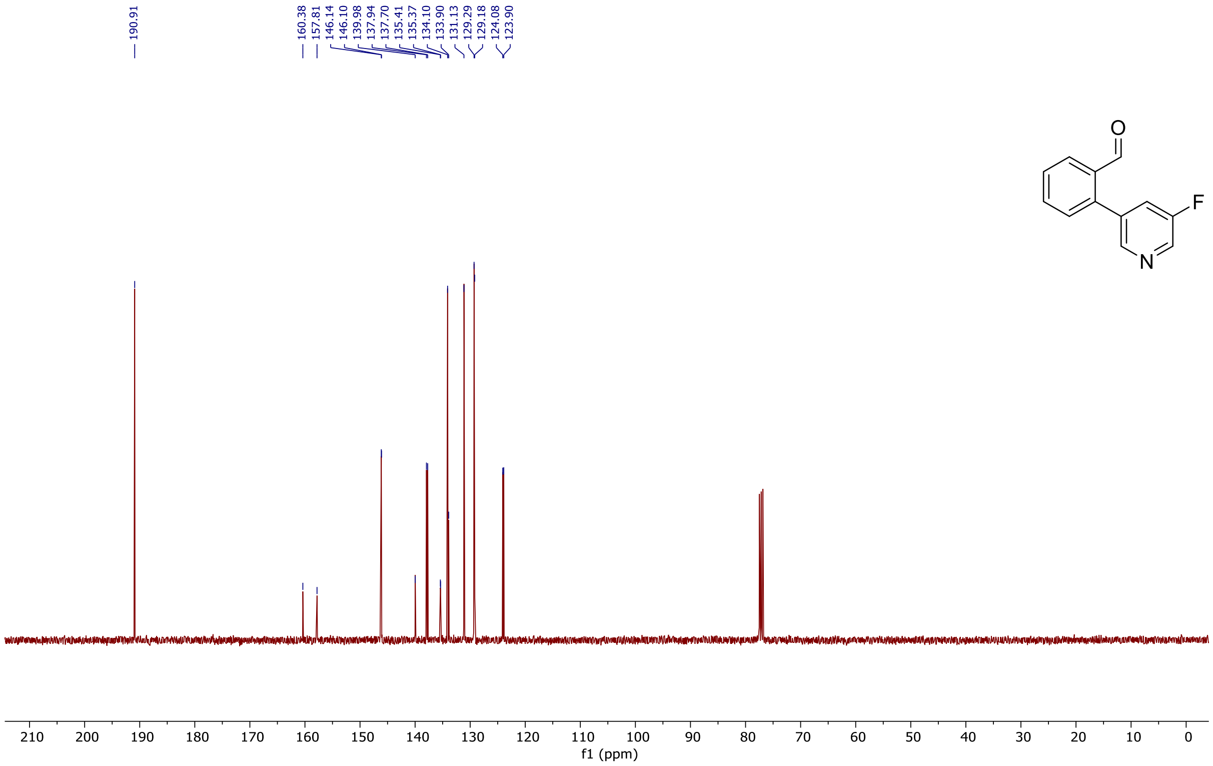

20210415-1827-B400_B.12-28.12.fid
Ref 528-3
Group Greaney_M
F19_CPD_Night CDCl3 /mnt/nmrdata/Greaney_M m31962tw 28

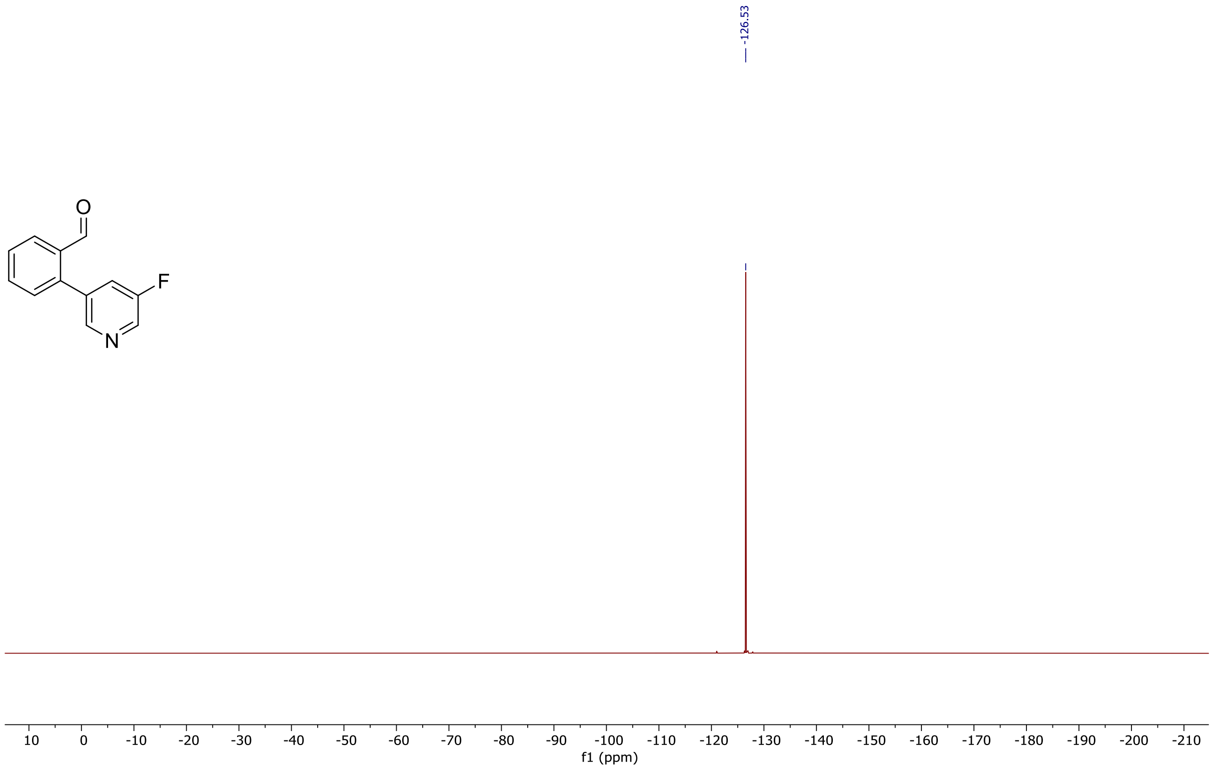

21u

20210415-1827-B400_B.12-27.11.fid
Ref 525-1
Group Greaney_M
H1_Night CDCl3 /mnt/nmrdata/Greaney_M m31962tw 27

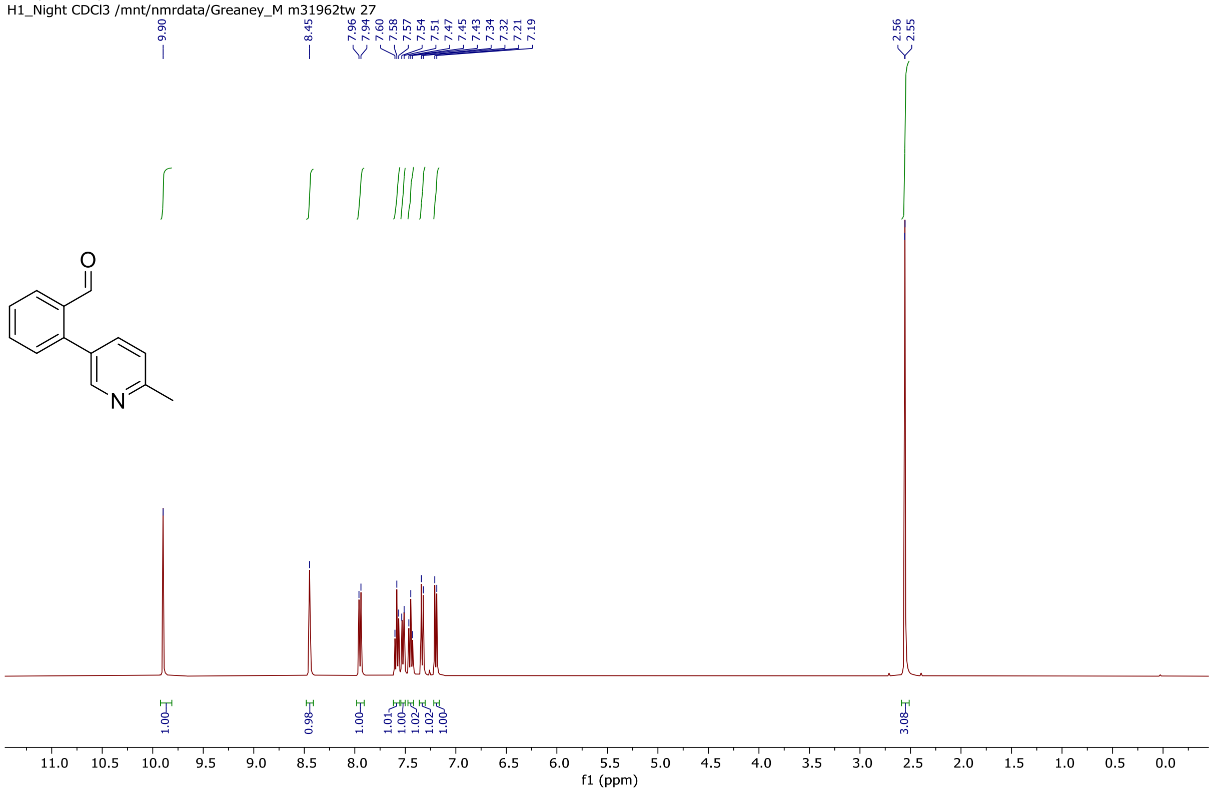

20210415-1827-B400_B.12-27.12.fid
Ref 525-1
Group Greaney_M
C13_CPD_Night256 CDCl3 /mnt/nmrdata/Greaney_M m31962tw 27

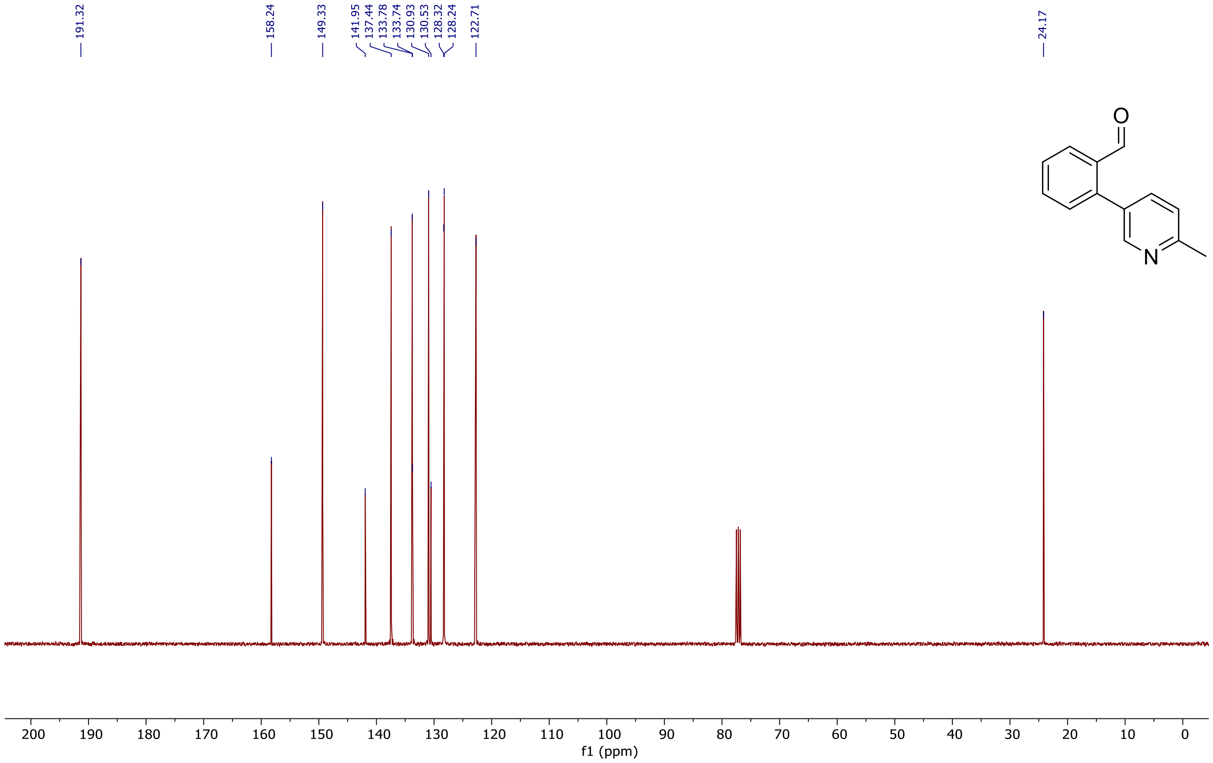

21w

20210812-1632-B400_B.11-20.10.fid
Ref 465-3
Group Greaney_M
H1_Night CDCl3 /mnt/nmrdata/Greaney_M m31962tw 20

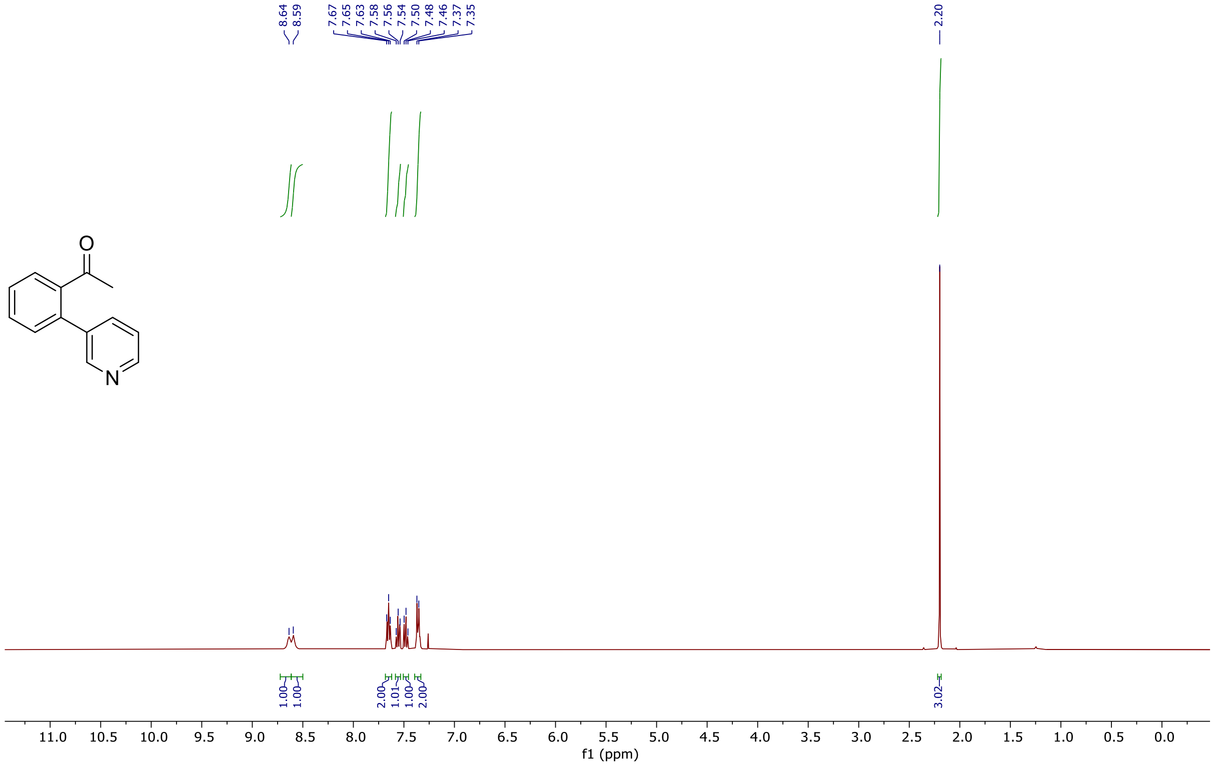

20210812-1632-B400_B.11-20.11.fid
Ref 465-3
Group Greaney_M
C13_CPD_Night256 CDCl3 /mnt/nmrdata/Greaney_M m31962tw 20

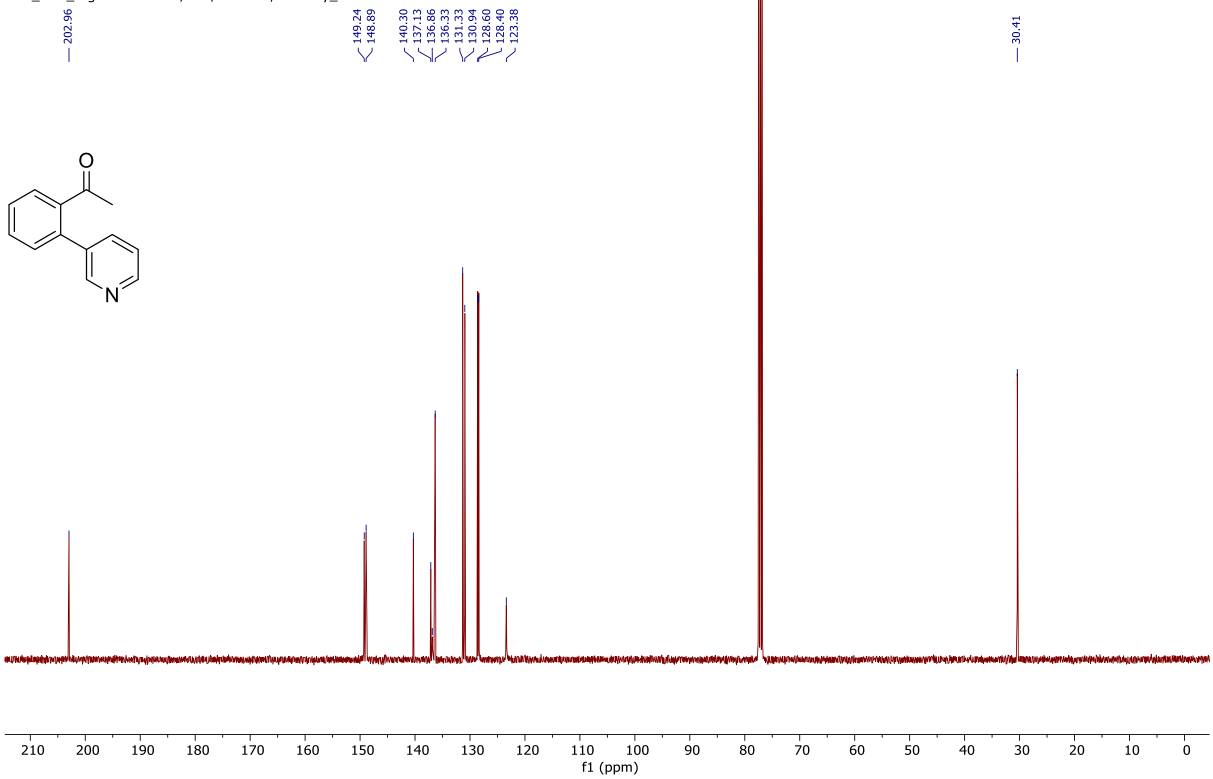

22a

20210831-1812-B500_B.14-10.10.fid
Ref 659-1
Group Greaney_M
H1_Night CDCl3 /mnt/nmrdata/Greaney_M m31962tw 10

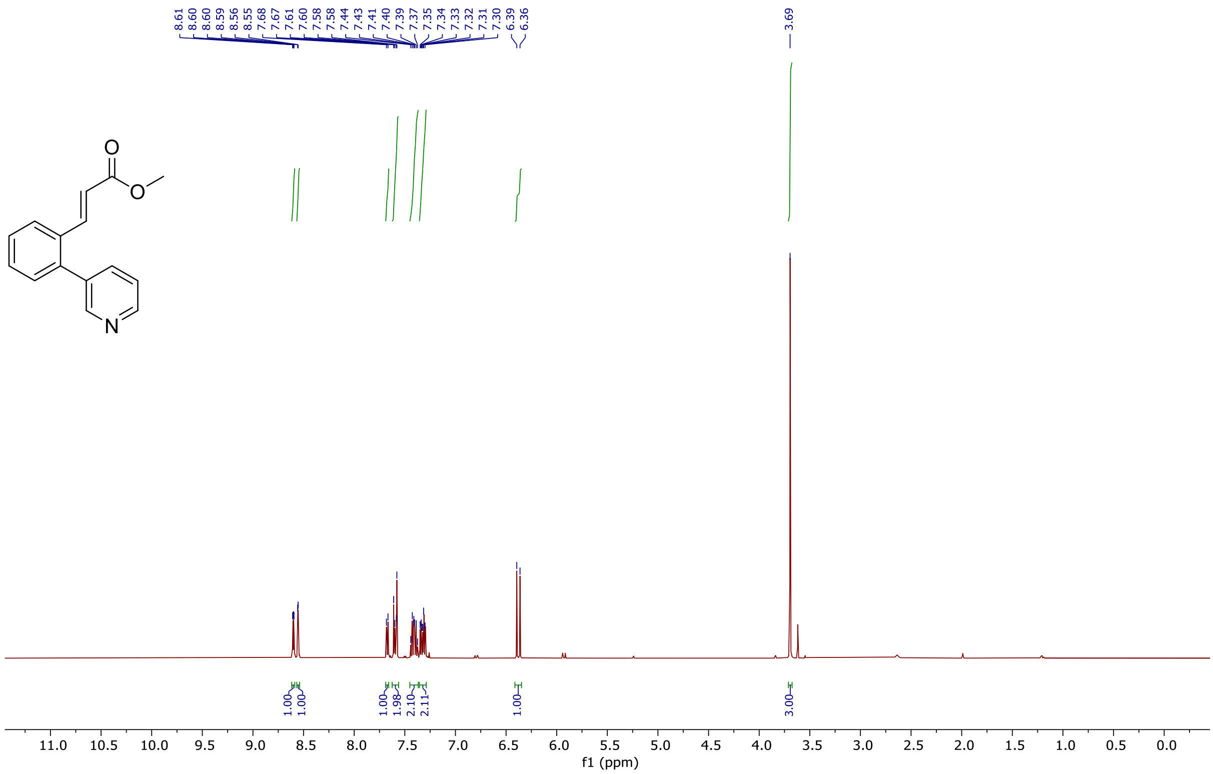

20210831-1812-B500_B.14-10.11.fid
Ref 659-1
Group Greaney_M
C13_CPD_Night256 CDCl3 /mnt/nmrdata/Greaney_M m31962tw 10

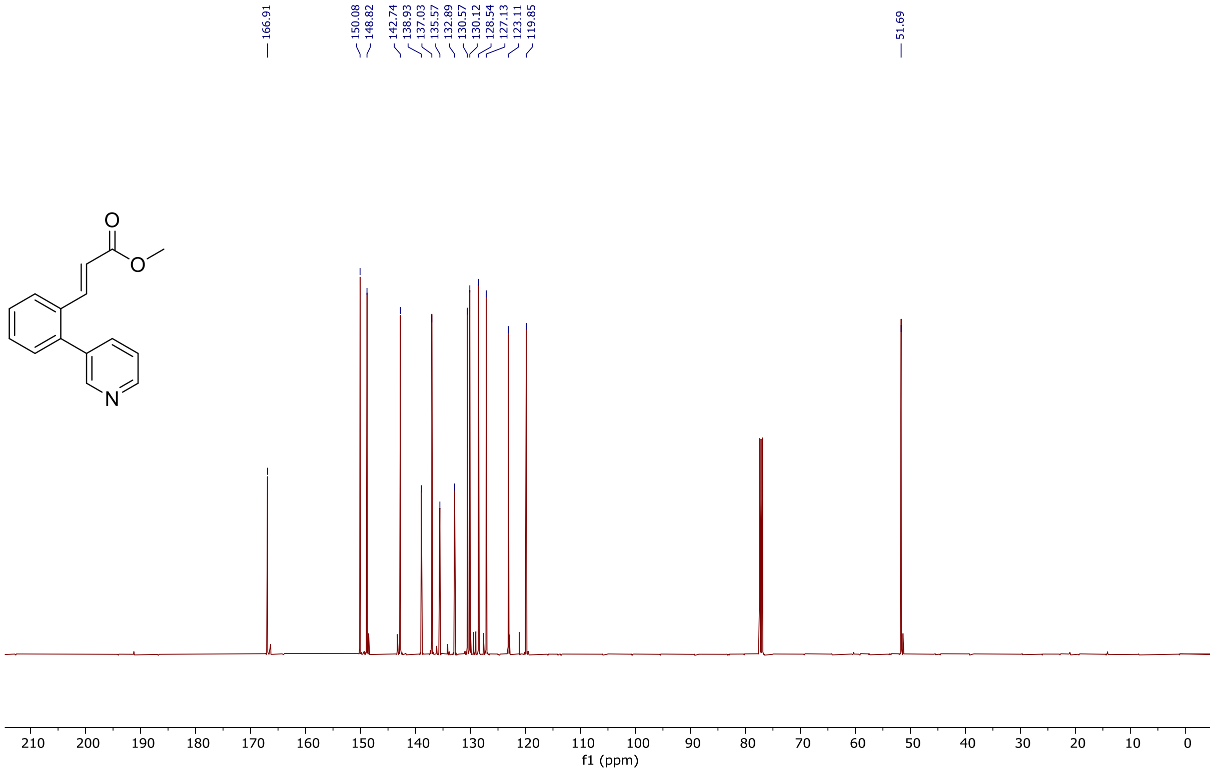

22a’

20211124-1554-B400_B.14-13.10.fid
Ref 706-3
Group Greaney_M
H1_Night CDCl3 /mnt/nmrdata/Greaney_M m31962tw 13

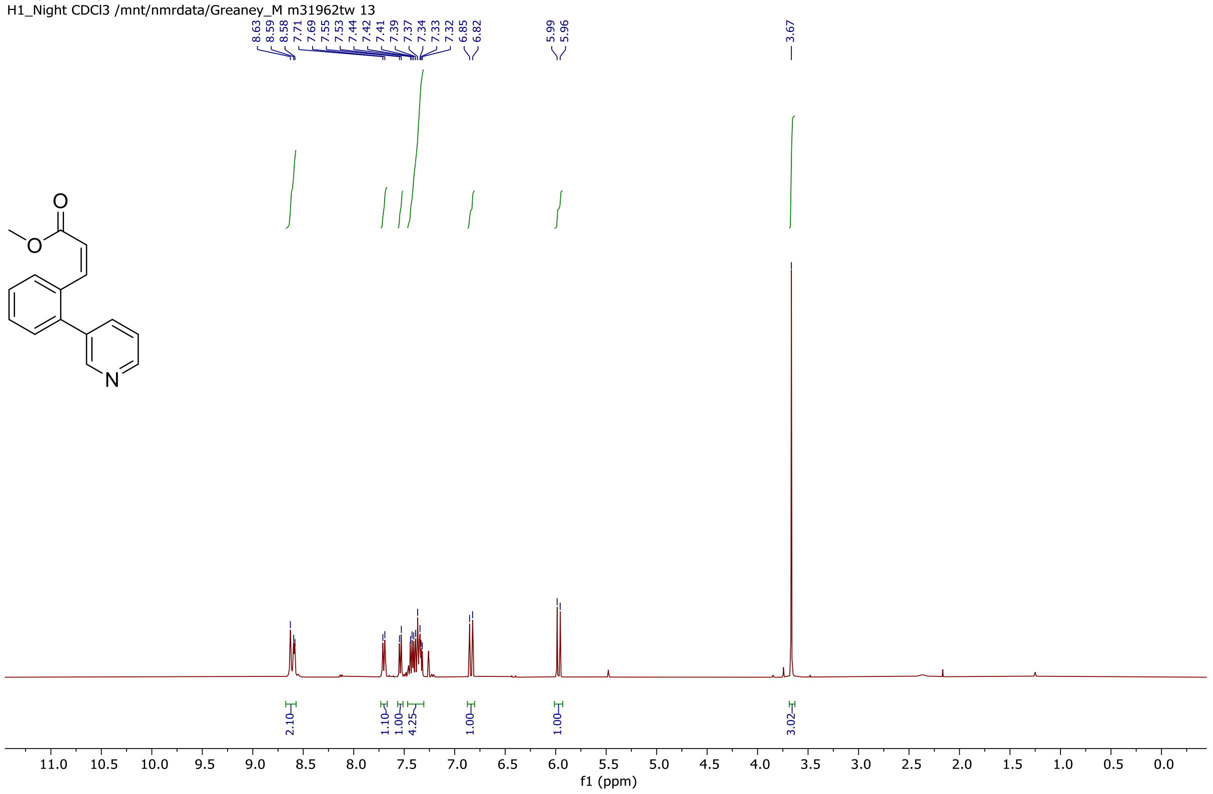

20211124-1554-B400_B.14-13.11.fid
Ref 706-3
Group Greaney_M
C13_CPD_Night256 CDCl3 /mnt/nmrdata/Greaney_M m31962tw 13

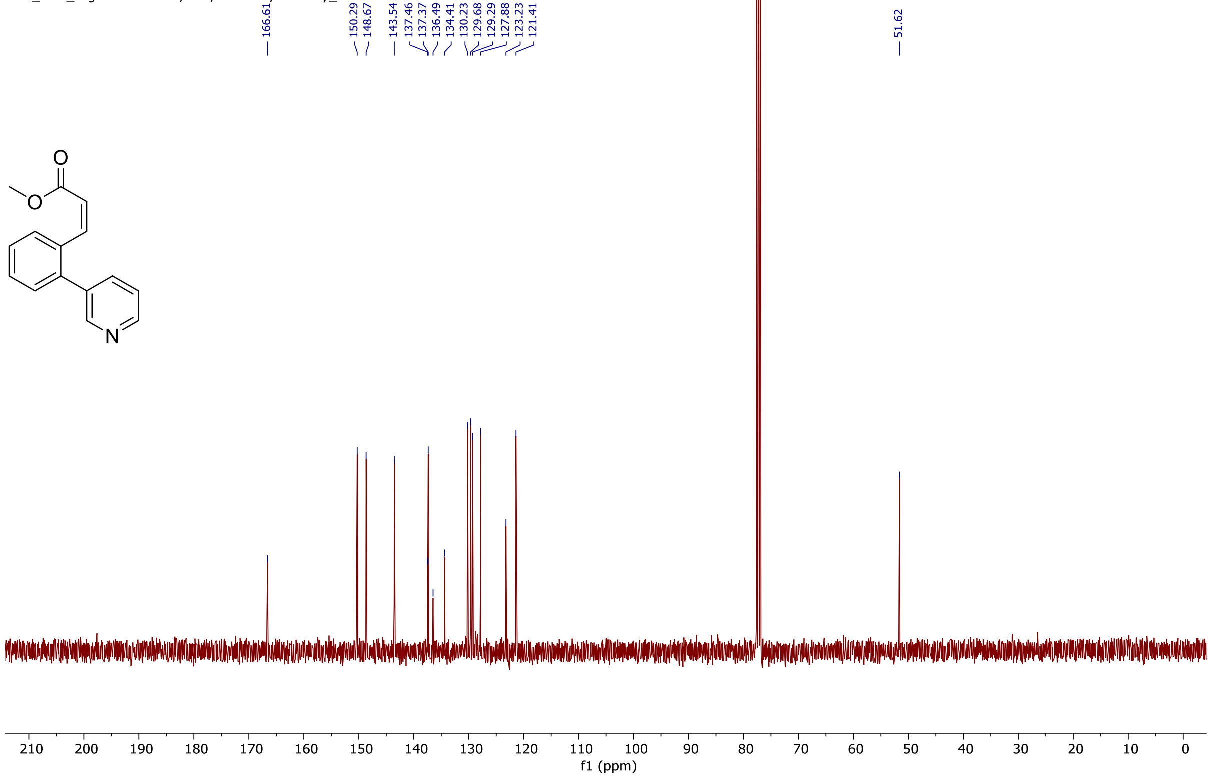

22g

20201202-1748-B400_B.12-35.10.fid
Ref 479-4
Group Greaney_M
H1_Night CDCl3 /mnt/nmrdata/Greaney_M m31962tw 35

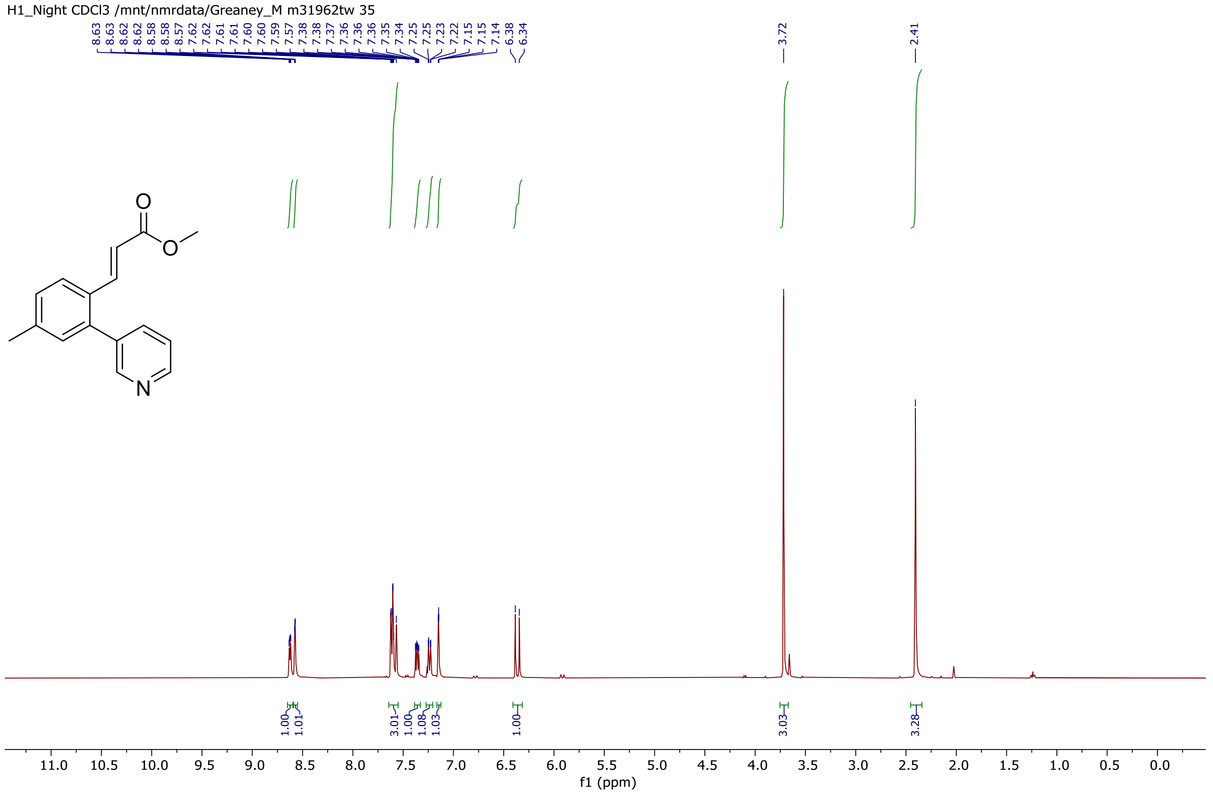

20201202-1748-B400_B.12-35.11.fid
Ref 479-4
Group Greaney_M
C13_CPD_Night256 CDCl3 /mnt/nmrdata/Greaney_M m31962tw 35

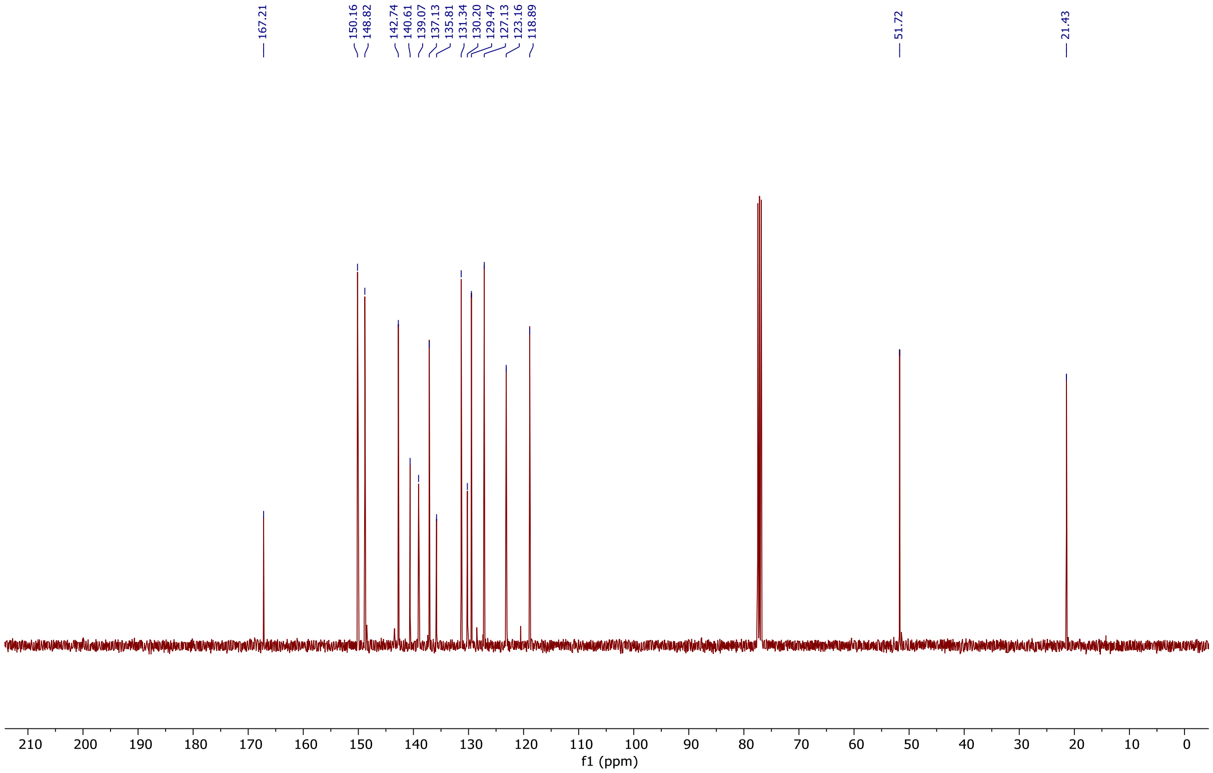

22h

20201202-1748-B400_B.12-34.10.fid
Ref 479-2
Group Greaney_M
H1_Night CDCl3 /mnt/nmrdata/Greaney_M m31962tw 34

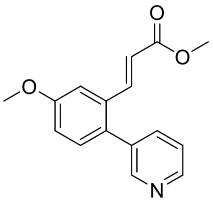

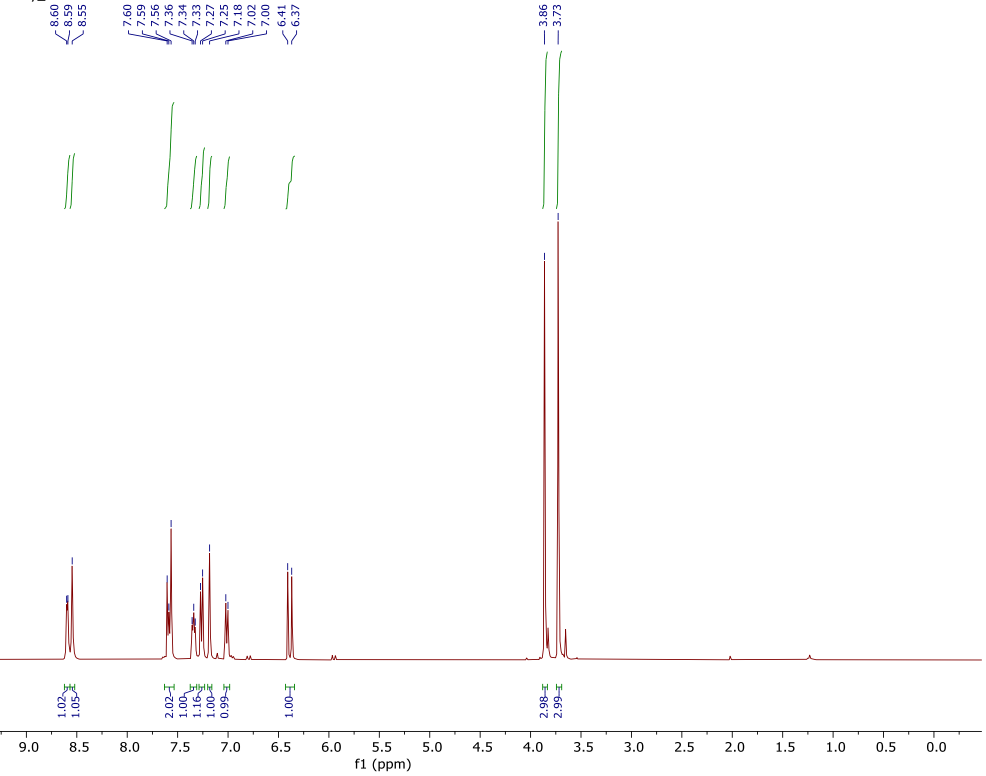

20201202-1748-B400_B.12-34.11.fid
Ref 479-2
Group Greaney_M
C13_CPD_Night256 CDCl3 /mnt/nmrdata/Greaney_M m31962tw 34

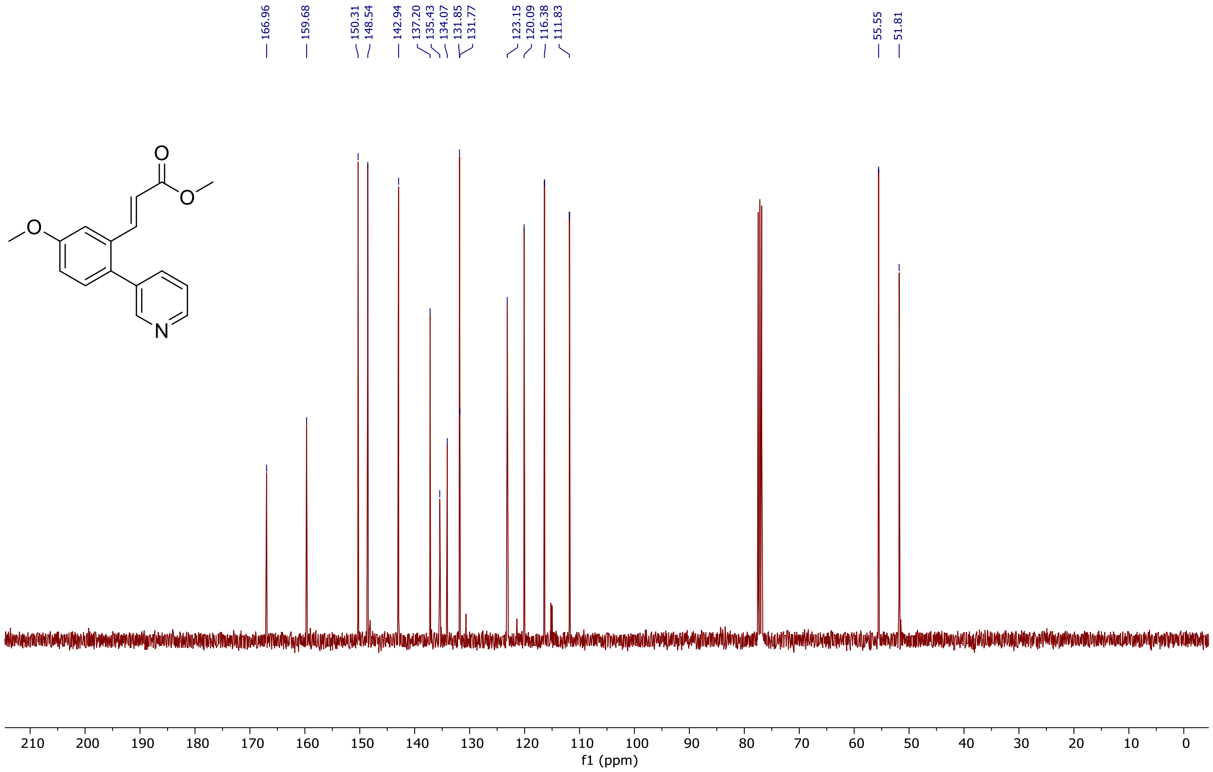

20201202-1729-B400_B.12-25.10.fid
Ref 479-6
Group Greaney_M
H1_Night CDCl3 /mnt/nmrdata/Greaney_M m31962tw 25

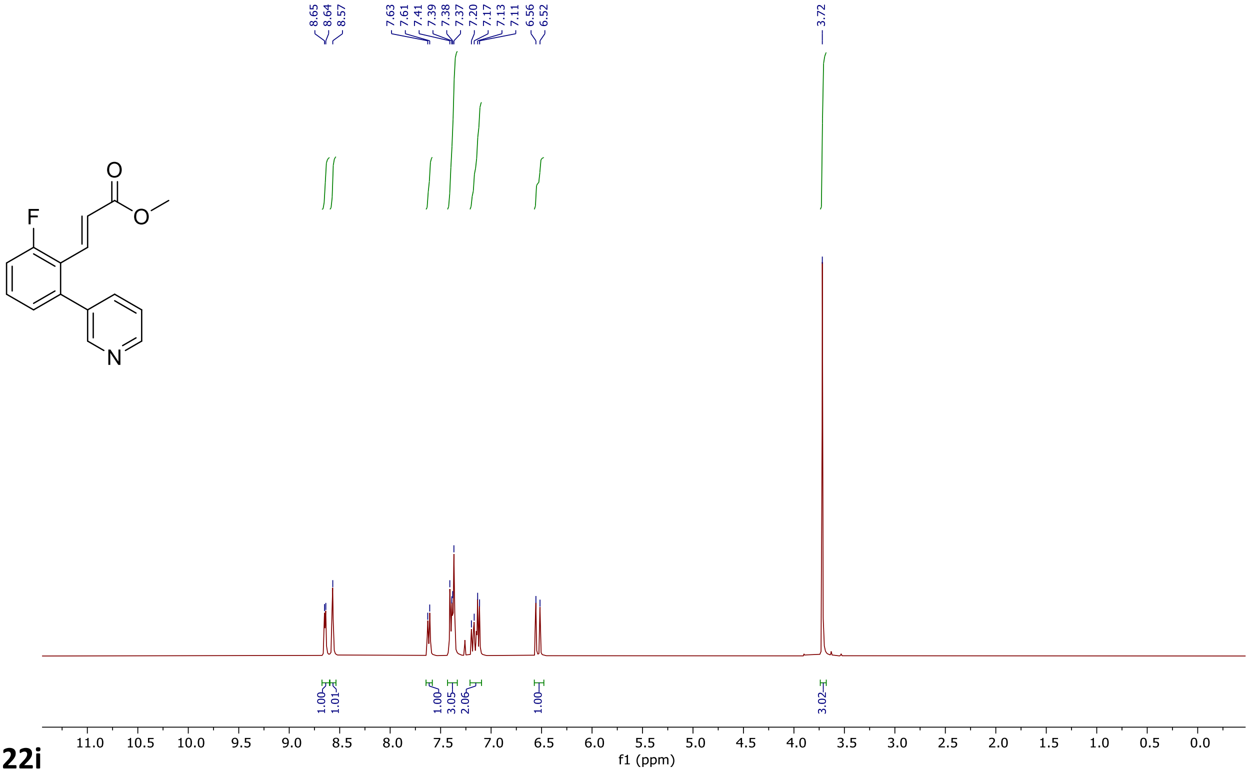

22i

20201202-1729-B400_B.12-25.11.fid
Ref 479-6
Group Greaney_M
C13_CPD_Night256 CDCl3 /mnt/nmrdata/Greaney_M m31962tw 25

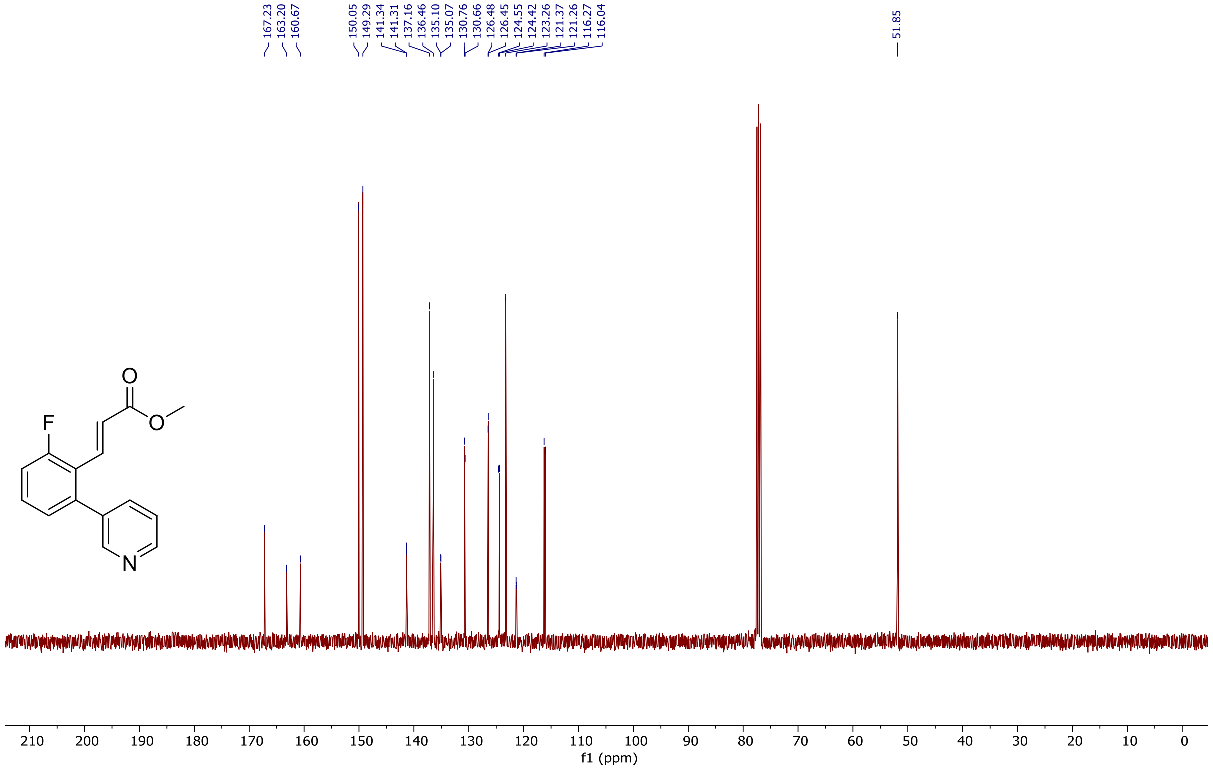

20201202-1729-B400_B.12-25.14.fid
Ref 479-6
Group Greaney_M
F19_CPD_Day CDCl3 /mnt/nmrdata/Greaney_M m31962tw 25

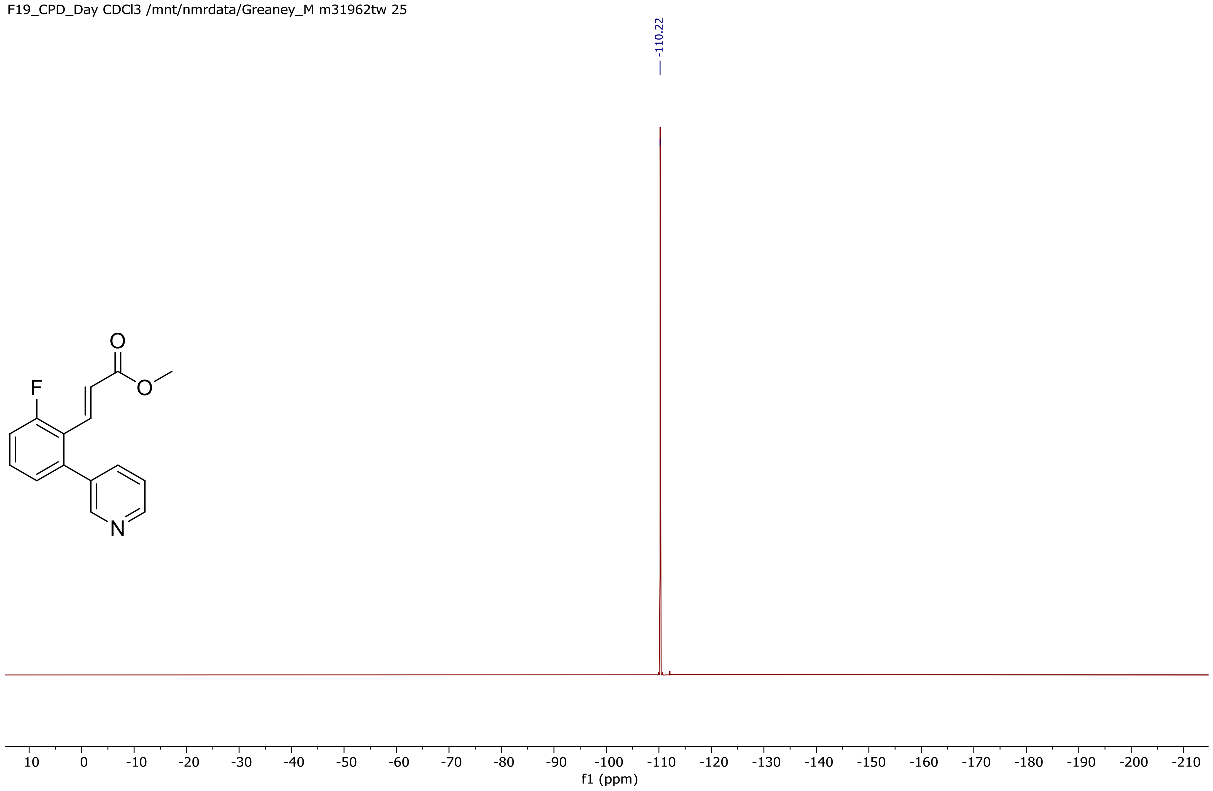

22j

20201215-1540-B400_B.11-22.10.fid
Ref 479-7
Group Greaney_M
H1_Night CDCl3 /mnt/nmrdata/Greaney_M m31962tw 22

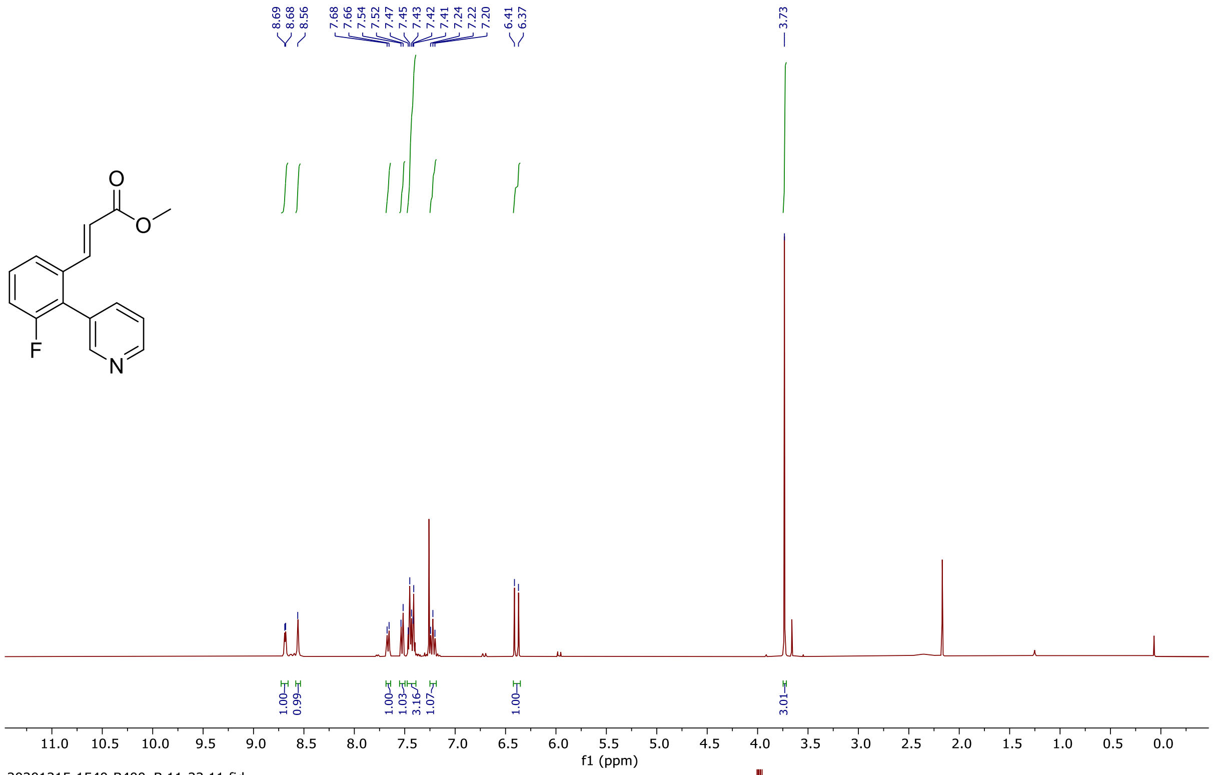

20201215-1540-B400_B.11-22.11.fid
Ref 479-7
Group Greaney_M
C13_CPD_Night256 CDCl3 /mnt/nmrdata/Greaney_M m31962tw 22

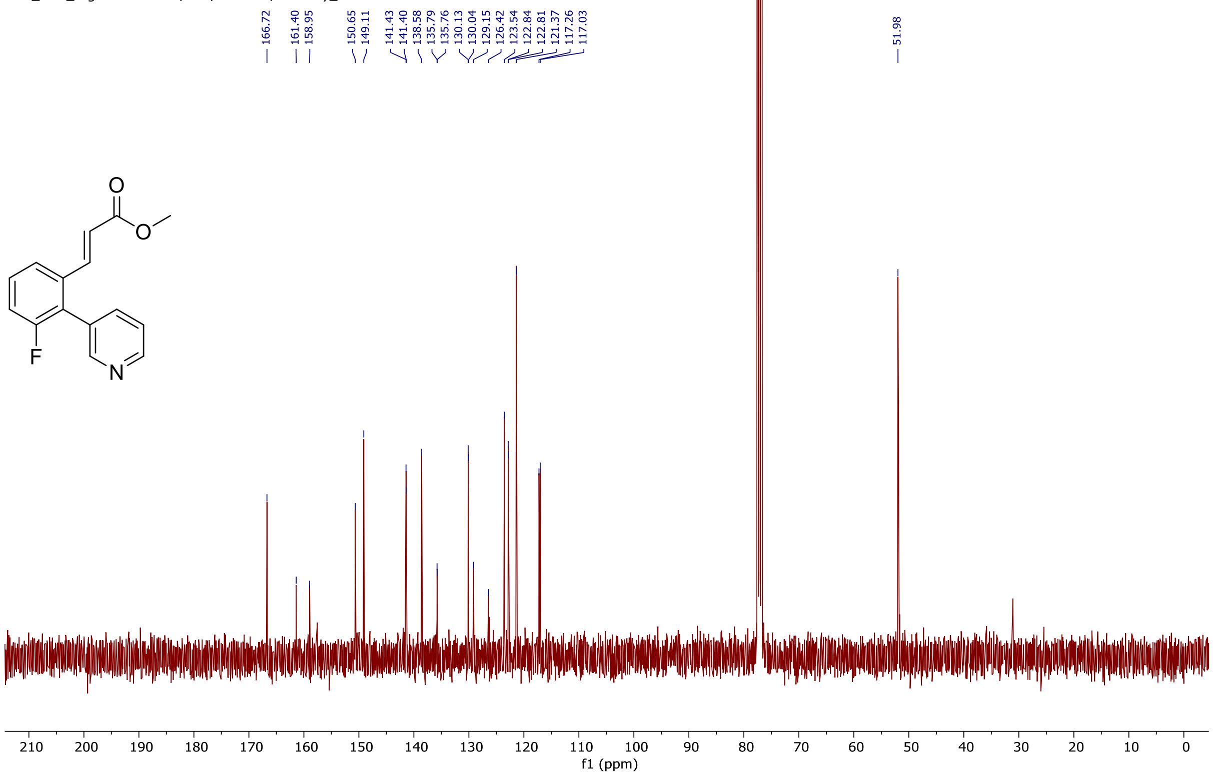

20201215-1540-B400_B.11-22.12.fid
Ref 479-7
Group Greaney_M
F19_NoCPD_Night CDCl3 /mnt/nmrdata/Greaney_M m31962tw 22

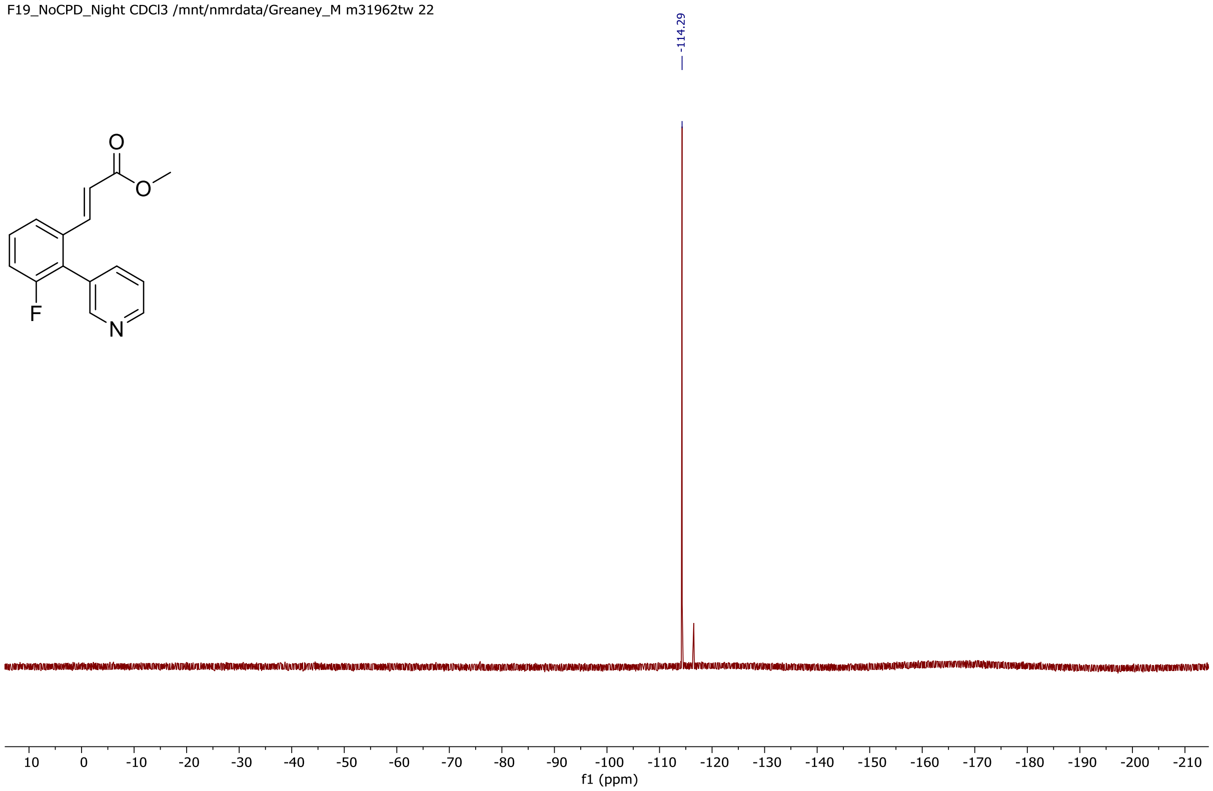

22k

20201202-1747-B400_B.12-32.10.fid
Ref 479-1
Group Greaney_M
H1_Night CDCl3 /mnt/nmrdata/Greaney_M m31962tw 32

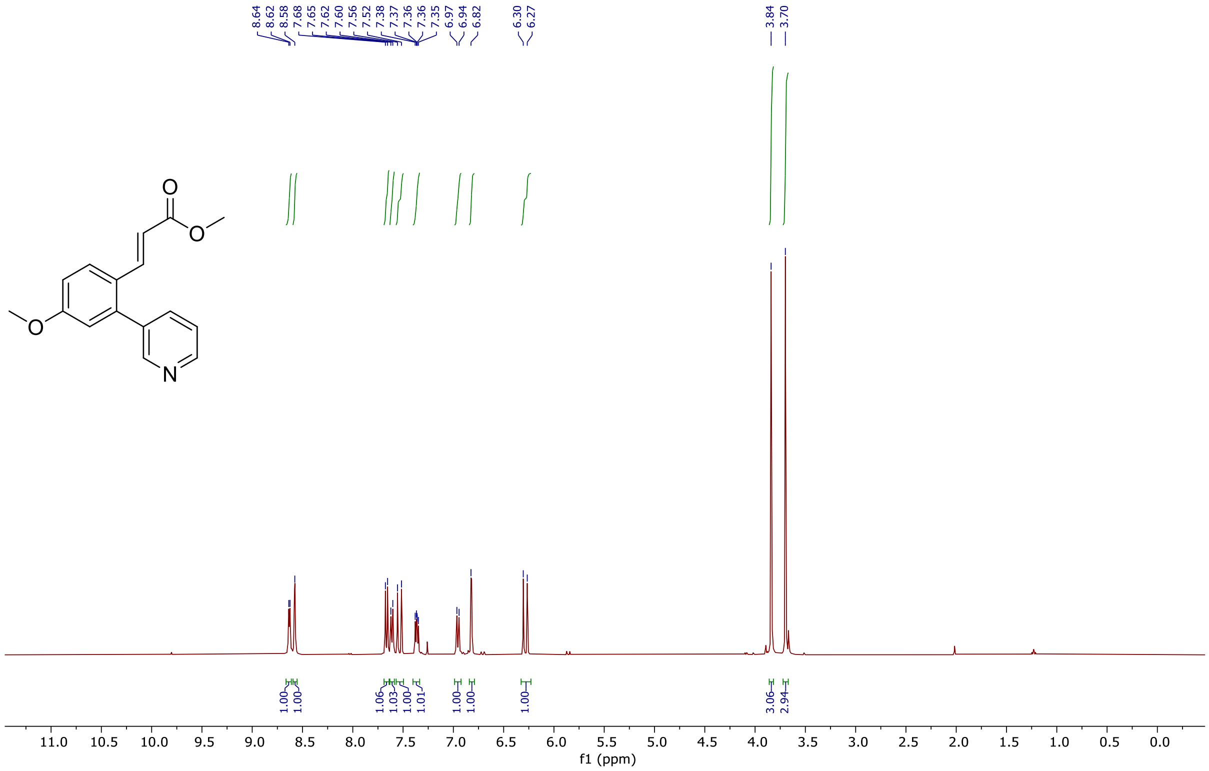

20201202-1747-B400_B.12-32.11.fid
Ref 479-1
Group Greaney_M
C13_CPD_Night256 CDCl3 /mnt/nmrdata/Greaney_M m31962tw 32

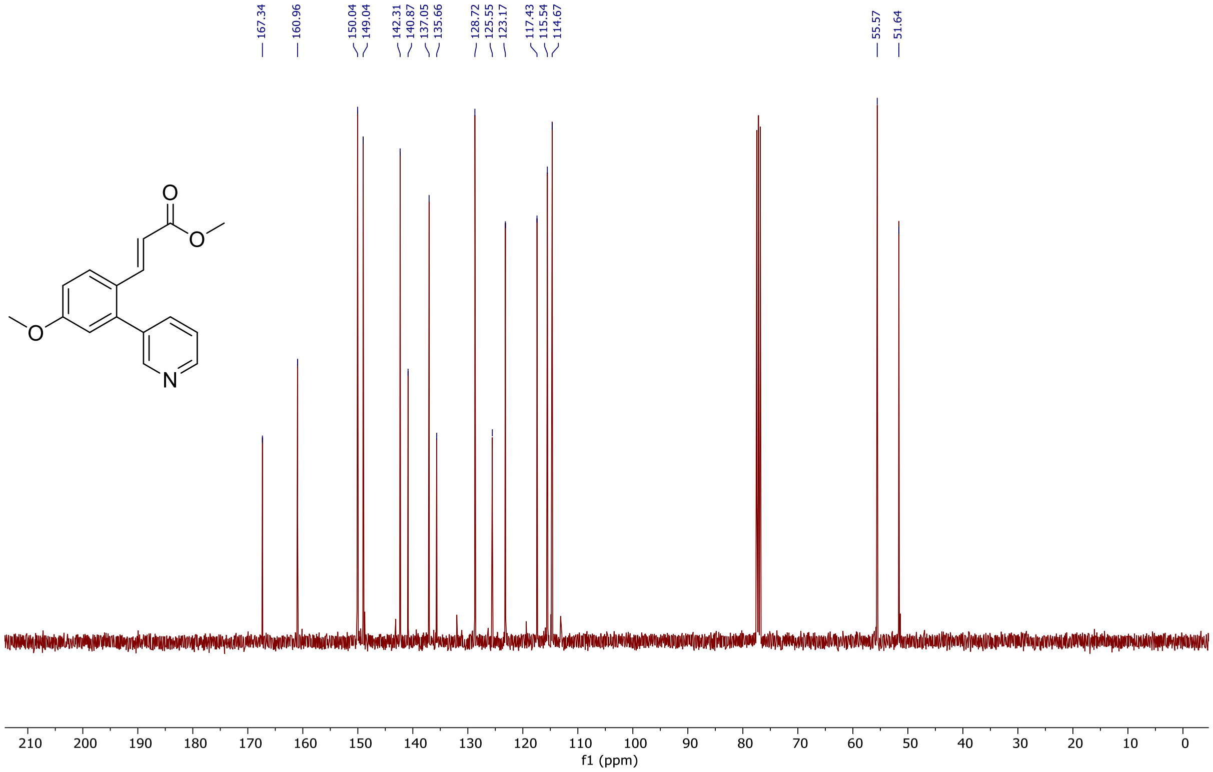

22m

20201215-1539-B400_B.11-20.10.fid
Ref 479-5
Group Greaney_M
H1_Night CDCl3 /mnt/nmrdata/Greaney_M m31962tw 20

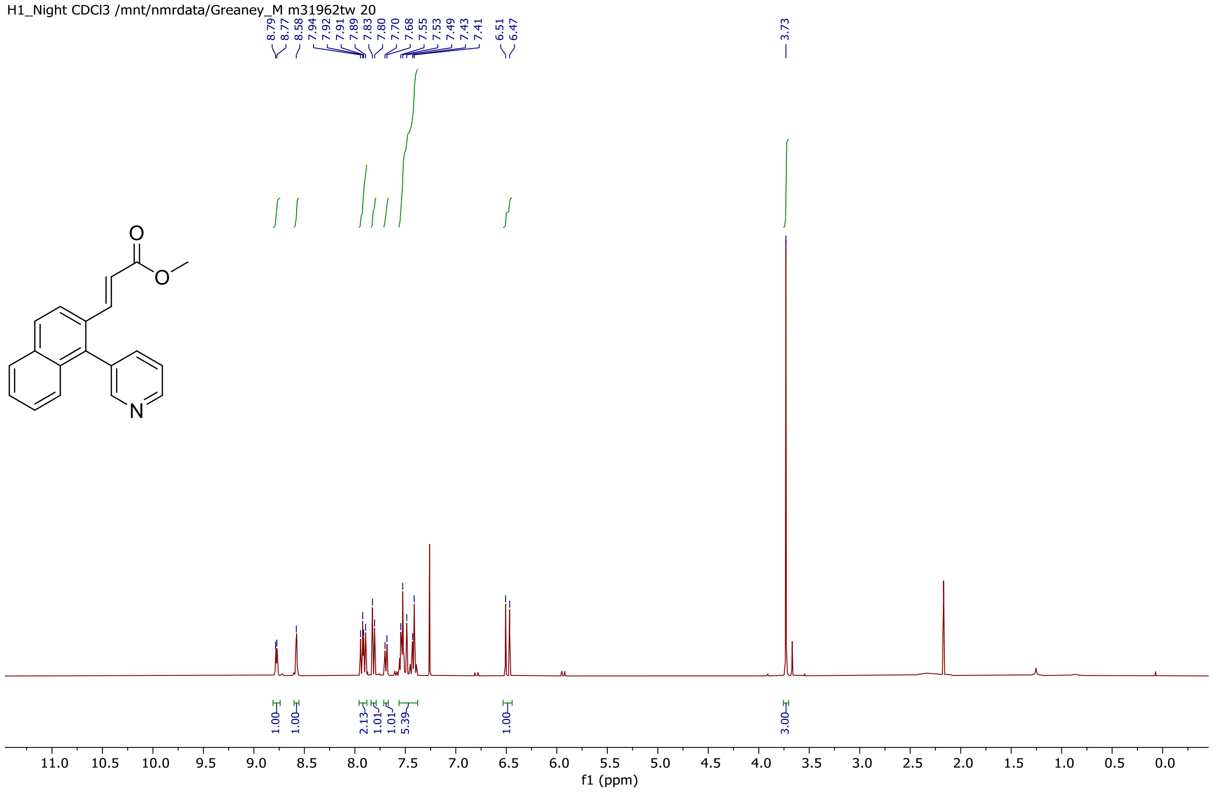

20201202-1729-B400_B.12-24.12.fid
Ref 479-5
Group Greaney_M
C13_CPD_Night256 CDCl3 /mnt/nmrdata/Greaney_M m31962tw 24

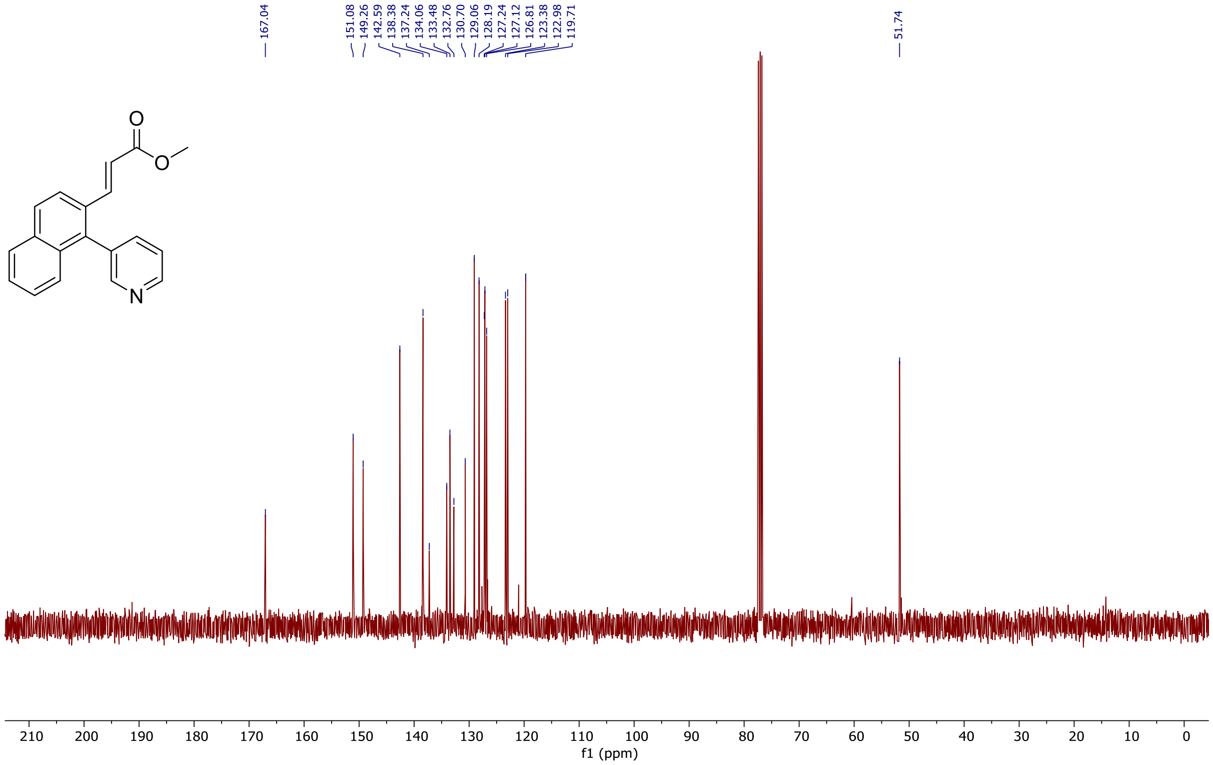

22n

20210416-0958-B400_B.11-13.10.fid
Ref 536-6
Group Greaney_M
H1_Night CDCl3 /mnt/nmrdata/Greaney_M m31962tw 13

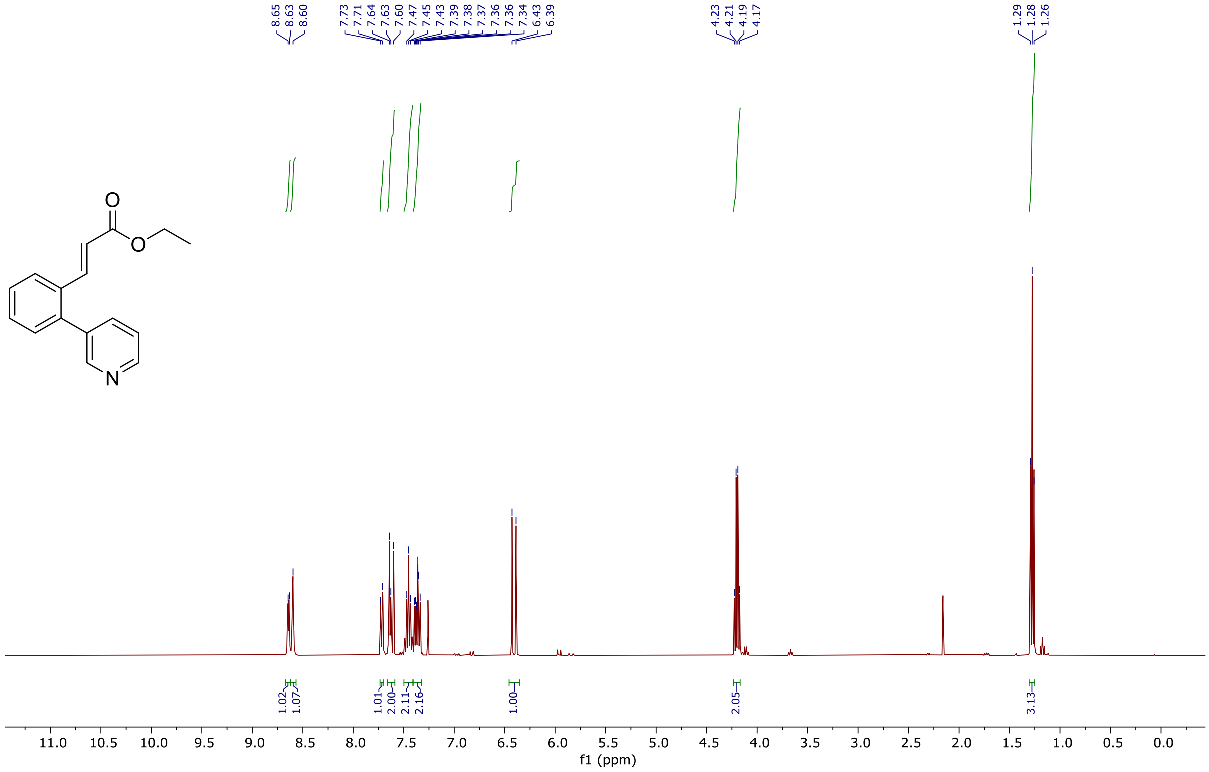

20210416-0958-B400_B.11-13.11.fid
Ref 536-6
Group Greaney_M
C13_CPD_Night256 CDCl3 /mnt/nmrdata/Greaney_M m31962tw 13

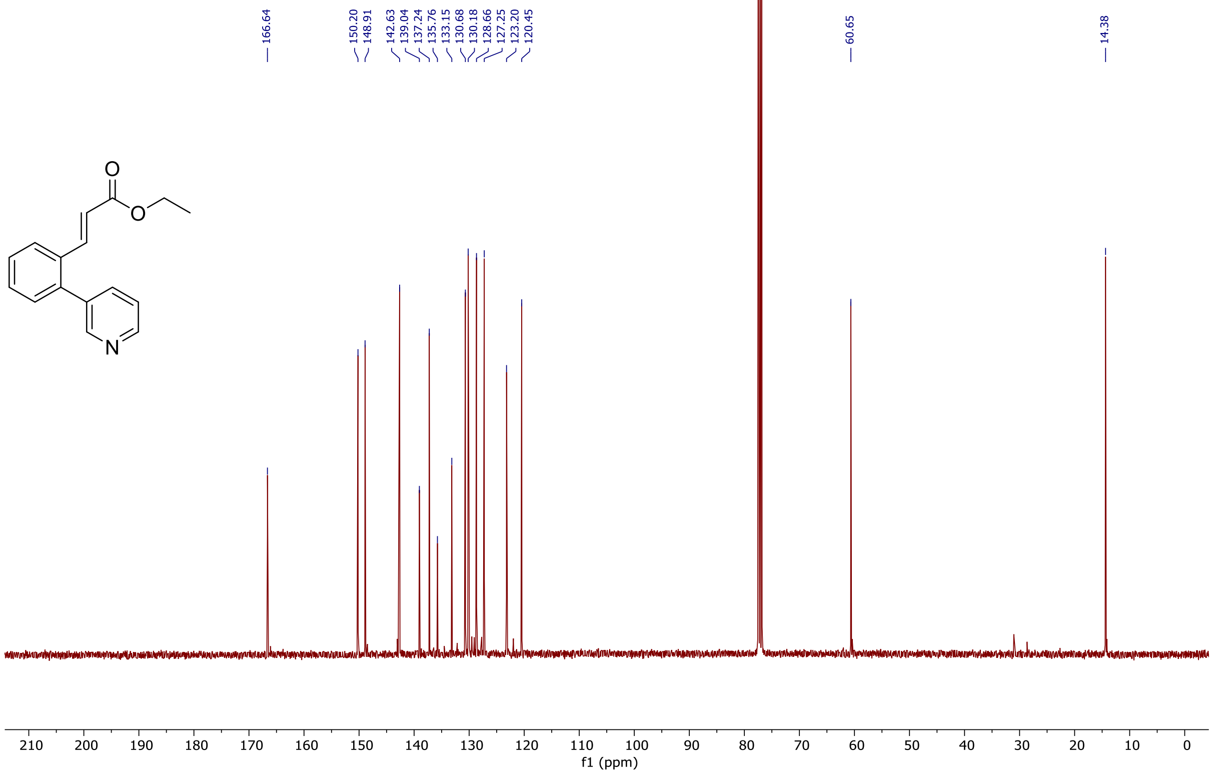

22o

20210408-1430-B400_B.11-3.10.fid
Ref 536-1
Group Greaney_M
H1_Night CDCl3 /mnt/nmrdata/Greaney_M m31962tw 3

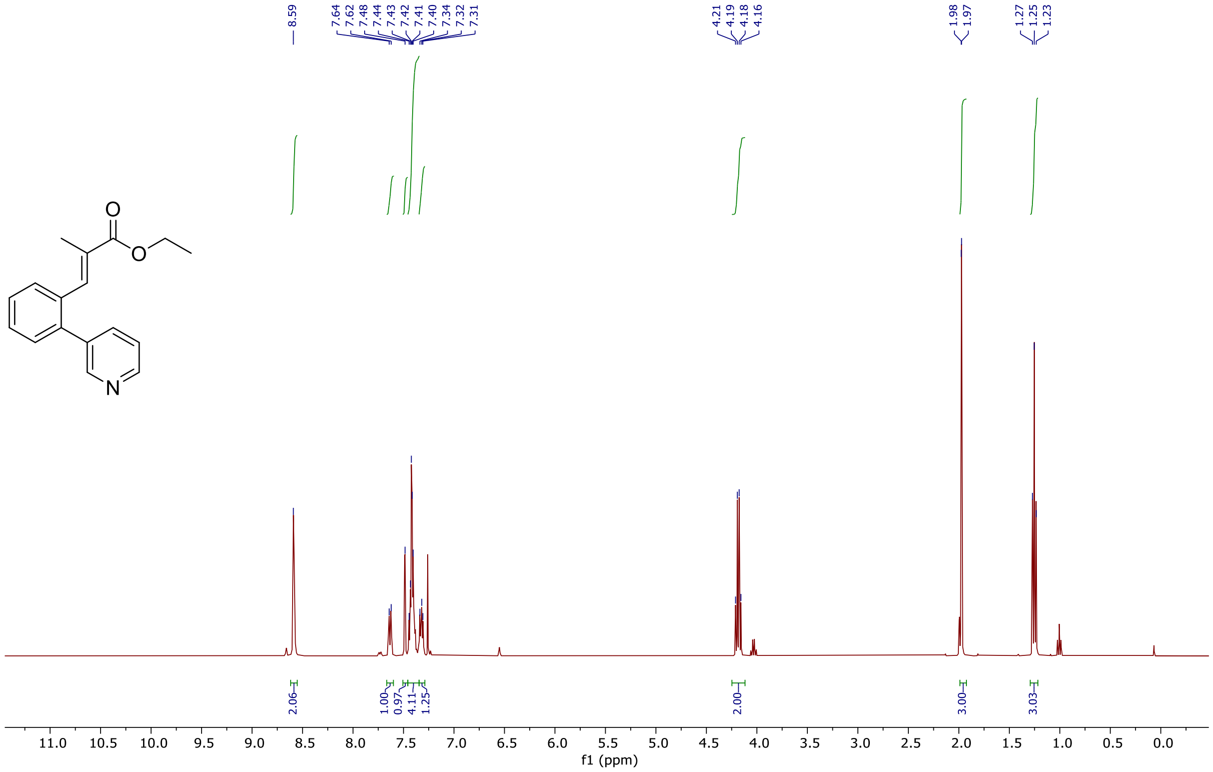

20210408-1430-B400_B.11-3.11.fid
Ref 536-1
Group Greaney_M
C13_CPD_Night256 CDCl3 /mnt/nmrdata/Greaney_M m31962tw 3

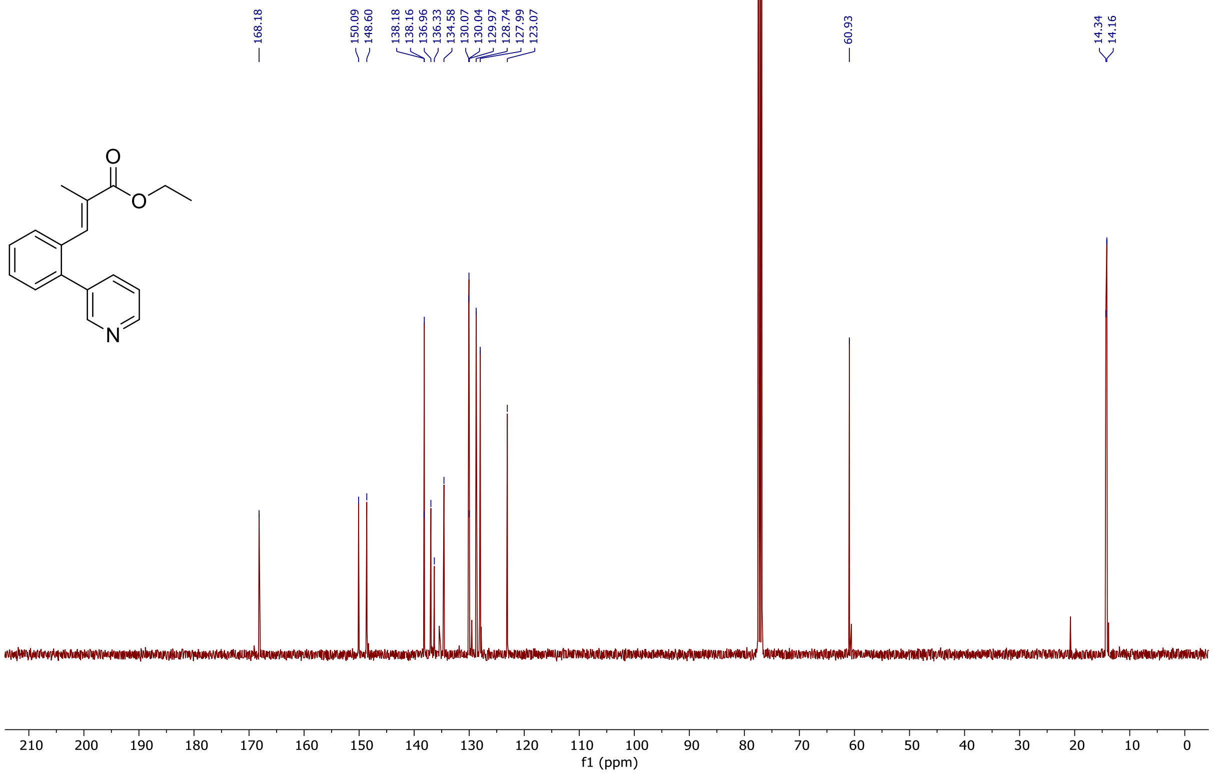

22p

20210408-1737-B400_B.11-46.10.fid
Ref 536-2
Group Greaney_M
H1_Night CDCl3 /mnt/nmrdata/Greaney_M m31962tw 46

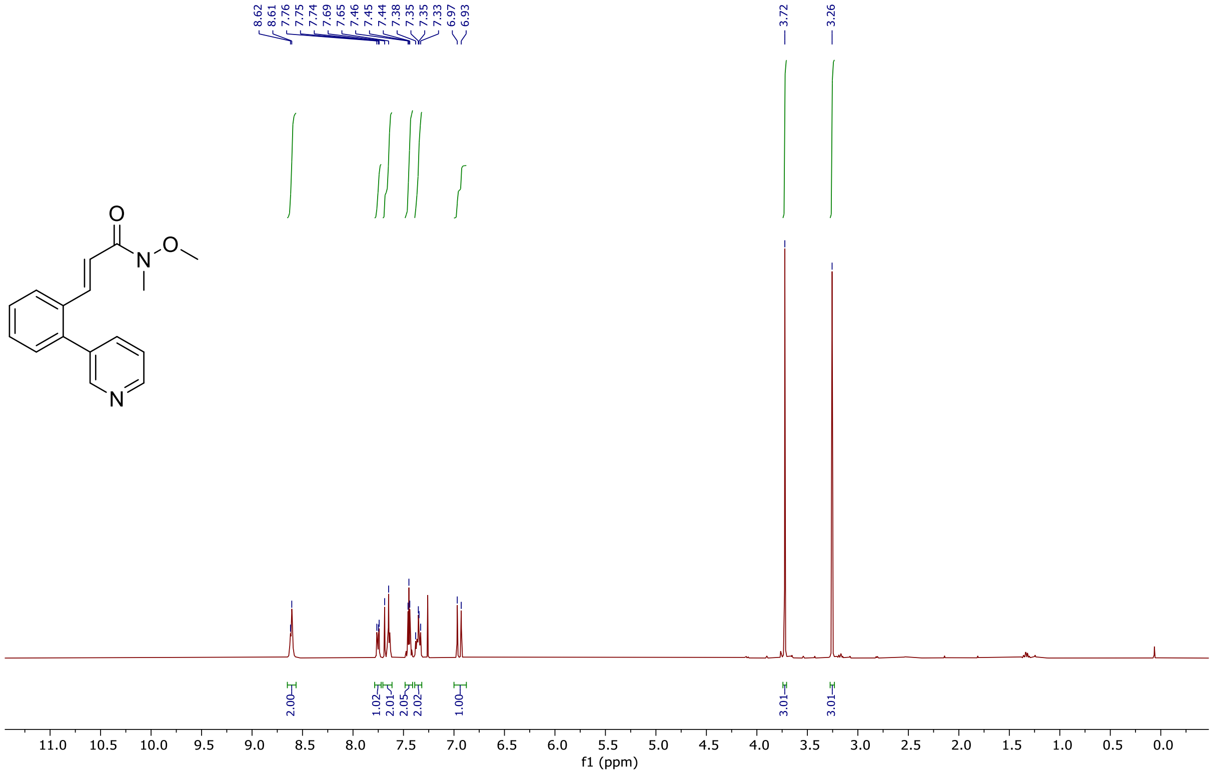

20210408-1737-B400_B.11-46.11.fid
Ref 536-2
Group Greaney_M
C13_CPD_Night256 CDCl3 /mnt/nmrdata/Greaney_M m31962tw 46

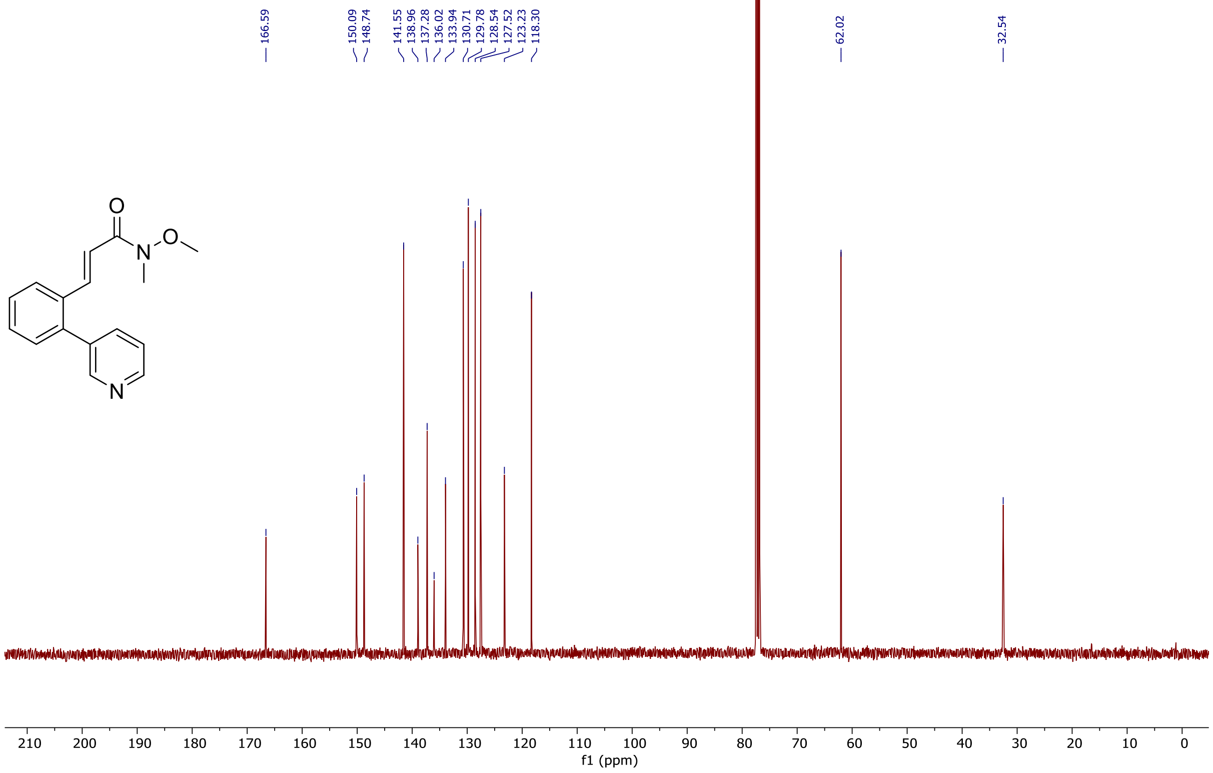

22q

20210409-1812-B500_B.14-4.10.fid
Ref 491-2
Group Greaney_M
H1_Night CDCl3 /mnt/nmrdata/Greaney_M m31962tw 4

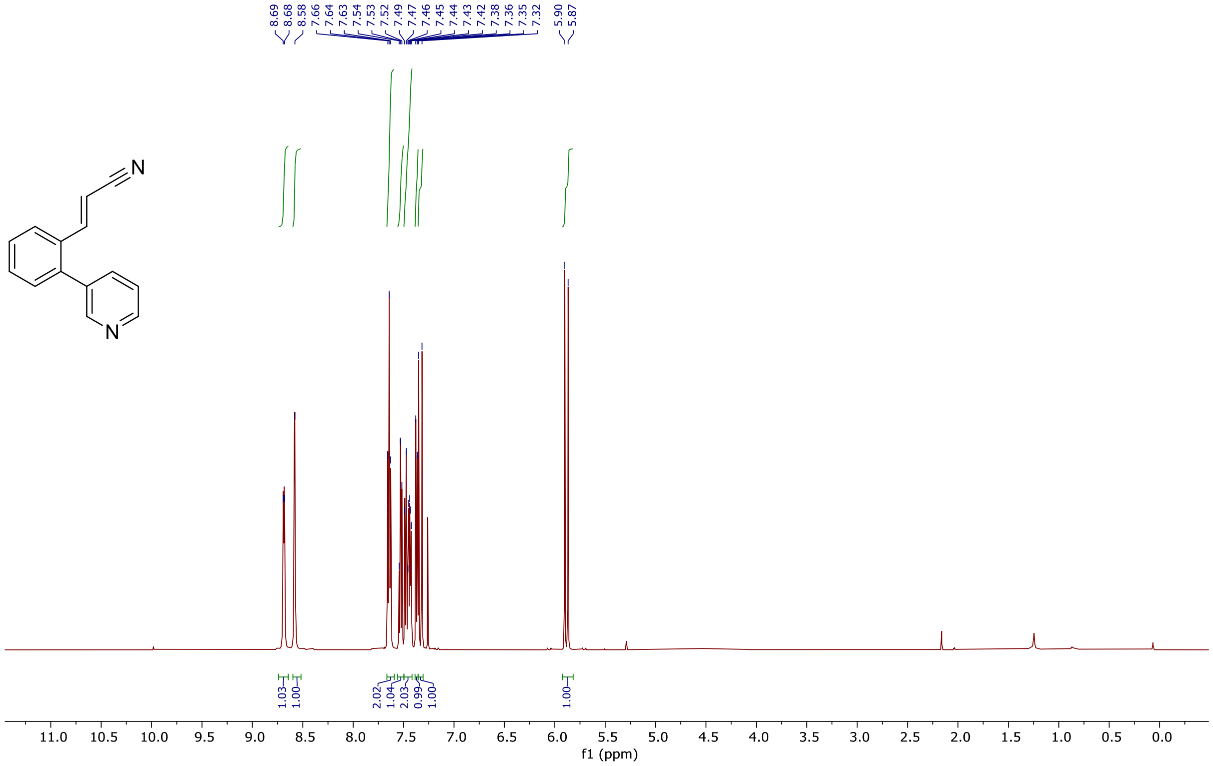

20210409-1812-B500_B.14-4.11.fid
Ref 491-2
Group Greaney_M
C13_CPD_Night256 CDCl3 /mnt/nmrdata/Greaney_M m31962tw 4

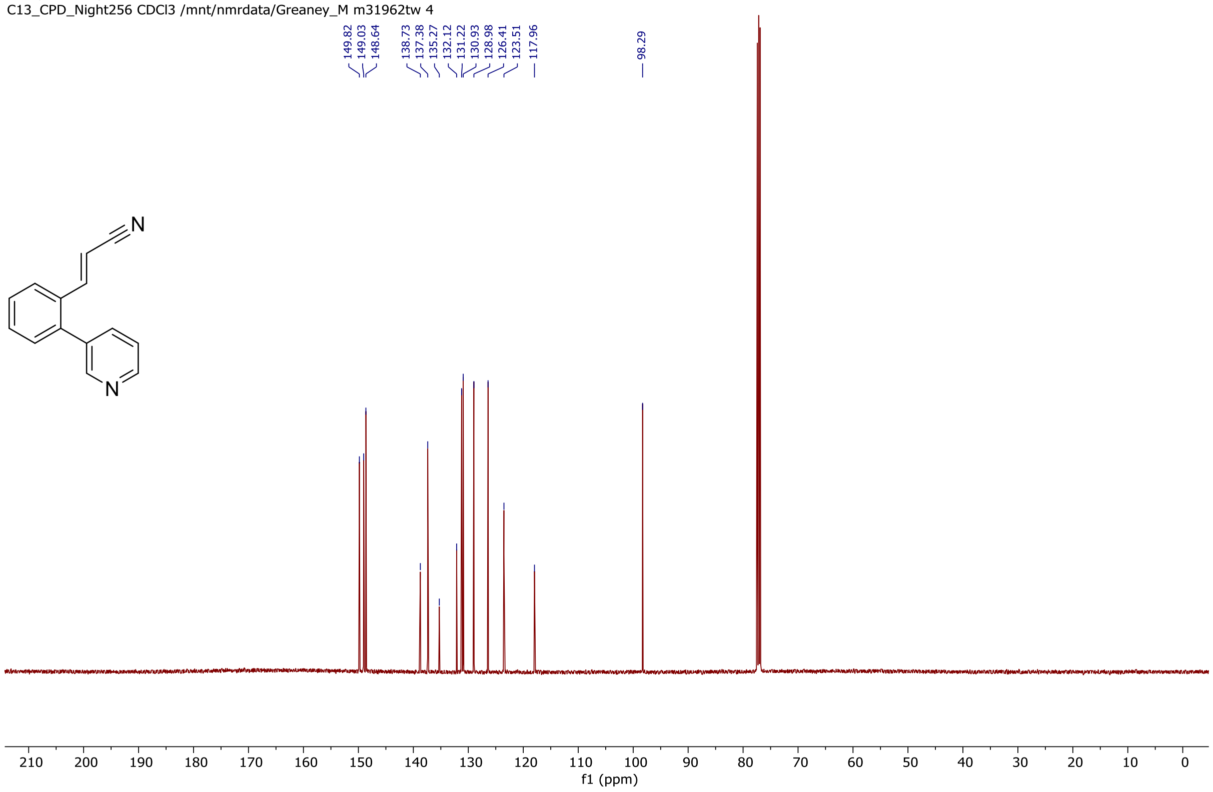

22r

20210407-1735-B400_B.11-22.10.fid
Ref 495-4
Group Greaney_M
H1_Night CDCl3 /mnt/nmrdata/Greaney_M m31962tw 22

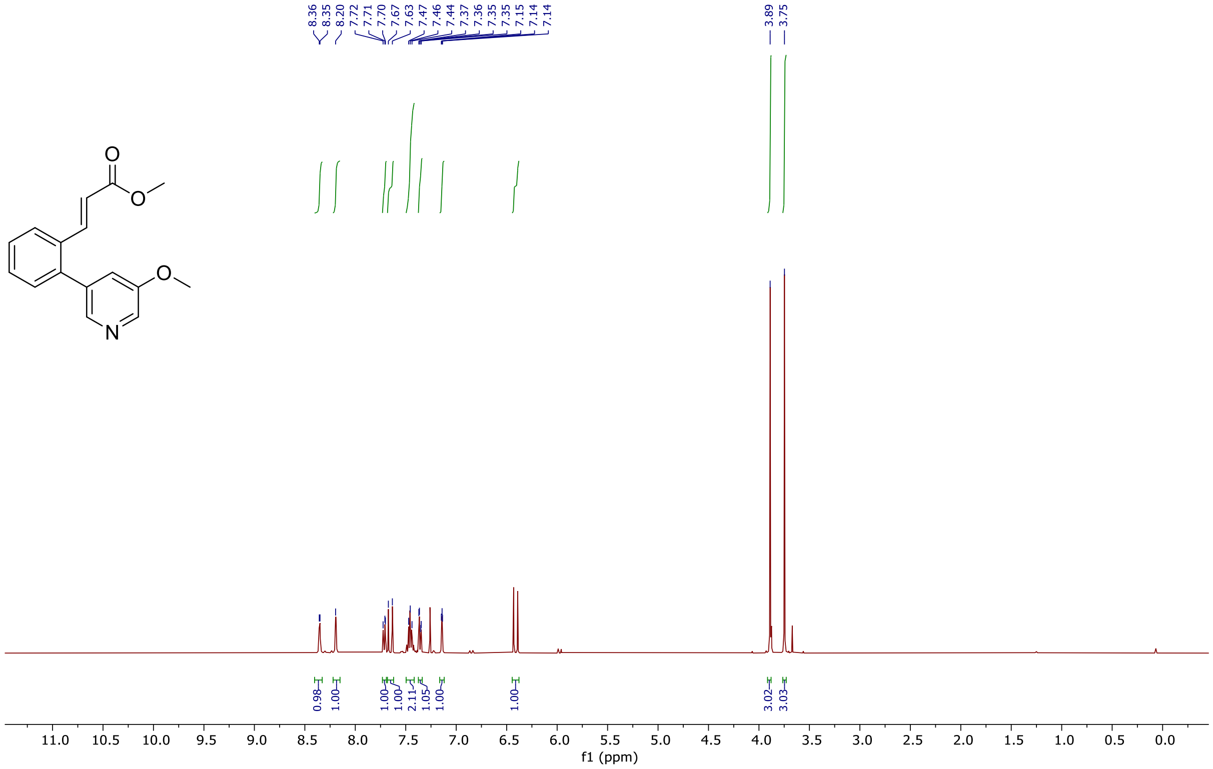

20210407-1735-B400_B.11-22.11.fid
Ref 495-4
Group Greaney_M
C13_CPD_Night256 CDCl3 /mnt/nmrdata/Greaney_M m31962tw 22

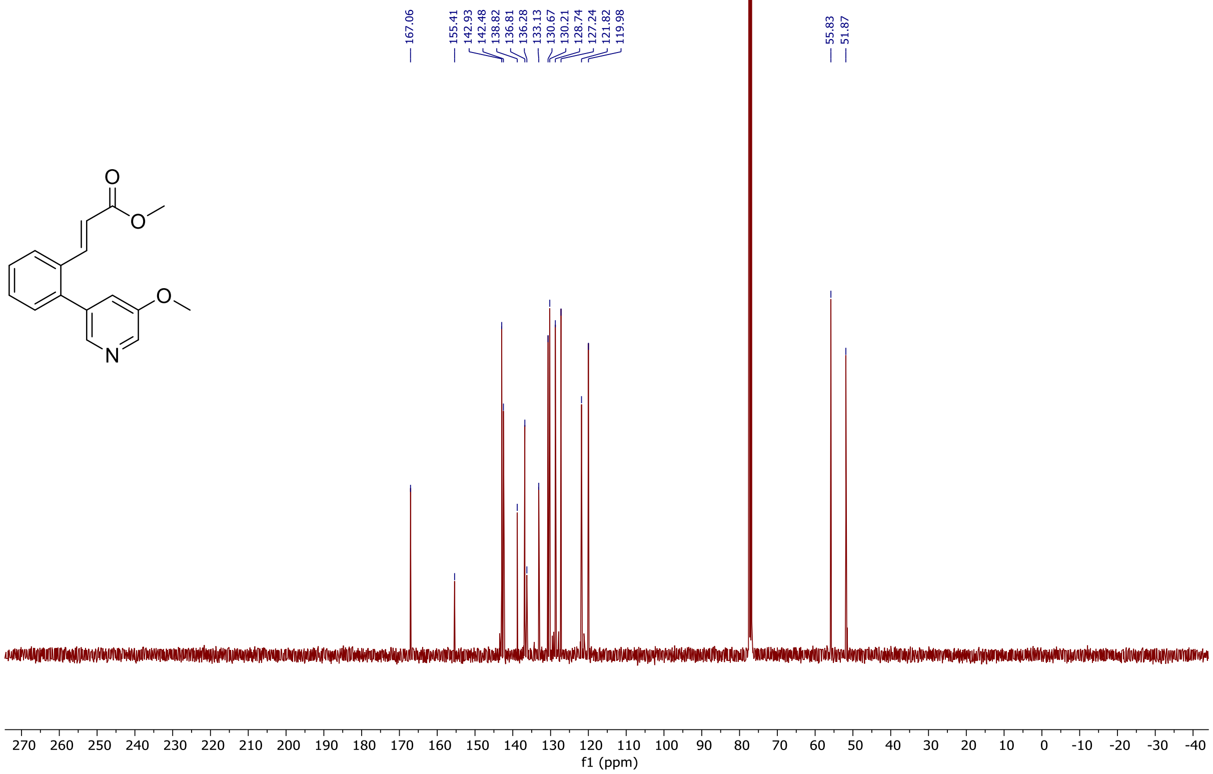

22s

20210408-1737-B400_B.11-44.10.fid
Ref 529-2
Group Greaney_M
H1_Night CDCl3 /mnt/nmrdata/Greaney_M m31962tw 44

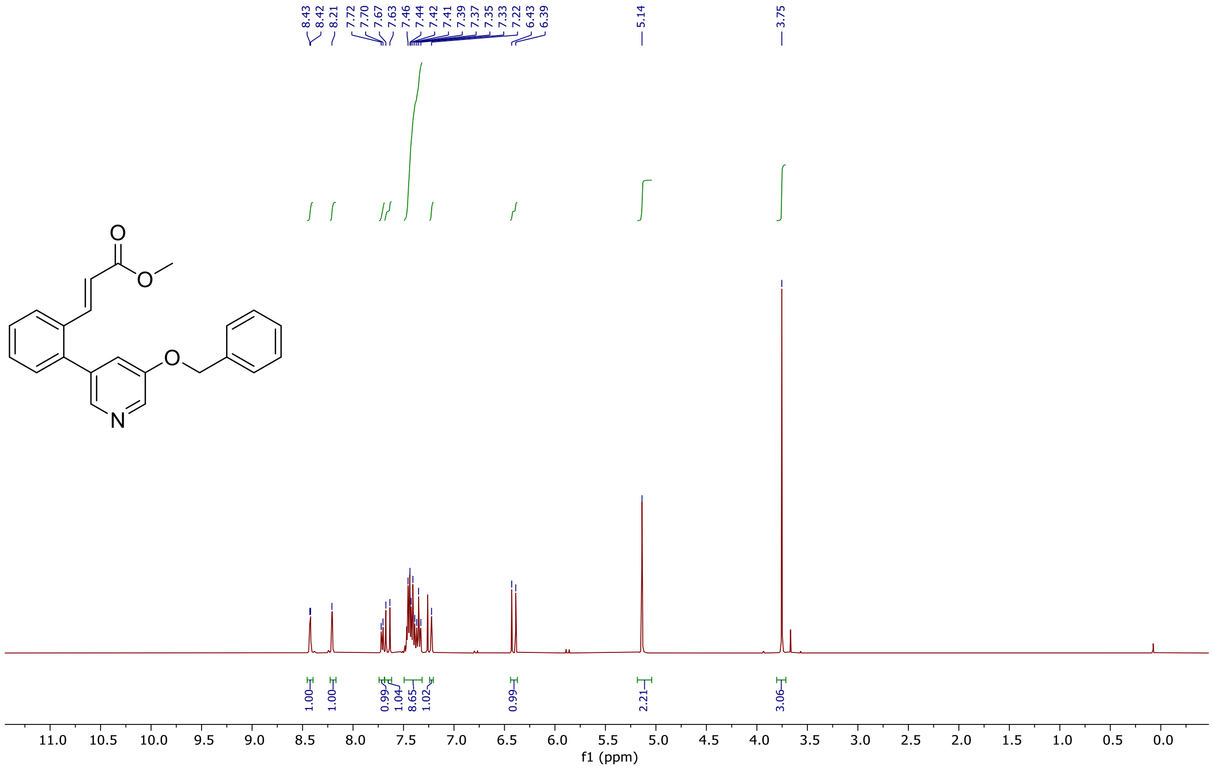

20210408-1737-B400_B.11-44.11.fid
Ref 529-2
Group Greaney_M
C13_CPD_Night256 CDCl3 /mnt/nmrdata/Greaney_M m31962tw 44

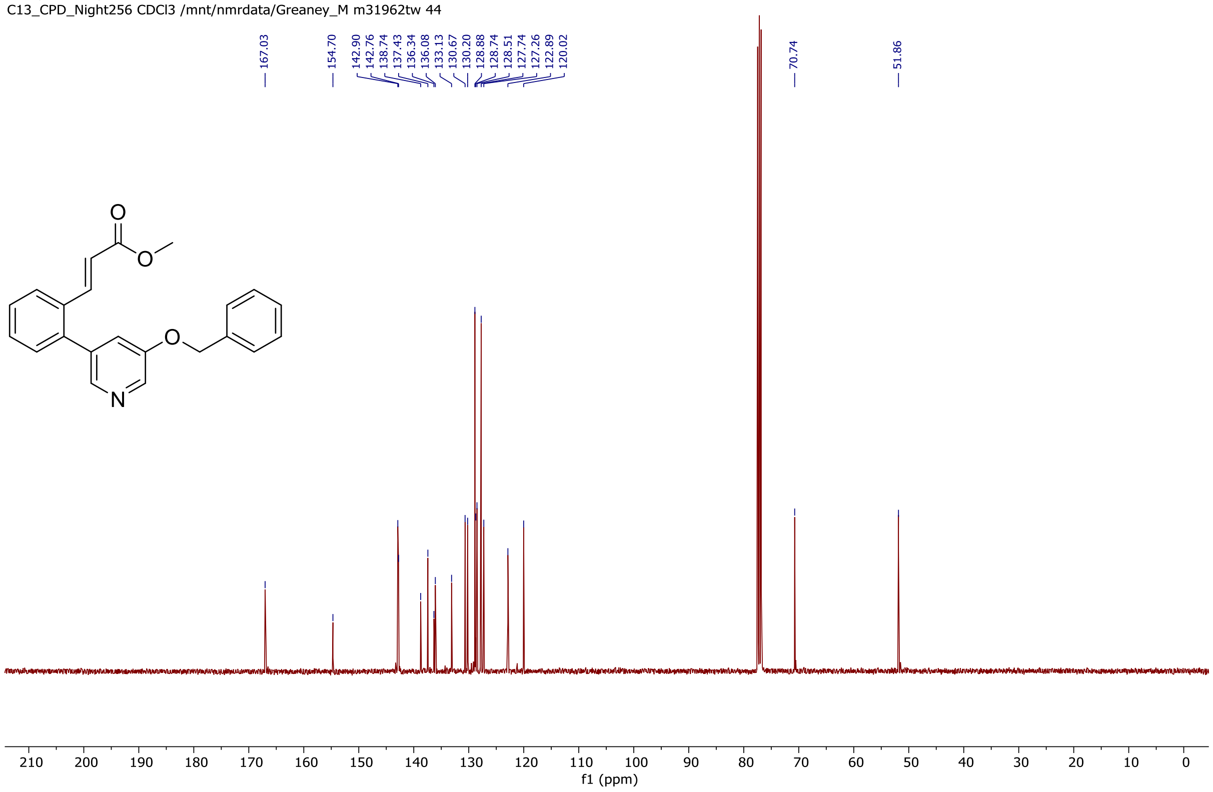

22t

20210408-1429-B400_B.11-2.10.fid
Ref 530-3
Group Greaney_M
H1_Night CDCl3 /mnt/nmrdata/Greaney_M m31962tw 2

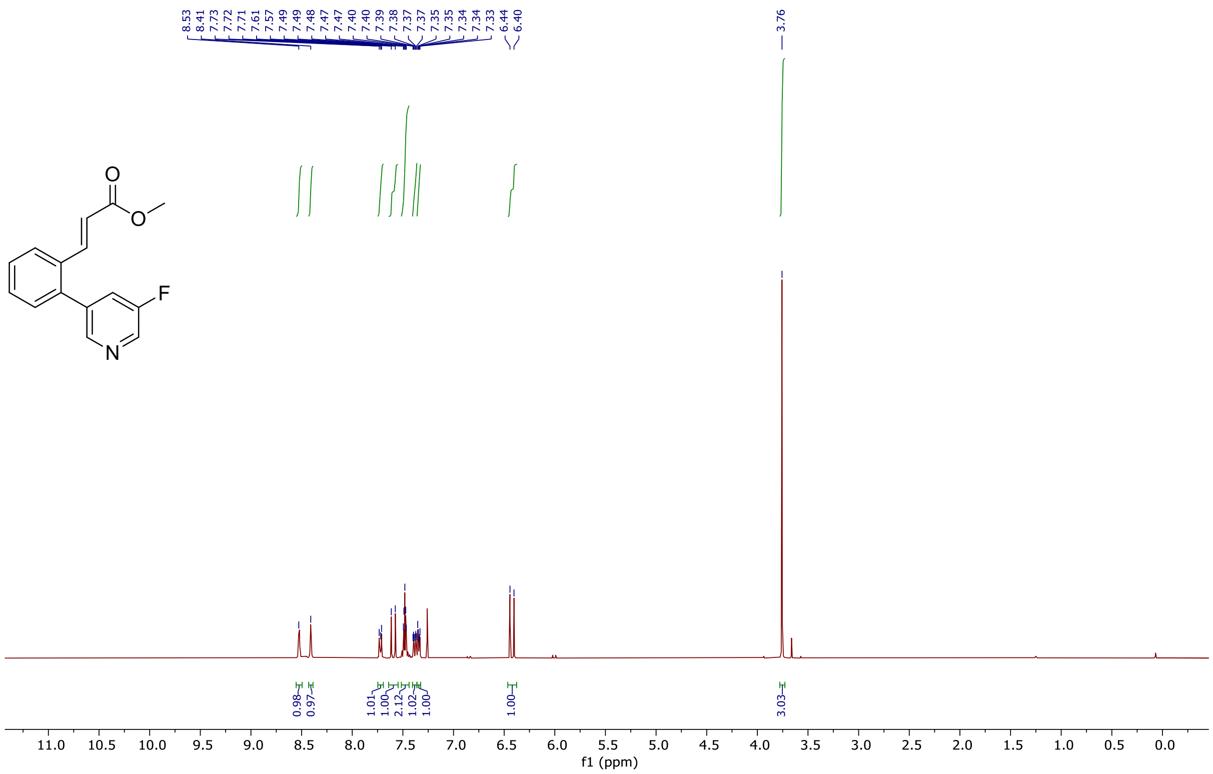

20210408-1429-B400_B.11-2.11.fid
Ref 530-3
Group Greaney_M
C13_CPD_Night256 CDCl3 /mnt/nmrdata/Greaney_M m31962tw 2

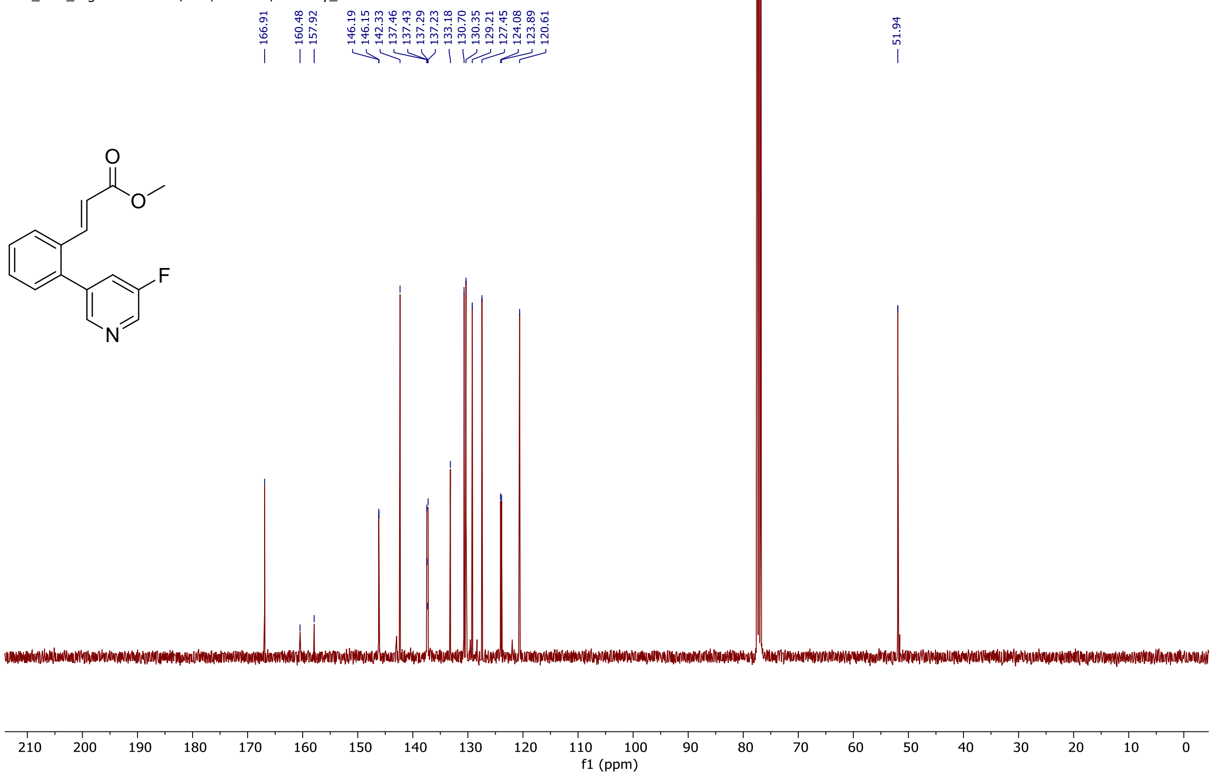

20210408-1429-B400_B.11-2.12.fid
Ref 530-3
Group Greaney_M
F19_NoCPD_Night CDCl3 /mnt/nmrdata/Greaney_M m31962tw 2

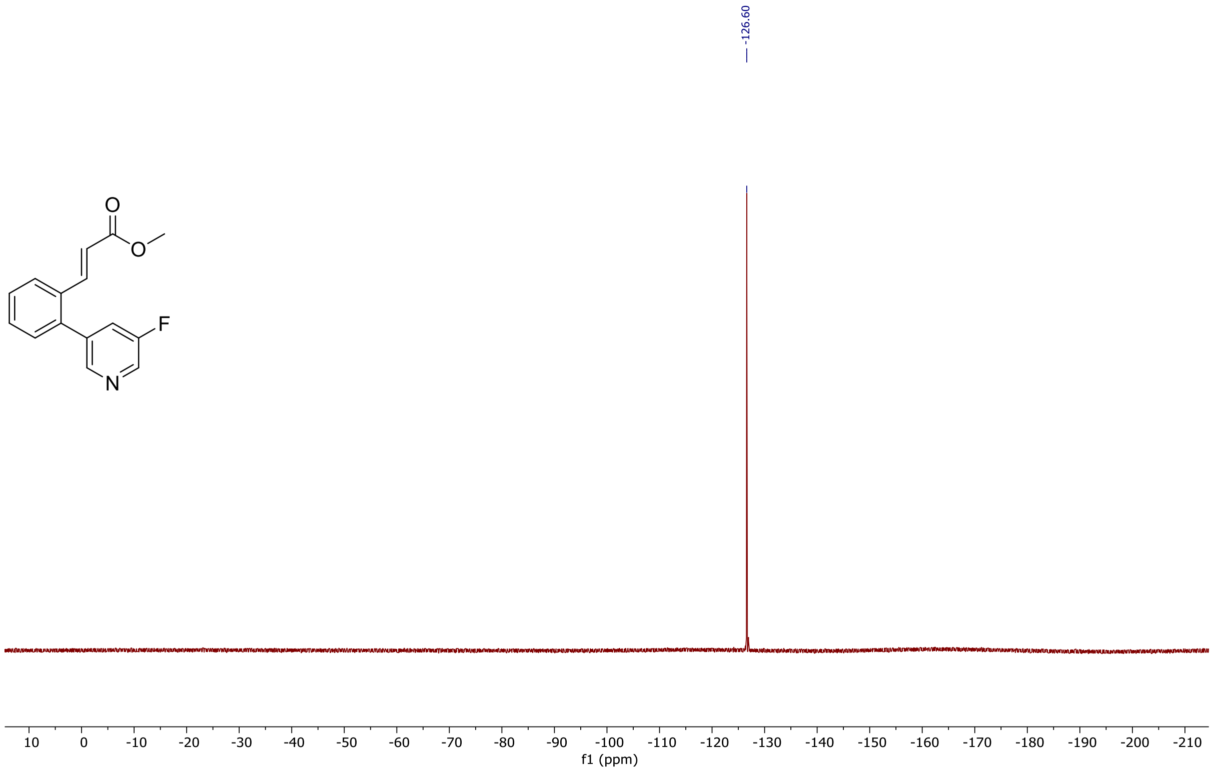

22u

20210408-1432-B400_B.11-53.10.fid
Ref 529-1
Group Greaney_M
H1_Night CDCl3 /mnt/nmrdata/Greaney_M m31962tw 53

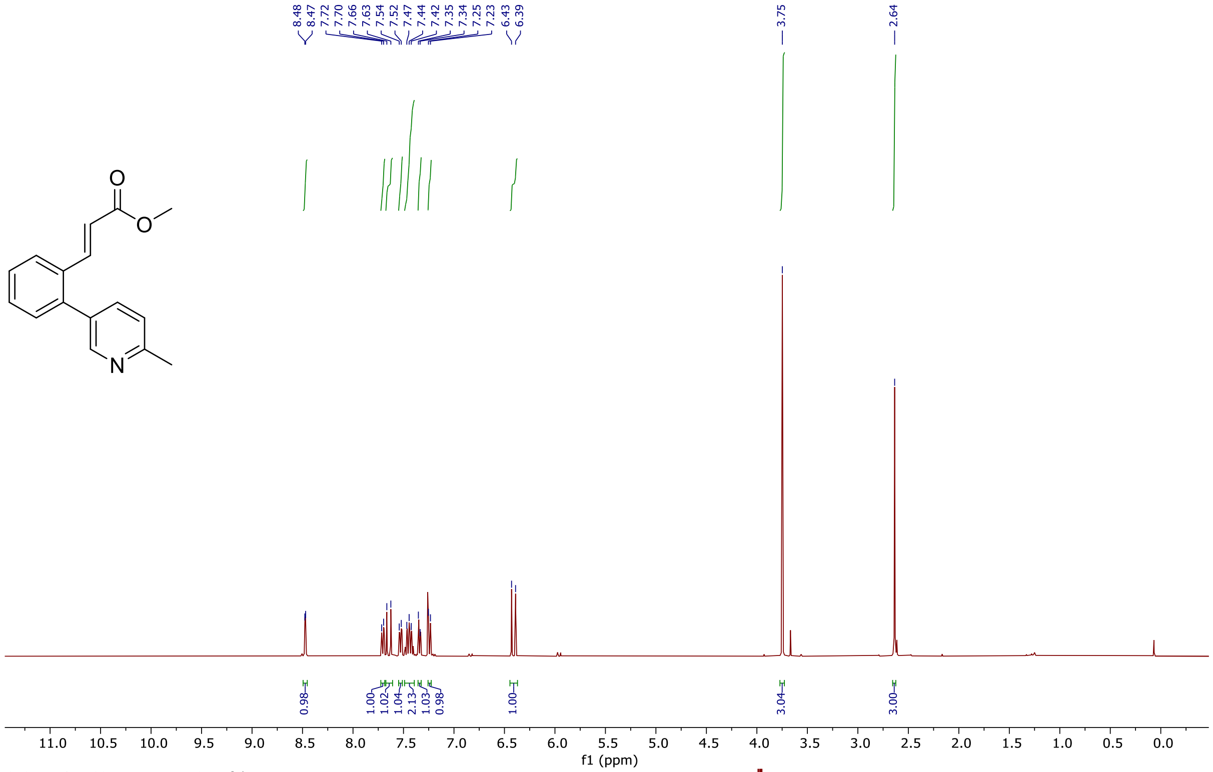

20210408-1432-B400_B.11-53.11.fid
Ref 529-1
Group Greaney_M
C13_CPD_Night256 CDCl3 /mnt/nmrdata/Greaney_M m31962tw 53

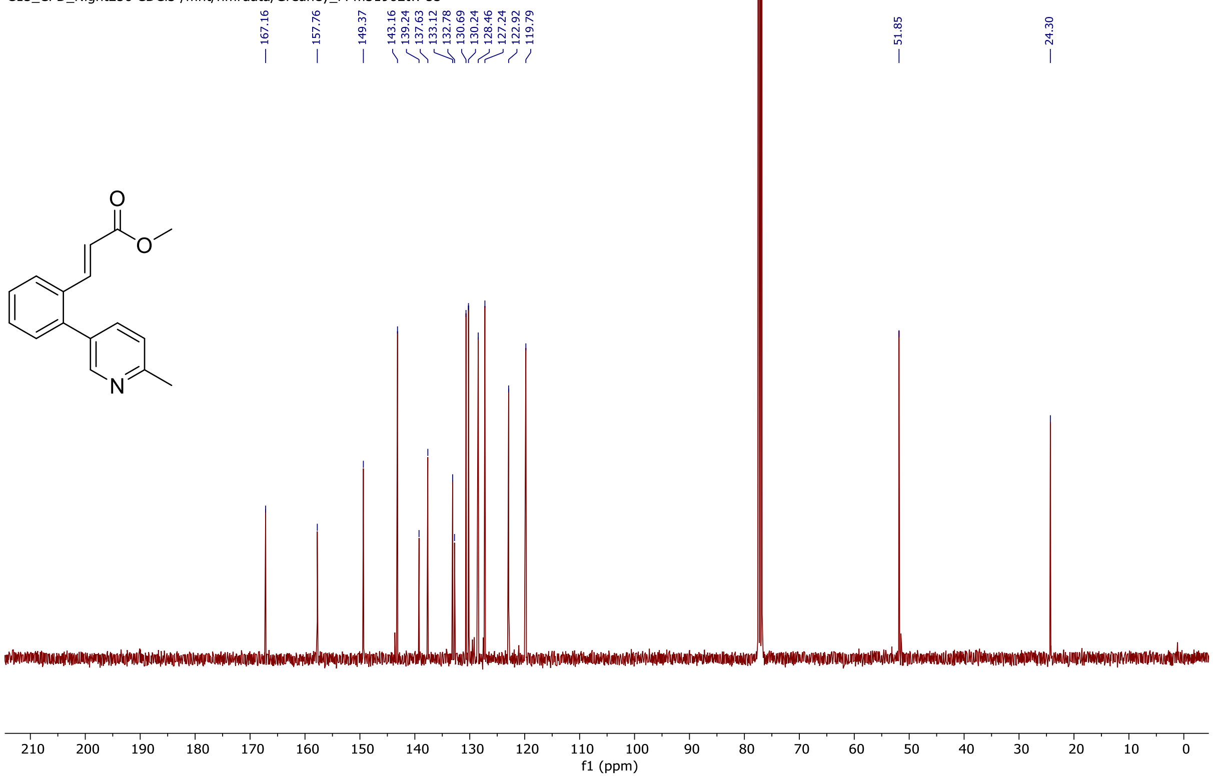

5a

20200225-1433-B400_B.12-49.12.fid
Ref 327-3
Group Greaney_M
H1_Night MeOD /mnt/nmrdata/Greaney_M m31962tw 49

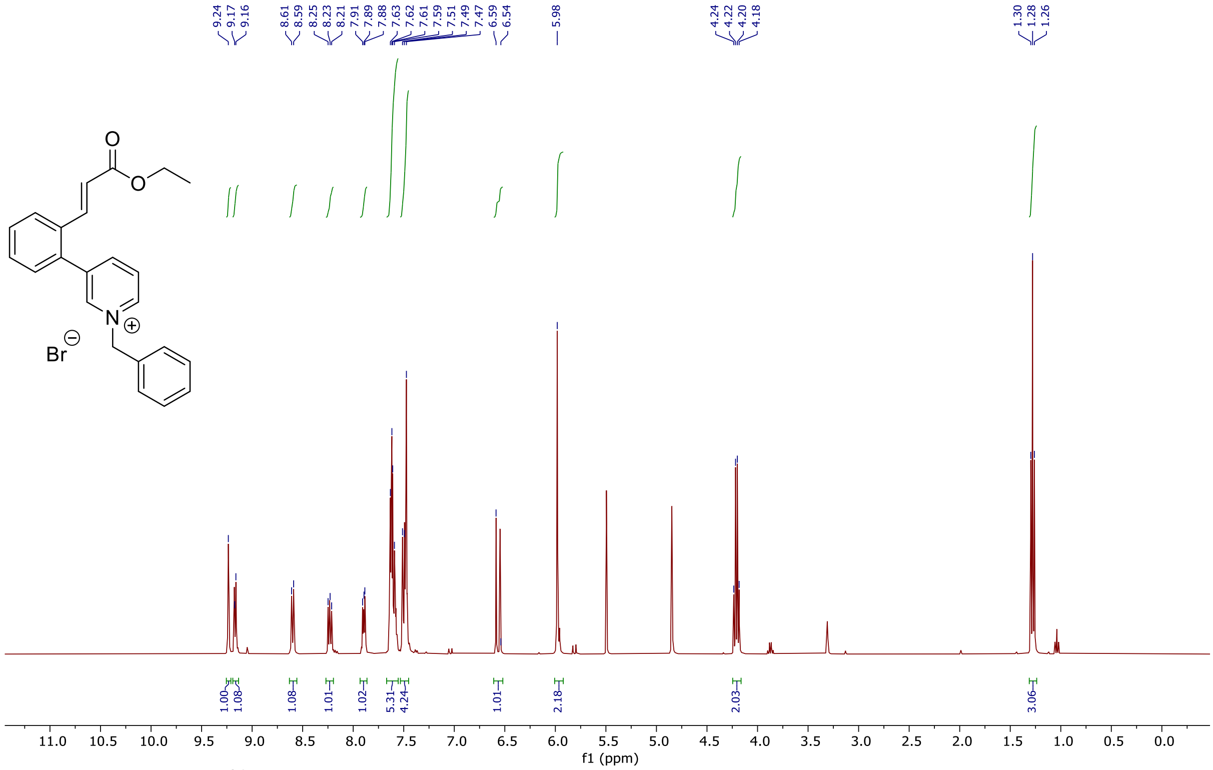

20200225-1433-B400_B.12-49.11.fid
Ref 327-3
Group Greaney_M
C13_CPD_Night256 MeOD /mnt/nmrdata/Greaney_M m31962tw 49

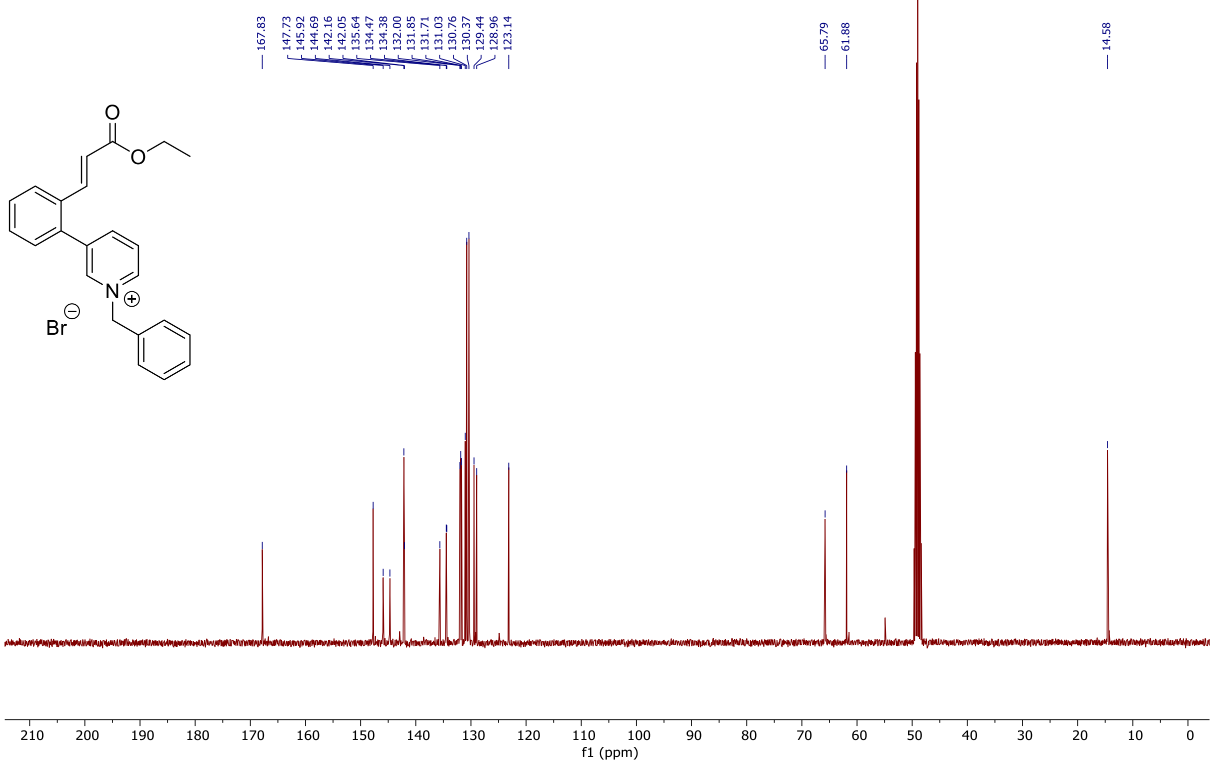

5a’

20210831-1812-B500_B.14-9.10.fid
Ref 358-2
Group Greaney_M
H1_Night MeOD /mnt/nmrdata/Greaney_M m31962tw 9

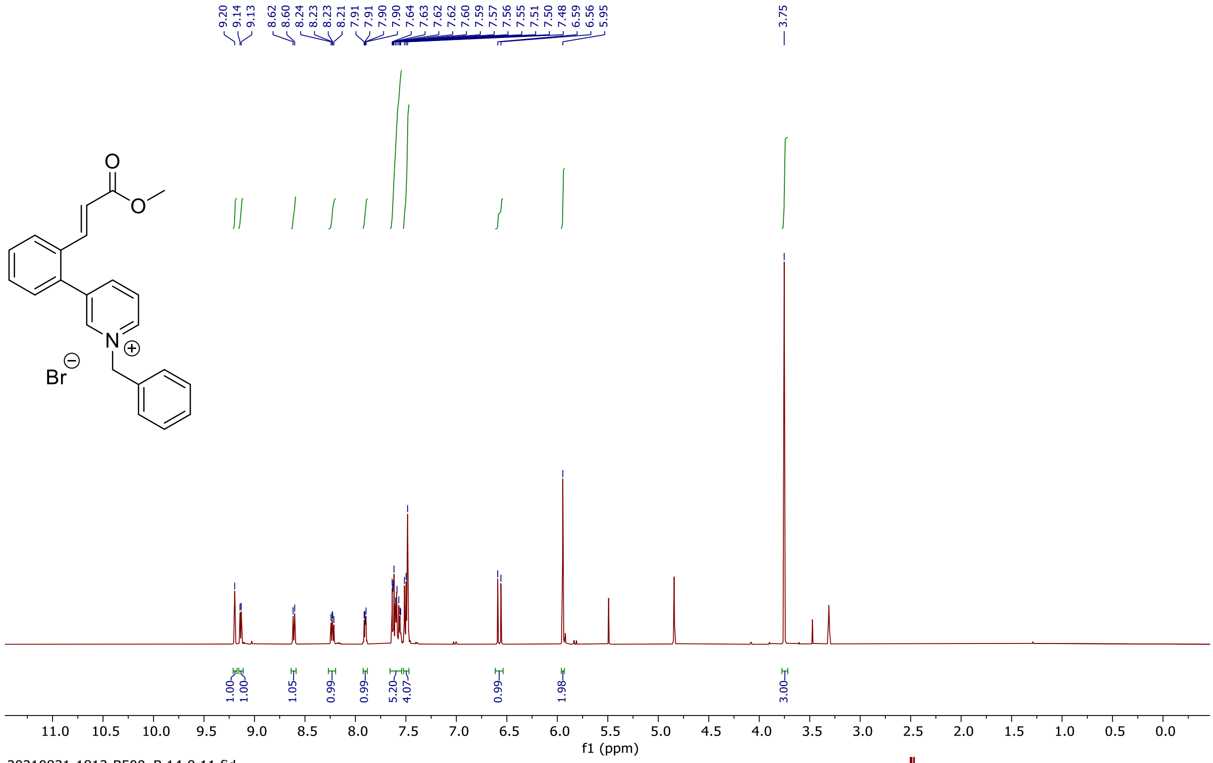

20210831-1812-B500_B.14-9.11.fid
Ref 358-2
Group Greaney_M
C13_CPD_Night256 MeOD /mnt/nmrdata/Greaney_M m31962tw 9

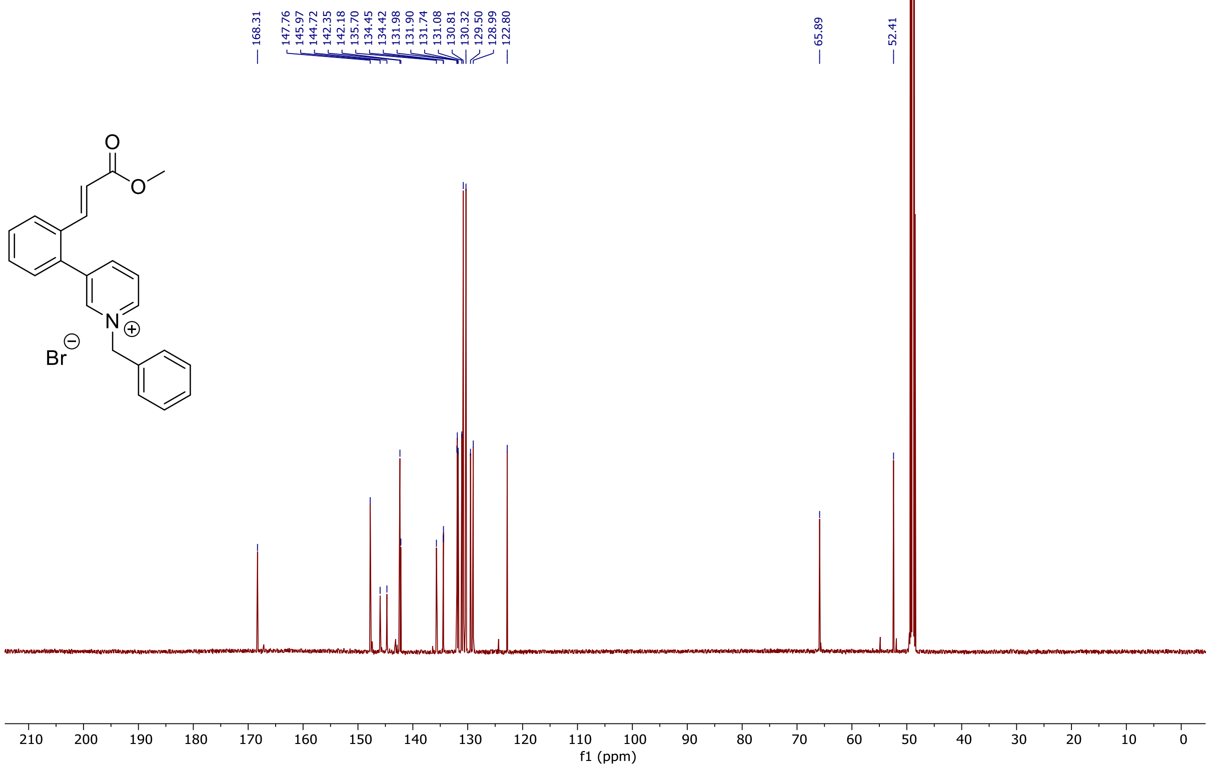

5b

20210409-1708-B400_B.11-58.10.fid
Ref 471-2
Group Greaney_M
H1_Night MeOD /mnt/nmrdata/Greaney_M m31962tw 58

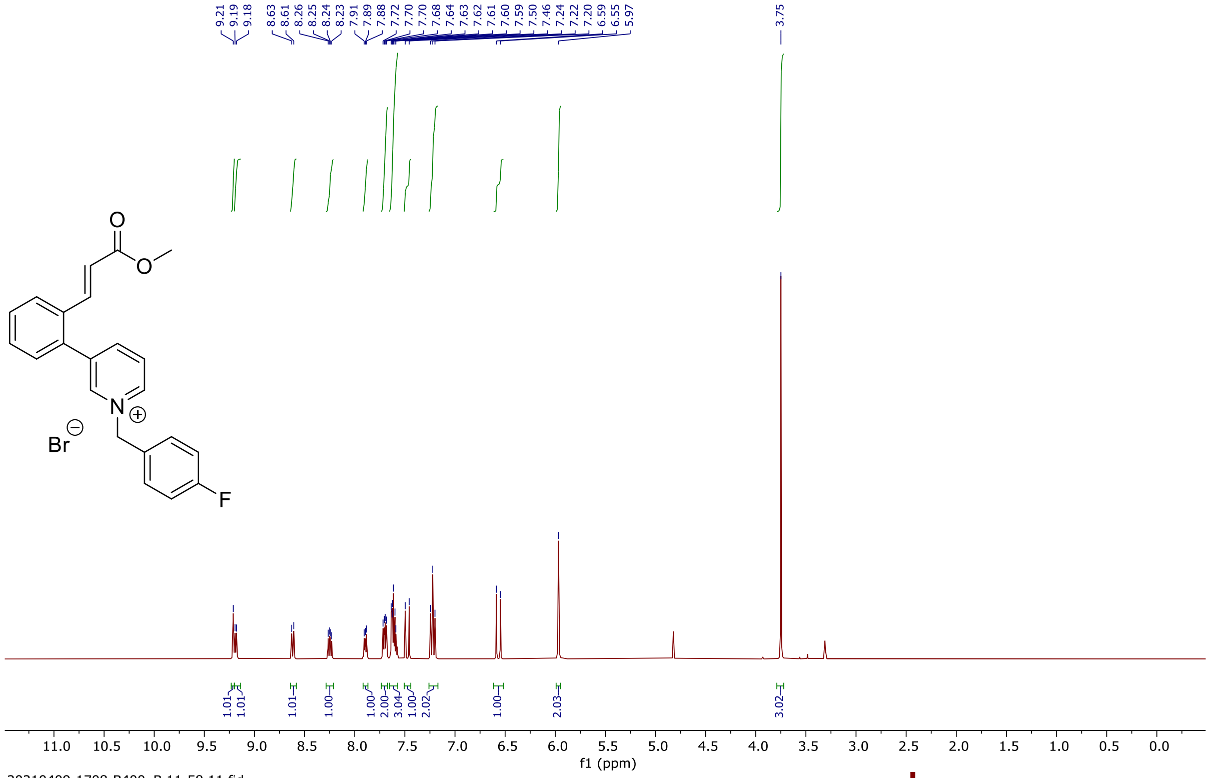

20210409-1708-B400_B.11-58.11.fid
Ref 471-2
Group Greaney_M
C13_CPD_Night256 MeOD /mnt/nmrdata/Greaney_M m31962tw 58

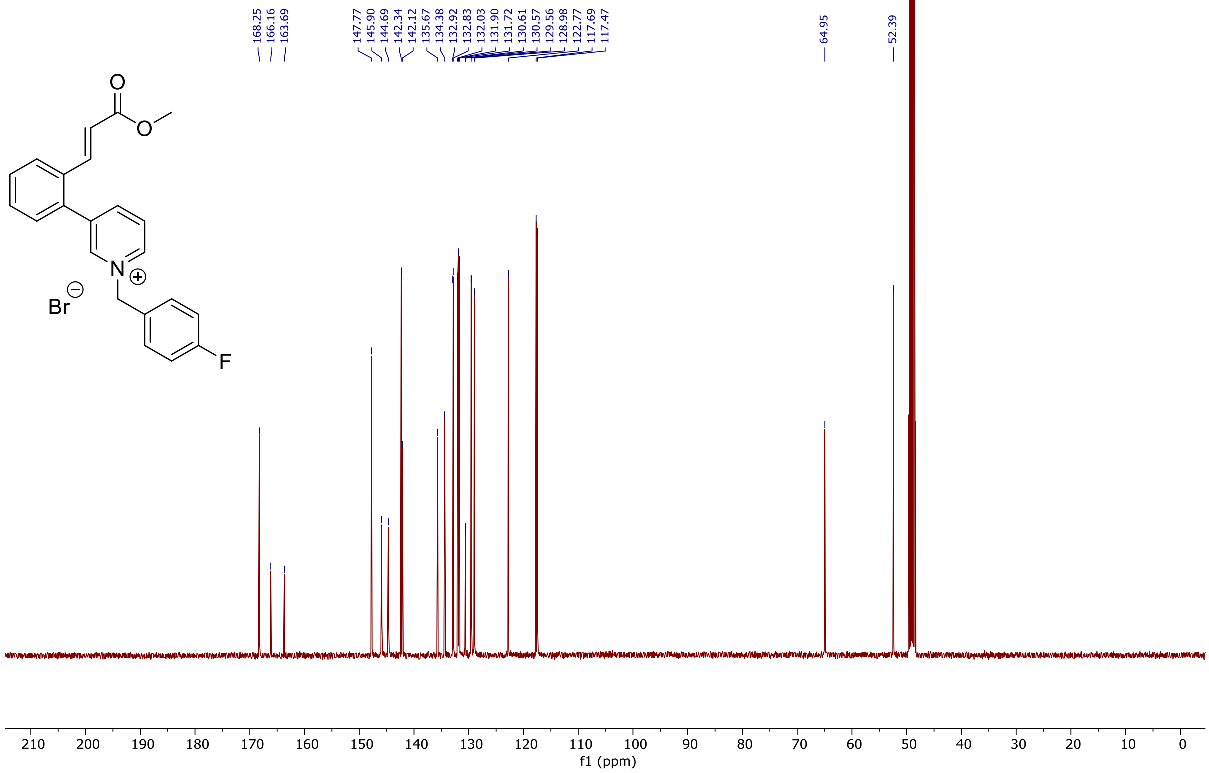

20210409-1708-B400_B.11-58.15.fid
Ref 471-2
Group Greaney_M
F19_NoCPD_Night MeOD /mnt/nmrdata/Greaney_M m31962tw 58

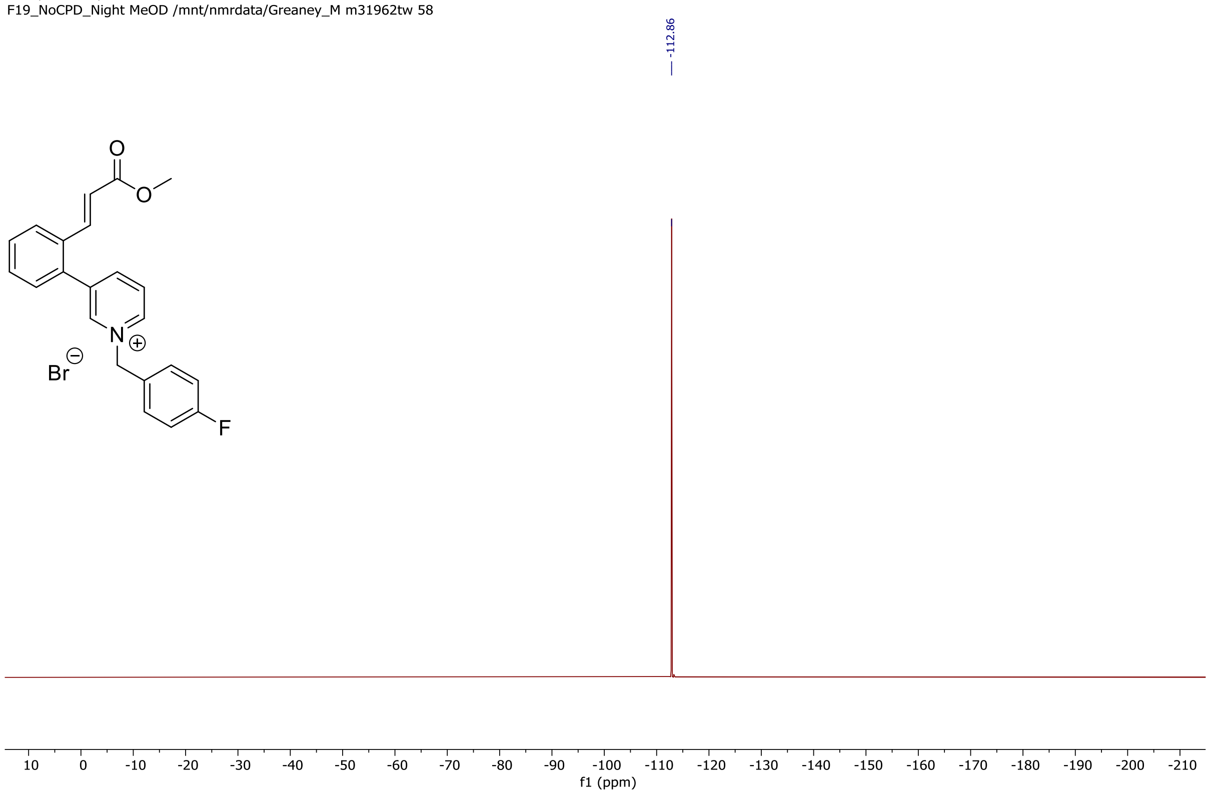

5b'

20211126-1550-B400_B.11-29.10.fid
Ref 710-2
Group Greaney_M
H1_Night MeOD /mnt/nmrdata/Greaney_M m31962tw 29

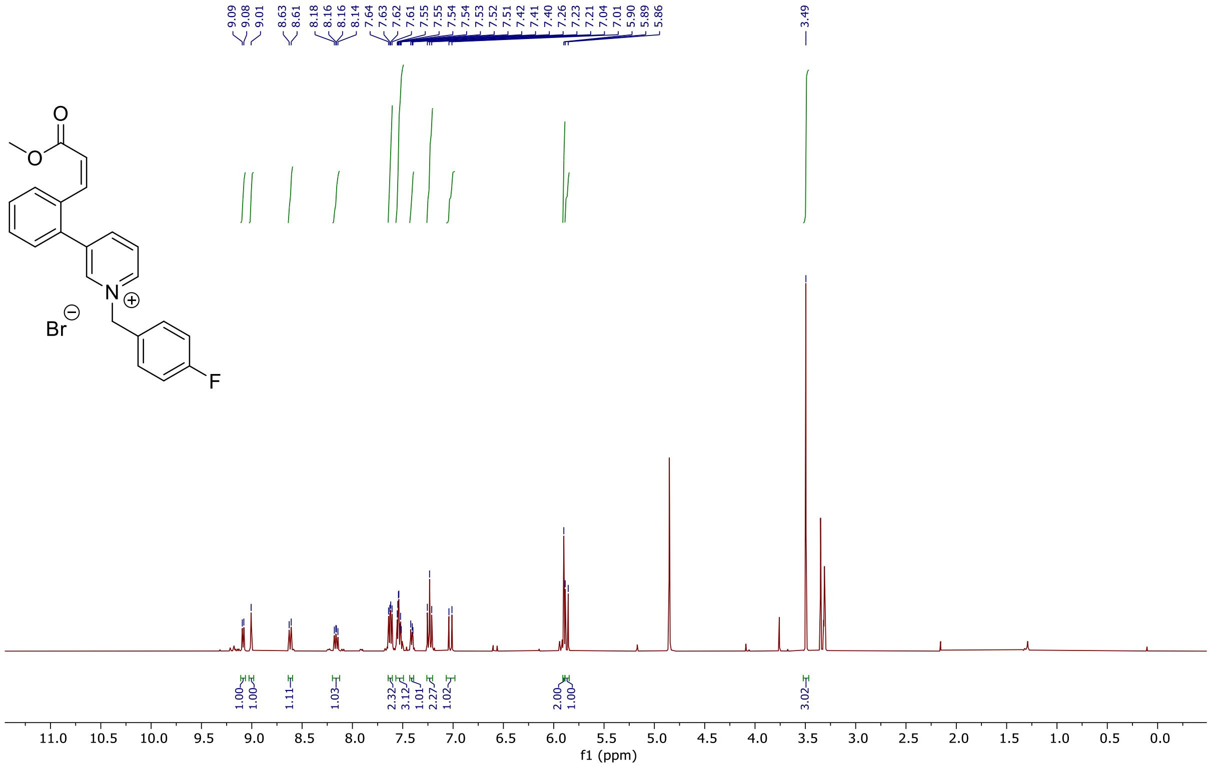

20211126-1550-B400_B.11-29.11.fid
Ref 710-2
Group Greaney_M
C13_CPD_Night256 MeOD /mnt/nmrdata/Greaney_M m31962tw 29

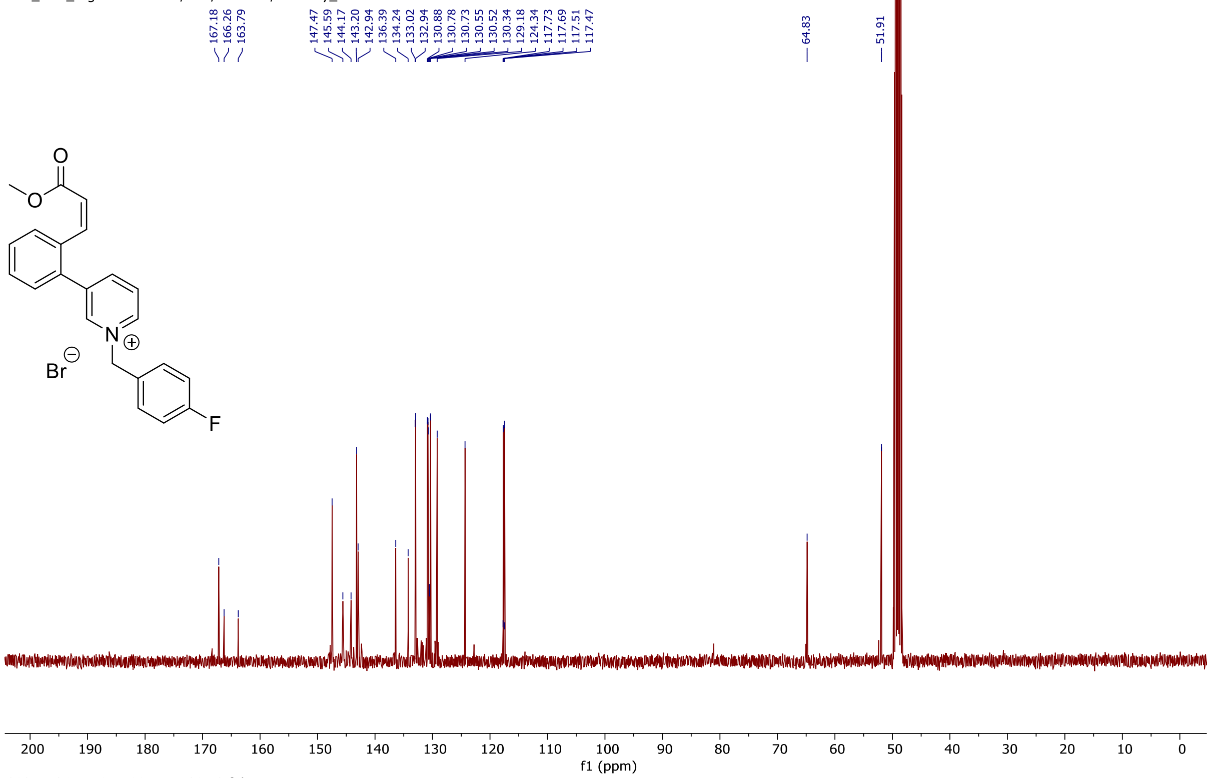

20211126-1550-B400_B.11-29.12.fid
Ref 710-2
Group Greaney_M
F19_NoCPD_Night MeOD /mnt/nmrdata/Greaney_M m31962tw 29

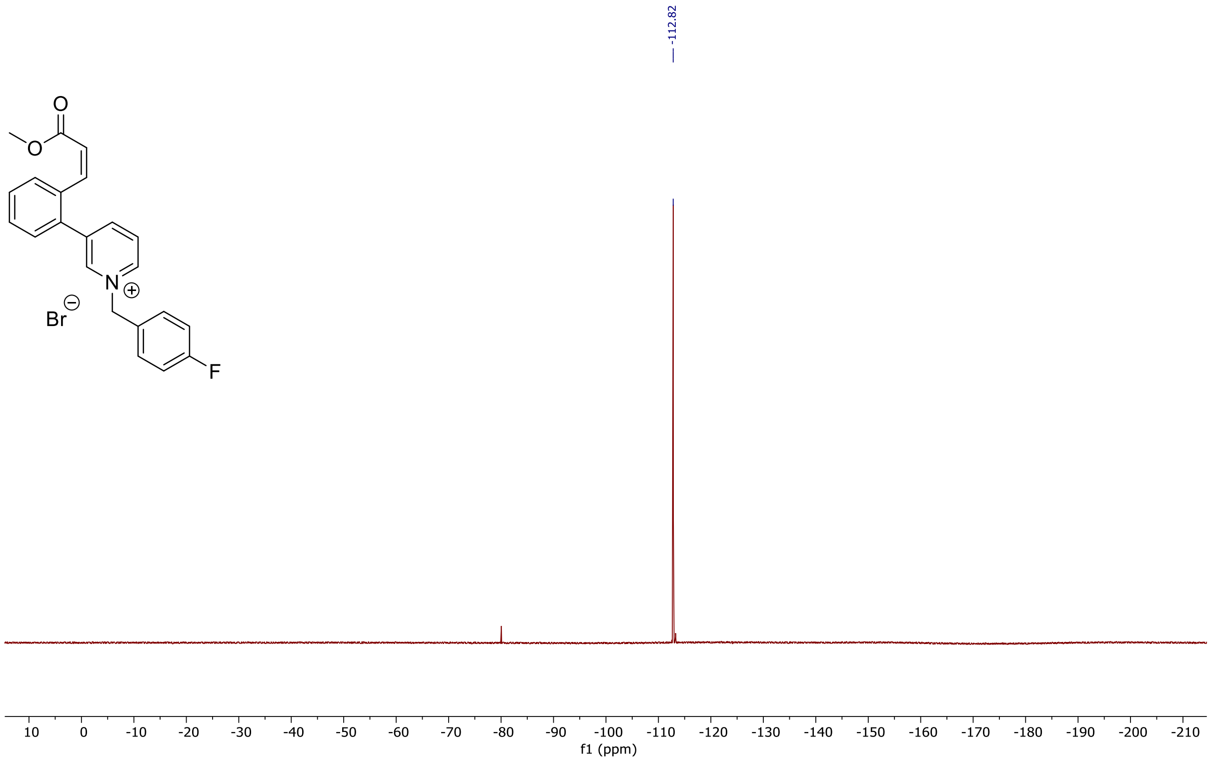

5c

20210427-1018-B400_B.11-32.10.fid
Ref 537-1
Group Greaney_M
H1_Night MeOD /mnt/nmrdata/Greaney_M m31962tw 32

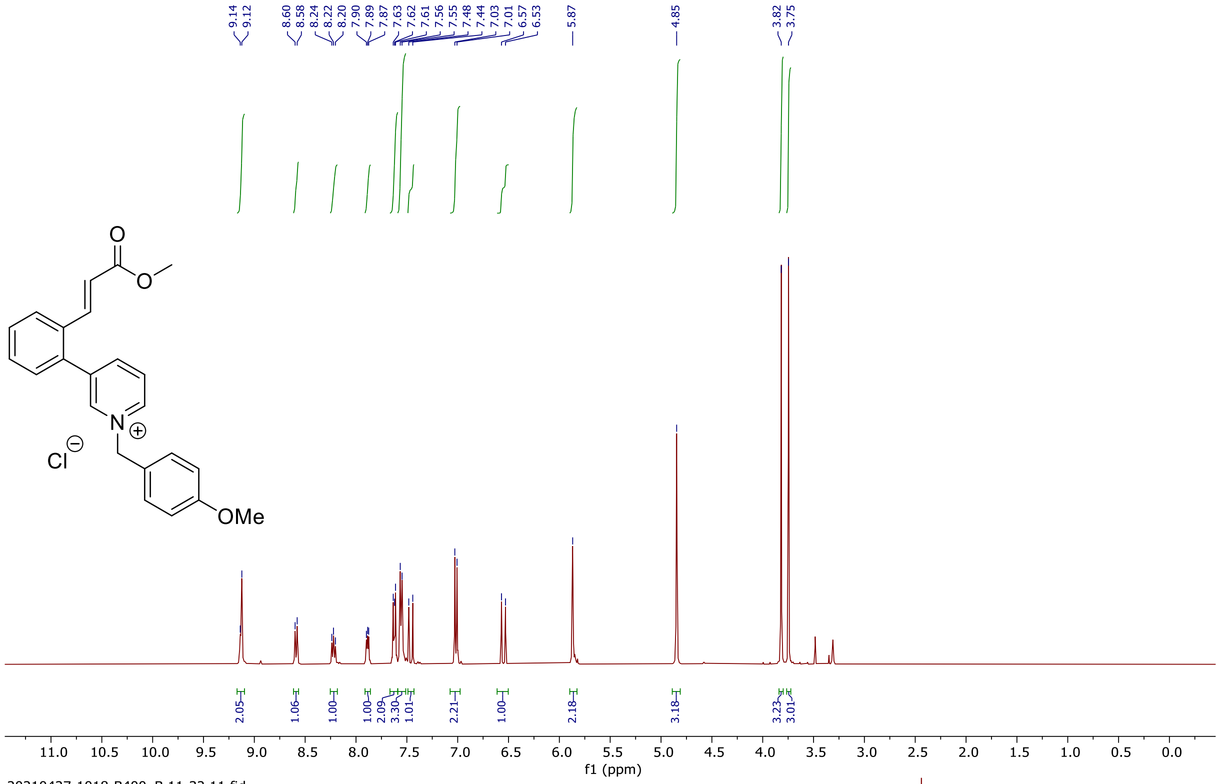

20210427-1018-B400_B.11-32.11.fid
Ref 537-1
Group Greaney_M
C13_CPD_Night256 MeOD /mnt/nmrdata/Greaney_M m31962tw 32

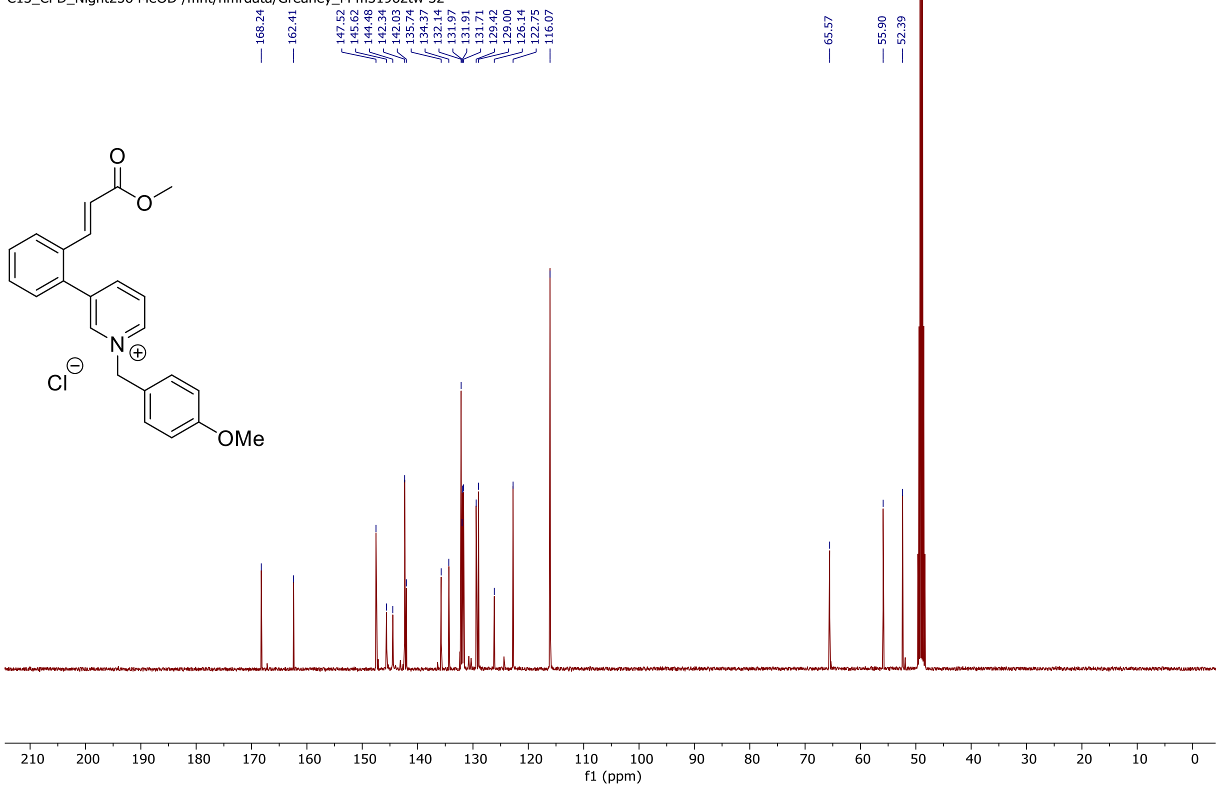

5d

20201016-1554-B500_B.14-60.10.fid
Ref 435-1
Group Greaney_M
H1_Day MeOD /mnt/nmrdata/Greaney_M m31962tw 60

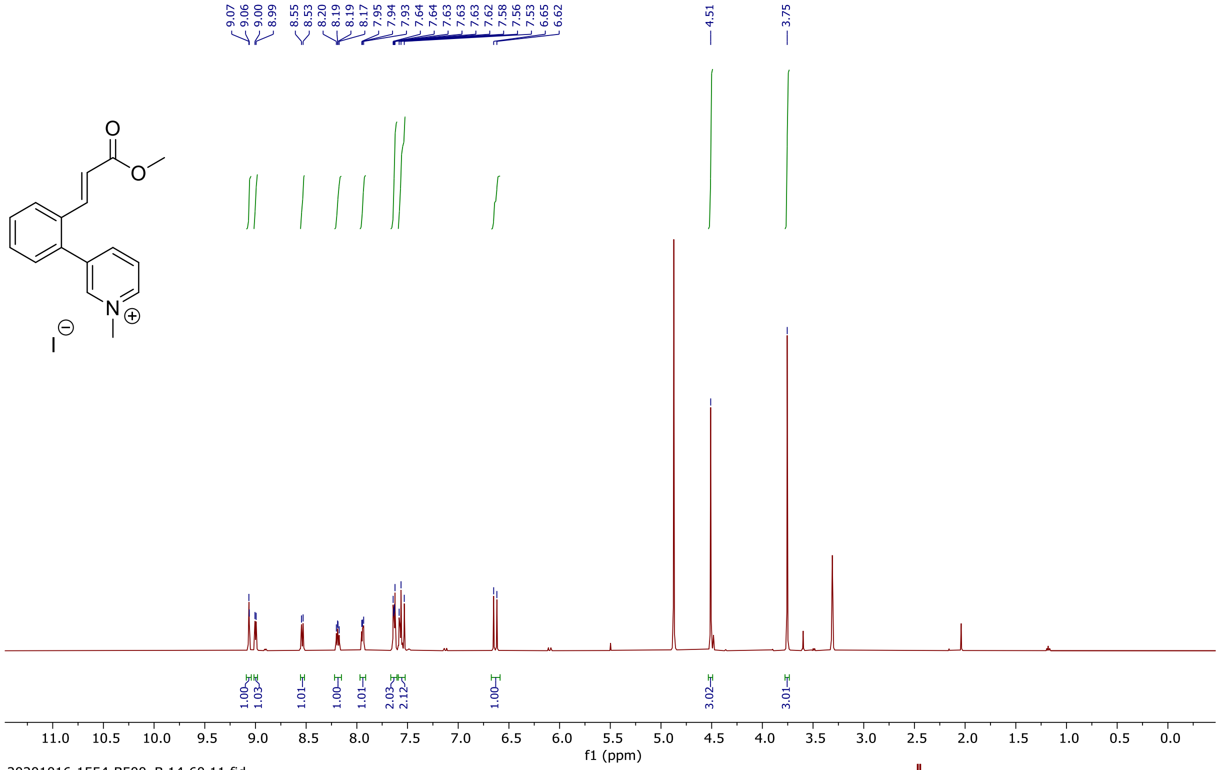

20201016-1554-B500_B.14-60.11.fid
Ref 435-1
Group Greaney_M
C13_CPD_Night256 MeOD /mnt/nmrdata/Greaney_M m31962tw 60

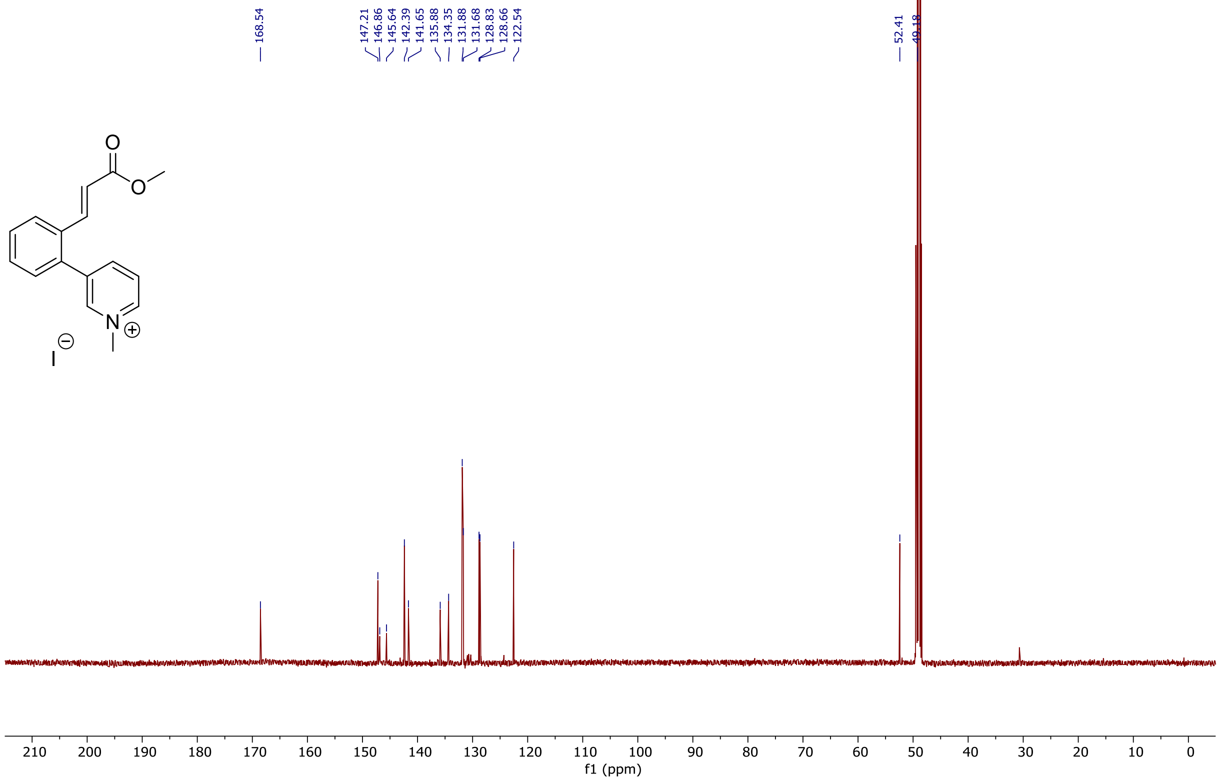

5e

20201123-1703-B400_B.11-41.10.fid
Ref 461-5
Group Greaney_M
H1_Night MeOD /mnt/nmrdata/Greaney_M m31962tw 41

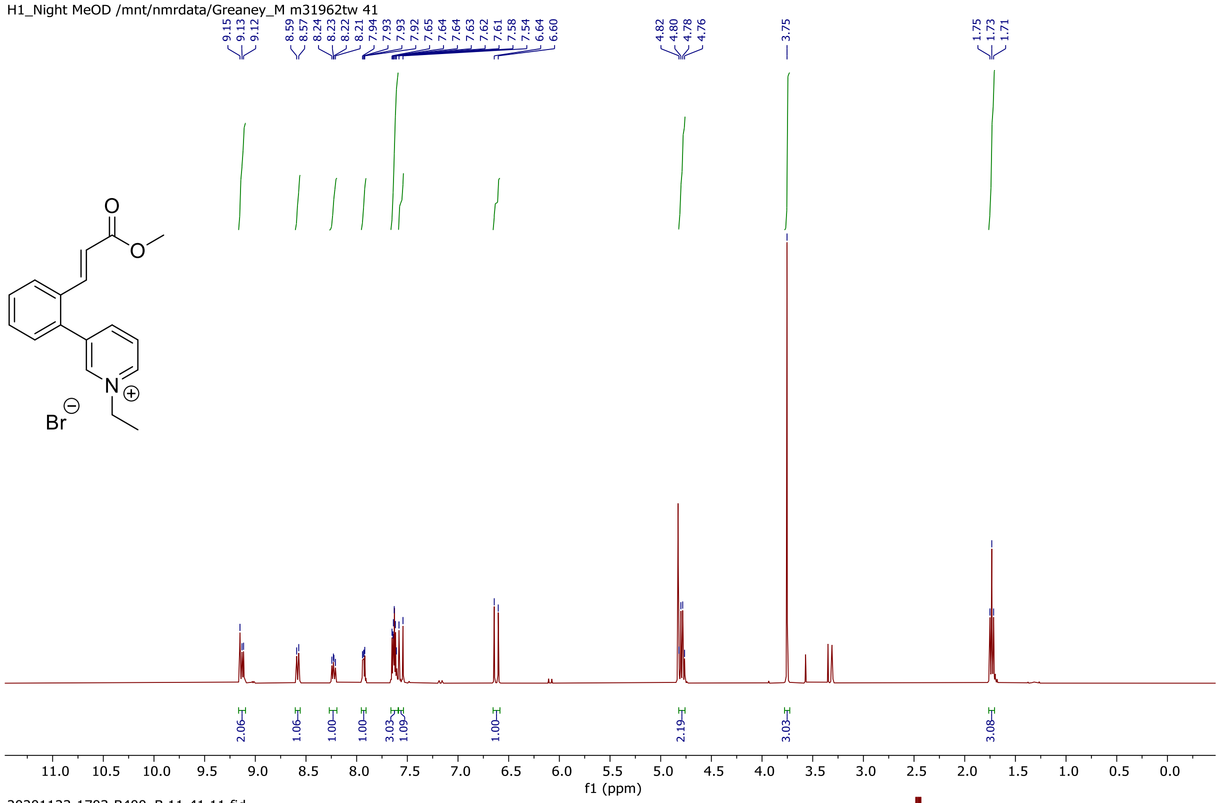

20201123-1703-B400_B.11-41.11.fid
Ref 461-5
Group Greaney_M
C13_CPD_Night256 MeOD /mnt/nmrdata/Greaney_M m31962tw 41

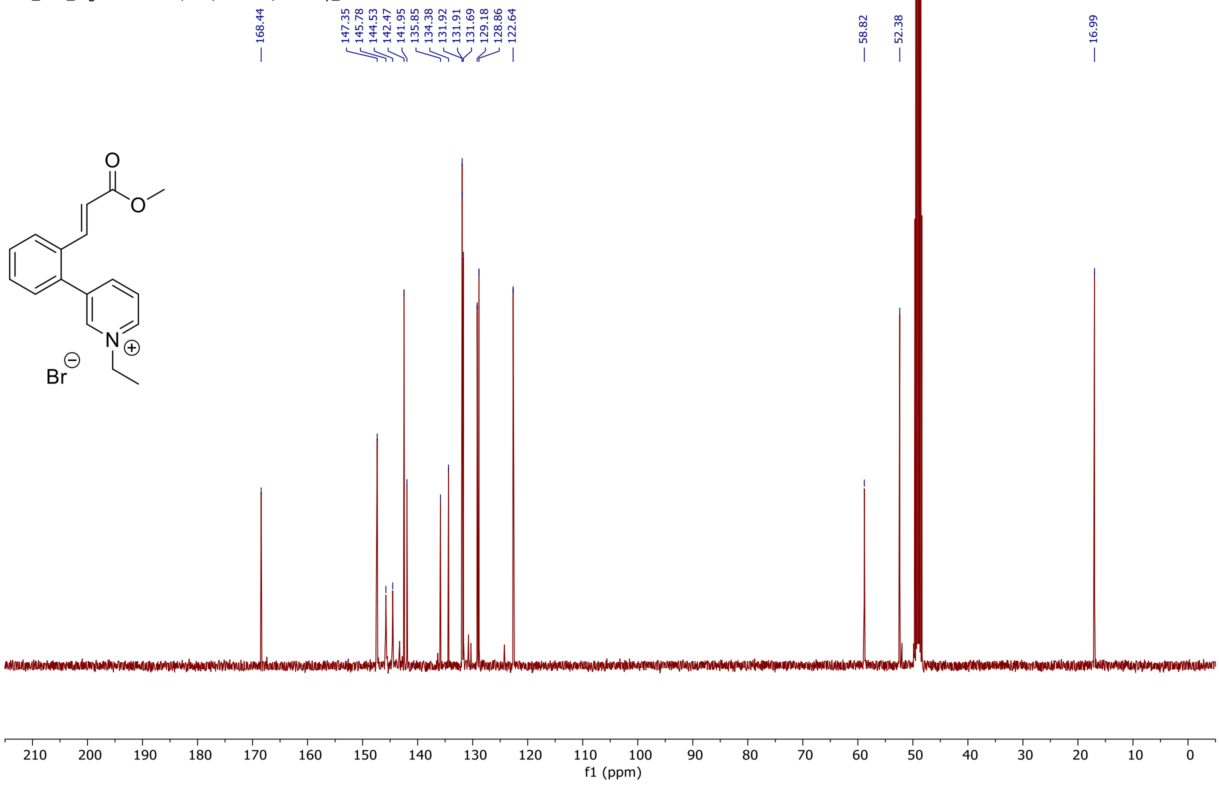

5f

20201123-1703-B400_B.11-40.10.fid
Ref 461-4
Group Greaney_M
H1_Night MeOD /mnt/nmrdata/Greaney_M m31962tw 40

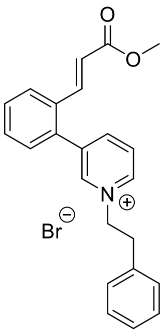

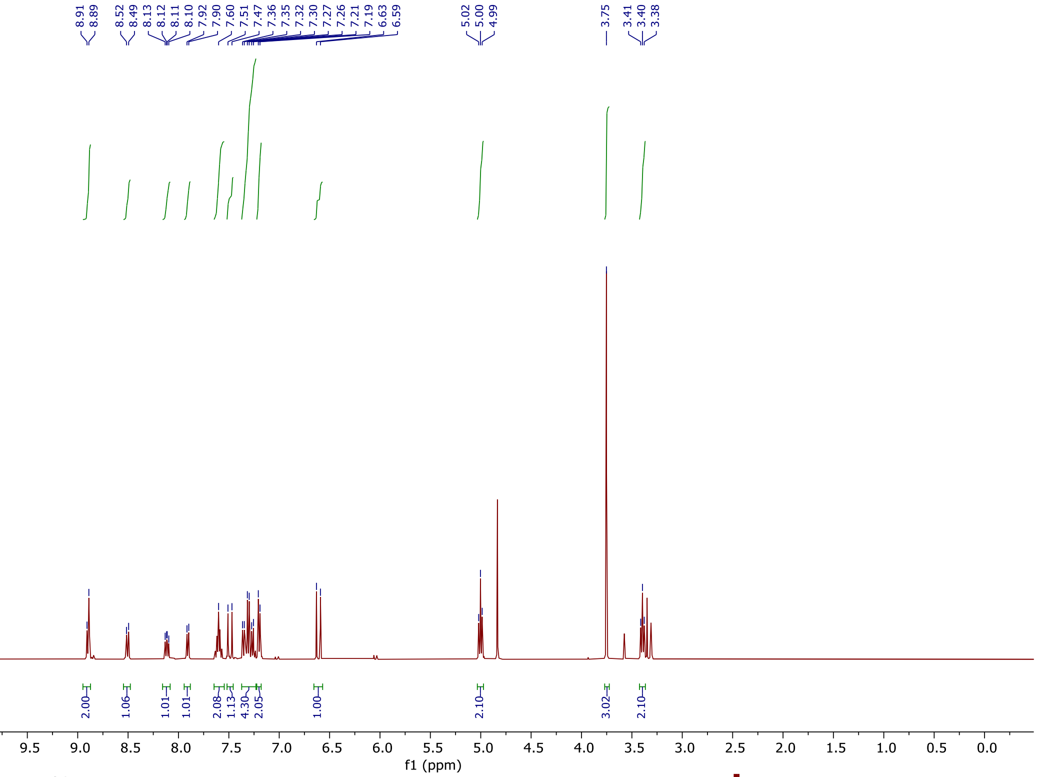

20201123-1703-B400_B.11-40.11.fid
Ref 461-4
Group Greaney_M
C13_CPD_Night256 MeOD /mnt/nmrdata/Greaney_M m31962tw 40

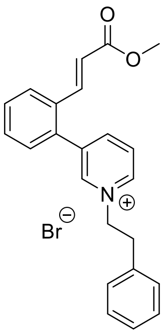

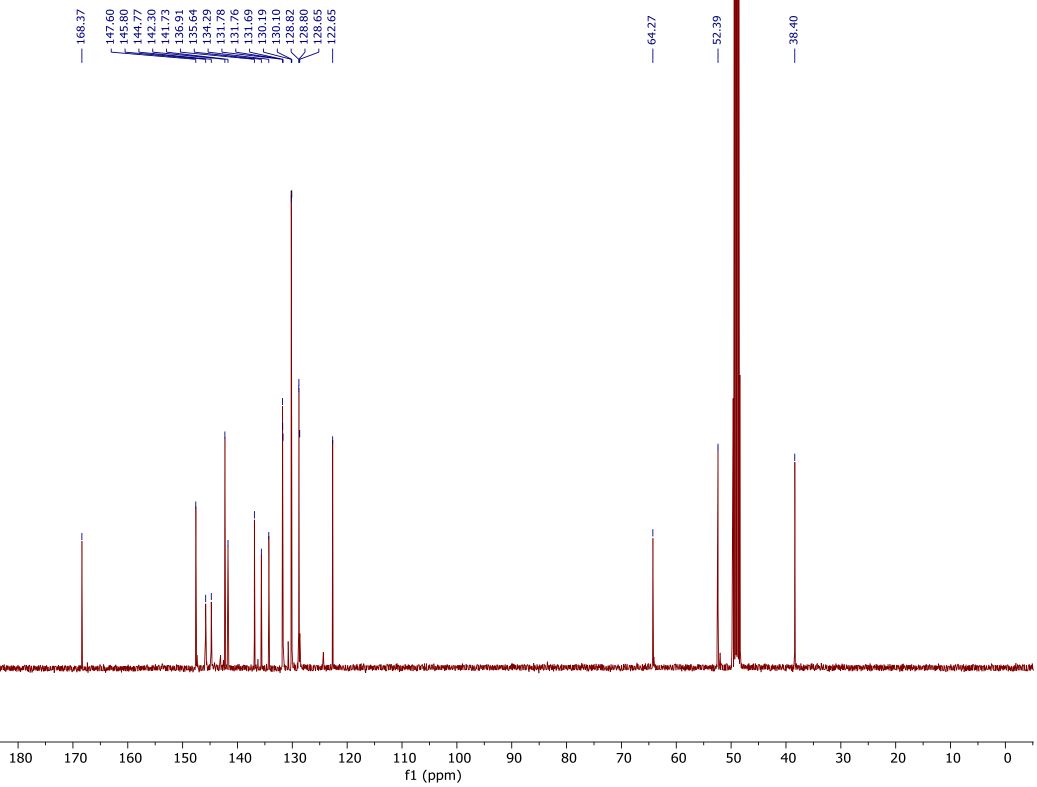

5g

20210429-1302-B400_B.11-43.10.fid
Ref 480-4
Group Greaney_M
H1_Night MeOD /mnt/nmrdata/Greaney_M m31962tw 43

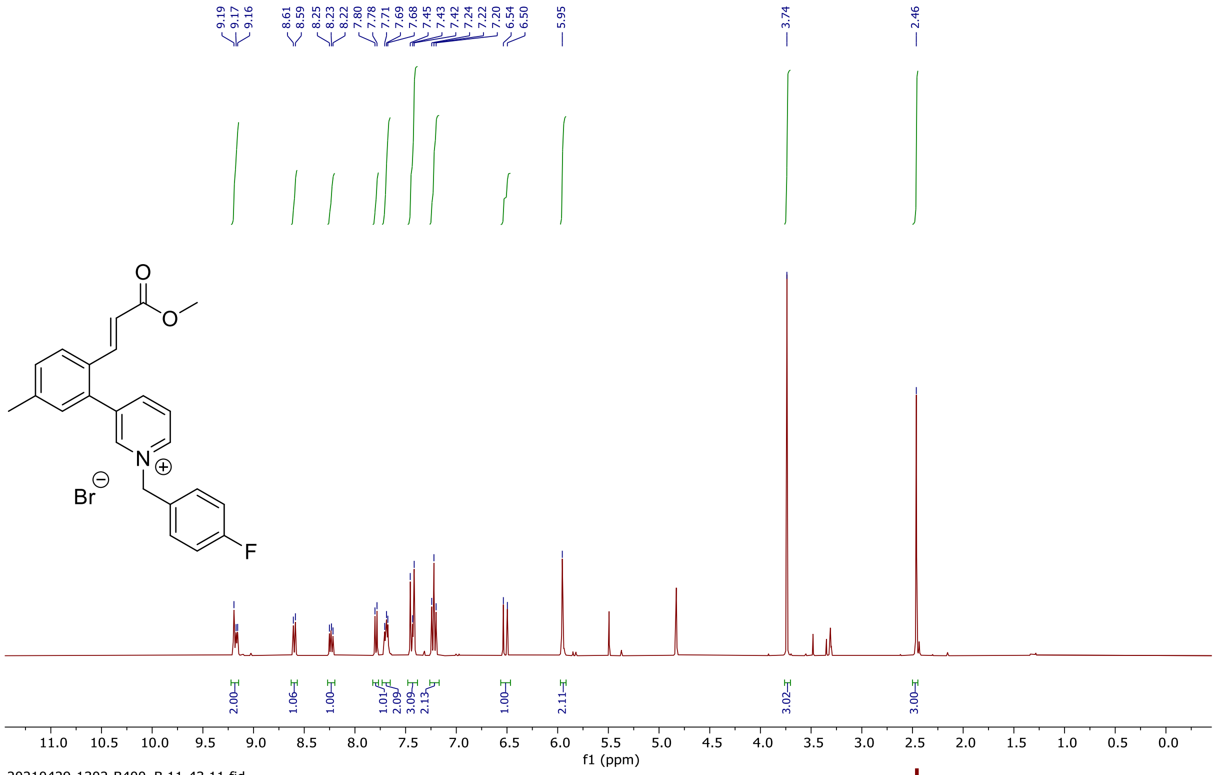

20210429-1302-B400_B.11-43.11.fid
Ref 480-4
Group Greaney_M
C13_CPD_Night256 MeOD /mnt/nmrdata/Greaney_M m31962tw 43

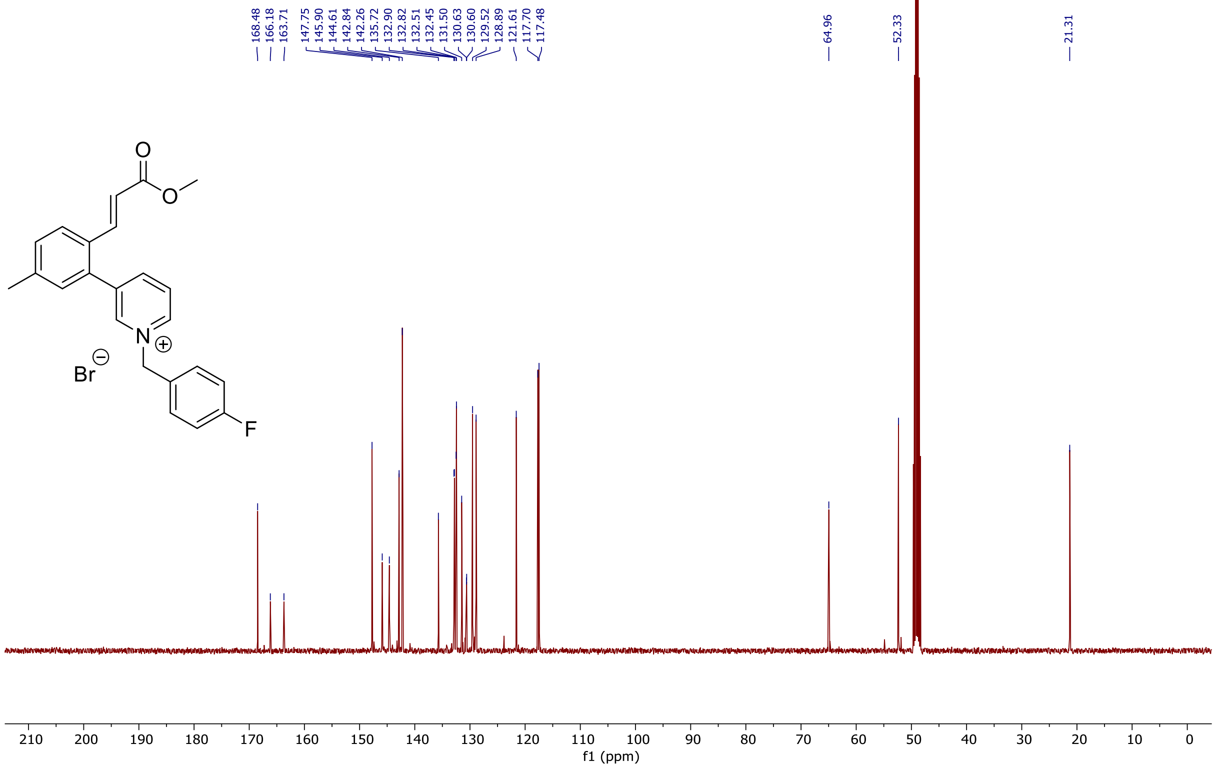

20210429-1302-B400_B.11-43.12.fid
Ref 480-4
Group Greaney_M
F19_NoCPD_Night MeOD /mnt/nmrdata/Greaney_M m31962tw 43

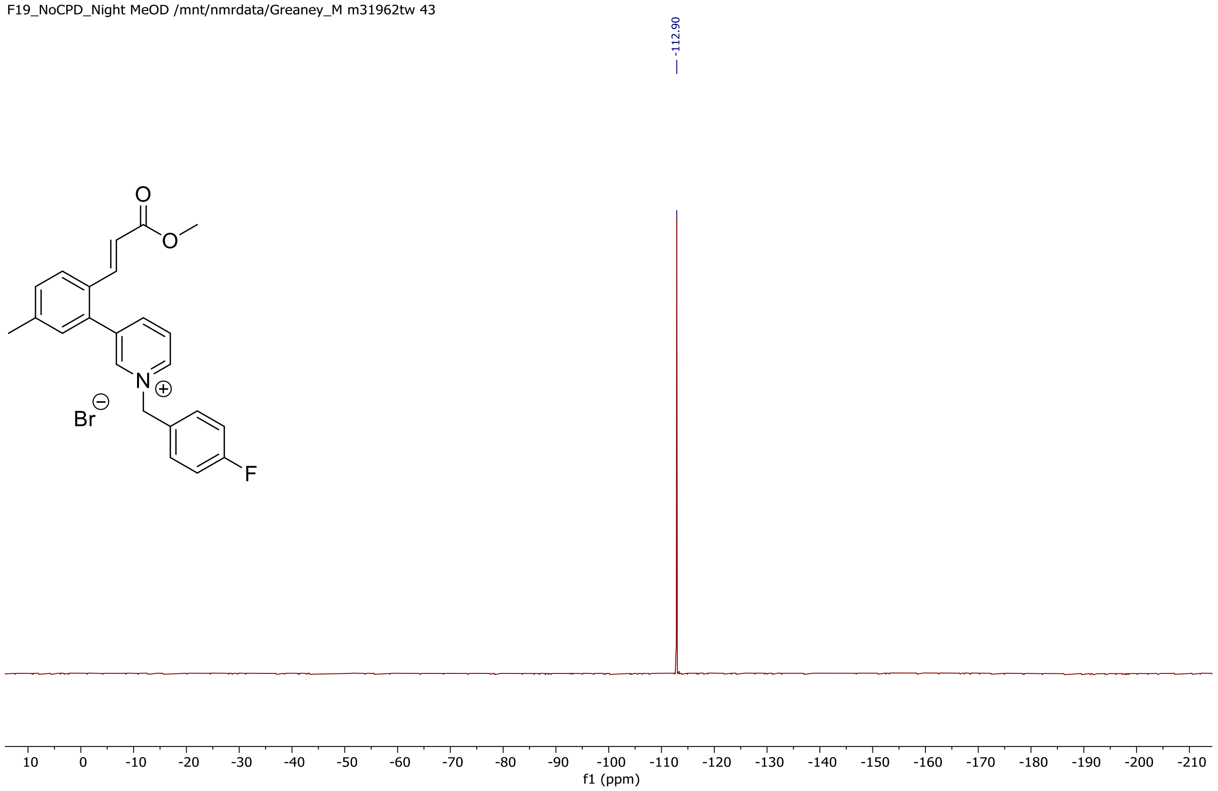

5h

20201204-1813-B400_B.12-17.10.fid
Ref 480-2
Group Greaney_M
H1_Night MeOD /mnt/nmrdata/Greaney_M m31962tw 17

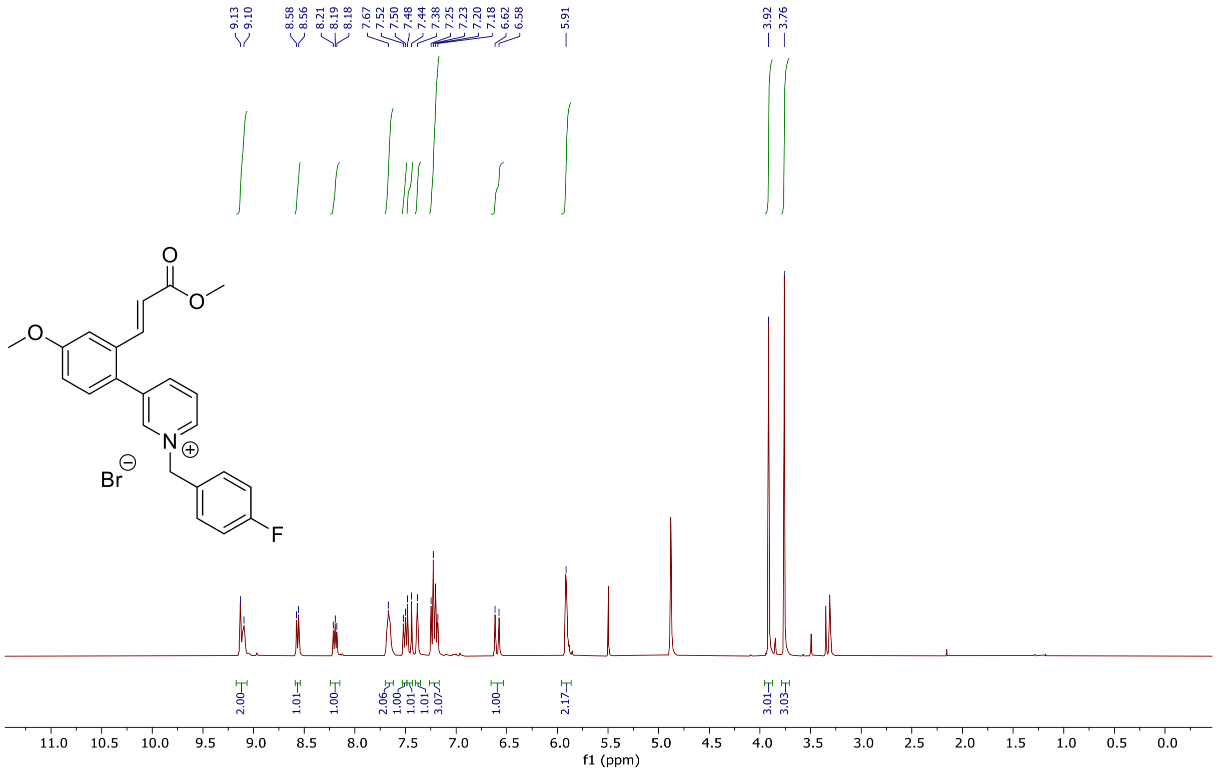

20201204-1813-B400_B.12-17.14.fid
Ref 480-2
Group Greaney_M
C13_CPD_Night256 MeOD /mnt/nmrdata/Greaney_M m31962tw 17

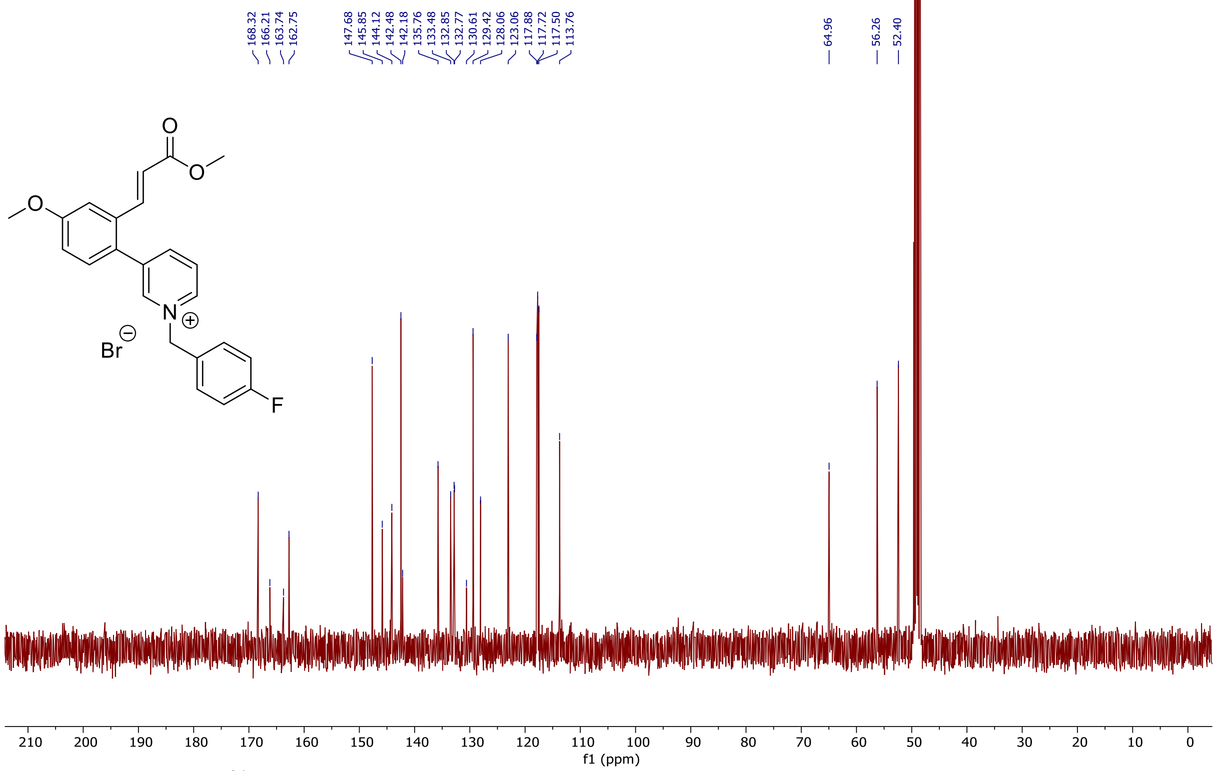

20201204-1813-B400_B.12-17.15.fid
Ref 480-2
Group Greaney_M
F19_CPD_Night MeOD /mnt/nmrdata/Greaney_M m31962tw 17

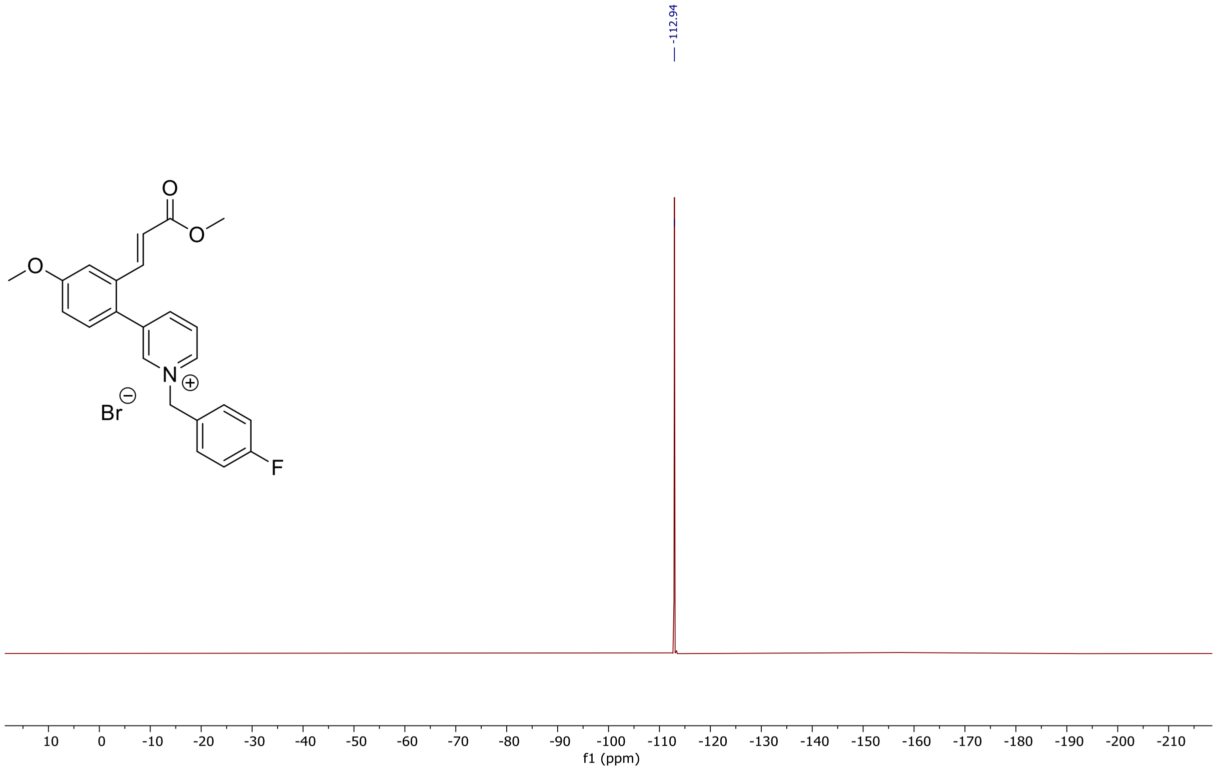

5i

20201204-1811-B400_B.12-21.10.fid
Ref 480-6
Group Greaney_M
H1_Night MeOD /mnt/nmrdata/Greaney_M m31962tw 21

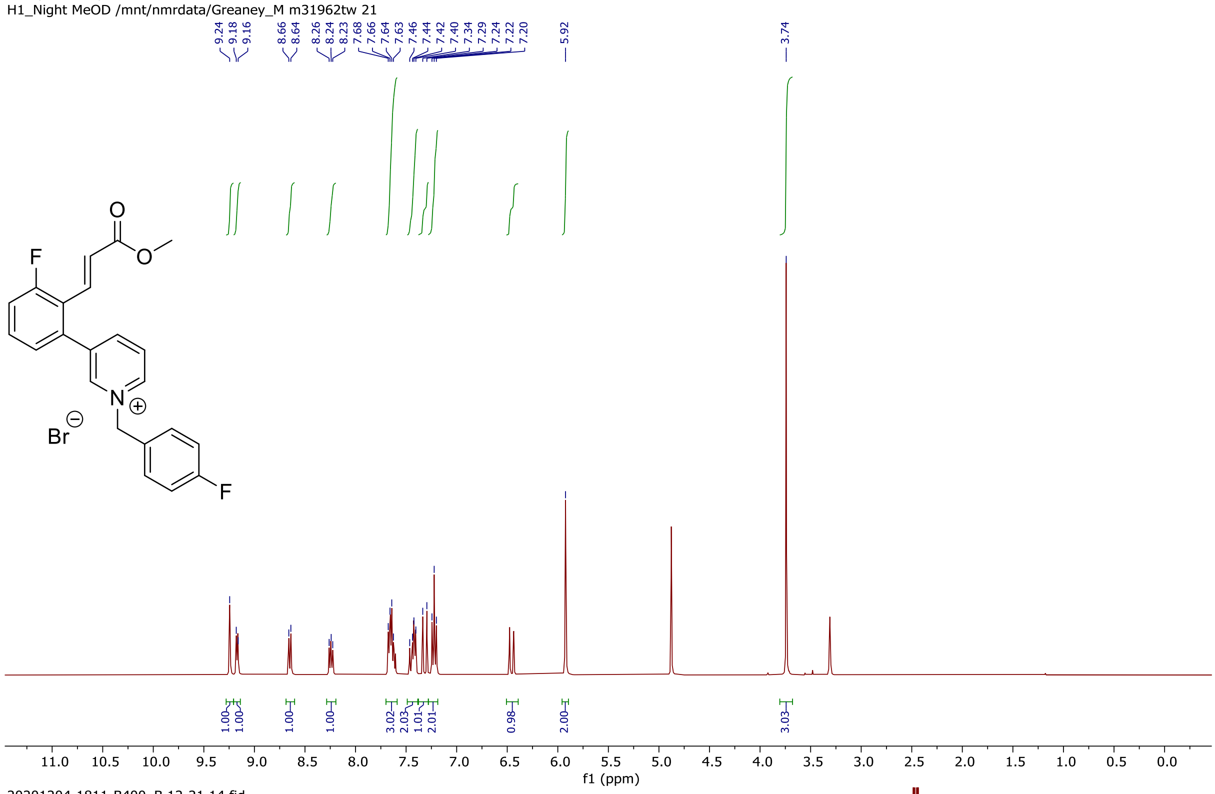

20201204-1811-B400_B.12-21.14.fid
Ref 480-6
Group Greaney_M
C13_CPD_Night256 MeOD /mnt/nmrdata/Greaney_M m31962tw 21

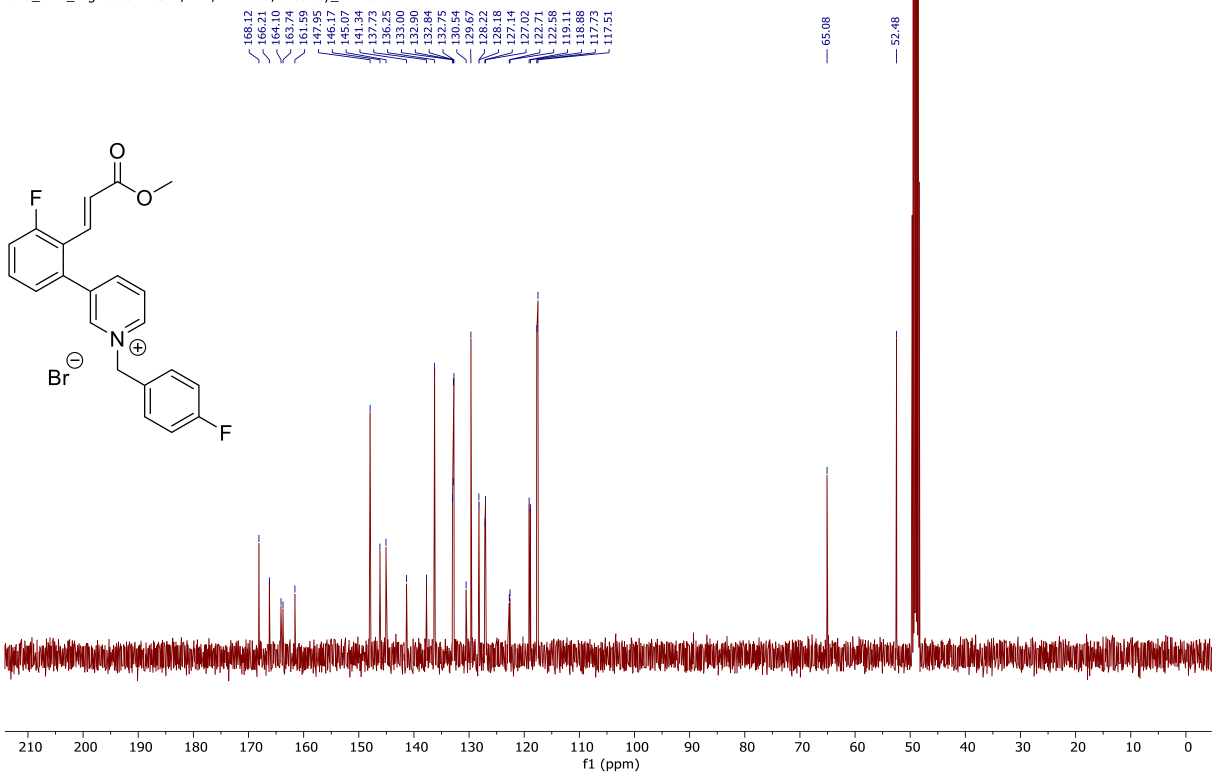

20201204-1811-B400_B.12-21.15.fid
Ref 480-6
Group Greaney_M
F19_CPD_Night MeOD /mnt/nmrdata/Greaney_M m31962tw 21

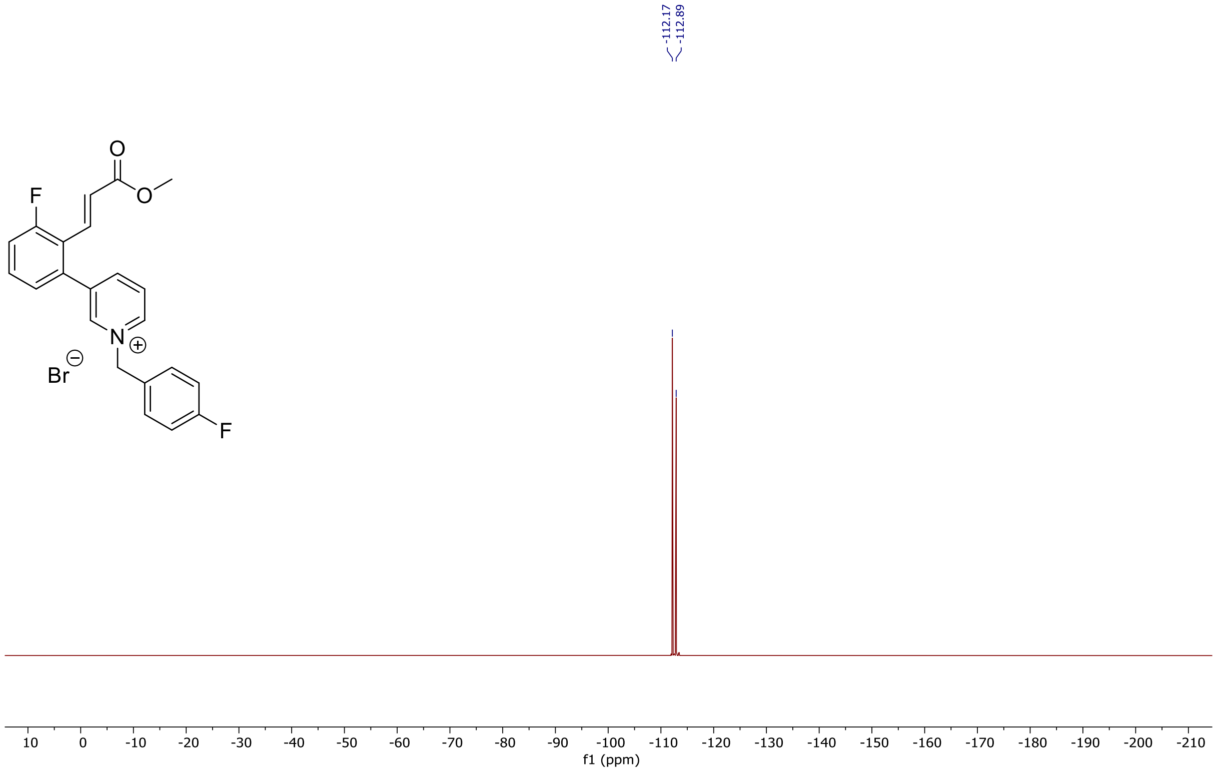

5j

20201204-1810-B400_B.12-22.10.fid
Ref 480-7
Group Greaney_M
H1_Night MeOD /mnt/nmrdata/Greaney_M m31962tw 22

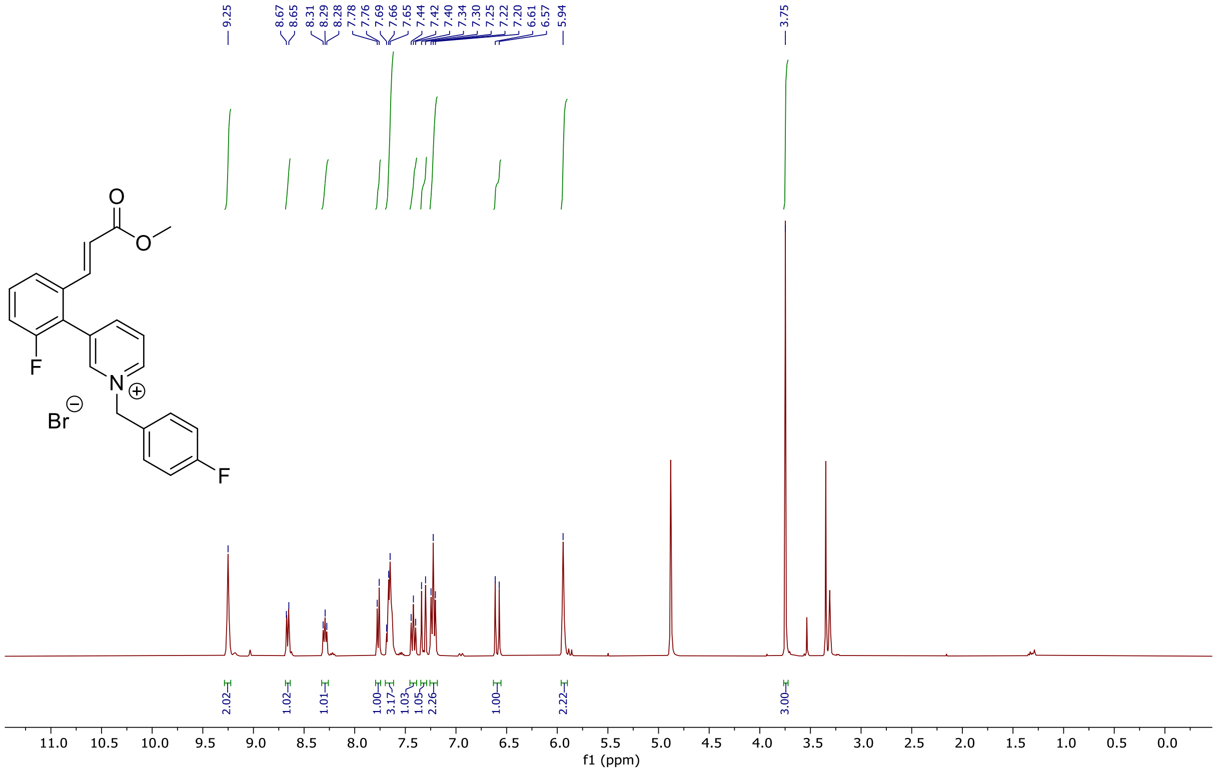

20201204-1810-B400_B.12-22.14.fid
Ref 480-7
Group Greaney_M
C13_CPD_Night256 MeOD /mnt/nmrdata/Greaney_M m31962tw 22

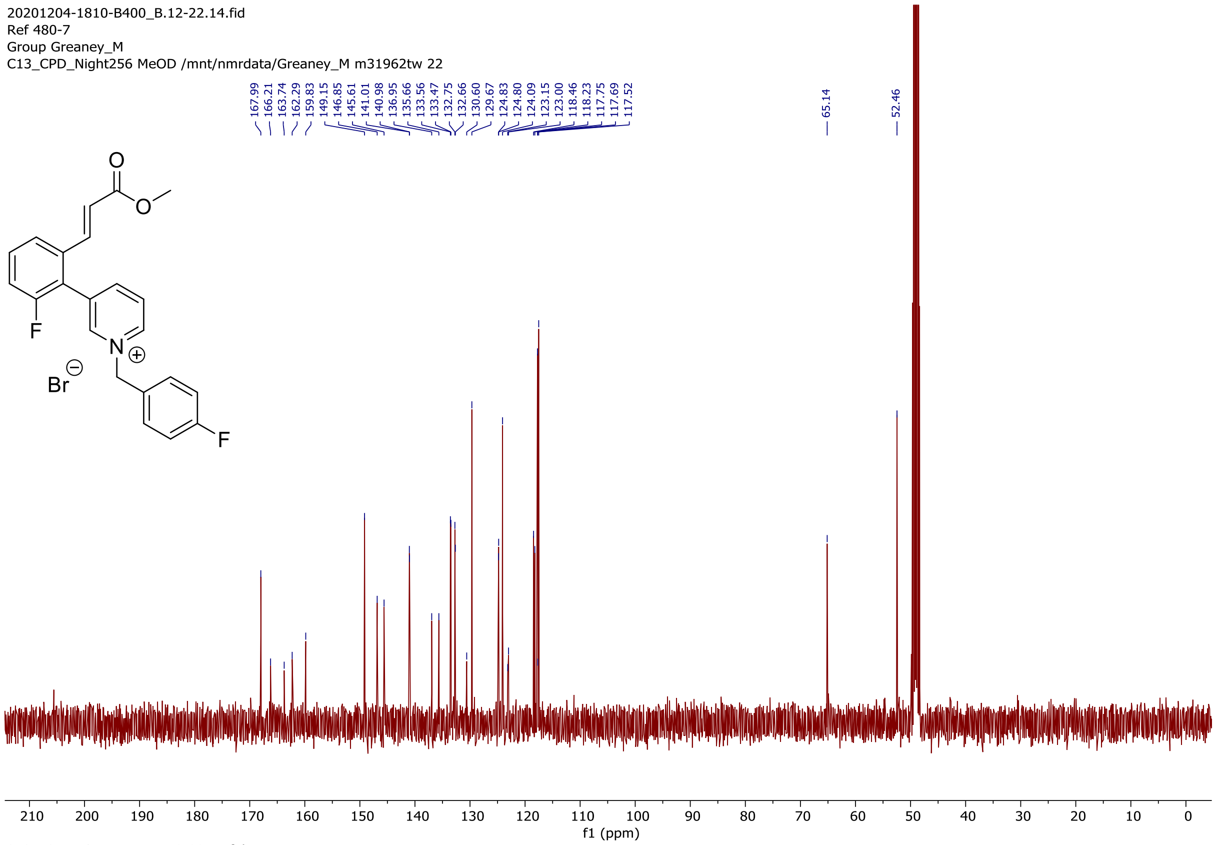

20201204-1810-B400_B.12-22.15.fid
Ref 480-7
Group Greaney_M
F19_CPD_Night MeOD /mnt/nmrdata/Greaney_M m31962tw 22

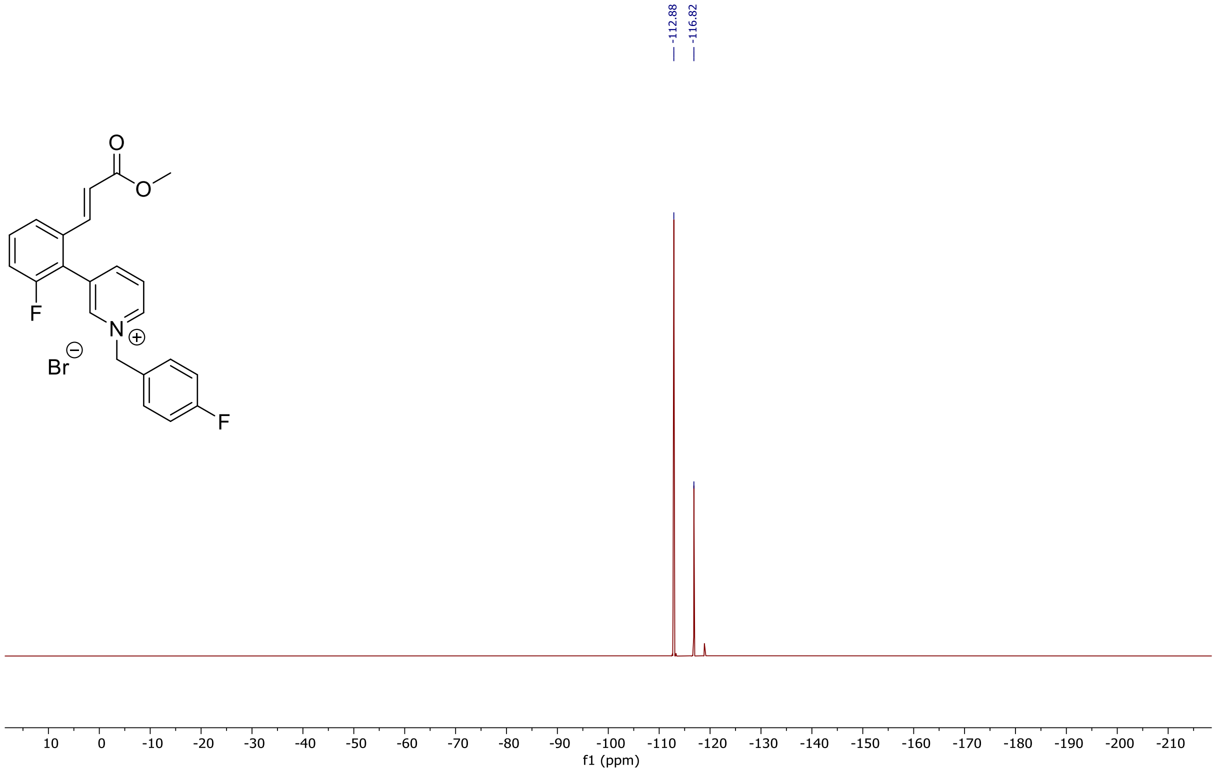

5k

20201204-1814-B400_B.12-16.10.fid
Ref 480-1
Group Greaney_M
H1_Night MeOD /mnt/nmrdata/Greaney_M m31962tw 16

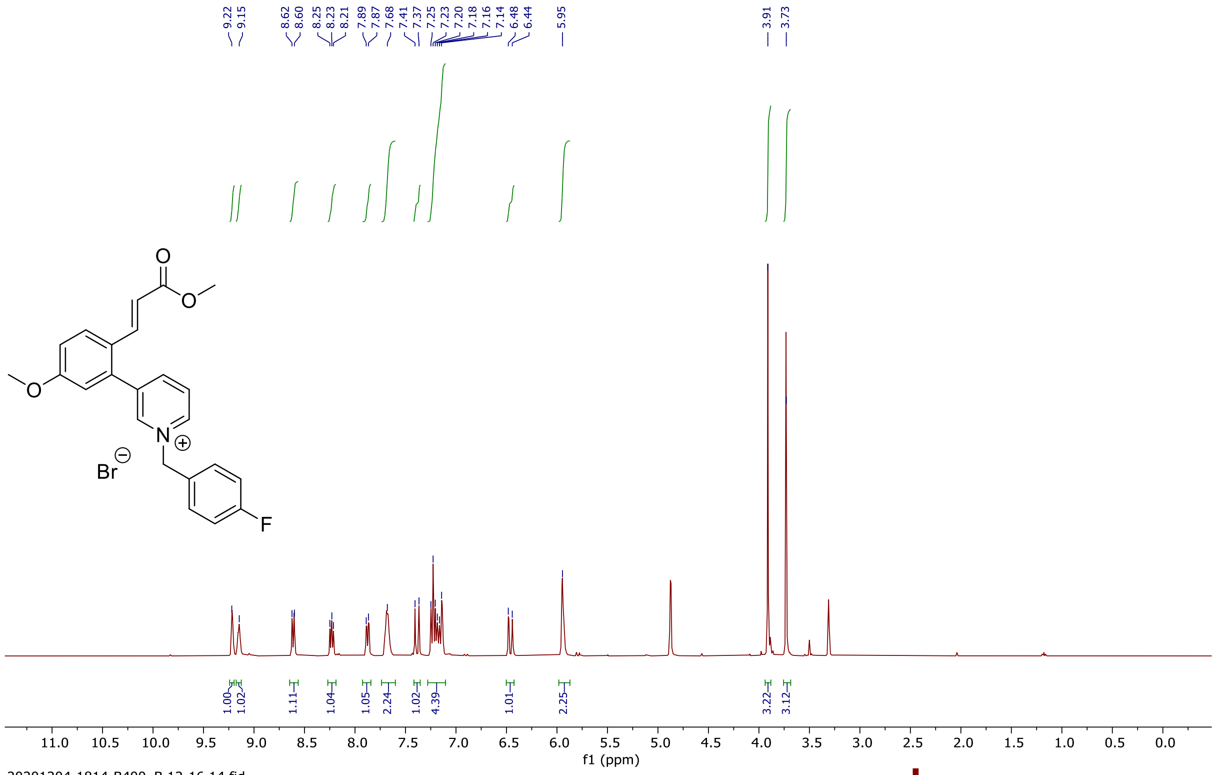

20201204-1814-B400_B.12-16.14.fid
Ref 480-1
Group Greaney_M
C13_CPD_Night256 MeOD /mnt/nmrdata/Greaney_M m31962tw 16

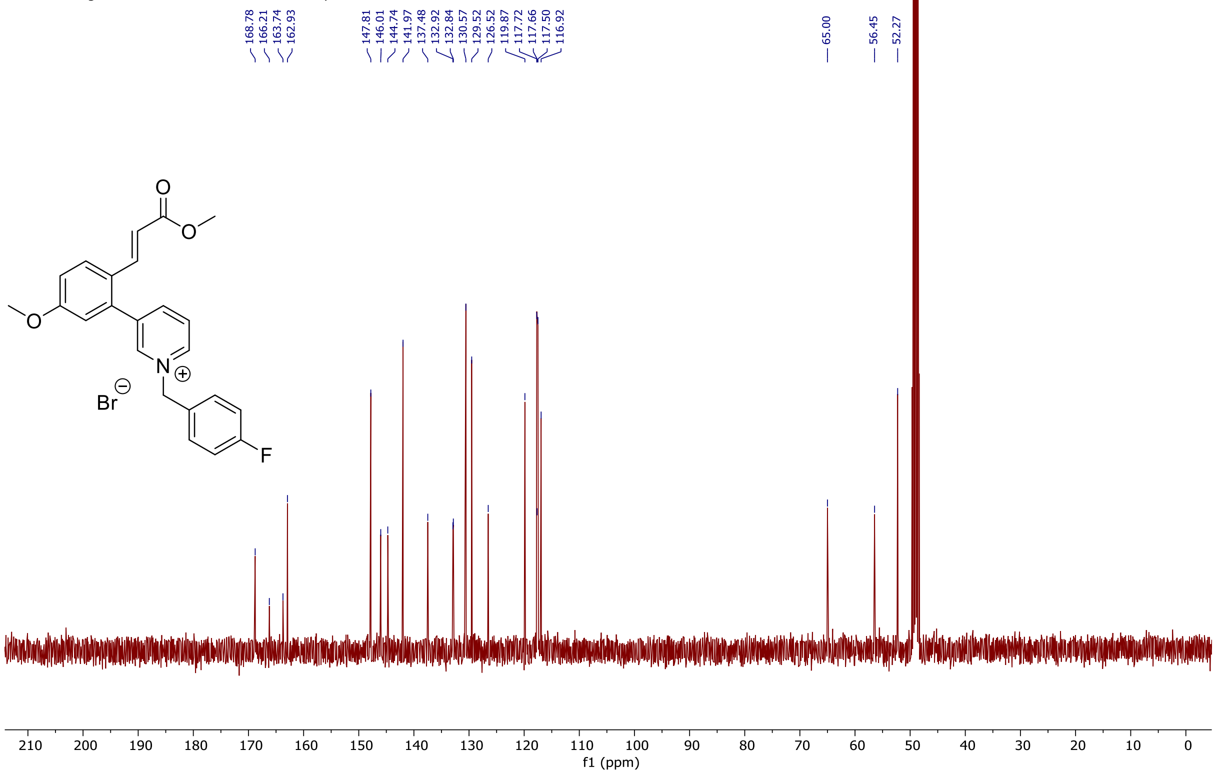

20201204-1814-B400_B.12-16.15.fid
Ref 480-1
Group Greaney_M
F19_CPD_Night MeOD /mnt/nmrdata/Greaney_M m31962tw 16

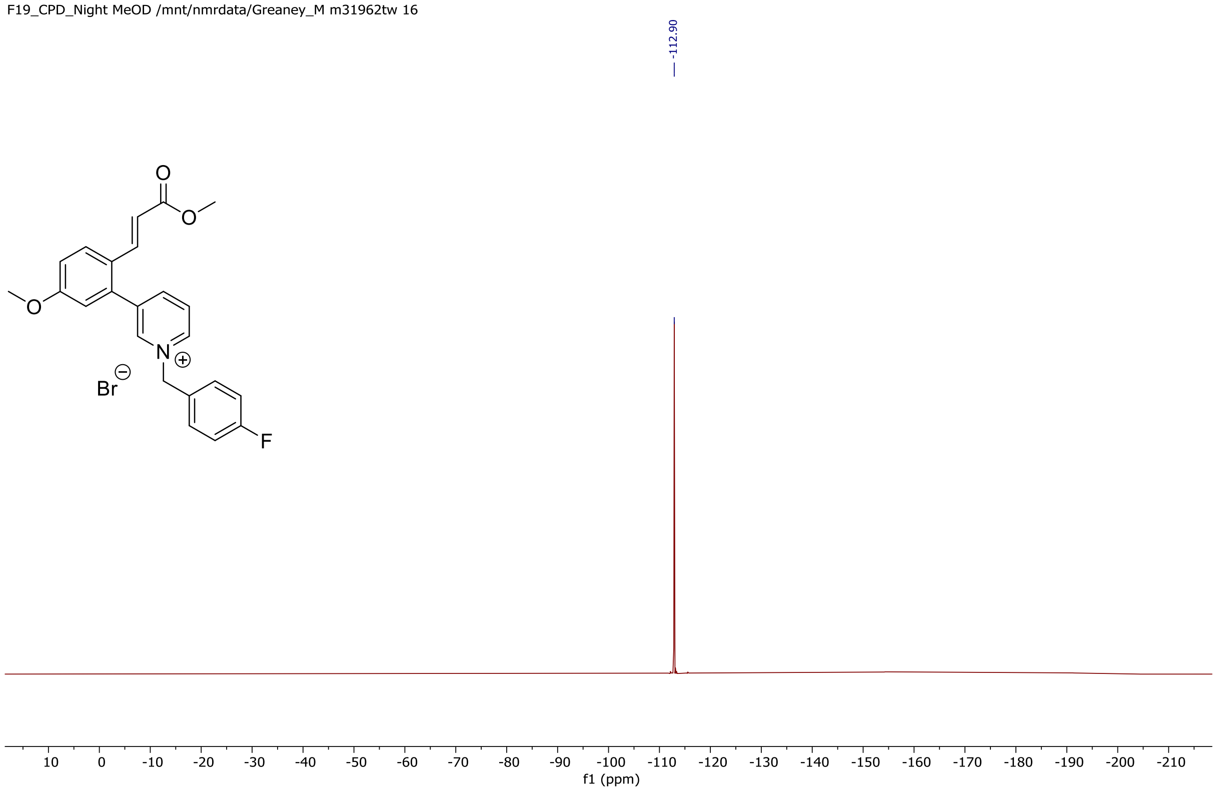

5l

20210505-1007-B400_B.11-27.10.fid
Ref 480-3
Group Greaney_M
H1_Night DMSO /mnt/nmrdata/Greaney_M m31962tw 27

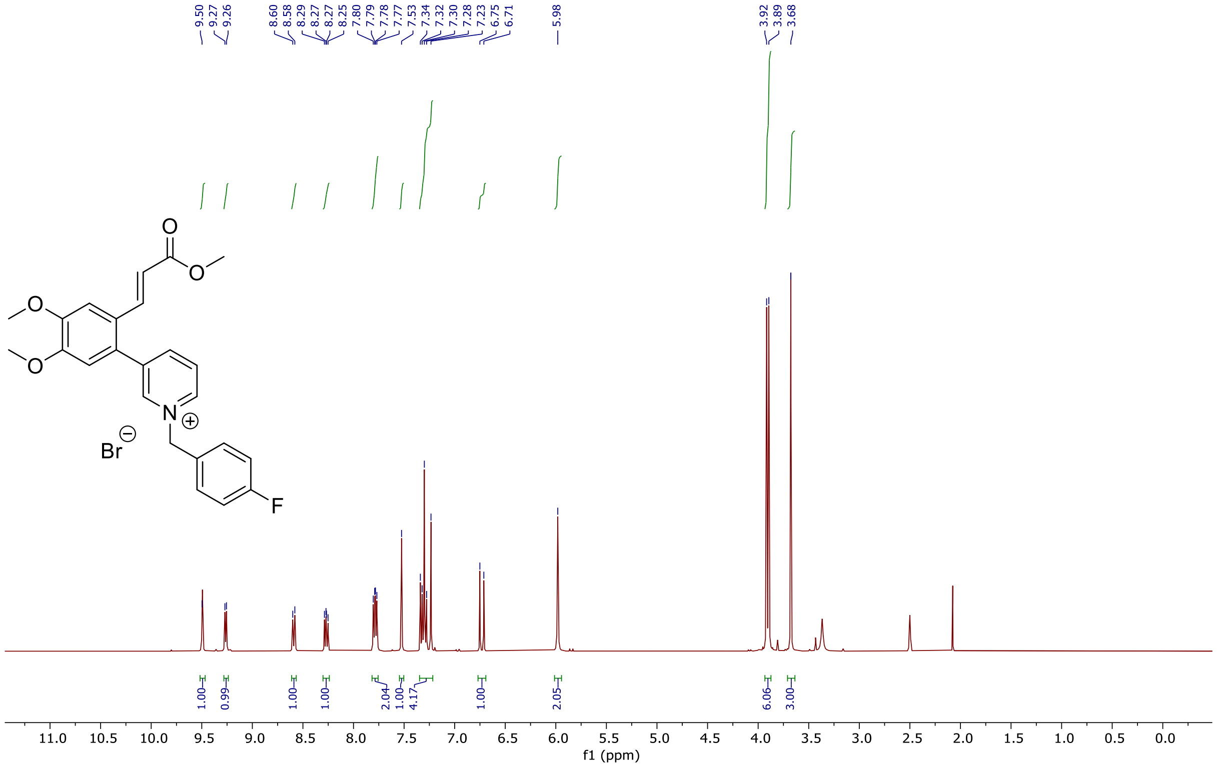

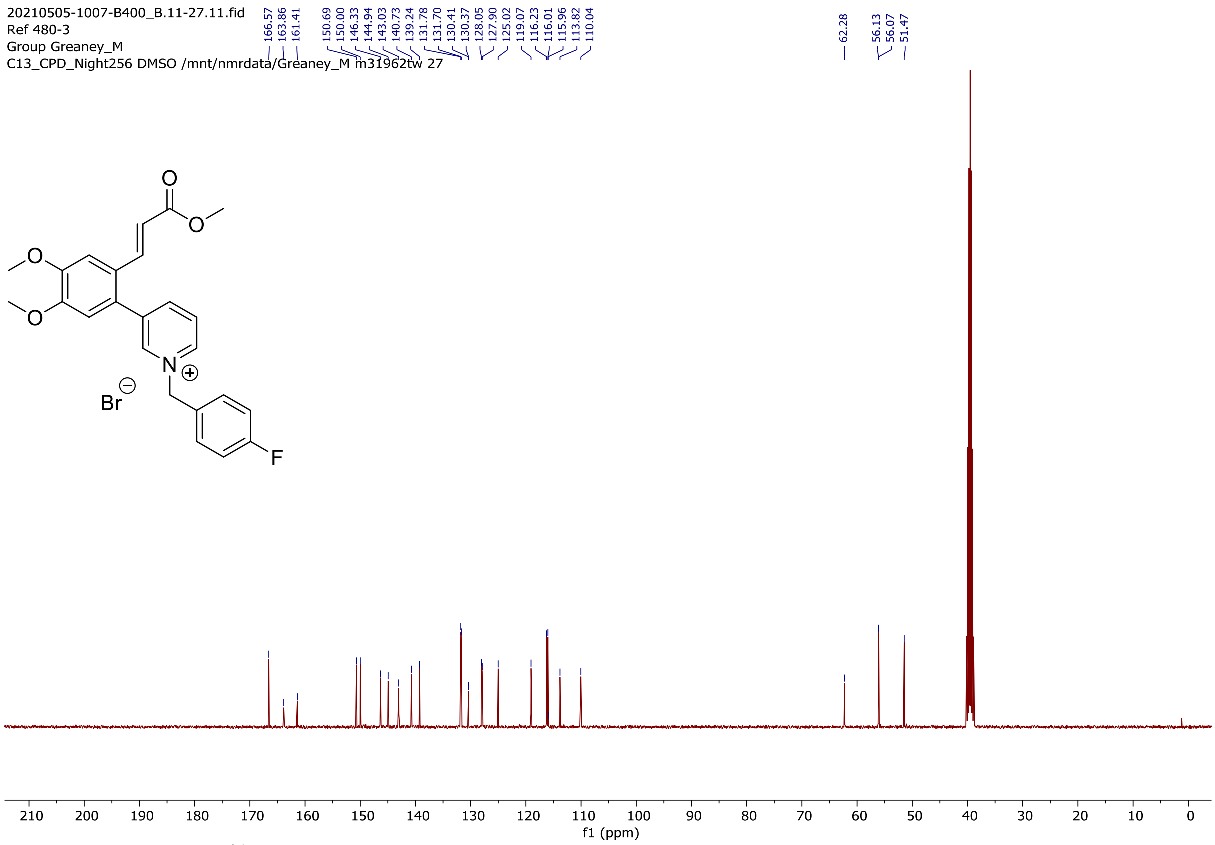
20210505-1007-B400_B.11-27.11.fid
Ref 480-3
Group Greaney_M
C13_CPD_Night256 DMSO /mnt/nmrdata/Greaney_M m31962tw 27
166.57
163.86
161.41
150.69
150.00
146.33
144.94
143.03
140.73
139.24
131.78
131.70
130.41
130.37
128.05
127.90
125.02
119.07
116.23
116.01
115.96
113.82
110.04
62.28
56.13
56.07
51.47
O
O
O
O
N
Br
F
f1 (ppm)

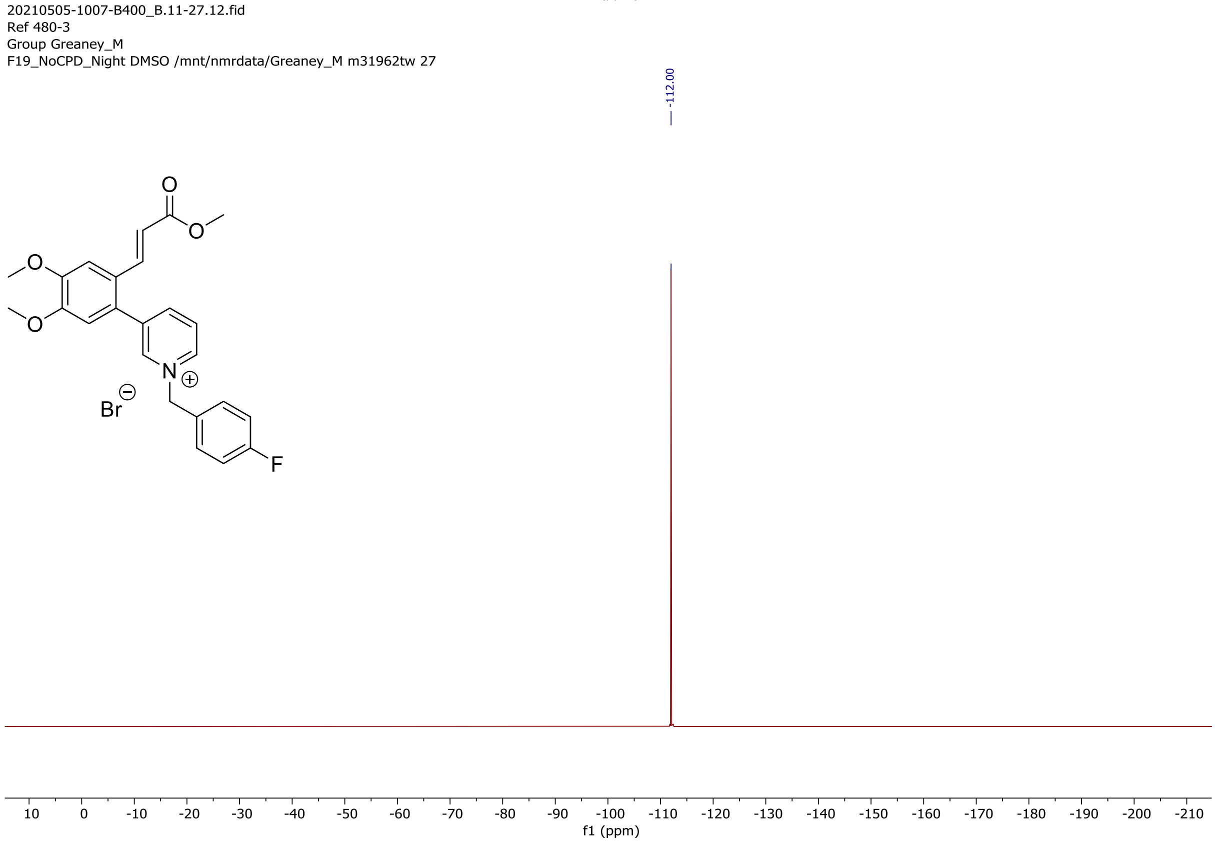
20210505-1007-B400_B.11-27.12.fid
Ref 480-3
Group Greaney_M
F19_NoCPD_Night DMSO /mnt/nmrdata/Greaney_M m31962tw 27
-112.00
O
O
O
O
N
Br
F
f1 (ppm)

5m

20201204-1811-B400_B.12-20.10.fid
Ref 480-5
Group Greaney_M
H1_Night MeOD /mnt/nmrdata/Greaney_M m31962tw 20

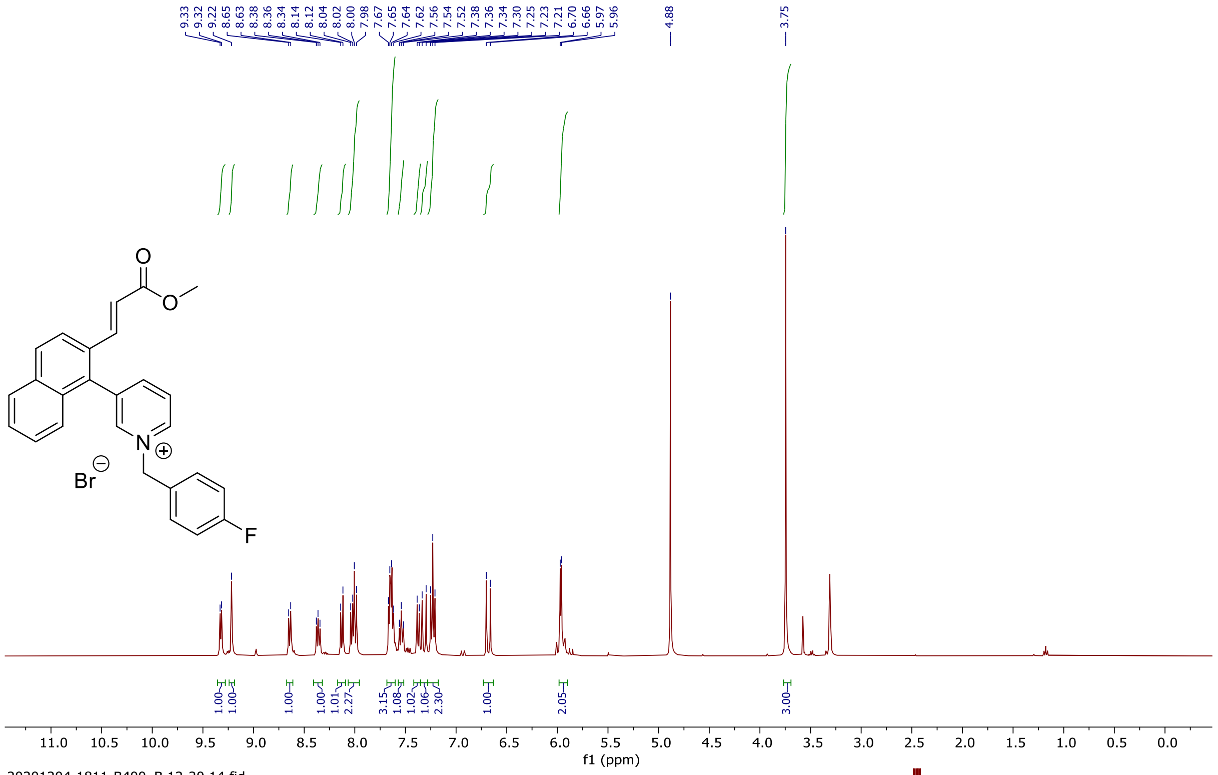

20201204-1811-B400_B.12-20.14.fid
Ref 480-5
Group Greaney_M
C13_CPD_Night256 MeOD /mnt/nmrdata/Greaney_M m31962tw 20

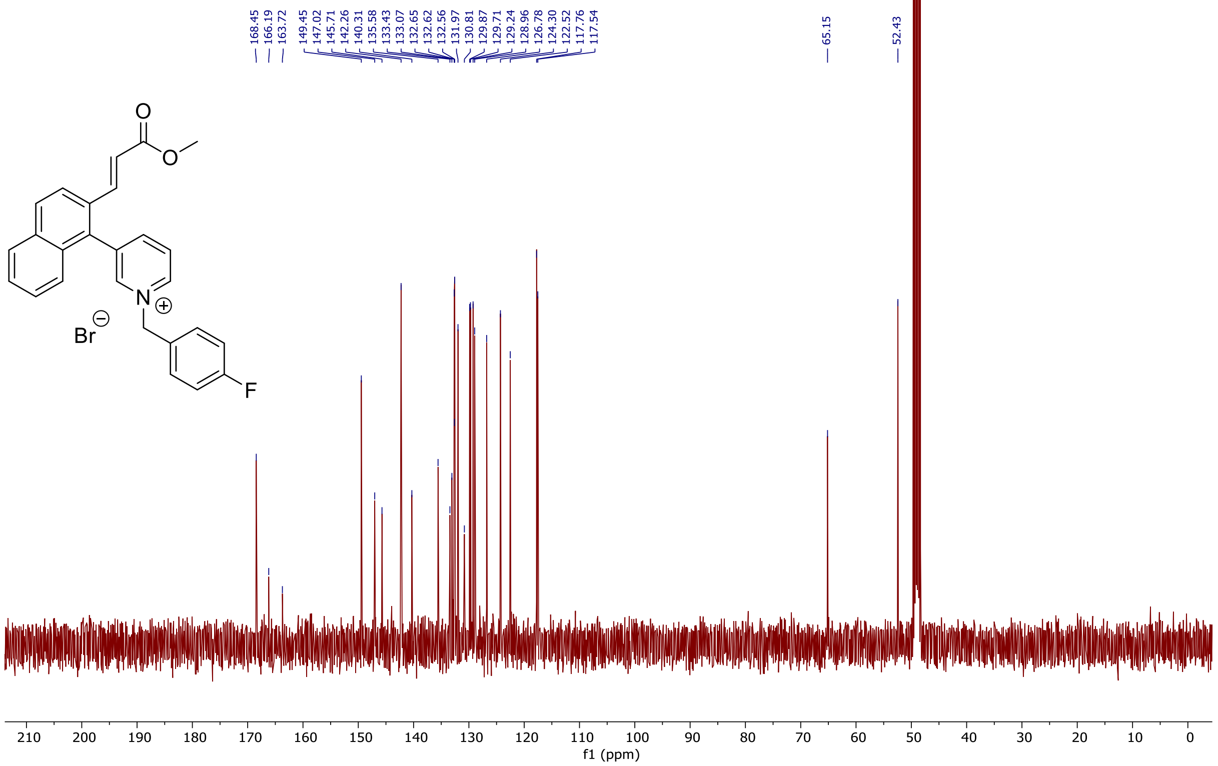

20201204-1811-B400_B.12-20.15.fid
Ref 480-5
Group Greaney_M
F19_CPD_Night MeOD /mnt/nmrdata/Greaney_M m31962tw 20

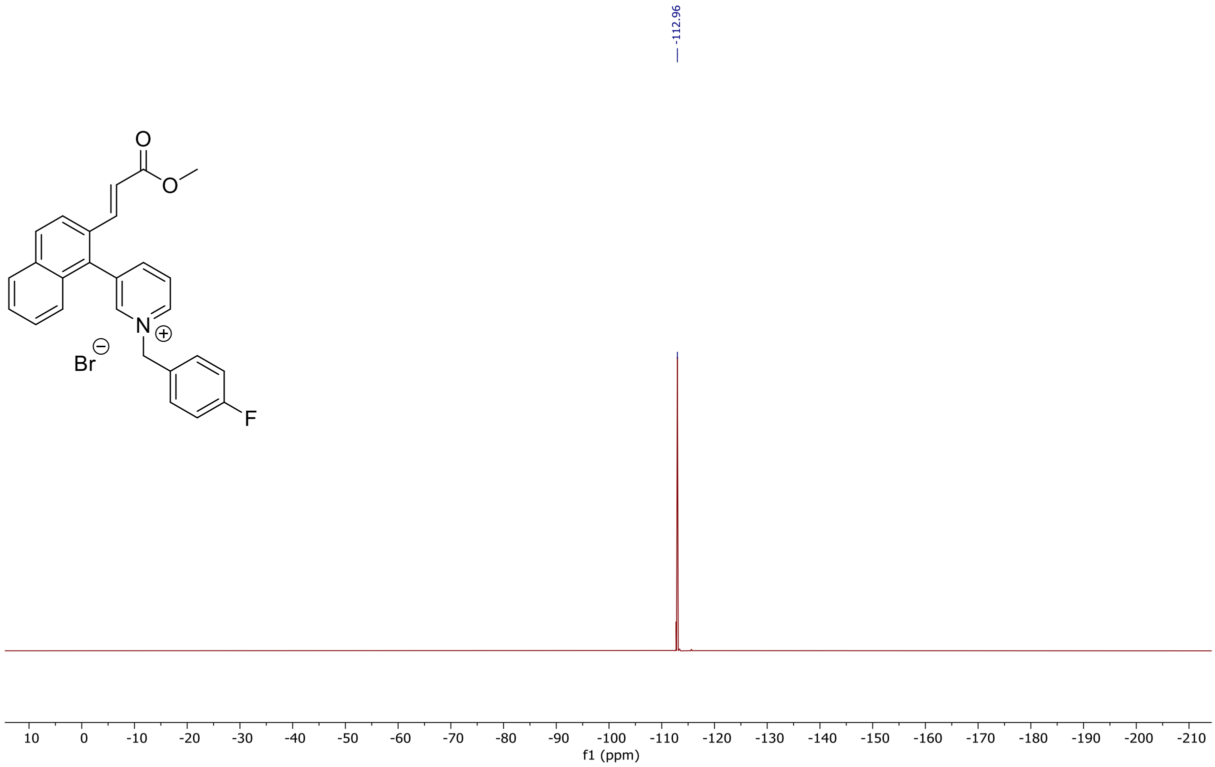

5n

20210409-1711-B400_B.11-5.10.fid
Ref 546-2
Group Greaney_M
H1_Night MeOD /mnt/nmrdata/Greaney_M m31962tw 5

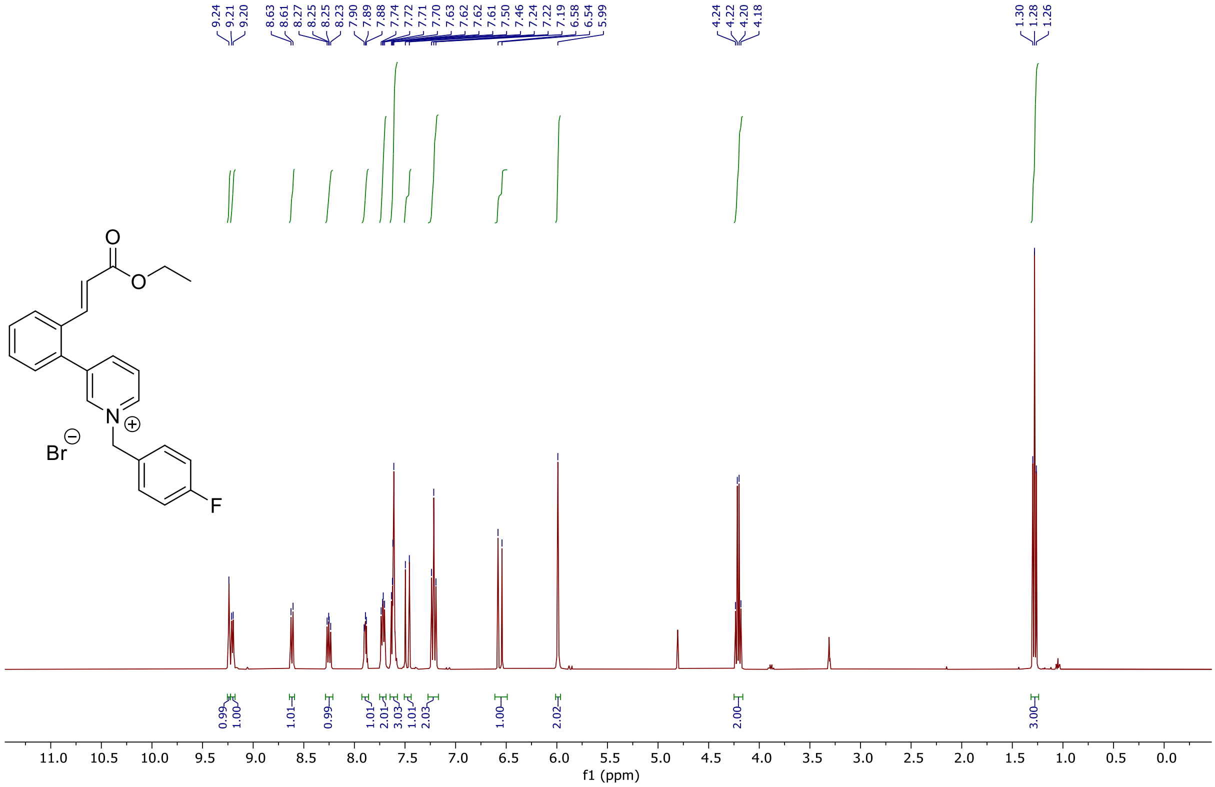

20210409-1711-B400_B.11-5.11.fid
Ref 546-2
Group Greaney_M
C13_CPD_Night256 MeOD /mnt/nmrdata/Greaney_M m31962tw 5

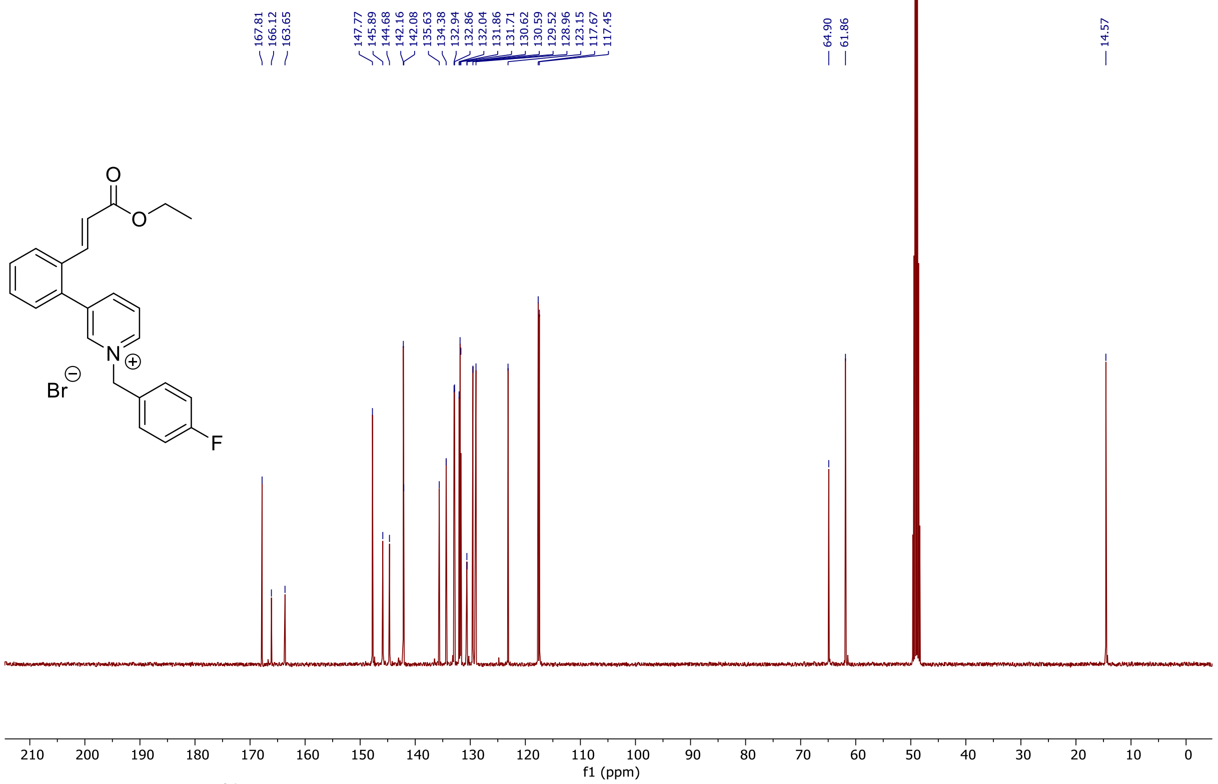

20210409-1711-B400_B.11-5.15.fid
Ref 546-2
Group Greaney_M
F19_NoCPD_Night MeOD /mnt/nmrdata/Greaney_M m31962tw 5

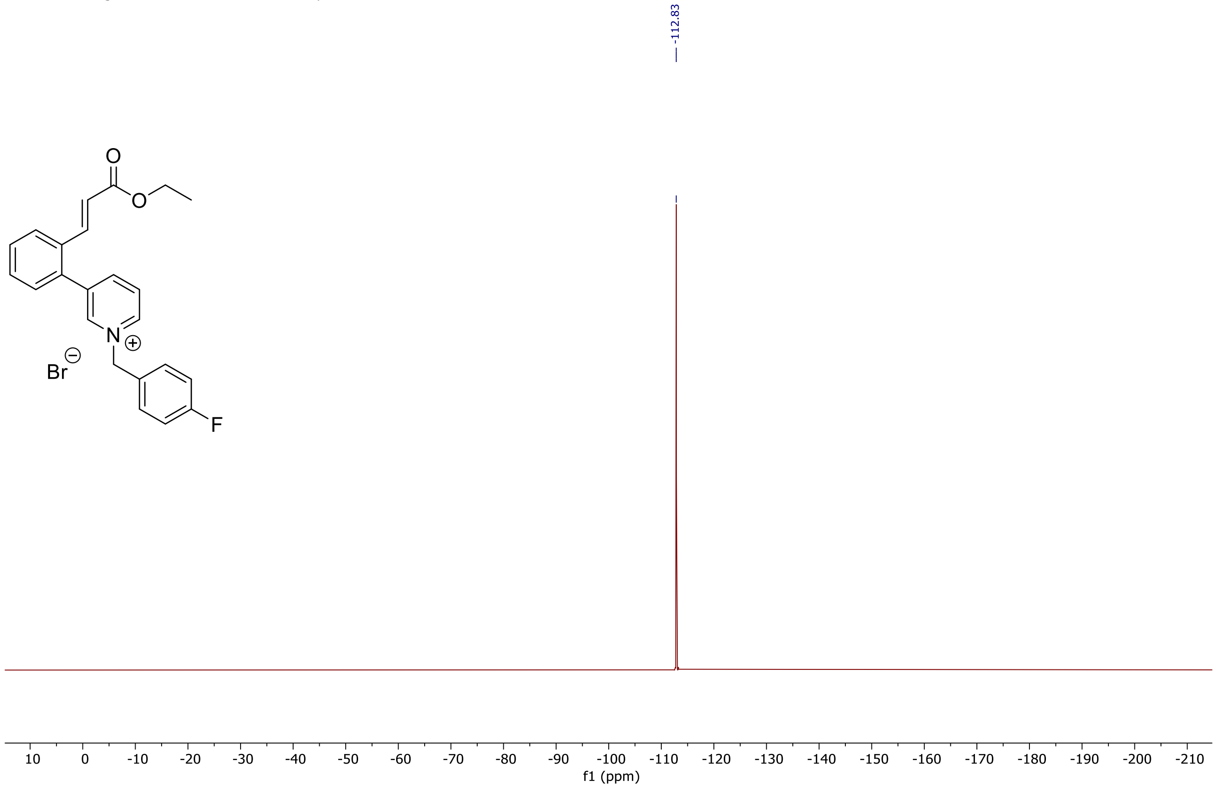

5o

20210409-1710-B400_B.11-4.10.fid
Ref 546-1
Group Greaney_M
H1_Night MeOD /mnt/nmrdata/Greaney_M m31962tw 4

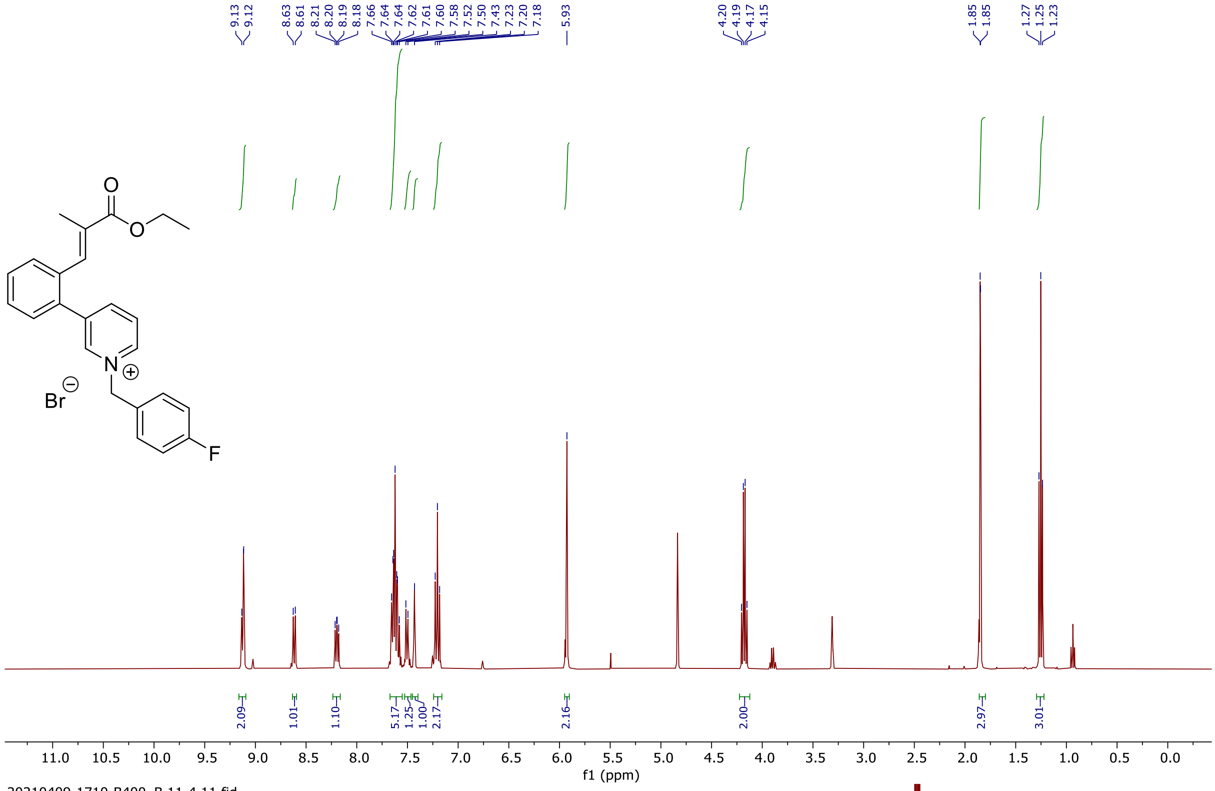

20210409-1710-B400_B.11-4.11.fid
Ref 546-1
Group Greaney_M
C13_CPD_Night256 MeOD /mnt/nmrdata/Greaney_M m31962tw 4

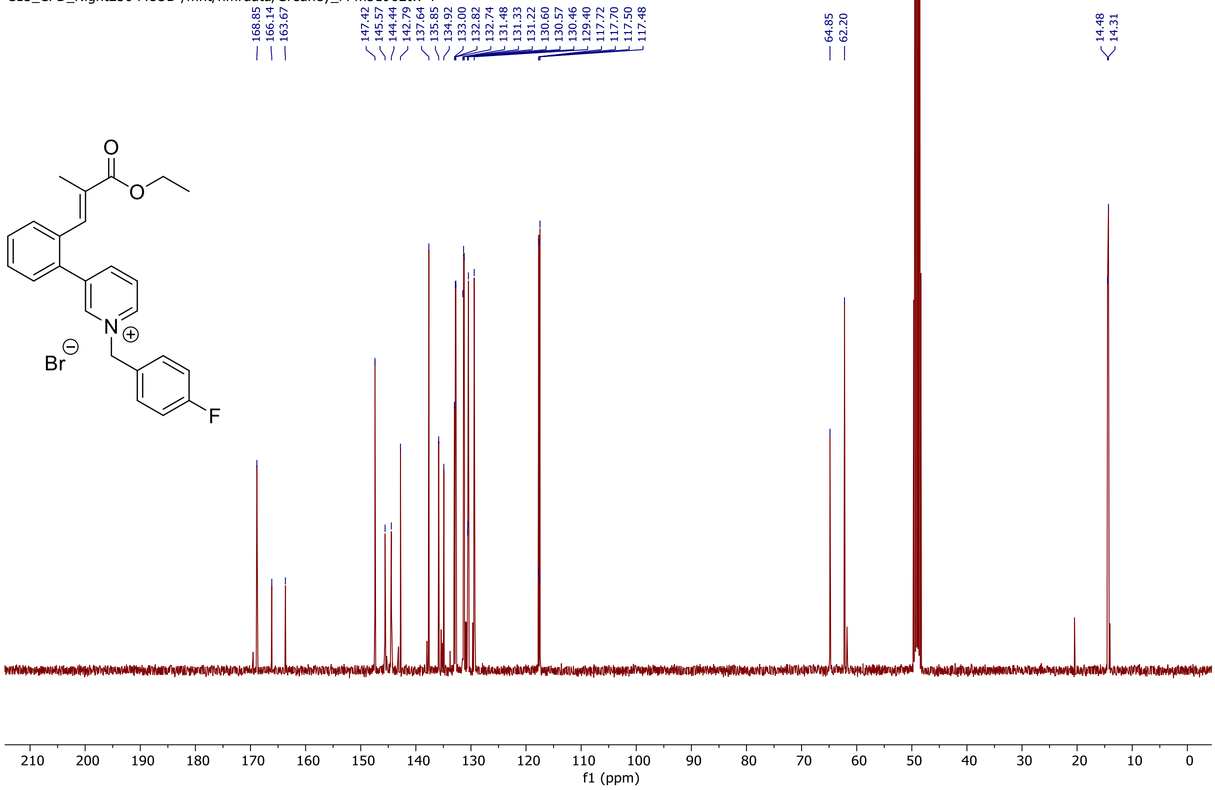

20210409-1710-B400_B.11-4.15.fid
Ref 546-1
Group Greaney_M
F19_NoCPD_Night MeOD /mnt/nmrdata/Greaney_M m31962tw 4

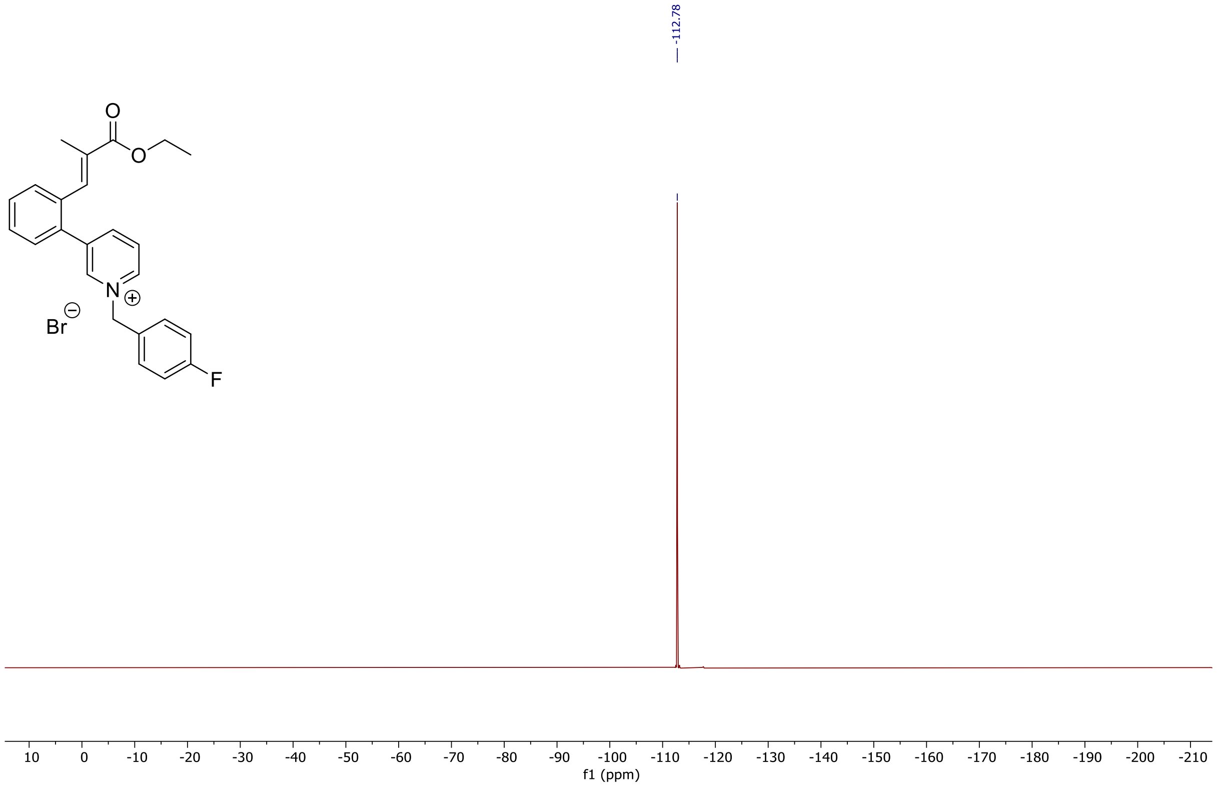

5p

20210416-1514-B400_B.11-37.10.fid
Ref 540-6
Group Greaney_M
H1_Night None /mnt/nmrdata/Greaney_M m31962tw 37

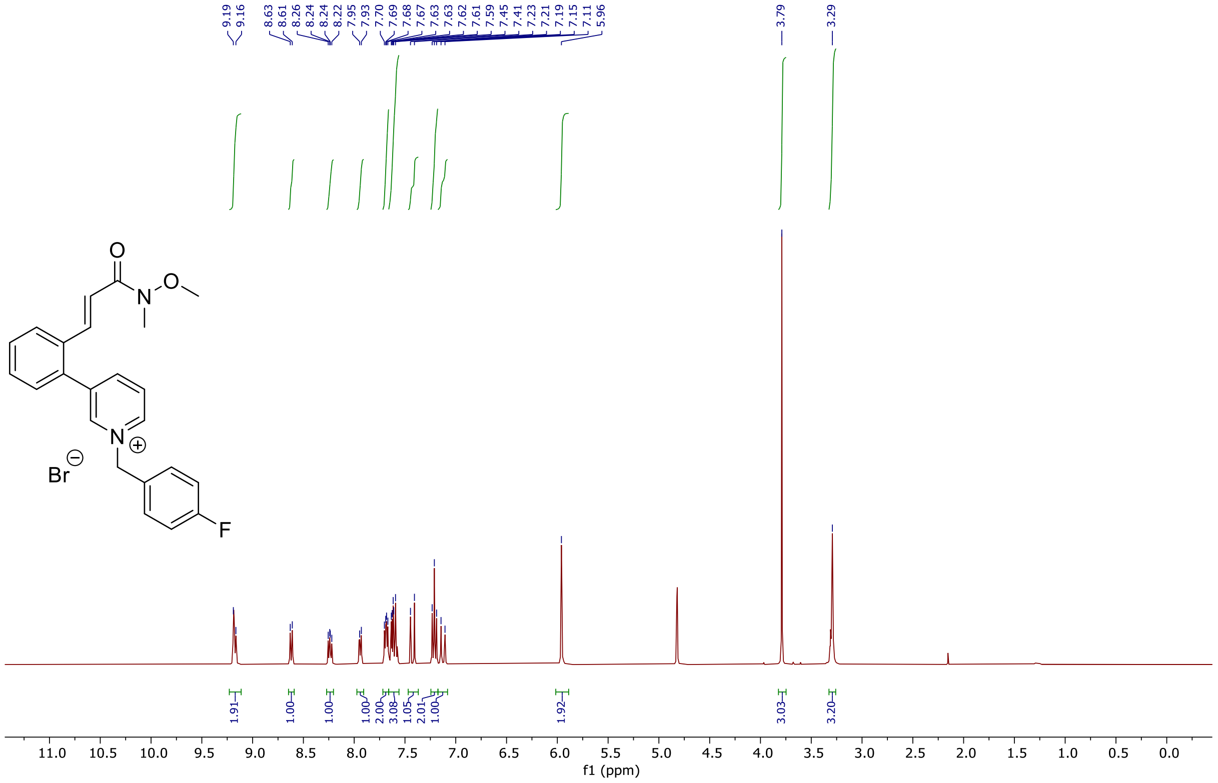

20210416-1514-B400_B.11-37.11.fid
Ref 540-6
Group Greaney_M
C13_CPD_Night256 None /mnt/nmrdata/Greaney_M m31962tw 37

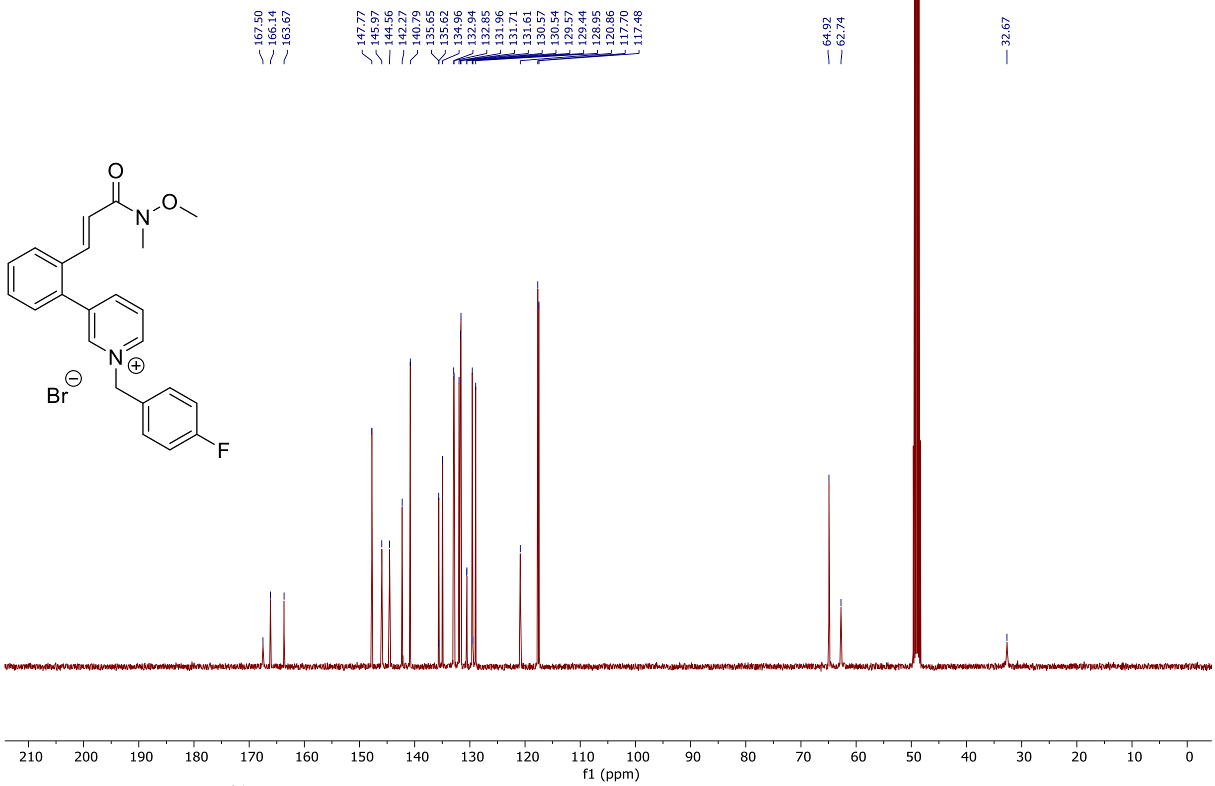

20210416-1514-B400_B.11-37.15.fid
Ref 540-6
Group Greaney_M
F19_NoCPD_Night None /mnt/nmrdata/Greaney_M m31962tw 37

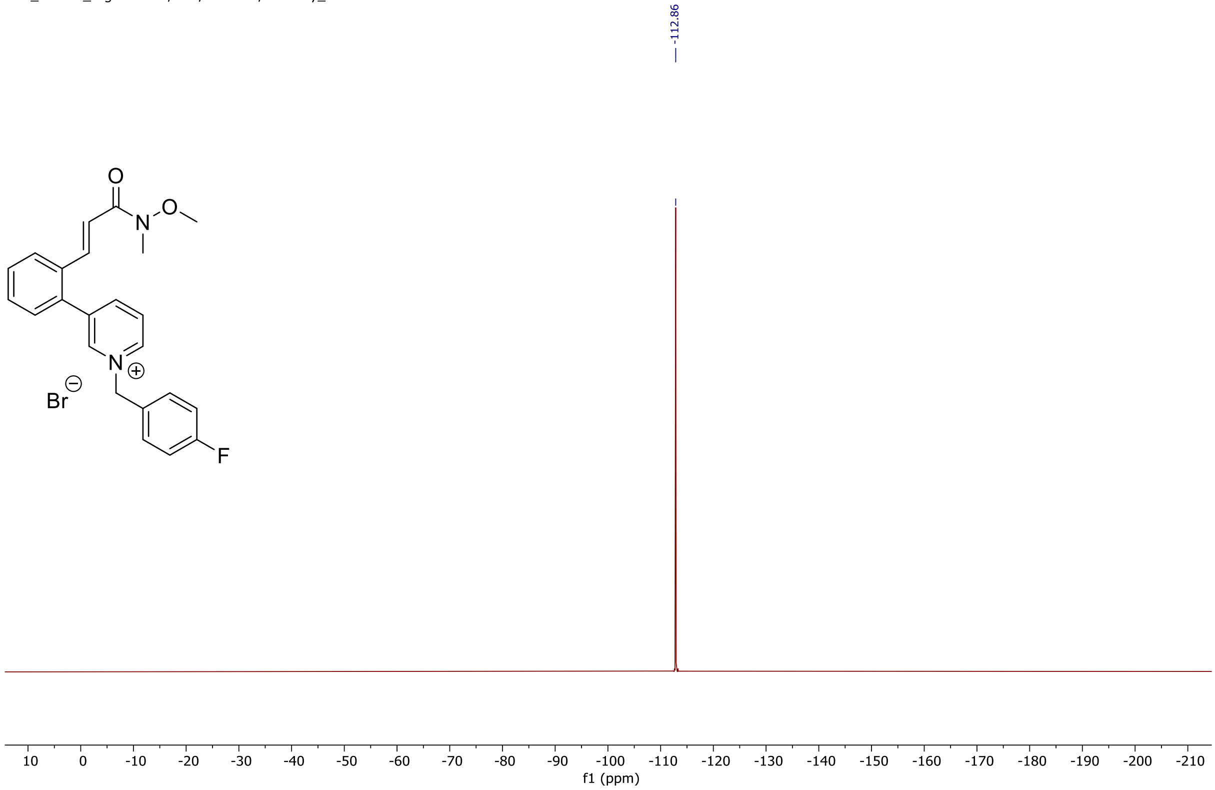

5q

20210409-1740-B500_B.14-41.10.fid
Ref 494-3
Group Greaney_M
H1_Night MeOD /mnt/nmrdata/Greaney_M m31962tw 41

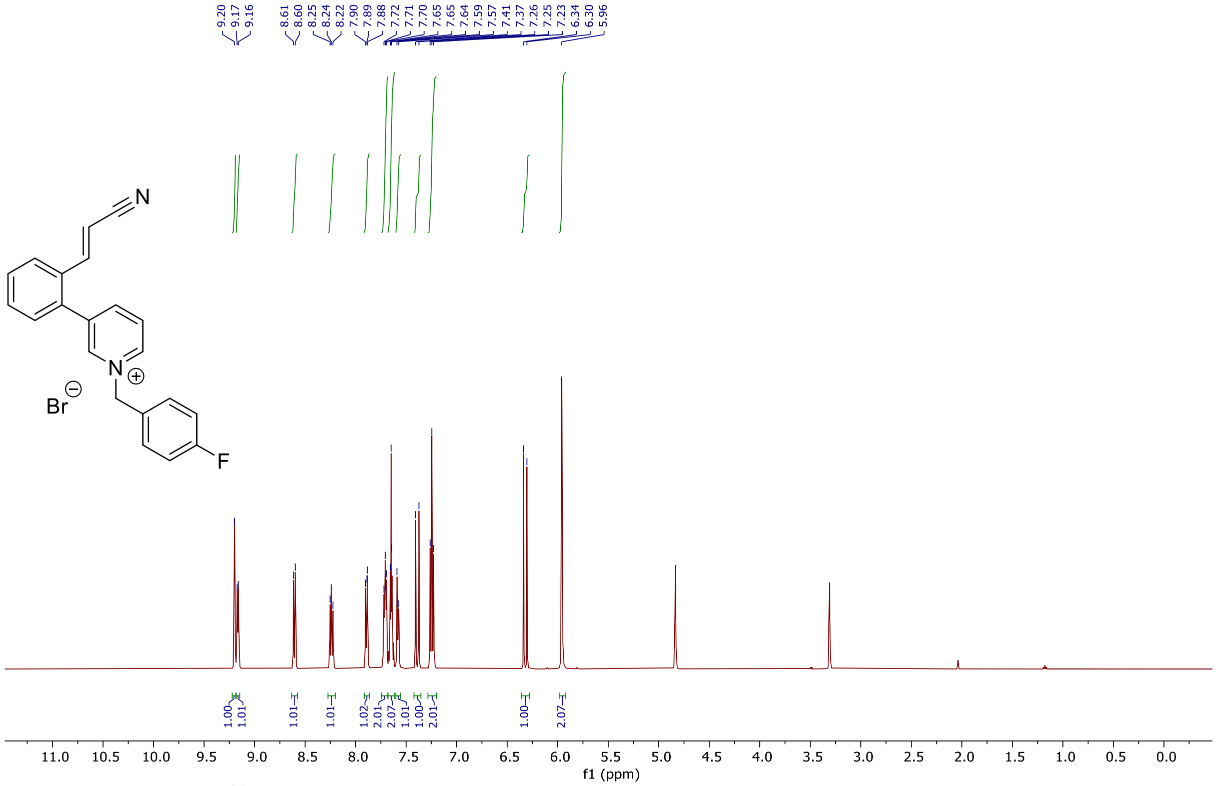

20210409-1740-B500_B.14-41.11.fid
Ref 494-3
Group Greaney_M
C13_CPD_Night256 MeOD /mnt/nmrdata/Greaney_M m31962tw 41

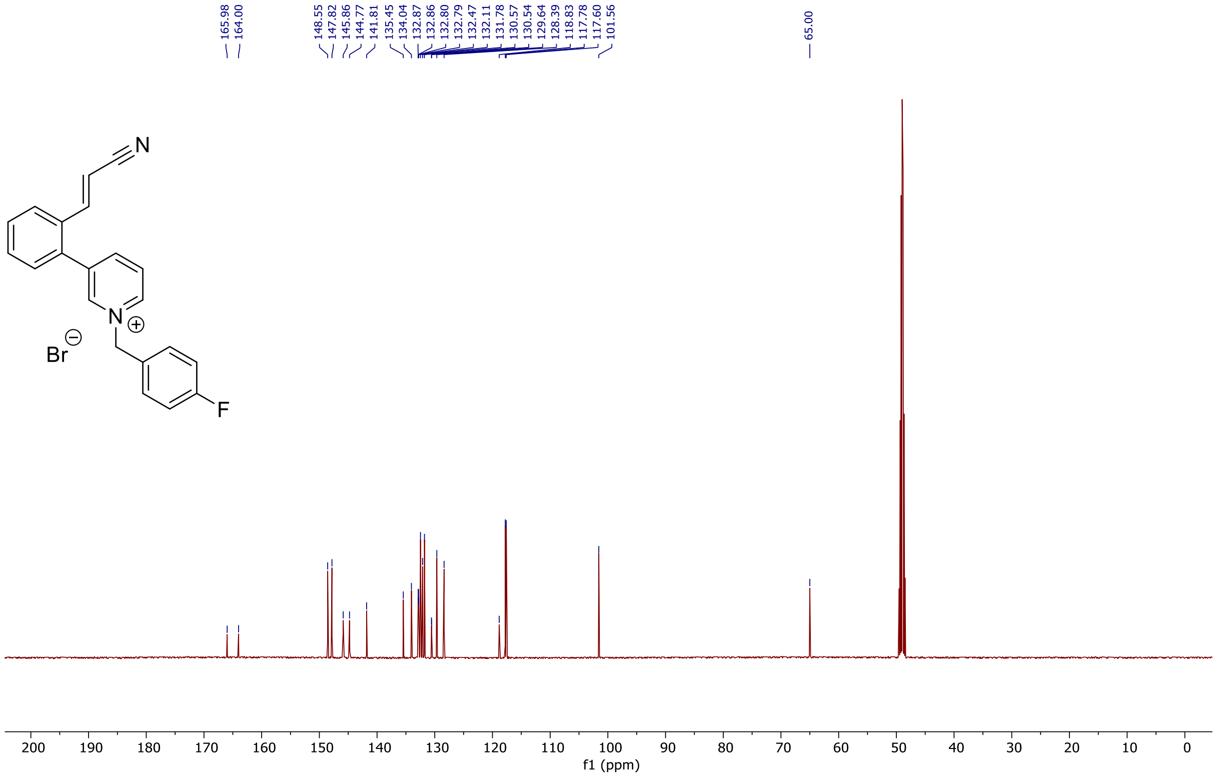

20210409-1740-B500_B.14-41.16.fid
Ref 494-3
Group Greaney_M
F19_NoCPD_Night MeOD /mnt/nmrdata/Greaney_M m31962tw 41

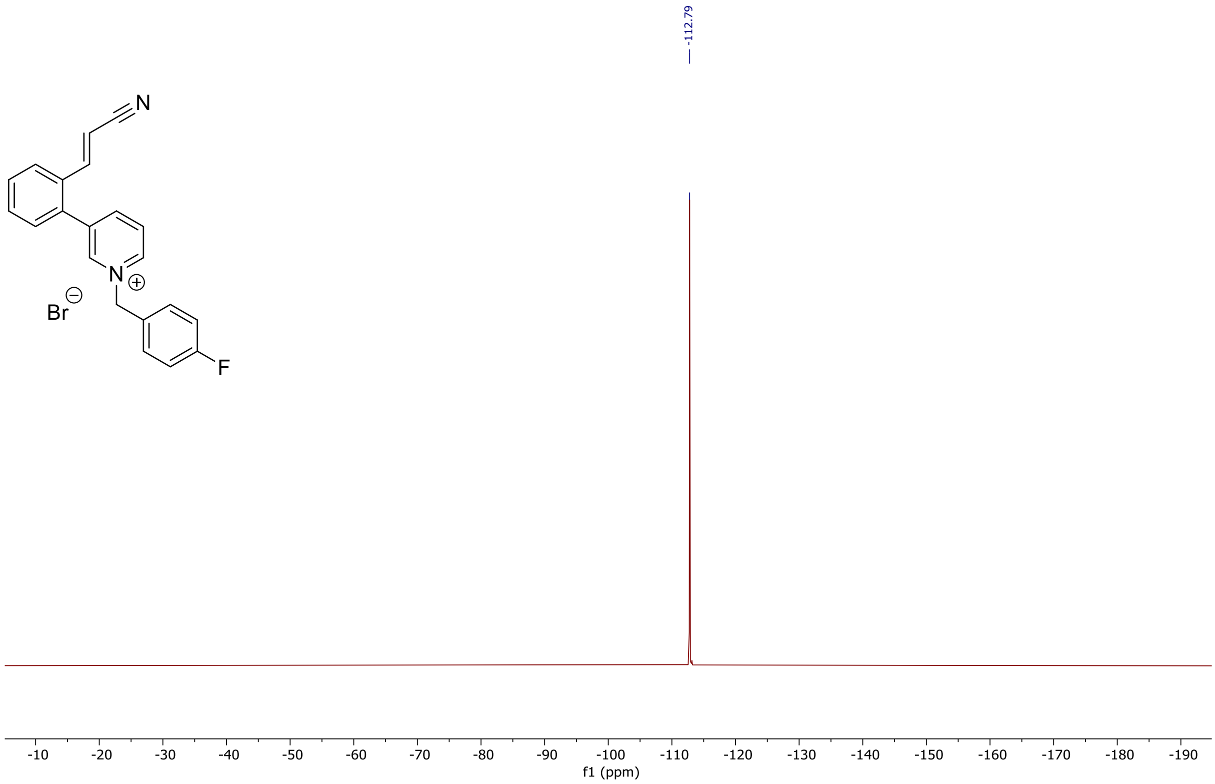

5r

20210409-1708-B400_B.11-59.10.fid
Ref 500-8
Group Greaney_M
H1_Night MeOD /mnt/nmrdata/Greaney_M m31962tw 59

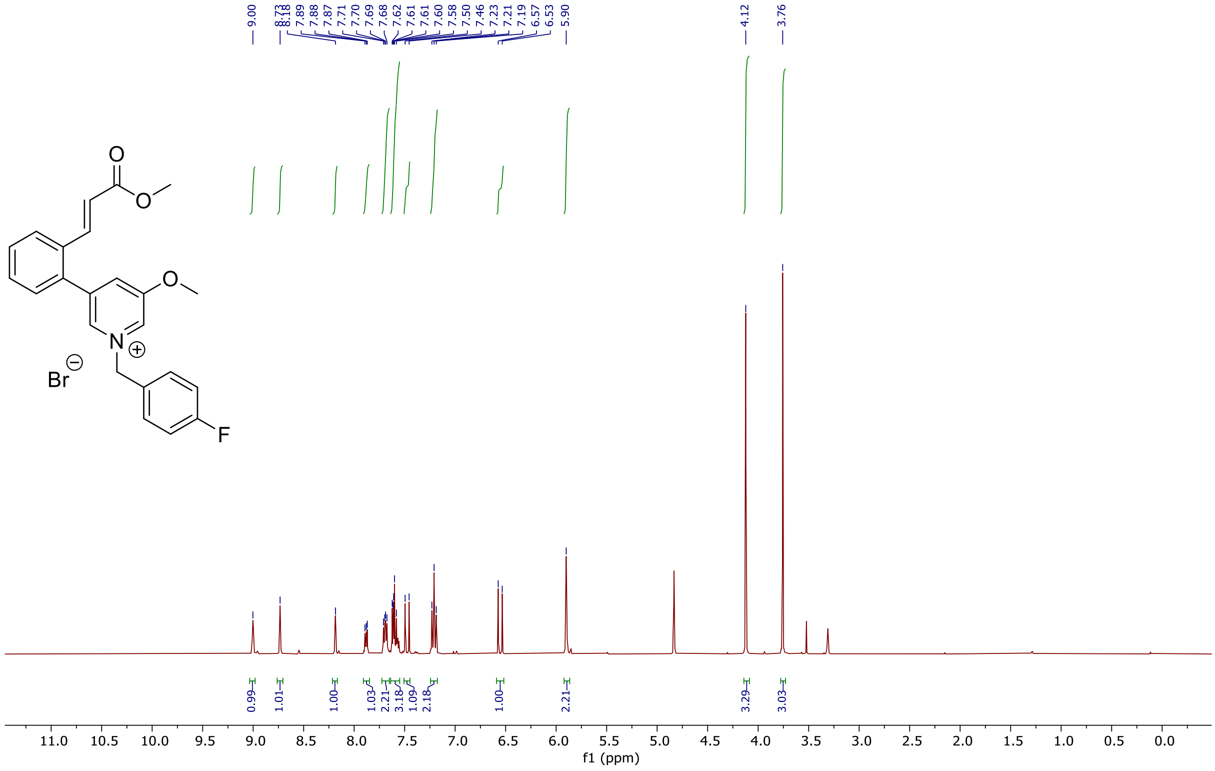

20210409-1708-B400_B.11-59.11.fid
Ref 500-8
Group Greaney_M
C13_CPD_Night256 MeOD /mnt/nmrdata/Greaney_M m31962tw 59

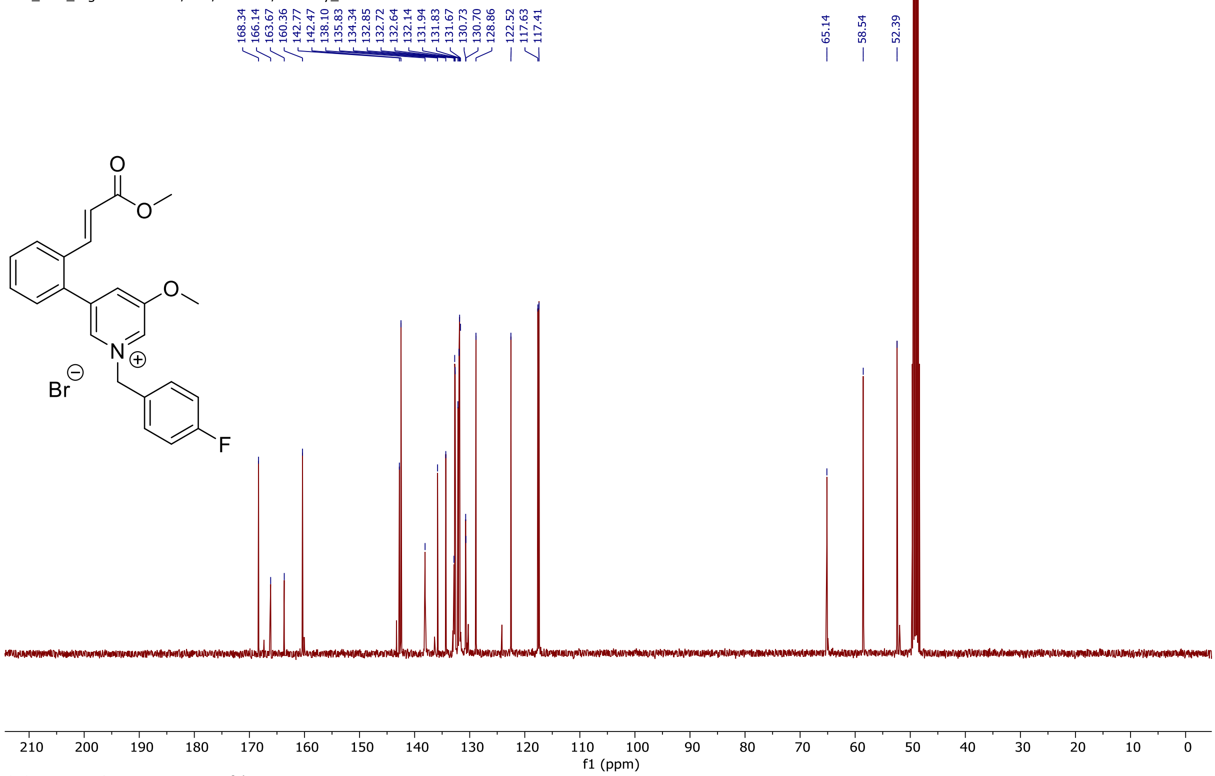

20210409-1708-B400_B.11-59.15.fid
Ref 500-8
Group Greaney_M
F19_NoCPD_Night MeOD /mnt/nmrdata/Greaney_M m31962tw 59

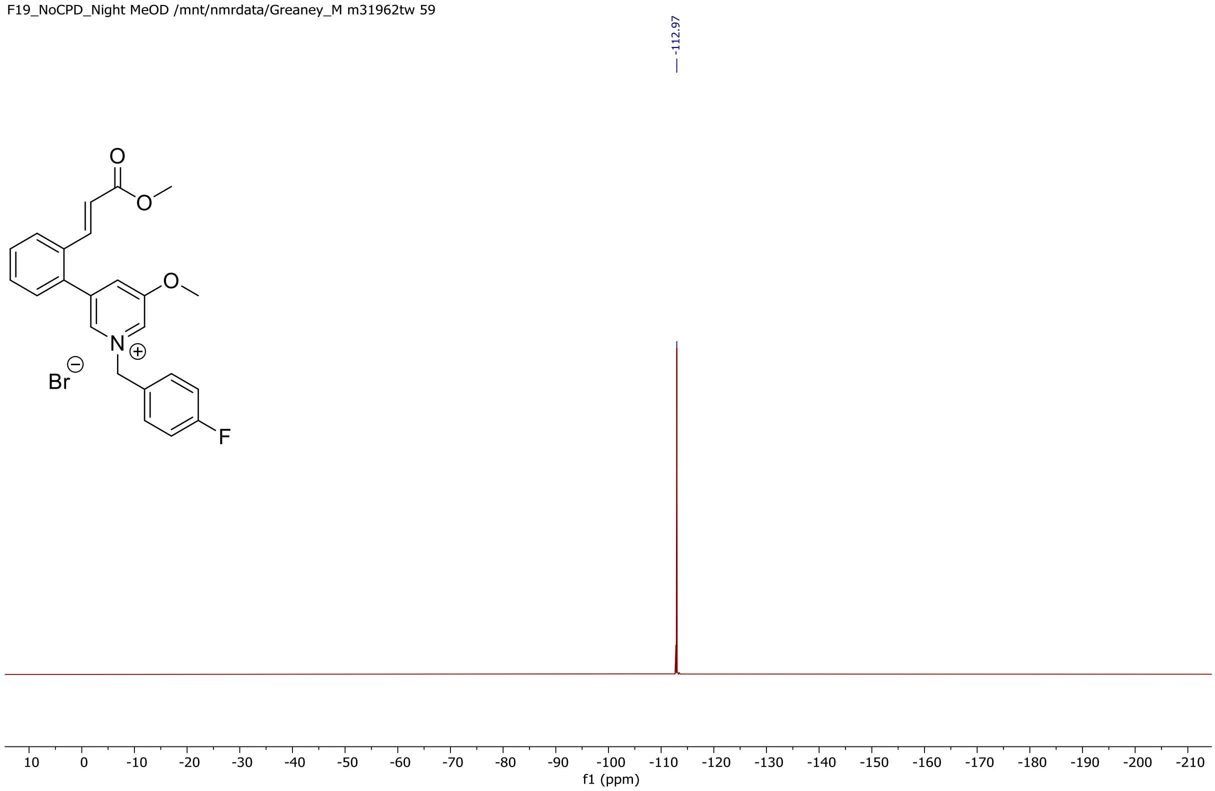

5s

20210409-1709-B400_B.11-1.10.fid
Ref 532-2
Group Greaney_M
H1_Night MeOD /mnt/nmrdata/Greaney_M m31962tw 1

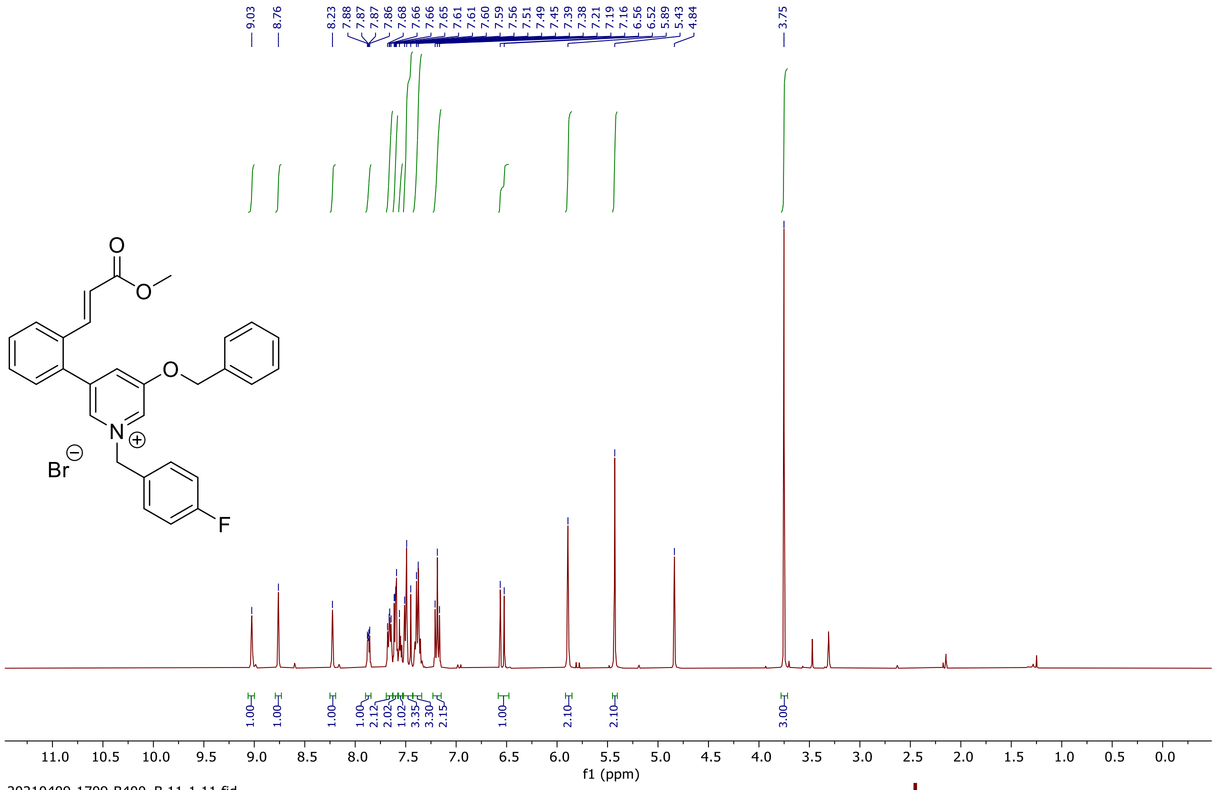

20210409-1709-B400_B.11-1.11.fid
Ref 532-2
Group Greaney_M
C13_CPD_Night256 MeOD /mnt/nmrdata/Greaney_M m31962tw 1

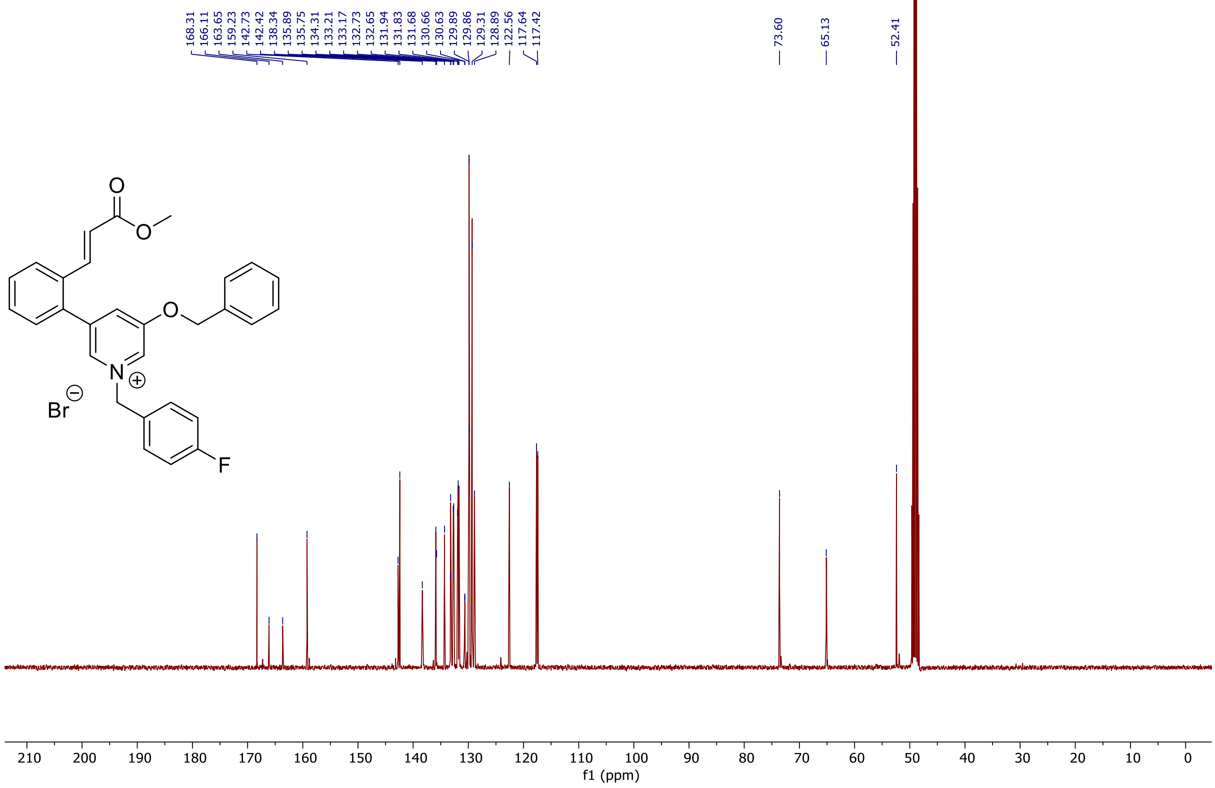

20210409-1709-B400_B.11-1.15.fid
Ref 532-2
Group Greaney_M
F19_NoCPD_Night MeOD /mnt/nmrdata/Greaney_M m31962tw 1

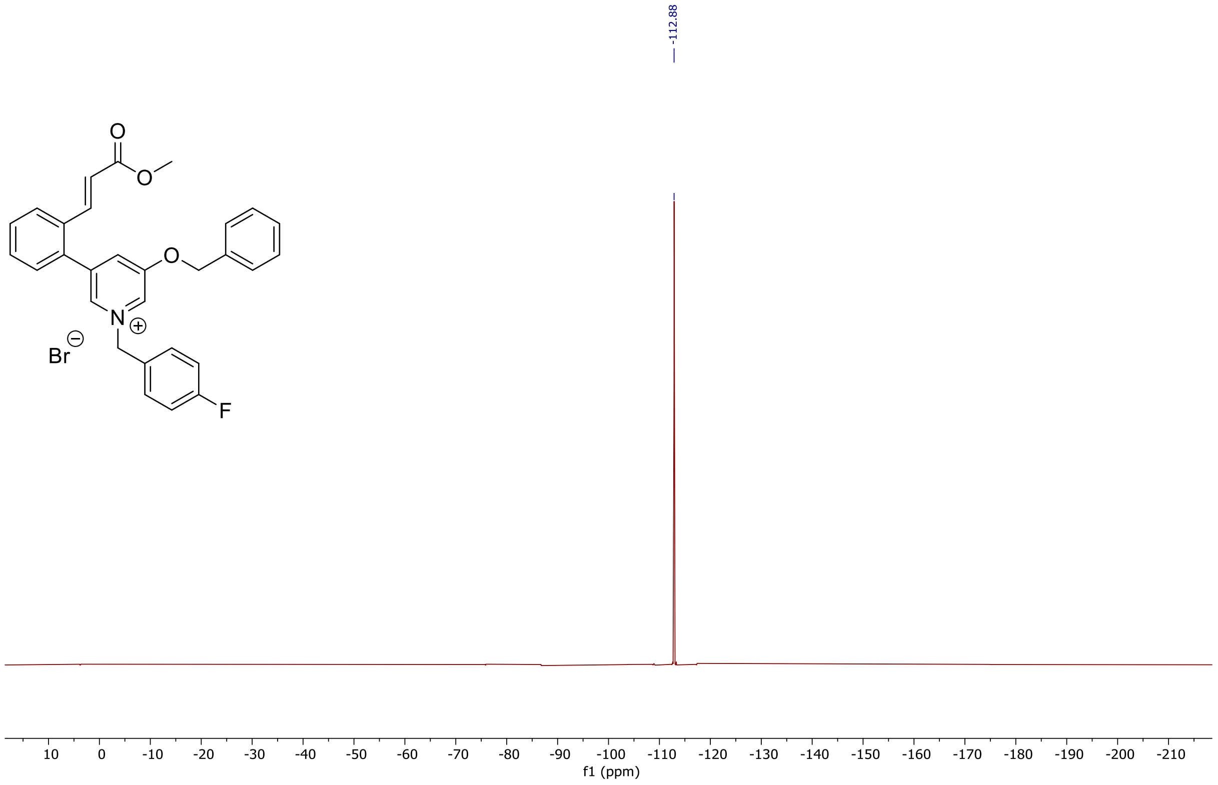

5t

20210416-1659-B500_B.14-19.10.fid
Ref 532-3
Group Greaney_M
H1_Night MeOD /mnt/nmrdata/Greaney_M m31962tw 19

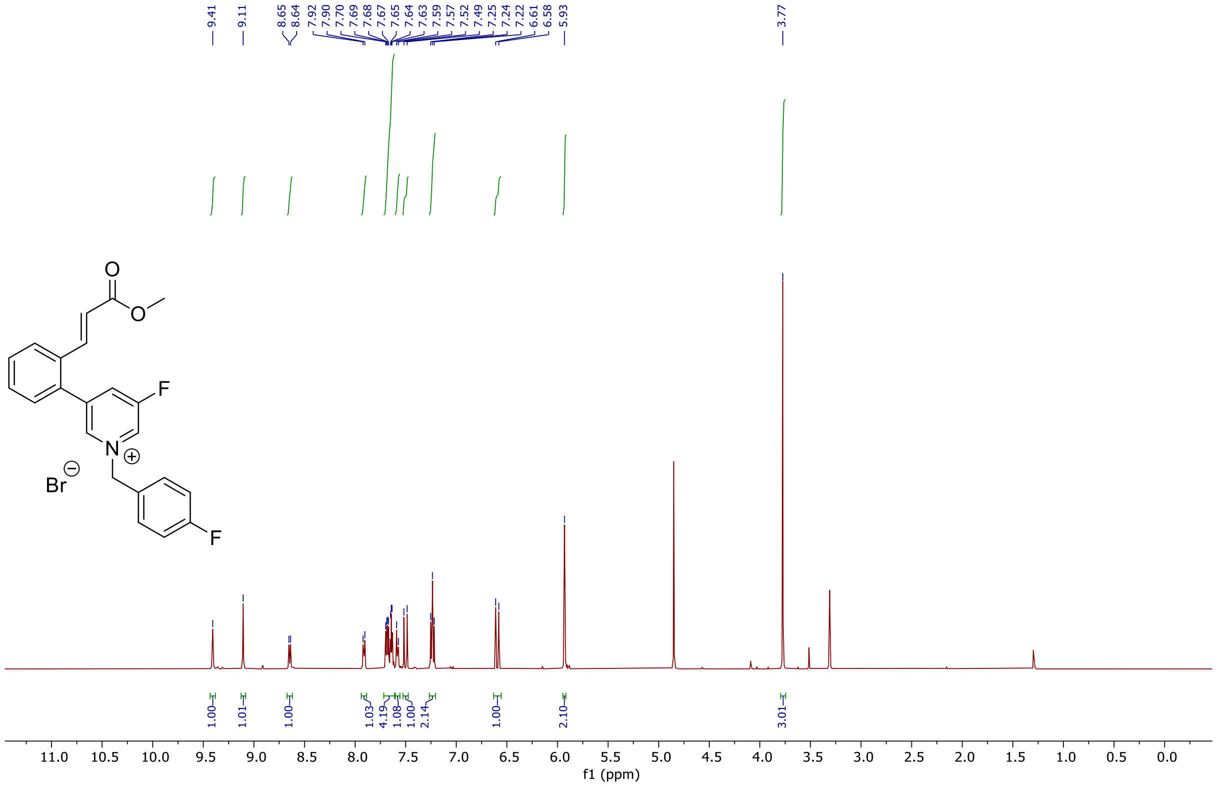

20210416-1659-B500_B.14-19.11.fid
Ref 532-3
Group Greaney_M
C13_CPD_Night256 MeOD /mnt/nmrdata/Greaney_M m31962tw 19

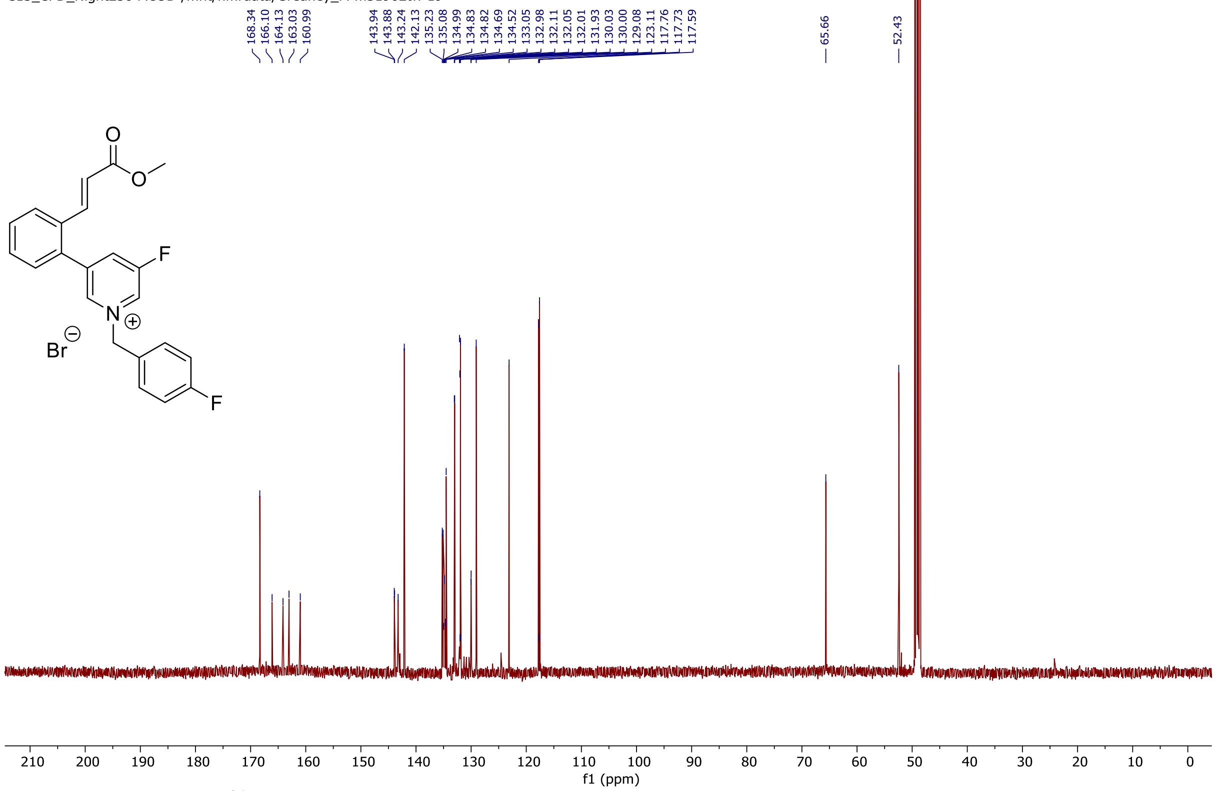

20210416-1659-B500_B.14-19.16.fid
Ref 532-3
Group Greaney_M
F19_NoCPD_Night MeOD /mnt/nmrdata/Greaney_M m31962tw 19

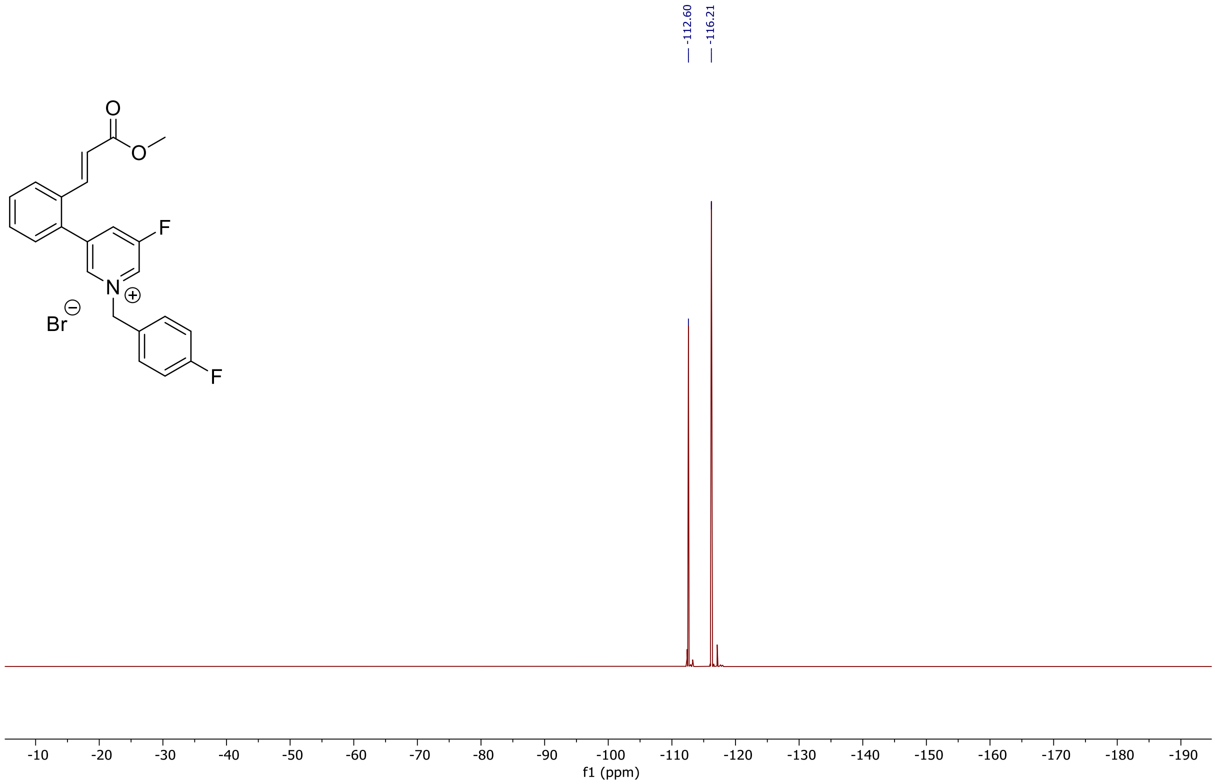

5u

20210409-1709-B400_B.11-2.10.fid
Ref 532-4
Group Greaney_M
H1_Night MeOD /mnt/nmrdata/Greaney_M m31962tw 2

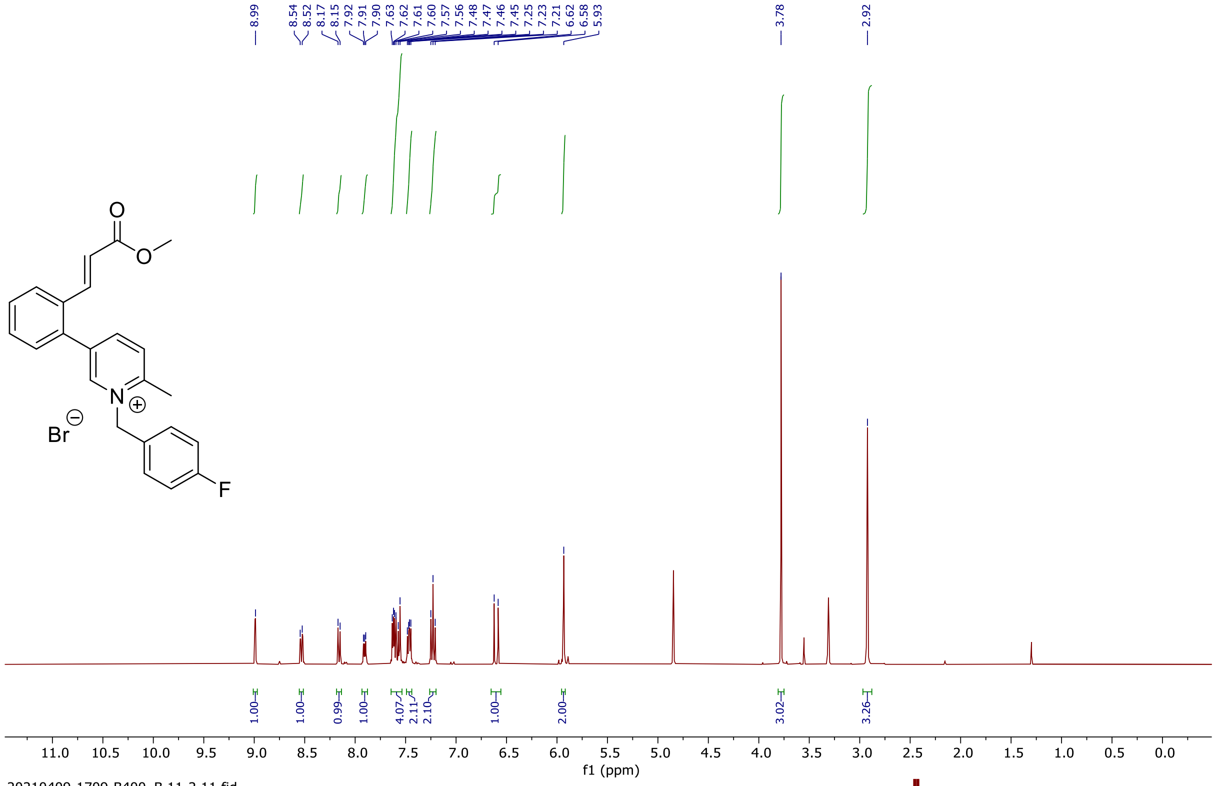

20210409-1709-B400_B.11-2.11.fid
Ref 532-4
Group Greaney_M
C13_CPD_Night256 MeOD /mnt/nmrdata/Greaney_M m31962tw 2

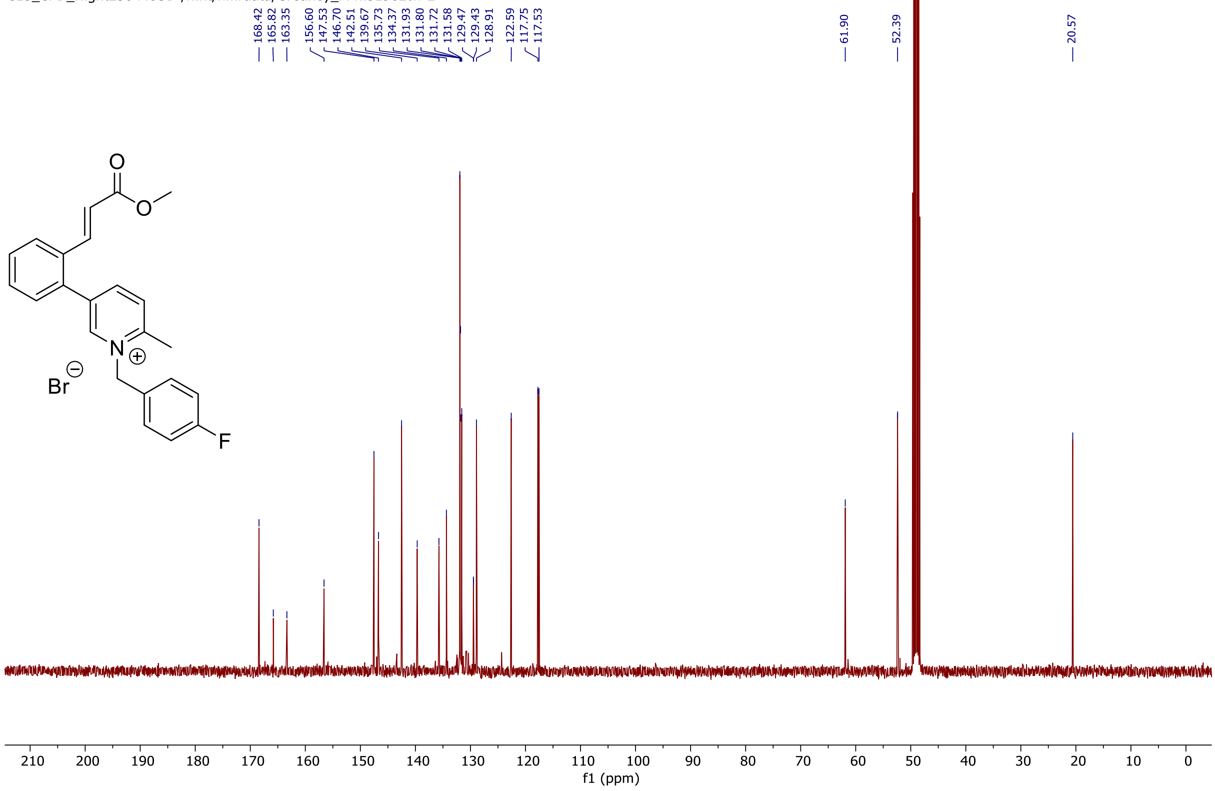

20210409-1709-B400_B.11-2.15.fid
Ref 532-4
Group Greaney_M
F19_NoCPD_Night MeOD /mnt/nmrdata/Greaney_M m31962tw 2

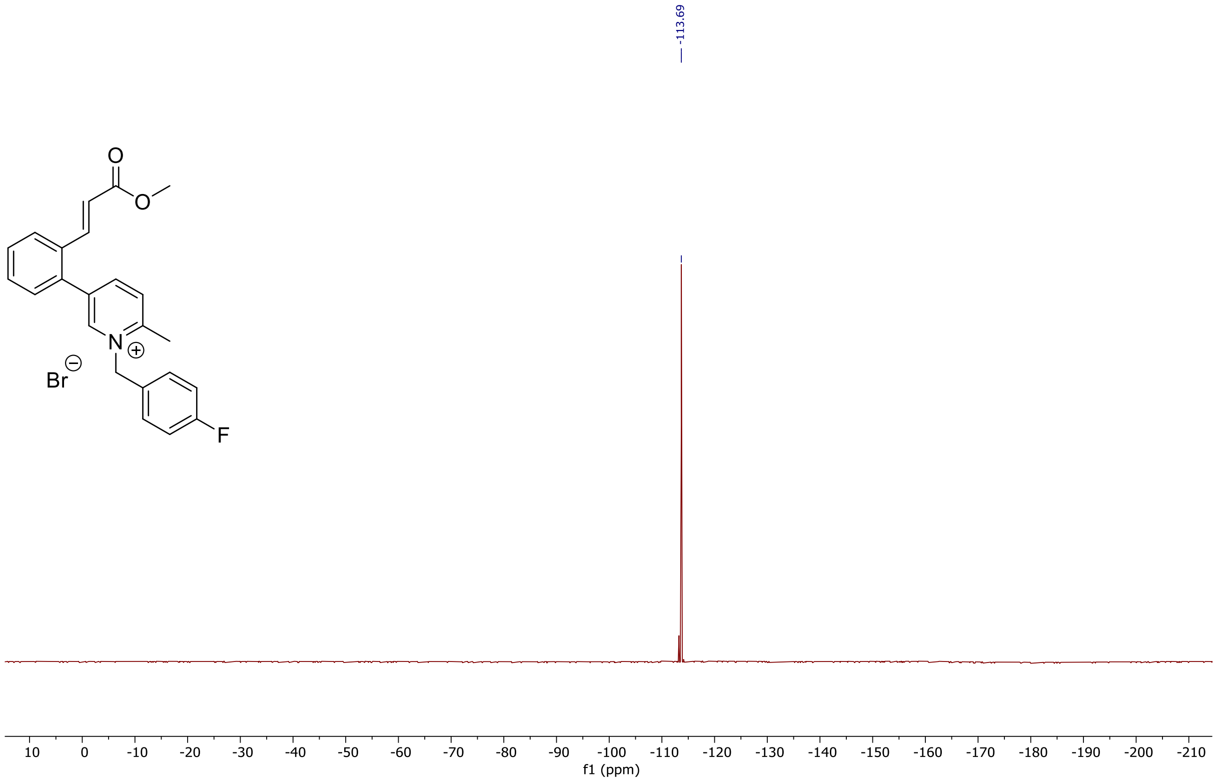

5v

20211125-1454-B400_B.11-36.10.fid
Ref 748-1
Group Greaney_M
H1_Night MeOD /mnt/nmrdata/Greaney_M m31962tw 36

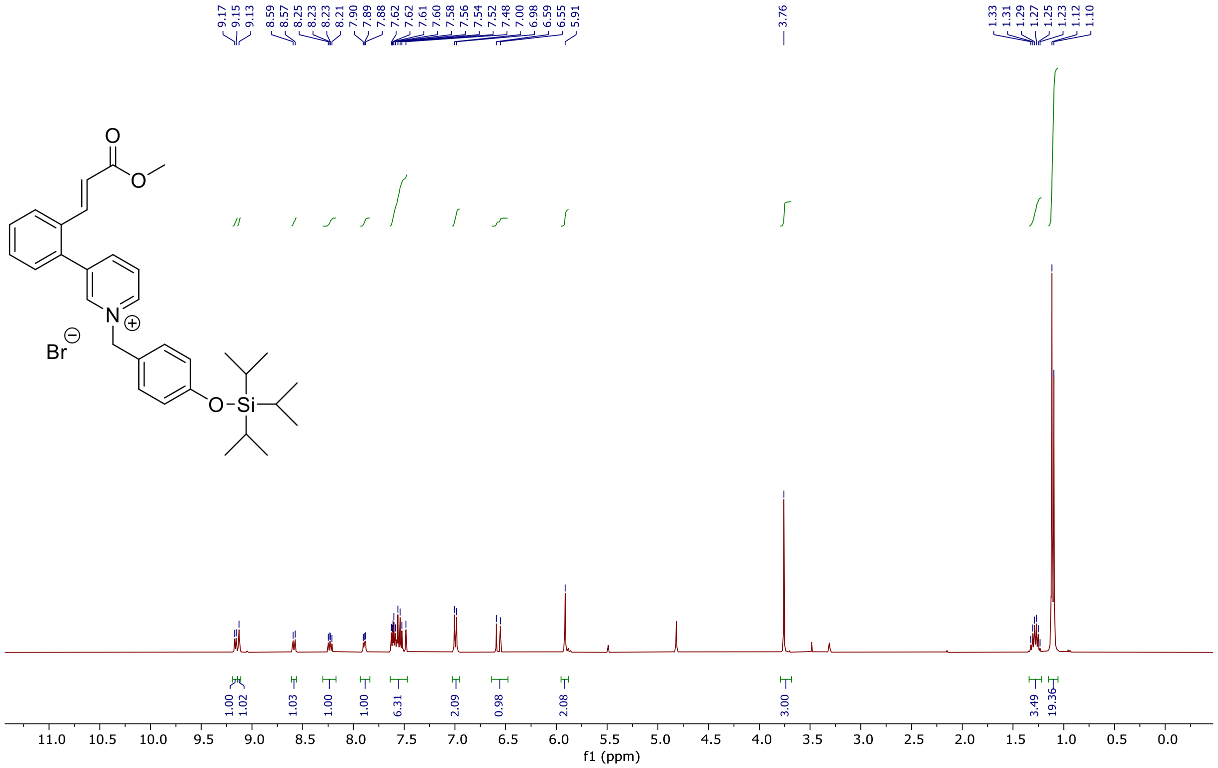

20211125-1454-B400_B.11-36.11.fid
Ref 748-1
Group Greaney_M
C13_CPD_Night256 MeOD /mnt/nmrdata/Greaney_M m31962tw 36

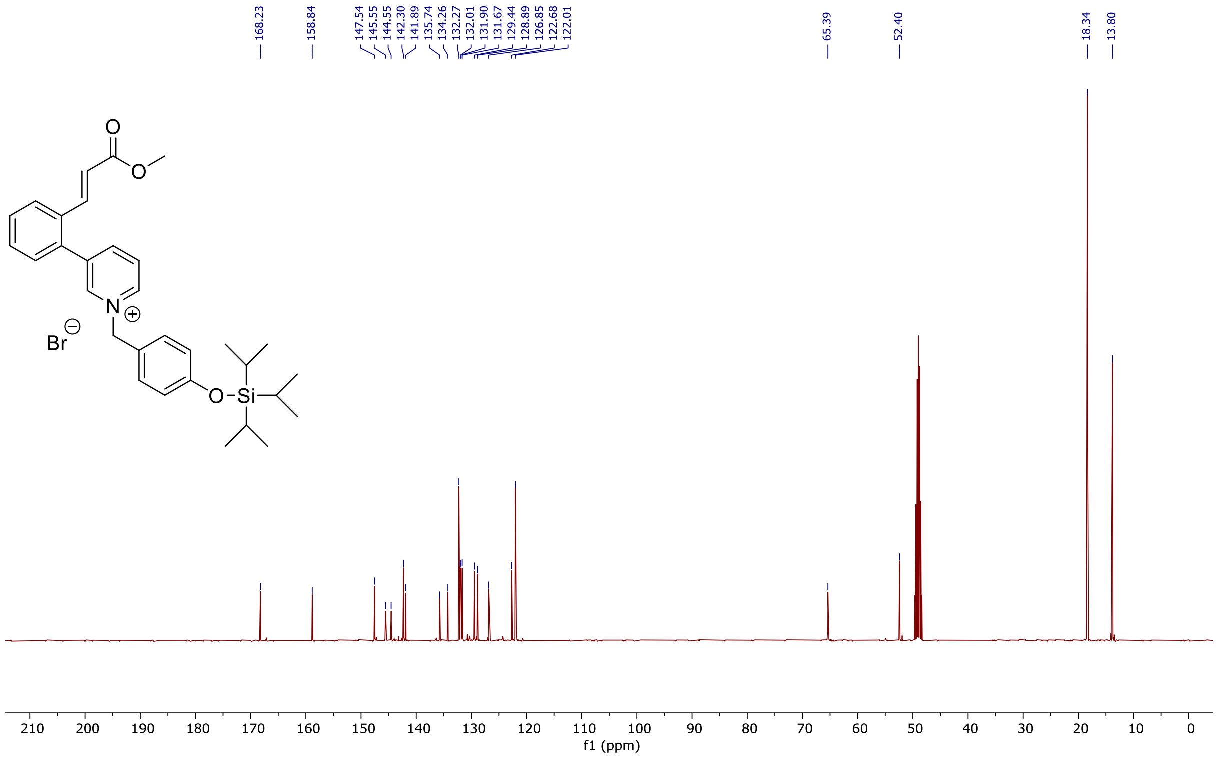

5w

20210805-1647-B400_B.11-54.10.fid
Ref 645-2
Group Greaney_M
H1_Night MeOD /mnt/nmrdata/Greaney_M m31962tw 54

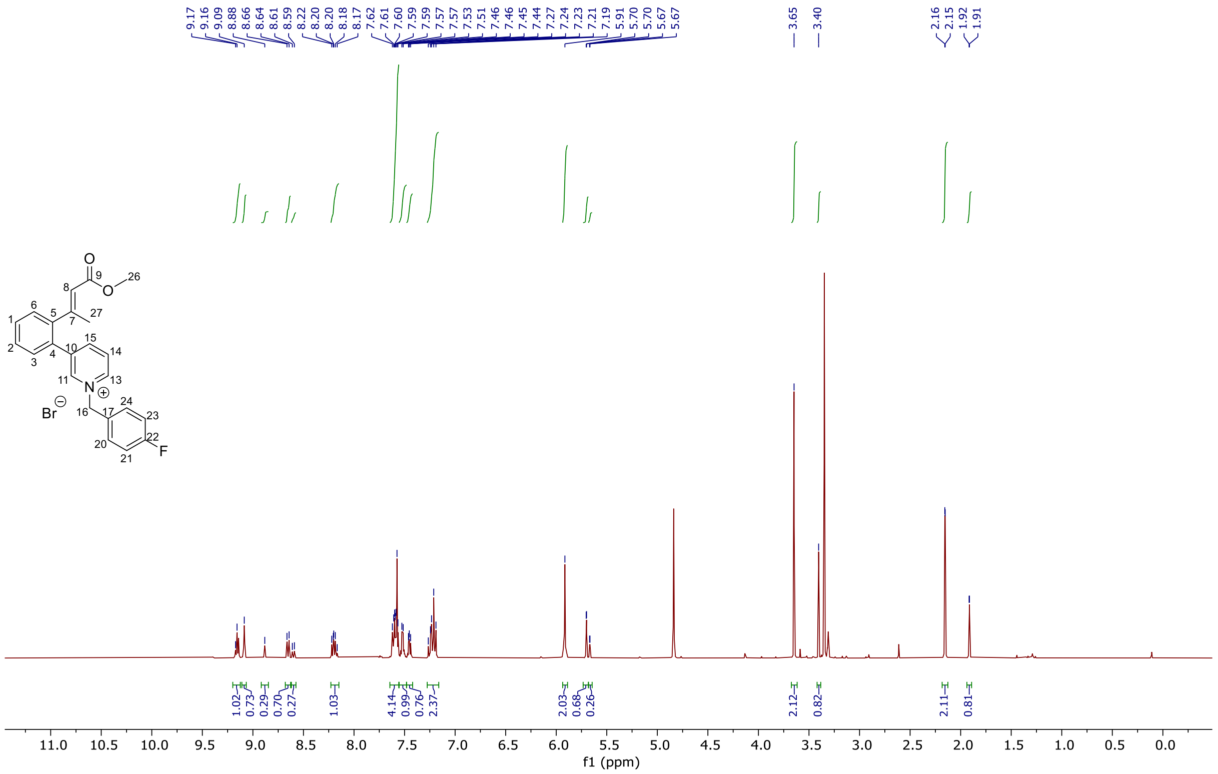

20210805-1647-B400_B.11-54.11.fid
Ref 645-2
Group Greaney_M
C13_CPD_Night256 MeOD /mnt/nmrdata/Greaney_M m31962tw 54

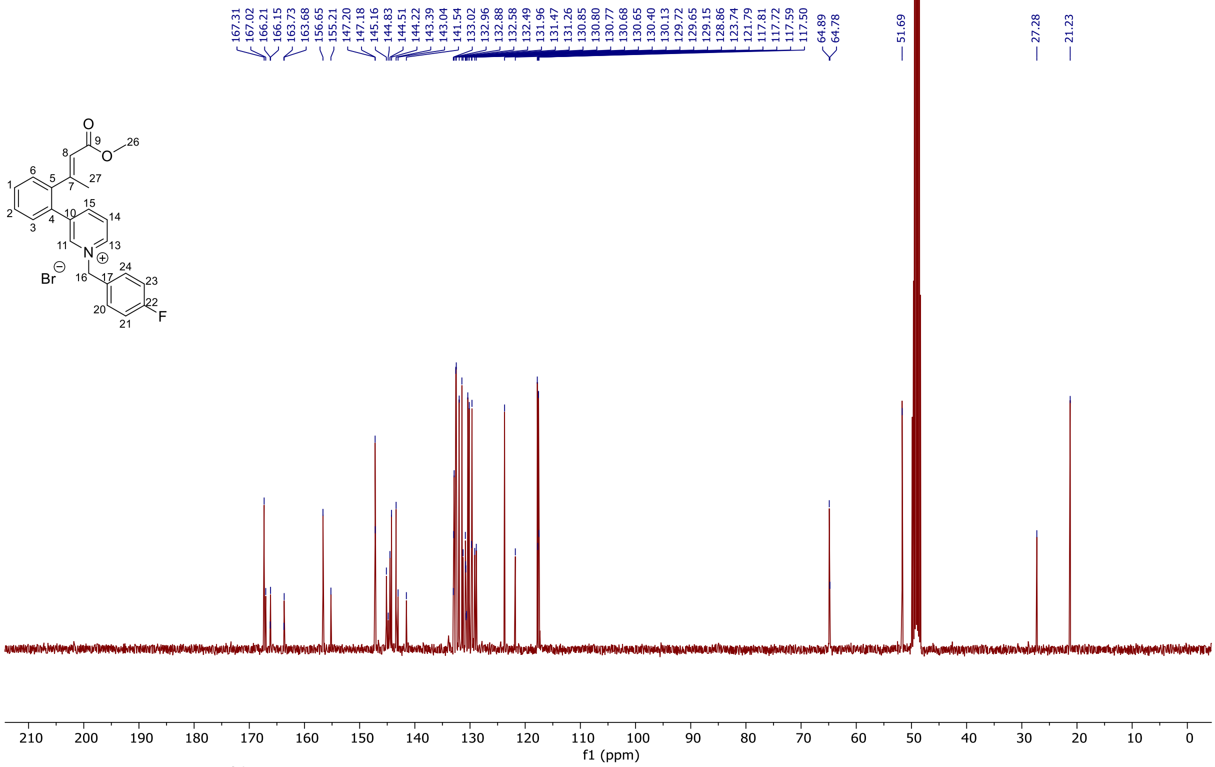

20210805-1647-B400_B.11-54.11.fid
Ref 645-2
Group Greaney_M
C13_CPD_Night256 MeOD /mnt/nmrdata/Greaney_M m31962tw 54

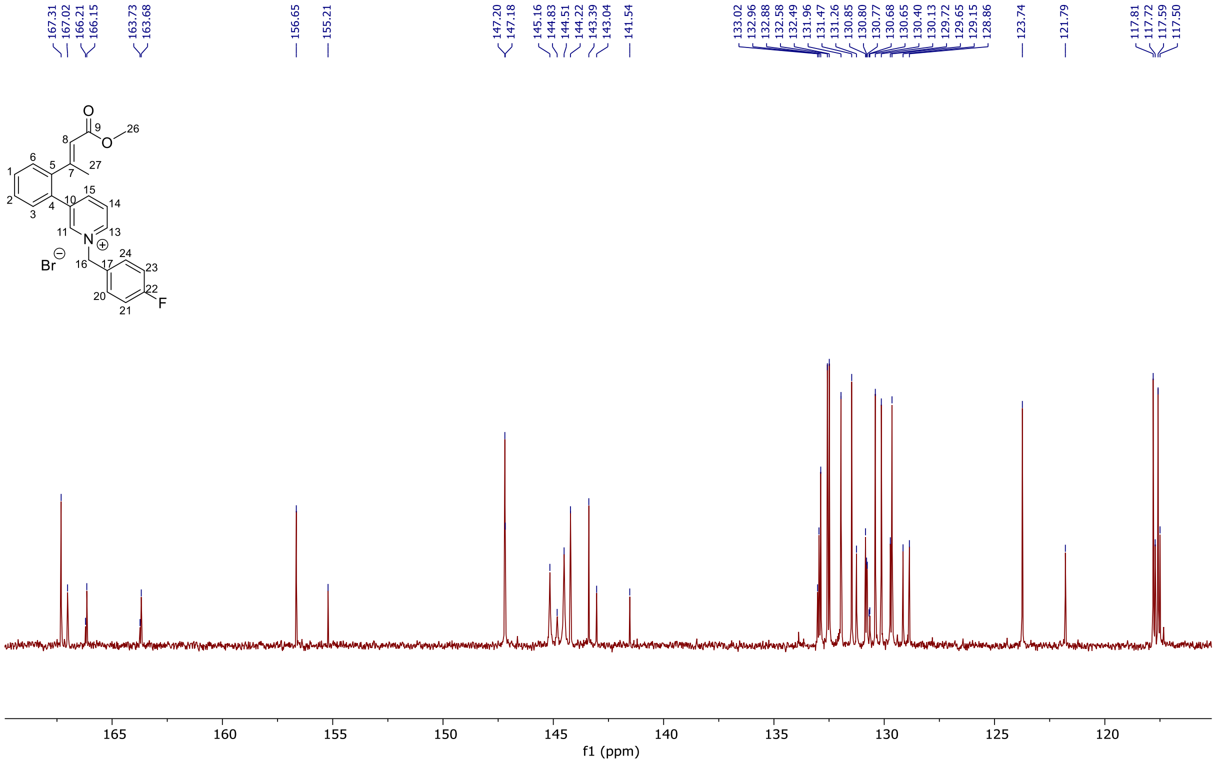

20210805-1647-B400_B.11-54.15.fid
Ref 645-2
Group Greaney_M
F19_NoCPD_Night MeOD /mnt/nmrdata/Greaney_M m31962tw 54

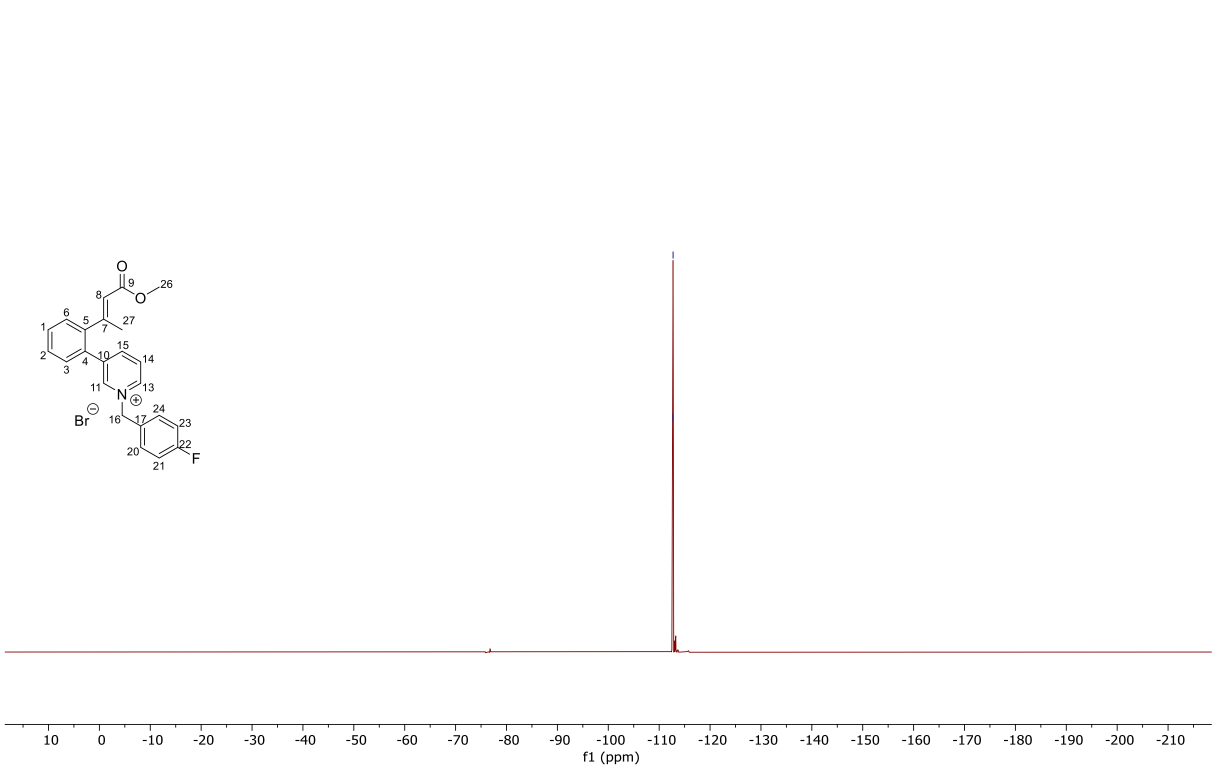

6a

20201125-1829-B400_B.12-37.10.fid
Ref 476-1
Group Greaney_M
H1_Night Acetone /mnt/nmrdata/Greaney_M m31962tw 37

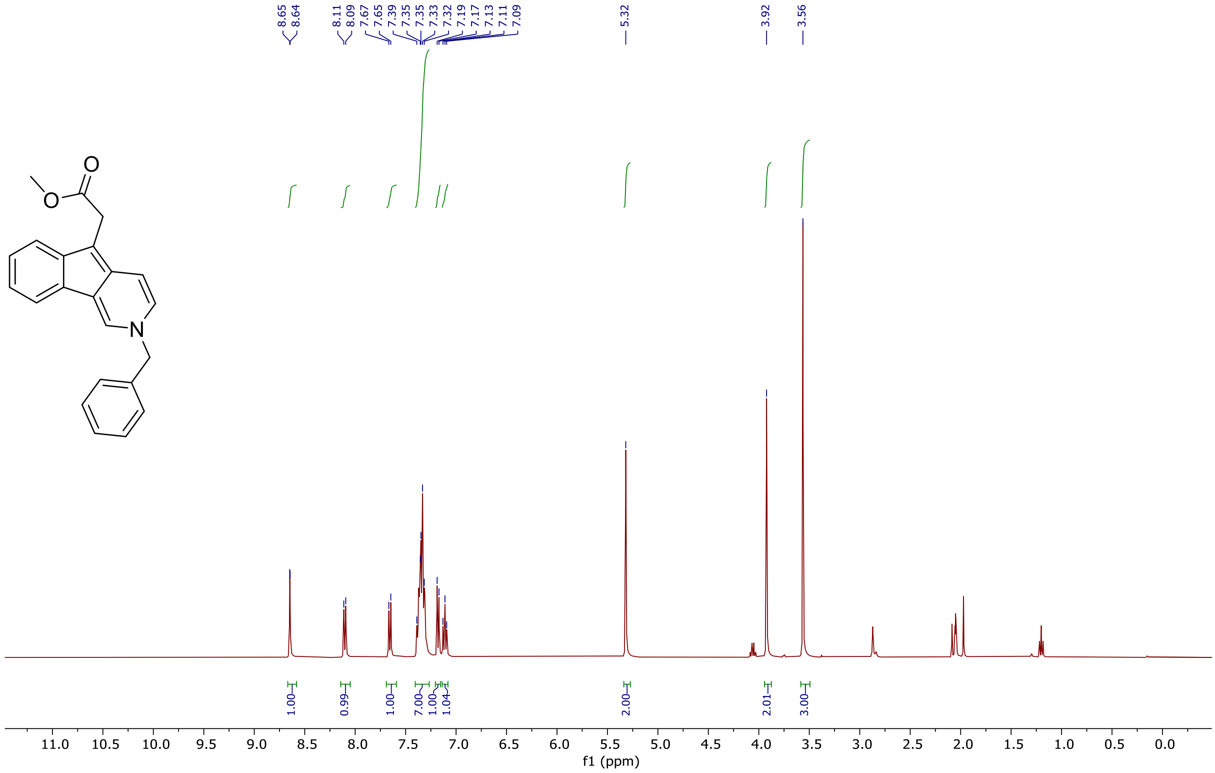

20201125-1829-B400_B.12-37.11.fid
Ref 476-1
Group Greaney_M
C13_CPD_Night256 Acetone /mnt/nmrdata/Greaney_M m31962tw 37

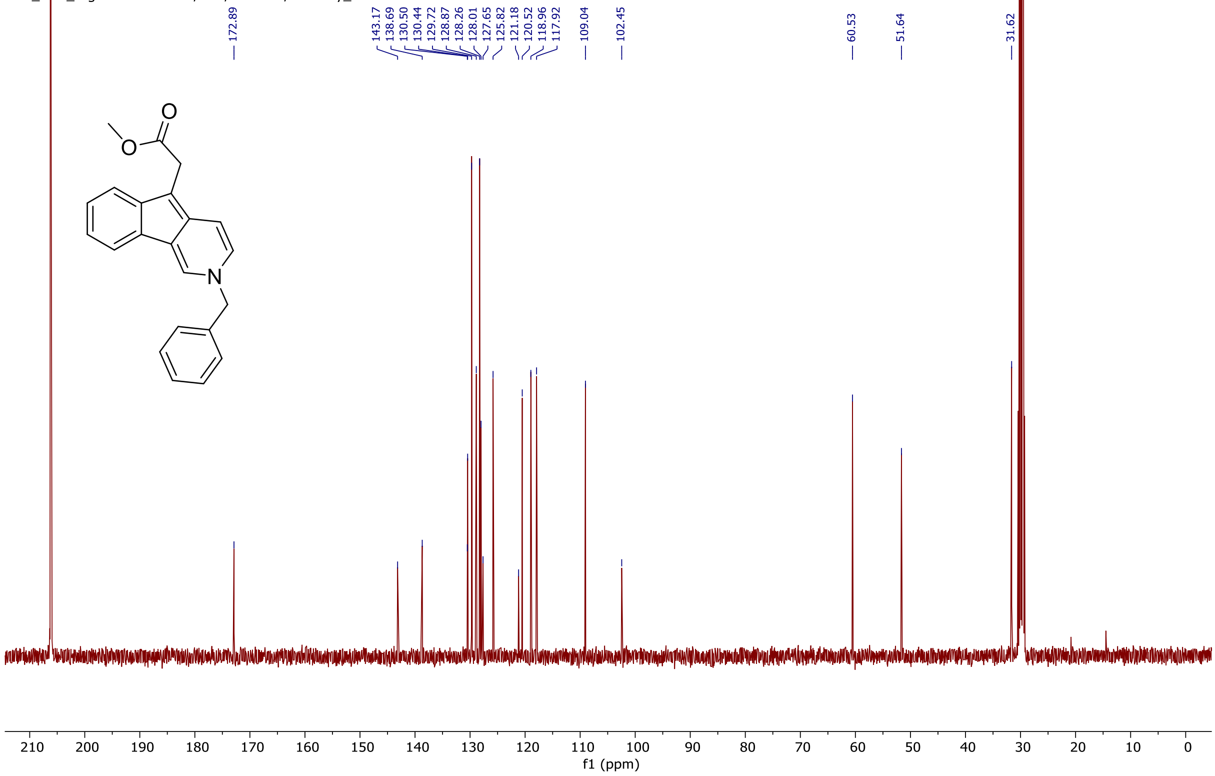

6b

20210507-1737-B400_B.11-32.10.fid
Ref 568-1
Group Greaney_M
H1_Night Acetone /mnt/nmrdata/Greaney_M m31962tw 32

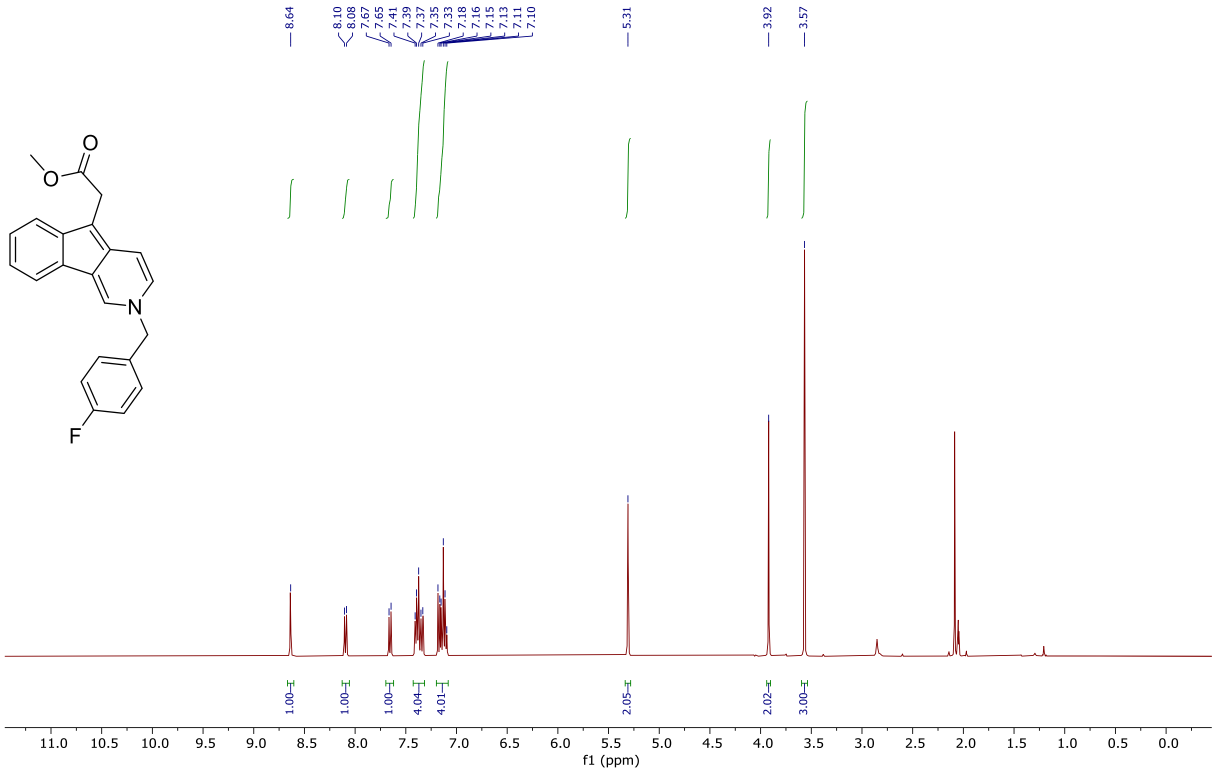

20210507-1737-B400_B.11-32.11.fid
Ref 568-1
Group Greaney_M
C13_CPD_Night256 Acetone /mnt/nmrdata/Greaney_M m31962tw 32

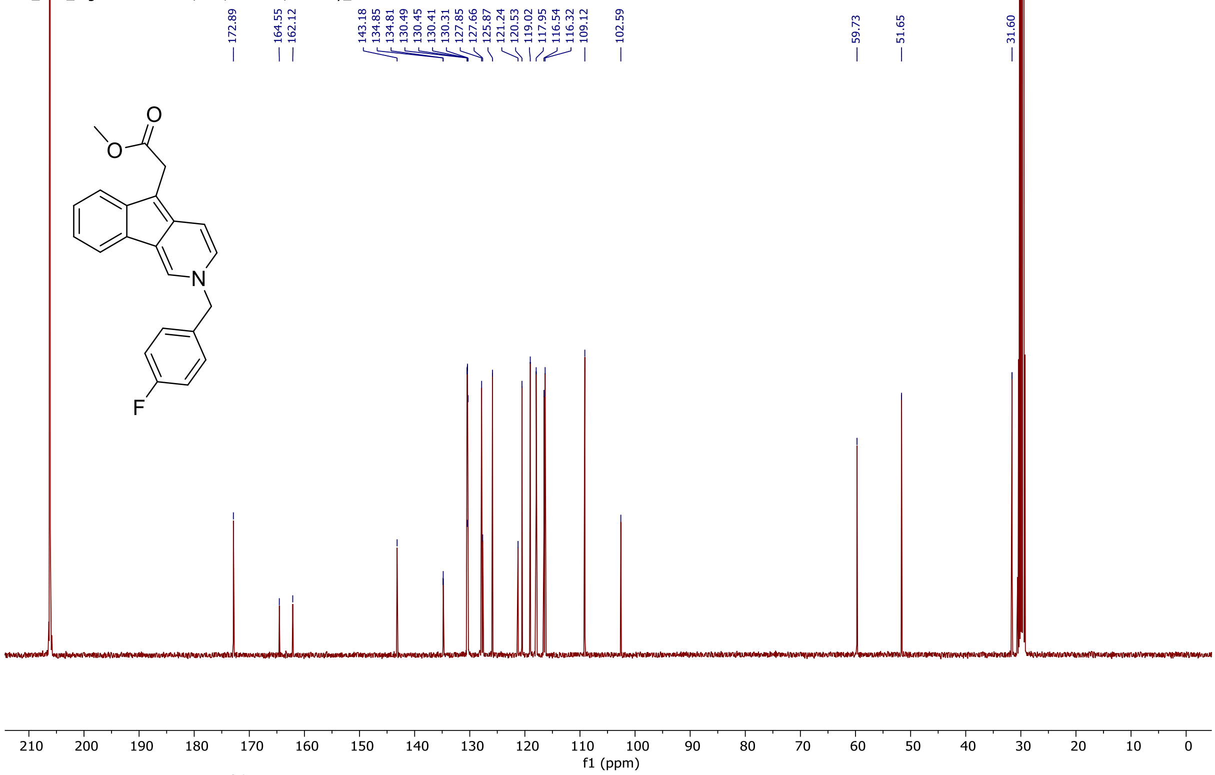

20210507-1737-B400_B.11-32.12.fid
Ref 568-1
Group Greaney_M
F19_NoCPD_Night Acetone /mnt/nmrdata/Greaney_M m31962tw 32

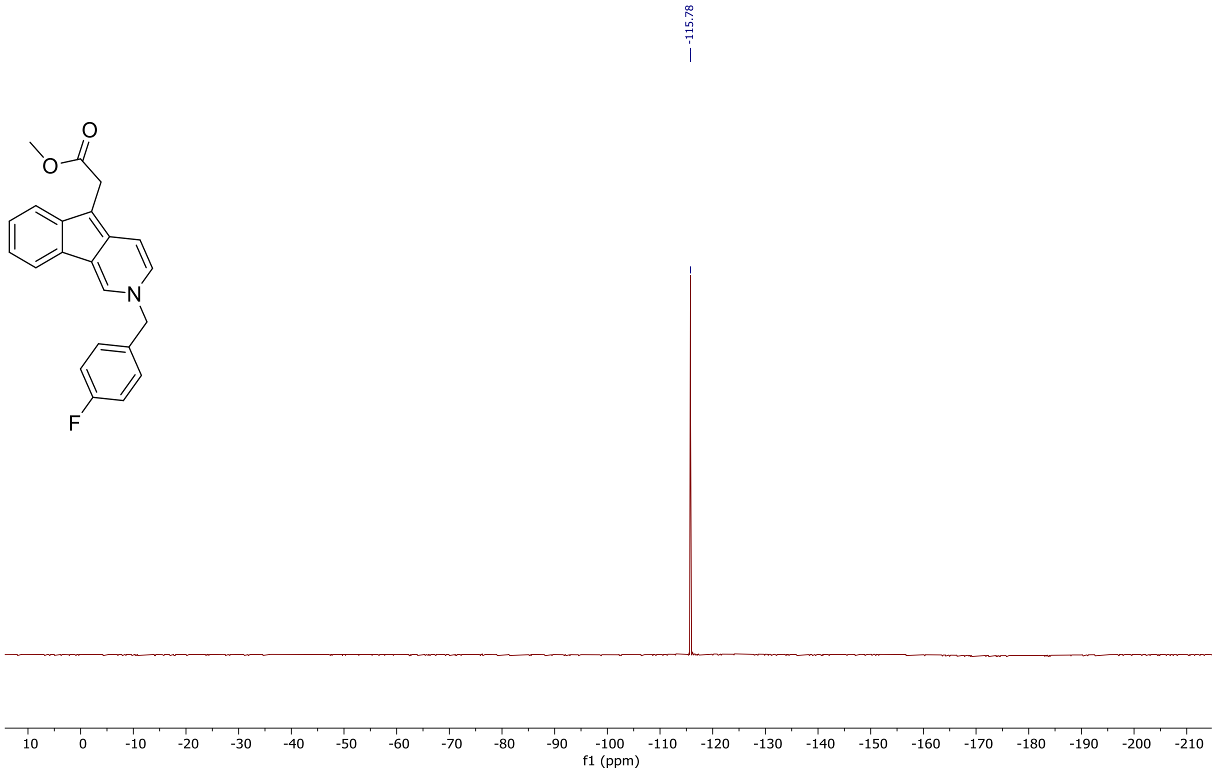

6c

20210303-1814-B400_B.12-36.10.fid
Ref 542-3
Group Greaney_M
H1_Night Acetone /mnt/nmrdata/Greaney_M m31962tw 36

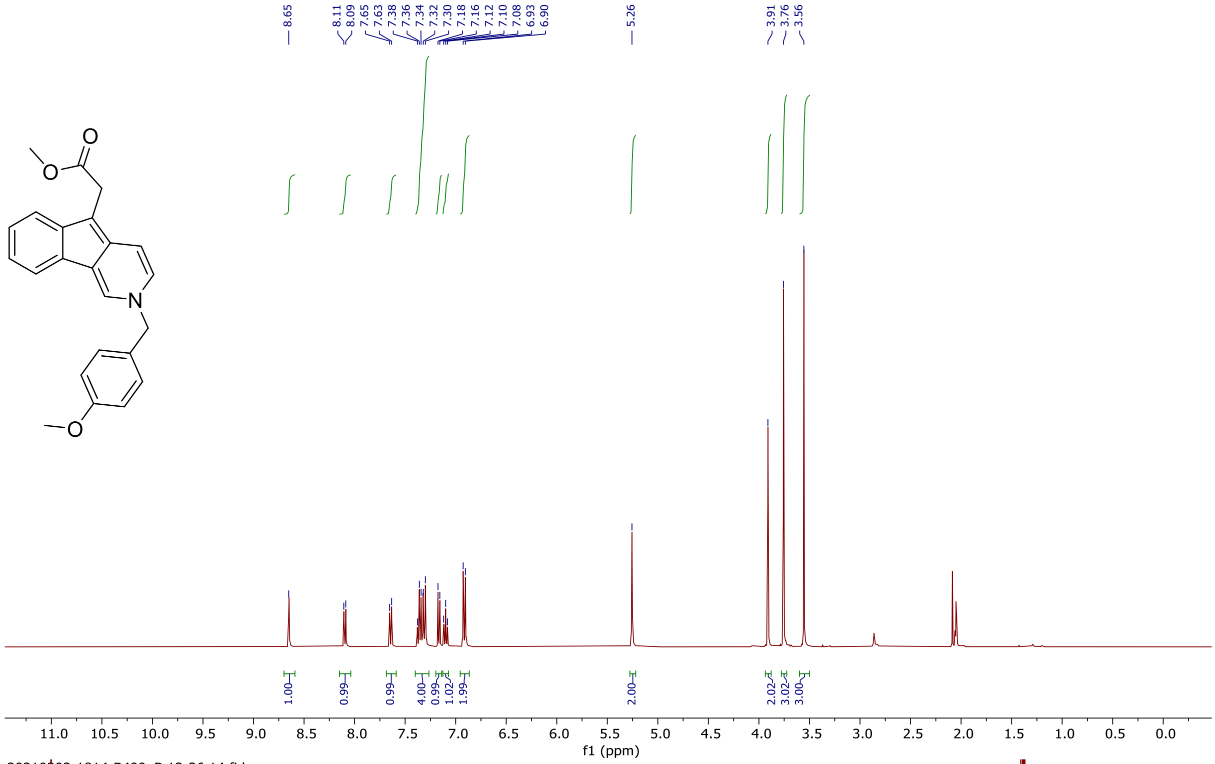

20210303-1814-B400_B.12-36.14.fid
Ref 542-3
Group Greaney_M
C13_CPD_Night256 Acetone /mnt/nmrdata/Greaney_M m31962tw 36

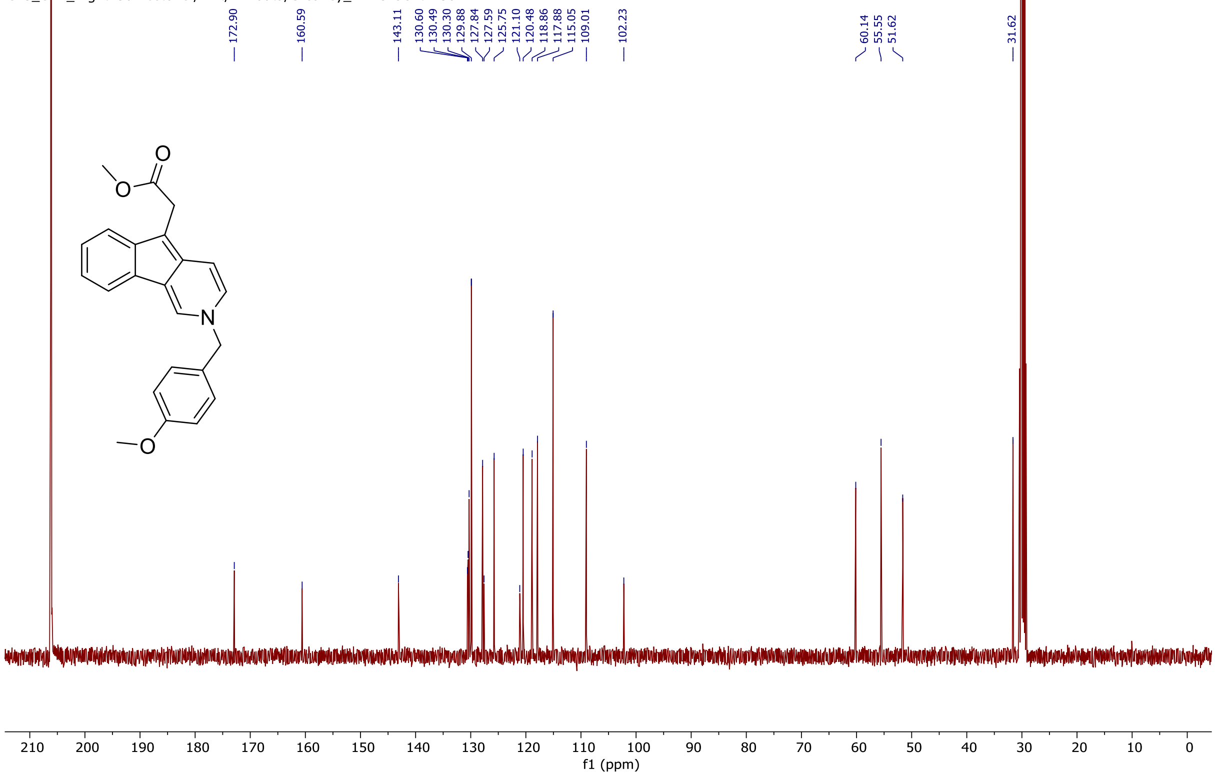

6d

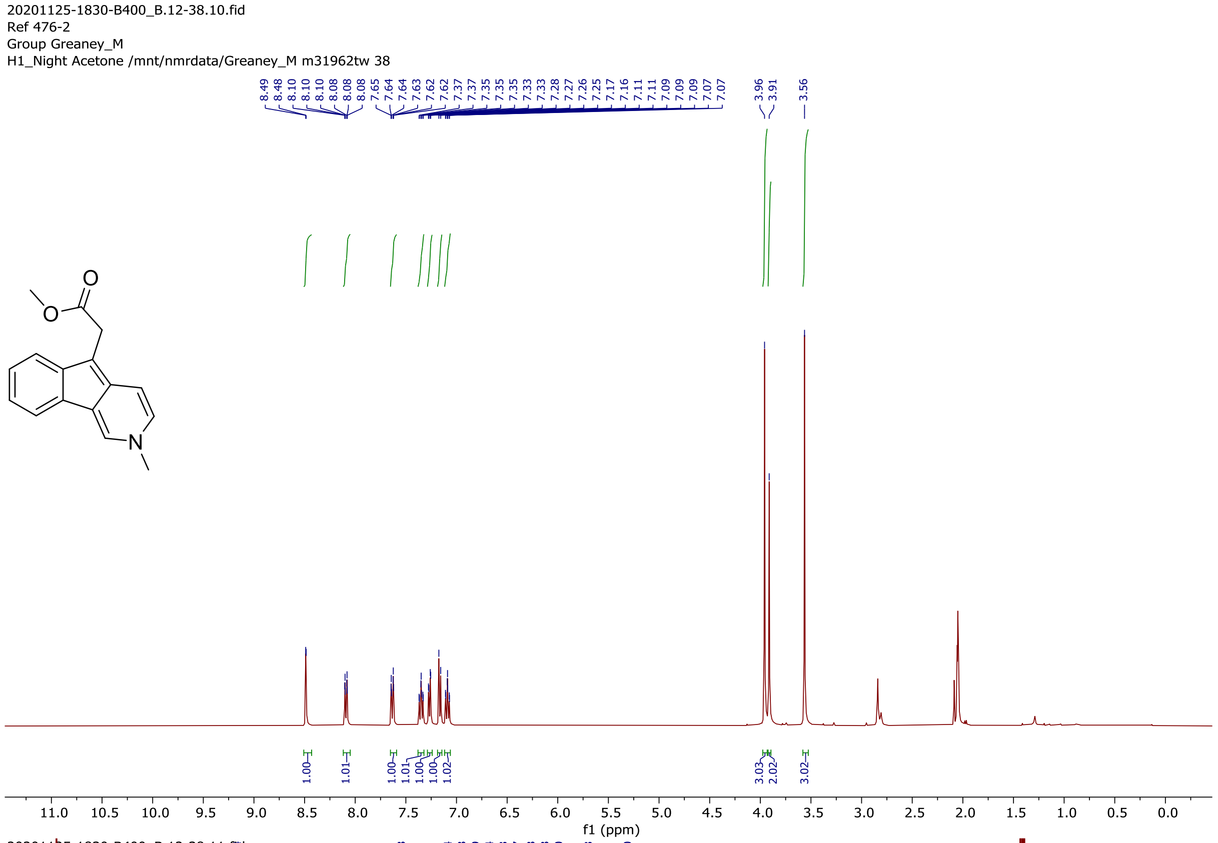
20201125-1830-B400_B.12-38.10.fid
Ref 476-2
Group Greaney_M
H1_Night Acetone /mnt/nmrdata/Greaney_M m31962tw 38
8.49
8.48
8.10
8.10
8.08
8.08
8.08
7.65
7.64
7.64
7.63
7.62
7.62
7.37
7.37
7.35
7.35
7.35
7.33
7.33
7.28
7.27
7.26
7.25
7.17
7.16
7.11
7.11
7.09
7.09
7.09
7.07
7.07
3.96
3.91
3.56
O
O
N
1.00
1.01
1.00
1.01
1.00
1.00
1.02
3.03
2.02
3.02
11.0
10.5
10.0
9.5
9.0
8.5
8.0
7.5
7.0
6.5
6.0
5.5
5.0
4.5
4.0
3.5
3.0
2.5
2.0
1.5
1.0
0.5
0.0
f1 (ppm)

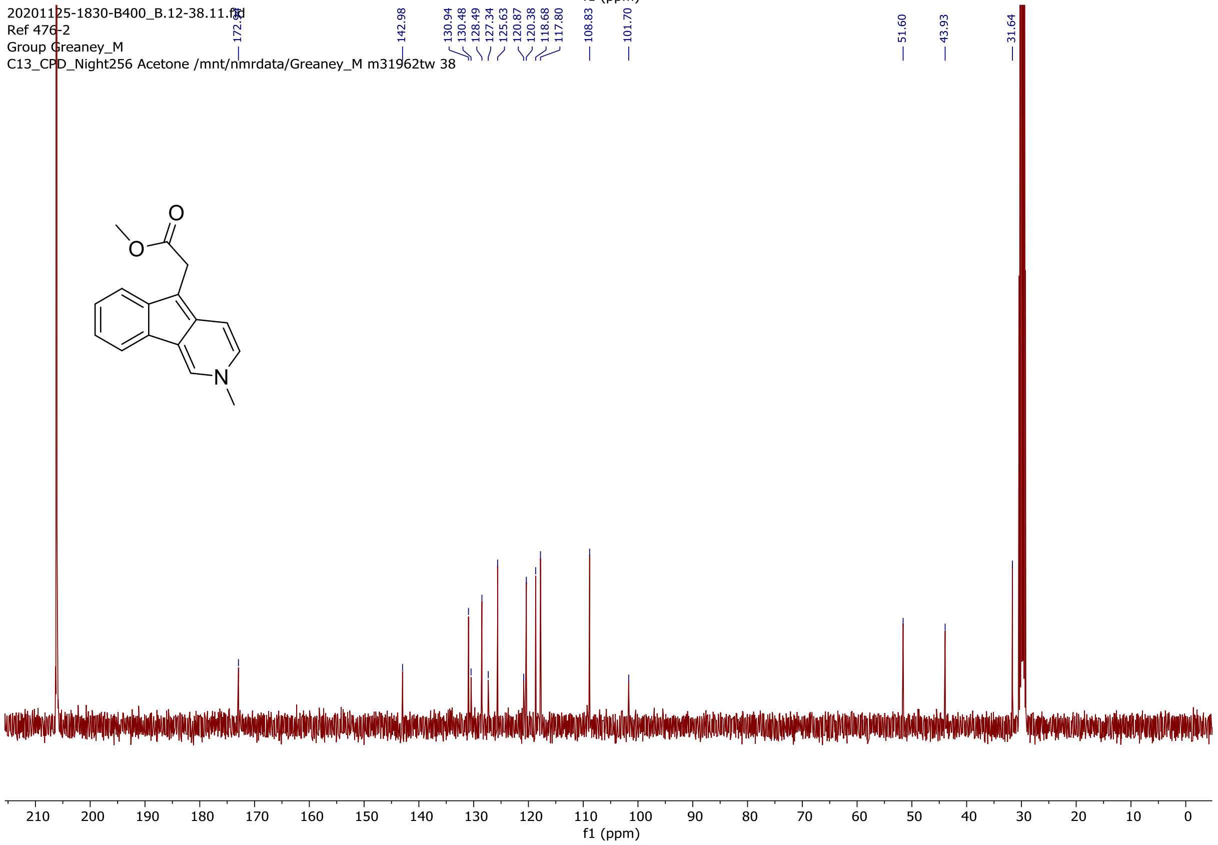
20201125-1830-B400_B.12-38.11.fid
Ref 476-2
Group Greaney_M
C13_CPD_Night256 Acetone /mnt/nmrdata/Greaney_M m31962tw 38
172.9
142.98
130.94
130.48
128.49
127.34
125.63
120.87
120.38
118.68
117.80
108.83
101.70
51.60
43.93
31.64
O
O
N
210
200
190
180
170
160
150
140
130
120
110
100
90
80
70
60
50
40
30
20
10
0
f1 (ppm)

6e

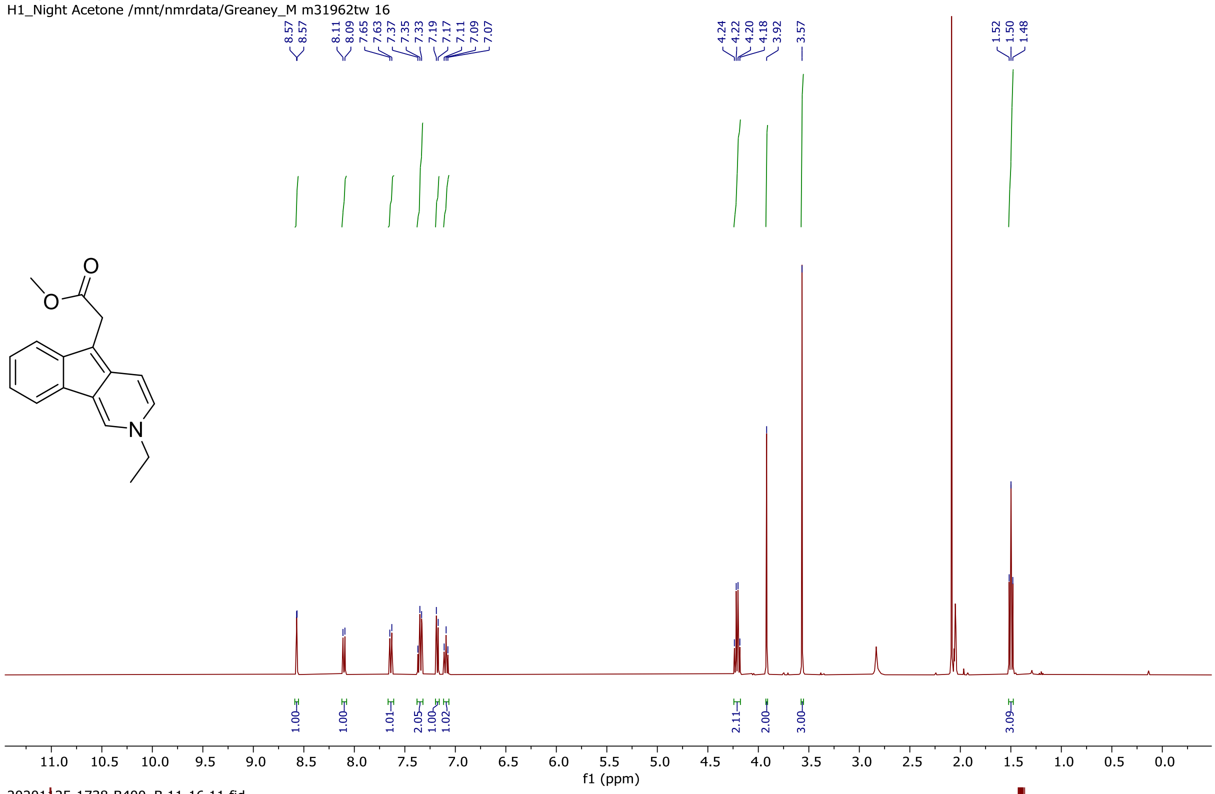

20201125-1738-B400_B.11-16.10.fid
Ref 474-5
Group Greaney_M
H1_Night Acetone /mnt/nmrdata/Greaney_M m31962tw 16
8.57
8.57
8.11
8.09
7.65
7.63
7.37
7.35
7.33
7.19
7.17
7.11
7.09
7.07
4.24
4.22
4.20
4.18
3.92
3.57
1.52
1.50
1.48
O
O
N
1.00
1.00
1.01
2.05
1.00
1.02
2.11
2.00
3.00
3.09
f1 (ppm)

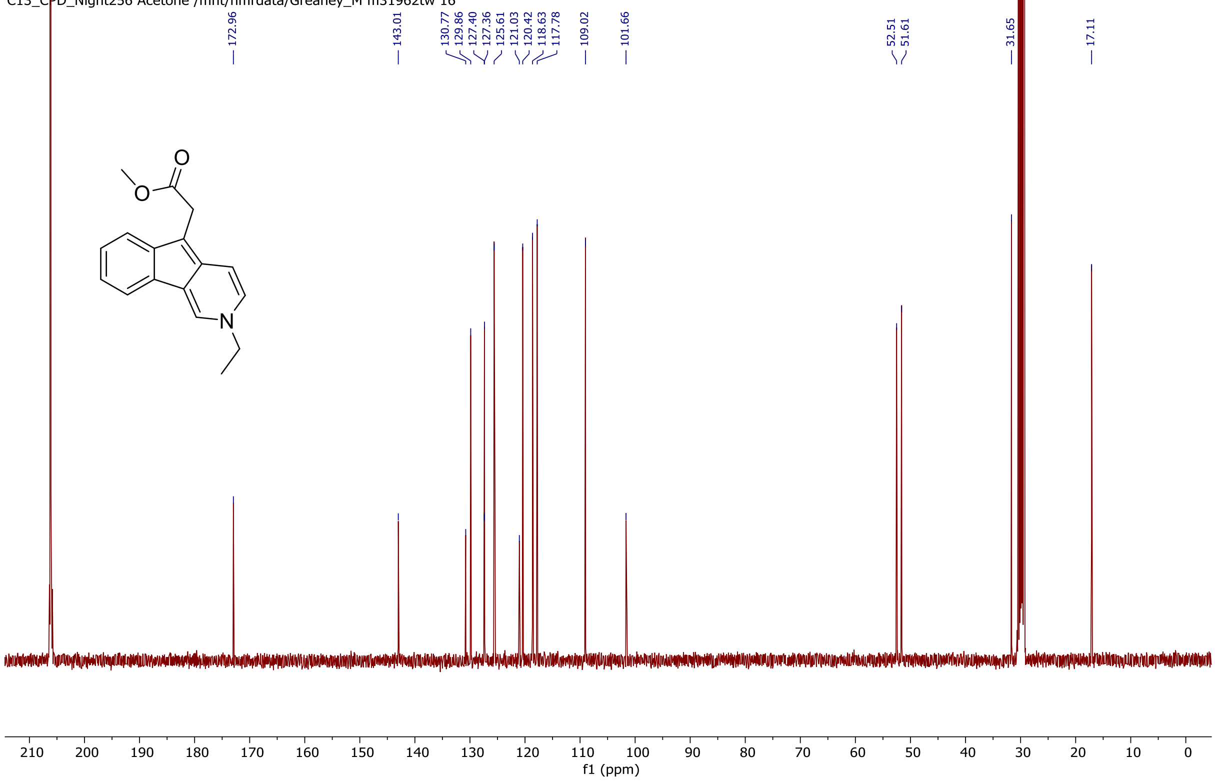

20201125-1738-B400_B.11-16.11.fid
Ref 474-5
Group Greaney_M
C13_CPD_Night256 Acetone /mnt/nmrdata/Greaney_M m31962tw 16
172.96
143.01
130.77
129.86
127.40
127.36
125.61
121.03
120.42
118.63
117.78
109.02
101.66
52.51
51.61
31.65
17.11
O
O
N
f1 (ppm)

6f

20201124-1858-B400_B.11-54.10.fid
Ref 474-4
Group Greaney_M
H1_Day Acetone /mnt/nmrdata/Greaney_M m31962tw 54

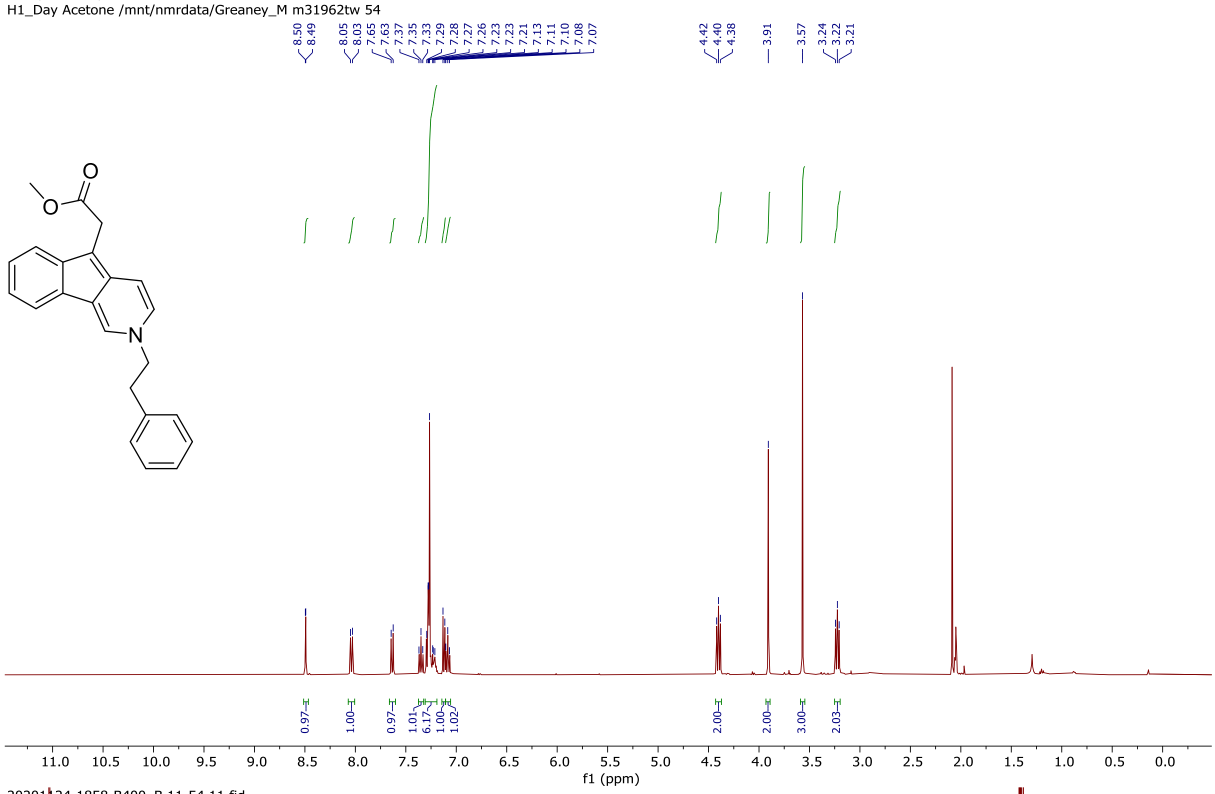

20201124-1858-B400_B.11-54.11.fid
Ref 474-4
Group Greaney_M
C13_CPD_Night256 Acetone /mnt/nmrdata/Greaney_M m31962tw 54

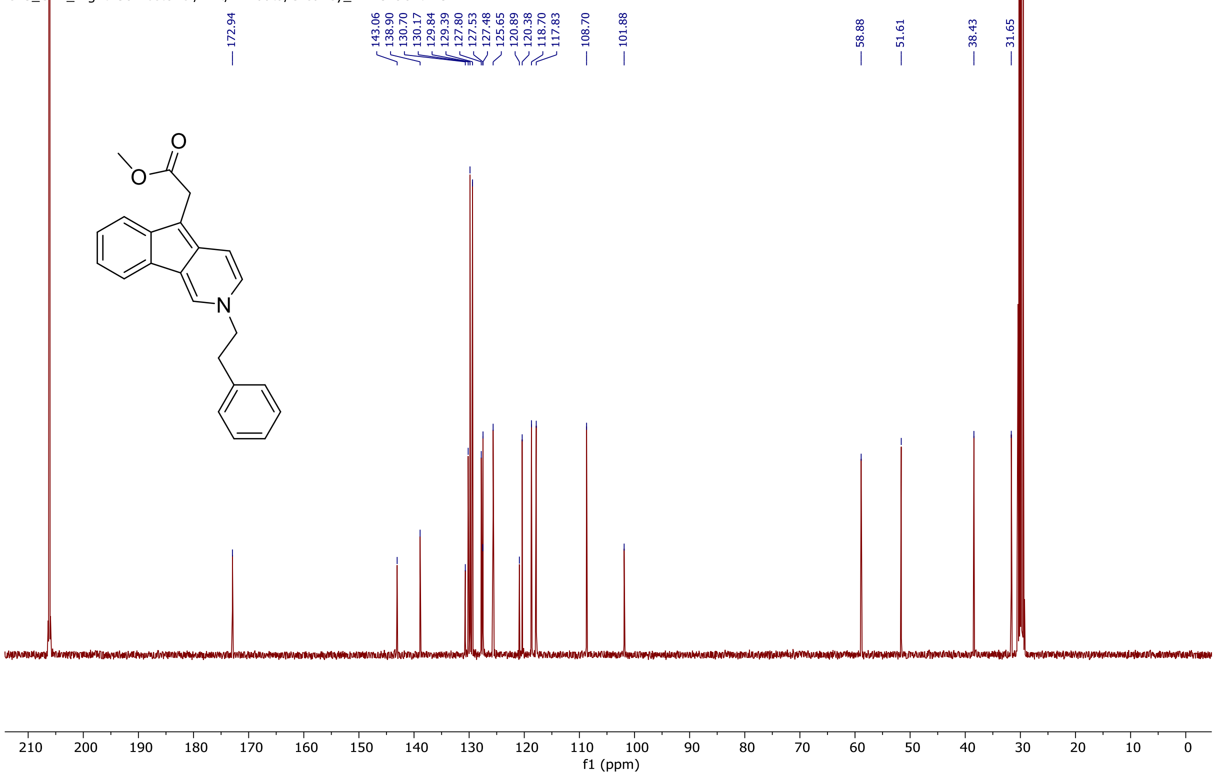

6g

20201208-1853-B400_B.11-48.10.fid
Ref 482-4
Group Greaney_M
H1_Night Acetone /mnt/nmrdata/Greaney_M m31962tw 48

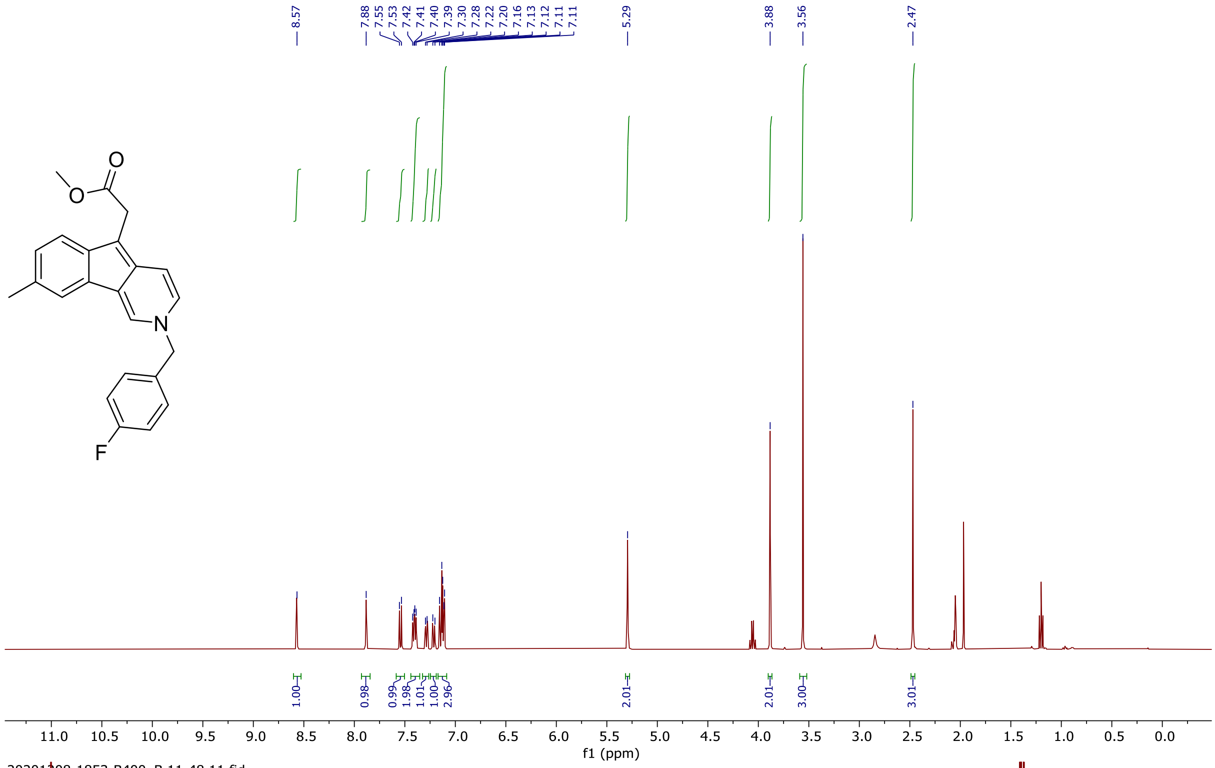

20201208-1853-B400_B.11-48.11.fid
Ref 482-4
Group Greaney_M
C13_CPD_Night256 Acetone /mnt/nmrdata/Greaney_M m31962tw 48

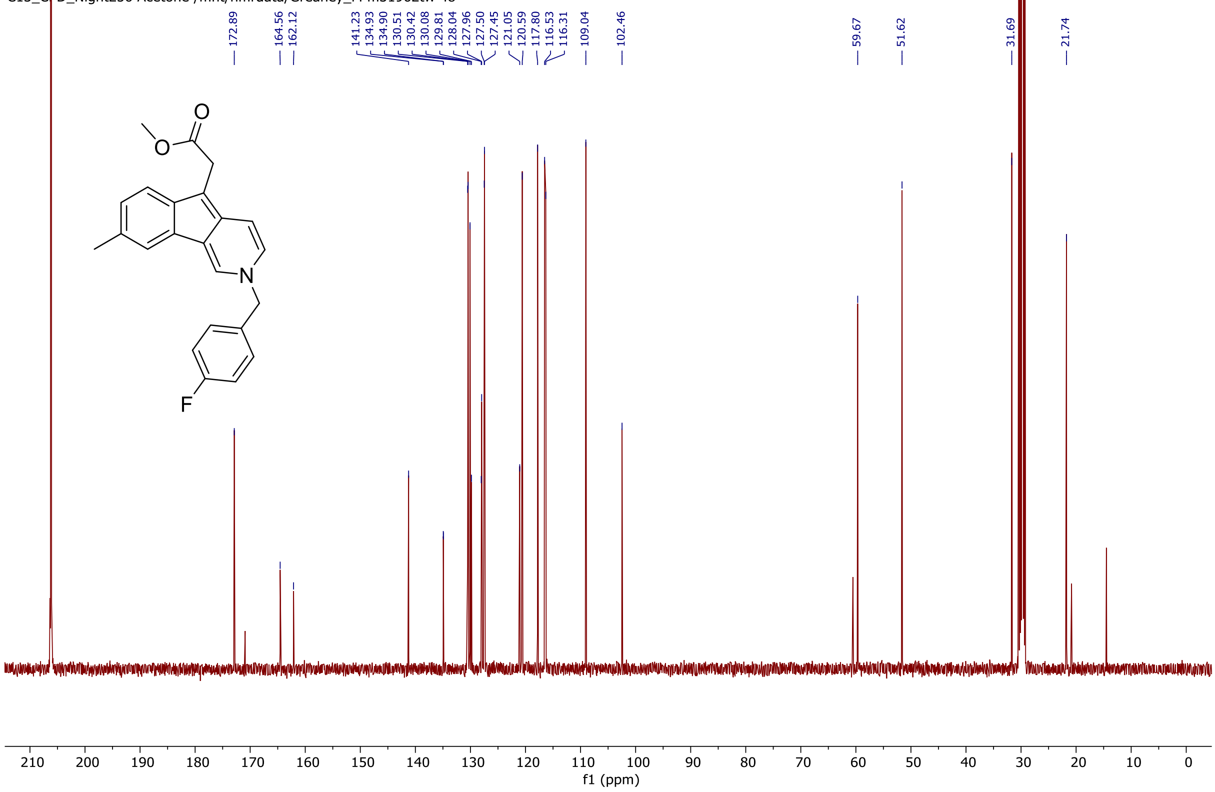

20201211-1136-B400_B.12-5.12.fid
Ref 482-4
Group Greaney_M
F19_CPD_Night Acetone /mnt/nmrdata/Greaney_M m31962tw 5

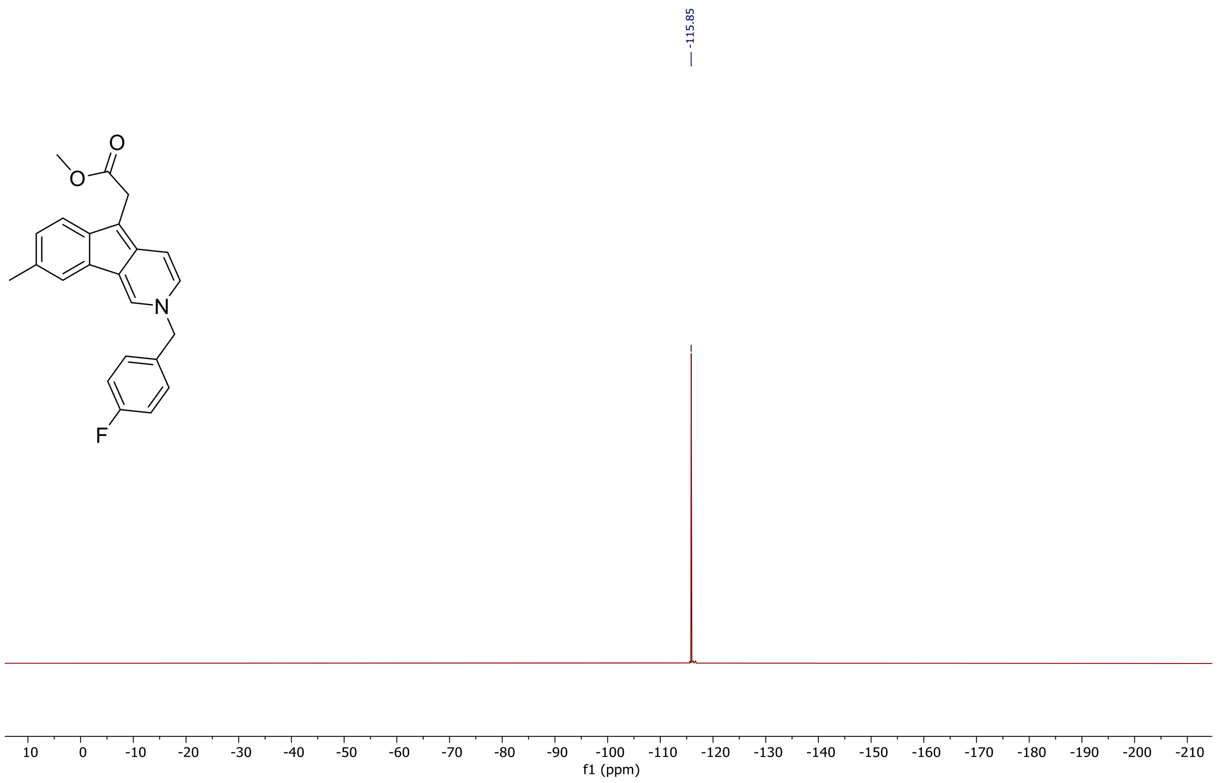

6h

20201208-1852-B400_B.11-46.10.fid
Ref 482-2
Group Greaney_M
H1_Night Acetone /mnt/nmrdata/Greaney_M m31962tw 46

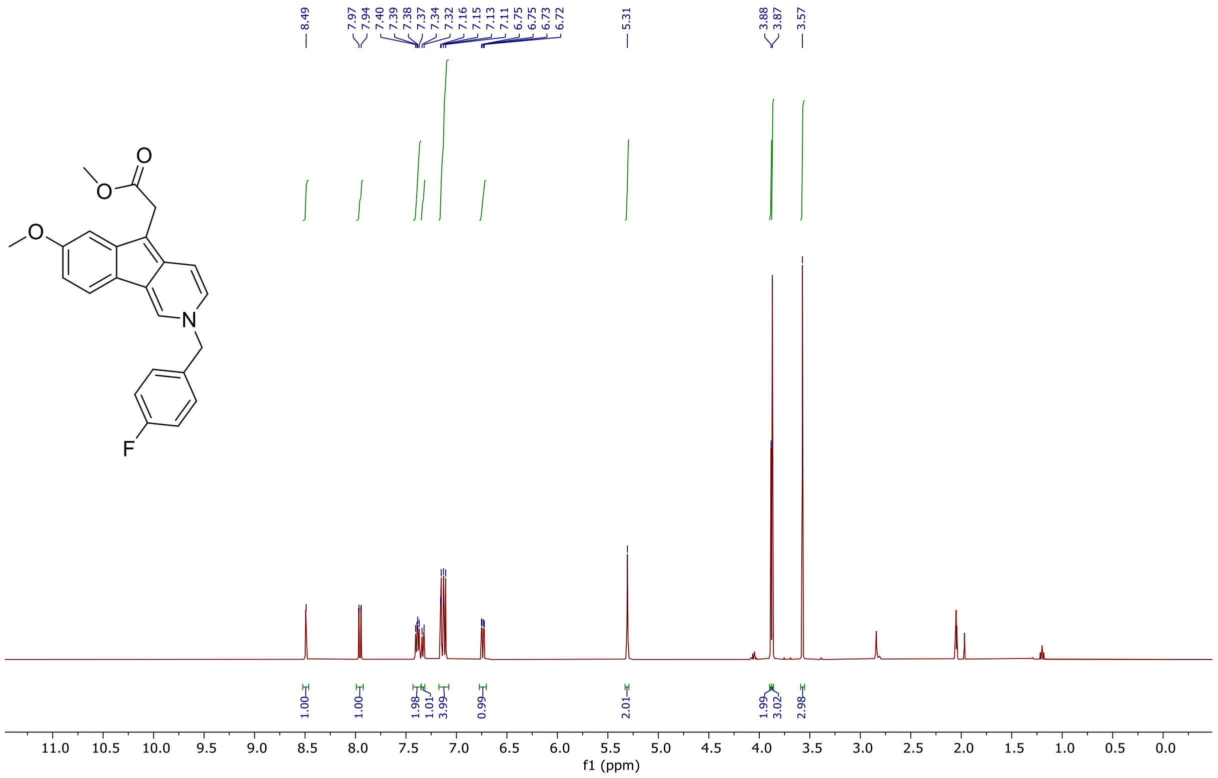

20201208-1852-B400_B.11-46.11.fid
Ref 482-2
Group Greaney_M
C13_CPD_Night256 Acetone /mnt/nmrdata/Greaney_M m31962tw 46

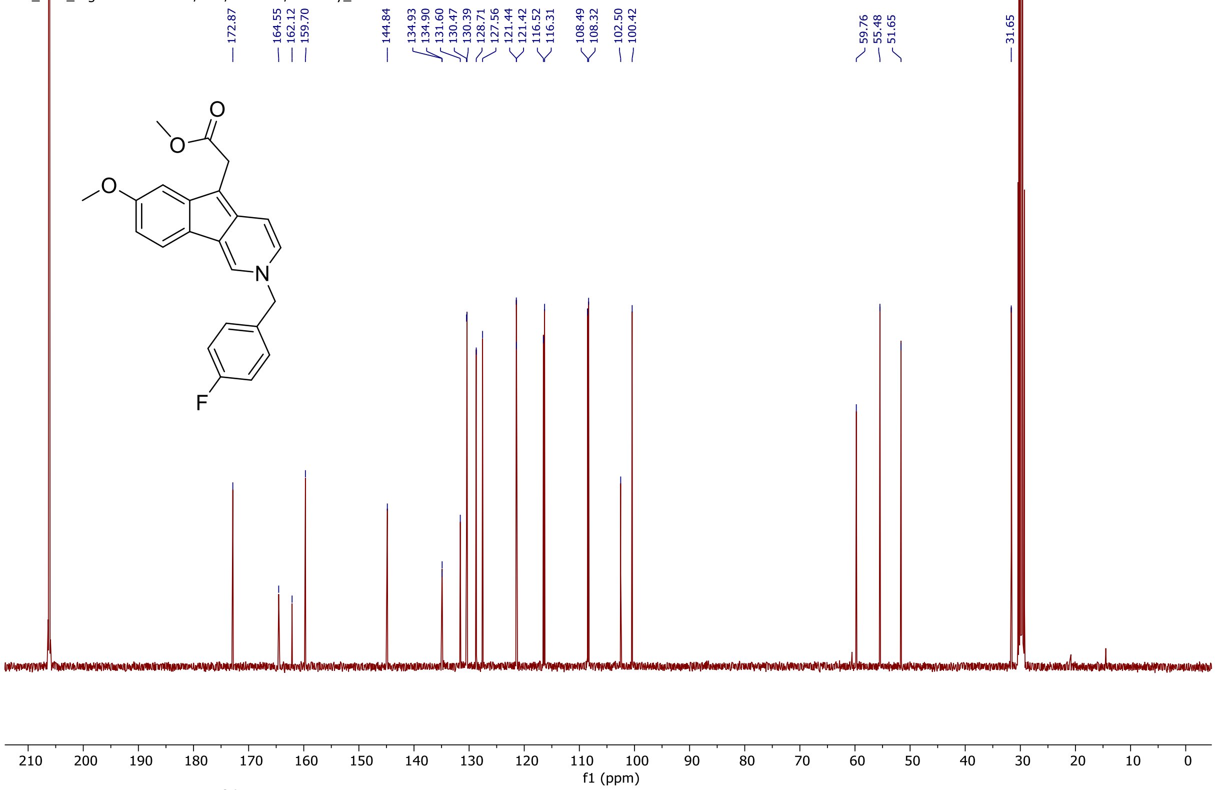

20201211-1135-B400_B.12-3.13.fid
Ref 482-2
Group Greaney_M
F19_CPD_Night Acetone /mnt/nmrdata/Greaney_M m31962tw 3

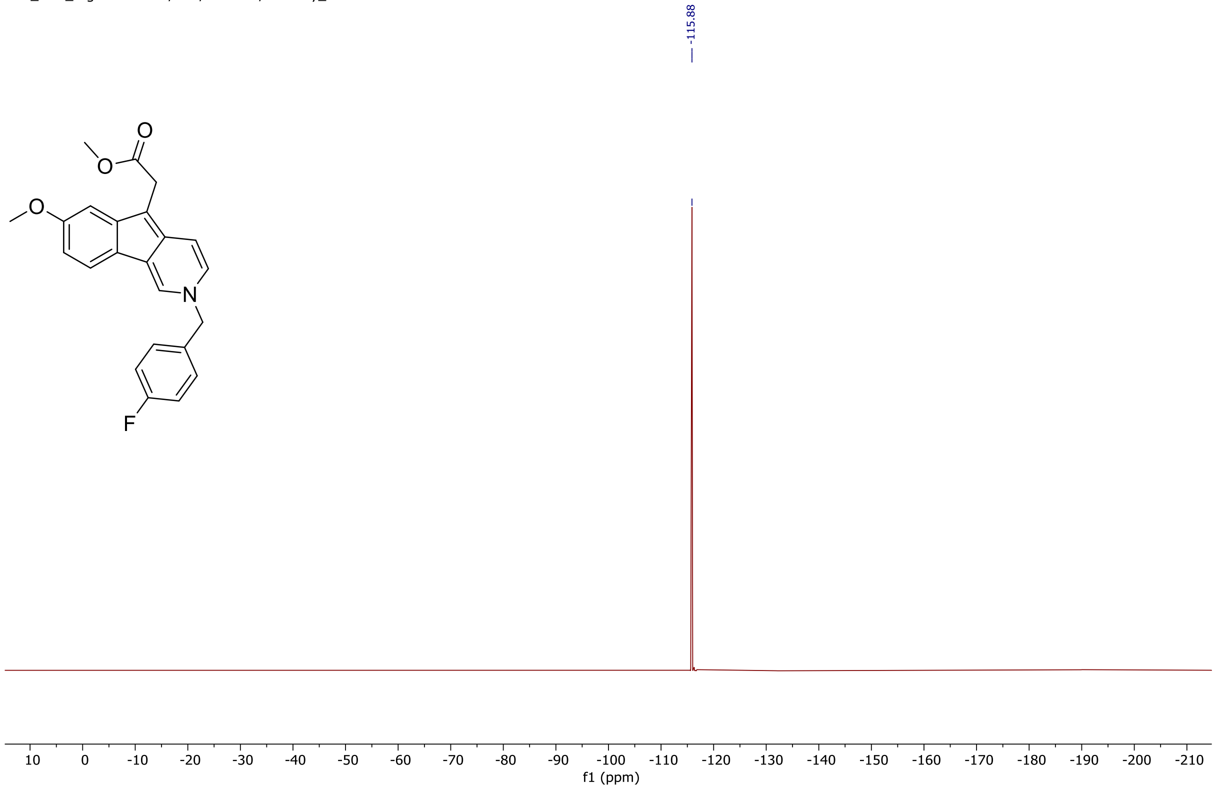

6i

20201210-1828-B400_B.11-2.10.fid
Ref 481-2
Group Greaney_M
H1_Night Acetone /mnt/nmrdata/Greaney_M m31962tw 2

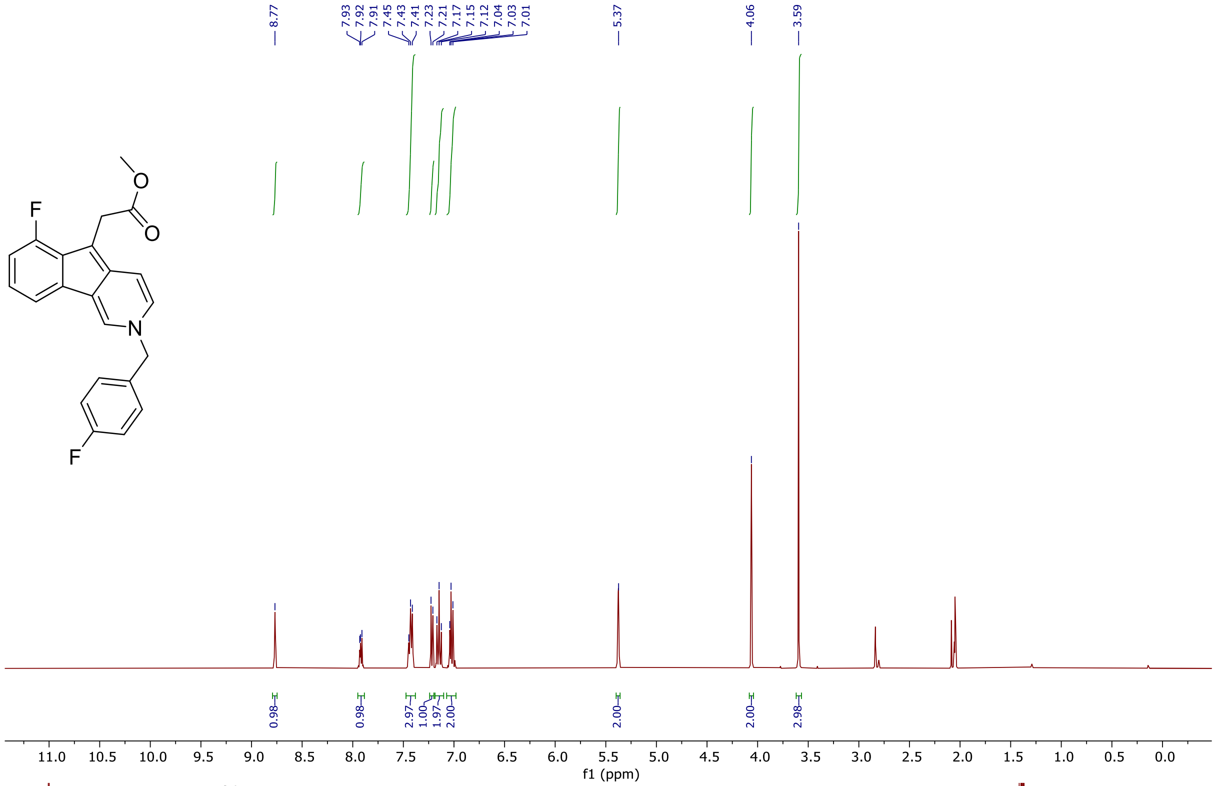

20201210-1828-B400_B.11-2.11.fid
Ref 481-2
Group Greaney_M
C13_CPD_Night256 Acetone /mnt/nmrdata/Greaney_M m31962tw 2

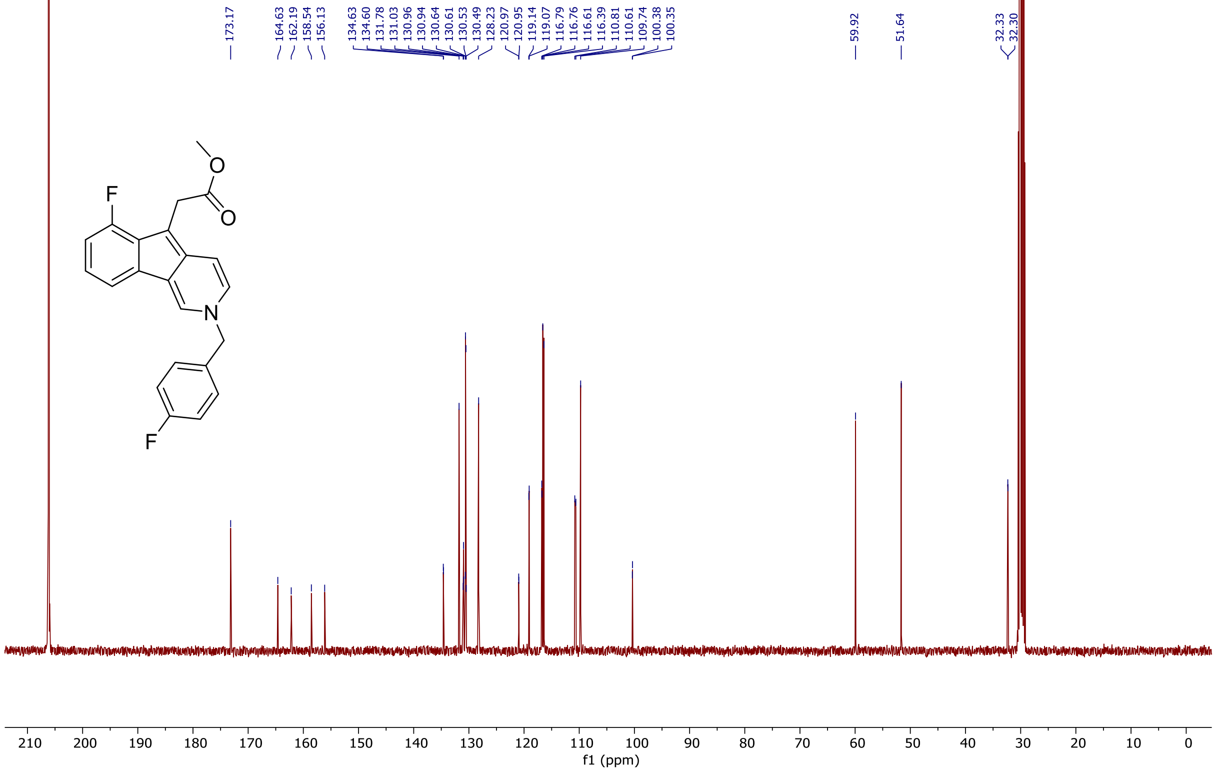

20201210-1828-B400_B.11-2.12.fid
Ref 481-2
Group Greaney_M
F19_NoCPD_Night Acetone /mnt/nmrdata/Greaney_M m31962tw 2

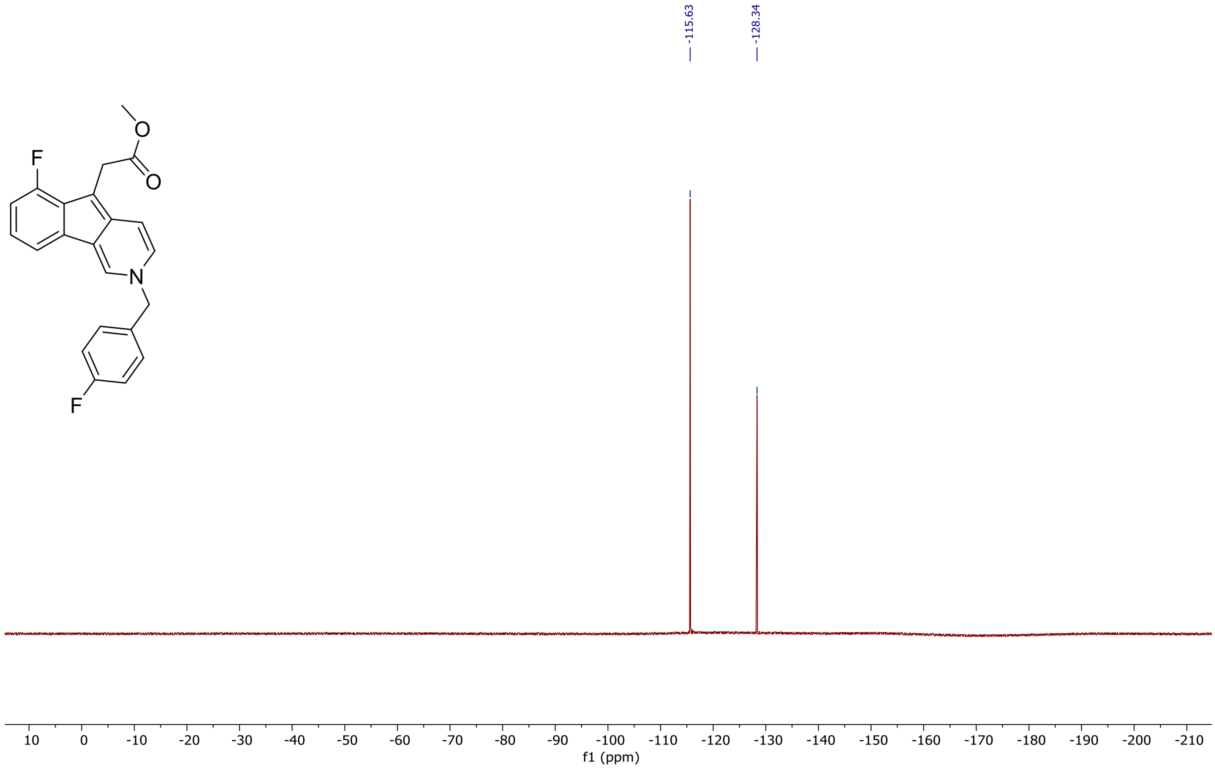

6j

20201210-1828-B400_B.11-3.10.fid
Ref 481-3
Group Greaney_M
H1_Night Acetone /mnt/nmrdata/Greaney_M m31962tw 3

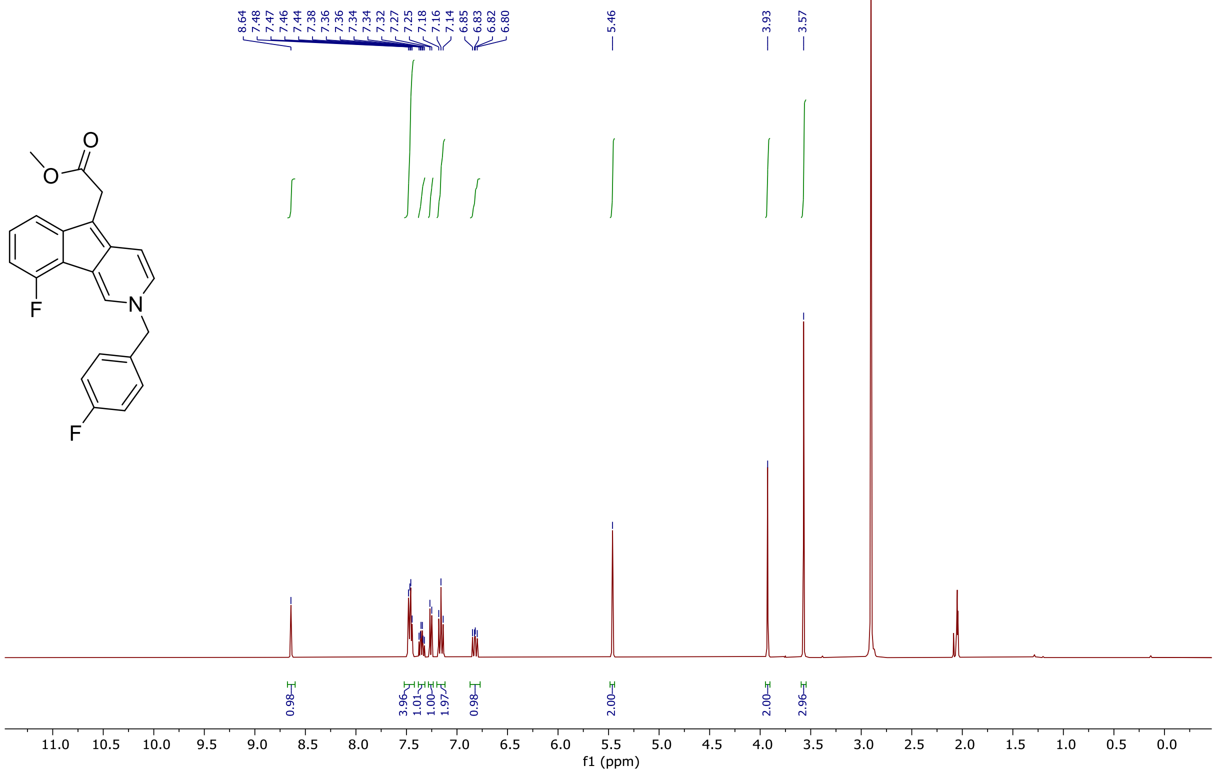

20201210-1828-B400_B.11-3.11.fid
Ref 481-3
Group Greaney_M
C13_CPD_Night256 Acetone /mnt/nmrdata/Greaney_M m31962tw 3

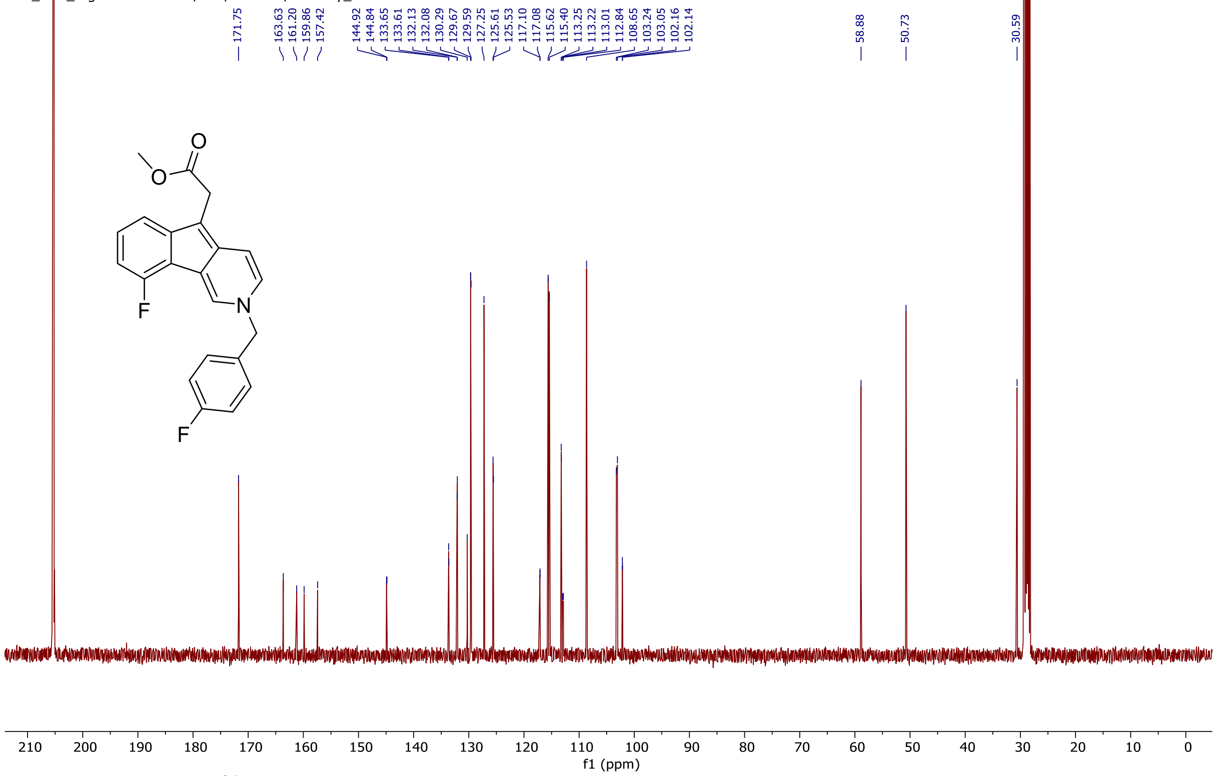

20201210-1828-B400_B.11-3.12.fid
Ref 481-3
Group Greaney_M
F19_NoCPD_Night Acetone /mnt/nmrdata/Greaney_M m31962tw 3

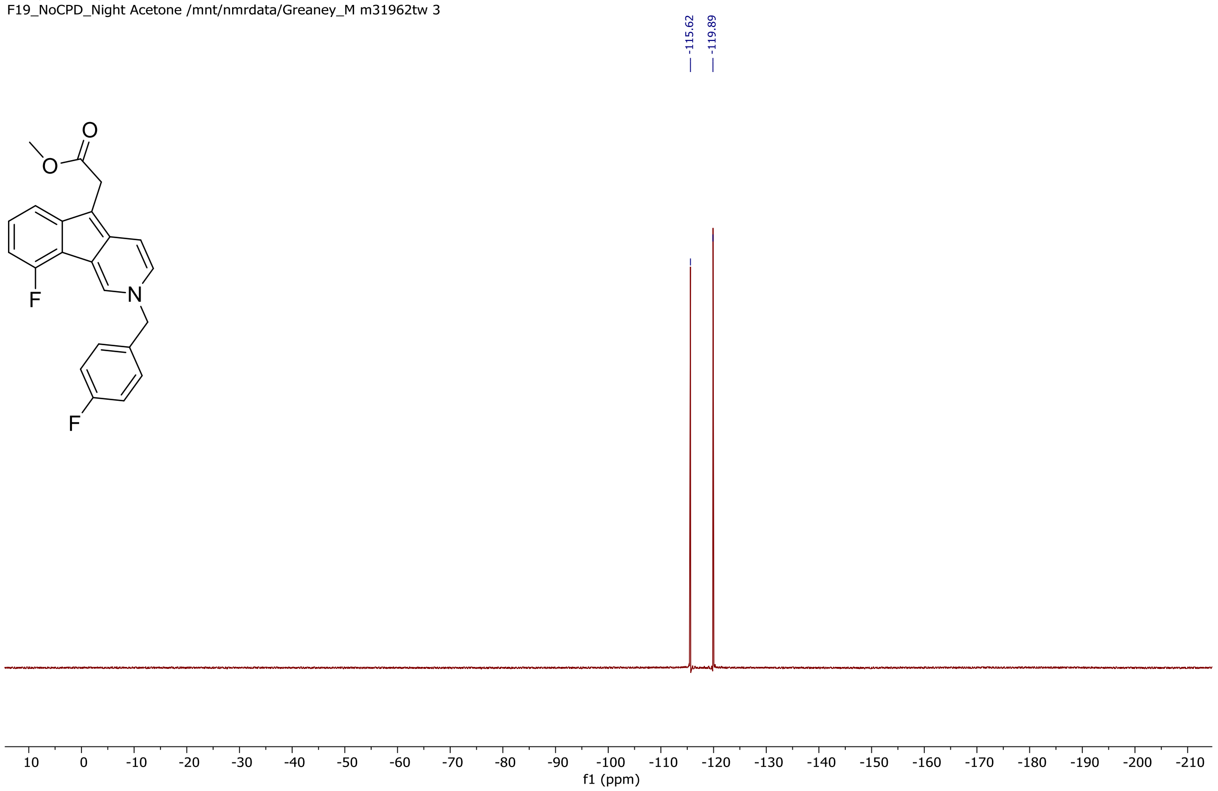

6k

20201208-1852-B400_B.11-45.10.fid
Ref 482-1
Group Greaney_M
H1_Night Acetone /mnt/nmrdata/Greaney_M m31962tw 45

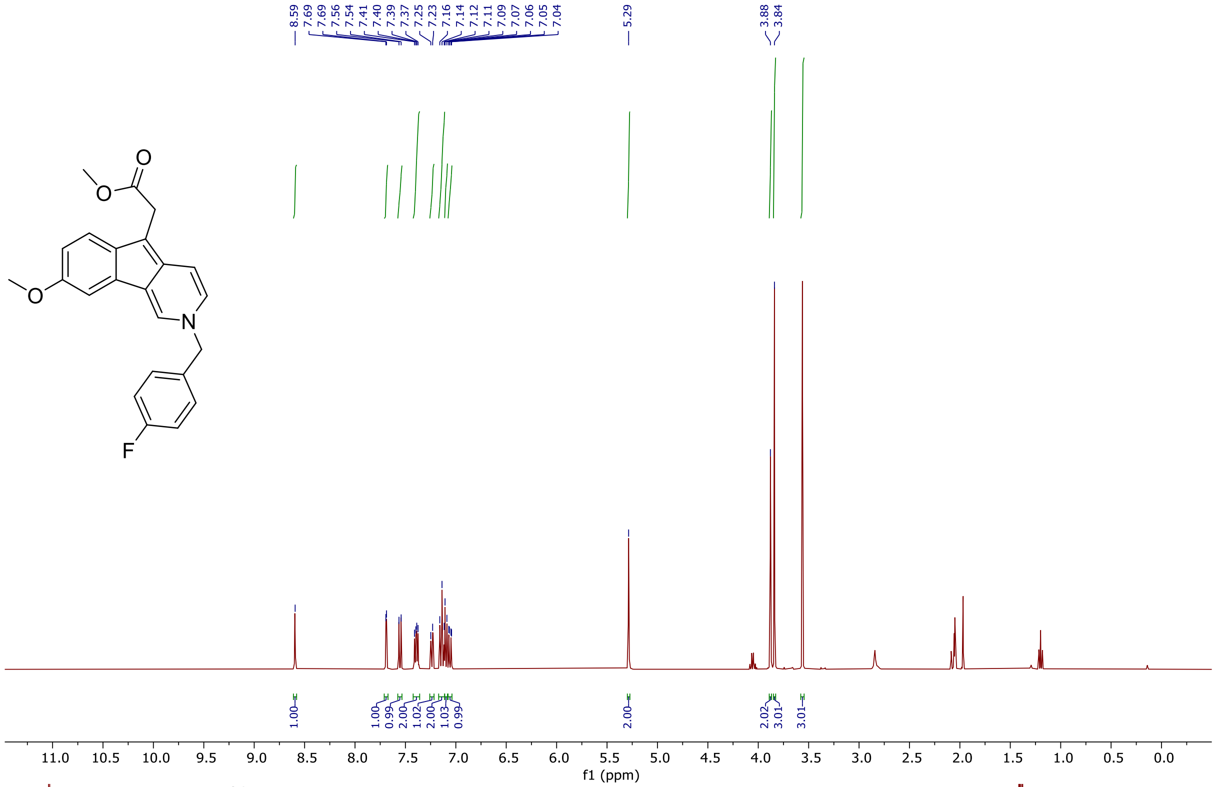

20201208-1852-B400_B.11-45.11.fid
Ref 482-1
Group Greaney_M
C13_CPD_Night256 Acetone /mnt/nmrdata/Greaney_M m31962tw 45

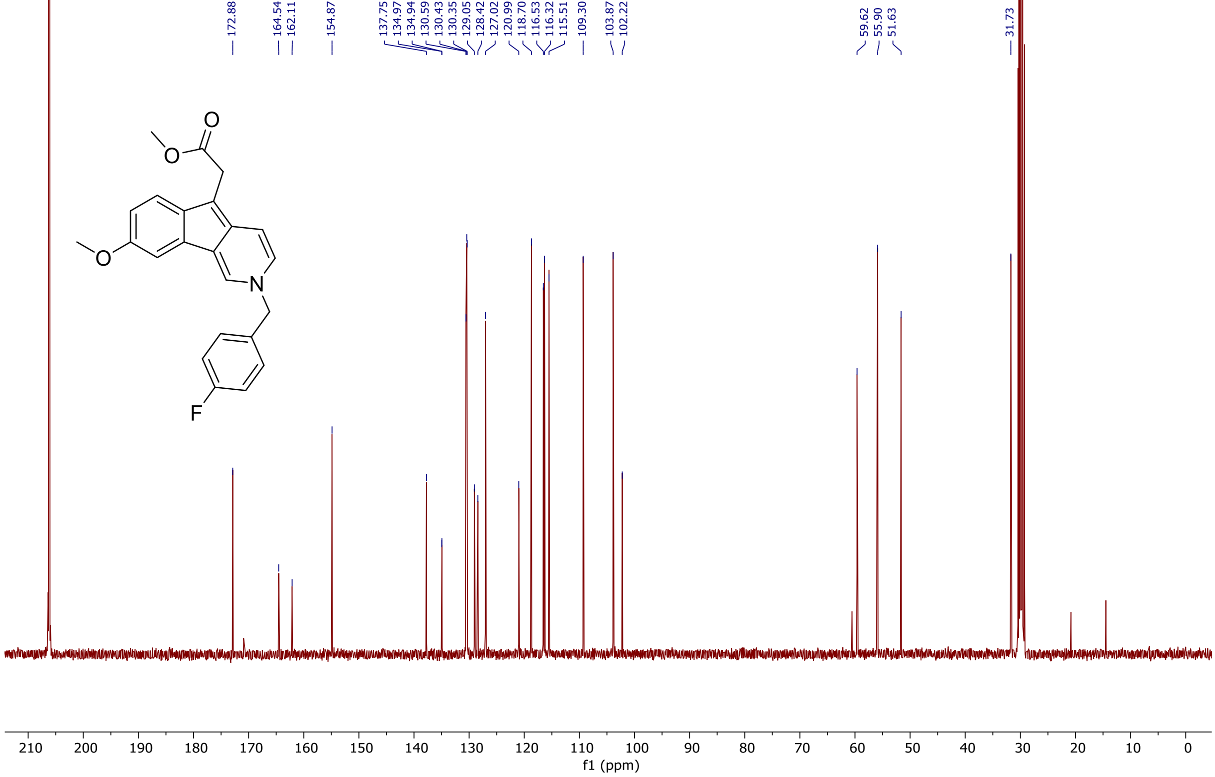

20201211-1134-B400_B.12-2.11.fid
Ref 482-1
Group Greaney_M
F19_CPD_Night Acetone /mnt/nmrdata/Greaney_M m31962tw 2

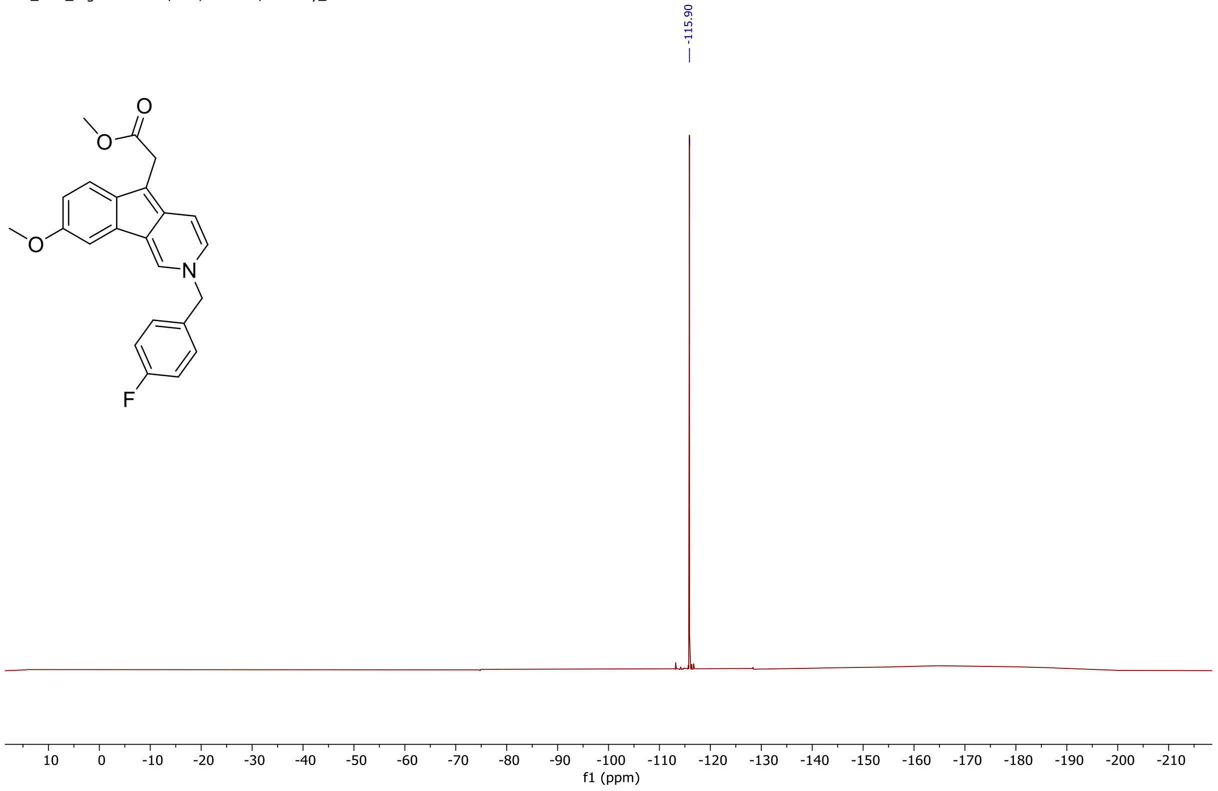

6l

20201208-1853-B400_B.11-47.10.fid
Ref 482-3
Group Greaney_M
H1_Night Acetone /mnt/nmrdata/Greaney_M m31962tw 47

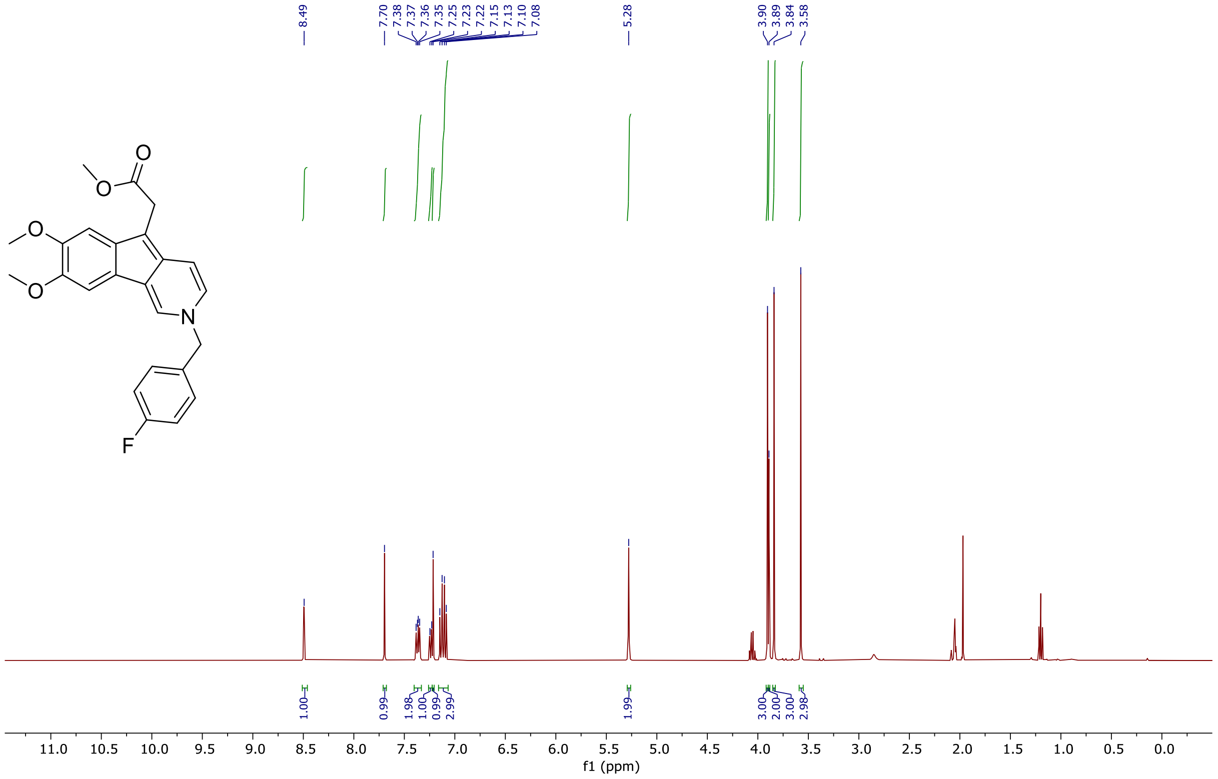

20201208-1853-B400_B.11-47.11.fid
Ref 482-3
Group Greaney_M
C13_CPD_Night256 Acetone /mnt/nmrdata/Greaney_M m31962tw 47

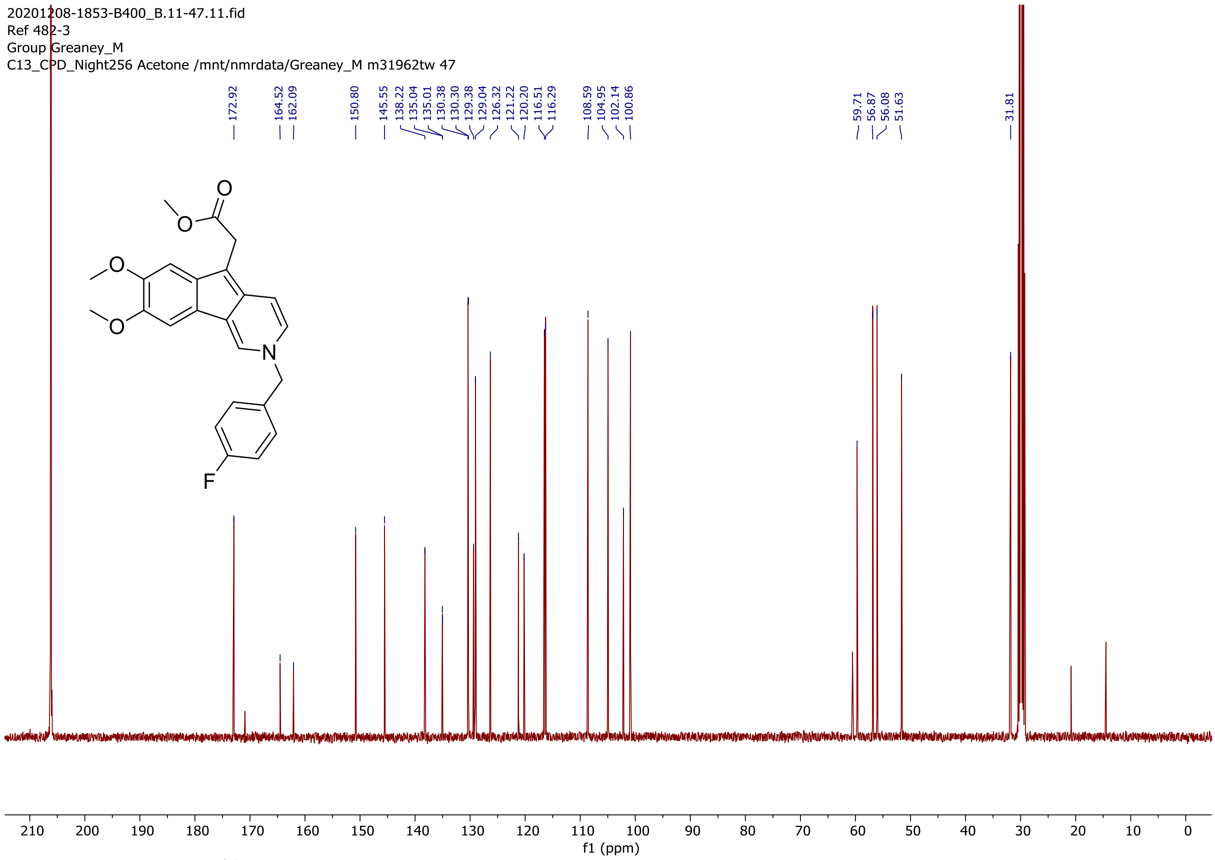

20201211-1136-B400_B.12-4.12.fid
Ref 482-3
Group Greaney_M
F19_CPD_Night Acetone /mnt/nmrdata/Greaney_M m31962tw 4

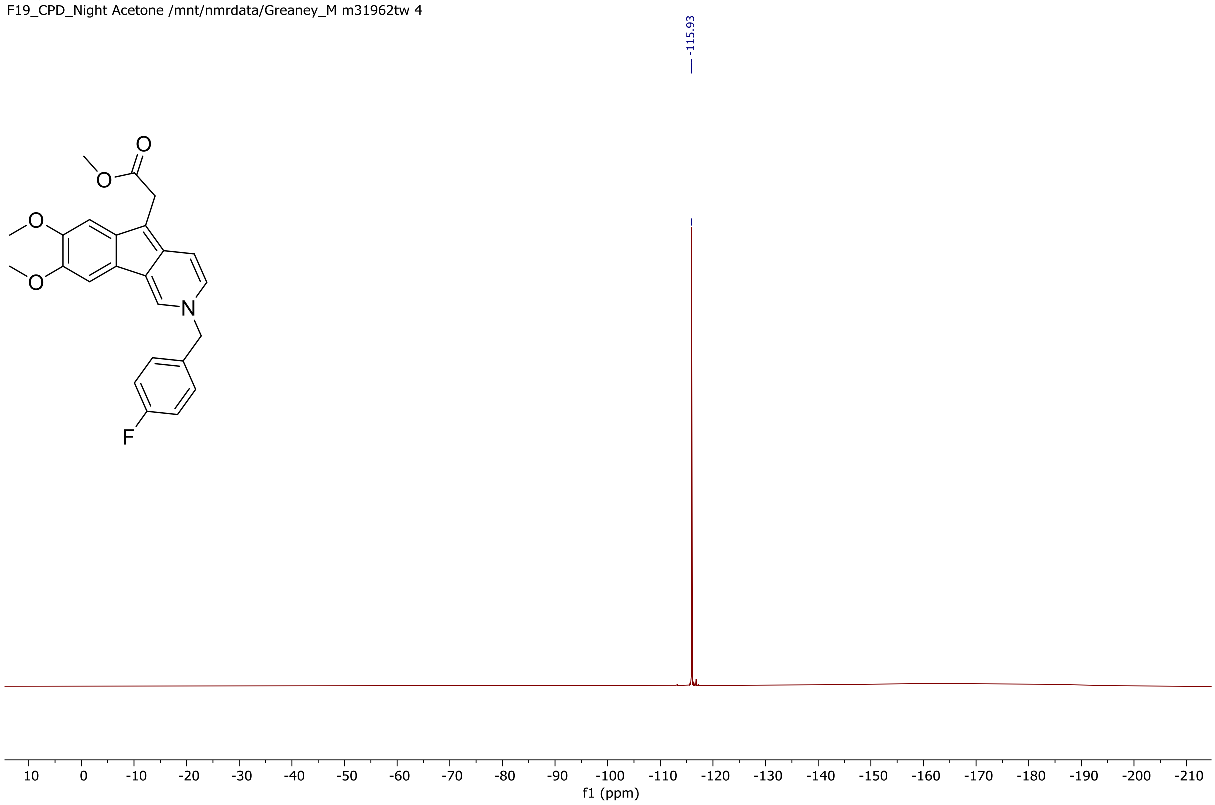

6m

20201208-1852-B400_B.11-44.10.fid
Ref 481-1
Group Greaney_M
H1_Day Acetone /mnt/nmrdata/Greaney_M m31962tw 44

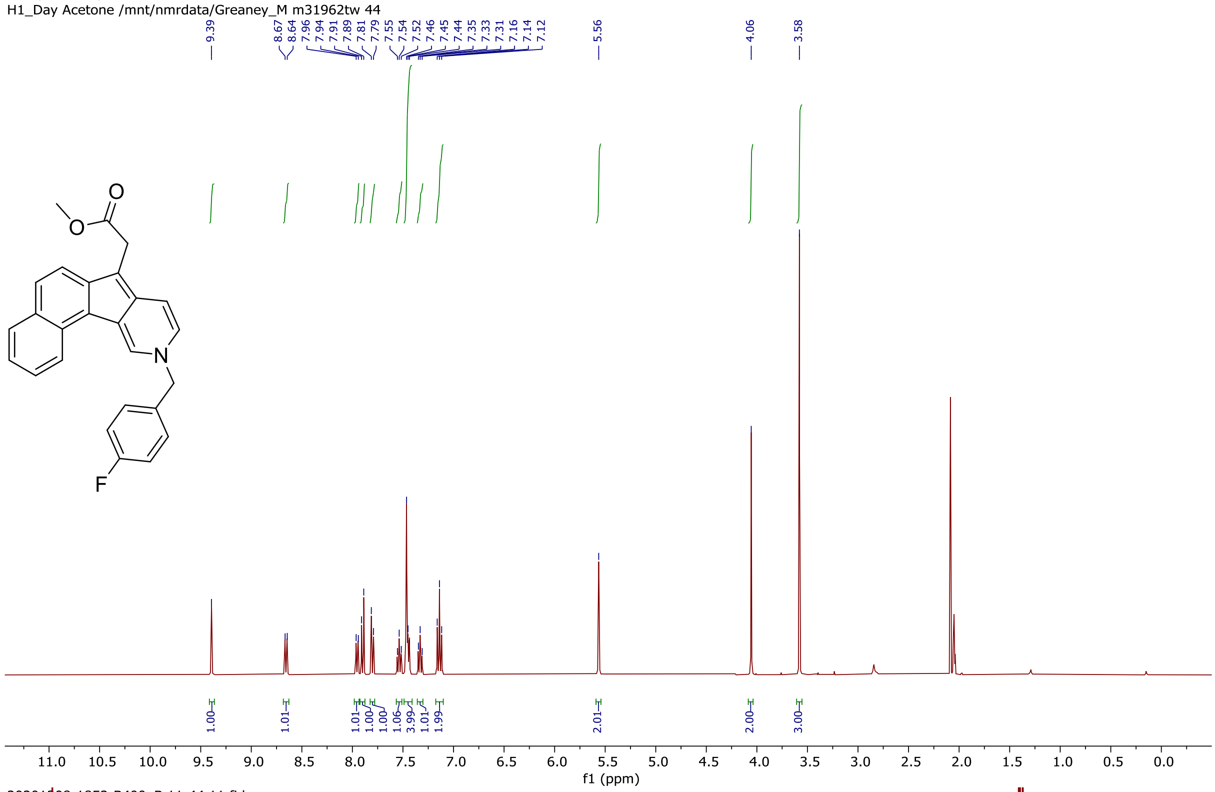

20201208-1852-B400_B.11-44.11.fid
Ref 481-1
Group Greaney_M
C13_CPD_Night256 Acetone /mnt/nmrdata/Greaney_M m31962tw 44

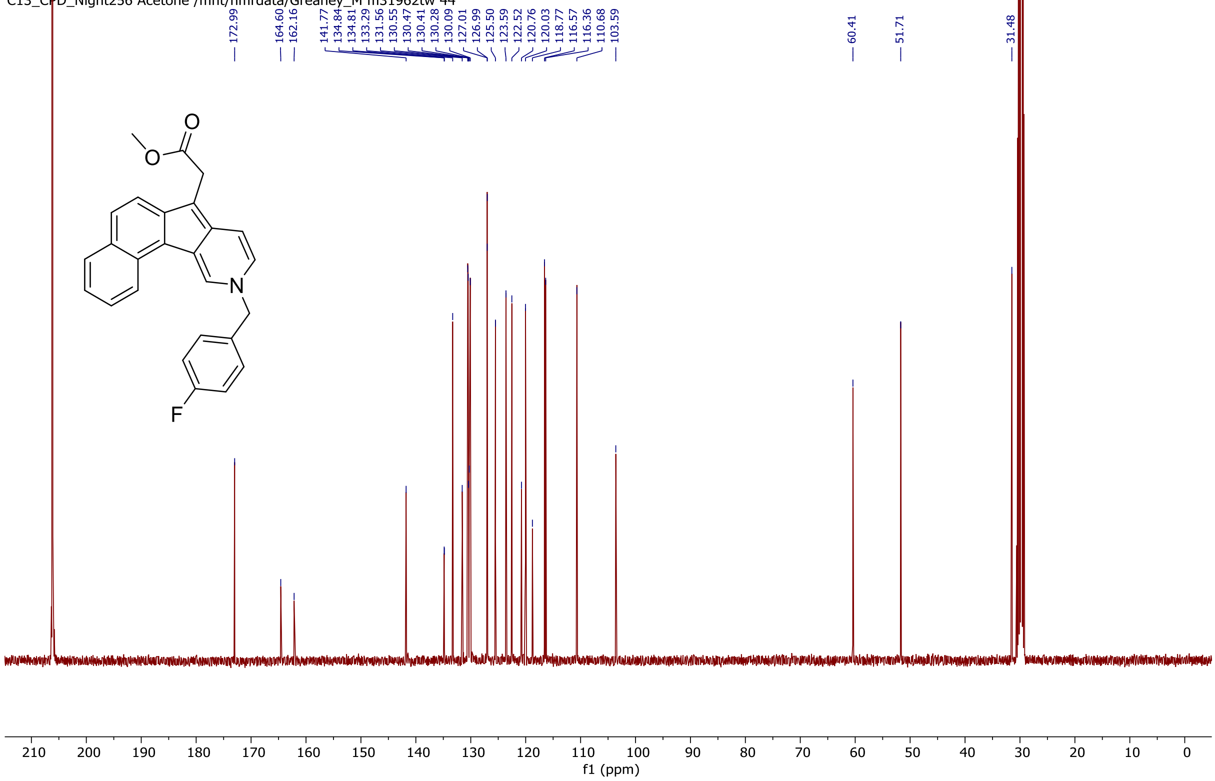

20201211-1134-B400_B.12-1.11.fid
Ref 481-1
Group Greaney_M
F19_CPD_Night Acetone /mnt/nmrdata/Greaney_M m31962tw 1

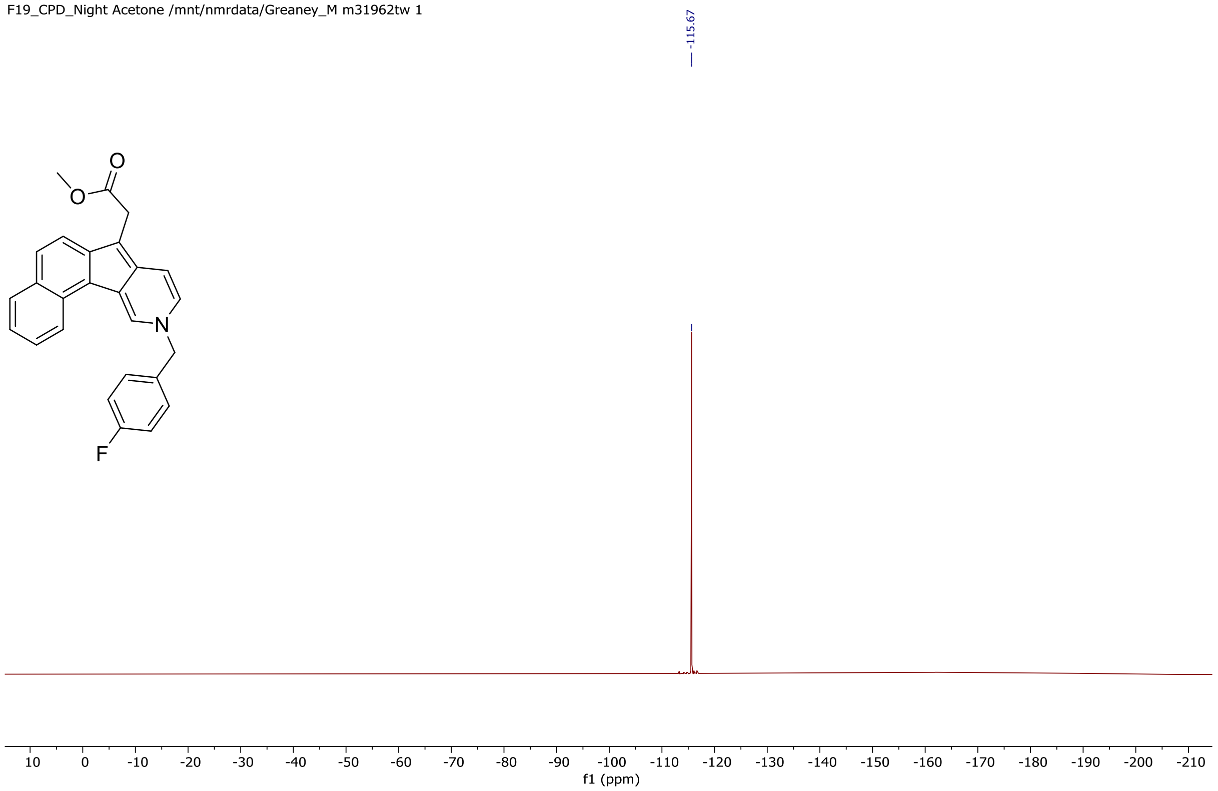

6n

20210311-1855-B400_B.11-25.10.fid
Ref 547-7
Group Greaney_M
H1_Night Acetone /mnt/nmrdata/Greaney_M m31962tw 25

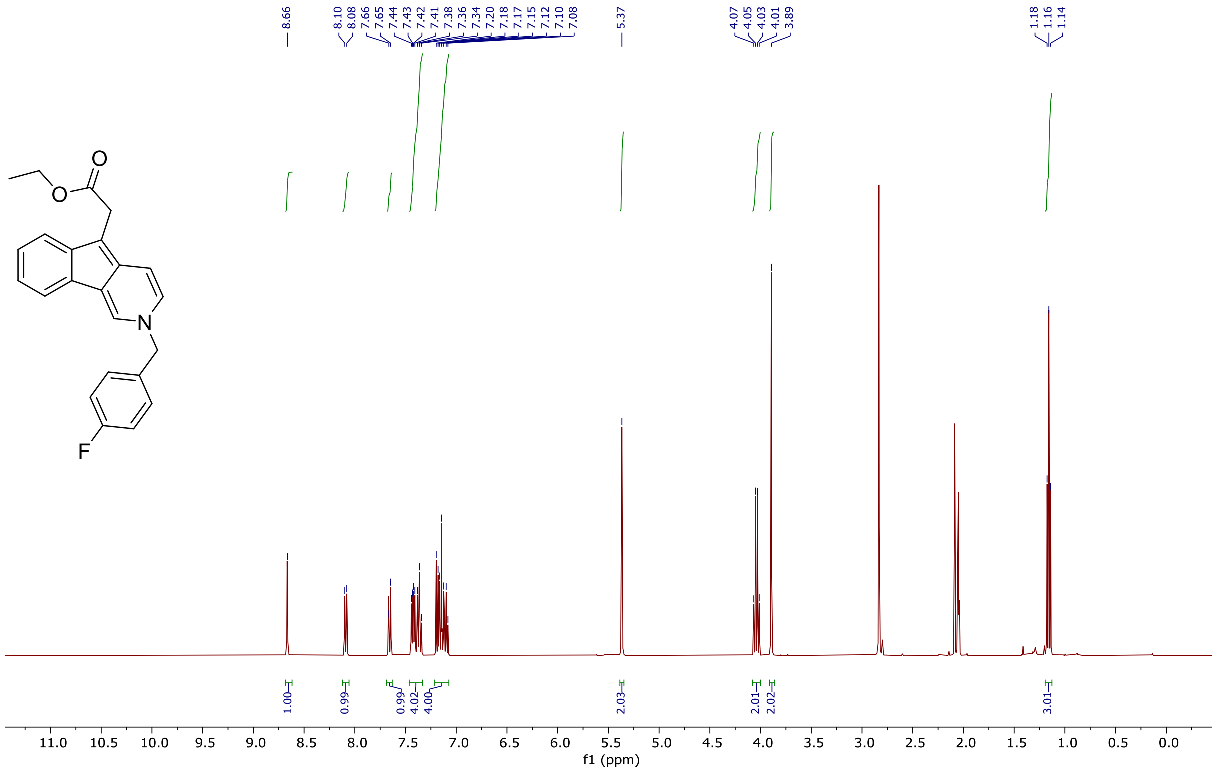

20210311-1855-B400_B.11-25.15.fid
Ref 547-7
Group Greaney_M
C13_CPD_Night1024 Acetone /mnt/nmrdata/Greaney_M m31962tw 25

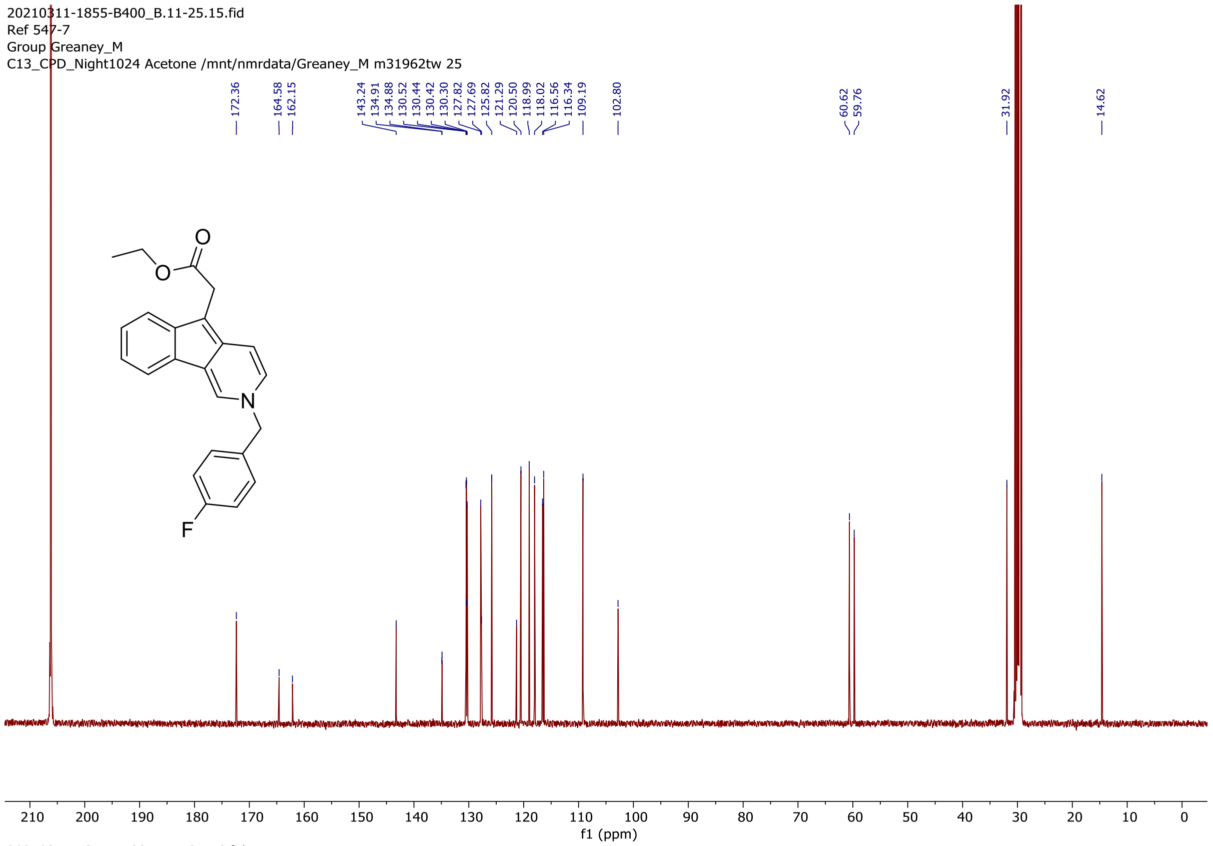

20210311-1855-B400_B.11-25.16.fid
Ref 547-7
Group Greaney_M
F19_NoCPD_Night Acetone /mnt/nmrdata/Greaney_M m31962tw 25

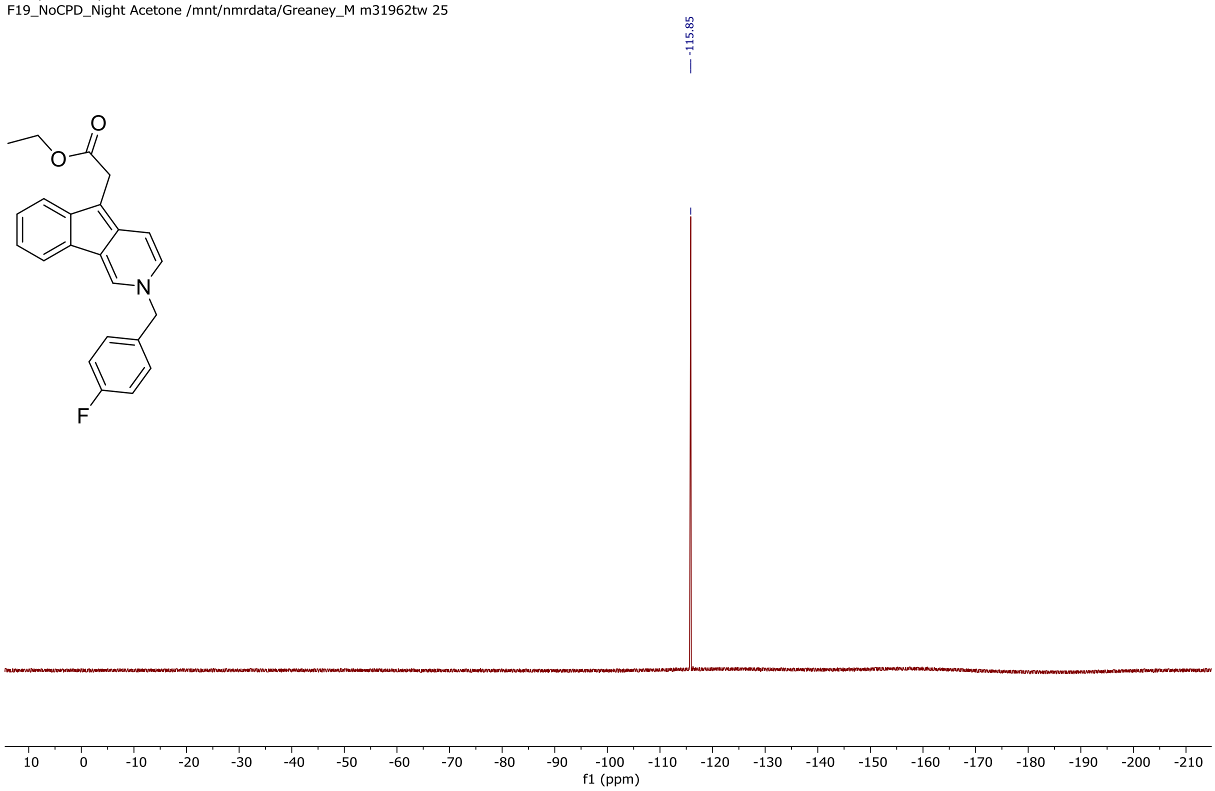

6o

20210423-1811-B400_B.11-15.10.fid
Ref 565-5
Group Greaney_M
H1_Night Acetone /mnt/nmrdata/Greaney_M m31962tw 15

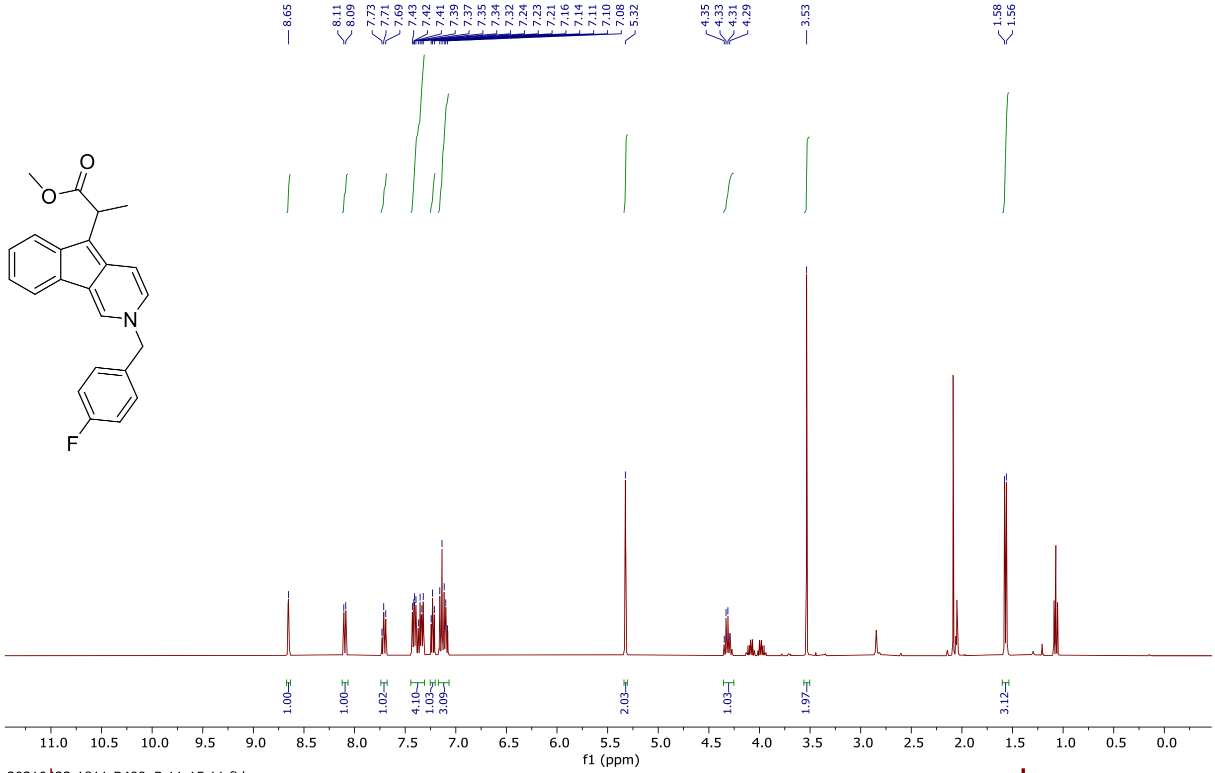

20210423-1811-B400_B.11-15.11.fid
Ref 565-5
Group Greaney_M
C13_CPD_Night256 Acetone /mnt/nmrdata/Greaney_M m31962tw 15

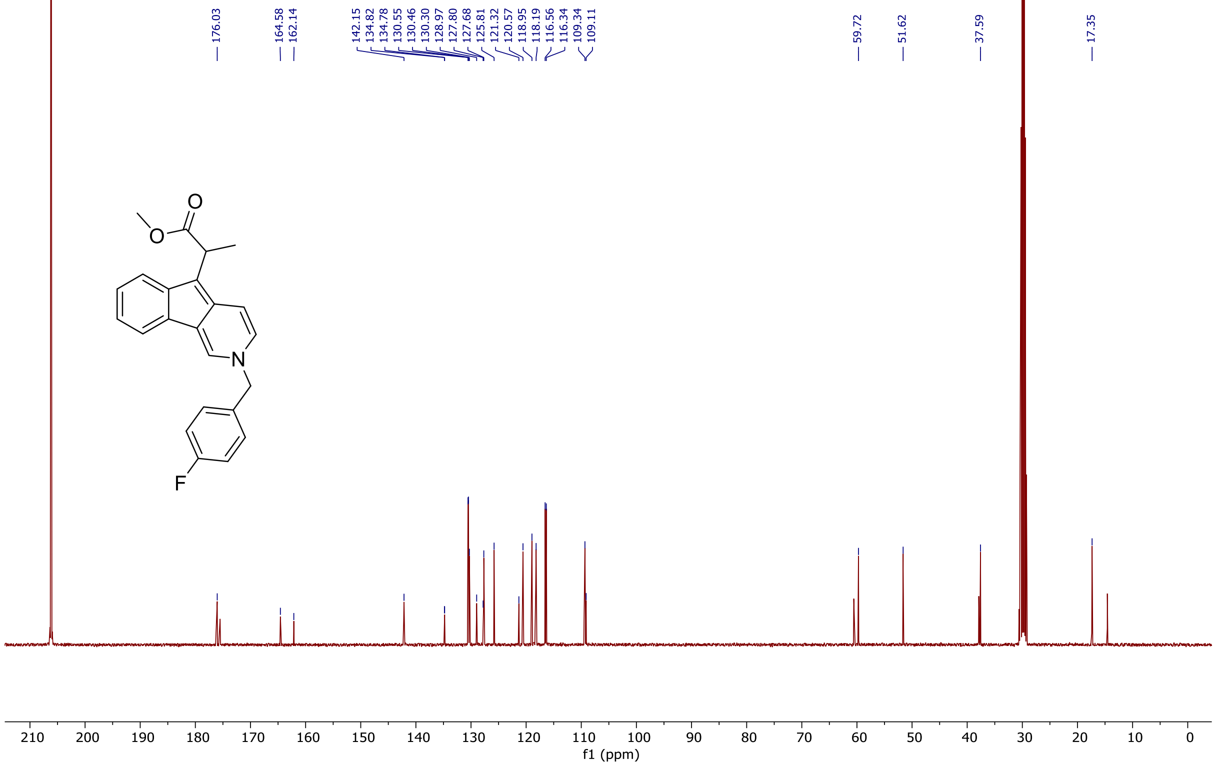

20210423-1811-B400_B.11-15.15.fid
Ref 565-5
Group Greaney_M
F19_NoCPD_Night Acetone /mnt/nmrdata/Greaney_M m31962tw 15

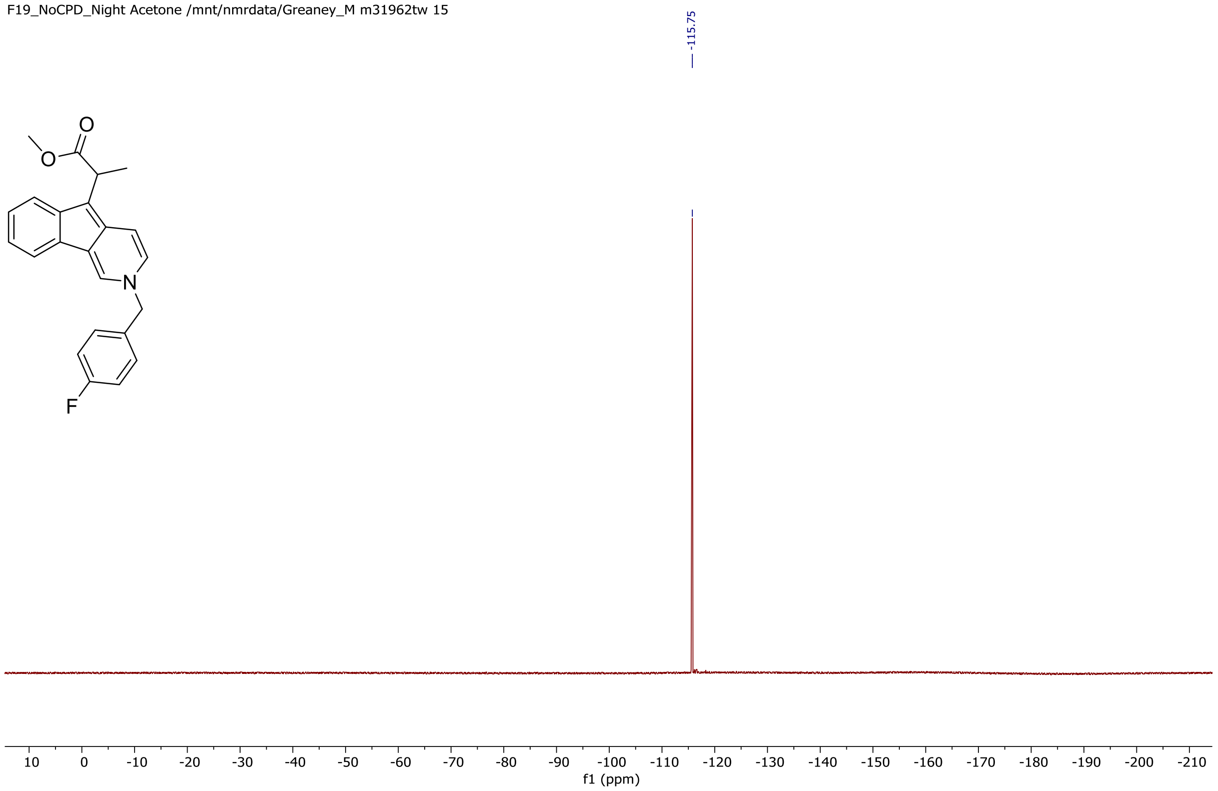

6p

20210309-1843-B400_B.11-14.10.fid
Ref 549-2
Group Greaney_M
H1_Night Acetone /mnt/nmrdata/Greaney_M m31962tw 14

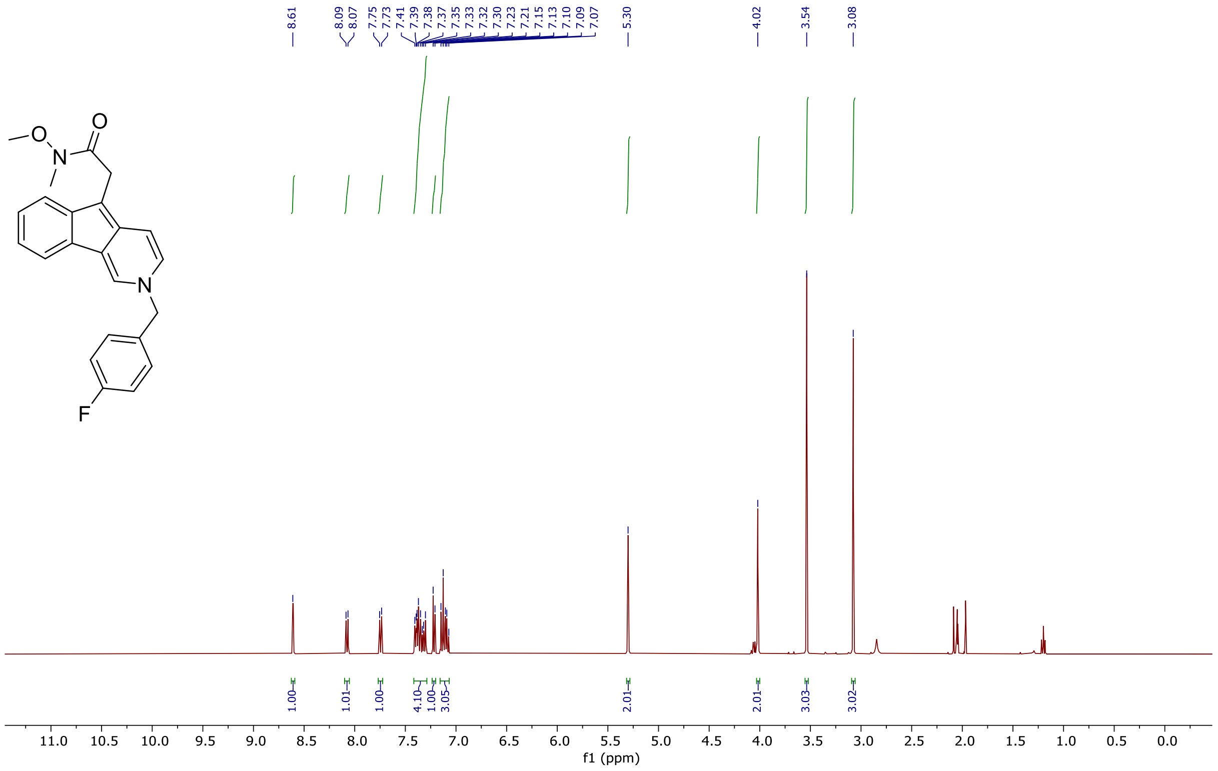

20210309-1843-B400_B.11-14.11.fid
Ref 549-2
Group Greaney_M
C13_CPD_Night256 Acetone /mnt/nmrdata/Greaney_M m31962tw 14

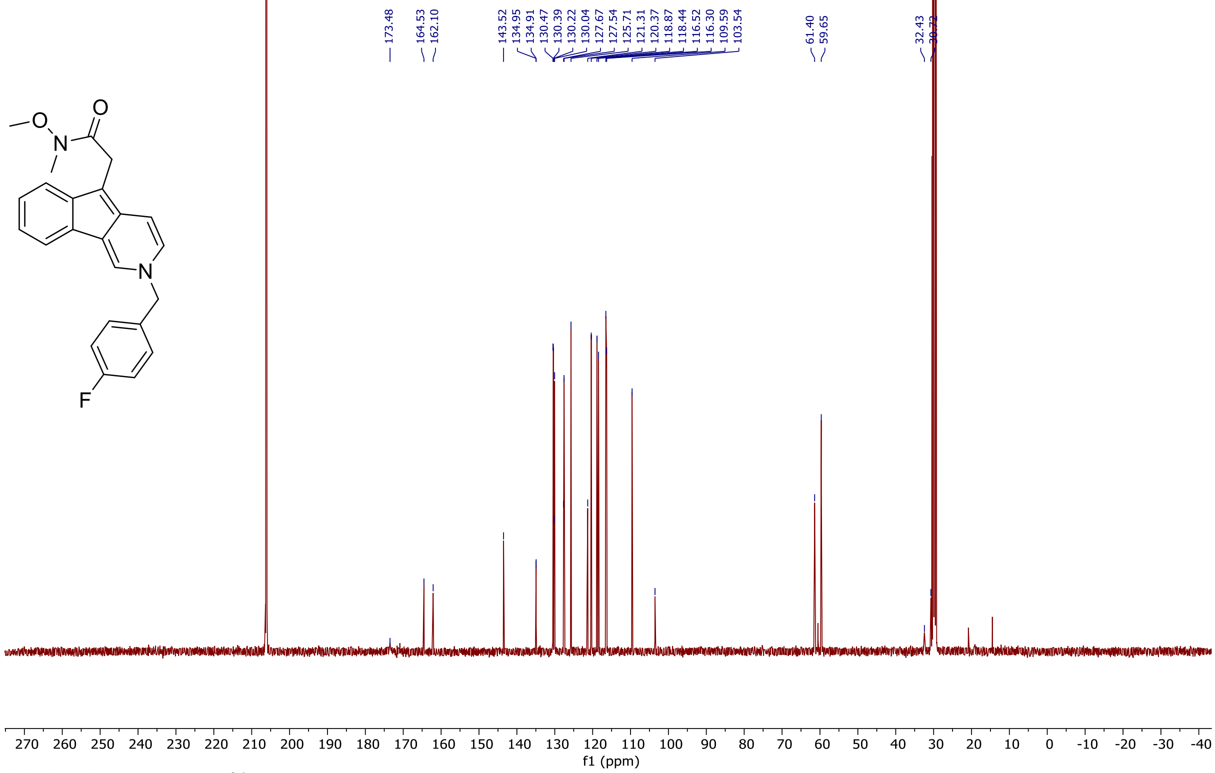

20210309-1843-B400_B.11-14.15.fid
Ref 549-2
Group Greaney_M
F19_NoCPD_Night Acetone /mnt/nmrdata/Greaney_M m31962tw 14

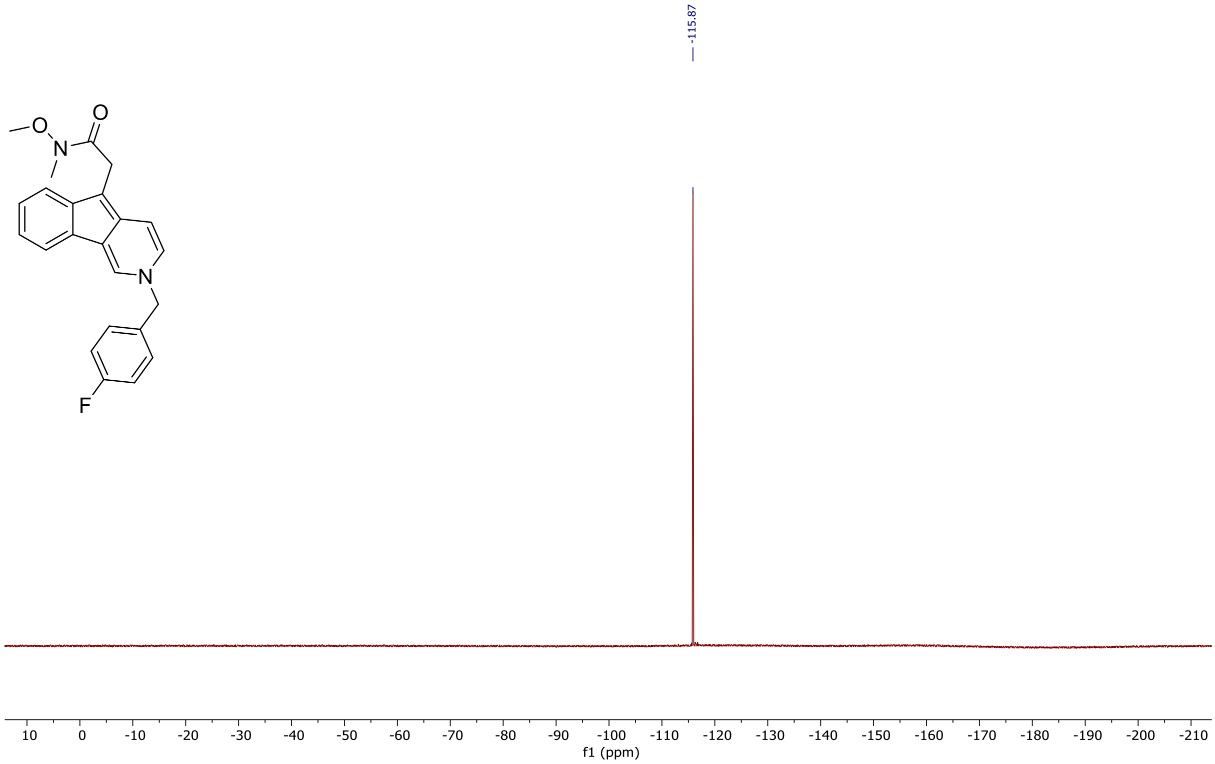

20210125-1647-B400_B.11-1.10.fid
Ref 501-8
Group Greaney_M
H1_Day Acetone /mnt/nmrdata/Greaney_M m31962tw 1

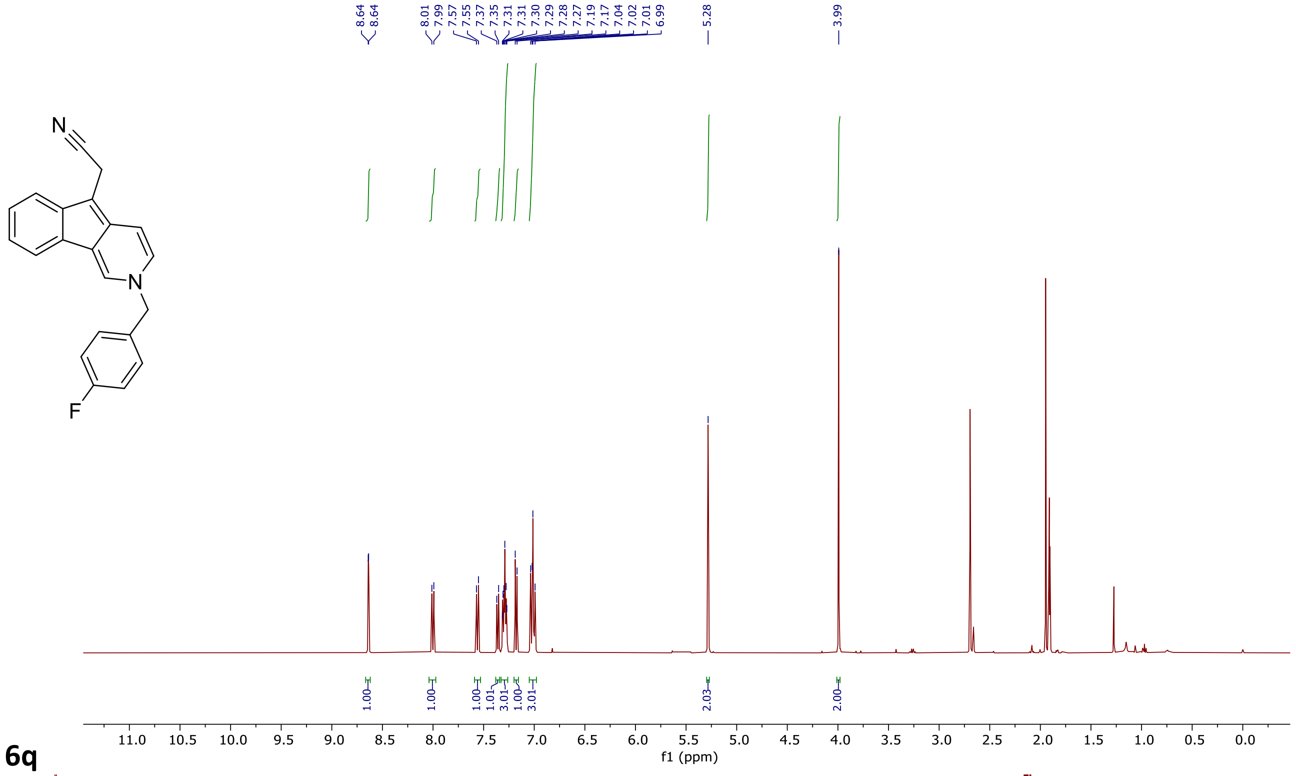

6q

20210125-1647-B400_B.11-1.11.fid
Ref 501-8
Group Greaney_M
C13_CPD_Night256 Acetone /mnt/nmrdata/Greaney_M m31962tw 1

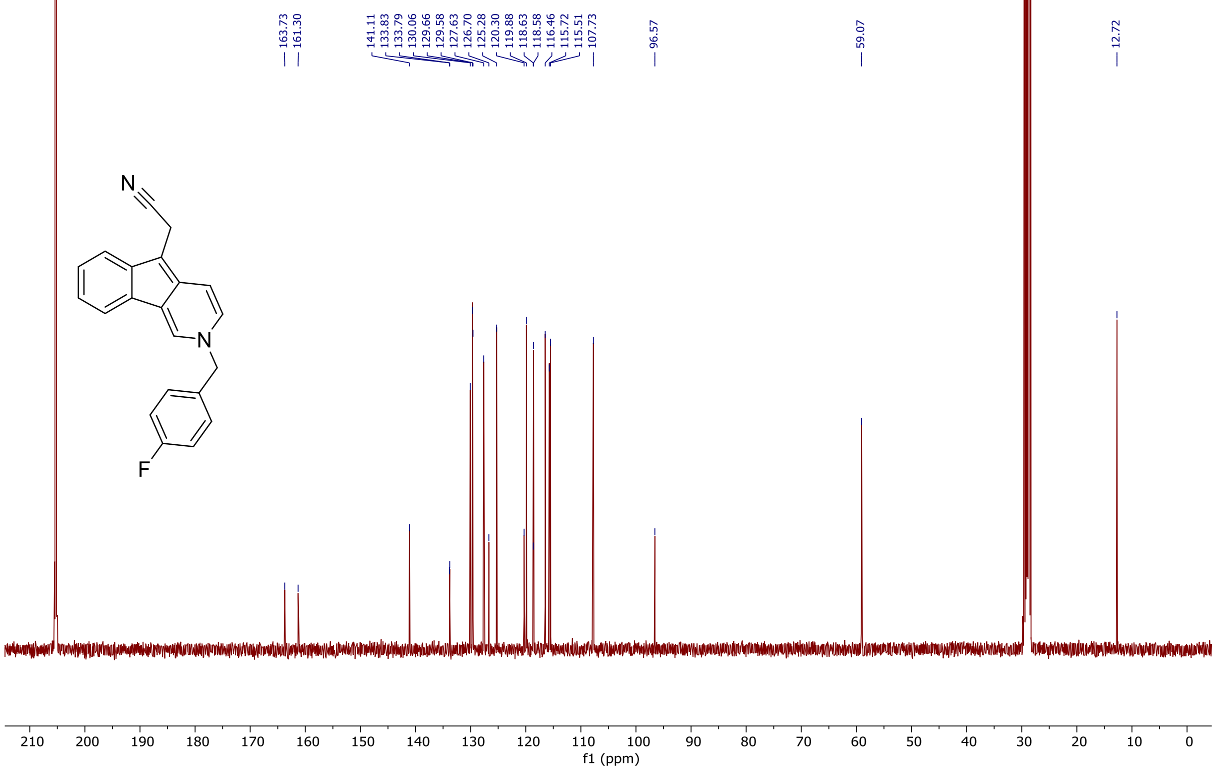

20210125-1647-B400_B.11-1.12.fid
Ref 501-9
Group Greaney_M
F19_NoCPD_Night Acetone /mnt/nmrdata/Greaney_M m31962tw 1

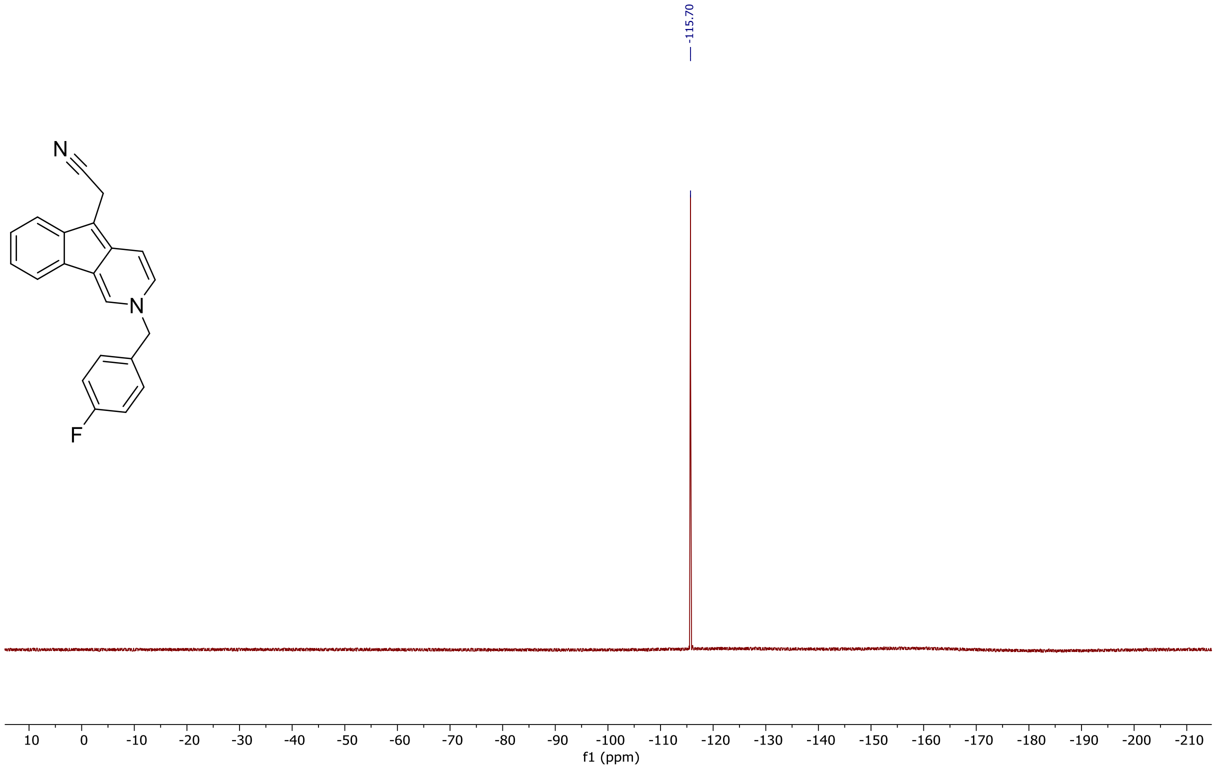

6r

20210126-1641-B400_B.11-19.10.fid
Ref 507-3
Group Greaney_M
H1_Night Acetone /mnt/nmrdata/Greaney_M m31962tw 19

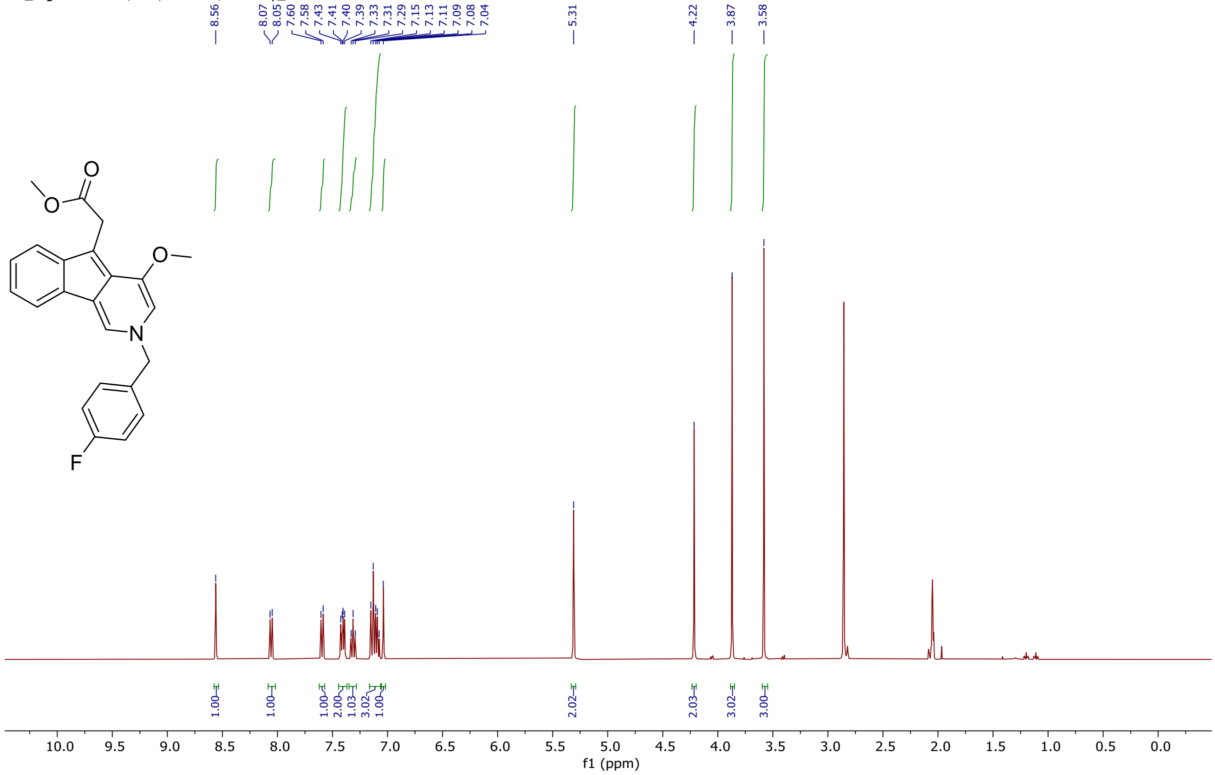

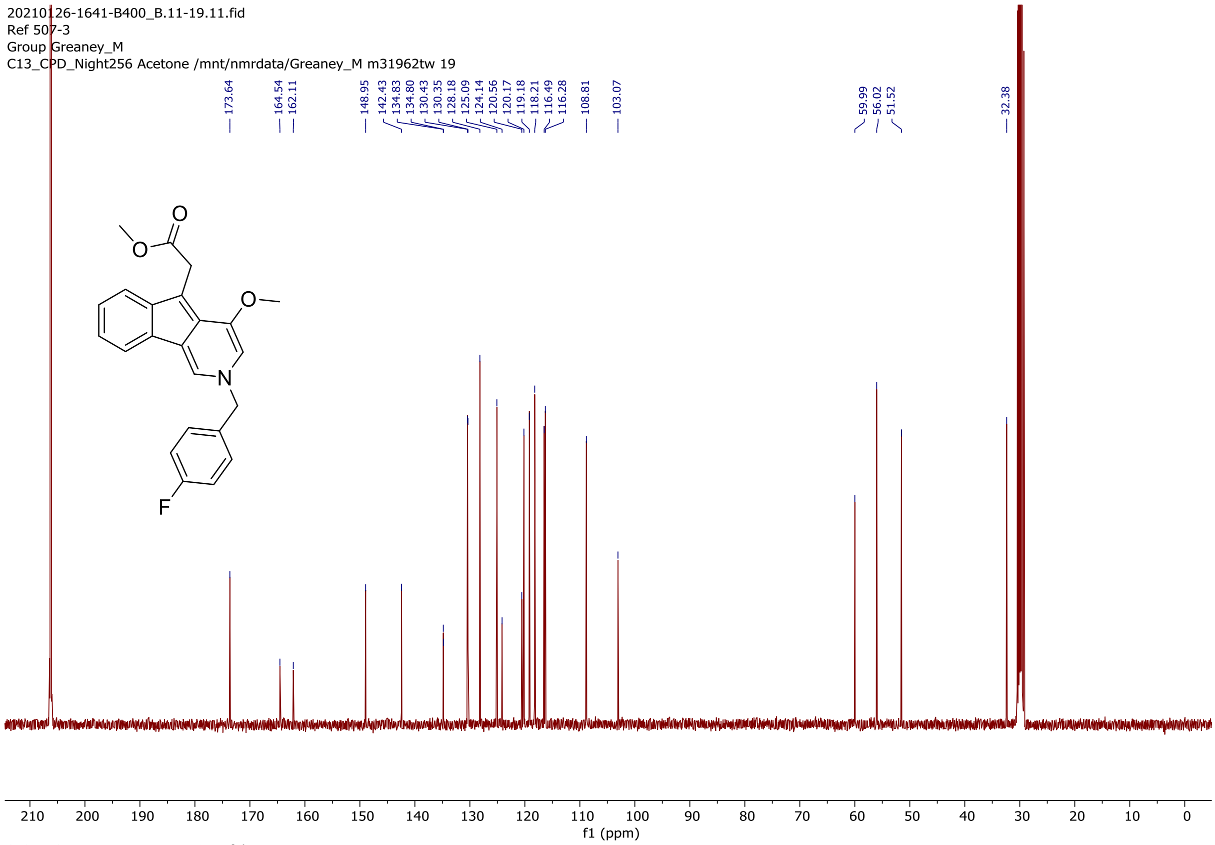
20210126-1641-B400_B.11-19.11.fid
Ref 507-3
Group Greaney_M
C13_CPD_Night256 Acetone /mnt/nmrdata/Greaney_M m31962tw 19
173.64
164.54
162.11
148.95
142.43
134.83
134.80
130.43
130.35
128.18
125.09
124.14
120.56
120.17
119.18
118.21
116.49
116.28
108.81
103.07
59.99
56.02
51.52
32.38
O
O
O
N
F
210 200 190 180 170 160 150 140 130 120 110 100 90 80 70 60 50 40 30 20 10 0
f1 (ppm)

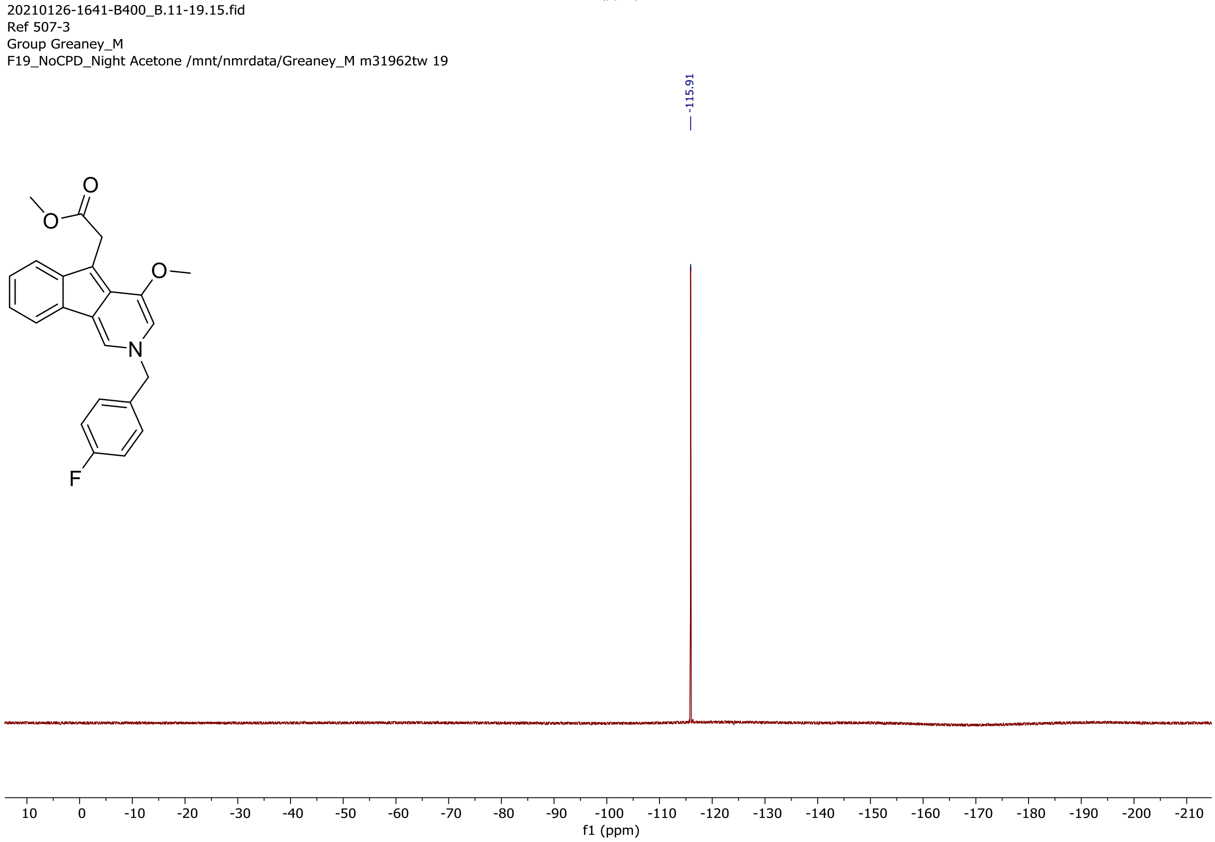
20210126-1641-B400_B.11-19.15.fid
Ref 507-3
Group Greaney_M
F19_NoCPD_Night Acetone /mnt/nmrdata/Greaney_M m31962tw 19
-115.91
O
O
O
N
F
10 0 -10 -20 -30 -40 -50 -60 -70 -80 -90 -100 -110 -120 -130 -140 -150 -160 -170 -180 -190 -200 -210
f1 (ppm)

6s

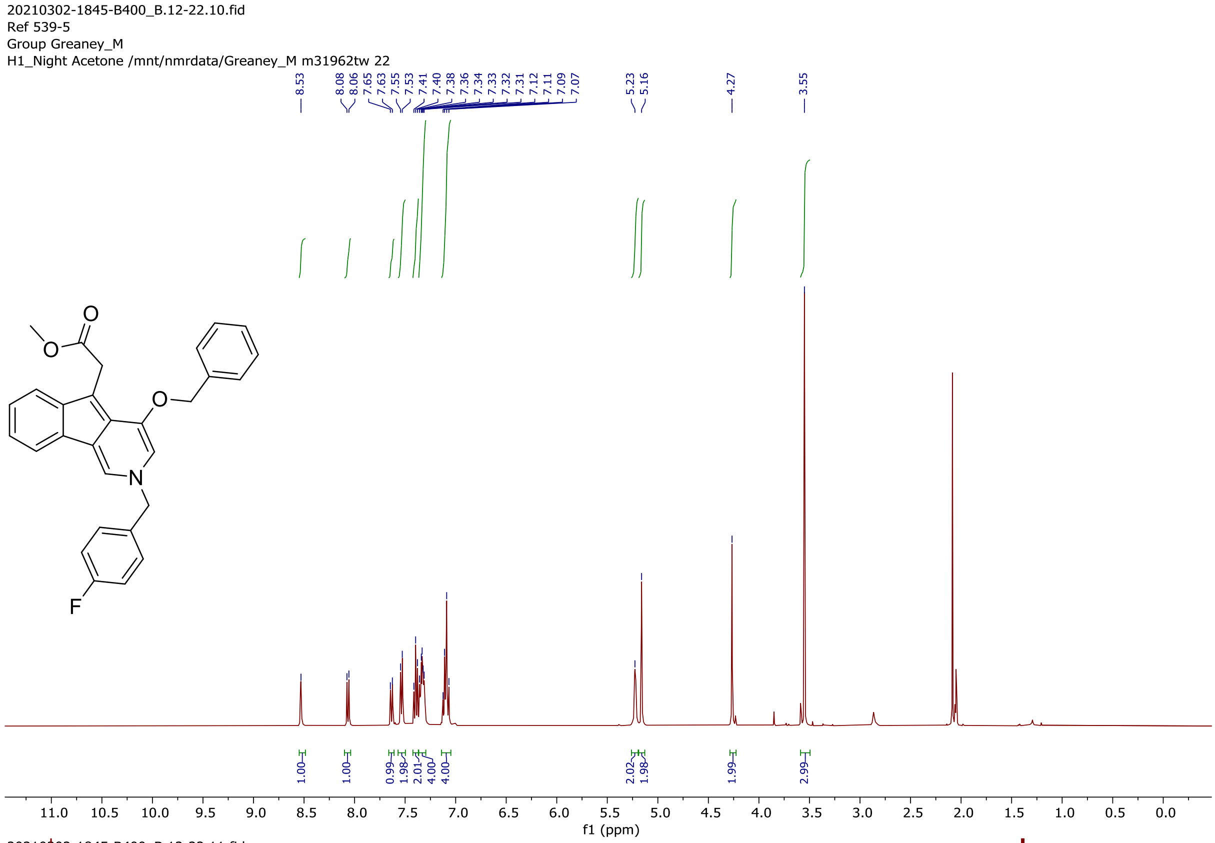

20210302-1845-B400_B.12-22.10.fid
Ref 539-5
Group Greaney_M
H1_Night Acetone /mnt/nmrdata/Greaney_M m31962tw 22
8.53
8.08
8.06
7.65
7.63
7.55
7.53
7.41
7.40
7.38
7.36
7.34
7.33
7.32
7.31
7.12
7.11
7.09
7.07
5.23
5.16
4.27
3.55
O
O
O
N
F
1.00
1.00
0.99
1.98
2.01
4.00
4.00
2.02
1.98
1.99
2.99
11.0 10.5 10.0 9.5 9.0 8.5 8.0 7.5 7.0 6.5 6.0 5.5 5.0 4.5 4.0 3.5 3.0 2.5 2.0 1.5 1.0 0.5 0.0
f1 (ppm)

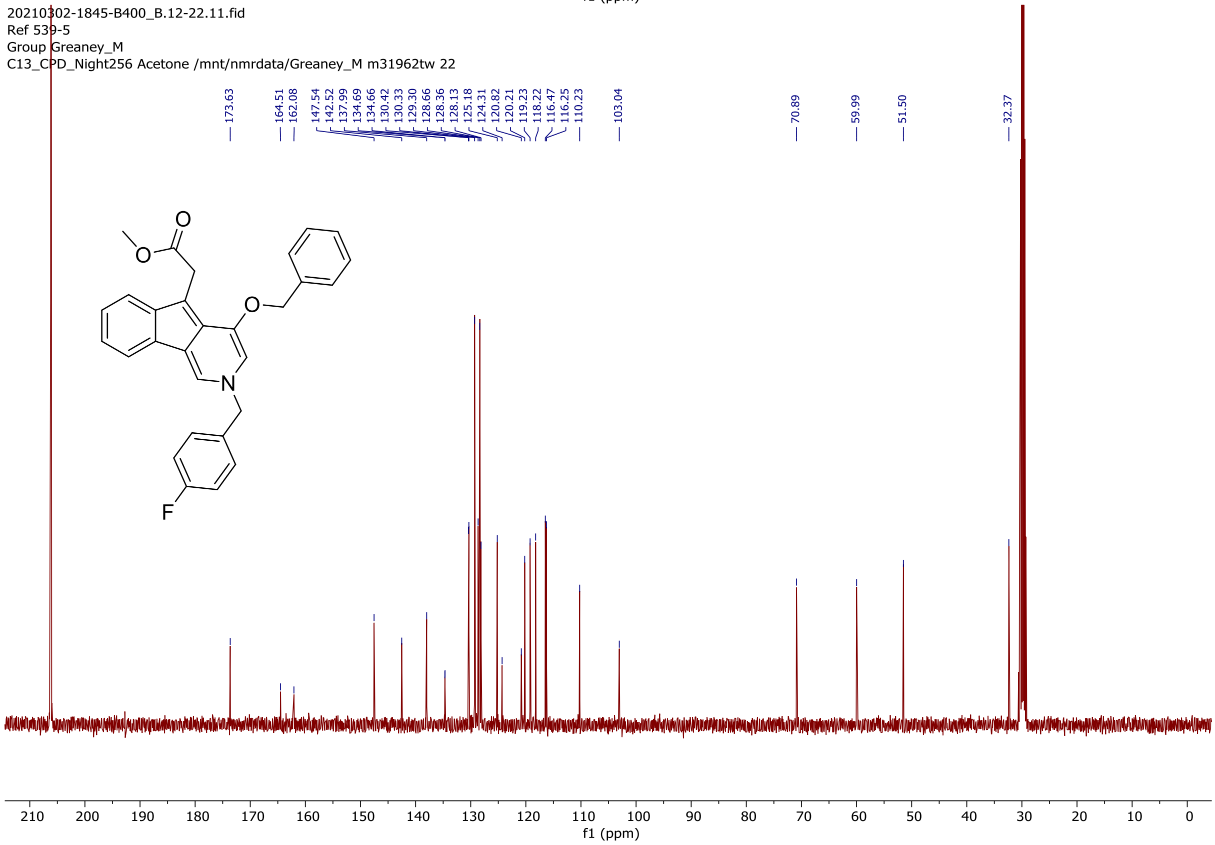

20210302-1845-B400_B.12-22.11.fid
Ref 539-5
Group Greaney_M
C13_CPD_Night256 Acetone /mnt/nmrdata/Greaney_M m31962tw 22
173.63
164.51
162.08
147.54
142.52
137.99
134.69
134.66
130.42
130.33
129.30
128.66
128.36
128.13
125.18
124.31
120.82
120.21
119.23
118.22
116.47
116.25
110.23
103.04
70.89
59.99
51.50
32.37
O
O
O
N
F
210 200 190 180 170 160 150 140 130 120 110 100 90 80 70 60 50 40 30 20 10 0
f1 (ppm)

20210302-1845-B400_B.12-22.12.fid
Ref 539-5
Group Greaney_M
F19_CPD_Night Acetone /mnt/nmrdata/Greaney_M m31962tw 22

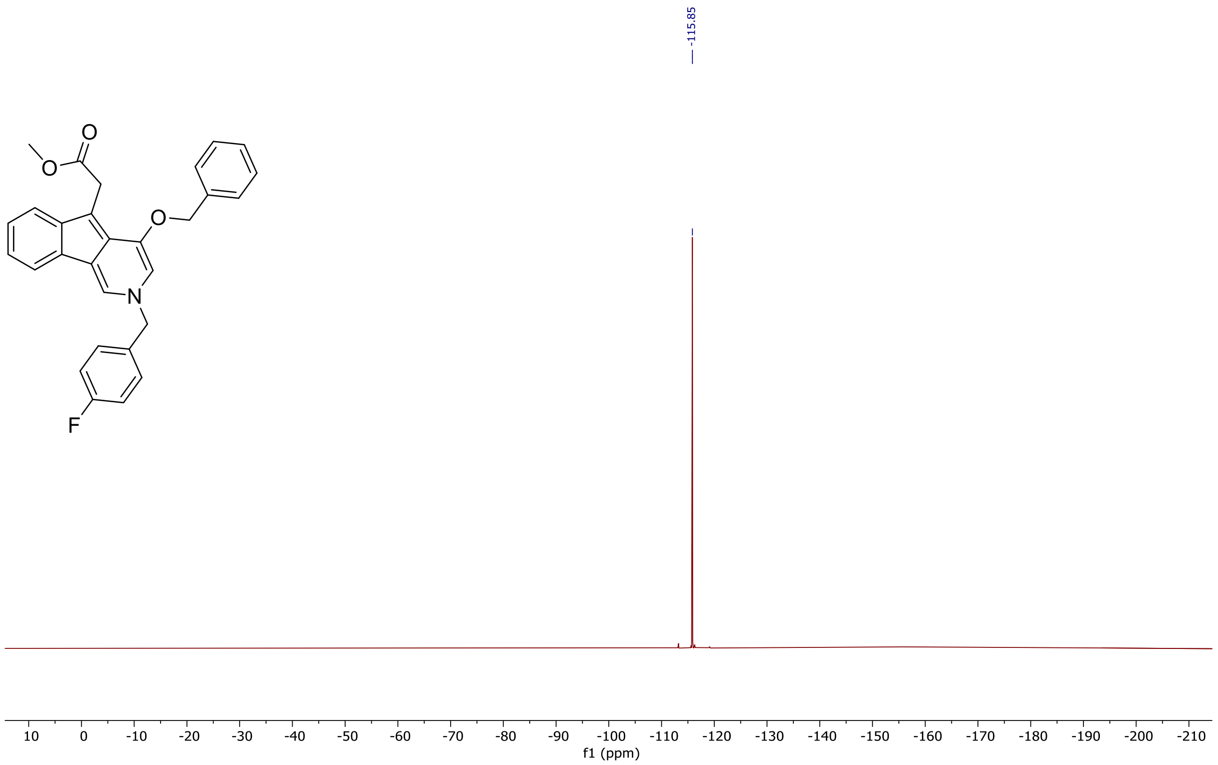

6t

20210427-1428-B500_B.14-1.10.fid
Ref 565-2
Group Greaney_M
H1_Night Acetone /mnt/nmrdata/Greaney_M m31962tw 1

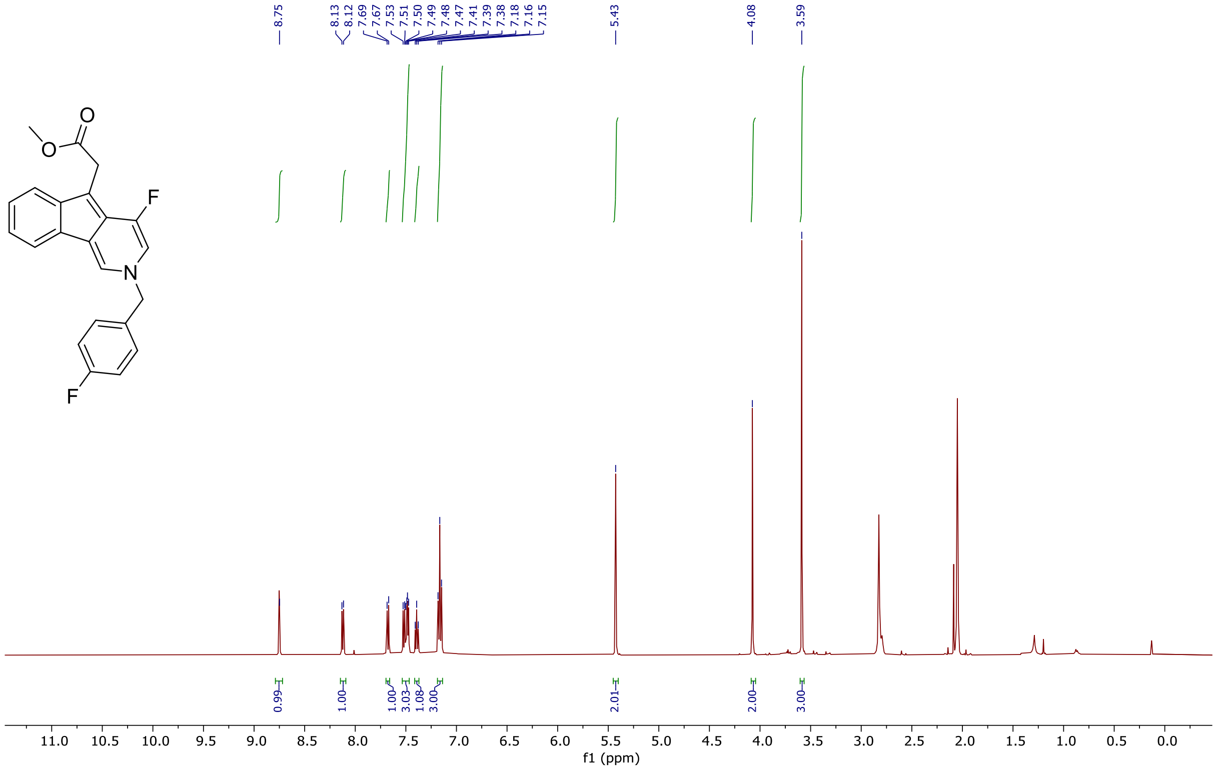

20210427-1428-B500_B.14-1.11.fid
Ref 565-2
Group Greaney_M
C13_CPD_Night2048 Acetone /mnt/nmrdata/Greaney_M m31962tw 1

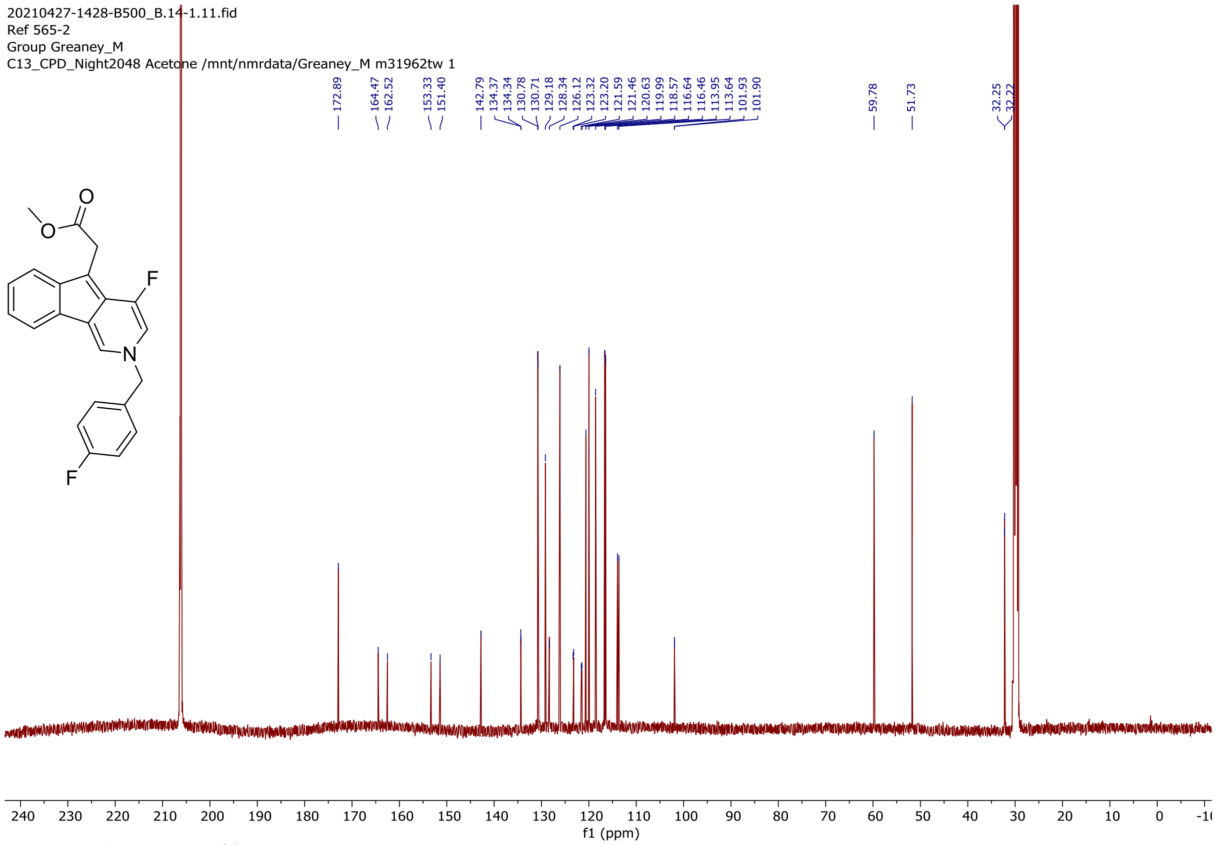

20210427-1428-B500_B.14-1.12.fid
Ref 565-2
Group Greaney_M
F19_NoCPD_Night Acetone /mnt/nmrdata/Greaney_M m31962tw 1

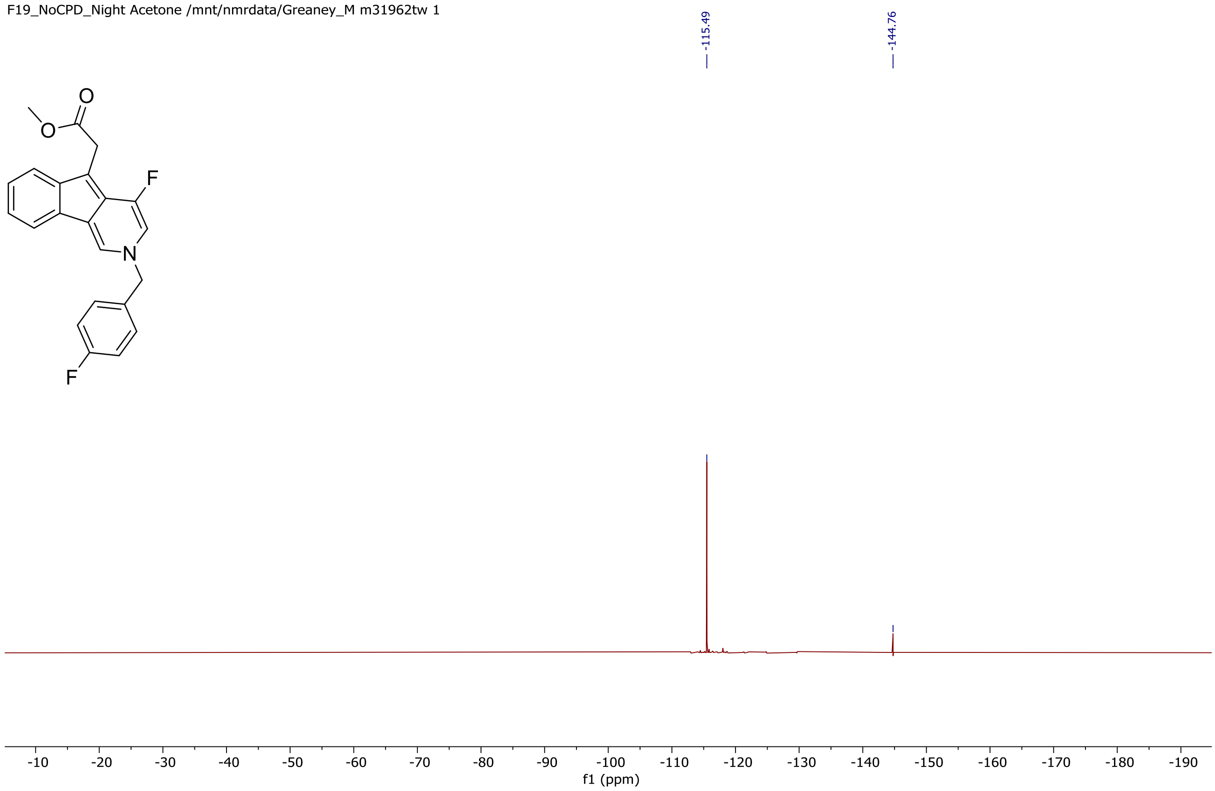

6u

20210302-1844-B400_B.12-20.10.fid
Ref 535-4
Group Greaney_M
H1_Night Acetone /mnt/nmrdata/Greaney_M m31962tw 20

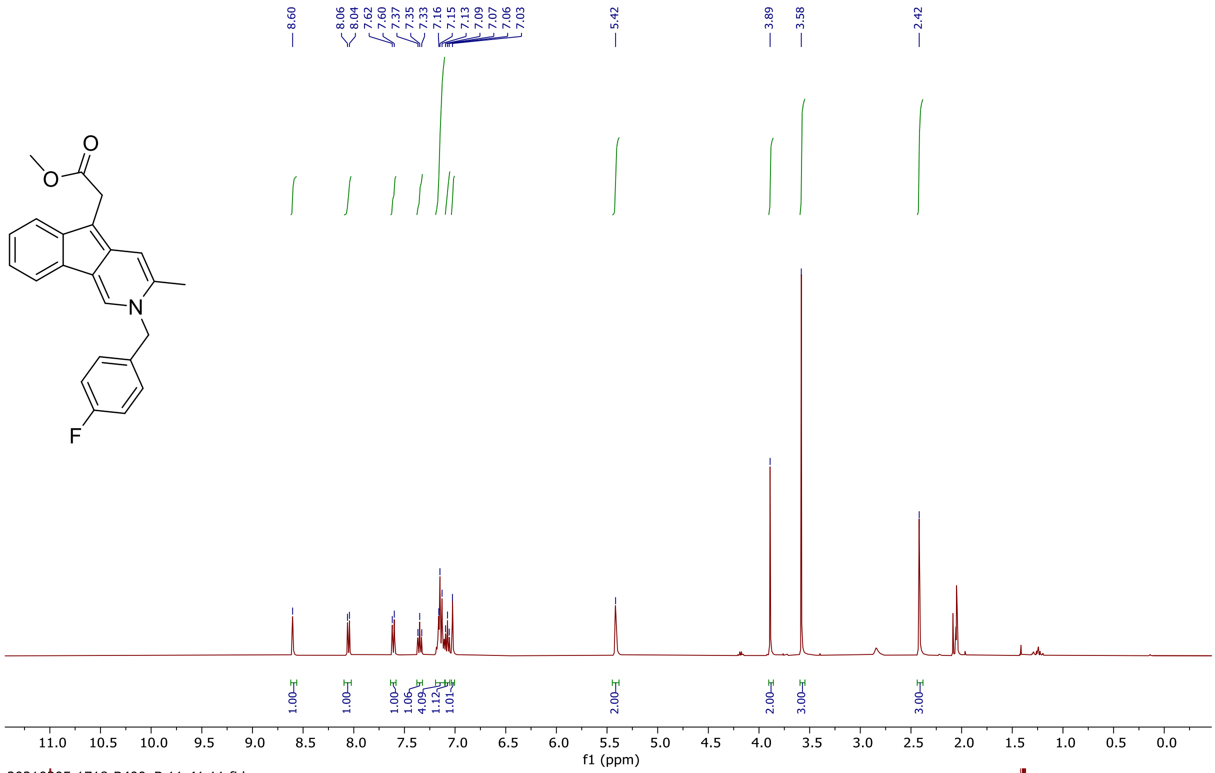

20210305-1718-B400_B.11-41.11.fid
Ref 535-4
Group Greaney_M
C13_CPD_Night1024 Acetone /mnt/nmrdata/Greaney_M m31962tw 41

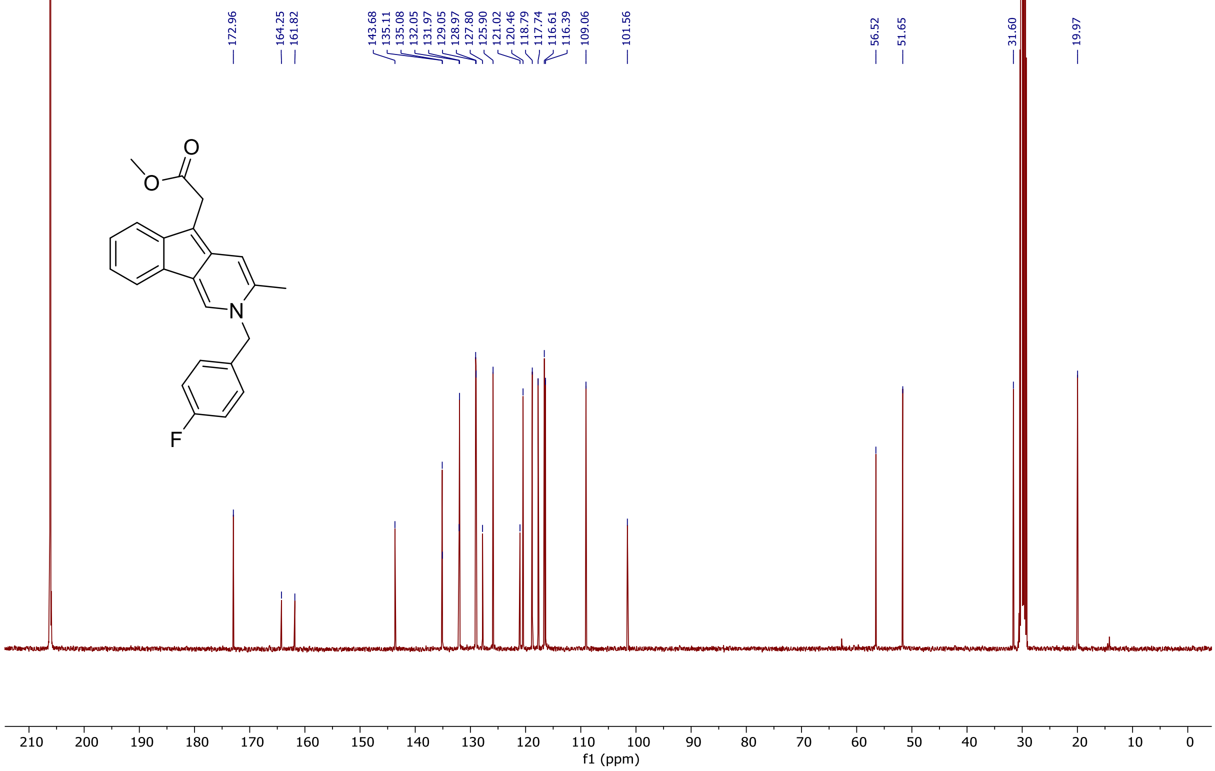

20210305-1718-B400_B.11-41.12.fid
Ref 535-4
Group Greaney_M
F19_NoCPD_Night Acetone /mnt/nmrdata/Greaney_M m31962tw 41

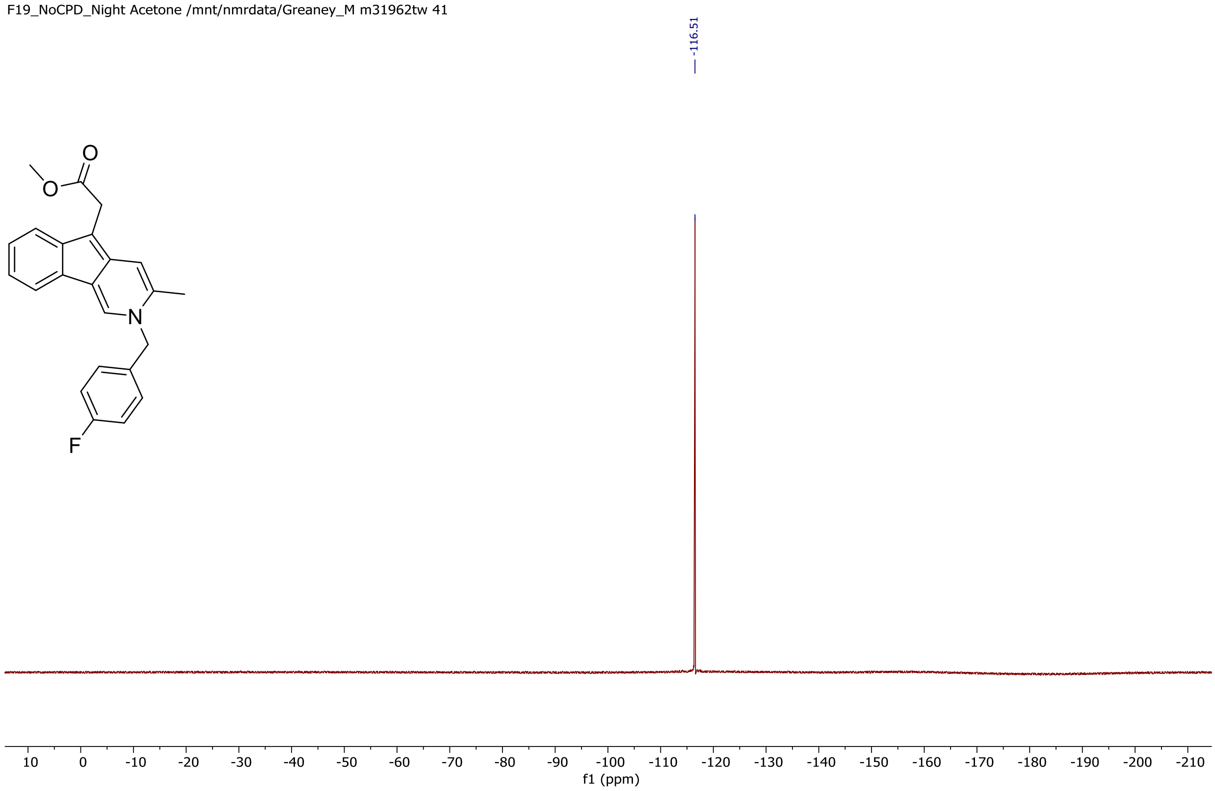

6v

20211130-1807-B500_B.14-22.10.fid
Ref 758-4
Group Greaney_M
H1_Night Acetone /mnt/nmrdata/Greaney_M m31962tw 22

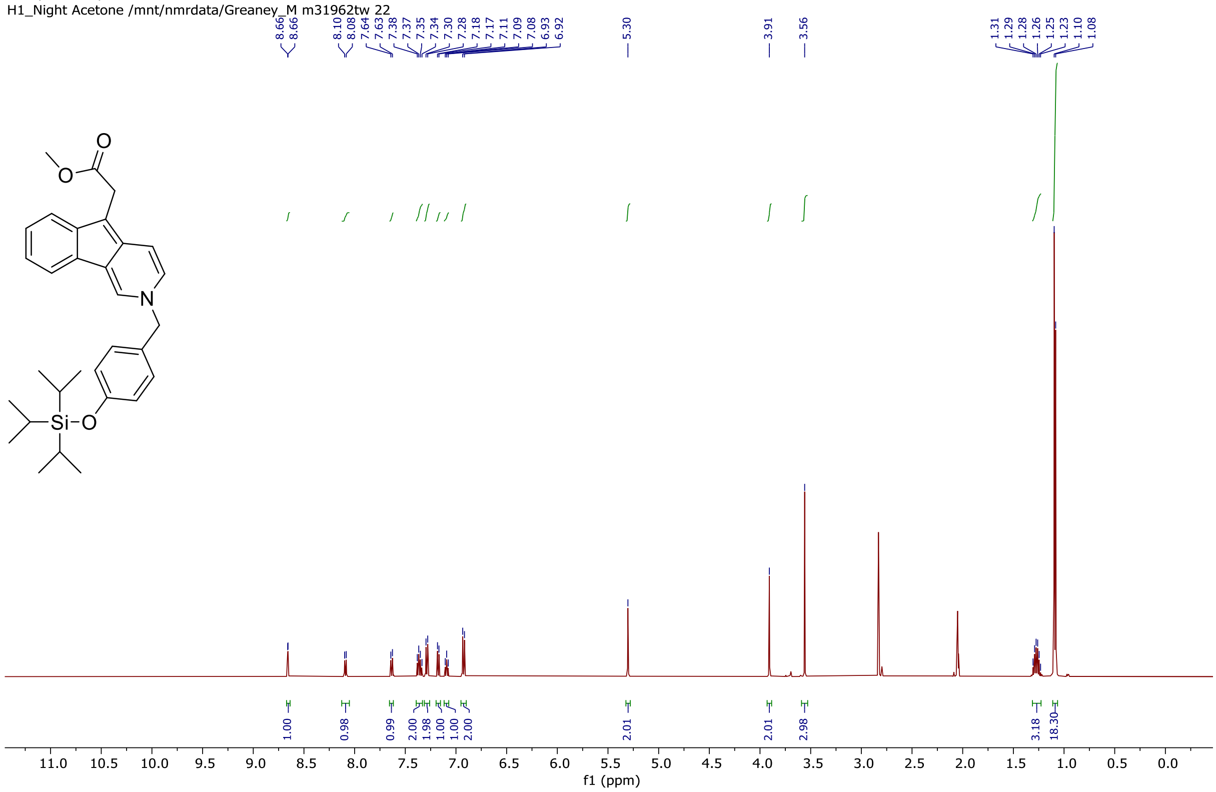

20211130-1807-B500_B.14-22.11.fid
Ref 758-4
Group Greaney_M
C13_CPD_Night256 Acetone /mnt/nmrdata/Greaney_M m31962tw 22

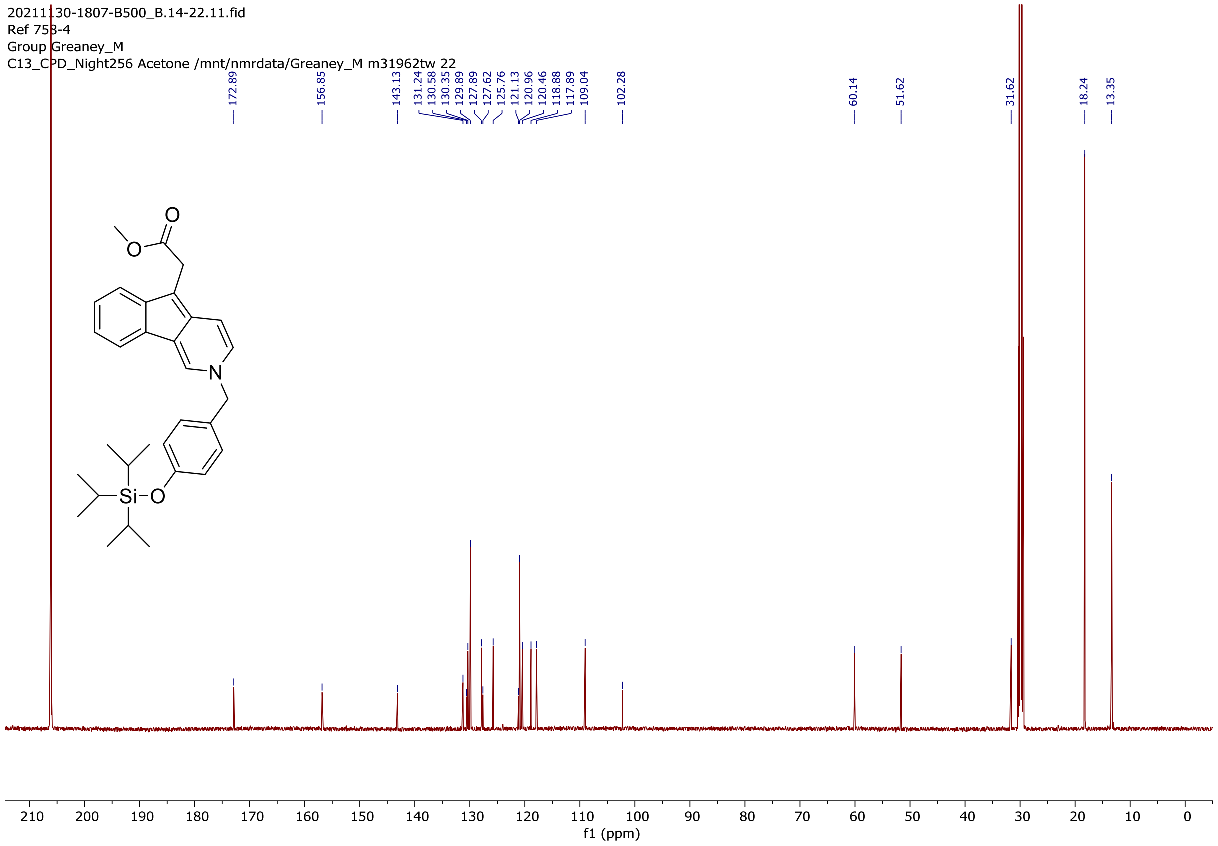

12a

20210617-1419-B400_B.11-22.10.fid
Ref 605-1
Group Greaney_M
H1_Night Acetone /mnt/nmrdata/Greaney_M m31962tw 22

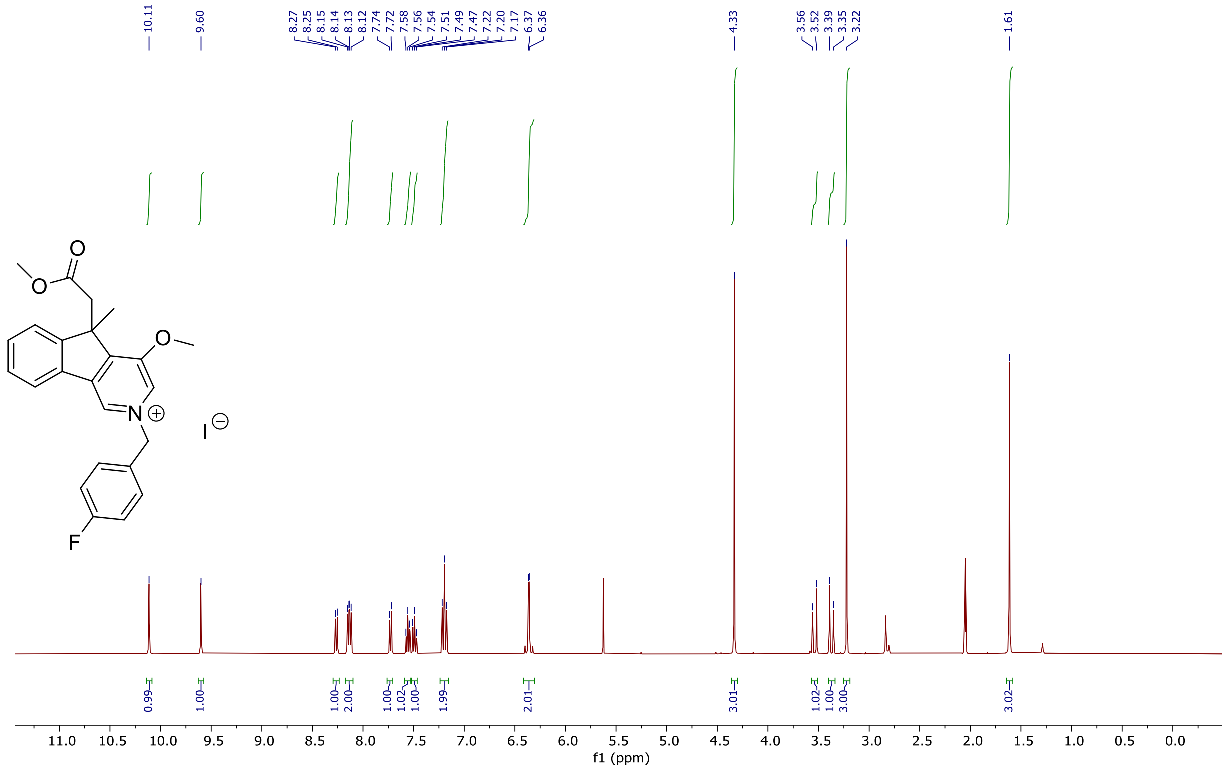

20210617-1419-B400_B.11-22.11.fid
Ref 605-1
Group Greaney_M
C13_CPD_Night256 Acetone /mnt/nmrdata/Greaney_M m31962tw 22

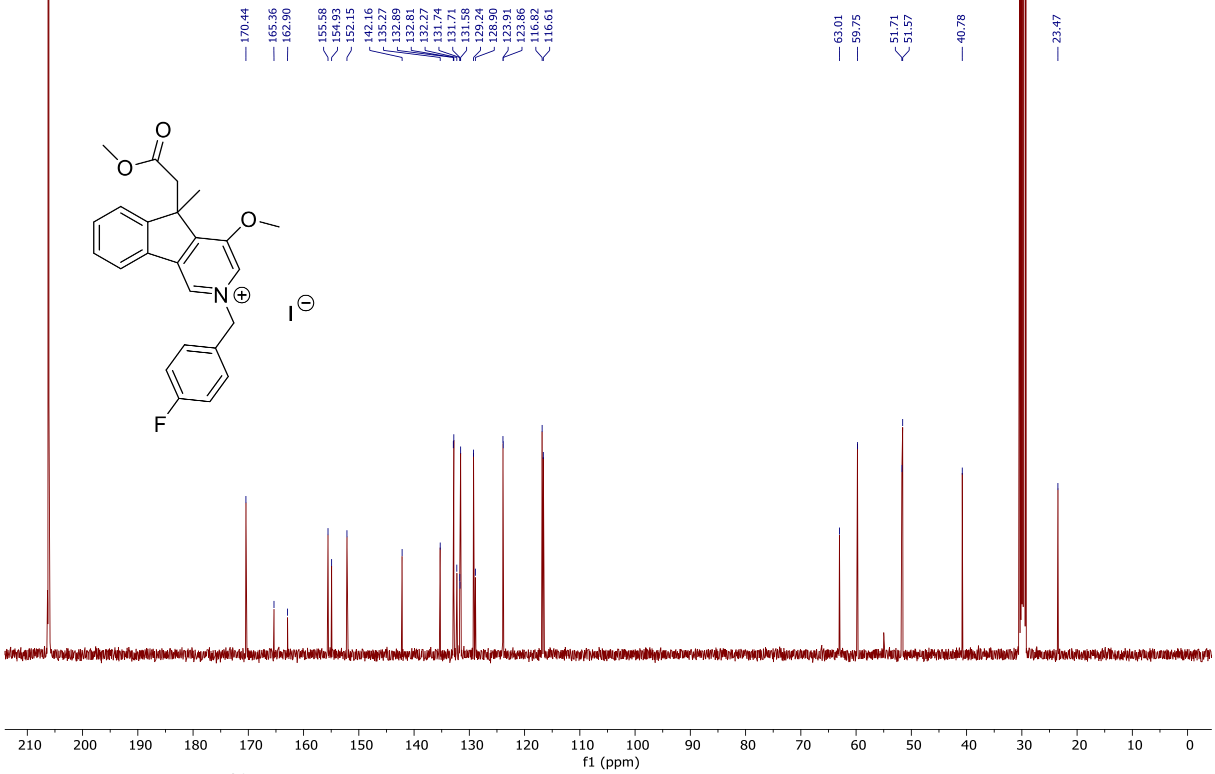

20210617-1419-B400_B.11-22.15.fid
Ref 605-1
Group Greaney_M
F19_NoCPD_Day Acetone /mnt/nmrdata/Greaney_M m31962tw 22

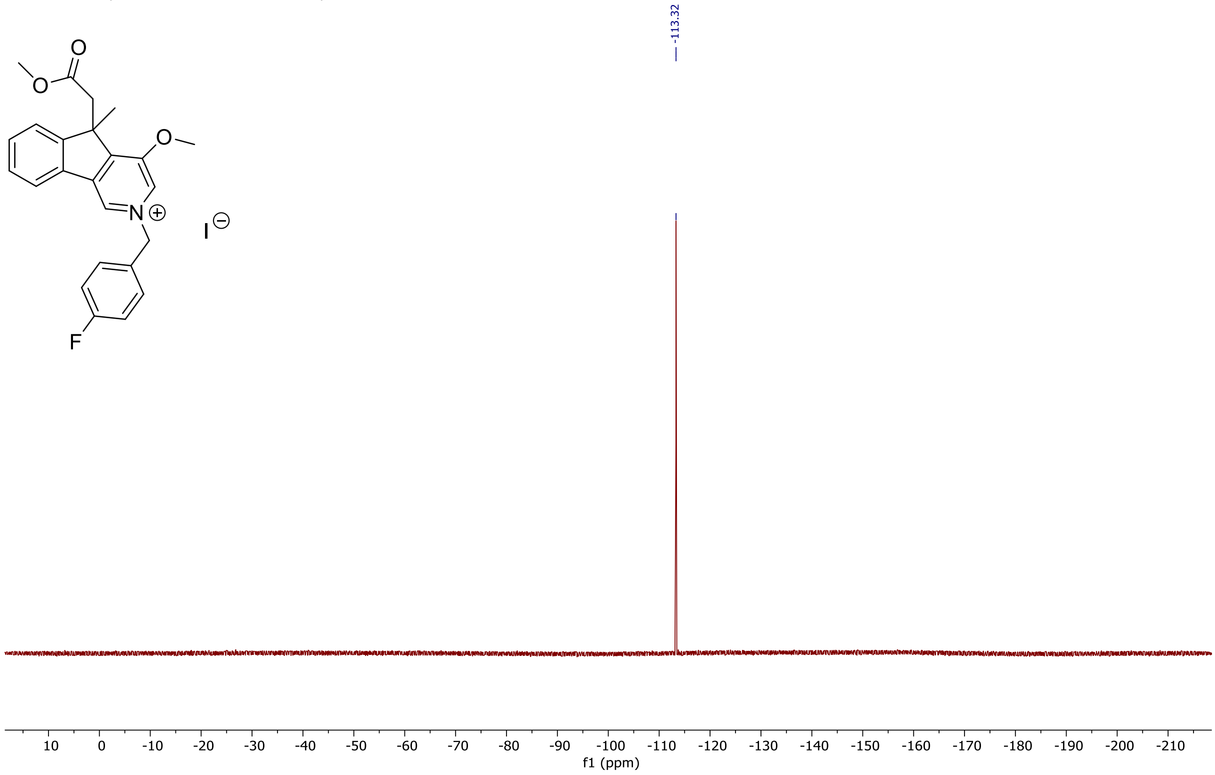

12b

20210622-1908-B400_B.11-21.10.fid
Ref 614-3
Group Greaney_M
H1_Night MeOD /mnt/nmrdata/Greaney_M m31962tw 21

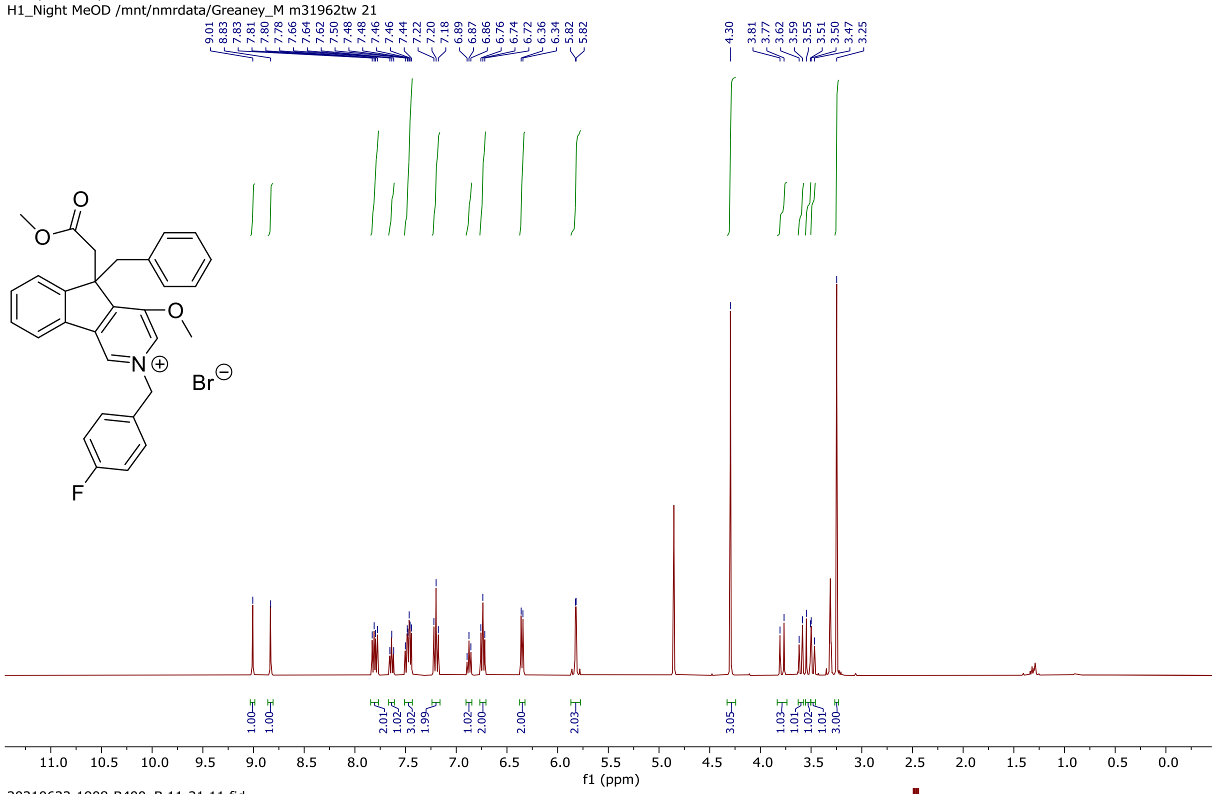

20210622-1908-B400_B.11-21.11.fid
Ref 614-3
Group Greaney_M
C13_CPD_Night256 MeOD /mnt/nmrdata/Greaney_M m31962tw 21

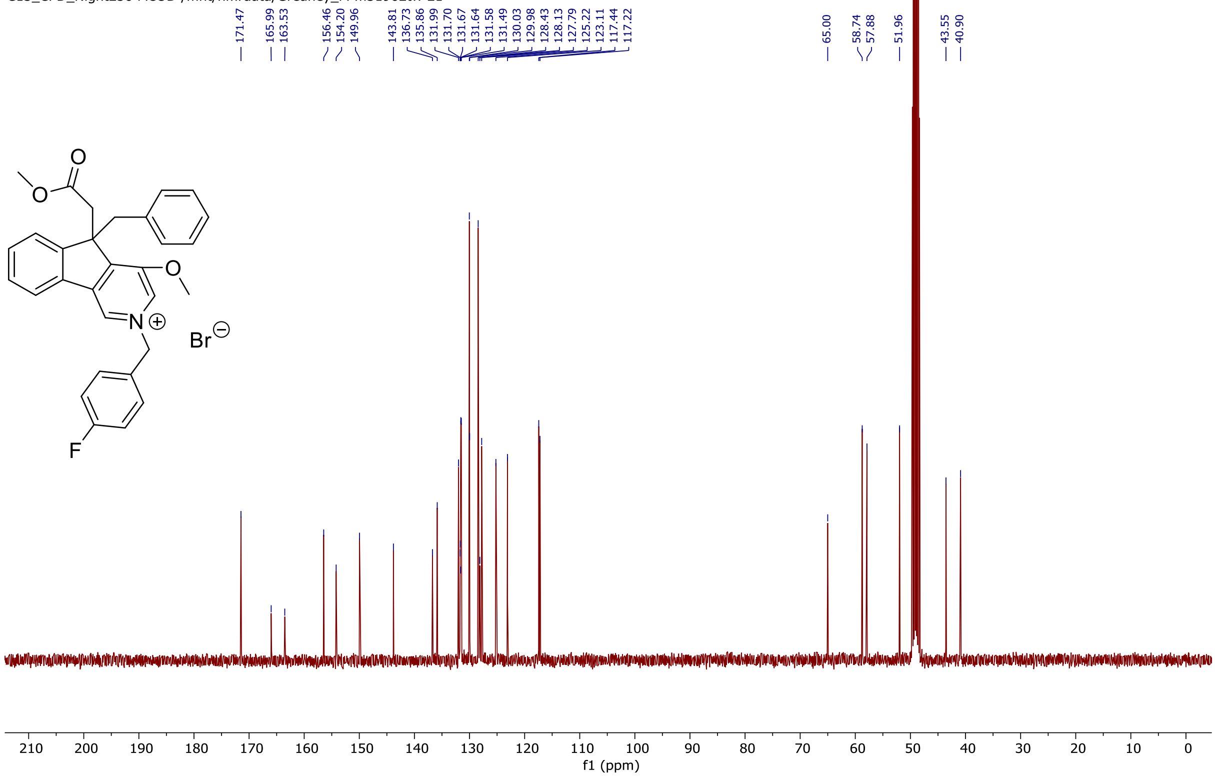

20210622-1908-B400_B.11-21.15.fid
Ref 614-3
Group Greaney_M
F19_NoCPD_Night MeOD /mnt/nmrdata/Greaney_M m31962tw 21

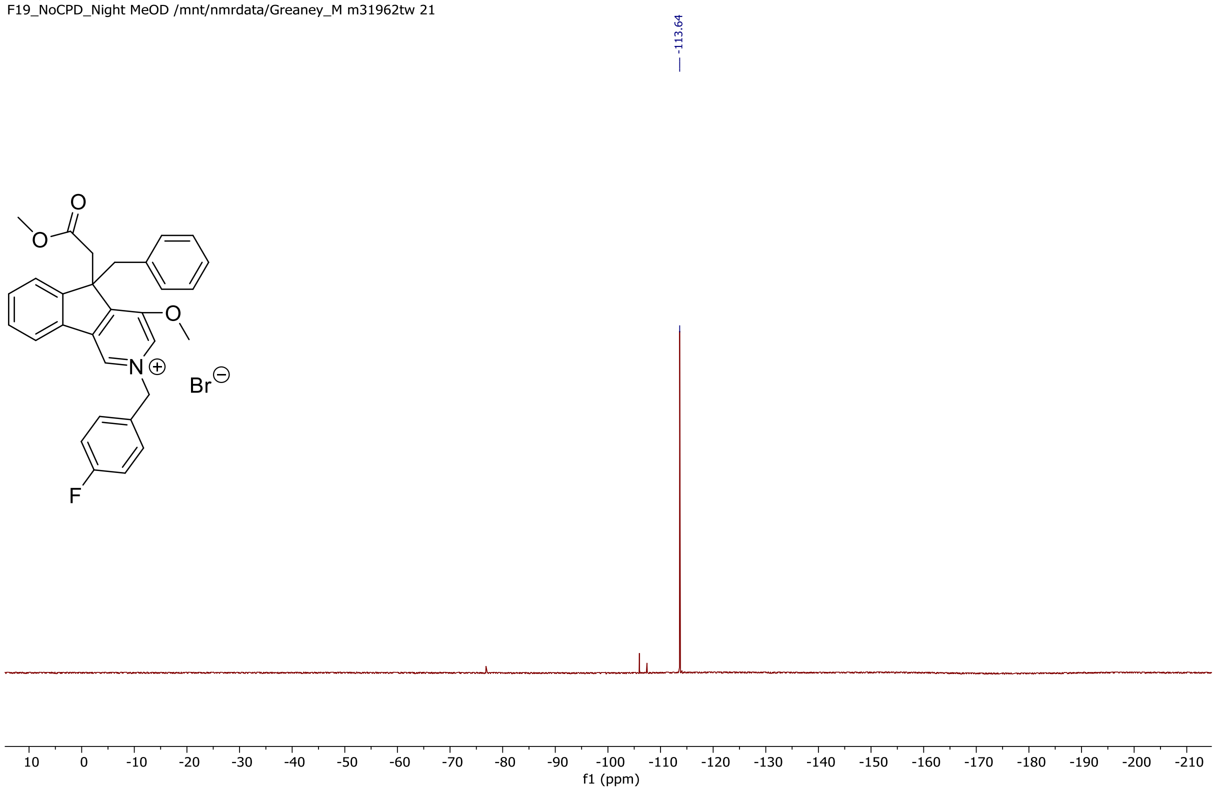

12c

20210622-1908-B400_B.11-22.10.fid
Ref 614-4
Group Greaney_M
H1_Night MeOD /mnt/nmrdata/Greaney_M m31962tw 22

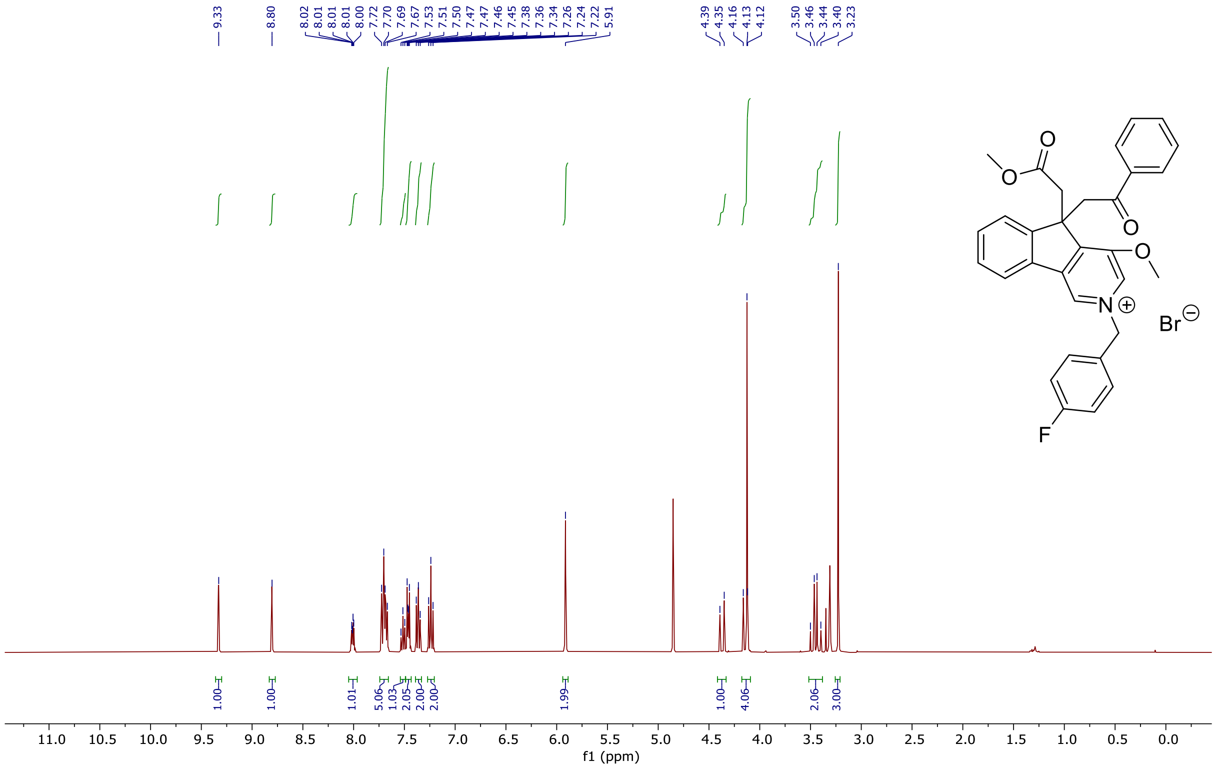

20210622-1908-B400_B.11-22.11.fid
Ref 614-4
Group Greaney_M
C13_CPD_Night256 MeOD /mnt/nmrdata/Greaney_M m31962tw 22

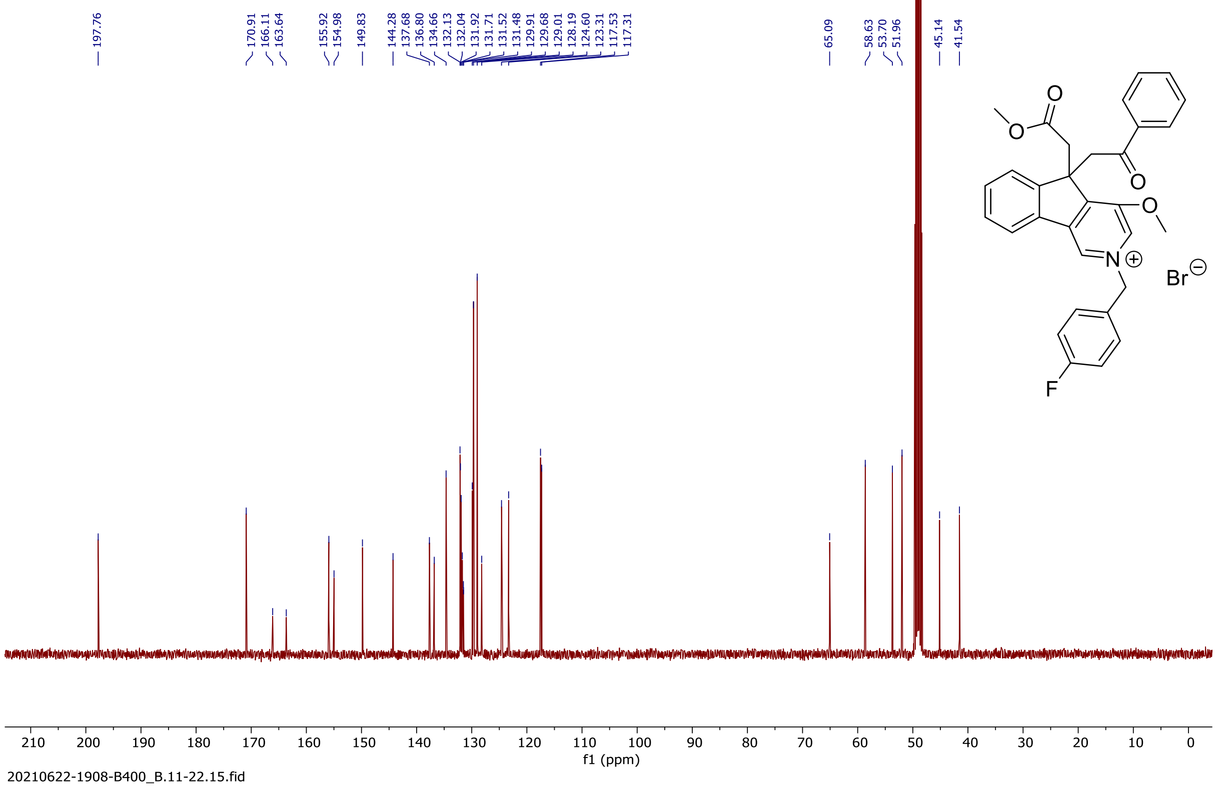

20210622-1908-B400_B.11-22.15.fid
Ref 614-4
Group Greaney_M
F19_NoCPD_Night MeOD /mnt/nmrdata/Greaney_M m31962tw 22

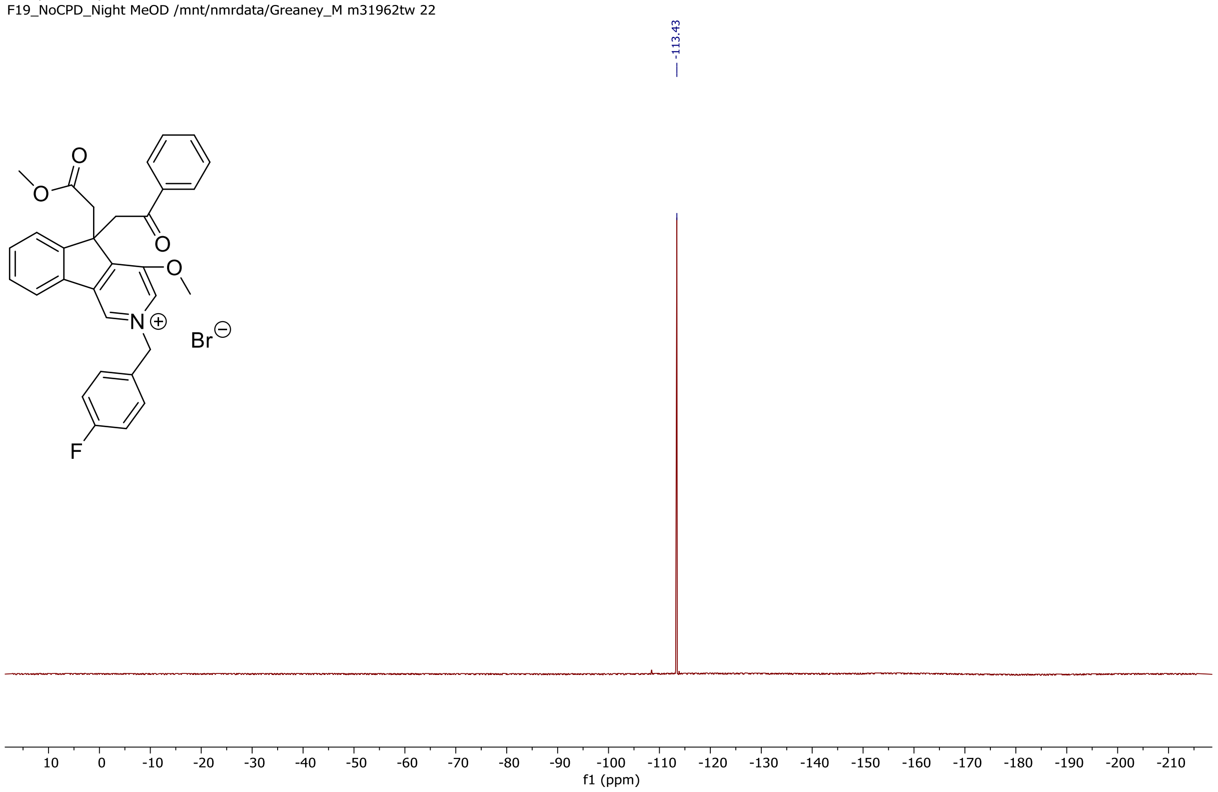

12d

20211126-1657-B400_B.11-20.10.fid
Ref 752-5
Group Greaney_M
H1_Night Acetone /mnt/nmrdata/Greaney_M m31962tw 20

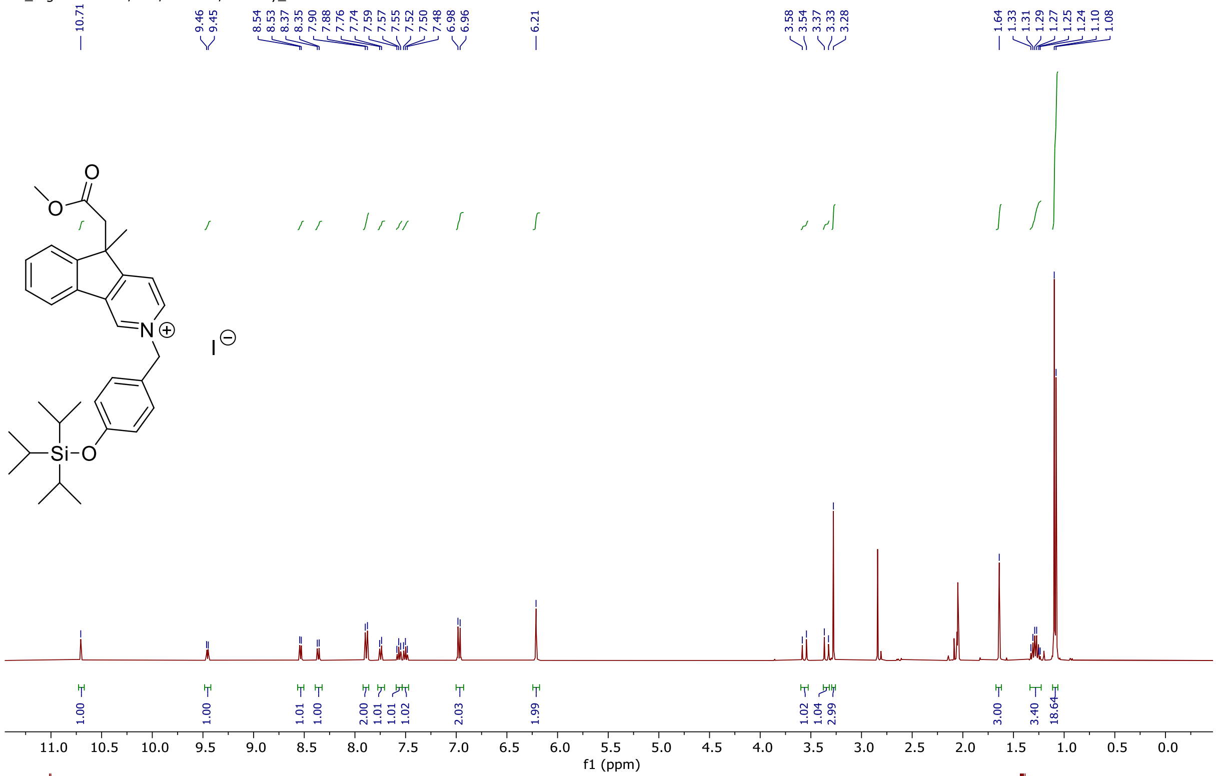

20211126-1657-B400_B.11-20.11.fid
Ref 752-5
Group Greaney_M
C13_CPD_Night256 Acetone /mnt/nmrdata/Greaney_M m31962tw 20

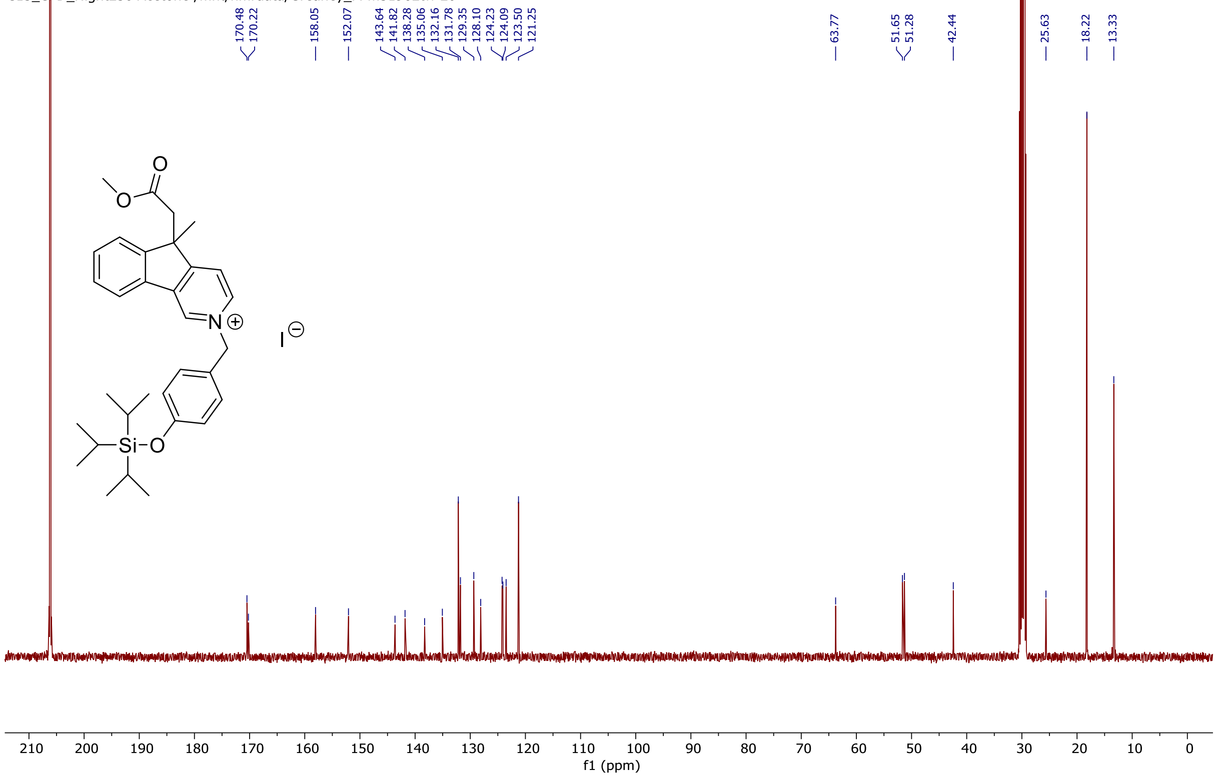

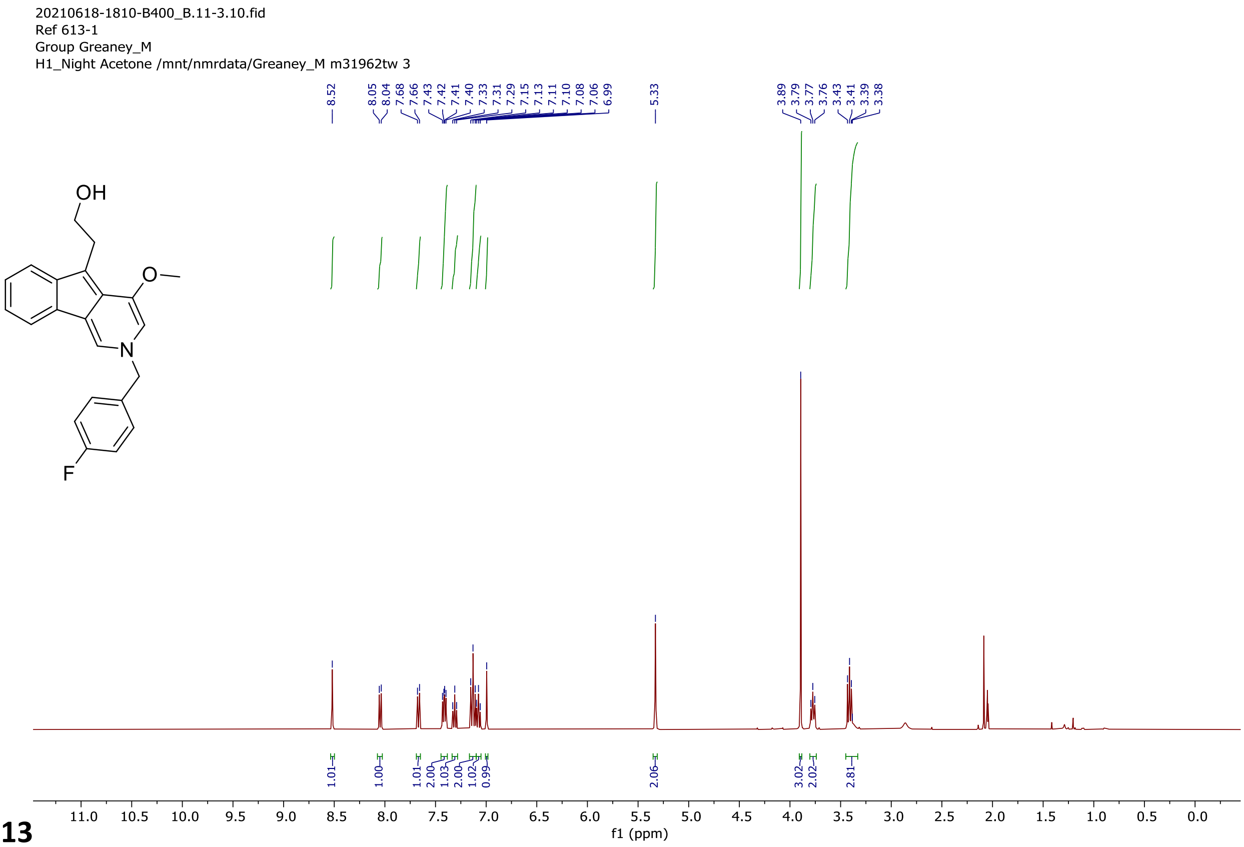
20210618-1810-B400_B.11-3.10.fid
Ref 613-1
Group Greaney_M
H1_Night Acetone /mnt/nmrdata/Greaney_M m31962tw 3
8.52
8.05
8.04
7.68
7.66
7.43
7.42
7.41
7.40
7.33
7.31
7.29
7.15
7.13
7.11
7.10
7.08
7.06
6.99
5.33
3.89
3.79
3.77
3.76
3.43
3.41
3.39
3.38
OH
O
N
F
1.01
1.00
1.01
2.00
1.03
2.00
1.02
0.99
2.06
3.02
2.02
2.81
f1 (ppm)

13

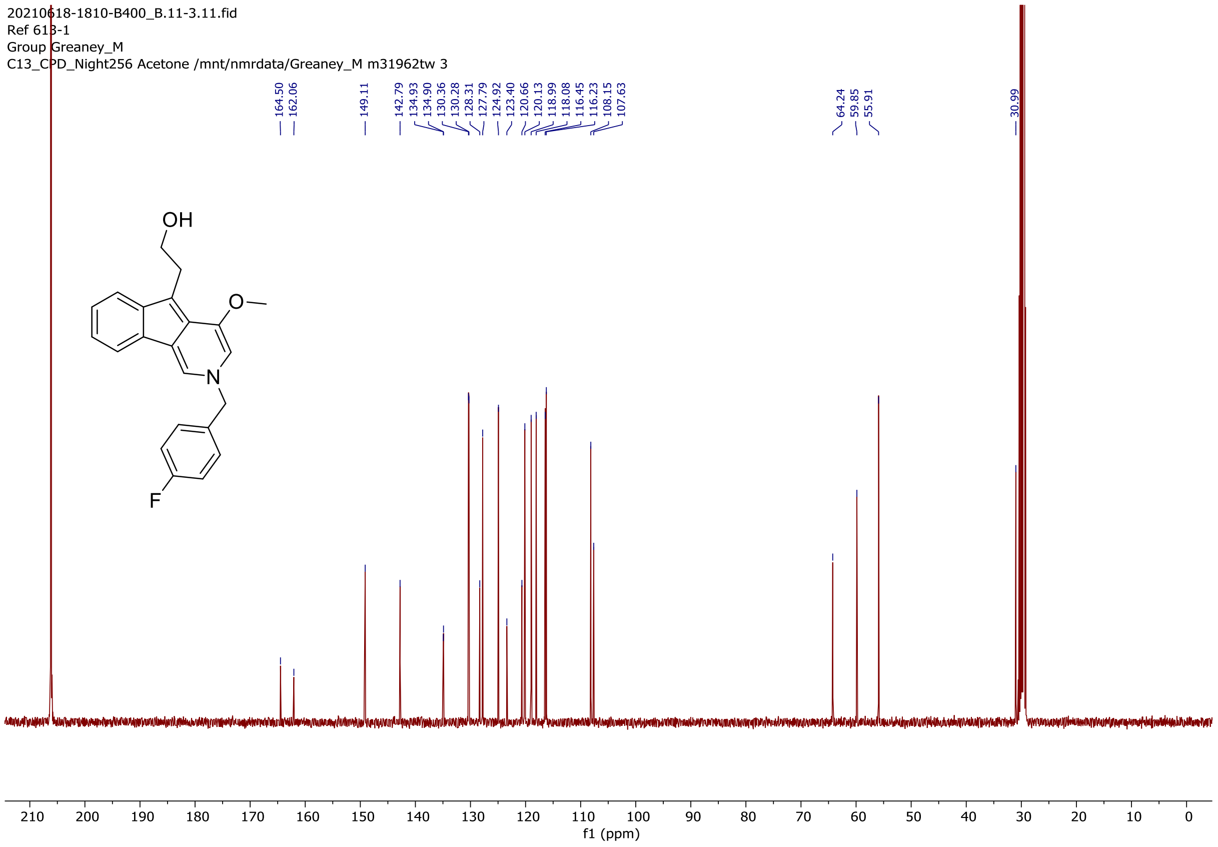
20210618-1810-B400_B.11-3.11.fid
Ref 613-1
Group Greaney_M
C13_CPD_Night256 Acetone /mnt/nmrdata/Greaney_M m31962tw 3
164.50
162.06
149.11
142.79
134.93
134.90
130.36
130.28
128.31
127.79
124.92
123.40
120.66
120.13
118.99
118.08
116.45
116.23
108.15
107.63
64.24
59.85
55.91
30.99
OH
O
N
F
f1 (ppm)

20210618-1810-B400_B.11-3.15.fid
Ref 613-1
Group Greaney_M
F19_NoCPD_Night Acetone /mnt/nmrdata/Greaney_M m31962tw 3

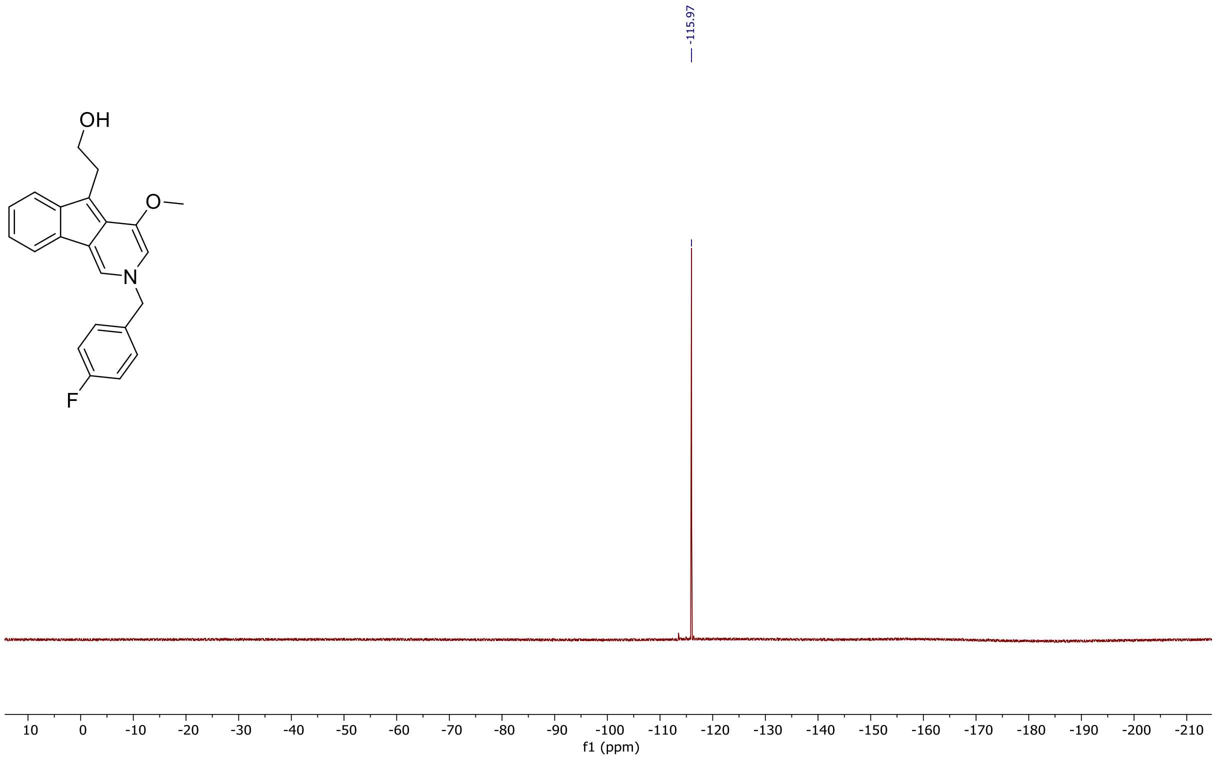

14

20211202-1743-B400_B.11-23.10.fid
Ref 752-4
Group Greaney_M
H1_Night CDCl3 /mnt/nmrdata/Greaney_M m31962tw 23

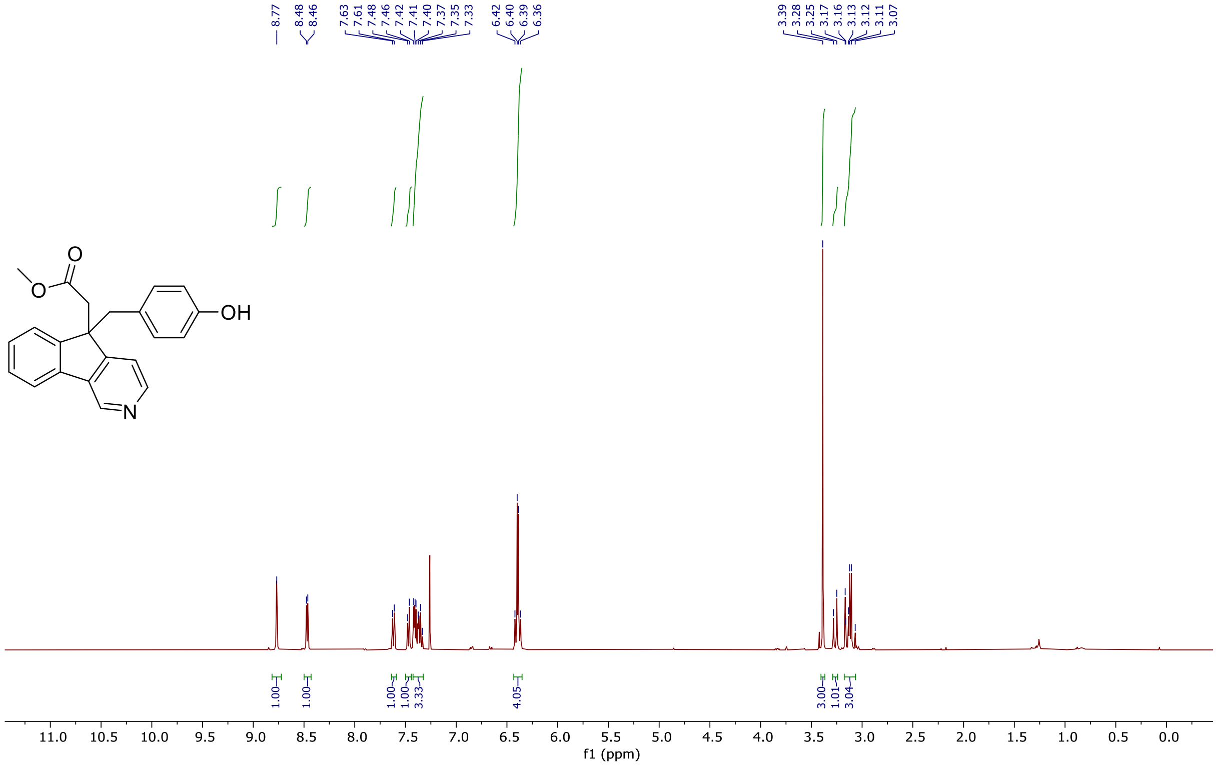

20211202-1743-B400_B.11-23.11.fid
Ref 752-4
Group Greaney_M
C13_CPD_Night256 CDCl3 /mnt/nmrdata/Greaney_M m31962tw 23

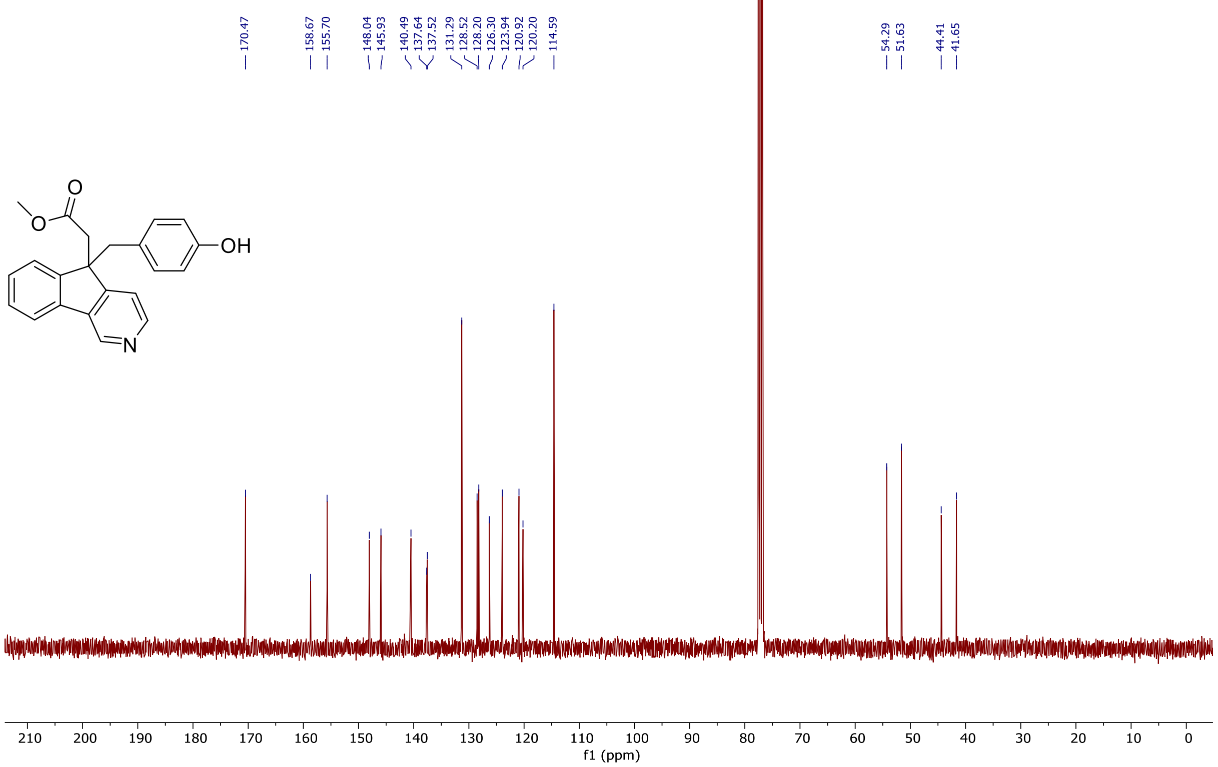

15

20211201-1735-B500_B.14-17.10.fid
Ref 760-2
Group Greaney_M
H1_Night CDCl3 /mnt/nmrdata/Greaney_M m31962tw 17

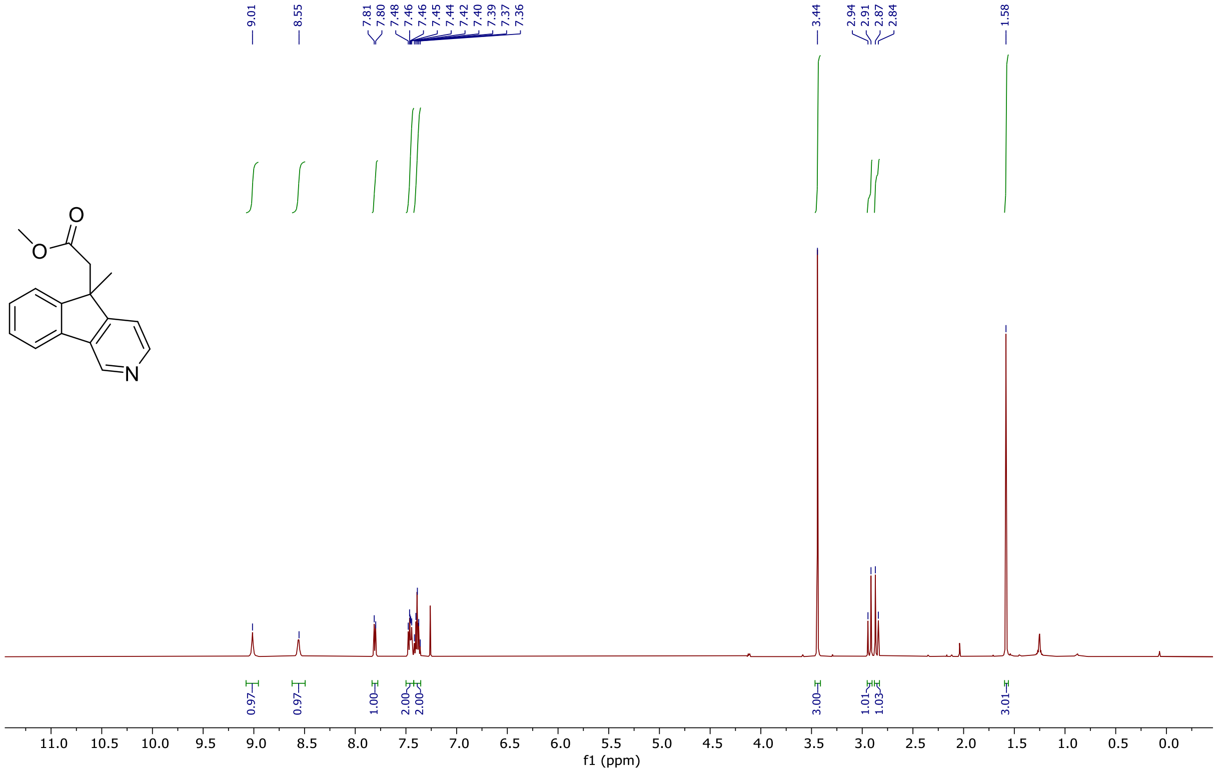

20211201-1735-B500_B.14-17.11.fid
Ref 760-2
Group Greaney_M
C13_CPD_Night256 CDCl3 /mnt/nmrdata/Greaney_M m31962tw 17

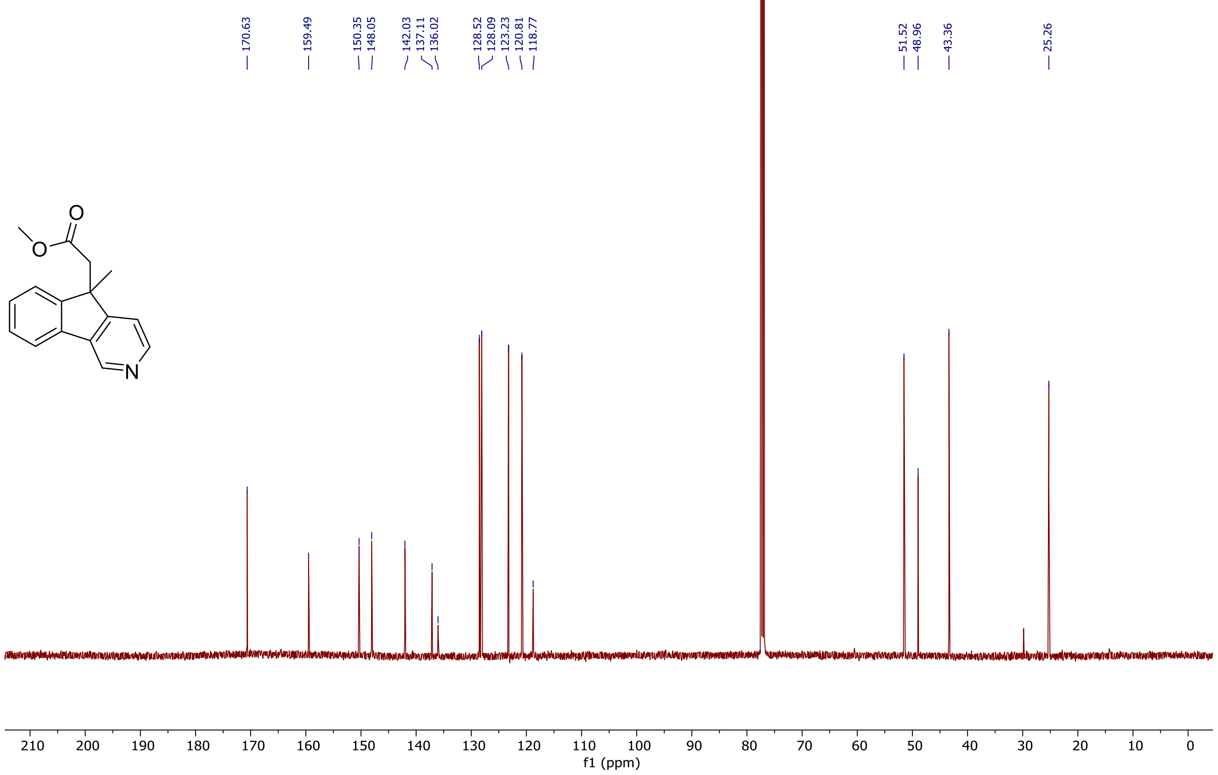

18

20210809-1520-B400_B.11-8.10.fid
Ref 658-16
Group Greaney_M
H1_Night Acetone /mnt/nmrdata/Greaney_M m31962tw 8

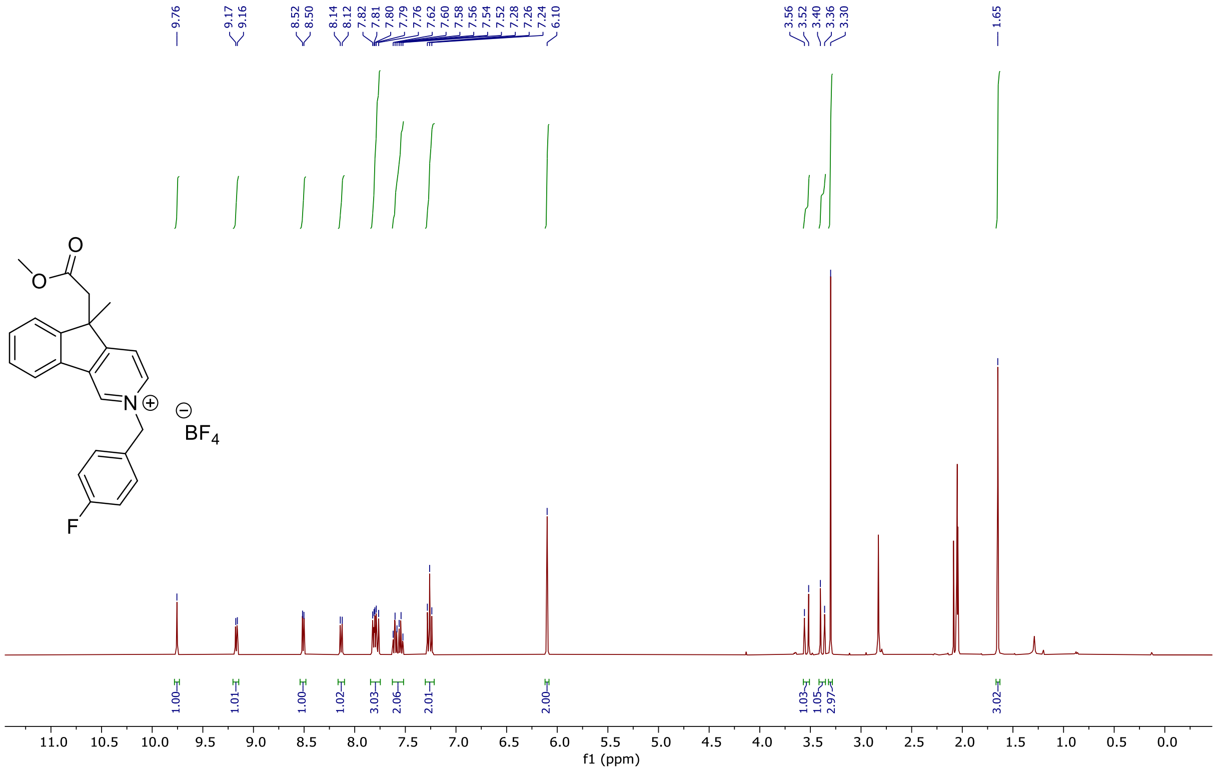

20210810-1432-B400_B.11-46.10.fid
Ref 658-16
Group Greaney_M
C13_CPD_Night1024 Acetone /mnt/nmrdata/Greaney_M m31962tw 46

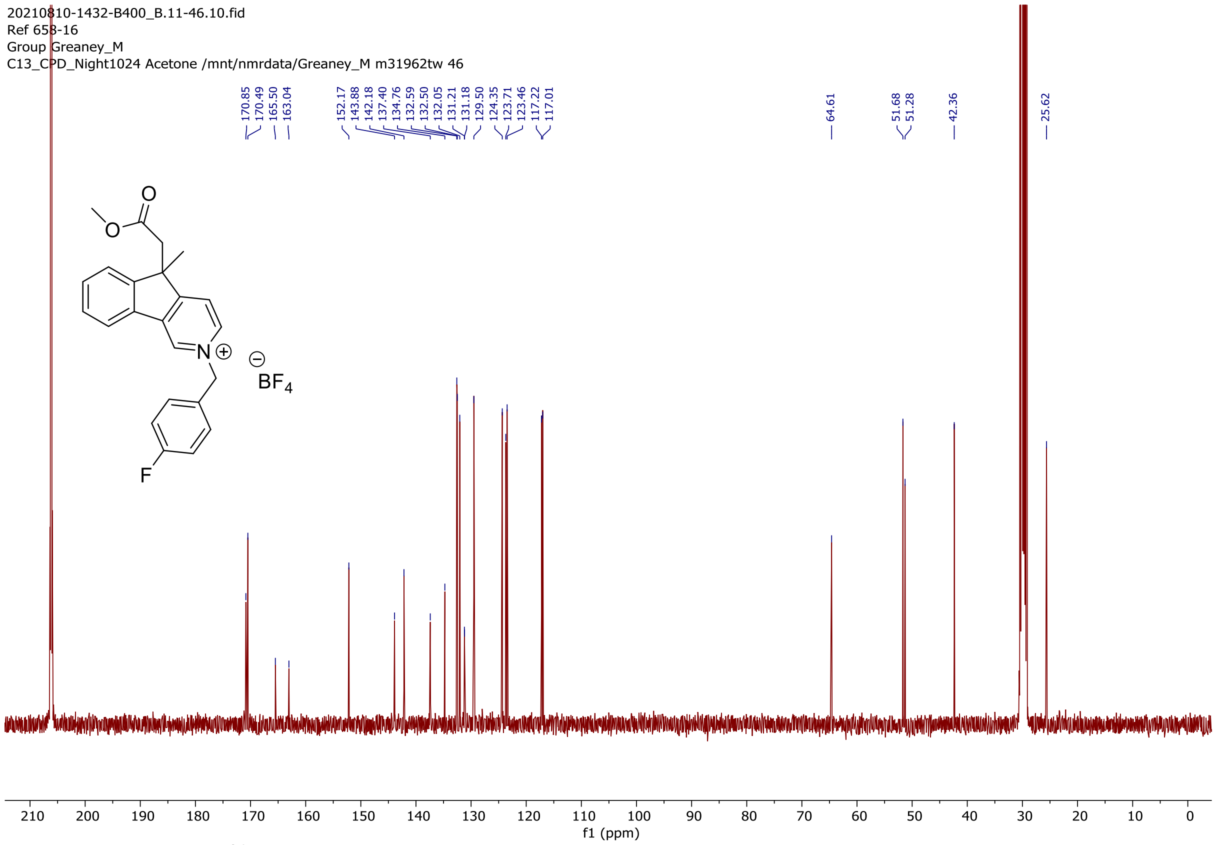

20210819-1534-B500_B.14-33.10.fid
Ref 658-16
Group Greaney_M
F19_NoCPD_Day Acetone /mnt/nmrdata/Greaney_M m31962tw 33

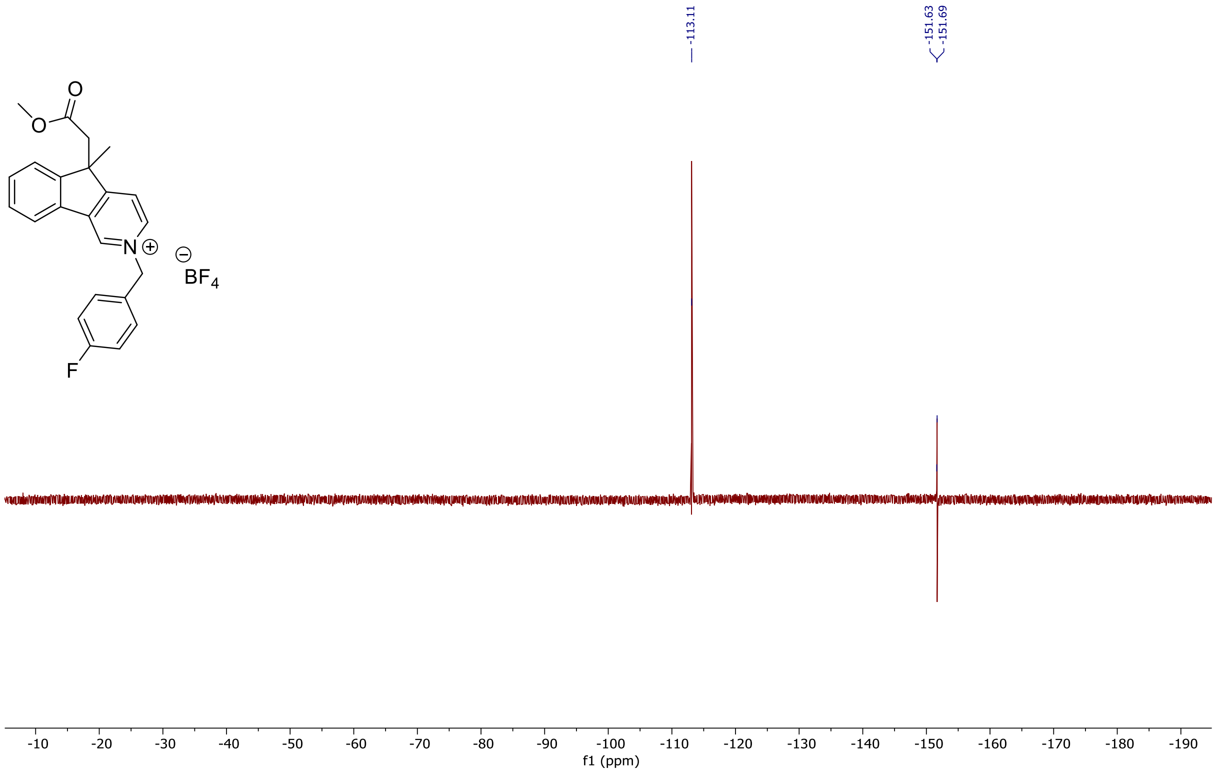

20210810-1425-B400_B.11-34.10.fid
Ref 639-1
Group Greaney_M
H1_Night MeOD /mnt/nmrdata/Greaney_M m31962tw 34

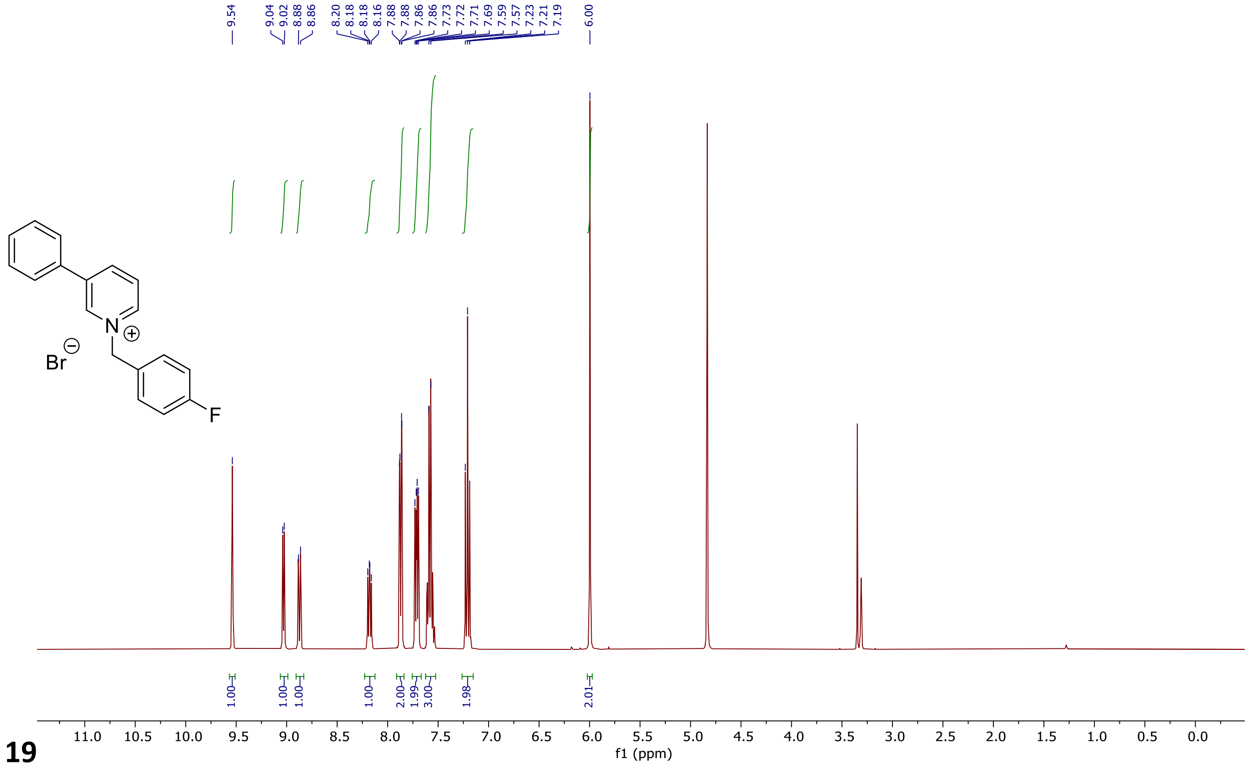

19

20210810-1425-B400_B.11-34.11.fid
Ref 639-1
Group Greaney_M
C13_CPD_Night256 MeOD /mnt/nmrdata/Greaney_M m31962tw 34

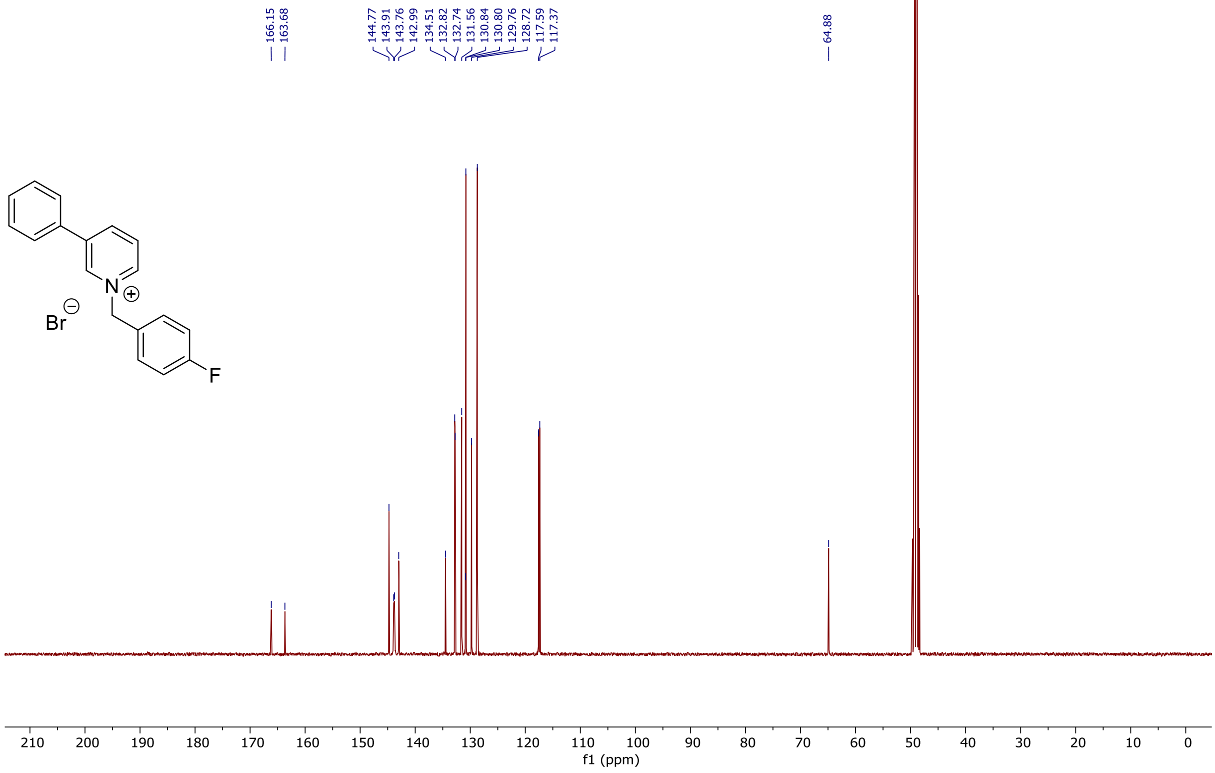

20210810-1425-B400_B.11-34.15.fid
Ref 639-1
Group Greaney_M
F19_NoCPD_Night MeOD /mnt/nmrdata/Greaney_M m31962tw 34

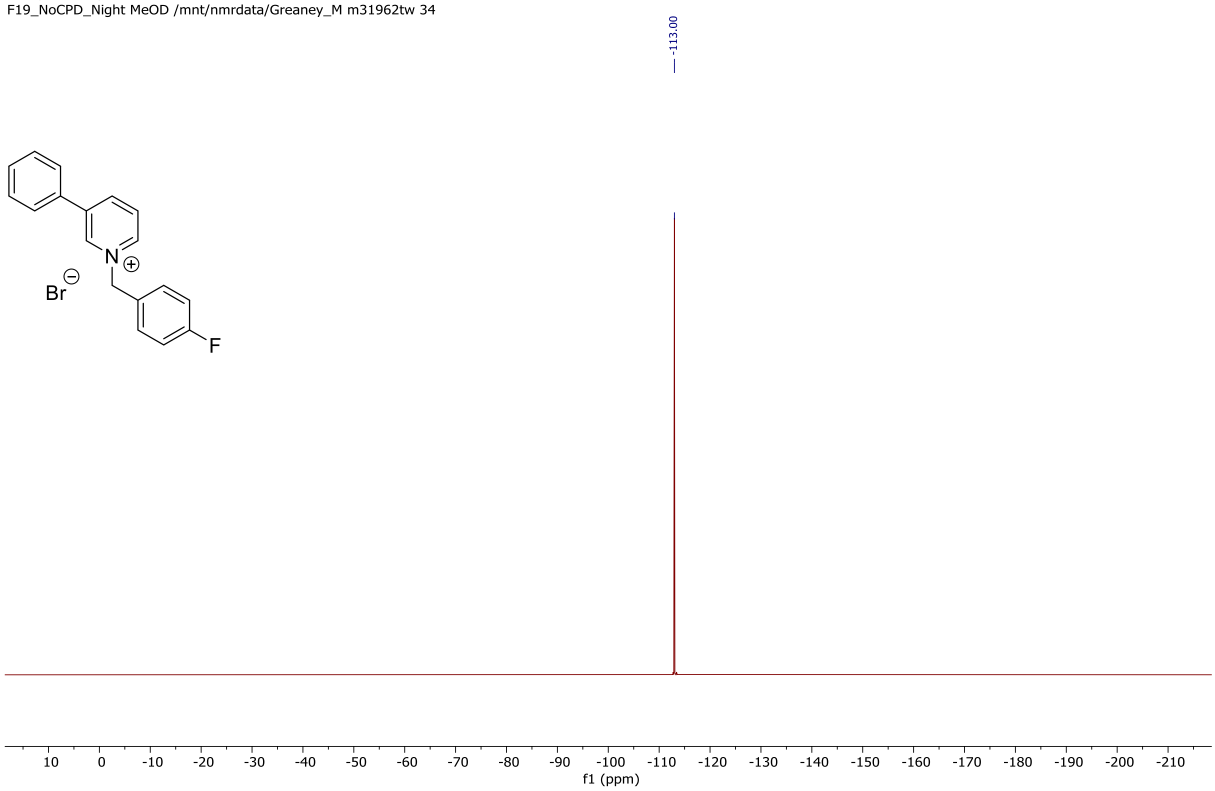

23

20210427-1017-B400_B.11-30.10.fid
Ref 519-1
Group Greaney_M
H1_Night CDCl3 /mnt/nmrdata/Greaney_M m31962tw 30

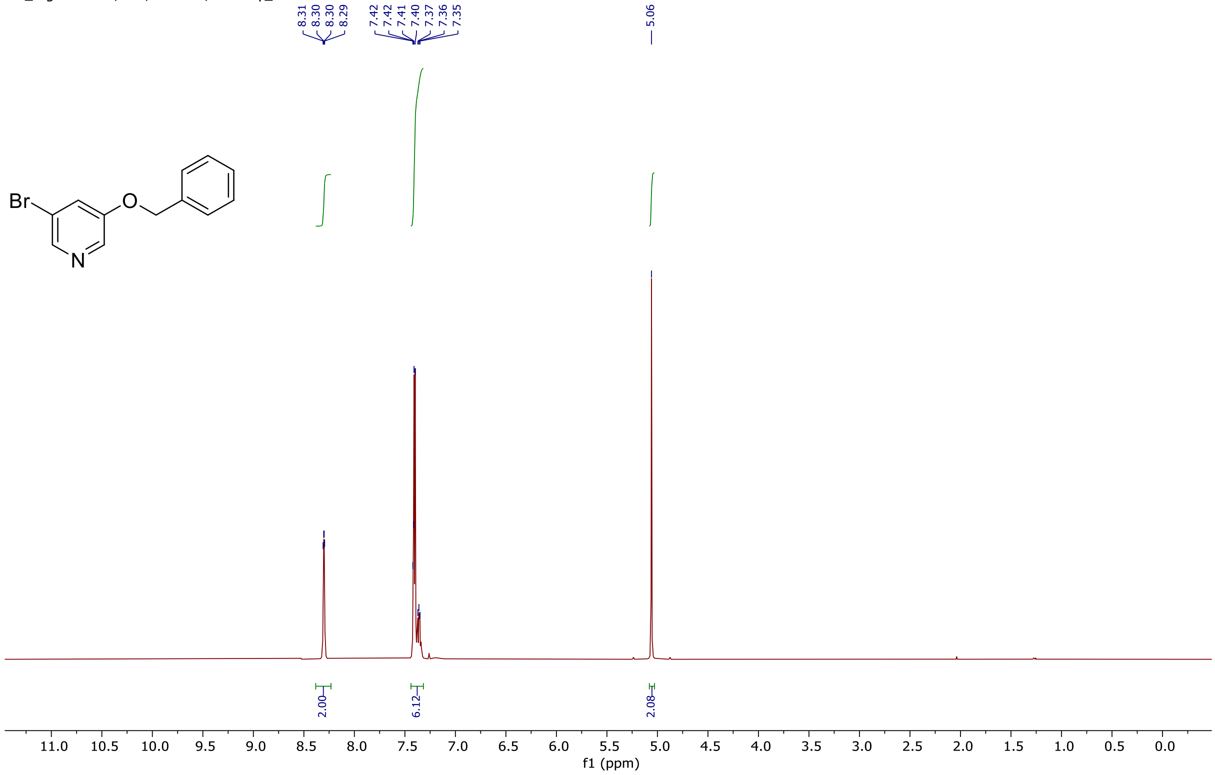

20210427-1017-B400_B.11-30.11.fid
Ref 519-1
Group Greaney_M
C13_CPD_Night256 CDCl3 /mnt/nmrdata/Greaney_M m31962tw 30

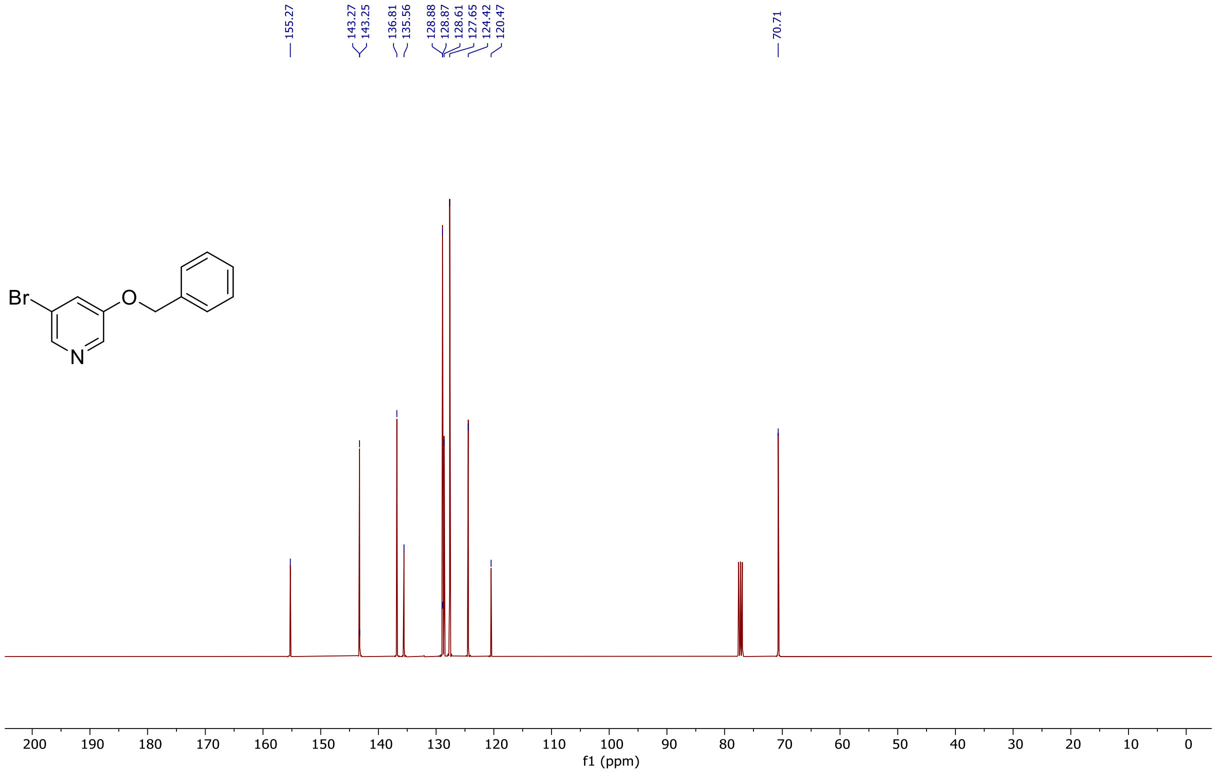

24

20210715-1541-B400_B.11-18.10.fid
Ref 637-1
Group Greaney_M
H1_Day CDCl3 /mnt/nmrdata/Greaney_M m31962tw 18

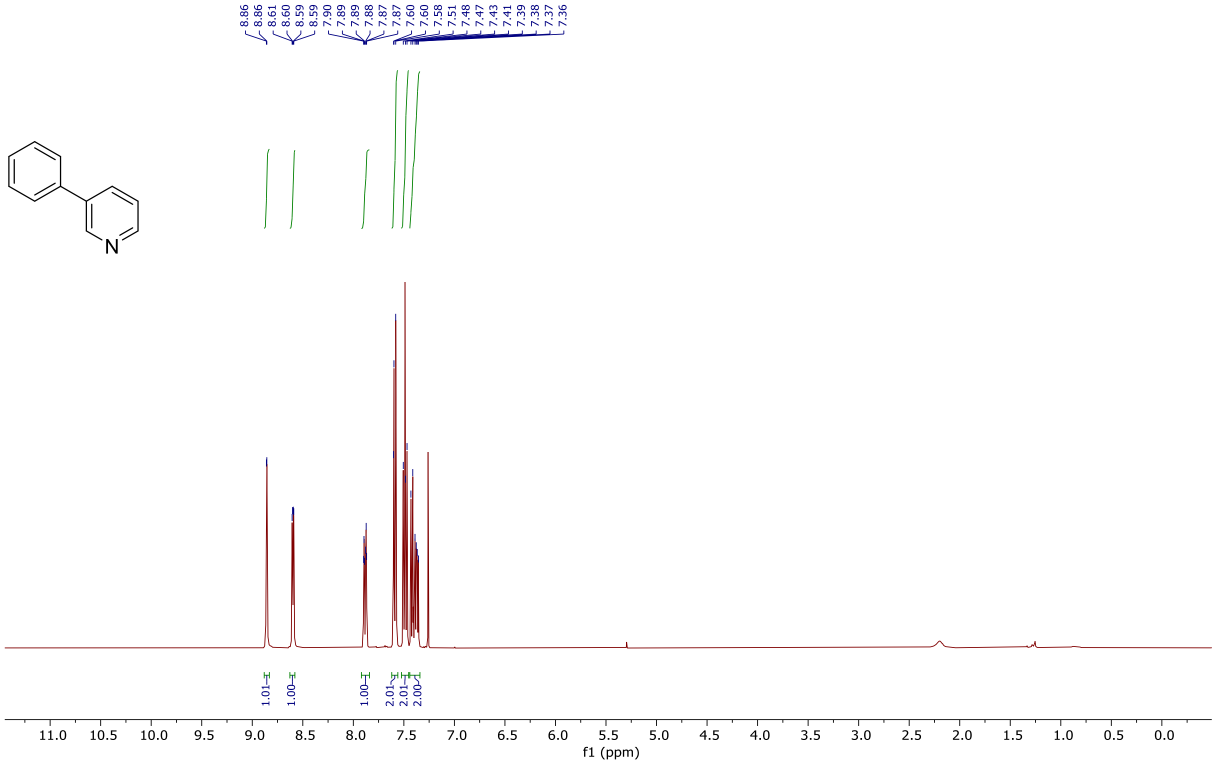

20210812-1632-B400_B.11-21.11.fid
Ref 664-2
Group Greaney_M
C13_CPD_Night256 CDCl3 /mnt/nmrdata/Greaney_M m31962tw 21

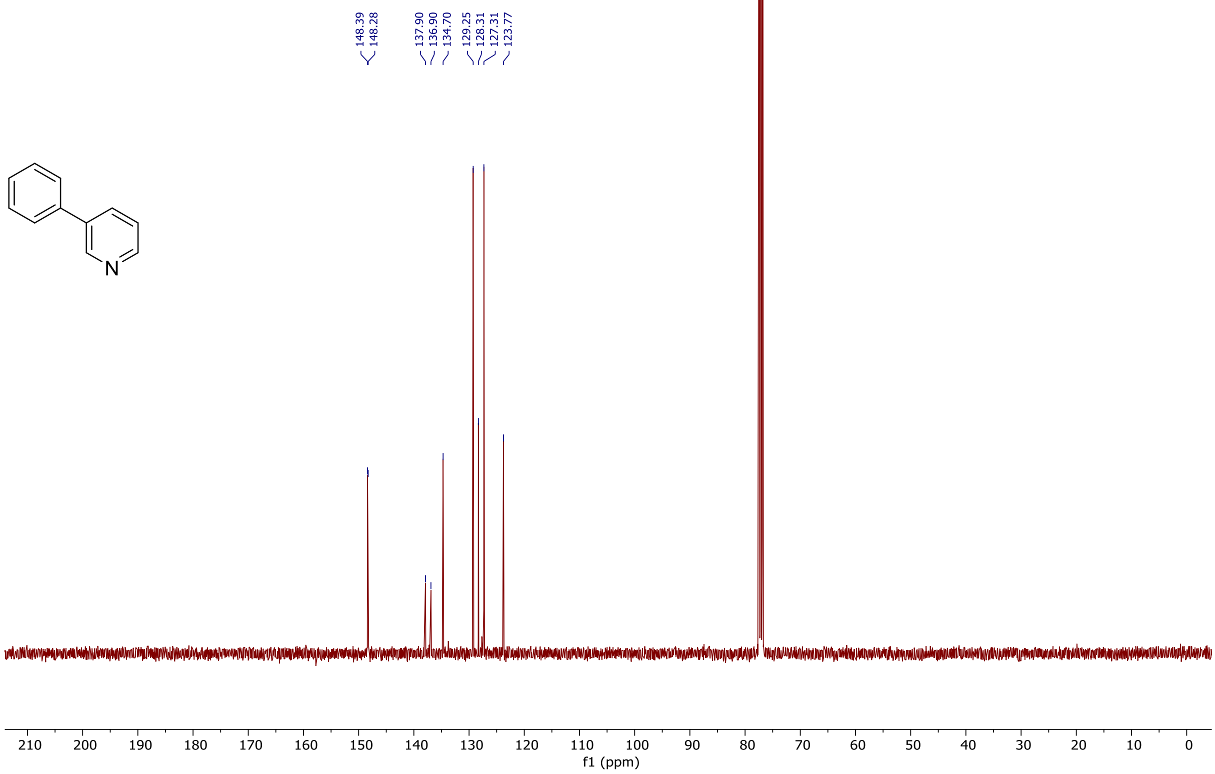

25

20211202-1743-B400_B.11-22.10.fid
Ref 746-6
Group Greaney_M
H1_Night CDCl3 /mnt/nmrdata/Greaney_M m31962tw 22

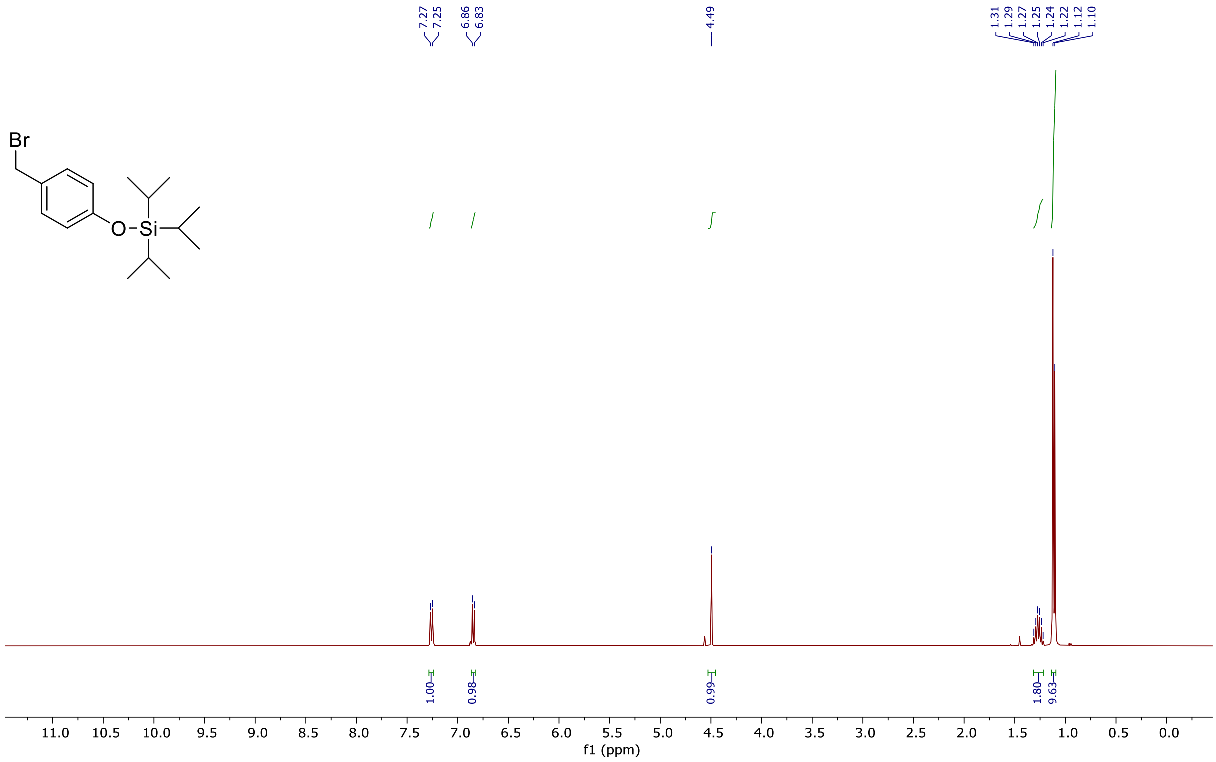

20211202-1743-B400_B.11-22.11.fid
Ref 746-6
Group Greaney_M
C13_CPD_Night256 CDCl3 /mnt/nmrdata/Greaney_M m31962tw 22

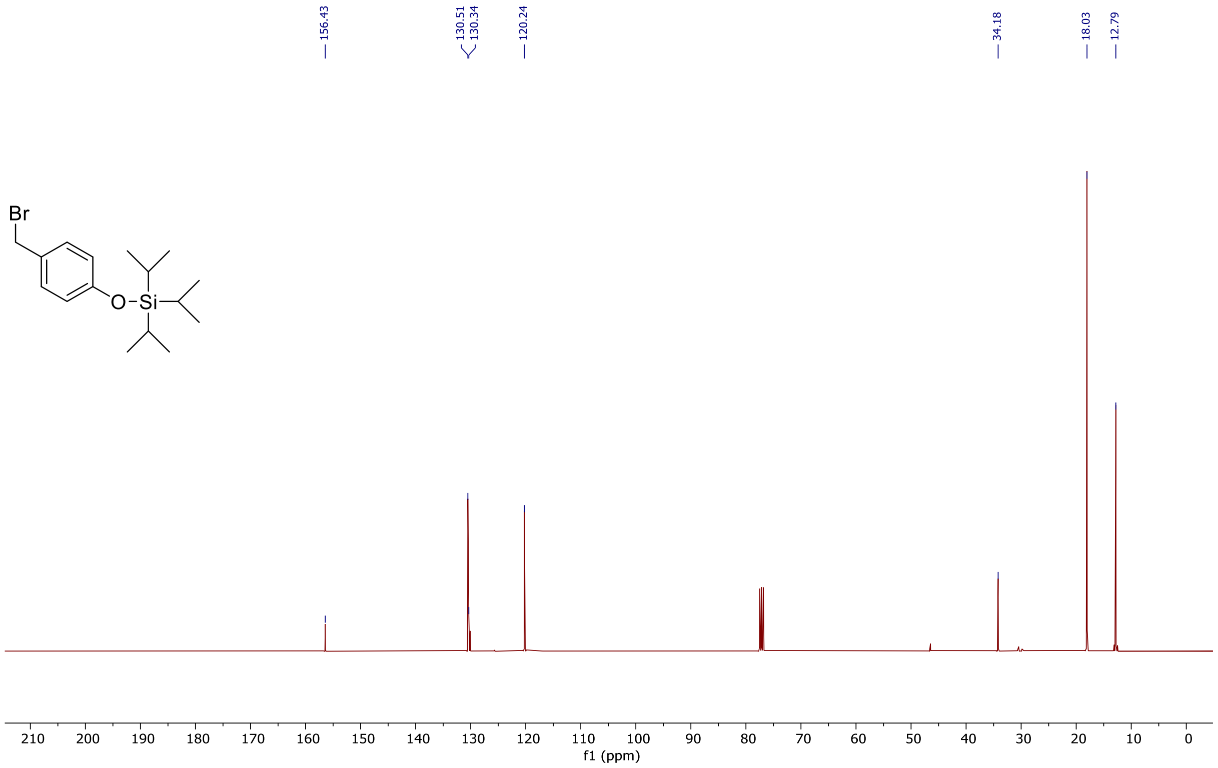

pre-NHC A

20210721-1009-B400_B.14-40.10.fid
Ref 427-1
Group Greaney_M
H1_Day MeOD /mnt/nmrdata/Greaney_M m31962tw 40

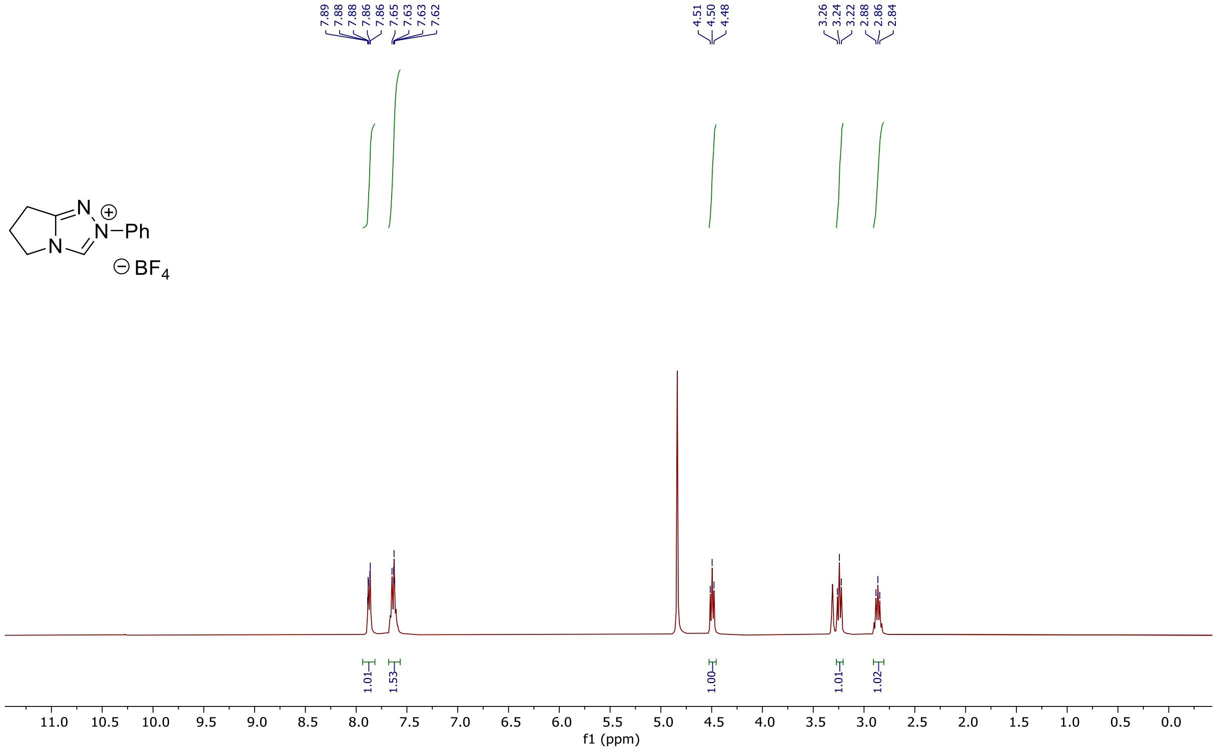

20210816-1814-B400_B.11-1.11.fid
Ref 427-1
Group Greaney_M
C13_CPD_Night256 MeOD /mnt/nmrdata/Greaney_M m31962tw 1

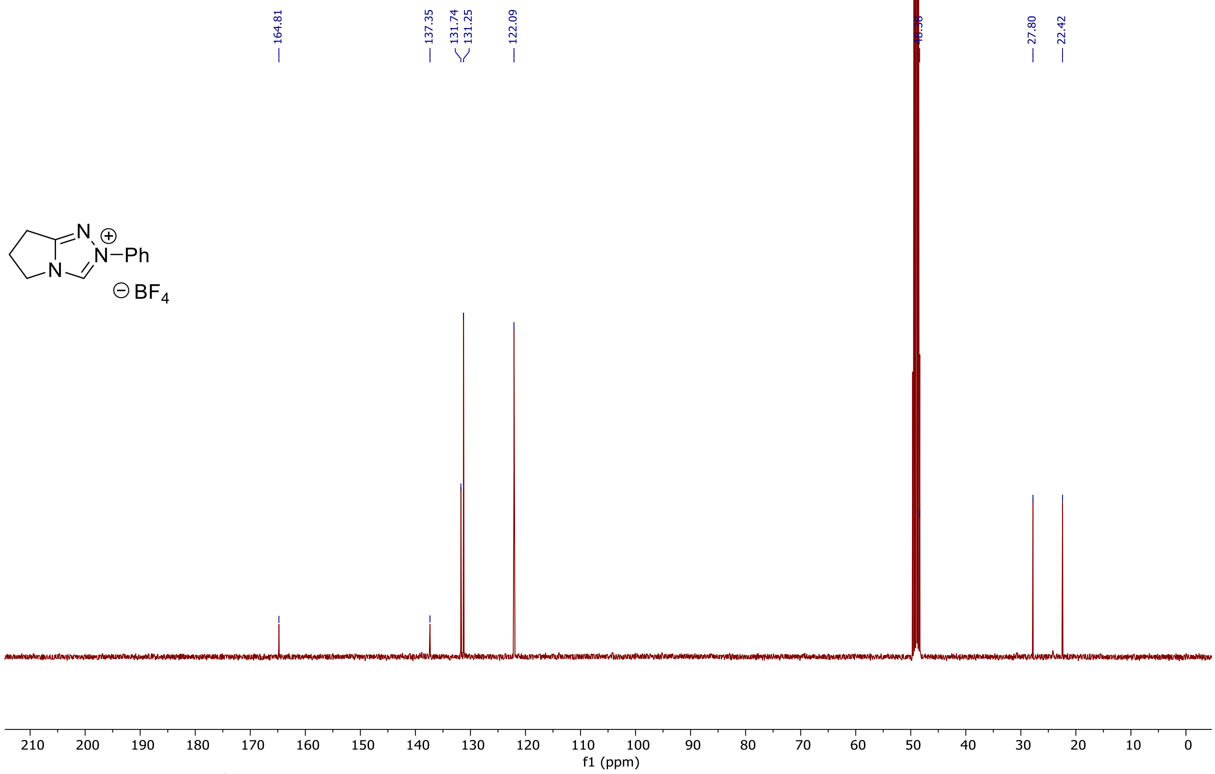

20210816-1814-B400_B.11-1.15.fid
Ref 427-1
Group Greaney_M
F19_NoCPD_Night MeOD /mnt/nmrdata/Greaney_M m31962tw 1

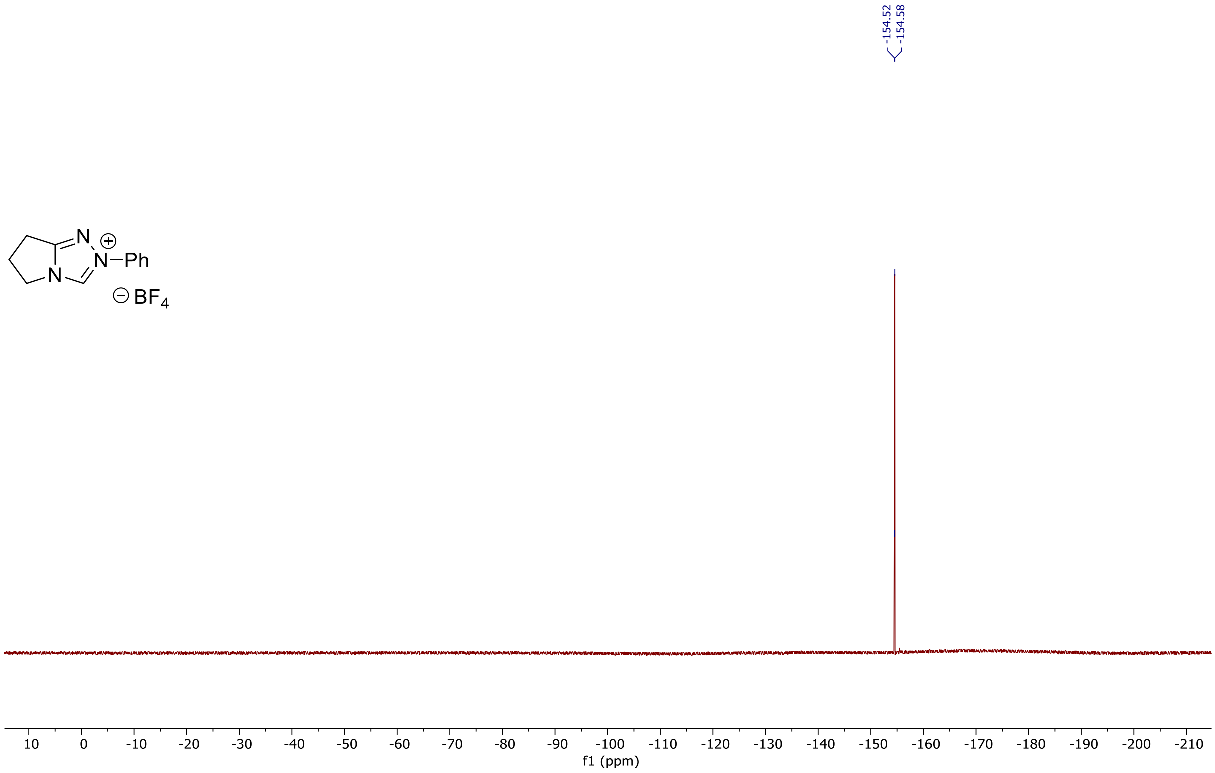

Supplement: Supplementary file 1 — Supporting Information [file ANIE-61-0-s001.pdf]
